# The Holistic Health Status of Chinese Homosexual and Bisexual Adults: A Scoping Review

**DOI:** 10.3389/fpubh.2021.710575

**Published:** 2021-08-24

**Authors:** Chanchan Wu, Edmond Pui Hang Choi, Pui Hing Chau

**Affiliations:** School of Nursing, LKS Faculty of Medicine, The University of Hong Kong, Hong Kong, China

**Keywords:** bisexual, Chinese, health, homosexual, men who have men with men

## Abstract

**Background:** Same-sex marriage is currently not legalized in China, despite the considerably large number of homosexual and bisexual Chinese populations. At the same time, their holistic health status remains unclear. This is the first scoping review conducted to comprehensively examine all the available literature and map existing evidence on the holistic health of homosexual and bisexual Chinese.

**Methods:** This scoping review used the framework of Arksey and O'Malley and followed the Preferred Reporting Items for Systematic Review and Meta-Analysis extension for scoping reviews (PRISMA-ScR). A comprehensive search strategy was carried out across 20 English (EN) and Chinese (both traditional and simplified) electronic databases from January 1, 2001, to May 31, 2020. Two reviewers conducted the reference screening and study selection independently and consulted a third senior reviewer whenever a consensus must be achieved. Data extraction was conducted using a structured data form based on the Cochrane template, after which a narrative synthesis of the findings was performed.

**Results:** A total of 2,879 references were included in the final analysis, with 2,478 research articles, 167 reviews, and 234 theses. Regarding the study populations, the vast majority of studies centered on men only (96.46%), especially men who have sex with men (MSM). Only 1.32% of the studies targeted female sexual minorities. The geographical distribution of all research sites was uneven, with most of them being conducted in mainland China (95.96%), followed by Hong Kong (2.05%), Taiwan (2.02%), and Macau (0.06%). Regarding the specific study focus in terms of the health domain, around half of the studies (45.93%) focused on sexual health only, and an additional quarter of the studies (24.15%) investigated both sexual health and social well-being. Meanwhile, the studies focusing on mental health only accounted for approximately 15% of the total.

**Conclusions:** This scoping review revealed that previous research focused more on male than female sexual minorities, on disease-centered surveys than person-centered interventions, and investigations on negative health conditions than positive health promotion. Therefore, investigations centered on the female sexual minorities and corresponding person-centered interventions are highly needed.

**Review Registration:** The protocol of this review has been registered within Open Science Framework (https://osf.io/82r7z) on April 27, 2020.

## Introduction

Homosexuality and bisexuality have long existed worldwide, but the recognition of same-sex marriage in many countries has only gradually occurred in recent years. In comparison, under the heavy influence of Confucianism, it has always been a traditional obligation of Chinese adults to bring offspring to the family. Thus, homosexuality is widely rejected by the Chinese and is considered not only a threat to the family but also a threat to society. For nearly 20 years (1979–1997), sex between men was considered illegal and criminalized as “hooliganism” (sodomy) in China until it was eliminated in 1997 (1). In 2001, homosexuality was no longer classified as a pathology under the Chinese Classification of Mental Disorders (2), marking a historical turning point in the progress of homosexuality in China. Despite such developments, in contemporary China, the mainland government still recognizes neither legal same-sex marriage nor civil unions, and the situations in Hong Kong and Macau are similar. In contrast, same-sex marriage has been legalized in Taiwan since 2019, even though the law was enacted outside the Civil Code (3). In general, homosexual and bisexual Chinese from the above Cross-Straits Four-Regions have experienced similar cultural and policy backgrounds in the past two decades. Thus, research on this population is of historical significance in such an era.

Compared to some Western countries where homosexuals could either cohabit or enter legal same-sex marriages when available (4), most Chinese homosexuals can only choose to either stay single or develop hidden relationships “in the closet,” and even fewer bisexual Chinese choose to disclose their sexual orientation (5). In recent decades, as research on these populations has gradually increased, some widely used behavioral concepts have been proposed by researchers to describe similar population groups regardless of their sexual identity (6–8), namely, men who have sex with men (MSM) and women who have sex with women (WSW). MSM/WSW populations may not only be involved in homosexual behaviors but also in bisexual behaviors. For instance, approximately 40% of MSM acknowledged being men who have sex with both men and women (MSMW) according to a national Chinese survey (9), while more than a quarter of the MSM claimed their sexual orientation to be bisexual (10). These indicate that research should not only focus on gays and lesbians from the perspective of sexual orientation but also on MSM and WSW from the perspective of sexual behavior.

According to the widely used definition of “health” introduced by the WHO, “*Health is a state of complete physical, mental and social well-being and not merely the absence of disease or infirmity”* (11). This definition explains the concept of “*holistic health*,” which is a broad conceptualization of health that encompasses various dimensions, including complete physical health, mental health, and social well-being. Notably, sexual health is the most prevalent health domain in studies targeting homosexual or bisexual Chinese. Specifically, such research has always focused on certain diseases, such as AIDS and the related topic of HIV prevention, or other Sexually Transmitted Infections (STI) and unsafe sexual behaviors. Specifically, the overall national prevalence of HIV among MSM was 5.7% from 2001 to 2018 (12), while that for syphilis for the same period was 11.8% (13). Thus far, only a few studies on Chinese WSW have been conducted compared to studies on MSM. According to the only domestic study investigating 224 Beijing WSW, 15.8% of this population were infected with gonorrhea though no HIV-positive cases were detected (14). Furthermore, about half of the WSW reported bleeding during or after sex, and many of them reported that they had experienced engaging in different kinds of high-risk sexual behaviors (15). All of these findings indicate that their worrying sexual health concerns may require further attention.

Although sexual minorities in China still face many significant psychosocial difficulties that are yet to be addressed, the most common of which is long-standing social discrimination or stigma based on sexual orientation (16), there are relatively a few studies on mental health and social well-being in this population compared to their counterparts in Western countries. In particular, both lesbians and gays in China reported feeling stressed and helpless in the face of expectations from society and their parents (17, 18). Moreover, both gay and bisexual Chinese men reported having suffered internalized homophobia (19, 20), which was found to be positively correlated with loneliness and negatively correlated with lower self-evaluation (19). At the same time, most psychosocial studies targeting sexual minorities were carried out in MSM populations from a behavioral perspective, with both qualitative and quantitative studies indicating that MSM often experienced homosexual stigma (21, 22) and HIV-related stigma (23). Furthermore, MSM in China reported significantly higher levels of internalized homophobia compared with those from outside China (24), with a mean score of 2.04 vs. 1.77, as measured by the 4-point Likert Internalized Homophobia Scale. Chinese MSM also reported experiencing a high prevalence of other mental health issues, such as loneliness (35.5%) (25), moderate-to-severe symptoms of depression (26.8–50.9%) (25–27), and anxiety (26.0–36.4%) (26, 27). Even worse are the high rates of suicide ideation and suicidal behavior. A study reported that the specific suicide ideation rates were 31% among gay and bisexual men in Taiwan (28) and 26% among MSM in nine cities of mainland China (29). Meanwhile, over 12% of MSM actually attempted suicide (29), which is several times higher than that of the normal adult men (30). All these indicate that many Chinese homosexuals and bisexuals suffer from poor mental health, which could lead to self-loathing and negative effects on their self-identity (31). These mental health issues could also have further negative effects on their sexual health and/or social well-being (32).

Currently, in China, there are no census data on either the homosexual or bisexual population. This has caused concerns, given that China is the most populous country in the world, which means that the number of sexual minorities could be proportionately higher compared with those in other countries. Furthermore, with the wide use of the Internet and increasing social tolerance, sexual minority groups are no longer as hidden as before; hence, their social and health needs should be understood and addressed. Nevertheless, prevailing public attitudes toward homosexuality remain negative. For example, over half (58.4%) of the MSM in Hong Kong reported experiencing public discrimination (33), similar to the situation in mainland China (34).

To date, there have been studies on homosexual and bisexual Chinese, especially within the MSM population. These studies mainly centered on STI-/HIV-related prevalence or prevention attempts (12, 35–43), or focused on human rights and the legalization of same-sex marriage, as conducted in the fields of sociology, anthropology, law, and psychology (44–46). However, other aspects of health and well-being have yet to be fully investigated. At the same time, there are even fewer studies on female sexual minorities compared with male ones (45), thus highlighting the need for further academic attention.

In summary, the current health-related research targeting Chinese homosexual and bisexual adults seem to be unbalanced from the perspective of the gender population and health domains, indicating the essential need for further scientific review evidence. So far, there is no systematically reviewed evidence available or ongoing review either in English (EN) or Chinese on the holistic health of homosexual and bisexual people within the Chinese context. In addition, the current evidence is difficult to summarize due to variations in the types of studies and the less precisely defined subjects and research variables.

In relation to the above, a scoping review, which is a type of systematic review, can be used to comprehensively map the known information about a topic based on the available information and then identify the potential gaps in the literature, thus facilitating an assessment of the state of knowledge about the specific topic (47–50). In 2018, the Preferred Reporting Items for Systematic Reviews and Meta-Analyses Statement was extended to Scoping Reviews (PRISMA-ScR) (51). Therefore, the current review was conducted as a systematic scoping review, following the PRISMA-ScR checklist (Supplementary Material 1). This review aims to comprehensively examine the literature to explore the breadth of current knowledge relating to the holistic health of homosexual and bisexual Chinese, identify potential knowledge gaps, and then inform future in-depth research on how to improve the health of this particular population.

## Methods

This study used the scoping review framework developed by Arksey and O'Malley (2005) (47) and further updated as recommended by the Joanna Briggs Institute (JBI) (50). This review was also conducted in accordance with a priori protocol currently under review (52), including five stages: 1) identification of the review questions, 2) identification of relevant studies, 3) study selection, 4) data extraction, and 5) summarization and reporting of the results.

### Stage 1: Identification of the Review Questions

The “PCC” mnemonic, representing Population-Concept-Context, is recommended by the JBI (53) as a guide to construct clear research questions for a scoping review. Correspondingly, in this review, “Population” refers to all Chinese homosexual and bisexual adults living in mainland China, Hong Kong, Macau, or Taiwan. Chinese sexual minorities who were born or living abroad were excluded due to policy and cultural differences. Furthermore, in this review, “Concept” refers to holistic health, a broad conceptualization of health defined by the WHO (11) that encompasses varied dimensions of health, including the complete physical, mental, and social well-being of an individual. This review, therefore, targeted all these health-related aspects. Finally, “Context” refers to the locations of study settings. Thus, following the overarching review question: “What is the holistic health status of Chinese homosexual and bisexual adults?” some detailed review questions are as follows: 1) “What health-related variables have been investigated about homosexual and bisexual Chinese?” 2) “What types of research have been conducted and which disciplines were most involved in carrying out studies targeting this population?” and 3) “What are the differences among the sample populations in terms of sexual orientation (between homosexuals and bisexuals) and gender (between male and female minorities)?”

### Stage 2: Identification of Relevant Studies and Search Strategy

The eligibility criteria for this scoping review using the PCC framework are shown in the a priori protocol. In addition to homosexual and bisexual people from the perspective of sexual orientation, MSM/MSMW and WSW groups were also included in terms of behavioral categories. Regarding the concept of holistic health, after reviewing health-related definitions (54–58), this review included both negative and positive variables related to mental health, physical (sexual) health, and social well-being. In terms of context, this review included studies conducted in all regions of China, including mainland cities, Hong Kong, Macau, and Taiwan. Regarding the inclusion criteria of the study types, all original studies using qualitative, quantitative, or mixed methods, reviews and published dissertations were included. Meanwhile, study protocols or blogs, book chapters, conference abstracts, research letters, editorial notes or commentaries were excluded.

For a comprehensive literature search, research articles, reviews, and theses published in 2001 or later were searched. This is because homosexuality has no longer been regarded as a mental illness in China since 2001 (2). All relevant databases in both EN and CN languages related to health care, psychology, and social science were searched. Specifically, 20 databases were searched, including 12 EN language databases (PubMed, Web of Science, CINAHL Plus, ScienceDirect, Social Work Abstracts, APA PsycInfo, etc.), four Simplified Chinese (SC) databases (China National Knowledge Infrastructure-CNKI, China Biological Medicine Database-SinoMed, etc.), and four traditional Chinese (TC) databases (Index to Taiwan periodical literature system, National Digital Library of Theses, Dissertation in Taiwan, etc.).

The search strategy for this review was adapted from the Peer Review of Electronic Search Strategies (PRESS) Evidence-Based Checklist (59). Handsearching was also used as a supplementary method, although unpublished documents were excluded from this review due to limited resources. Pilot searching was conducted in both EN (PubMed) and CN (SinoMed) language databases before the formal search, with the aim of identifying all relevant keywords or subject headings before finalizing the search strategy. The Medical Subject Headings (MeSH terms) and corresponding Chinese translation MeSH terms (CMeSH terms) are summarized in the Supplemental Material 2. The final search of the above mentioned databases was conducted throughout May 2020 and updated on May 31, 2020. Some updated studies were further reviewed on a monthly basis by checking the available email alerts (Supplementary Material 3).

### Stage 3: Study Selection

After searching, the identified records were exported to and managed by EndNote X9 (for EN and TC literature) and NoteExpress (for SC literature). Both software programs could automatically identify duplicate records. Then, two reviewers independently performed the study selection, which included title screening, abstract screening, and full-text screening according to the JBI guidelines (51, 53). Specifically, the title and abstract screening were carried out simultaneously referring to the PCC criterion, and the number of excluded references in each step was recorded and compared. In case of inconsistencies, the two reviewers discussed until data consistency was achieved. Afterward, the full texts of all potentially eligible references were retrieved for further screening, and disagreements on the study selection were resolved by a discussion between the two main reviewers and consultation with a third senior researcher. Finally, those references that were excluded during full-text screening were recorded following specific exclusion reasons, in line with the PCC framework (Supplementary Material 4).

### Stage 4: Data Extraction

Initially, a data extraction template was developed based on the Cochrane Data Extraction Template (60), after confirming all essential variables and key information to extract. Then, after conducting the pilot extraction using both qualitative and quantitative eligible studies, a revised and detailed version of the data extraction form was used. The data extraction was also conducted independently by two reviewers with regular discussion, and then continuously updated in an iterative manner. Specifically, the data extracted included specific details about the “Reference Characteristics,” “Study Characteristics,” and also “PCC-related Information,” including authors and affiliations, year of publication, study design, population and sample size, study settings, health domains, and corresponding findings.

### Stage 5: Summarization and Reporting of the Results

All retrieved information from the data extraction form was documented in Microsoft Excel, and narrative synthesis was used. The quantitative findings were descriptively summarized in the form of tables using frequencies and percentages. Next, the summarization of qualitative evidence including the collaboration network and co-word analysis was conducted *via* social network analysis by UCINET (61) and then visualized using the NetDraw program (62). The critical appraisal process is not necessary for a scoping review (48, 49), and it is also not feasible to evaluate the quality of each included reference (Supplementary Material 5). Nevertheless, this review tried to perform a quality evaluation of the journals in which all the included articles were published (Supplementary Material 6).

## Results

**Figure 1:**
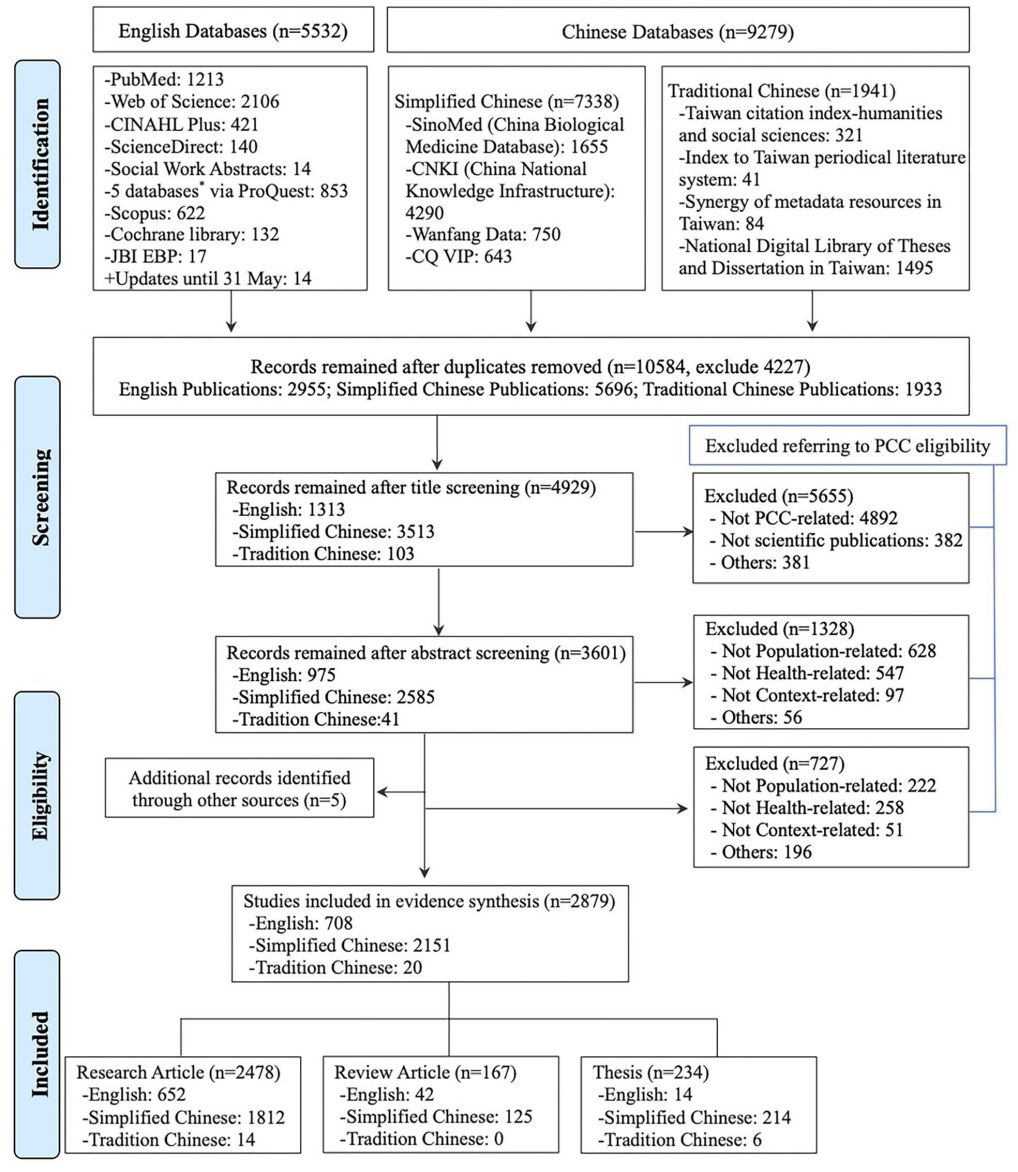
Search results and study selection process referring to Preferred Reporting Items for Systematic Review and Meta-Analysis (PRISMA). *: ProQuest searching of the 5 databases: ProQuest Dissertations & Theses A&I; ProQuest Dissertations & Theses Global; APA PsycInfo; Sociological Abstracts; Social Services Abstracts.

The final database search from January 1, 2001, to May 31, 2020, yielded a total of 14,811 references. After removing 4,227 duplicates, 10,584 references remained for further screening. Specifically, 5,655 were excluded after title screening, and 1,328 were excluded after abstract screening as they did not meet the PCC eligibility criteria. Of the 3,601 remaining records for full-text screening, 727 were excluded due to specific reasons (Supplementary Material 4). Finally, a total of 2,879 references incorporating an additional five references obtained through the manual search were included in the final analysis (Supplementary Material 5), including 2,645 articles and 234 theses. From the perspective of the publishing language, there were 708 EN references, 2,151 SC records, and 20 TC records. The study screening and selection were conducted following the PRISMA flow diagram (63). The detailed process and specific results are presented in Figure 1.

### Time Trends of Publications and Corresponding Studies

**Figure 2:**
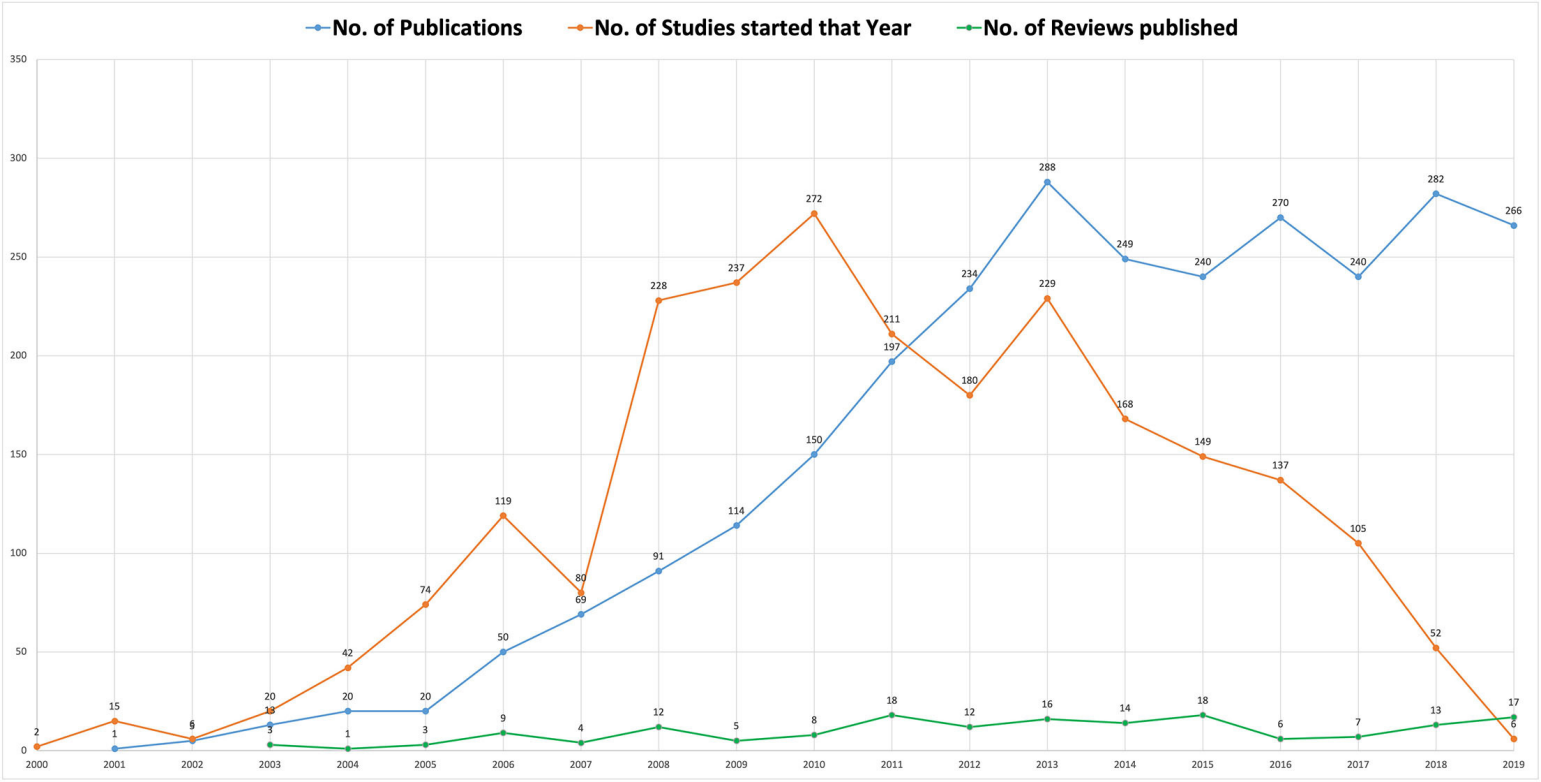
Time trends of publications, corresponding studies, and reviews. Since this study only included references published before May 2020, the data in 2020 was dropped as it would be distracting the trends.

**Figure 3:**
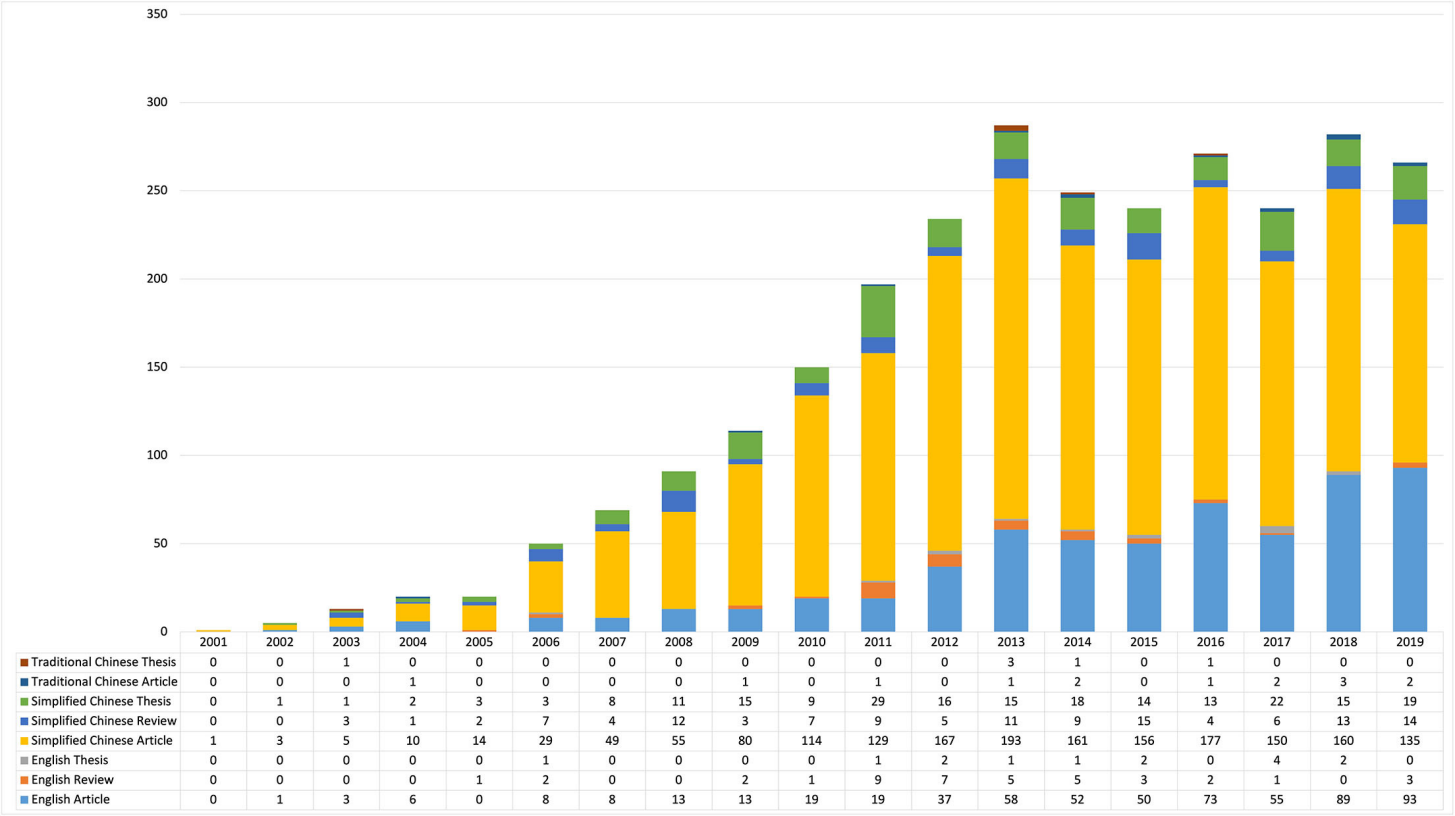
Publication distribution by study types per year (2001–2019). Since this study only included references published before May 2020, the data in 2020 was dropped as it would be distracting the trends.

Among all the 2,879 included references, the overall number of publications gradually increased and then stabilized in the past two decades (blue line in Figure 2). As this study only included references published before May 2020, the data in 2020 were dropped from both Figures 2, 3, so as not to distract the trends. Regarding the trend in the number of studies started each year over time, it can be seen that the research conducted before 2010 increased year by year, while the following decade (2011–2019) showed a significant downward trend (orange line in Figure 2). Review articles first appeared in 2003, and around 10 reviews were conducted every year thereafter without an obvious trend of increase or decrease (green line in Figure 2).

Among the 2,879 included publications, 74.71% (2,151) were written in SC and 86.07% (2,478) were research articles. Figure 3 shows the distribution of study types in both EN and CN publications. Among the 234 theses, some incorporated two or more independent studies (cross-sectional survey and following intervention study); thus a total of 276 identical studies were carried out.

### Author Affiliations and Funding Information

Regarding the distribution of author affiliations, the majority of affiliations (89.41%) were located in mainland China, of which more than half (54.74%) were affiliated with the Center for Disease Control and Prevention (CDC), and around a third (33.53%) were affiliated with universities, mainly medical universities (MUs). As for institutional cooperation, over half of the studies (55.24%) were conducted by a single institution; and approximately one-third of the research (30.40%) was carried out through domestic cooperation, which was more common in CN articles than in EN publications (29.38 vs. 1.02%, respectively). In contrast, the proportion of international cooperation among authors in EN publications exceeded that in CN publications (12.40 vs. 1.97%, respectively). Regarding the funding information, 36.68% (1,056/2,879), 1.84% (53), and 1.08% (31) of studies obtained funding from mainland China, Hong Kong, and Taiwan, respectively, while over one-third (37.93%) of studies were not funded. Furthermore, around 23% of the studies were funded by foreign organizations or global sources (Table 1), thereby revealing the global nature of these related studies.

**Table 1:** Institutional characteristics of 2,645 articles and 234 theses.

**Article institutions (***n*** = 2,645)**	**No. (%)**	**Thesis institutions (***n*** = 234)**	**No. (%)**
**First author affiliation**		**Degree**	
CDC	1,448 (54.74)	Master thesis	200 (85.47)
University	887 (33.53)	PhD thesis	34 (14.53)
Hospital	256 (9.68)	**Author affiliation**	
Scientific Institution	29 (1.10)	Mainland China	214 (91.45)
Health authority	10 (0.38)	Hong Kong	10 (4.27)
Others	15 (0.57)	Taiwan	6 (2.56)
**First author affiliation location**		Overseas	4 (1.71)
Mainland China	2365 (89.41)	**Universities**	
Overseas	144 (5.44)	中国疾病预防控制中心 (China CDC)	28 (11.97)
Hong Kong	85 (3.21)	安徽医科大学 (Anhui MU)	24 (10.26)
Taiwan	48 (1.81)	重庆医科大学 (Chongqing MU)	15 (6.41)
Macau	2 (0.08)	山东大学 (Shandong University)	11 (4.70)
Not available	1 (0.04)	The Chinese University of Hong Kong	10 (4.27)
**First author profession** ^a^		第三军医大学 (TMMU)	9 (3.85)
Public health	430 (63.70)	天津医科大学 (Tianjin MU)	9 (3.85)
Psychology	43 (6.37)	河北医科大学 (Hebei MU)	8 (3.42)
Global health	26 (3.85)	青岛大学 (Qingdao University)	8 (3.42)
Nursing	18 (2.67)	吉林大学 (Jilin University)	7 (2.99)
Sociology	18 (2.67)	昆明医科大学 (Kunming MU)	6 (2.56)
Medicine	11 (1.63)	新疆医科大学 (Xinjiang MU)	6 (2.56)
Psychiatry	10 (1.48)	中国医科大学 (China MU)	6 (2.56)
Not available	15 (2.22)	山西医科大学 (Shanxi MU)	5 (2.14)
Others	104 (15.41)	Others	82 (35.04)
**If involved nursing profession**		**Profession/Major**	
Yes	92 (3.48)	Epidemiology	112 (47.86)
No	2553 (96.52)	Public health	53 (22.65)
**First author is nursing profession**		Social Medicine	13 (5.56)
Yes	55 (2.08)	Psychology	10 (4.27)
No	2590 (97.92)	Dermatology	8 (3.42)
**Institution cooperation**		Sociology	6 (2.56)
International cooperation	380 (14.37)	Nursing	4 (1.71)
Domestic cooperation	804 (30.40)	Maternal, Child and Adolescent Health	4 (1.71)
Single institution	1461 (55.24)	Others	24 (10.25)
**Funding sources** ^b^		**Funding sources** ^b^	
Not funded/ Not available	1092 (37.93)	Global Funding	107 (3.72)
Mainland China	1056 (36.68)	Hong Kong	53 (1.84)
China and foreign funding	343 (11.91)	Taiwan	31 (1.08)
America	130 (4.52)	Funding from other organizations	67 (2.33)

a *Among 675 studies published in English (EN) (652 research papers and 23 studies from 14 theses)*.

b *Among all 2,879 publications*.

### Characteristics of the Included Reviews

Review articles accounted for the smallest proportion (167/2,645, 6.31%) of all articles, among which 25.15% (42/167) were published in EN and 74.85% (125/167) were published in SC. Most of the reviews centered only on the male population, especially MSM (151, 90.42%), while only four reviews focused on female sexual minorities (Table 2). These findings indicate the insufficient attention given to this population, thus highlighting the need for further research in this group. Notably, there was just one scoping review among all the included reviews, which summarized only the HIV prevalence and corresponding prevention intervention programs for MSM and transgender populations (64). Overall, the included reviews mostly focused on sexual health (86.83%), such as the incidence or prevalence of HIV/STI and related treatment. In comparison, less attention was given to mental health (7.19%) and social well-being (2.40%).

**Table 2:** Characteristics of the included reviews.

**Target population**	**English reviews**	**Chinese reviews**	**Total**	**%**
**Male only**	41	113	154	92.22%
MSM	40	111	151	90.42%
Gay	/	2	2	1.20%
MSMW	1	/	1	0.60%
**Female only**	1	3	4	2.40%
Lesbian	1	1	2	1.20%
WSW	/	2	2	1.20%
**Both gender**	0	9	9	5.39%
Gay & Lesbian	/	6	6	3.59%
MSM & WSW	/	3	3	1.80%
**Clinical people or not**				
HIV-infected	1	4	5	2.99%
Non patient	41	121	162	97.01%
**Review type**				
General literature review	11	111	122	73.05%
Systematic Review (SR)	6	2	8	4.79%
SR and Meta-analysis	24	12	36	21.56%
Scoping review	1		1	0.60%
**Review focus**				
Sexual health	40	105	145	86.83%
Mental health	1	11	12	7.19%
Social well-being	1	3	4	2.40%
Overall review or others	/	6	6	3.59%

### Characteristics of the Included Studies

There are 2,754 studies in all the included publications, including 2,478 research articles (1,812 SC, 14 TC, and 652 EN publications) and 276 studies from 234 theses. The majority of the studies (2,677, 97.20%) used the originally collected data, while the remaining ones were conducted as secondary analyses using previous data. Of all the original studies, nearly half (1,324, 48.46%) had sample sizes between 101 and 500; and 23.53% had sample sizes larger than 1,000, most of which involved series of cross-sectional surveys. Given that there were a certain number of published CN studies with unclear descriptions of the methodologies used, the detailed characteristics of the study design and sampling methods used data from the 675 published EN studies (652 research articles and 23 studies from 14 theses). Specifically, most of the studies (75.41%) were conducted as cross-sectional investigations, followed by qualitative studies (54, 8.00%); less than 1% were mixed-method studies (Table 3). Around a quarter of the studies used multiple sampling methods, followed by the Internet- and venue-based sampling methods.

**Table 3:** Study and sampling characteristics of included EN studies.

	**Mixed method study**	**Qualitative study**	**Quantitative study**	**Total**
**Sampling method**				
Multiple methods	1 (0.15)	12 (1.78)	160 (23.7)	173 (25.63)
Internet-based sampling	1 (0.15)	3 (0.44)	112 (16.59)	116 (17.19)
Venue-based sampling	/	5 (0.74)	97 (14.37)	102 (15.11)
Convenience sampling	/	4 (0.59)	82 (12.15)	86 (12.74)
Snowball sampling	2 (0.30)	9 (1.33)	65 (9.63)	76 (11.26)
Respondent Driven Sampling	2 (0.30)	/	62 (9.19)	64 (9.48)
Purposive sampling	/	18 (2.67)	6 (0.89)	24 (3.56)
Not available	/	/	13 (1.93)	13 (1.93)
Others	/	3 (0.44)	7 (1.04)	10 (1.48)
Random sampling	/	/	9 (1.33)	9 (1.33)
Time-Location Sampling	/	/	2 (0.30)	2 (0.30)
**Modality of recruitment**				
Offline	3 (0.44)	41 (6.07)	294 (43.56)	338 (50.07)
Mixed	2 (0.30)	8 (1.19)	172 (25.48)	182 (26.96)
Online	1 (0.15)	5 (0.74)	135 (20.00)	141 (20.89)
Not clear	/	/	14 (2.07)	14 (2.07)
**Sample size**				
1–50	/	49 (7.26)	1 (0.15)	50 (7.41)
51–100	/	5 (0.74)	14 (2.07)	19 (2.81)
101–200	1 (0.15)	/	39 (5.78)	40 (5.93)
201–300	1 (0.15)	/	68 (10.07)	69 (10.22)
301–400	/	/	87 (12.89)	87 (12.89)
401–500	/	/	97 (14.37)	97 (14.37)
501–600	/	/	67 (9.93)	67 (9.93)
601–700	/	/	29 (4.30)	29 (4.30)
701–800	/	/	15 (2.22)	15 (2.22)
801–900	/	/	20 (2.96)	20 (2.96)
901–1000	2 (0.30)	/	14 (2.07)	16 (2.37)
>1000	2 (0.30)	/	164 (24.3)	166 (24.59)
**Data collection person**				
Researcher	1 (0.15)	52 (7.70)	252 (37.33)	305 (45.19)
Participant	1 (0.15)	/	259 (38.37)	260 (38.52)
Researcher or Participants	4 (0.59)	2 (0.30)	75 (11.11)	81 (12.00)
Not clear	/	/	29 (4.30)	29 (4.30)
**Data collection method**				
Questionnaires	1 (0.15)	1 (0.15)	388 (57.48)	390 (57.78)
Laboratory tests & questionnaire	/	/	213 (31.56)	213 (31.56)
Interviews	/	43 (6.37)	2 (0.30)	45 (6.67)
Laboratory tests	/	/	10 (1.48)	10 (1.48)
Questionnaires & interviews	5 (0.74)	/	/	5 (0.74)
Focus Group Discussion (FGD)	/	5 (0.74)	/	5 (0.74)
Interviews & FGD	/	4 (0.59)	/	4 (0.59)
Others/ Multiple approaches	1 (0.15)	1 (0.15)	1 (0.15)	3 (0.45)
**Total**	*6 (0.89)*	*54 (8)*	*615 (91.11)*	*675 (100)*

### Population Information

Among all the 2,879 publications, the vast majority of the research, whether original studies or reviews, focused on MSM (2,667, 92.64%). Specifically, in the 675 EN publications with detailed population descriptions, most average ages ranged between 20 and 35 years. There was just one study that targeted bisexual people only (65), while over 40 studies involved homosexuals only. In addition, in all studies that indicated specific sexual orientation, the proportion of homosexuals was greatly higher than that of bisexuals (Table 4). Nearly two-thirds of the studies collected data on the marital status of the sample populations (around 10–30% were married). Thus, the marital and living conditions of homosexuals and bisexuals, along with their spouses, are worthy of further exploration.

**Table 4:** Population characteristics of all included references.

**Population characteristics**	**No. (%)**	**Characteristics**	**No. (%)**
**Target population^a^**		**Gender^a^**	
MSM	2,667 (92.64)	Male only	2,777 (96.46)
Gay	66 (2.29)	Both gender	60 (2.08)
Gay & Lesbian	33 (1.15)	Female only	38 (1.32)
Homosexual & bisexual Male	28 (0.97)	Other (include transgender)	4 (0.14)
General homosexual & bisexual	21 (0.73)	**Whether patients or not^a^**	
Lesbian	19 (0.66)	Non patients	2,626 (91.21)
WSW	15 (0.52)	HIV-infected people	241 (8.37)
MSMW	10 (0.35)	Others (Syphilis)	12 (0.42)
Bisexual Male	6 (0.21)	**Orientation^b^**	
MSM and WSW	6 (0.21)	All homosexual	40 (5.93)
Homosexual & bisexual Female	4 (0.14)	All bisexual	1 (0.15)
Others (LGBTQ+)	4 (0.14)	**Homosexual rate**	
**Mean years of age^b^**		<40.0%	9 (1.34)
<20.0	1 (0.15)	40.0–49.9%	26 (3.85)
20.0~29.9	226 (33.48)	50.0–59.9%	50 (7.41)
30.0~39.9	100 (14.81)	60.0–69.9%	82 (12.15)
≥40.0	4 (0.89)	70.0–79.9%	119 (17.63)
NA	342 (50.67)	≥80.0%	80 (11.85)
**Marriage rate (ever)^b^**		NA	268 (39.70)
0–9.9%	73 (10.81)	**Bisexual rate**	
10.0–19.9%	183 (27.11)	<20.0%	82 (12.15)
20.0–29.9%	104 (15.41)	20.0–29.9%	107 (15.85)
30.0–39.9%	47 (6.96)	30.0–39.9%	48 (7.11)
40.0–59.9%	30 (4.44)	40.0–49.9%	21 (3.11)
60.0–88.6%	12 (1.78)	50.0–59.9%	6 (0.89)
All married	3 (0.44)	≥60.0%	2 (0.30)
NA	223 (33.04)	NA	368 (54.52)

a *Data from all 2,879 publications*;

b *Data from 675 studies published in EN only*.

### Context Information

Among all 2,712 research references (2,478 research articles and 234 theses), 2,704 provided clear setting information, including 3,310 study sites (both single and multiple sites), although the geographical distribution of all study sites was uneven. Specifically, when analyzing all study sites from publications in both languages, 95.96% of the studies were conducted in mainland China, followed by Hong Kong (2.05%), and Taiwan (2.02%); meanwhile, only two nationwide studies (66, 67) involved Macau (0.06%), and no original single-site study was conducted in Macau. Furthermore, when analyzing study sites in EN publications, similarly, the majority of studies (83.41%) were conducted in mainland China, followed by Hong Kong (8.89%) and Taiwan (6.52%). However, when considering the geographical area or population size, the results showed that more studies were conducted per unit area or population in Hong Kong, compared with Taiwan, the mainland, and Macau (in descending order). In all the research conducted in mainland China, study sites were mostly located in the east (841, 25.41%) and southwest (742, 22.42%). Three places that had the highest number of studies were Sichuan Province (383, 11.57%), Guangdong Province (316, 9.55%), and Beijing Municipality (286, 8.64%).

In addition, this review attempted to provide an economic description of the study sites in terms of gross domestic product (GDP). The results showed that all study sites can be categorized into three groups, “Economically developed areas” [per capita GDP exceeding CNY¥100,000, Chinese Yuan (CNY)], “Economically moderate areas” (per capita GDP between CNY¥50,000 and CNY¥100,000), and “Economically underdeveloped areas” (per capita GDP less than CNY¥50,000). Most of the studies were conducted in economically moderate or developed areas (57.43 vs. 32.57%, respectively), while only 10% were carried out in economically underdeveloped areas, indicating the insufficient attention given to relatively poor places. Regarding the specific study settings, over half of the studies (63.85%) were conducted offline [usually in CDCs, some gay communities, or through non-governmental organizations (NGOs)], while around one-fifth of the studies (14.81%) were conducted online *via* Internet websites (Table 5).

**Table 5:** Context characteristics of all included references.

**Context characteristics**	**No. (%)**	**Context characteristics**	**No. (%)**
**Single or multi-sites**		**Study conducted setting^a^**	
Single site	2,251 (83.00)	***Offline***	
Multi-sites	453 (16.70)	Offline-CDC	92 (13.63)
Not clear	8 (0.29)	Offline-Clinic	66 (9.78)
**Geographical division of all sites^b^**		Offline-Gay community	81 (12.00)
*Hong Kong*	68 (2.05)	Offline-NGO	33 (4.89)
*Taiwan*	67 (2.02)	Offline-Not clear	158 (23.41)
*Macau*	2 (0.06)	Offline-University	1 (0.15)
*Mainland*	*3,173 (95.86*)	***Online***	
East China	841 (25.41)	Online-Application (App)	5 (0.74)
Southwest China	742 (22.42)	Online-Internet	100 (14.81)
South China	445 (13.44)	Online-Internet & App	27 (4.00)
North China	436 (13.17)	Online-Not clear	3 (0.44)
Central China	269 (8.13)	Online-Telephone	10 (1.48)
Northeast China	252 (7.61)	***Online & Offline***	54 (8.00)
Northwest China	188 (5.68)	Not mentioned	45 (6.67)
**Geographical division of English publications^a^**		**Site division according to economic status^a^**	
Mainland	563 (83.41)	Economically developed areas	1,078 (32.57)
Hong Kong	60 (8.89)	Economically moderate areas	1,901 (57.43)
Taiwan	44 (6.52)	Economically underdeveloped areas	331 (10.00)
Multi-sites	8 (1.19)		

a *Data from 675 studies published in EN only*.

b *Data from all studies mentioning the study sites (3,310 sites in total)*.

c *According to the Gross Domestic Product (GDP) per capita, “Economically developed areas” include Hong Kong, Macau, Taiwan, Beijing, Shanghai, Jiangsu, Fujian, Tianjin, and Zhejiang; “Economically moderate areas” include Guangdong, Chongqing, Hubei, Shandong, Inner Mongolia, Shaanxi, Anhui, Hunan, Liaoning, Sichuan, Jiangxi, Henan, Hainan, Ningxia, Xinjiang, Xizang, Yunnan, Qinghai, Jilin, and Shanxi; and “Economically underdeveloped areas” include Hebei, Guizhou, Guangxi, Heilongjiang, and Gansu*.

### Concept Information

**Figure 4:**
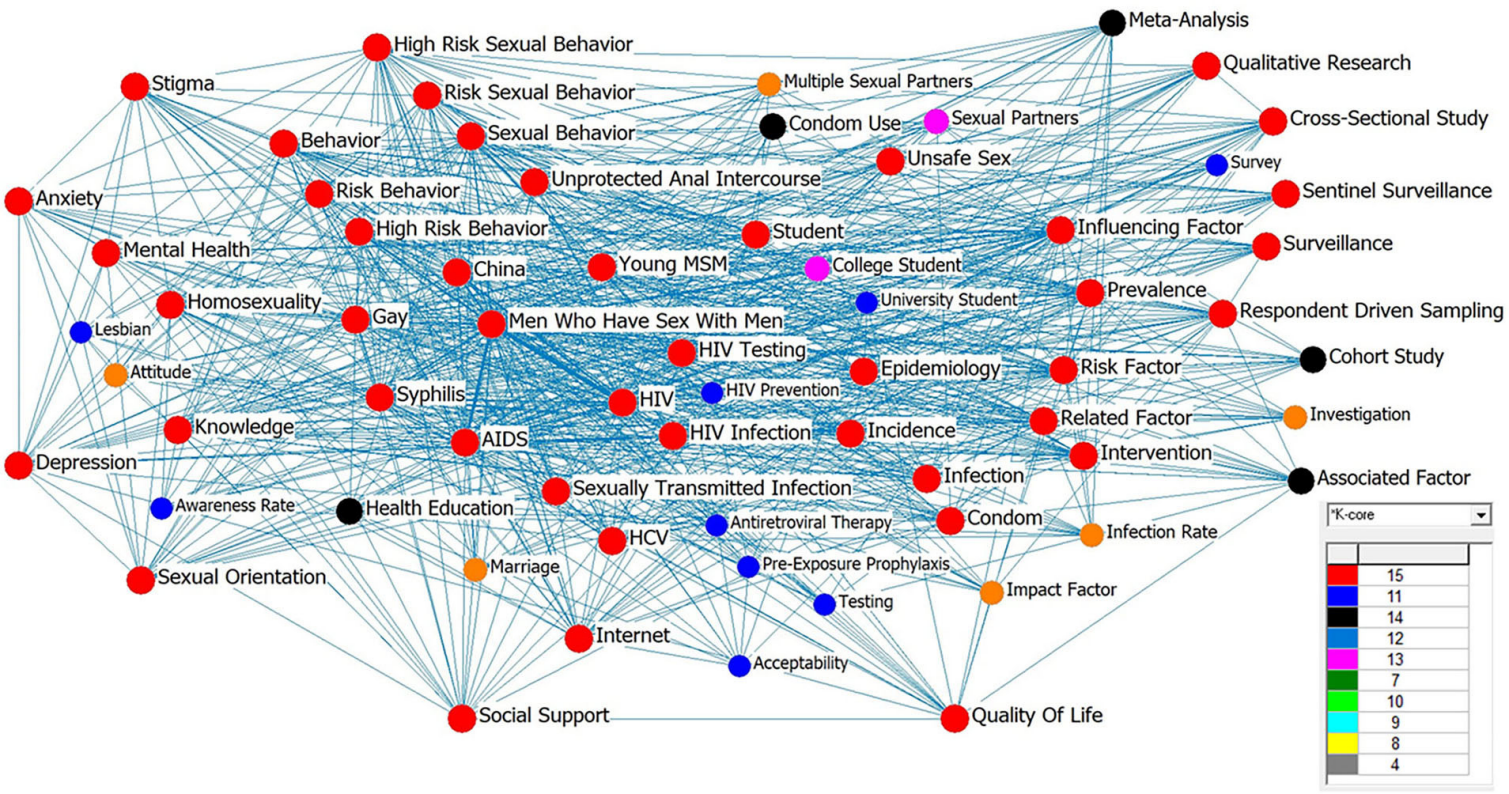
Co-word analysis of all English (EN) keywords and translation of Chinese ones. The colors and lines were automatically generated after K-core analysis, specifically, the larger the number corresponding to the color, the higher the frequency of co-occurrence; the thicker the line, the stronger the degree of co-occurrence.

Among all keywords that appeared in the included publications, “MSM” had the highest frequency as population variables in both CN and EN publications, followed by some sexual health-related concepts (e.g., HIV, STI, and sexual behaviors) and concepts related to mental health (e.g., stigma and depression), as shown in Supplementary Material 7. However, the concept of “social well-being” or other positive health-related concepts did not appear frequently. Co-word analysis was conducted to map the relationships among all keywords using UCINET and NetDraw (61), in which a higher number of co-occurrences indicated a closer relationship between the two keywords, as shown in Figure 4. The colors and lines were automatically generated after K-core analysis, specifically, the larger the number corresponding to the color, the higher the frequency of co-occurrence; the thicker the line, the stronger the degree of co-occurrence. Correspondingly, the results showed that “MSM” was the keyword with the highest frequency (1,908 times), co-occurring most frequently with other keywords. This was followed by “HIV” and “AIDS.” In addition, HIV-/STI- and sexual health-related words were also high-frequency keywords. At the same time, keywords, such as “University students” appeared, indicating that young people were gaining increasing research attention. However, the frequency of the keyword “Lesbian” was very low, and that of “Bisexual” was even lower. Overall, male- and sexual health-related keywords appeared more frequently than female- and other health-related variables.

In addition, this review extracted the specific foci of 675 published EN studies, which had clear descriptions of the study variables they used. Among them, around half (45.93%) focused on sexual health only, and an additional quarter (24.15%) investigated both sexual health and social well-being. In comparison, the total number of studies focused on mental health accounted for only about 15%, whether it was the only variable in the research or one of the variables used (Table 6). Although the number of studies conducted for different genders varied greatly (627 vs. 16), the comparison revealed that male-related research focused more on sexual health (HIV/STI), while female-related research was relatively more concerned with mental health (25.00 vs. 3.35%). Furthermore, four studies investigated breast-related health among female sexual minorities (68–71), all of which were conducted in Taiwan.

**Table 6:** Study focus and gender differences in 675 studies published in EN.

**Study focus**	**Female (% in 16)**	**Male(% in 627)**	**Both gender** **(% in 32)**	**Total** **(% in 675)**
Sexual health	2 (12.50)	307 (48.96)	1 (3.13)	310 (45.93)
Sexual health, Social well-being	/	160 (25.52)	2 (6.25)	163 (24.15)
Mental health, Social well-being	3 (18.75)	36 (5.74)	10 (31.25)	50 (7.41)
Sexual health, Mental health	/	43 (6.86)	3 (9.38)	46 (6.81)
Social well-being	3 (18.75)	33 (5.26)	8 (25.00)	44 (6.52)
Mental health	4 (25.00)	21 (3.35)	7 (21.88)	32 (4.74)
Holistic health	/	25 (3.99)	/	25 (3.70)
Breast health	4 (25.00)	/	/	3 (0.44)
Other physical health	/	2 (0.32)	1 (3.13)	3 (0.44)
***Total***	16	627	32	675

This review also extracted all the health-related variables and corresponding measurement tools mentioned in the 675 studies published in EN. In comparison, less than a quarter of the studies clearly stated that they used validated scales (19.48%) or self-developed tools (4.73%), most of which reported their reliability or validity. Specifically, the most studied variables were still sexual health-related, such as sexual behavior and HIV screening (Table 7). The word clouds (Supplementary Material 7) also showed comparisons of variables investigated among the 16 female-specific studies and 627 male-specific studies.

**Table 7:** Specific health-related variables and corresponding measurements.

**Concept characteristics**		**No. of studies**	**Percentage**
**Specific variables (**≥30**)**	**Health domain**		
General sexual behavior	Sexual health	218	32.30%
HIV screening	Sexual health	107	15.85%
Condom use	Sexual health	94	13.93%
HIV testing behavior	Sexual health	78	11.56%
Syphilis screening	Sexual health	69	10.22%
Drug use	Social well-being	65	9.63%
Depression	Mental health	64	9.48%
HIV/AIDS knowledge	Sexual health	58	8.59%
HIV prevalence	Sexual health	54	8.00%
Risky behavior^b^	Sexual health	44	6.52%
Social support	Social well-being	33	4.89%
Syphilis prevalence	Sexual health	31	4.59%
Unprotected anal intercourse	Sexual health	31	4.59%
Anxiety	Mental health	30	4.44%
**Measurements of all specific variables**			
Not mentioned		1399	60.15%
Scales/Questionnaires^a^		453	19.48%
Laboratory tests		340	14.62%
Self-developed scales^a^		110	4.73%
Not available		24	1.03%
**Validation of measurements** ^**a**^			
Yes		468	83.13%
No		95	16.87%

a *Validation of those measurement tools explicitly mentioned in the study*.

b *“Risky behavior” refers to different types of high-risk behavior, such as multiple sex partners, one-night stands, alcohol abuse, or other uncategorized behaviors that may cause health hazards*.

## Discussion

To the best of our knowledge, this is the first scoping review conducted to synthesize the holistic health of homosexual and bisexual populations in mainland China, Hong Kong, Macau, and Taiwan. So far, it is also the most comprehensive systematic scoping review, with the highest number of included literature published in both EN and CN. Specifically, this review included all scientific literature published from January 2001 to May 2020, since homosexuality was removed from the list of mental illnesses in China in 2001 (2). The trends of all publications showed that research on related minorities increased significantly year by year, reaching a peak in 2013, and then gradually stabilized in the following years with some fluctuations. However, when observing the trend of new studies carried out every year, the trend of a gradual decrease in research over the past decade (2011–2020) is evident, indicating that research attention on this population is gradually decreasing. Considering the reality that more and more sexual minorities are choosing to “come out” (72, 73), thus decreasing trend is a cause for concern and warrants further academic attention.

In response to the core review question in line with the PCC framework, this review found that there are far more studies on homosexuals than on bisexuals and other sexual minorities. In particular, the attention given to the male population was much higher than that to female groups (96.46 vs. 1.32%, respectively), especially in the most extensive MSM-related publications (92.64%). Regarding specific health-related concepts, there were far more studies on sexual health than investigations of mental health or social well-being (Table 7). In particular, the HIV-/STI-centered research was more common, with the concerns of scholars gradually evolving from HIV/STI prevalence (41, 74–76), treatment (77, 78), and corresponding adherence (79, 80) toward various prevention approaches, including both professional-led (81, 82) and self-testing/self-care programs (83–85). However, despite the prevalence of disease-centric research, less attention was paid to positive health concepts, such as sexual satisfaction, with only one survey conducted in the male group (86) while no relevant investigations for female minorities. All the above findings suggest that more research on female sexual minorities is needed in the future, whether research on mental health or the positive aspects of sexual health.

Specifically, there was an increased prevalence of HIV, syphilis, and gonorrhea among MSM (12, 87, 88). Although HIV was not detected in the female population in the existing studies, other diseases were relatively common, such as gonorrhea, chlamydia, and bacterial vaginosis (14), all indicating a worrying trend in sexual health in these sexual minorities. In terms of mental health, homophobia was universally experienced by gay men (19, 20), along with helplessness and other stressful feelings, which were also common among general homosexuals (17, 18). These may have further negative effects on their well-being directly or indirectly (32). Regarding social well-being, there were a certain number of investigations on the quality of life of general MSM (89, 90), MSMW (91), and HIV-infected MSM (92–94); and their perceived or received social support (95–99). These findings suggest that social support, which could serve as a protective factor against mental issues, should be further enhanced. While there were no such studies targeting female sexual minorities, similar studies among this relatively unnoticed population should be conducted to investigate and improve their quality of life.

In addition, this review provided some other novel discoveries that have not yet been reported. First, the comparison of the cooperation between author institutions in CN and EN language journals showed greater international cooperation when publishing EN articles and greater domestic cooperation when publishing CN ones. This finding can be attributed to factors such as the language advantage and increasing international exchanges and cooperation of scholars. Second, the distribution of the profession of authors showed that the most common academic major was public health (63.7%), followed by psychology (6.4%), global health (3.9%), and nursing (2.7%). This indicates that scholars in the field of public health have relatively more experience, insights, and contributions to research on the health of sexual minorities. This finding also serves as a reminder for researchers in other disciplines to take the initiative to carry out relevant research. Third, regarding the dynamic development of sampling methods, with the increased visibility of these minority populations and the popularization of the Internet, sampling methods have gradually transitioned from using a venue-based approach to using multiple methods. Thus, more recent surveys now rely on the Internet or smartphone applications. Correspondingly, many new investigation approaches and more innovative intervention projects have been promoted, such as crowdsourcing interventions to promote HIV/STI prevention (100, 101) and the use of Internet popular opinion leaders (iPOL) interventions that rely on online peer support (102). This finding suggests that academics should carry out research with a developmental and more person-centered perspective to better promote health outcomes in these populations.

This review has some implications for relevant stakeholders regarding the holistic health improvement of homosexual and bisexual Chinese. First, for health service providers, especially medical staff in the field of public health, their gender, and sexuality literacy should be improved, as they are the direct providers of health care. Therefore, it is recommended that health professionals learn relevant knowledge so as to provide more diverse and professional advice. In addition, health-related institutions need to be as open and diversified as possible to provide more health services and increase health care accessibility, allowing more sexual minorities to actively seek help to a greater extent. Second, studies have shown that community engagement can help with health improvement by enhancing knowledge dissemination and facilitating testing (22, 103), thus highlighting the importance of a diverse and supportive community environment. Therefore, for social service providers, it is recommended that they proactively reach out to help and provide more social and psychological information to eliminate homophobia, biphobia, and other forms of discrimination. At the same time, all service providers should consciously participate in multidisciplinary cooperation to better provide person-centered holistic health care that can empower all sexual minorities to take pride in their own health and encourage them to maintain healthy behaviors. Finally, compared to studies on various general populations under the heteronormativity context, there are very few studies on sexual minorities. Thus, there is an urgent need for researchers or scholars from different disciplines to conduct research from different perspectives, with the common goal of helping everyone express their true selves and lead healthy lives.

This review has several limitations that should be addressed. First, despite the inclusion of numerous references in both languages, it is still difficult for a single review to accurately conclude the holistic health status of homosexual and bisexual Chinese from a comprehensive perspective. Due to the uneven quality of the included literature (as described in Supplementary Material 6), this review only summarized the objective demographic data (population and context information) from all the publications. While the summarization of concept information and study characteristics was based on the data extracted from EN publications only, most of which were peer-reviewed. Second, although this review attempted to conduct quality assessments of all included journals, nearly a quarter of the included articles were published in relatively low-quality journals, thereby leading to a potential limitation. Third, this review only included studies conducted in mainland China, Hong Kong, Macau, and Taiwan, while studies on overseas Chinese were excluded due to different social ideologies and policy backgrounds. This could mean that the findings were less representative of all homosexual and bisexual Chinese worldwide. Further comparison studies among the Chinese in various regions can be carried out in the future. Finally, this review involved a large number of references; hence, the findings might not be sufficiently population-specific or health-specific. Thus, more precise reviews or more targeted studies are needed in the future.

## Conclusions

This scoping review summarized all the literature on the holistic health of homosexual and bisexual Chinese from 2001 to 2020. Existing evidence showed that previous research focused more on male than female sexual minorities, on HIV-/STI-centered surveys than person-centered investigations, on CDC-led interventions than crowdsourcing programs, and negative health conditions than positive health promotion. Thus, more investigations centered on the female population and following person-centered interventions are highly needed, along with the implementation of health promotion programs for this population group. Furthermore, projects that increase community engagement should be carried out among different communities, whether in communities based on gender or sexual orientation. Finally, researchers with more multidisciplinary backgrounds need to participate in the research on sexual minorities. In particular, there should be greater involvement of professionals from different disciplines to deliver adequate health care and social care services for Chinese homosexuals and bisexuals.

## Data Availability Statement

The original contributions presented in the study are included in the article/Supplementary Material, further inquiries can be directed to the corresponding author/s.

## Author Contributions

CW and EPHC conceptualized this review and designed the research questions. CW prepared and drafted the manuscript. CW and EPHC performed the initial screening of the articles and were involved in the development of the data extraction form. CW piloted the data extraction form and conducted the data extraction with EPHC independently. CW carried out data analysis under the guidance of EPHC. EPHC and PHC are the guarantors and have contributed to the critical revision of the manuscript. All the authors have read and approved the final manuscript.

## Conflict of Interest

The authors declare that the research was conducted in the absence of any commercial or financial relationships that could be construed as a potential conflict of interest.

## Publisher's Note

All claims expressed in this article are solely those of the authors and do not necessarily represent those of their affiliated organizations, or those of the publisher, the editors and the reviewers. Any product that may be evaluated in this article, or claim that may be made by its manufacturer, is not guaranteed or endorsed by the publisher.

## Abbreviations

WHO: World Health Organization
MSM: Men/Males who have Sex with Men/Males
WSW: Women who have Sex with Women
MSMW: Men who have Sex with both Men and Women
HIV: Human Immunodeficiency Virus
AIDS: Acquired Immune Deficiency Syndrome
STI: Sexually Transmitted Infections
JBI: Joanna Briggs Institute
PRISMA: Preferred Reporting Items for Systematic Reviews and Meta-Analyses statement
PRISMA-ScR: Preferred Reporting Items for Systematic Review and Meta-Analysis Extension for Scoping Reviews
PRISMA-P: Preferred Reporting Items for Systematic Reviews and Meta-analysis Protocols
PCC: Population Concept Context
MeSH: Medical Subject Headings
CMeSH: Chinese translation of Medical Subject Headings
CDC: Center for Disease Control and prevention
MU: Medical University
EN: English
CN: Chinese
SC: Simplified Chinese
TC: Traditional Chinese
NGO: Non-Governmental Organization
LGBTQ: Lesbian, Gay, Bisexual, Transgender or Queer people
GDP: Gross Domestic Product
CNY: Chinese Yuan.
