# Supplementary material for: The Holistic Health Status of Chinese Homosexual and Bisexual Adults: A Scoping Review
Source: Front Public Health. 2021 Aug 24;9:710575. doi: 10.3389/fpubh.2021.710575 (PMC8421524; doi:10.3389/fpubh.2021.710575)
Supplement: Supplementary file 2 [file Data_Sheet_2.pdf]

Search strategy and Keywords/Subject Headings/Terms under PCC framework

**Concept**	**Keywords/MeSH terms* in English**	**Keywords/CMeSH terms# in Chinese**
Population:<br><br>Homosexual and Bisexual	Homosexuality*<br>*(Sub-terms including: Homosexuality, Female*; Homosexuality, Male*)*	同性恋#<br>*-同性恋，女性#*<br>*-同性恋，男性#*
	Bisexuality*	双性恋#
	Sexual and Gender Minorities*	性少数群体#
	Supplementary keywords search:<br>gay OR lesbian OR homosexual- OR non-heterosexual OR homoerotic OR homophile- OR queer OR same-sex attraction OR sexual inversion OR bisexual OR bi-sexual OR bi-gender OR bigender OR same gender love OR same gender attraction OR LGB OR LGB- OR GLB OR GBL- OR men who have sex with men OR male who have sex with male OR MSM OR male-male sex OR women who have sex with women OR WSW OR men who have sex with both men and women OR MSMW<br>同志 OR 男同 OR 女同 OR 男性同性恋/男同性恋 OR女性同性恋/女同性恋 OR 非异性恋 OR 双性恋 OR 同性爱 OR LGB(TQ)+人群 OR GLB(TQ)人群 OR男男性行为(者/人群) OR MSM OR女女性行为(者/人群) OR WSW OR MSMW	
Context:<br><br>China/Chinese	China*<br>*(Sub-terms including Hong Kong*; Macau*)*<br>Taiwan*	中国#<br>*-香港#*<br>*-澳门#*<br>台湾#
	Supplementary keywords search:<br>• Chinese OR Taiwanese<br>• No limitation on context in searching Chinese databases	
Concept:<br><br>Health<br>-Overview	Health*<br>*(Sub-terms including: Holistic Health*; Mental Health*; Sexual Health*; Reproductive Health*; Physical Fitness*)*	健康状况#<br>健康行为#<br>生殖健康#
	Health Services*<br>*(Sub-terms including: Mental Health Services*; Personal Health Services*; Reproductive Health Services*; Women's Health Services*)*	卫生服务#<br>*(次主题词：精神卫生服务#；个人保健服务#；预防卫生服务#；生殖健康服务#)*
Mental health	Emotions*	情绪#

-Supplementary	*(Sub-terms including: Anxiety*; Anger*; Fear*; Happiness*; Hate*; Hope*; Loneliness*; Love*; Pleasure*; Psychological Distress*; Sadness*)*	*(次主题词: 情感#; 焦虑#; 恐惧#; 幸福#; 憎恨#; 希望#; 孤独#; 爱恋#; 愉快#; 心理困扰; 悲痛#)*
	Mental Disorders*<br>*(Sub-terms including: Anxiety Disorders*; Mood Disorders*; Depressive Disorder*; Personality Disorders*; Sleep Wake Disorders*; Substance-Related Disorders*-Sexual Dysfunctions, Psychological*)*	精神障碍#<br>*(次主题词: 焦虑症#; 性欲倒错障碍#; 人格障碍#; 情绪障碍#; 抑郁症#; 睡眠觉醒障碍#; 物质相关性障碍#; 性功能障碍，心理性#)*
	Depression*<br>Stress, Psychological*<br>Social Stigma*<br>Social Discrimination*<br>Prejudice*<br>*(including: Homophobia*)*<br>Resilience, Psychological*<br>Self Concept*<br>Social Identification*<br>Identification, Psychological*<br>*(including Gender Identity*)*<br>Psychosexual Development*	抑郁#<br>应激，心理学#<br>社会污点#<br>社会歧视#<br>偏见# -*同性恋恐惧症#*<br>韧性，心理#<br>自我概念#<br>社会认同#<br>认同，心理学#<br>-*性别特性#*<br>性心理发育#
	Self-Injurious Behavior*<br>*(Sub-terms including: Suicide*; -Suicidal Ideation* and -Suicide, Attempted*)*	自我伤害行为#<br>*(次主题词: 自杀#; -自杀意念#; -自杀未遂#)*
Sexual health<br>-Supplementary	Sexually Transmitted Diseases*<br>*(Sub-terms including: HIV Infections*; Chlamydia Infections*; Chancroid*; Gonorrhea*; Granuloma Inguinale*; Syphilis*; Condylomata Acuminata*; Herpes Genitalis*; Acquired Immunodeficiency Syndrome*)*<br>HIV*<br>Sarcoma, Kaposi*<br>Reproductive Tract Infections*<br>Female Urogenital Diseases*<br>Male Urogenital Diseases*	性传播疾病#<br>*(次主题词: HIV感染#; 软下疳#; 衣原体感染#; 淋病#; 腹股沟肉芽肿#; 梅毒#; 生殖器疱疹#; 获得性免疫缺陷综合征#)*<br>HIV#<br>肉瘤，卡波西#<br>生殖道感染#<br>女性泌尿生殖病#<br>男性泌尿生殖病#
	Sexual Behavior*	性行为#

	(Sub-terms including: Safe Sex; Unsafe Sex*; Sex Work (Prostitution)*; Sexual Abstinence*; Sexual Harassment*)*<br>Sexual Partners*<br>Condoms*<br>Condoms, Female*<br>Libido*<br>Sexual Dysfunction, Physiological*<br>Orgasm*	*(次主题词: 安全性行为#; 危险性行为#; 性工作#; 性欲节制#; 性骚扰#)*<br>性伴侣#<br>避孕套#<br>避孕套，女用#<br>性力#<br>性功能障碍，生理性#<br>性欲高潮#
	Violence*<br>*(Sub-terms including: Domestic Violence*; Gender; Based Violence*; Intimate Partner Violence*; Physical Abuse*; Workplace Violence*)*<br>Sex Offenses*<br>*(Sub-term including: Rape*)*	暴力#<br>*(次主题词: 亲密伴侣暴力#; 身体虐待#; 暴力遭遇#; 家庭暴力#; 工作场所暴力#)*<br>性犯罪#<br>*-强奸#*
Social well-being -Supplementary	Quality of Life*<br>Social Support*<br>Psychology, Social*<br>Social Networking (+Online)*<br>Marital Status*<br>*(including: Marriage*)*<br>Family Relations*	生活质量#<br>社会支持#<br>心理学，社会#<br>社交网络#<br>婚姻状况#<br>*-婚姻#*<br>家庭关系#
	Supplementary keywords search of ‘Health’:<br>well being OR self esteem OR helpless OR risk(y) behaviors OR risk taking behavior OR drug use OR alcohol drinking OR Human Immunodeficiency Virus OR sexual compulsory OR sexual addiction OR gender identity violence<br>健康 OR 性健康 OR 卡波西肉瘤 OR 艾滋病 OR 性暴力 OR 性虐待OR 风险行为 OR 危险行为 OR 吸毒 OR 酗酒 OR 强迫性性行为 OR 性瘾OR 心理健康 OR OR 肛交 OR 口交 OR 药物滥用 OR 心理弹性OR性别认同 OR 自尊 OR 无助OR 性别认同暴力	

Note: * refers to MeSH terms; # refers to Chinese MeSH terms (CMeSH).
