# Supplementary material for: The Holistic Health Status of Chinese Homosexual and Bisexual Adults: A Scoping Review
Source: Front Public Health. 2021 Aug 24;9:710575. doi: 10.3389/fpubh.2021.710575 (PMC8421524; doi:10.3389/fpubh.2021.710575)
Supplement: Supplementary file 3 [file Data_Sheet_3.pdf]

Summary of databases searching results

Searching Date: May 7-31, 2020

Search Date	Databases	Results
	In English	
May 7, 2020	PubMed	1,213
May 12, 2020	Web of Science (update)	2,106
May 8, 2020	CINAHL Plus	421
May 8, 2020	ScienceDirect	140
May 8, 2020	Social Work Abstracts	14
May 8, 2020	ProQuest searching of the 5 databases:<br>1. ProQuest Dissertations & Theses A&I<br>2. ProQuest Dissertations & Theses Global<br>3. APA PsycInfo<br>4. Sociological Abstracts<br>5. Social Services Abstracts	853
May 8, 2020	Scopus	622
May 8, 2020	Cochrane library	132
May 12, 2020	Joanna Briggs Institute EBP Database	17
	Total search	5,518
	Duplicate papers	2,577
May 31, 2020	Updates via setting alerts	14
	Total left to screen	2,955
	In Simplified Chinese	
May 9, 2020	SinoMed (China Biological Medicine Database)	1,655
May 9, 2020	CNKI (China National Knowledge Infrastructure)	4,290
May 9, 2020	Wanfang Data	750
May 9, 2020	CQ VIP	643
	Total search	7,338
	Duplicate papers	1,642
	Total left to screen	5,696
	In Traditional Chinese	
May 9, 2020	Taiwan citation index-humanities and social sciences	321
May 9, 2020	Index to Taiwan periodical literature system	41
May 9, 2020	Synergy of metadata resources in Taiwan (SMRT)	84
May 9, 2020	National Digital Library of Theses and Dissertation in Taiwan	1,495
	Total search	1,941
	Duplicate papers	11
	Total left to screen	1,933

Syntax of English literature search

PubMed

PubMed - May 7, 2020		
Search	Query	Results
#1	Homosexuality[Mesh Terms] OR Bisexuality[Mesh Terms] OR Sexual and Gender Minorities[Mesh Terms]	33,679
#2	(gay[Title/Abstract] OR lesbian[Title/Abstract] OR homosexual*[Title/Abstract] OR non-heterosexual[Title/Abstract] OR homoerotic[Title/Abstract] OR homophile*[Title/Abstract] OR queer[Title/Abstract] OR same-sex attraction same gender love[Title/Abstract] OR same gender attraction[Title/Abstract] OR LGB*[Title/Abstract] OR GBL*[Title/Abstract]) OR (bisexual[Title/Abstract] OR bi-sexual[Title/Abstract] OR bi-gender[Title/Abstract] OR bigender[Title/Abstract] OR men who have sex with men[Title/Abstract] OR male who have sex with male[Title/Abstract] OR MSM[Title/Abstract] OR male-male sex[Title/Abstract] OR women who have sex with women[Title/Abstract] OR WSW[Title/Abstract] OR men who have sex with both men and women[Title/Abstract] OR MSMW[Title/Abstract])	29,725
#3	#1 OR #2	45,643
#4	(China OR Taiwan[MeSH Terms]) OR (Chinese[Title/Abstract] OR Taiwanese[Title/Abstract])	1,793,046
#5	((((Health OR Health Services OR Holistic Health[MeSH Terms])) OR (Mental Health OR Emotions OR Mental Disorders OR Depression OR Stress, Psychological OR Social Stigma OR Social Discrimination OR Prejudice OR Homophobia OR Resilience, Psychological OR Self Concept OR Social Identification OR Identification, Psychological OR Gender Identity OR Psychosexual Development[MeSH Terms])) OR (Physical Fitness OR Sexual Health OR Reproductive Health OR Sexually Transmitted Diseases OR HIV OR Sarcoma, Kaposi OR Reproductive Tract Infections OR Female Urogenital Diseases OR Male Urogenital Diseases OR Sexual Behavior OR Sexual Partners OR Condoms OR Condoms, Female OR Libido OR Sexual Dysfunction, Physiological OR Orgasm OR Violence OR Sex Offenses[MeSH Terms])) OR (Quality of Life OR Social Support OR Psychology, Social OR Social Networking OR Marital Status OR Family Relations[MeSH Terms])	8,899,265
#6	(well being[Title/Abstract] OR self esteem[Title/Abstract] OR helpless[Title/Abstract] OR sexually transmitted infection ORrisk* behavior*[Title/Abstract] OR risk taking behavior[Title/Abstract]	151,182

	OR drug use[Title/Abstract] OR alcohol drinking[Title/Abstract] OR Human Immunodeficiency Virus[Title/Abstract] OR sexual compulsory[Title/Abstract] OR sexual addiction[Title/Abstract] OR gender identity violence[Title/Abstract])	
#7	#5 OR #6	8,912,752
#8	#3 AND #4 AND #7	1,724
#9	Limited to publication between 2001-2020, Human studies and English language	1,213

Query	Items found
Search **(((((((well being[Title/Abstract] OR self esteem[Title/Abstract] OR helpless[Title/Abstract] OR sexually transmitted infection ORrisk* behavior*[Title/Abstract] OR risk taking behavior[Title/Abstract] OR drug use[Title/Abstract] OR alcohol drinking[Title/Abstract] OR Human Immunodeficiency Virus[Title/Abstract] OR sexual compulsory[Title/Abstract] OR sexual addiction[Title/Abstract] OR gender identity violence[Title/Abstract])))) OR ((((((Health OR Health Services OR Holistic Health[MeSH Terms])) OR (Mental Health OR Emotions OR Mental Disorders OR Depression OR Stress, Psychological OR Social Stigma OR Social Discrimination OR Prejudice OR Homophobia OR Resilience, Psychological OR Self Concept OR Social Identification OR Identification, Psychological OR Gender Identity OR Psychosexual Development[MeSH Terms])) OR (Physical Fitness OR Sexual Health OR Reproductive Health OR Sexually Transmitted Diseases OR HIV OR Sarcoma, Kaposi OR Reproductive Tract Infections OR Female Urogenital Diseases OR Male Urogenital Diseases OR Sexual Behavior OR Sexual Partners OR Condoms OR Condoms, Female OR Libido OR Sexual Dysfunction, Physiological OR Orgasm OR Violence OR Sex Offenses[MeSH Terms])) OR (Quality of Life OR Social Support OR Psychology, Social OR Social Networking OR Marital Status OR Family Relations[MeSH Terms]))))) AND (((China OR Taiwan[MeSH Terms]) OR (Chinese[Title/Abstract] OR Taiwanese[Title/Abstract])))) AND (((((gay[Title/Abstract] OR lesbian[Title/Abstract] OR homosexual*[Title/Abstract] OR non-heterosexual[Title/Abstract] OR homoerotic[Title/Abstract] OR homophile*[Title/Abstract] OR queer[Title/Abstract] OR same-sex attraction same gender love[Title/Abstract] OR same gender attraction[Title/Abstract] OR LGB*[Title/Abstract] OR GBL* [Title/Abstract]) OR (bisexual[Title/Abstract] OR bi-sexual[Title/Abstract] OR bi-gender[Title/Abstract] OR bigender[Title/Abstract] OR men who have sex with men[Title/Abstract] OR male who have sex with male[Title/Abstract] OR MSM[Title/Abstract] OR male-male sex[Title/Abstract] OR women who have sex with women[Title/Abstract] OR WSW[Title/Abstract] OR men who have sex with both men[Title/Abstract] AND women[Title/Abstract] OR MSMW[Title/Abstract])))) OR ((Homosexuality[Mesh Terms] OR Bisexuality[Mesh Terms] OR Sexual and Gender Minorities[Mesh Terms])))** Sort by: **Author** Filters: **Publication date from 2001/01/01 to 2020/12/31; Humans; English**	1213

Web of Science

Web of Science - May 12, 2020		
Search	Query	Results
#1	TOPIC: (homosexuality OR gay OR lesbian OR homosexual OR homoerotic OR homophile OR bisexuality OR bisexual OR bigender OR MSM OR WSW OR MSMW OR LGB* OR GBL* OR queer) OR TOPIC: (sexual minorit*) OR TOPIC: (men who have sex with men) OR TOPIC: (women who have sex with women) OR TOPIC: (men who have sex with both men and women)	105,088
#2	TOPIC: (China OR Chinese) OR TOPIC: (Taiwan OR Taiwanese)	1,006,824
#3	TOPIC: (Health OR Health Service OR Holistic Health) OR TOPIC: (Mental Health OR Emotion OR Mental Disorder OR Depression OR Stress OR Stigma OR Discrimination OR	6,884,165

	Prejudice OR Homophobia OR Resilience OR Self Concept OR Identification OR Gender Identity OR Psychosexual Development) OR TOPIC: (Physical Health OR Sexual Health OR Reproductive Health OR Sexually Transmitted Diseases OR sexually transmitted infection OR HIV OR Human Immunodeficiency Virus OR Acquired Immunodeficiency Syndrome OR Chlamydia OR Gonorrhea OR Granuloma Inguinale OR Syphilis OR Condylomata Acuminata OR Herpes Genitalis OR Kaposi OR Reproductive Tract Infections OR Female Urogenital Disease OR Male Urogenital Disease OR Sexual Behavior OR Sexual Partner OR Condom OR Libido OR Sexual Dysfunction OR Orgasm OR Violence OR Abuse OR Suicide OR Sex Offense OR sexual compulsory OR sexual addiction OR gender identity violence OR Unsafe Sex OR Sexual Abstinence OR Sexual Harassment	
#4	#1 AND #2 AND #3	2,188
#5	Limited to publication between 2001-2020 and English language	2,106

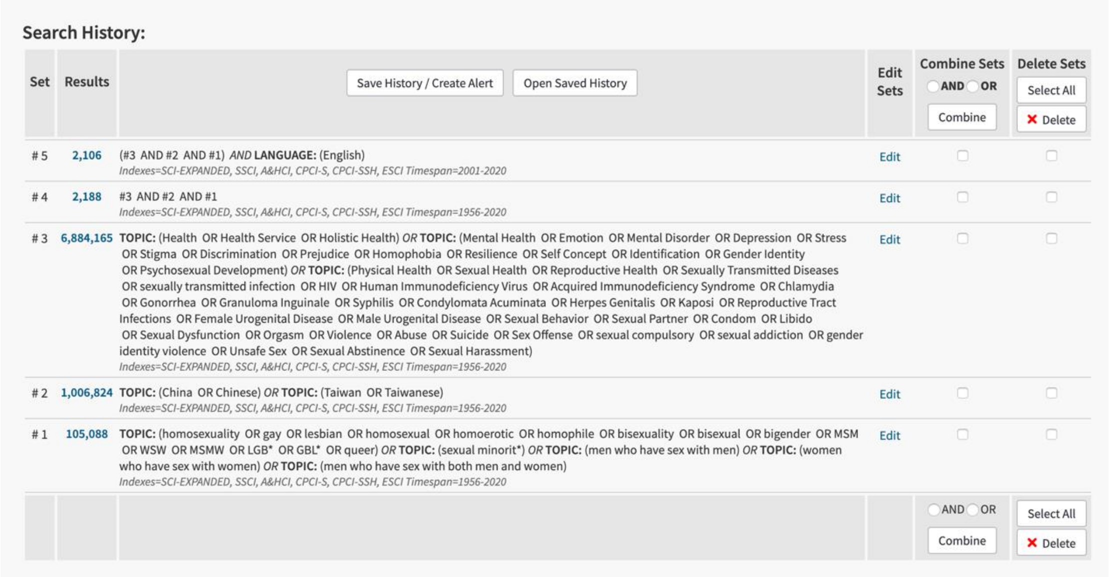

Search History:

Set	Results	Save History / Create Alert · Open Saved History	Edit Sets	Combine Sets (AND / OR, Combine)	Delete Sets (Select All, Delete)
# 5	2,106	(#3 AND #2 AND #1) *AND* **LANGUAGE:** (English)<br>*Indexes=SCI-EXPANDED, SSCI, A&HCI, CPCI-S, CPCI-SSH, ESCI Timespan=2001-2020*	Edit		
# 4	2,188	#3 AND #2 AND #1<br>*Indexes=SCI-EXPANDED, SSCI, A&HCI, CPCI-S, CPCI-SSH, ESCI Timespan=1956-2020*	Edit		
# 3	6,884,165	**TOPIC:** (Health OR Health Service OR Holistic Health) *OR* **TOPIC:** (Mental Health OR Emotion OR Mental Disorder OR Depression OR Stress OR Stigma OR Discrimination OR Prejudice OR Homophobia OR Resilience OR Self Concept OR Identification OR Gender Identity OR Psychosexual Development) *OR* **TOPIC:** (Physical Health OR Sexual Health OR Reproductive Health OR Sexually Transmitted Diseases OR sexually transmitted infection OR HIV OR Human Immunodeficiency Virus OR Acquired Immunodeficiency Syndrome OR Chlamydia OR Gonorrhea OR Granuloma Inguinale OR Syphilis OR Condylomata Acuminata OR Herpes Genitalis OR Kaposi OR Reproductive Tract Infections OR Female Urogenital Disease OR Male Urogenital Disease OR Sexual Behavior OR Sexual Partner OR Condom OR Libido OR Sexual Dysfunction OR Orgasm OR Violence OR Abuse OR Suicide OR Sex Offense OR sexual compulsory OR sexual addiction OR gender identity violence OR Unsafe Sex OR Sexual Abstinence OR Sexual Harassment)<br>*Indexes=SCI-EXPANDED, SSCI, A&HCI, CPCI-S, CPCI-SSH, ESCI Timespan=1956-2020*	Edit		
# 2	1,006,824	**TOPIC:** (China OR Chinese) *OR* **TOPIC:** (Taiwan OR Taiwanese)<br>*Indexes=SCI-EXPANDED, SSCI, A&HCI, CPCI-S, CPCI-SSH, ESCI Timespan=1956-2020*	Edit		
# 1	105,088	**TOPIC:** (homosexuality OR gay OR lesbian OR homosexual OR homoerotic OR homophile OR bisexuality OR bisexual OR bigender OR MSM OR WSW OR MSMW OR LGB* OR GBL* OR queer) *OR* **TOPIC:** (sexual minorit*) *OR* **TOPIC:** (men who have sex with men) *OR* **TOPIC:** (women who have sex with women) *OR* **TOPIC:** (men who have sex with both men and women)<br>*Indexes=SCI-EXPANDED, SSCI, A&HCI, CPCI-S, CPCI-SSH, ESCI Timespan=1956-2020*	Edit		
				AND / OR, Combine	Select All, Delete

CINAHL Plus (via EBSCOhost)

CINAHL Plus (via EBSCOhost) – May 8, 2020		
Search	Query	Results
#1	SU (homosexuality OR gay OR lesbian OR homosexual OR homoerotic OR homophile OR bisexuality OR bisexual OR bigender OR LGB* OR GBL* OR queer) OR SU (homosexuality OR gay OR lesbian OR homosexual OR homoerotic OR homophile OR bisexuality OR bisexual OR bigender OR LGB* OR GBL* OR queer) OR SU (men who have sex with men OR MSM) OR SU (women who have sex with women OR WSW) OR SU (men who have sex with both men and women OR MSMW)	19,018
#2	SU China OR Taiwan OR Chinese OR Taiwanese	89,166

#3	SU (Health OR Health Services OR Holistic Health) OR SU (Mental Health OR Emotion OR Mental Disorder OR Depression OR Stress OR Stigma OR Discrimination OR Prejudice OR Homophobia OR Resilience OR Self Concept OR Identification OR Gender Identity OR Psychosexual Development) OR SU (Physical Health OR Sexual Health OR Reproductive Health OR Sexually Transmitted Diseases OR sexually transmitted infection OR HIV OR Human Immunodeficiency Virus OR Acquired Immunodeficiency Syndrome OR Chlamydia OR Gonorrhea OR Granuloma Inguinale OR Syphilis OR Condylomata Acuminata OR Herpes Genitalis OR Kaposi OR Reproductive Tract Infections OR Female Urogenital Disease OR Male Urogenital Disease OR Sexual Behavior OR Sexual Partner OR Condom OR Libido OR Sexual Dysfunction OR Orgasm OR Violence OR Abuse OR Suicide OR Sex Offense OR sexual compulsory OR sexual addiction OR gender identity violence OR Unsafe Sex OR Sexual Abstinence OR Sexual Harassment) OR SU (Quality of Life OR Social Support OR Psychology, Social OR Social Networking OR Marital Status OR Family Relations OR well being OR self esteem OR helpless OR risk behavior OR risk taking behavior OR drug use OR alcohol drinking)	1,952,901
#4	#1 AND #2 AND #3	523
#5	Limited to publication between 2001-2020, Human studies and English language	421

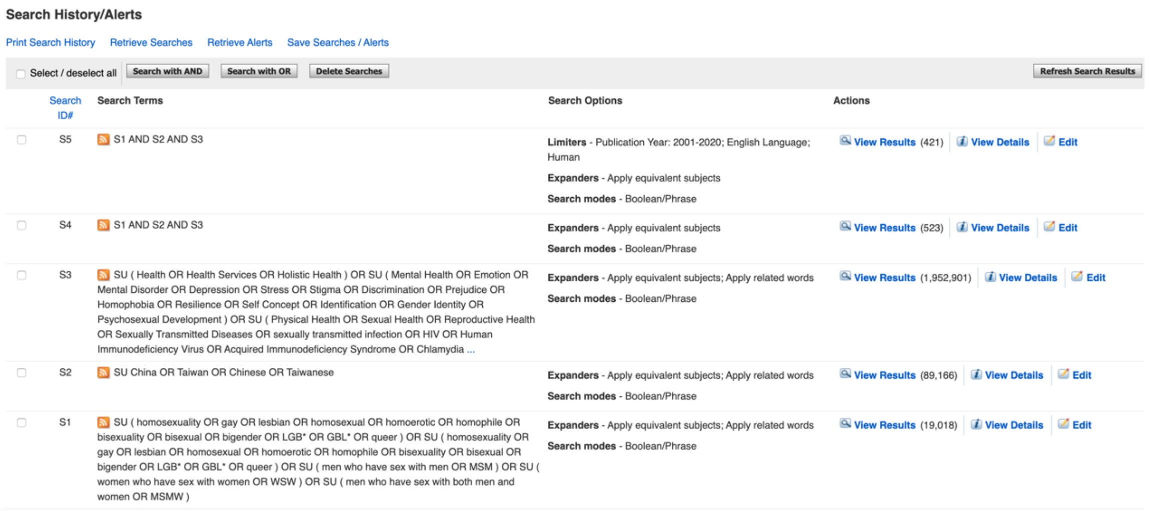

Search History/Alerts

Print Search History Retrieve Searches Retrieve Alerts Save Searches / Alerts

Select / deselect all | Search with AND | Search with OR | Delete Searches | Refresh Search Results

Search ID#	Search Terms	Search Options	Actions
S5	S1 AND S2 AND S3	Limiters - Publication Year: 2001-2020; English Language; Human<br>Expanders - Apply equivalent subjects<br>Search modes - Boolean/Phrase	View Results (421) View Details Edit
S4	S1 AND S2 AND S3	Expanders - Apply equivalent subjects<br>Search modes - Boolean/Phrase	View Results (523) View Details Edit
S3	SU (Health OR Health Services OR Holistic Health) OR SU (Mental Health OR Emotion OR Mental Disorder OR Depression OR Stress OR Stigma OR Discrimination OR Prejudice OR Homophobia OR Resilience OR Self Concept OR Identification OR Gender Identity OR Psychosexual Development) OR SU (Physical Health OR Sexual Health OR Reproductive Health OR Sexually Transmitted Diseases OR sexually transmitted infection OR HIV OR Human Immunodeficiency Virus OR Acquired Immunodeficiency Syndrome OR Chlamydia ...	Expanders - Apply equivalent subjects; Apply related words<br>Search modes - Boolean/Phrase	View Results (1,952,901) View Details Edit
S2	SU China OR Taiwan OR Chinese OR Taiwanese	Expanders - Apply equivalent subjects; Apply related words<br>Search modes - Boolean/Phrase	View Results (89,166) View Details Edit
S1	SU (homosexuality OR gay OR lesbian OR homosexual OR homoerotic OR homophile OR bisexuality OR bisexual OR bigender OR LGB* OR GBL* OR queer) OR SU (homosexuality OR gay OR lesbian OR homosexual OR homoerotic OR homophile OR bisexuality OR bisexual OR bigender OR LGB* OR GBL* OR queer) OR SU (men who have sex with men OR MSM) OR SU (women who have sex with women OR WSW) OR SU (men who have sex with both men and women OR MSMW)	Expanders - Apply equivalent subjects; Apply related words<br>Search modes - Boolean/Phrase	View Results (19,018) View Details Edit

ScienceDirect

ScienceDirect – May 8, 2020		
Search	Query	Results
#1	(homosexual OR gay OR lesbian OR bisexual OR men who have sex with men OR women who have sex with women) AND health AND (China OR Taiwan) AND Limited to publication between 2001-2020	140

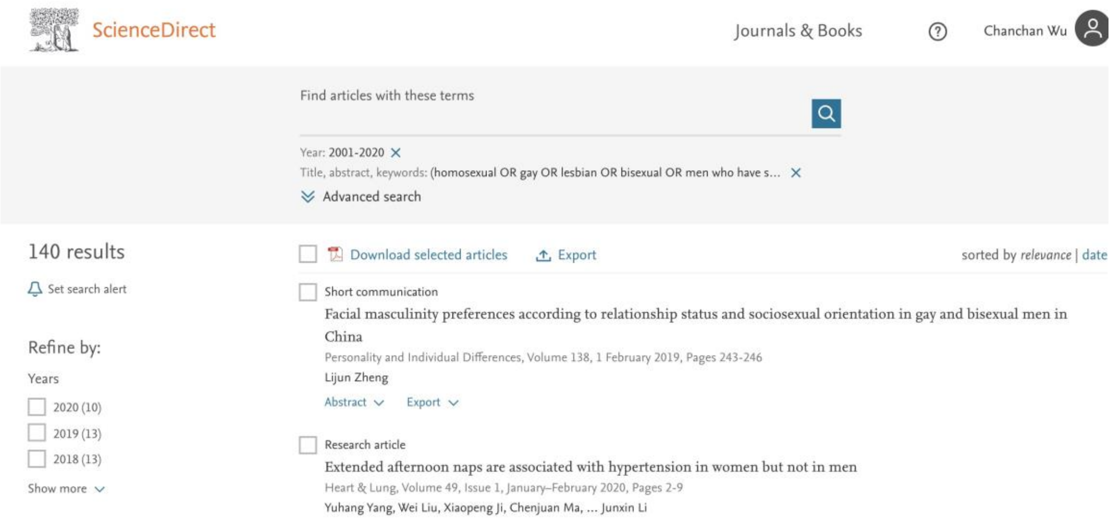

Social Work Abstracts (via Ovid)

Social Work Abstracts – May 8, 2020		
Search	Query	Results
#1	(homosexuality or gay or lesbian or homosexual or homoerotic or homophile or bisexuality or bisexual or bigender or LGB* or GBL* or queer or sexual minorit* or men who have sex with men or MSM or women who have sex with women or WSW or (men who have sex with both men and women) or MSMW).mp. [mp=title, abstract, subject heading, heading word]	1,799
#2	(China or Hong Kong or Macau or Taiwan or Chinese or Taiwanese).mp. [mp=title, abstract, subject heading, heading word]	1,039
#3	(Health or Health Services or Holistic Health or Mental Health or Emotion or Mental Disorder or Depression or Stress or Stigma or Discrimination or Prejudice or Homophobia or Resilience or Self Concept or Identification or Gender Identity or Psychosexual Development).mp. [mp=title, abstract, subject heading, heading word]	21,706
#4	(Physical Health or Sexual Health or Reproductive Health or Sexually Transmitted Diseases or sexually transmitted infection or HIV or Human Immunodeficiency Virus or Acquired Immunodeficiency Syndrome or Chlamydia or Gonorrhea or Granuloma Inguinale or Syphilis or Condylomata Acuminata or Herpes Genitalis or Kaposi or Reproductive Tract Infections or Female Urogenital Disease or Male Urogenital Disease or Sexual Behavior or Sexual Partner or Condom or Libido or Sexual Dysfunction or Orgasm or Violence or Abuse or Suicide or Sex Offense or sexual compulsory or sexual addiction or gender identity violence or Unsafe Sex or Sexual Abstinence or Sexual Harassment).mp. [mp=title, abstract, subject heading, heading word]	10,930

#5	(Quality of Life or Social Support or Social Psychology or Social Networking or Marital Status or Family Relations or well being or self esteem or helpless or risk behavior or risk taking behavior or drug or alcohol).mp. [mp=title, abstract, subject heading, heading word]	10,419
#6	#3 OR #4 OR #5	32,789
#7	#1 AND #2 AND #6	15
#8	Limited to publication between 2001-2020	14

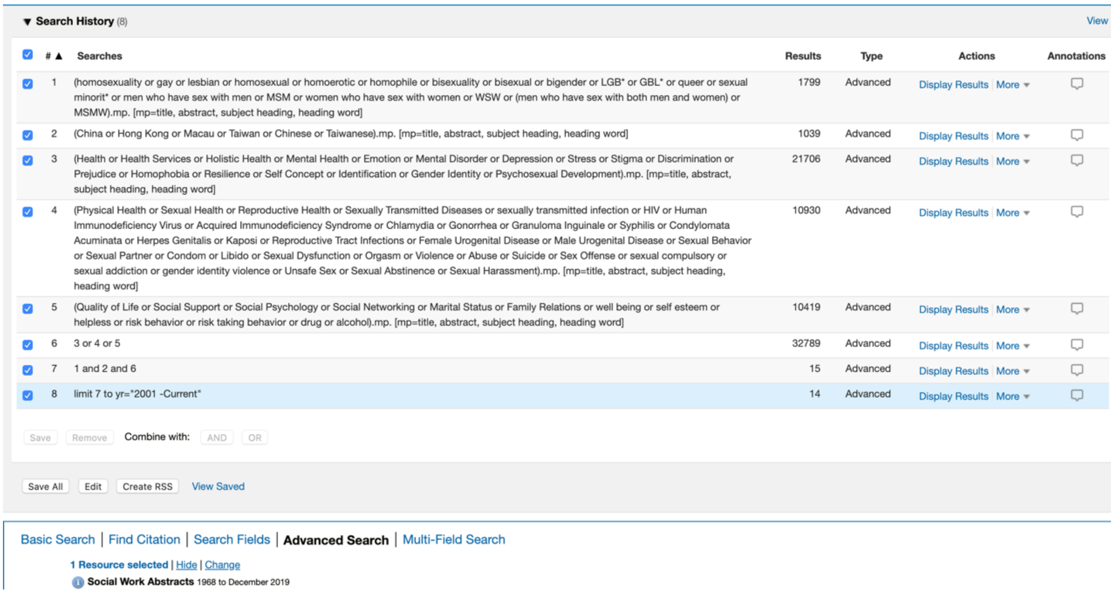

▼ Search History (8) View

# ▲	Searches	Results	Type	Actions	Annotations
1	(homosexuality or gay or lesbian or homosexual or homoerotic or homophile or bisexuality or bisexual or bigender or LGB* or GBL* or queer or sexual minorit* or men who have sex with men or MSM or women who have sex with women or WSW or (men who have sex with both men and women) or MSMW).mp. [mp=title, abstract, subject heading, heading word]	1799	Advanced	Display Results | More	
2	(China or Hong Kong or Macau or Taiwan or Chinese or Taiwanese).mp. [mp=title, abstract, subject heading, heading word]	1039	Advanced	Display Results | More	
3	(Health or Health Services or Holistic Health or Mental Health or Emotion or Mental Disorder or Depression or Stress or Stigma or Discrimination or Prejudice or Homophobia or Resilience or Self Concept or Identification or Gender Identity or Psychosexual Development).mp. [mp=title, abstract, subject heading, heading word]	21706	Advanced	Display Results | More	
4	(Physical Health or Sexual Health or Reproductive Health or Sexually Transmitted Diseases or sexually transmitted infection or HIV or Human Immunodeficiency Virus or Acquired Immunodeficiency Syndrome or Chlamydia or Gonorrhea or Granuloma Inguinale or Syphilis or Condylomata Acuminata or Herpes Genitalis or Kaposi or Reproductive Tract Infections or Female Urogenital Disease or Male Urogenital Disease or Sexual Behavior or Sexual Partner or Condom or Libido or Sexual Dysfunction or Orgasm or Violence or Abuse or Suicide or Sex Offense or sexual compulsory or sexual addiction or gender identity violence or Unsafe Sex or Sexual Abstinence or Sexual Harassment).mp. [mp=title, abstract, subject heading, heading word]	10930	Advanced	Display Results | More	
5	(Quality of Life or Social Support or Social Psychology or Social Networking or Marital Status or Family Relations or well being or self esteem or helpless or risk behavior or risk taking behavior or drug or alcohol).mp. [mp=title, abstract, subject heading, heading word]	10419	Advanced	Display Results | More	
6	3 or 4 or 5	32789	Advanced	Display Results | More	
7	1 and 2 and 6	15	Advanced	Display Results | More	
8	limit 7 to yr="2001 -Current"	14	Advanced	Display Results | More	

Save Remove Combine with: AND OR

Save All Edit Create RSS View Saved

Basic Search | Find Citation | Search Fields | Advanced Search | Multi-Field Search

1 Resource selected | Hide | Change

Social Work Abstracts 1968 to December 2019

APA PsycInfo, Dissertations & Theses, Sociological Abstracts via ProQuest

ProQuest searching of the 5 databases below (May 8, 2020):<br>1. ProQuest Dissertations & Theses A&I<br>2. ProQuest Dissertations & Theses Global<br>3. APA PsycInfo<br>4. Sociological Abstracts<br>5. Social Services Abstracts		
Search	Query	Results
#1	su(homosexuality OR gay OR lesbian OR homosexual OR homoerotic OR homophile OR bisexuality OR bisexual OR bigender OR LGB* OR GBL* OR queer) OR su(sexual minorit* OR men who have sex with men OR MSM OR women who have sex with women OR WSW OR (men who have sex with both men and women) OR MSMW)	77,891
#2	su(China OR Hong Kong Or Macau OR Taiwan OR Chinese OR Taiwanese)	145,732
#3	su(Health OR Health Services OR Holistic Health OR Mental Health OR Emotion OR Mental Disorder OR Depression OR	3,171,127

	Stress OR Stigma OR Discrimination OR Prejudice OR Homophobia OR Resilience OR Self Concept OR Identification OR Gender Identity OR Psychosexual Development) OR su(Physical Health OR Sexual Health OR Reproductive Health OR Sexually Transmitted Diseases OR sexually transmitted infection OR HIV OR Human Immunodeficiency Virus OR Acquired Immunodeficiency Syndrome OR Chlamydia OR Gonorrhea OR Granuloma Inguinale OR Syphilis OR Condylomata Acuminata OR Herpes Genitalis OR Kaposi OR Reproductive Tract Infections OR Female Urogenital Disease OR Male Urogenital Disease OR Sexual Behavior OR Sexual Partner OR Condom OR Libido OR Sexual Dysfunction OR Orgasm OR Violence OR Abuse OR Suicide OR Sex Offense OR sexual compulsory OR sexual addiction OR gender identity violence OR Unsafe Sex OR Sexual Abstinence OR Sexual Harassment) OR su(Quality of Life OR Social Support OR Social Psychology OR Social Networking OR Marital Status OR Family Relations OR well being OR self esteem OR helpless OR risk behavior OR risk taking behavior OR drug use OR alcohol drinking)	
#4	#1 AND #2 AND #3	933
#5	Limited to publication between 2001-2020 and English language	853

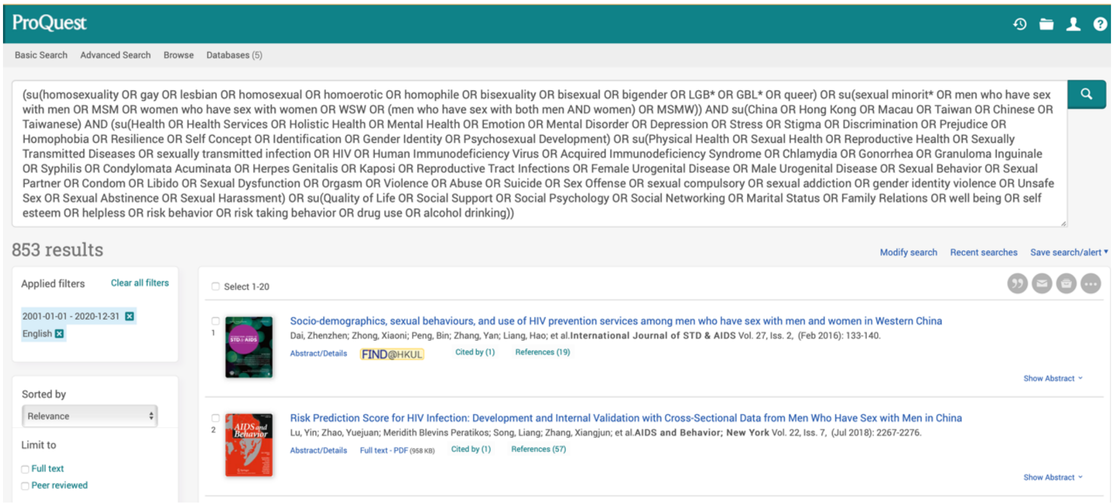

Scopus

Scopus – May 8, 2020		
Search	Query	Results
#1	TITLE-ABS-KEY (homosexuality OR gay OR lesbian OR homosexual OR homoerotic OR homophile OR bisexuality OR bisexual OR bigender OR lgb* OR gbl* OR queer OR sexual AND minorit* OR (men AND who AND have AND sex AND with AND men) OR msm OR (women AND who AND have	39,280

	AND sex AND with AND women) OR wsw OR (men AND who AND have AND sex AND with AND both AND men AND women) OR msmw)	
#2	TITLE-ABS-KEY (china OR hong AND kong OR macau OR taiwan OR chinese OR taiwanese)	283,740
#3	TITLE-ABS-KEY (health OR (health AND services) OR (holistic AND health) OR (mental AND health) OR emotion OR (mental AND disorder) OR depression OR stress OR stigma OR discrimination OR prejudice OR homophobia OR resilience OR (self AND concept) OR identification OR (gender AND identity) OR (psychosexual AND development) OR (physical AND health) OR (sexual AND health) OR (reproductive AND health) OR (sexually AND transmitted AND disease) OR (sexually AND transmitted AND infection) OR hiv OR (human AND immunodeficiency AND virus) OR (acquired AND immunodeficiency AND syndrome) OR chlamydia OR gonorrhea OR (granuloma AND inguinale) OR syphilis OR (condylomata AND acuminata) OR (herpes AND genitalis) OR kaposi OR (reproductive AND tract AND infection) OR (female AND urogenital AND disease) OR (male AND urogenital AND disease) OR (sexual AND behavior) OR (sexual AND partner) OR condom OR libido OR (sexual AND dysfunction) OR orgasm OR violence OR abuse OR suicide OR (sex AND offense) OR (sexual AND compulsory) OR (sexual AND addiction) OR (gender AND identity AND violence) OR (unsafe AND sex) OR (sexual AND abstinence) OR (sexual AND harassment) OR (quality AND of AND life) OR (social AND support) OR (social AND psychology) OR (social AND networking) OR (marital AND status) OR (family AND relations) OR (well AND being) OR (self AND esteem) OR helpless OR (risk AND behavior) OR (risk AND taking AND behavior) OR (drug AND use) OR (alcohol AND drinking))	23,635,628
#4	#1 AND #2 AND #3	711
#5	Limited to publication between 2001-2020 and English language	622

Search history | Combine queries...

11	(TITLE-ABS-KEY (homosexuality OR gay OR lesbian OR homosexual OR homoerotic OR homophile OR bisexuality OR bisexual OR bigender OR lgb* OR gbl* OR queer OR sexual AND minorit* OR (men AND who AND have AND sex AND with AND men) OR msm OR (women AND who AND have AND sex AND with AND women) OR wsw OR (men AND who AND have AND sex AND with AND both AND men AND women) OR msmw)) AND (TITLE-ABS-KEY (china OR hong AND kong OR macau OR taiwan OR chinese OR taiwanese)) AND (TITLE-ABS-KEY (health OR (health AND services) OR (holistic AND health) OR (mental AND health) OR emotion OR (mental AND disorder) OR depression OR stress OR stigma OR discrimination OR prejudice OR homophobia OR resilience OR (self AND concept) OR identification OR (gender AND identity) OR (psychosexual AND development) OR (physical AND health) OR (sexual AND health) OR (reproductive AND health) OR (sexually AND transmitted AND disease) OR (sexually AND transmitted AND infection) OR hiv OR (human AND immunodeficiency AND virus) OR (acquired AND immunodeficiency AND syndrome) OR chlamydia OR gonorrhea OR (granuloma AND inguinale) OR syphilis OR (condylomata AND acuminata) OR (herpes AND genitalis) OR kaposi OR (reproductive AND tract AND infection) OR (female AND urogenital AND disease) OR (male AND urogenital AND disease) OR (sexual AND behavior) OR (sexual AND partner) OR condom OR libido OR (sexual AND dysfunction) OR orgasm OR violence OR abuse OR suicide OR (sex AND offense) OR (sexual AND compulsory) OR (sexual AND addiction) OR (gender AND identity AND violence) OR (unsafe AND sex) OR (sexual AND abstinence) OR (sexual AND harassment) OR (quality AND of AND life) OR (social AND support) OR (social AND psychology) OR (social AND networking) OR (marital AND status) OR (family AND relations) OR (well AND being) OR (self AND esteem) OR helpless OR (risk AND behavior) OR (risk AND taking AND behavior) OR (drug AND use) OR (alcohol AND drinking))) AND (LIMIT-TO (PUBYEAR , 2020) OR LIMIT-TO (PUBYEAR , 2019) OR LIMIT-TO (PUBYEAR , 2018) OR LIMIT-TO (PUBYEAR , 2017) OR LIMIT-TO (PUBYEAR , 2016) OR LIMIT-TO (PUBYEAR , 2015) OR LIMIT-TO (PUBYEAR , 2014) OR LIMIT-TO (PUBYEAR , 2013) OR LIMIT-TO (PUBYEAR , 2012) OR LIMIT-TO (PUBYEAR , 2011) OR LIMIT-TO (PUBYEAR , 2010) OR LIMIT-TO (PUBYEAR , 2009) OR LIMIT-TO (PUBYEAR , 2008) OR LIMIT-TO (PUBYEAR , 2007) OR LIMIT-TO (PUBYEAR , 2006) OR LIMIT-TO (PUBYEAR , 2005) OR LIMIT-TO (PUBYEAR , 2004) OR LIMIT-TO (PUBYEAR , 2003) OR LIMIT-TO (PUBYEAR , 2002) OR LIMIT-TO (PUBYEAR , 2001)) AND (LIMIT-TO (LANGUAGE , "English"))	622 document results

View Less

Cochrane library

Cochrane library – May 8, 2020			
Search	Query	Results	
#1	(homosexuality OR gay OR lesbian OR homosexual OR homoerotic OR homophile OR bisexuality OR bisexual OR bigender OR LGB* OR GBL* OR queer OR sexual minorit* OR (men who have sex with men) OR MSM OR (women who have sex with women) OR WSW OR (men who have sex with both men and women) OR MSMW) in Title Abstract Keyword	Cochrane Reviews	123
		Trials	3,444
		Total	3,572
#2	#1 AND (Health OR (Health Services) OR (Holistic Health) OR (Mental Health OR Emotion) OR (Mental Disorder) OR Depression OR Stress OR Stigma OR Discrimination OR Prejudice OR Homophobia OR Resilience OR (Self Concept) OR Identification OR (Gender Identity) OR (Psychosexual Development) OR (Physical Health) OR (Sexual Health) OR (Reproductive Health) OR (Sexually Transmitted Disease) OR (sexually transmitted infection) OR HIV OR (Human Immunodeficiency Virus) OR (Acquired Immunodeficiency Syndrome) OR Chlamydia OR Gonorrhea OR (Granuloma Inguinale) OR Syphilis OR (Condylomata Acuminata) OR (Herpes Genitalis) OR Kaposi OR (Reproductive Tract Infection) OR (Female Urogenital Disease) OR (Male Urogenital Disease) OR (Sexual Behavior) OR (Sexual Partner) OR Condom OR Libido OR (Sexual Dysfunction) OR Orgasm OR Violence OR Abuse OR Suicide OR (Sex Offense) OR (sexual compulsory) OR (sexual addiction) OR (gender	Cochrane Reviews	120
		Trials	2,983
		Total	3,108

	identity violence) OR (Unsafe Sex) OR (Sexual Abstinence) OR (Sexual Harassment) OR (Quality of Life) OR (Social Support) OR (Social Psychology) OR (Social Networking) OR (Marital Status) OR (Family Relations) OR (well being) OR (self esteem) OR helpless OR (risk behavior) OR (risk taking behavior) OR (drug use) OR (alcohol drinking)) in Title Abstract Keyword		
#3	#1 AND #2 AND<br>(China OR Hong Kong Or Macau OR Taiwan OR Chinese OR Taiwanese) in Title Abstract Keyword	Cochrane Reviews	12
		Trials	124
		Total	136
#4	#3 with publication data between 2001-2020	Cochrane Reviews	12
		Trials	120
		Total	132

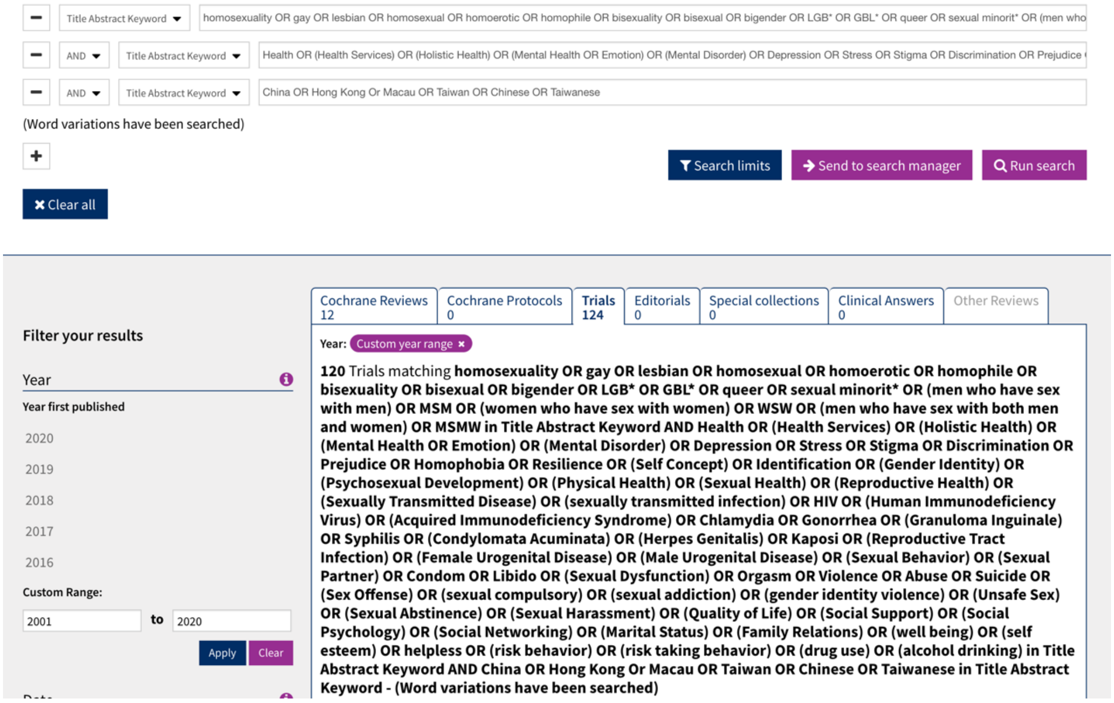

Joanna Briggs Institute EBP Database (via Ovid)

JBI EBP – May 12, 2020		
Search	Query	Results
#1	(homosexuality or gay or lesbian or homosexual or homoerotic or homophile or bisexuality or bisexual or bigender or MSM or WSW or MSMW or LGB* or GBL* or queer or sexual minorit* or men who have sex with men or women who have sex with women or (men who have sex with both men and women)).mp. [mp=text, heading word, subject area node, title]	67
#2	(China or Chinese or (Taiwan or Taiwanese)).mp. [mp=text, heading	674

	word, subject area node, title]	
#3	(Health or Health Service or Holistic Health or (Mental Health or Emotion or Mental Disorder or Depression or Stress or Stigma or Discrimination or Prejudice or Homophobia or Resilience or Self Concept or Identification or Gender Identity or Psychosexual Development) or (Physical Health or Sexual Health or Reproductive Health or Sexually Transmitted Diseases or sexually transmitted infection or HIV or Human Immunodeficiency Virus or Acquired Immunodeficiency Syndrome or Chlamydia or Gonorrhea or Granuloma Inguinale or Syphilis or Condylomata Acuminata or Herpes Genitalis or Kaposi or Reproductive Tract Infections or Female Urogenital Disease or Male Urogenital Disease or Sexual Behavior or Sexual Partner or Condom or Libido or Sexual Dysfunction or Orgasm or Violence or Abuse or Suicide or Sex Offense or sexual compulsory or sexual addiction or gender identity violence or Unsafe Sex or Sexual Abstinence or Sexual Harassment)).mp. [mp=text, heading word, subject area node, title]	6,537
#4	#1 and #2 and #3	17
#5	Limit #4 to yr="2001 - 2020"	17

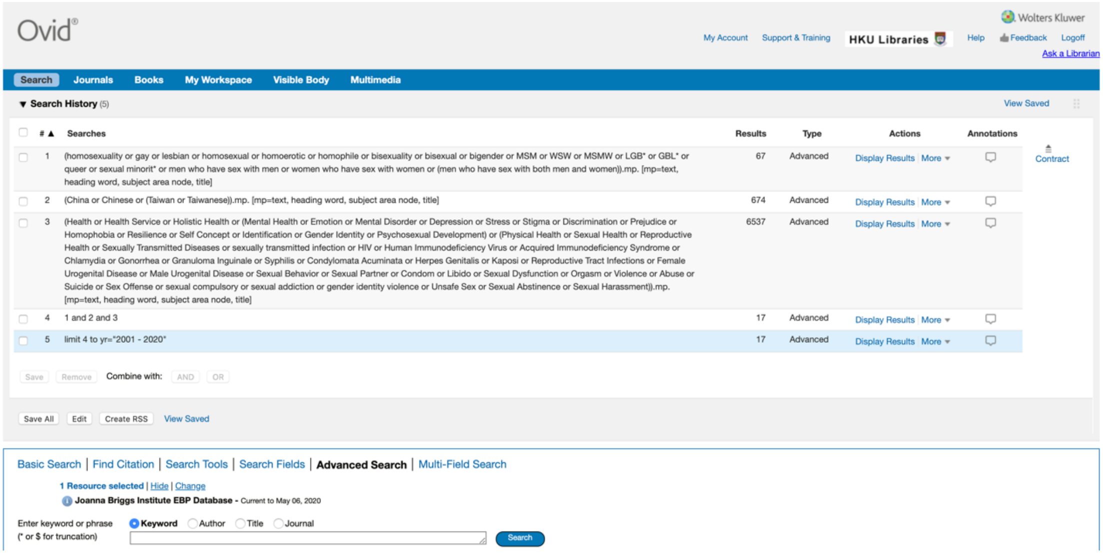

Update records

Web of Science

Web of Science Clarivate Analytics

Greetings! You have a saved search alert. View records

Your search, (#3 AND #2 AND #1) *AND* **LANGUAGE:** (English) has 2 new records since May 12th 2020.

Showing 2 of the 2

Filial Piety, Internalized Homonegativity, and Depressive Symptoms Among Taiwanese Gay and Bisexual Men: A Mediation Analysis

Huang, Yu-Te; Chan, Randolph Chun Ho; Cui, Lixian
American Journal Of Orthopsychiatry

A theme emerging from the current literature is that Chinese gay and bisexual men are likely to struggle to accept themselves because of the cultural emphasis on filial piety. However, our understanding of this culturally particular proc...

A growing trend of females and dermatologists among top medical graduates in 30 years

Chang, Yung-Wei; Lee, Chih-Hung
Bmc Medical Education

Background Career outcomes of top medical graduates (TMG) are seldom studied. The Tsungming Tu Foundation (TTF) has awarded the number one graduate from each medical school in Taiwan since 1981. We aimed to study whether TMG differ from ...

ProQuest

ProQuest

View all search results - see all results for your search including past figure & tables

Do more with documents in this alert at ProQuest See available formats(e.g.,Citation/Abstract, Full text, etc.). Export, email, and print documents.

Alert DetailsProQuest Search alert

ProQuest will send alerts **Weekly** until **November 04, 2020**.

**Alert name:**	ProQuest search of 5 databases
**Alert ID:**	680121
**Dates of coverage:**	May 13 2020 through May 20 2020
**Subject:**	(su(homosexuality OR gay OR lesbian OR homosexual OR homoerotic OR homophile OR bisexuality OR bisexual OR bigender OR LGB* OR GBL* OR queer) OR su(sexual minorit* OR men who have sex with men OR MSM OR women who have sex with women OR WSW OR (men who have sex with both men AND women) OR MSMW)) AND su(China OR Hong Kong OR Macau OR Taiwan OR Chinese OR Taiwanese) AND (su(Health OR Health Services OR Holistic Health OR Mental Health OR Emotion OR Mental Disorder OR Depression OR Stress OR Stigma OR Discri...
**Message:**	Scoping review

2 new documents found for **(su(homosexuality OR gay OR lesbian OR homosexual OR homoerotic OR homophile OR bisexuality OR bisexual OR**

Syntax of Simplified Chinese literature search

SinoMed (China Biological Medicine Database)

SinoMed (China Biological Medicine Database; 中国生物医学文献服务系统) - May 9, 2020		
Search	Query	Results
#1	("同性恋"[不加权:扩展]) OR "性少数群体"[不加权:扩展]	988
#2	同志"[关键词:智能] OR "同性爱"[关键词:智能] OR "双性恋"[关键词:智能] OR "男男性行为者"[关键词:智能] OR "女女性行为者"[关键词:智能] OR "MSM"[关键词:智能] OR "WSW"[关键词:智能] OR "LGB*"[关键词:智能]	851
#3	#1 OR #2	1,776
#4	((((((((((("健康状况"[不加权:扩展]) OR "健康行为"[不加权:扩展]) OR "卫生服务"[不加权:扩展]) OR "情感"[不加权:扩展]) OR "情绪障碍"[不加权:扩展] OR "情感症状"[不加权:扩展] OR "应激, 心理学"[不加权:扩展] OR "情绪"[不加权:扩展]) OR "物质相关性障碍"[不加权:扩展] OR "精神障碍"[不加权:扩展]) OR "抑郁"[不加权:扩展] OR "抑郁症"[不加权:扩展]) OR "社会歧视"[不加权:扩展]) OR "社会污点"[不加权:扩展]) OR "偏见"[不加权:扩展] OR "性别歧视"[不加权:扩展]) OR "自我概念"[不加权:扩展]) OR "认同(心理学)"[不加权:扩展] OR "社会认同"[不加权:扩展]) OR "自我伤害行为"[不加权:扩展]	2,285,589
#5	((((((((("性传播疾病"[不加权:扩展]) OR "HIV"[不加权:扩展] OR "HIV感染"[不加权:扩展]) OR "肉瘤, 卡波西"[不加权:扩展]) OR "生殖道感染"[不加权:扩展]) OR "女(雌)性泌尿生殖系统疾病"[不加权:扩展] OR "男(雄)性泌尿生殖系统疾病"[不加权:扩展]) OR "性行为"[不加权:扩展] OR "危险性行为"[不加权:扩展] OR "安全性行为"[不加权:扩展]) OR "性伴侣"[不加权:扩展]) OR "避孕套"[不加权:扩展]) OR "性力"[不加权:扩展]) OR "性功能障碍, 生理性"[不加权:扩展] OR "性功能障碍, 心理性"[不加权:扩展]) OR "性欲高潮"[不加权:扩展]	888,257
#6	((((((("生活质量"[不加权:扩展]) OR "暴力"[不加权:扩展] OR "家庭暴力"[不加权:扩展] OR "工作场所暴力"[不加权:扩展] OR "暴力遭遇"[不加权:扩展] OR "身体虐待"[不加权:扩展] OR "亲密伴侣暴力"[不加权:扩展]) OR "社会支持"[不加权:扩展]) OR "心理学, 社会"[不加权:扩展]) OR "社交网络"[不加权:扩展]) OR "性犯罪"[不加权:扩展]) OR "婚姻"[不加权:扩展] OR "婚姻状况"[不加权:扩展] OR "家庭冲突"[不加权:扩展]) OR "家庭关系"[不加权:扩展]	465,832
#7	"健康"[关键词:智能] OR "性健康"[关键词:智能] OR "性暴力"[关键词:智能] OR "强迫性性行为"[关键词:智能] OR "心理弹性"[关键词:智能] OR "自尊"[关键词:智能] OR "孤独"[关键词:智能] OR "无助"[关键词:智能] OR "心理弹性"[关键词:智能]	5,694

#8	#4 OR #5 OR #6 OR #7	3,111,819
#9	#3 AND #8	1,764
#10	Limited to publication between 2001-2020	1,655

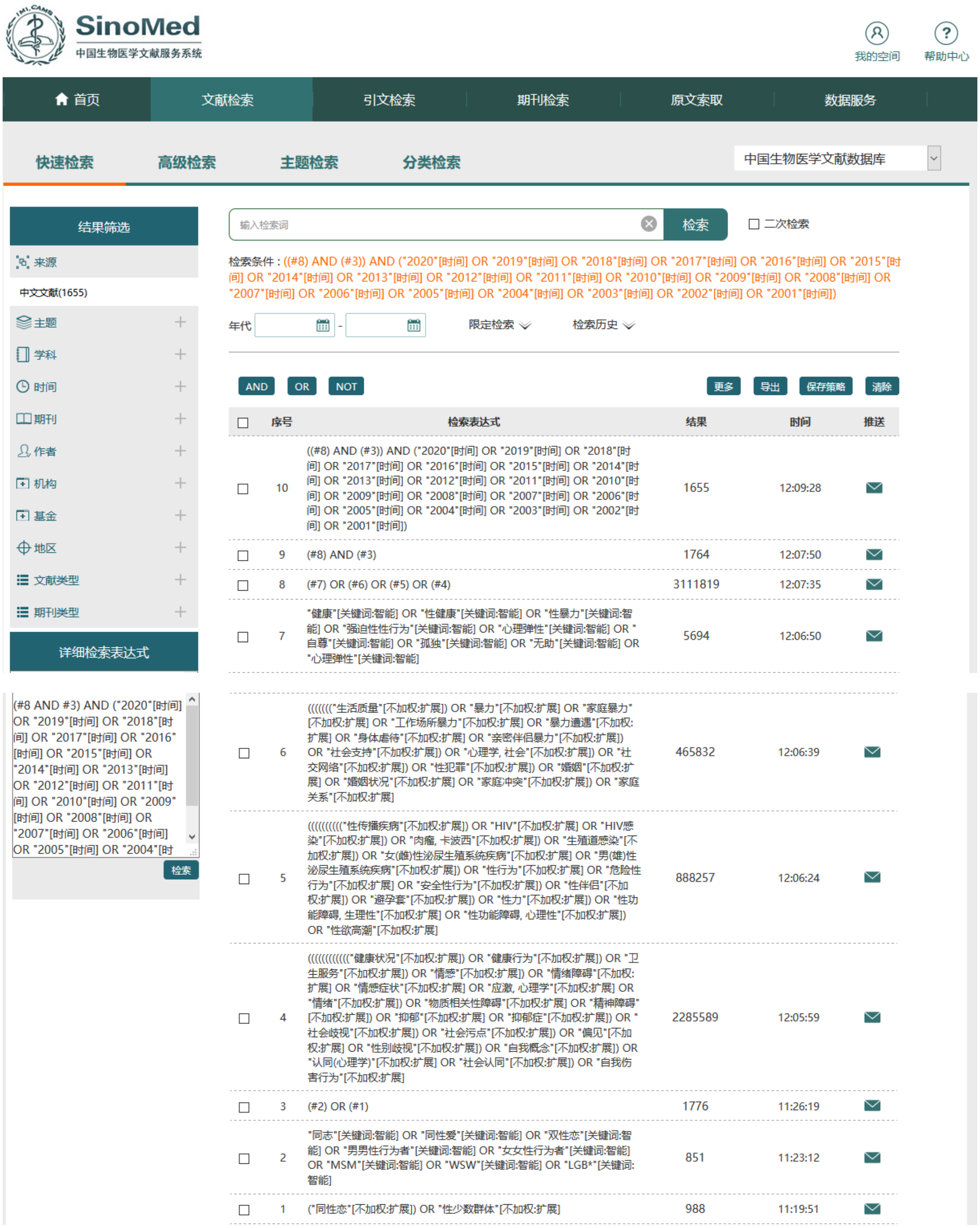

CNKI (China National Knowledge Infrastructure)

CNKI (China National Knowledge Infrastructure; 中国知网) – May 9, 2020		
Search	Query	Results
#1	((((((((主题=同义词扩展(同性恋)）或者（题名=同义词扩展	10,786

	(同性恋))) 或者 ((主题=同义词扩展(同性爱)) 或者 (题名=同义词扩展(同性爱)))) 或者 (((主题=同义词扩展(双性恋)) 或者 (题名=同义词扩展(双性恋))) 或者 ((主题=同义词扩展(性少数)) 或者 (题名=同义词扩展(性少数))))) 或者 (((主题=同义词扩展(LGB)) 或者 (题名=同义词扩展(LGB))) 或者 ((主题=同义词扩展(GBL)) 或者 (题名=同义词扩展(GBL))))) 或者 (((主题=同义词扩展(男男性行为者)) 或者 (题名=同义词扩展(男男性行为者))) 或者 ((主题=同义词扩展(MSM)) 或者 (题名=同义词扩展(MSM))))) 或者 (((主题=同义词扩展(女女性行为者)) 或者 (题名=同义词扩展(女女性行为者))) 或者 ((主题=同义词扩展(WSW)) 或者 (题名=同义词扩展(WSW))))) 或者 ((主题=同义词扩展(MSMW)) 或者 (题名=同义词扩展(MSMW)))) (模糊匹配),：全部; 数据库：文献 跨库检索	
#2	在结果中检索 #1 AND<br>并且 (((((((((主题=中英文扩展(健康)) 或者 (题名=同义词扩展(健康))) 或者 ((主题=同义词扩展(性传播疾病)) 或者 (题名=同义词扩展(性传播疾病)))) 或者 (((主题=同义词扩展(生活质量)) 或者 (题名=同义词扩展(生活质量))) 或者 ((主题=同义词扩展(焦虑)) 或者 (题名=同义词扩展(焦虑))))) 或者 (((主题=同义词扩展(抑郁)) 或者 (题名=同义词扩展(抑郁))) 或者 ((主题=同义词扩展(歧视)) 或者 (题名=同义词扩展(歧视))))) 或者 (((主题=同义词扩展(孤独)) 或者 (题名=同义词扩展(孤独))) 或者 ((主题=同义词扩展(精神障碍)) 或者 (题名=同义词扩展(精神障碍))))) 或者 (((主题=同义词扩展(性行为)) 或者 (题名=同义词扩展(性行为))) 或者 ((主题=同义词扩展(暴力)) 或者 (题名=同义词扩展(暴力))))) 或者 (((主题=同义词扩展(HIV)) 或者 (题名=同义词扩展(HIV))) 或者 ((主题=同义词扩展(心理问题)) 或者 (题名=同义词扩展(心理问题))))) 或者 (((主题=同义词扩展(社会支持)) 或者 (题名=同义词扩展(社会支持))) 或者 ((主题=同义词扩展(自我伤害)) 或者 (题名=同义词扩展(自我伤害))))) (模糊匹配),：全部; 数据库：文献 跨库检索<br>检索方式：跨库检索	4,594
#3	在结果中检索<br>#2 AND Limited to publication between 2001-2020<br>并且 发表时间 between (2001-01-01,2020-12-31)	4,290

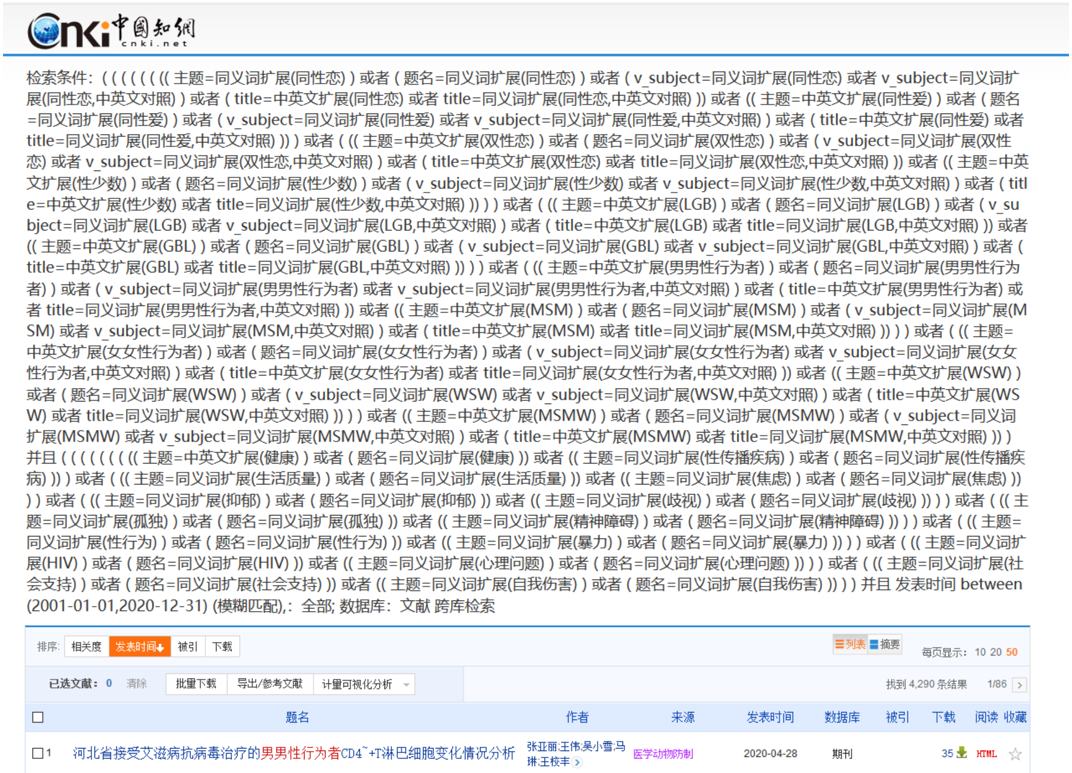

Wanfang Data

Wanfang Data (万方数据) – May 9, 2020		
Search	Query	Results
#1	主题:('同性恋'+'双性恋'+'性少数'+'男男性行为'+'MSM'+'女女性行为'+'WSW'+'LGB')*主题:('健康'+'性行为'+'性传播疾病'+'焦虑'+'抑郁'+'生活质量'+'社会支持'+'HIV'+'STD'+'STI'+'孤独'+'歧视'+'无助'+'污名'+'幸福')	1,913
#2	Limited to publication between 2001-2020	1,707
#3	Limited to Chinese language-限定语种为：中文	750

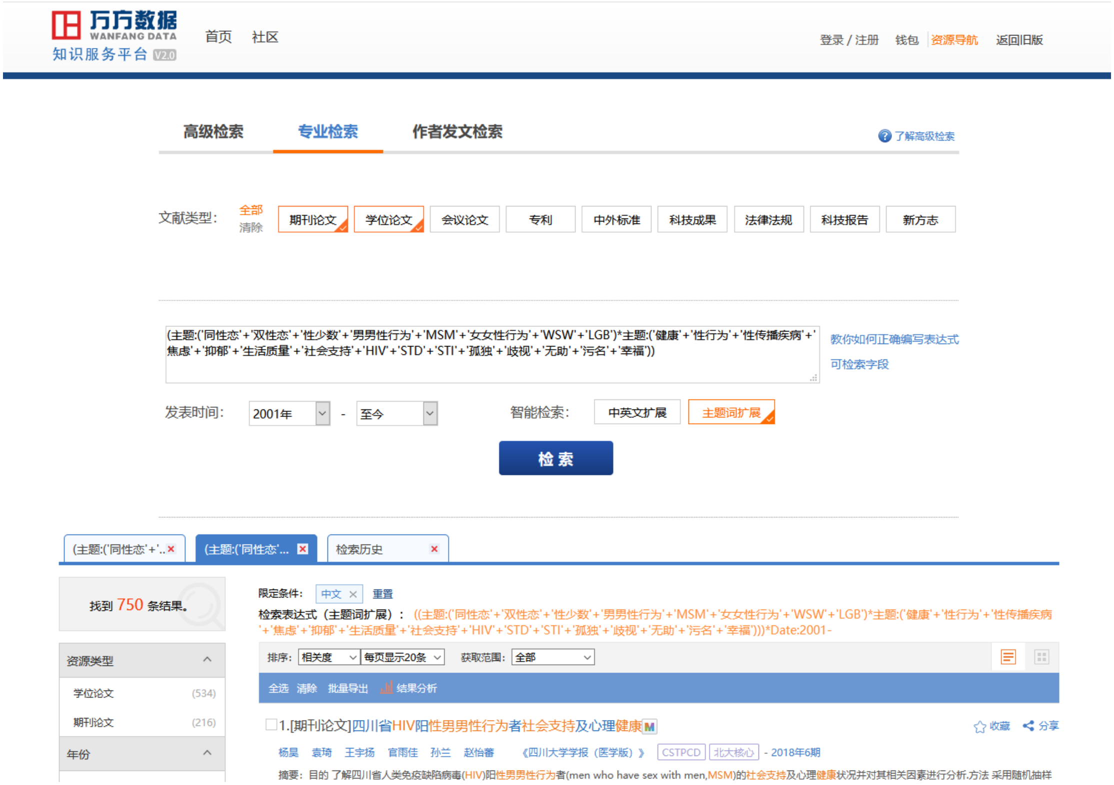

CQ VIP

CQ VIP (维普资讯) – May 9, 2020		
Search	Query	Results
#1	((((((((题名或关键词=健康 OR 题名或关键词=生活质量) OR 题名或关键词=社会支持) OR 题名或关键词=幸福) OR 题名或关键词=希望) OR 题名或关键词=心理弹性) OR (((((((((题名或关键词=性病 OR 题名或关键词=std) OR 题名或关键词=venereal) OR 题名或关键词=venereal disease) OR 题名或关键词=venereal diseases) OR 题名或关键词=生殖感染) OR 题名或关键词=性传播疾病) OR 题名或关键词=花柳毒淋) OR 题名或关键词=性传播性疾病) OR 题名或关键词=花柳病) OR 题名或关键词=淋病)) OR ((((((((题名或关键词=心理问题 OR 题名或关键词=心理失衡) OR 题名或关键词=焦虑) OR 题名或关键词=抑郁) OR 题名或关键词=孤独) OR 题名或关键词=无助) OR 题名或关键词=污名) OR 题名或关键词=歧视) OR 题名或关键词=暴力) OR 题名或关键词=自我伤害)) AND ((((((((题名或关键词=同性恋 OR 题名或关键词=双性恋) OR 题名或关键词=性少数) OR 题名或关键词=男男性行为者) OR 题名或关键词=men who have sex with men) OR 题名或关键词=msm) OR 题名或关键词=男男同性性行为者) OR 题名或关键词=男男性行为者人群) OR 题名或关键词=女女性行为者) OR 题名或关键词=WSW))	720
#2	Limited to publication between 2001-2020	643

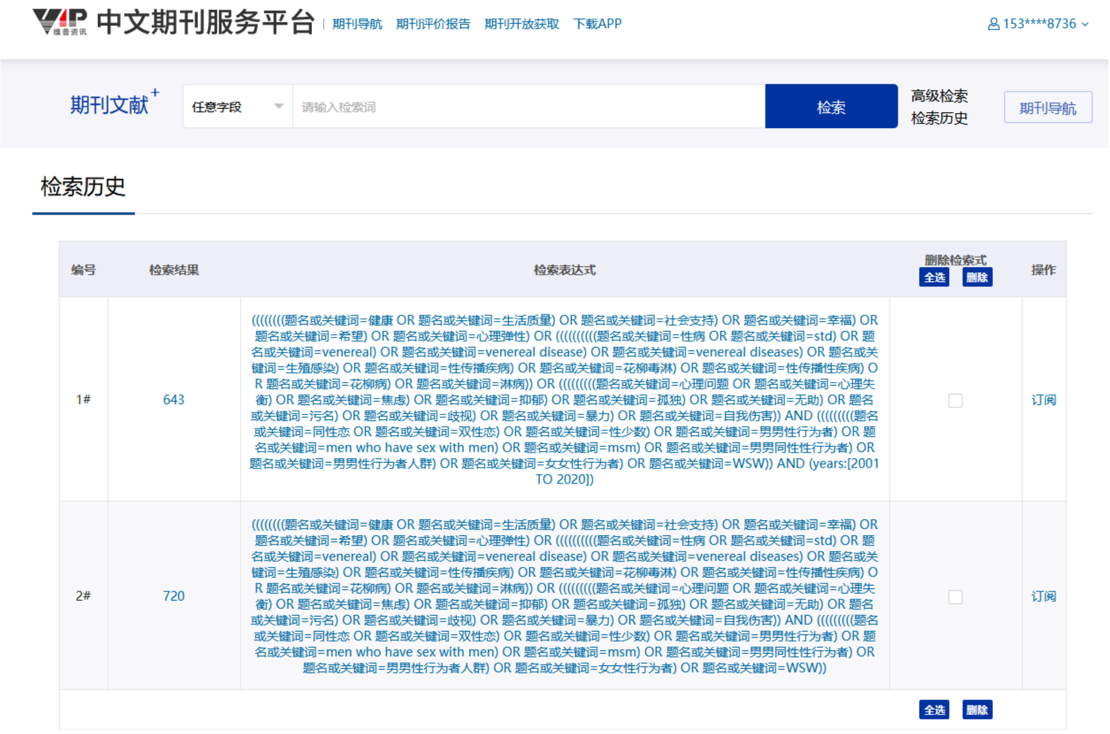

中文期刊服务平台 | 期刊导航 期刊评价报告 期刊开放获取 下载APP 153****8736

期刊文献 任意字段 请输入检索词 检索 高级检索 检索历史 期刊导航

检索历史

编号	检索结果	检索表达式	删除检索式 全选 删除	操作
1#	643	(((((((题名或关键词=健康 OR 题名或关键词=生活质量) OR 题名或关键词=社会支持) OR 题名或关键词=幸福) OR 题名或关键词=希望) OR 题名或关键词=心理弹性) OR ((((((((((题名或关键词=性病 OR 题名或关键词=std) OR 题名或关键词=venereal) OR 题名或关键词=venereal disease) OR 题名或关键词=venereal diseases) OR 题名或关键词=生殖感染) OR 题名或关键词=性传播疾病) OR 题名或关键词=花柳毒淋) OR 题名或关键词=性传播性疾病) OR 题名或关键词=花柳病) OR 题名或关键词=淋病)) OR (((((((((题名或关键词=心理问题 OR 题名或关键词=心理失衡) OR 题名或关键词=焦虑) OR 题名或关键词=抑郁) OR 题名或关键词=孤独) OR 题名或关键词=无助) OR 题名或关键词=污名) OR 题名或关键词=歧视) OR 题名或关键词=暴力) OR 题名或关键词=自我伤害)) AND (((((((((题名或关键词=同性恋 OR 题名或关键词=双性恋) OR 题名或关键词=性少数) OR 题名或关键词=男男性行为者) OR 题名或关键词=men who have sex with men) OR 题名或关键词=msm) OR 题名或关键词=男男同性性行为者) OR 题名或关键词=男男性行为者人群) OR 题名或关键词=女女性行为者) OR 题名或关键词=WSW)) AND (years:[2001 TO 2020])		订阅
2#	720	(((((((题名或关键词=健康 OR 题名或关键词=生活质量) OR 题名或关键词=社会支持) OR 题名或关键词=幸福) OR 题名或关键词=希望) OR 题名或关键词=心理弹性) OR ((((((((((题名或关键词=性病 OR 题名或关键词=std) OR 题名或关键词=venereal) OR 题名或关键词=venereal disease) OR 题名或关键词=venereal diseases) OR 题名或关键词=生殖感染) OR 题名或关键词=性传播疾病) OR 题名或关键词=花柳毒淋) OR 题名或关键词=性传播性疾病) OR 题名或关键词=花柳病) OR 题名或关键词=淋病)) OR (((((((((题名或关键词=心理问题 OR 题名或关键词=心理失衡) OR 题名或关键词=焦虑) OR 题名或关键词=抑郁) OR 题名或关键词=孤独) OR 题名或关键词=无助) OR 题名或关键词=污名) OR 题名或关键词=歧视) OR 题名或关键词=暴力) OR 题名或关键词=自我伤害)) AND (((((((((题名或关键词=同性恋 OR 题名或关键词=双性恋) OR 题名或关键词=性少数) OR 题名或关键词=男男性行为者) OR 题名或关键词=men who have sex with men) OR 题名或关键词=msm) OR 题名或关键词=男男同性性行为者) OR 题名或关键词=男男性行为者人群) OR 题名或关键词=女女性行为者) OR 题名或关键词=WSW))		订阅

全选 删除

Syntax of Traditional Chinese literature search

Taiwan citation index-humanities and social sciences

Taiwan citation index-humanities and social sciences (臺灣人文及社會科學引文索引資料庫) - May 9, 2020		
Search	Query	Results
#1	"同性戀 or gay or 拉拉".kw or "雙性戀".kw or "性少數 or LGB or GBL".kw or "男男性行為 or MSM".kw or "女女性行為 or WSW".kw and ((jsb1="綜合" or jsb1="人類學" or jsb1="心理學" or jsb1="社會學" or jsb1="傳播學" or jsb1="管理學" or jsb1="體育學" or jsb1="圖書資訊學") and (stype="*") and lg="中文")	1,133
#2	"健康 or 生活質量 or 生存質量 or 社會支持 or 幸福".kw or "性傳播疾病 or HIV or STD or STI or 性行為 or 暴力 or 心理疾病 or 焦慮 or 抑鬱 or 孤獨 or 無助 or 自我傷害 or 自殺".kw and ((jsb1="綜合" or jsb1="人類學" or jsb1="心理學" or jsb1="社會學" or jsb1="傳播學" or jsb1="管理學" or jsb1="體育學" or jsb1="圖書資訊學") and (stype="*") and lg="中文")	47,170
#3	#1 and #2	385
#4	Limited to publication between 2001-2020	321

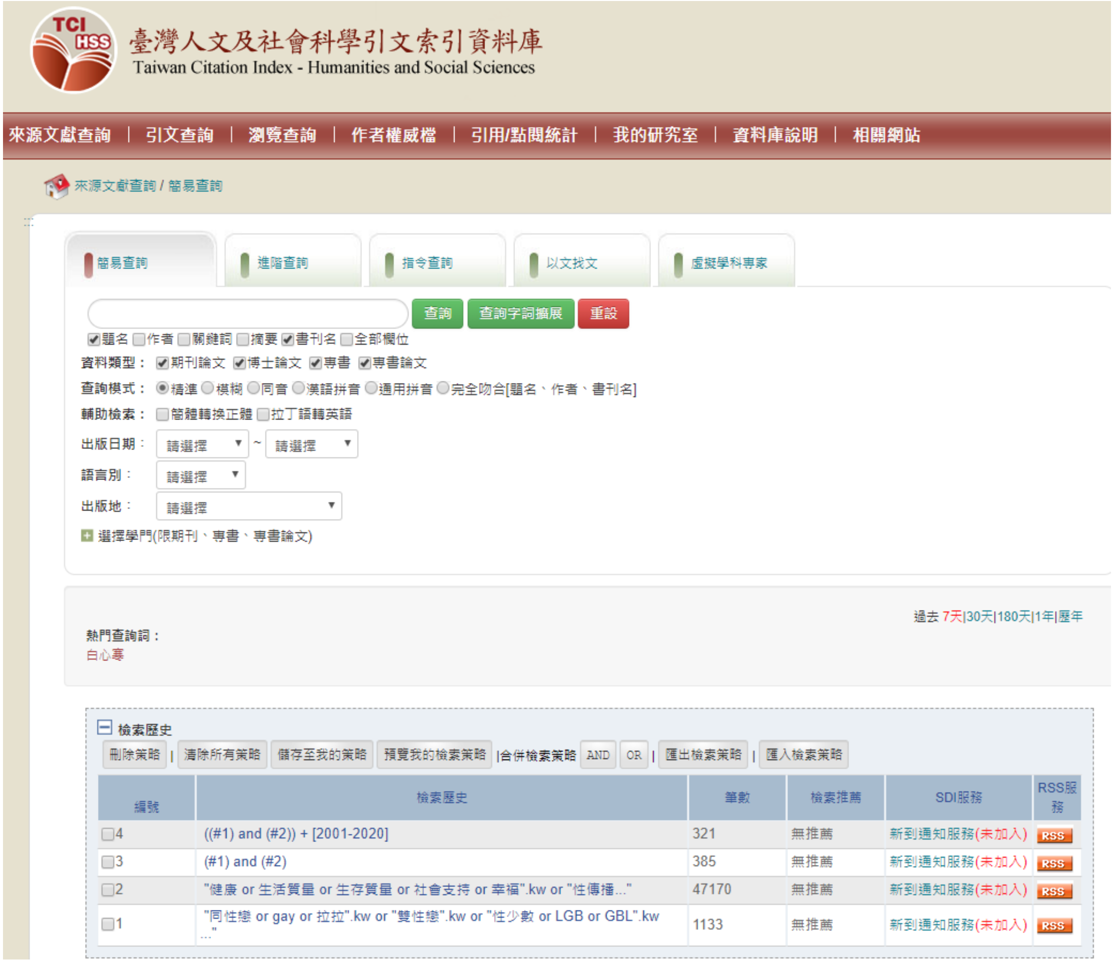

Index to Taiwan periodical literature system

Index to Taiwan periodical literature system (臺灣期刊論文索引系統) - May 9, 2020	
Search Query	Results
(KW=同性戀 OR gay OR 拉拉OR 雙性戀 OR 性少數 OR LGB OR GBL OR 男男性行為 OR MSM OR 女女性行為 OR WSW) [AND] (KW=健康 OR 生活質量 OR 生存質量 OR 社會支持 OR 幸福 OR 性傳播疾病 OR HIV OR 性行為 OR 暴力 OR 心理疾病 OR 焦慮 OR 抑鬱 OR 孤獨 OR 無助 OR 自我傷害 OR 自殺) [AND] (20010101<=PD<=20201231) [AND] (LA=chi)	41

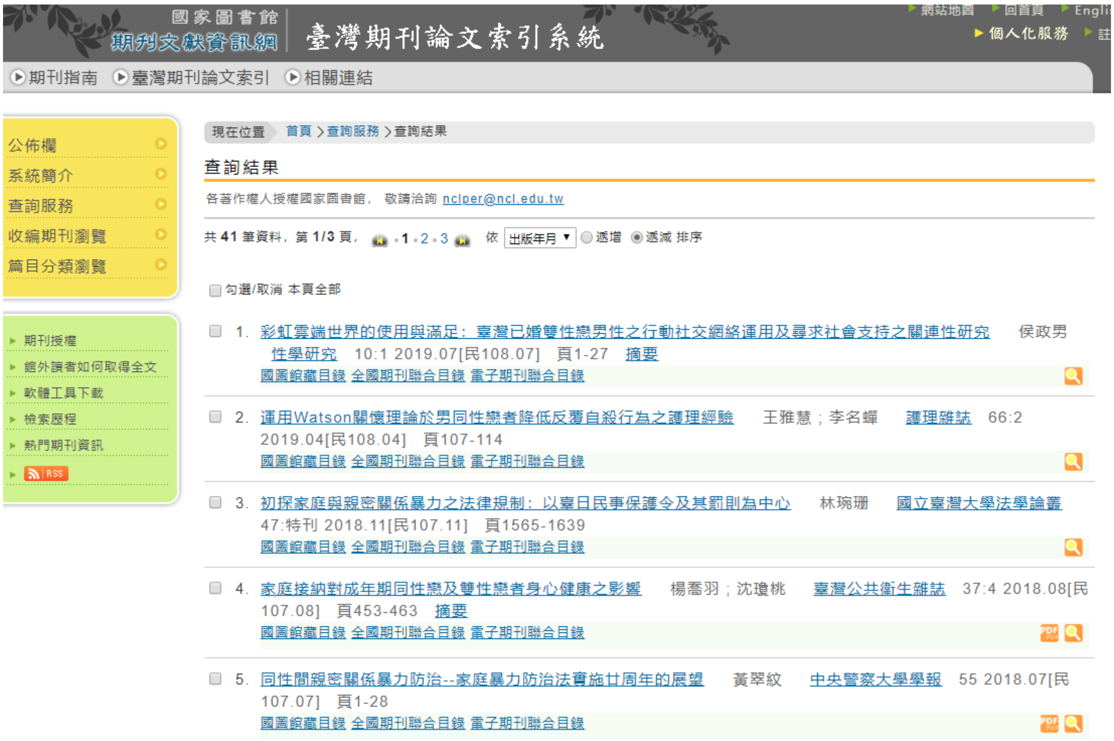

Synergy of metadata resources in Taiwan

Synergy of metadata resources in Taiwan (臺灣書目整合查詢系統) - May 9, 2020		
Search	Query	Results
#1	(同性戀)@KW,CL OR (雙性戀)@KW,CL OR (性少數)@KW,CL OR (男男性行為者)@KW,CL OR (MSM)@KW,CL OR (女女性行為者)@KW,CL OR (WSW)@KW,CL AND YR=2001:2020[YR=出版年Pub.Year,KW=關鍵字Keyword,CL=主題Subject]	1,238
#2	(健康)@KW,CL OR (生活質量 OR 生存質量 OR 社會支持 OR 幸福)@KW,CL OR (性傳播疾病 OR HIV OR STD OR STI OR 性行為)@KW,CL OR (暴力 OR 自我傷害 OR 自殺)@KW,CL OR (心理疾病 OR 焦慮 OR 抑鬱 OR 孤獨 OR 無助)@KW,CL OR (歧視 OR 污名)@KW,CL OR (愉悅 OR 心理彈性)@KW,CL AND YR=2001:2020[YR=出版年Pub.Year,KW=關鍵字Keyword,CL=主題Subject]	60,332
#3	#1 AND #2	84

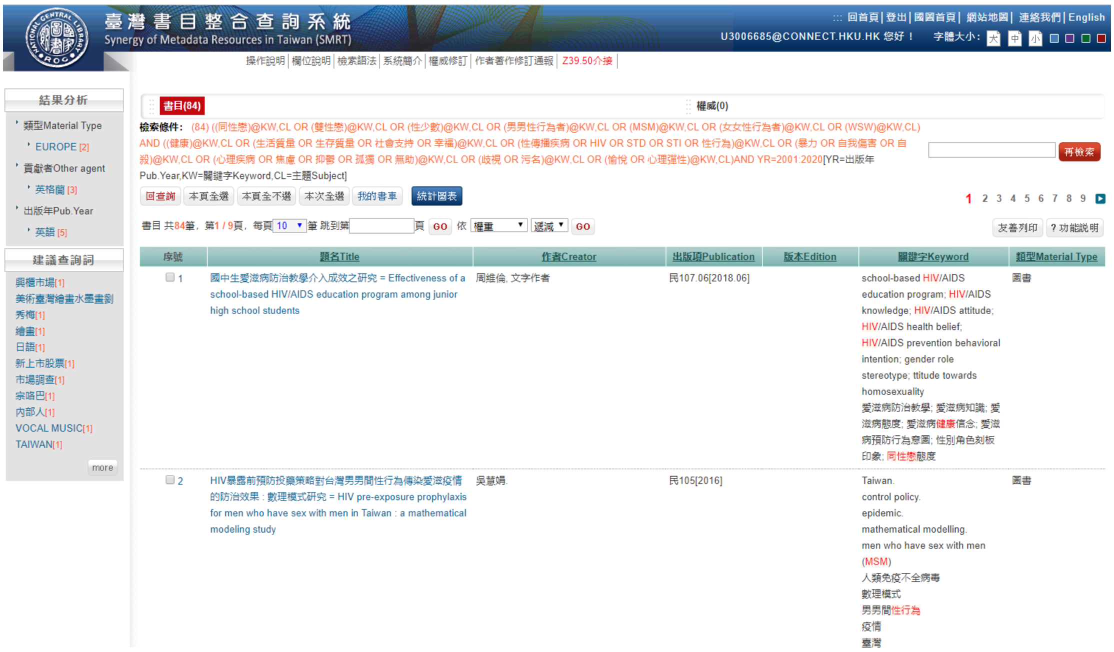

National Digital Library of Theses and Dissertation in Taiwan

National Digital Library of Theses and Dissertation in Taiwan (臺灣博碩士論文知識加值系統) - May 9, 2020		
Search	Query	Results
#1	("同性戀".kw or "雙性戀".kw or "性少數".kw or "男男性行為者".kw or "女女性行為者".kw)/lg="中文",yr="90-109",ty="博士" AND Limited to publication between 2001(民国90年)-2020(民国109), (模糊) Chinese language	2,902
#2	("健康".kw or "生活質量 or 生存質量".kw or "性傳播疾病 or STD or STI".kw or "HIV".kw or "社會支持".kw or "心理問題".kw or "性行為".kw or "暴力".kw or "焦慮 or 抑鬱 or 歧視 or 孤獨".kw or "自我傷害 or 自殺".kw)/lg="中文",yr="90-109",ty="博士" AND Limited to publication between 2001(民国90年)-2020(民国109), (模糊) Chinese language	7,495
#3	#1 AND #2	1,495

*(精確) search, result = 0

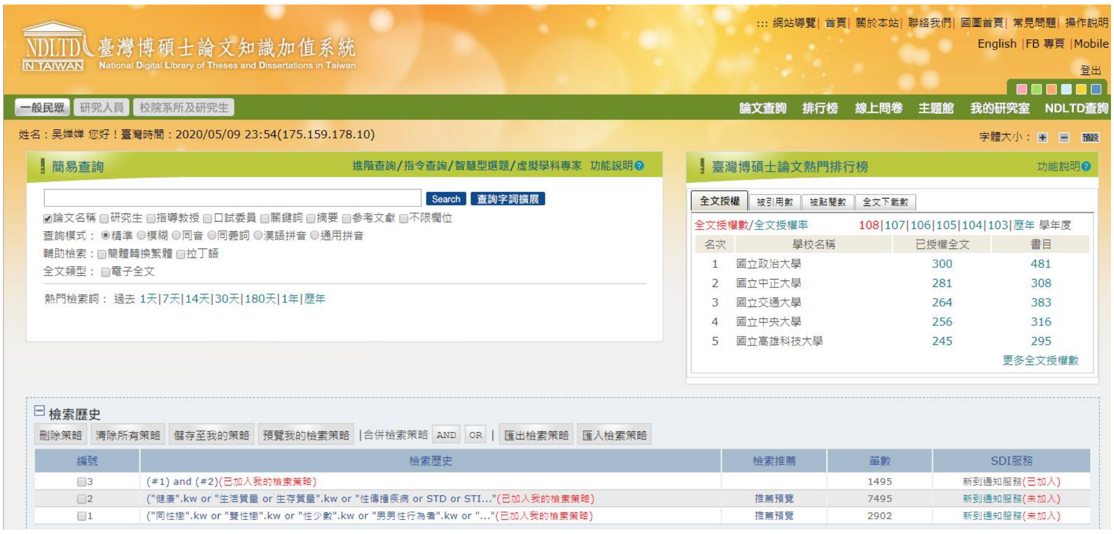

Updates checking by E-mail alerts

ProQuest databases (till December 30, 2020)

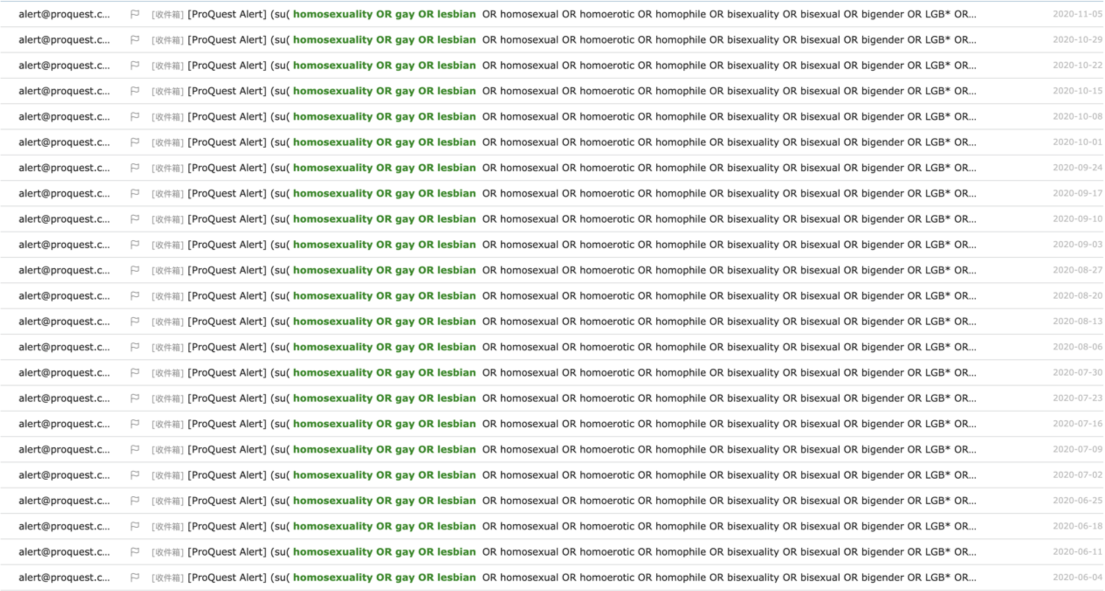

alert@proquest.c...	[收件箱] [ProQuest Alert] (su(homosexuality OR gay OR lesbian OR homosexual OR homoerotic OR homophile OR bisexuality OR bisexual OR bigender OR LGB* OR...	2020-11-05
alert@proquest.c...	[收件箱] [ProQuest Alert] (su(homosexuality OR gay OR lesbian OR homosexual OR homoerotic OR homophile OR bisexuality OR bisexual OR bigender OR LGB* OR...	2020-10-29
alert@proquest.c...	[收件箱] [ProQuest Alert] (su(homosexuality OR gay OR lesbian OR homosexual OR homoerotic OR homophile OR bisexuality OR bisexual OR bigender OR LGB* OR...	2020-10-22
alert@proquest.c...	[收件箱] [ProQuest Alert] (su(homosexuality OR gay OR lesbian OR homosexual OR homoerotic OR homophile OR bisexuality OR bisexual OR bigender OR LGB* OR...	2020-10-15
alert@proquest.c...	[收件箱] [ProQuest Alert] (su(homosexuality OR gay OR lesbian OR homosexual OR homoerotic OR homophile OR bisexuality OR bisexual OR bigender OR LGB* OR...	2020-10-08
alert@proquest.c...	[收件箱] [ProQuest Alert] (su(homosexuality OR gay OR lesbian OR homosexual OR homoerotic OR homophile OR bisexuality OR bisexual OR bigender OR LGB* OR...	2020-10-01
alert@proquest.c...	[收件箱] [ProQuest Alert] (su(homosexuality OR gay OR lesbian OR homosexual OR homoerotic OR homophile OR bisexuality OR bisexual OR bigender OR LGB* OR...	2020-09-24
alert@proquest.c...	[收件箱] [ProQuest Alert] (su(homosexuality OR gay OR lesbian OR homosexual OR homoerotic OR homophile OR bisexuality OR bisexual OR bigender OR LGB* OR...	2020-09-17
alert@proquest.c...	[收件箱] [ProQuest Alert] (su(homosexuality OR gay OR lesbian OR homosexual OR homoerotic OR homophile OR bisexuality OR bisexual OR bigender OR LGB* OR...	2020-09-10
alert@proquest.c...	[收件箱] [ProQuest Alert] (su(homosexuality OR gay OR lesbian OR homosexual OR homoerotic OR homophile OR bisexuality OR bisexual OR bigender OR LGB* OR...	2020-09-03
alert@proquest.c...	[收件箱] [ProQuest Alert] (su(homosexuality OR gay OR lesbian OR homosexual OR homoerotic OR homophile OR bisexuality OR bisexual OR bigender OR LGB* OR...	2020-08-27
alert@proquest.c...	[收件箱] [ProQuest Alert] (su(homosexuality OR gay OR lesbian OR homosexual OR homoerotic OR homophile OR bisexuality OR bisexual OR bigender OR LGB* OR...	2020-08-20
alert@proquest.c...	[收件箱] [ProQuest Alert] (su(homosexuality OR gay OR lesbian OR homosexual OR homoerotic OR homophile OR bisexuality OR bisexual OR bigender OR LGB* OR...	2020-08-13
alert@proquest.c...	[收件箱] [ProQuest Alert] (su(homosexuality OR gay OR lesbian OR homosexual OR homoerotic OR homophile OR bisexuality OR bisexual OR bigender OR LGB* OR...	2020-08-06
alert@proquest.c...	[收件箱] [ProQuest Alert] (su(homosexuality OR gay OR lesbian OR homosexual OR homoerotic OR homophile OR bisexuality OR bisexual OR bigender OR LGB* OR...	2020-07-30
alert@proquest.c...	[收件箱] [ProQuest Alert] (su(homosexuality OR gay OR lesbian OR homosexual OR homoerotic OR homophile OR bisexuality OR bisexual OR bigender OR LGB* OR...	2020-07-23
alert@proquest.c...	[收件箱] [ProQuest Alert] (su(homosexuality OR gay OR lesbian OR homosexual OR homoerotic OR homophile OR bisexuality OR bisexual OR bigender OR LGB* OR...	2020-07-16
alert@proquest.c...	[收件箱] [ProQuest Alert] (su(homosexuality OR gay OR lesbian OR homosexual OR homoerotic OR homophile OR bisexuality OR bisexual OR bigender OR LGB* OR...	2020-07-09
alert@proquest.c...	[收件箱] [ProQuest Alert] (su(homosexuality OR gay OR lesbian OR homosexual OR homoerotic OR homophile OR bisexuality OR bisexual OR bigender OR LGB* OR...	2020-07-02
alert@proquest.c...	[收件箱] [ProQuest Alert] (su(homosexuality OR gay OR lesbian OR homosexual OR homoerotic OR homophile OR bisexuality OR bisexual OR bigender OR LGB* OR...	2020-06-25
alert@proquest.c...	[收件箱] [ProQuest Alert] (su(homosexuality OR gay OR lesbian OR homosexual OR homoerotic OR homophile OR bisexuality OR bisexual OR bigender OR LGB* OR...	2020-06-18
alert@proquest.c...	[收件箱] [ProQuest Alert] (su(homosexuality OR gay OR lesbian OR homosexual OR homoerotic OR homophile OR bisexuality OR bisexual OR bigender OR LGB* OR...	2020-06-11
alert@proquest.c...	[收件箱] [ProQuest Alert] (su(homosexuality OR gay OR lesbian OR homosexual OR homoerotic OR homophile OR bisexuality OR bisexual OR bigender OR LGB* OR...	2020-06-04

Web of science (till April 30, 2021)

alerts-noreply	[收件箱] Web of Science Alert - WoS- **Scoping review of health** - 7 results	4月29日
alerts-noreply	[收件箱] Web of Science Alert - WoS- **Scoping review of health** - 6 results	4月22日
alerts-noreply	[收件箱] Web of Science Alert - WoS- **Scoping review of health** - 9 results	4月15日
alerts-noreply	[客户端删信] Web of Science Alert - WoS- **Scoping review of health** - 3 results	4月1日
alerts-noreply	[收件箱] Web of Science Alert - WoS- **Scoping review of health** - 10 results	3月25日
alerts-noreply	[收件箱] Web of Science Alert - WoS- **Scoping review of health** - 10 results	3月18日
alerts-noreply	[收件箱] Web of Science Alert - WoS- **Scoping review of health** - 8 results	3月11日
alerts-noreply	[收件箱] Web of Science Alert - WoS- **Scoping review of health** - 2 results	3月4日
alerts-noreply	[收件箱] Web of Science Alert - WoS- **Scoping review of health** - 5 results	2月25日
alerts-noreply	[收件箱] Web of Science Alert - WoS- **Scoping review of health** - 7 results	2月19日
alerts-noreply	[收件箱] Web of Science Alert - WoS- **Scoping review of health** - 10 results	2月11日
alerts-noreply	[收件箱] Web of Science Alert - WoS- **Scoping review of health** - 2 results	2月4日
alerts-noreply	[收件箱] Web of Science Alert - WoS- **Scoping review of health** - 9 results	1月28日
alerts-noreply	[收件箱] Web of Science Alert - WoS- **Scoping review of health** - 7 results	1月21日
alerts-noreply	[收件箱] Web of Science Alert - WoS- **Scoping review of health** - 4 results	1月14日
alerts-noreply	[收件箱] Web of Science Alert - WoS- **Scoping review of health** - 8 results	1月7日
alerts-noreply	[收件箱] Web of Science Alert - WoS- **Scoping review of health** - 4 results	2020-12-31
alerts-noreply	[收件箱] Web of Science Alert - WoS- **Scoping review of health** - 6 results	2020-12-24
alerts-noreply	[收件箱] Web of Science Alert - WoS- **Scoping review of health** - 5 results	2020-12-10
alerts-noreply	[收件箱] Web of Science Alert - WoS- **Scoping review of health** - 8 results	2020-12-03
alerts-noreply	[收件箱] Web of Science Alert - WoS- **Scoping review of health** - 9 results	2020-11-26
alerts-noreply	[收件箱] Web of Science Alert - WoS- **Scoping review of health** - 3 results	2020-11-19
alerts-noreply	[收件箱] Web of Science Alert - WoS- **Scoping review of health** - 3 results	2020-11-12
alerts-noreply	[收件箱] Web of Science Alert - WoS- **Scoping review of health** - 5 results	2020-11-05
alerts-noreply	[收件箱] Web of Science Alert - WoS- **Scoping review of health** - 6 results	2020-10-22
alerts-noreply	[收件箱] Web of Science Alert - WoS- **Scoping review of health** - 5 results	2020-10-10
alerts-noreply	[收件箱] Web of Science Alert - WoS- **Scoping review of health** - 3 results	2020-10-01
alerts-noreply	[收件箱] Web of Science Alert - WoS- **Scoping review of health** - 1 results	2020-09-24
alerts-noreply	[收件箱] Web of Science Alert - WoS- **Scoping review of health** - 6 results	2020-09-17
alerts-noreply	[收件箱] Web of Science Alert - WoS- **Scoping review of health** - 5 results	2020-09-10
alerts-noreply	[收件箱] Web of Science Alert - WoS- **Scoping review of health** - 4 results	2020-08-27
alerts-noreply	[收件箱] Web of Science Alert - WoS- **Scoping review of health** - 7 results	2020-08-20
alerts-noreply	[收件箱] Web of Science Alert - WoS- **Scoping review of health** - 6 results	2020-08-13
alerts-noreply	[收件箱] Web of Science Alert - WoS- **Scoping review of health** - 10 results	2020-07-30
alerts-noreply	[收件箱] Web of Science Alert - WoS- **Scoping review of health** - 2 results	2020-07-02
alerts-noreply	[收件箱] Web of Science Alert - WoS- **Scoping review of health** - 3 results	2020-06-25
alerts-noreply	[收件箱] \n Web of Science Alert - WoS- **Scoping review of health** - 5 results\n\n\n	2020-06-11
alerts-noreply	[收件箱] Web of Science Alert - WoS- **Scoping review of health** - 6 results	2020-06-04
