# Supplementary material for: The Holistic Health Status of Chinese Homosexual and Bisexual Adults: A Scoping Review
Source: Front Public Health. 2021 Aug 24;9:710575. doi: 10.3389/fpubh.2021.710575 (PMC8421524; doi:10.3389/fpubh.2021.710575)
Supplement: Supplementary file 4 [file Data_Sheet_4.pdf]

Summary of searching of English databases and study selection

English databases									
	**PubMed**	**Web of Science**	**CINAHL**	**ScienceDirect**	**Social Work Abstract**	**ProQuest***	**Scopus**	**Cochrane**	**JBI EBP**
Date (2020)	May 7	May 12	May 8	May 8	May 8	May 8	May 8	May 8	May 8
**Searching results referring to PCC**	**1213**	**2106**	**421**	**140**	**14**	**853**	**622**	**132**	**17**
**Total search**	**5518**								
**Updates until May 31**	**+14**								
Duplicate publications	2577								
**Total left to screen**	**2955**								
**Title screening**	Exclude **1642** = 1466 non-PCC related + 48 non-academic references + 128 further duplications								
Publications remained after screening title	**1313**								
**Abstract screening**	Exclude **338** = 83 (Non-Population) + 146 (Non-Health) + 69 (Non-Context) + 40 (Others)								
Publications remained after screening abstracts	**975**								
**Full text screening**	Exclude **272** (see details in ***Studies ineligible following full text screening-Eng***)								
Publications remained after screening full text	**703**								
**Plus publications from references of eligible studies**	**5**								
**Total included publications**	**708** (14 theses + 694 articles)								

*ProQuest searching of the following 5 databases: 1) ProQuest Dissertations & Theses A&I; 2) ProQuest Dissertations & Theses Global; 3) APA PsycInfo; 4) Sociological Abstracts; 5) Social Services Abstracts.

Summary of searching of Traditional Chinese databases and study selection

**Traditional Chinese databases**				
**Databases**	**Taiwan citation index-humanities and social sciences (臺灣人文及社會科學引文索引資料庫)**	**Index to Taiwan periodical literature system (臺灣期刊論文索引系統)**	**Synergy of metadata resources in Taiwan (臺灣書目整合查詢系統)**	**National Digital Library of Theses and Dissertation in Taiwan (臺灣博碩士論文知識加值系統)**
Date	May 9, 2020	May 9, 2020	May 9, 2020	May 9, 2020
**Searching results referring to PCC**	**321**	**41**	**84**	**1,495**
**Total search**	**1,941**			
Duplicate publications	8			
**Total left to screen**	**1,933**			
Further duplicates	Exclude 33			
**Title screening**	Exclude **1,797** (Non PCC-related)			
Publications remained after screening title	**103**			
**Abstract screening**	Exclude **62** = 37 (Non-Population) + 22 (Non-Health) + 2 (Non-Context) + 1 (Other)			
Publications remained after screening abstracts	**41** (14 theses+ 27 articles)			
**Full text screening**	Exclude **21** (see details in ***Studies ineligible following full text screening-TC***)			
Publications remained after screening full text	**20**			
**Total included publications**	**20** (6 theses+ 14 articles)			

Summary of searching of Simplified Chinese databases and study selection

**Simplified Chinese databases**				
**Databases**	**SinoMed (China Biological Medicine Database; 中国生物医学文献服务系统)**	**CNKI (China National Knowledge Infrastructure; 中国知网)**	**Wanfang Data (万方数据)**	**CQ VIP (维普资讯)**
Date	May 9, 2020	May 9, 2020	May 9, 2020	May 9, 2020
**Searching results referring to PCC**	**1655**	**4290**	**750**	**643**
**Total search**	**7338**#			
Duplicate publications	1642			
**Total left to screen**	**5696**#			
**Title screening**	Exclude: **2094** = 34(Translations) + 334 (Non-academic) + 97(Further duplicates) + 1629 (Non PCC-related)			
Articles excluded according to the quality of journals	Exclude: **89** (publications from very low-quality journals*)			
Publications remained after screening title	**3513** (439 theses + 3074 articles)			
**Abstract screening**	**Theses exclude: 185 =** 95 (Non-Population) + 89 (Non-health) + 1 (Non-Context)			
	**Articles exclude: 743 =** 413 (Non-Population) + 290 (Non-health) + 25 (Non-Context) + 15 others			
Publications remained after screening abstracts	**2585** (254 theses+ 2331 articles)			
**Full text screening**	**Exclude: 434 = 40 theses + 394 articles** (see details in the ***Studies ineligible following full text screening-CN***)			
**Total included publications**	**2151** (214 theses+ 1937 articles)			

#: Journals do not belong to any core journals included in any of the three criteria and have no impact factors. [Four 'Nos']

Chinese Core Journals according to three standard criteria, namely: Key magazine of China technology(中国科技核心 CSTPCD), Chinese core journal criterion of PKU(中文核心期刊) and Chinese Science Citation Database (中国科学引文数据库 CSCD).

- Non-academic references if publications come from book chapter, or they are news/ letters-to-editor/ study protocols/ editorial commentaries or notes;
- Further duplications if publications are duplicate records manually found.
- Non-PCC criteria:
  - Non-Population related refers to 'studies did not target on homosexual or bisexual adults, or epidemiology of HIV/STI among all risk populations only; besides, studies only investigating Money Boys (MB) or Male sex workers were not included since it is not about sexual orientation.'
  - Non-Health related refers to 'studies did not investigate health-related domains; or research is not human-centered, but only genetic analysis or molecular epidemiology, or laboratory/ serological tests, or just prediction model, or merely the evaluation of the effectiveness of drugs or testing methods, or just population size estimation, or the development/ validation of some scales/measurements, or studies only analyzed the causes of sexual orientation (homosexuality), or studies merely focused on minority law/ rights.'
  - Non-Context related refers to 'study settings were not any part of China or participants were non-Chinese, or the location of the investigation is unknown or unclear, or the studies were conducted in the year before 2001, or global reviews/ Asia studies (without specific descriptions of China).'
  - "Others" refers to 'publication erratum/ corrections, or study protocols/ ongoing trials, or commentaries, or book chapters, or (conference) abstracts without full-text, or news, or English records of Chinese publications', or 'Translation of English publications or reports on the treatment of single case with multiple HIV/STI-related symptoms'.

Studies ineligible following full text screening-English

Record of Excluded studies	Reference type	Reasons for exclusion	Summary
[1-12]	Journal article	MSM population was one sub-group among all other high-risk HIV groups, while no specific analysis focusing on the MSM group.	**Non-Population**<br><br>**N=63**<br><br>Did not meet the inclusion criteria in terms of study population
[13]	Journal article	This paper reviewed invasive amebiasis by analyzing serial blood and stool samples from HIV-infected patients, but not recruited MSM.	
[14,15]	Journal article	The populations were attendees of gay bars or gay bathhouse without mentioning their sexual orientation or identifications.	
[16]	Journal article	This study interviewed bathhouse staff members and also bathhouse patrons without mentioning their sexual orientation or sexual behavior.	
[17]	Journal article	Though study populations were sexual minorities, there were no descriptions of their specific sexual orientation and this study included people <18.	
[18,19]	Journal article	Study populations were homosexual male senior high school students, or gay (tongzhi) students in secondary schools aged 14-18.	
[20]	Journal article	Study included a large sample of general adults in the north eastern China.	
[21]	Journal article	Study investigated formality marriage (Xinghun) by analyzing ads posted on Tianya.cn, while not included any actual population.	
[22]	Journal article	Study populations were individuals who visited VCT sites in Taiwan.	
[23]	Journal article	Study recruited all residents with minority regions but not sexual minorities.	
[24-27]	Journal article	The articles summarized work of community-based organizations (CBO), or described more stakeholders' views than MSM's.	
[28]	Journal article	Study was a case study of a public place in Guangzhou (People's park).	
[29-32]	Journal article	The population of these studies were college students or young adults.	
[33-40]	Journal article	Study populations were HIV-positive men or people infected by Hepatitis A or Shigellosis.	

[41]	Journal article	The target population of this study was rural-to-urban migrants who were at high risk of HIV infection.	
[42]	Journal article	Study population was people at risk of hypertension.	
[43]	Journal article	This article reviewed the MSM-associated HIV cases among blood donors.	
[44,45]	Journal article	Study populations were male sex workers (money boys) only.	
[46]	Journal article	Study was a youth survey comparing their disparities by sex and living areas.	
[47]	Journal article	Study populations were LGBQ students (age 15-19).	
[48-50]	Journal article	Study populations were wives of MSM (tongqi), or women who have regular sexual relations with MSM.	
[51,52]	Journal article	There were no clear descriptions of the study populations, only categorized as queer or non-heterosexual people.	
[53]	Journal article	Study populations included both ethic Chinese and overseas sexual minorities and also public who lived in Hong Kong.	
[54]	Journal article	Studies explored feasibility or sustainability issues of non-governmental organizations (NGOs), but not targeted on MSM or other populations.	
[55]	Journal article	Study populations were mainly young adults in a university population and only small part of sexual minorities without mentioning their orientation.	
[56]	Journal article	Participants were Mainland Chinese citizens identifying themselves with a heterosexual orientation.	
[57]	Journal article	Study populations were male attendees from STD clinic.	
[58]	Journal article	Study populations were physicians of STD treatments.	
[59]	Journal article	Study collected only urine samples of potential MSM.	
[60]	Journal article	Study populations were people who interested in improving clinical services for MSM.	
[61]	Journal article	Study populations were all adults in Taiwan and no clear description of nonheterosexual people.	
[62,63]	Journal article	Study analyzed only MSM apps or other online communities/networks.	

[64]	Journal article	This study only conducted from family's perspective, while did not mention or investigate health-related question.	**Non-Concept (health)**<br><br>**N=97**<br><br>Did not meet the inclusion criteria in terms of concept, such as studies that focused on basic science issues.
[65]	Journal article	No clear description of the study, no details of either participants, methods or outcomes, and not health related.	
[66,67]	Journal article	Did not mention or investigate health-related question.	
[68-71]	Journal article	These articles mainly discussed or compared some sampling methods.	
[72]	Thesis	This study investigated only some stages of being sexual minority, but no more investigations of health-related issues.	
[73-75]	Journal article	Studies re-analyzed previous data about screening tests/methods only.	
[76-81]	Journal article	Studies investigated the hemodynamic brain response, function or activation of sexual stimuli but did not investigate health from the person's perspective.	
[82-88]	Journal article	These papers only described some stories, experiences, or perspectives by cultural lens while did not investigated any health-related issues.	
[89-104]	Journal article	Studies either investigated the cost-utility of testing methods, or molecular characteristics or HIV subtypes' genetic characteristics, but did not investigate MSM group's perceived health.	
[105-131]	Journal article	Studies aimed to develop a mathematical risk/prediction models or validate specific measurements/scales but did not investigate people's health.	
[132]	Thesis		
[133,134]	Journal article	Study only described the experience of coming out or homosexual identities without any other health-related issues.	
[135]	Journal article	This article only examined how homosexuality is created, regulated while no investigations of health-related variables.	
[136]	Journal article	Study merely tried to test the safety and tolerability of tenofovir.	
[137-139]	Journal article	Studies assessed and compared some estimating methods, or just estimating the number of MSM people.	
[140]	Journal article	This article was experience of a researcher and methodological reflections.	

[141]	Journal article	This study aimed to investigate factors associated with Loss-to-Follow-Up.	
[142-150]	Journal article	These articles studied masculinity preferences, visuospatial performance or association with sexism or comparisons, but no perceived health.	
[151,152]	Journal article	These studies discussed the development of sexual identity or focused on the ideology but no other investigation of health.	
[153]	Journal article	Study investigated the relationship between second to fourth digit ratio (2D:4D) and sexual orientation.	
[154-157]	Journal article	Studies only analyzed temporal and spatial distribution characteristics or spatiotemporal characteristics of MSM.	
[158]	Journal article	This paper described the dancing and chatting experience with Chinese gay men from the researchers' perspective.	
[159,160]	Journal article	Study analyzed the posts and/or opinions on Weibo/Blued only without mentioning any health issues.	
[161,162]	Journal article	Studies conducted in or before 2000.	**Non-Context**<br><br>**N=22**<br><br>Did not meet the inclusion criteria in terms of context (Chinese, in/after 2001)
[163]	Journal article	This study was carried out from November 1, 1999 to January 18, 2000	
[164-178]	Journal article	These reviews or studies included data/participants from China and also overseas, while not focused on or discussed little about situation in China.	
[179]	Journal article	The study setting was Melbourne Sexual Health Centre, not in China.	
[180-182]	Journal article	Study samples were ethic Chinese while immigrated abroad or studied abroad.	
[183-203]	Conference paper-Meeting Abstracts	These publications were meeting abstracts published in some specific journals	**Others**<br><br>**N=90**
[204-230]	Conference paper-Abstracts	These publications were poster abstracts/presentations published in some specific journals	

[231-240]	Letters-Letter to the Editor	These were reporting/summarizing letters by some experts, and generally the relevant studies have already been published.	Did not meet the inclusion criteria in terms of study types, such as meeting abstracts, poster abstracts, protocols, letters to the editor, comments, etc.
[241-245]	Registrations of Cochrane Trials	These were only the records of registration of some clinical trials, representing unfinished research.	
[246-249]	Comment	It was mainly viewpoints from experts with some suggestions, or editorial commentary.	
[250]	Protocol	It was a protocol for a Cochrane Review (Intervention).	
[251]	Magazine article	This article has no reference and written by a journalist.	
[252]	Journal article	Descriptions of sample size was inconsistent and no data of the setting.	
[253]	Journal article	This article used the data from a previous review (Chow, 2011) to conduct a secondary analysis, but there are no new findings except for implication.	
[254-259]	Journal article	These were perspectives or summaries or personal analysis or viewpoints.	
[260]	Journal article	The description of the study sample was inconsistent.	
[261]	Journal article	This paper only reviewed and analyzed articles published in '*People's Daily*', a magazine in mainland China.	
[262]	Journal article	The numbers of sample size were not insistent in the text.	
[263]	Journal article	There were no available full texts.	
[264-271]	Thesis		
[272]	Journal article	No sample size	
**Exclude: 272 references**			

Excluded references:

1. Li N, Wang Z, Sun D, et al. HIV among plasma donors and other high-risk groups in Henan, China. J Acquir Immune Defic Syndr. 2010 Feb;53 Suppl 1:S41-7.
2. Tucker JD, Yin YP, Wang B, et al. An expanding syphilis epidemic in China: epidemiology, behavioural risk and control strategies with a focus on low-tier female sex workers and men who have sex with men. Sex Transm Infect. 2011 Dec;87 Suppl 2:ii16-8.

3. Sun HY, Chang SY, Yang ZY, et al. Recent Hepatitis C Virus Infections in HIV-Infected Patients in Taiwan: Incidence and Risk Factors. Journal of Clinical Microbiology. 2012 Mar;50(3):781-787.
4. Xun HM, Kang DM, Huang T, et al. Factors Associated with Willingness to Accept Oral Fluid HIV Rapid Testing among Most-at-Risk Populations in China. Plos One. 2013 Nov;8(11).
5. Marley G, Kang D, Wilson EC, et al. Introducing rapid oral-fluid HIV testing among high risk populations in Shandong, China: feasibility and challenges. BMC Public Health. 2014;14(1):422-422.
6. Yan H, Yang H, Li J, et al. Emerging Disparity in HIV/AIDS Disease Progression and Mortality for Men Who Have Sex with Men, Jiangsu Province, China. AIDS & Behavior. 2014 Jan;18:5-10.
7. Yang L, Chen M, Ma Y, et al. The changing trends of HIV-1 prevalence and incidence from sentinel surveillance of five sub-populations in Yunnan, China, 2001-2010. BMC Public Health. 2015 Apr 12;15:376.
8. Cui Y, Guo W, Li D, et al. Estimating HIV incidence among key affected populations in China from serial cross-sectional surveys in 2010-2014. J Int AIDS Soc. 2016;19(1):20609.
9. Ong JJ, Fu H, Smith MK, et al. Expanding syphilis testing: a scoping review of syphilis testing interventions among key populations. Expert Rev Anti Infect Ther. 2018 May;16(5):423-432.
10. Soe NN, Ong JJ, Ma X, et al. Should human papillomavirus vaccination target women over age 26, heterosexual men and men who have sex with men? A targeted literature review of cost-effectiveness. Hum Vaccin Immunother. 2018;14(12):3010-3018.
11. Lee YM, Hung PS, Lin CW. Seroepidemiology and phylogenetic analysis of human herpesvirus type 8 in injection drug users and men who have sex with men in northern Taiwan. Journal of International Medical Research. 2020 Jan;48(1):300060518764747.
12. Liu C-R, Li X, Chan P-l, et al. Prevalence of hepatitis C virus infection among key populations in China: A systematic review. International Journal of Infectious Diseases. 2019 2019/03/01/;80:16-27.
13. Hung CC, Ji DD, Sun HY, et al. Increased risk for Entamoeba histolytica infection and invasive amebiasis in HIV seropositive men who have sex with men in Taiwan. PLoS Negl Trop Dis. 2008 Feb 27;2(2):e175.
14. Ko N-Y, Lee H-C, Hung C-C, et al. Effects of structural intervention on increasing condom availability and reducing risky sexual behaviours in gay bathhouse attendees. AIDS Care. 2009 Dec;21(12):1499-1507.
15. Ko N-Y, Lee H-C, Hung C-C, et al. Trends of HIV and sexually transmitted infections, estimated HIV incidence, and risky sexual behaviors among gay bathhouse attendees in Taiwan: 2004–2008. AIDS and Behavior. 2011 Feb;15(2):292-297.
16. Ko N-Y, Lee H-C, Chang J-L, et al. Condom availability in Taiwanese gay bathhouses: The right things in the wrong places. AIDS Education and Prevention. 2008 Aug;20(4):338-346.
17. Lau H, Stotzer RL. Employment discrimination based on sexual orientation: A Hong Kong study. Employee Responsibilities and Rights Journal. 2011 Mar;23(1):17-35.
18. Kwok D, Winter S, Yuen M. Heterosexism in school: the counselling experience of Chinese tongzhi students in Hong Kong. British Journal of Guidance & Counselling. 2012;40(5):561-575.

19. Chen IC. Loneliness of homosexual male students: Parental bonding attitude as a moderating factor. The Spanish Journal of Psychology. 2013 Aug 13;16:9.
20. Bogaert AF, Liu J. Physical Size and Sexual Orientation: Analysis of the Chinese Health and Family Life Survey. Archives of Sexual Behavior. 2013 Nov;42(8):1555-1559.
21. Liu M. Two Gay Men Seeking Two Lesbians: An Analysis of Xinghun (Formality Marriage) Ads on China's Tianya.cn. Sexuality & Culture. 2013 September;17(3):494-511.
22. Tseng F-C, Ko N-Y, Lee H-C, et al. HIV risk profiles differed by gender and experience of men who had sex with men among attendees of anonymous voluntary counseling and testing in Taiwan. AIDS Care. 2013;25(9):1092-1101.
23. Dong C, Huang ZJ, Martin MC, et al. The impact of social factors on human immunodeficiency virus and hepatitis C virus co-infection in a minority region of Si-chuan, the People's Republic of China: A population-based survey and testing study [Article]. PLoS ONE. 2014;9(7):e101241.
24. Fan EL. HIV testing as prevention among MSM in China: The business of scaling-up. Global Public Health: An International Journal for Research, Policy and Practice. 2014 Feb;9(1-2):85-97.
25. Tucker JD, Muessig KE, Cui R, et al. Organizational characteristics of HIV/syphilis testing services for men who have sex with men in South China: a social entrepreneurship analysis and implications for creating sustainable service models. BMC Infectious Diseases. 2014;14(1):601-601.
26. Yan H, Zhang M, Zhao J, et al. The increased effectiveness of HIV preventive intervention among men who have sex with men and of follow-up care for people living with HIV after 'task-shifting' to community-based organizations: a 'cash on service delivery' model in China. PLoS One. 2014;9(7):e103146.
27. Cheng W, Cai Y, Tang W, et al. Providing HIV-related services in China for men who have sex with men. Bull World Health Organ. 2016 Mar 1;94(3):222-7.
28. Qian J. Narrating the trope of abnormality: The making of closeted experiences in gay public cruising. Geoforum. 2014 March;52:157-166.
29. Tu J-W, Lee T-T. The effects of media usage and interpersonal contacts on the stereotyping of lesbians and gay men in China. Journal of Homosexuality. 2014 Jul;61(7):980-1002.
30. Lee SL. What factors influence comfort with same-sex sexuality among undergraduate social work students? Evidence from Taiwan. Asia Pacific Journal of Social Work and Development. 2018;28(2):97-110.
31. Cui JM, Pei M, Su YJ. Gender-related friend preferences of youths with different sexual orientations: the effects of gender role and sexual attraction. Psychology & Sexuality. 2020:1-18.
32. Lin ZY, Yu WH, Su KH. Comparing same- and different-sex relationship dynamics: Experiences of young adults in Taiwan. Demographic Research. 2019 Mar;40:431-462.

33. Chen L, Yang JZ, Zhang RJ, et al. Rates and risk factors associated with the progression of HIV to AIDS among HIV patients from Zhejiang, China between 2008 and 2012. Aids Research and Therapy. 2015 Sep;12.
34. Su S, Chen X, Mao L, et al. Superior Effects of Antiretroviral Treatment among Men Who have Sex with Men Compared to Other HIV At-Risk Populations in a Large Cohort Study in Hunan, China. Int J Environ Res Public Health. 2016 Mar 8;13(3).
35. Yu NX, Chen L, Ye Z, et al. Impacts of making sense of adversity on depression, posttraumatic stress disorder, and posttraumatic growth among a sample of mainly newly diagnosed HIV-positive Chinese young homosexual men: the mediating role of resilience. AIDS Care. 2017 Jan;29(1):79-85.
36. Chen GJ, Lin KY, Sun HY, et al. Incidence of acute hepatitis A among HIV-positive patients during an outbreak among MSM in Taiwan: Impact of HAV vaccination. Liver Int. 2018 Apr;38(4):594-601.
37. Cheng CY, Wu HH, Zou H, et al. Epidemiological characteristics and associated factors of acute hepatitis A outbreak among HIV-coinfected men who have sex with men in Taiwan, June 2015-December 2016. J Viral Hepat. 2018 Oct;25(10):1208-1215.
38. Aunon FM, Simoni JM, Yang JP, et al. Depression and anxiety among HIV-positive men who have sex with men and men who have sex with women in China. AIDS Care. 2020 Mar;32(3):362-369.
39. Jia D, Zhao J, Liu Y, et al. Two-year cross-sectional studies reveal that single, young MSMs in Shenzhen, China are at high risk for HIV infection. Virol J. 2019 Jun 22;16(1):83.
40. Wu HH, Shen YT, Chiou CS, et al. Shigellosis outbreak among MSM living with HIV: a case-control study in Taiwan, 2015-2016. Sex Transm Infect. 2019 Feb;95(1):67-70.
41. Chen X, Yu B, Zhou D, et al. A Comparison of the Number of Men Who Have Sex with Men among Rural-To-Urban Migrants with Non-Migrant Rural and Urban Residents in Wuhan, China: A GIS/GPS-Assisted Random Sample Survey Study. PLoS One. 2015;10(8):e0134712.
42. Ke L, Ho J, Feng JZ, et al. Prevalence, Awareness, Treatment and Control of Hypertension in Macau: Results From a Cross-Sectional Epidemiological Study in Macau, China. American Journal of Hypertension. 2015 Feb;28(2):159-165.
43. Lu J, Xu J, Reilly KH, et al. The proportion and trend of human immunodeficiency virus infections associated with men who have sex with men from Chinese voluntary blood donors: a systematic review and meta-analysis. Transfusion. 2015 Mar;55(3):576-585.
44. Luo Y, Zhu C, Chen S, et al. Risk factors for HIV and syphilis infection among male sex workers who have sex with men: a cross-sectional study in Hangzhou, China, 2011. BMJ Open. 2015 Apr 28;5(4):e006791.
45. Tian T, Wang D, Papamichael C, et al. HPV vaccination acceptability among men who have sex with men in Urumqi, China. Hum Vaccin Immunother. 2019;15(4):1005-1012.
46. Guo C, Pang LH, Zhang L, et al. Disparities of sexual orientations by sex and urban or rural residence among youth in China. Sexual Health. 2016 Aug 19;13(5):497-499.
47. Kwok DK. School experience of Chinese sexual minority students in Hong Kong. Journal of Lgbt Youth. 2016;13(4):378-396.

48. Li X, Zhang B, Li Y, et al. Mental health and suicidal ideation among Chinese women who have sex with men who have sex with men (MSM). Women Health. 2016 Nov-Dec;56(8):940-56.
49. Li X, Zhang B, Wang J, et al. Sexual health status of women who have regular sexual relations with men who have sex with men in mainland China. BMC Public Health. 2017 Feb 6;17(1):168.
50. Cao WN, Wong HM, Yang XY, et al. The impact of serostatus disclosure on spousal relationships between wives and HIV-positive husbands who have sex with men in China. Women & Health. 2020 Jul;60(6):664-675.
51. Brainer A. Materializing "Family Pressure" among Taiwanese Queer Women. Feminist Formations. 2017 Winter;29(3):1-24.
52. Chan KK. Family and homosexuality in Chinese culture: Rights claims by non-heterosexuals in Hong Kong. Sexuality & Culture: An Interdisciplinary Quarterly. 2017 Sep;21(3):845-859.
53. Lau H, Yeung G, Stotzer RL, et al. Assessing the Tongzhi Label: Self-Identification and Public Opinion. J Homosex. 2017;64(4):509-522.
54. Lau JTF, Wang Z, Kim Y, et al. Low sustainability, poor governance, and other challenges encountered by grassroots non-governmental organizations targeting HIV prevention for men who have sex with men in China—A nation-wide study. AIDS Care. 2017 Dec;29(12):1480-1490.
55. Wong JY, Choi EP, Lo HH, et al. Dating violence, quality of life and mental health in sexual minority populations: a path analysis. Qual Life Res. 2017 Apr;26(4):959-968.
56. Wu Y, Mou Y, Wang Y, et al. Exploring the de-stigmatizing effect of social media on homosexuality in China: an interpersonal-mediated contact versus parasocial-mediated contact perspective [Article]. Asian Journal of Communication. 2017;28(1):20-37.
57. Xin HN, Li HJ, Li Z, et al. Genital HPV infection among heterosexual and homosexual male attendees of sexually transmitted diseases clinic in Beijing, China. Epidemiol Infect. 2017 Oct;145(13):2838-2847.
58. Cao BL, Zhao PP, Bien-Gund C, et al. The Web-Based Physician is Ready to See You: A Nationwide Cross-Sectional Survey of Physicians Using a Mobile Medical App to Evaluate Patients With Sexually Transmitted Diseases in China. Jmir Mhealth and Uhealth. 2018 Oct;6(10):e10531.
59. Xia D, Feng X, He X, et al. Feasibility of an Internet-based HIV testing service: Anonymous urine collection from men who have sex with men. AIDS Care. 2018 Oct;30(10):1228-1230.
60. Li C, Xiong Y, Sit HF, et al. A men who have sex with men-friendly doctor finder hackathon in guangzhou, China: Development of a mobile health intervention to enhance health care utilization [Article]. JMIR mHealth and uHealth. 2020 Feb 27;8(2):e16030.
61. Lin IH, Ko NY, Huang YT, et al. Effect of Same-Sex Marriage Referendums on the Suicidal Ideation Rate among Nonheterosexual People in Taiwan. Int J Environ Res Public Health. 2019 Sep 17;16(18).
62. Yang G, Long J, Luo D, et al. The Characteristics and Quality of Mobile Phone Apps Targeted at Men Who Have Sex With Men in China: A Window of Opportunity for Health Information Dissemination? JMIR Mhealth Uhealth. 2019 Mar 27;7(3):e12573.

63. Liu C, Lu X. Network Evolution of a Large Online MSM Dating Community: 2005-2018. Int J Environ Res Public Health. 2019 Nov 6;16(22).
64. Kam LYL. Noras on the road: Family and marriage of lesbian women in Shanghai. Journal of Lesbian Studies. 2006 2006;10(3-4):87-103.
65. Jones RH. A walk in the park: Frames and positions in AIDS prevention outreach among gay men in China. Journal of Sociolinguistics. 2002 Nov 2002;6(4):575-588.
66. Wah-Shan C. Homosexuality and the cultural politics of tongzhi in Chinese societies. J Homosex. 2001;40(3-4):27-46.
67. Kong TSK. The Seduction of the Golden Boy: The Body Politics of Hong Kong Gay Men. Body & Society. 2002 March 2002;8(1):29-48.
68. He Q, Wang Y, Li Y, et al. Accessing men who have sex with men through long-chain referral recruitment, Guangzhou, China. AIDS & Behavior. 2008;12:S93-6.
69. Yan H, Yang H, Zhao J, et al. Long-chain peer referral of men who have sex with men: A novel approach to establish and maintain a cohort to measure HIV incidence, Nanjing, China. JAIDS Journal of Acquired Immune Deficiency Syndromes. 2012 Feb 01;59(2):177-184.
70. Zhao J, Cai WD, Gan YX, et al. A comparison of HIV infection and related risk factors between money boys and noncommercial men who have sex with men in Shenzhen, China. Sex Transm Dis. 2012 Dec;39(12):942-8.
71. Tang W, Yang H, Mahapatra T, et al. Feasibility of recruiting a diverse sample of men who have sex with men: observation from Nanjing, China. PLoS One. 2013;8(11):e77645.
72. Wei W. Going public: The production and transformation of queer spaces in postsocialist Chengdu, China [[Ph.D.]]. Chicago Loyola University Chicago; 2007.
73. Zhang D, Li C, Meng S, et al. Attrition of MSM with HIV/AIDS along the continuum of care from screening to CD4 testing in China. AIDS Care. 2014 Sep 02;26(9):1118-1121.
74. Zhang D, Qi J, Fu X, et al. Case finding advantage of HIV rapid tests in community settings: men who have sex with men in 12 programme areas in China, 2011. International Journal of STD & AIDS. 2015 May;26(6):402-413.
75. He L, Yang J, Ma Q, et al. Reduction in HIV community viral loads following the implementation of a "Treatment as Prevention" strategy over 2 years at a population-level among men who have sex with men in Hangzhou, China. BMC Infectious Diseases. 2018;18:1-N.PAG.
76. Hu SH, Wei N, Wang QD, et al. Patterns of brain activation during visually evoked sexual arousal differ between homosexual and heterosexual men. AJNR Am J Neuroradiol. 2008 Nov;29(10):1890-6.
77. Hu SH, Wang QD, Xu Y, et al. Haemodynamic brain response to visual sexual stimuli is different between homosexual and heterosexual men. J Int Med Res. 2011;39(1):199-211.
78. Zhang M, Hu S, Xu L, et al. Neural circuits of disgust induced by sexual stimuli in homosexual and heterosexual men: an fMRI study. Eur J Radiol. 2011 Nov;80(2):418-25.

79. Hu S, Xu D, Peterson BS, et al. Association of cerebral networks in resting state with sexual preference of homosexual men: a study of regional homogeneity and functional connectivity. PLoS One. 2013;8(3):e59426.
80. Hu S, Xu D, Peterson BS, et al. Differing default mode network activities in men with homosexual or heterosexual preferences. Journal of Sexual Medicine. 2014 Oct;11(10):2474-2484.
81. Hou J, Sui L, Jiang X, et al. Facial attractiveness of Chinese college students with different sexual orientation and sex roles. Frontiers in Human Neuroscience. 2019 Apr 30;13:12.
82. Engebretsen EL. Intimate Practices, Conjugal Ideals: Affective Ties and Relationship Strategies Among Lala (Lesbian) Women in Contemporary Beijing. Sexuality Research and Social Policy: Journal of NSRC. 2009 September;6(3):3-14.
83. Worth H, Jing J, McMillan K, et al. “Under the same quilt”: The paradoxes of sex between men in the cultural revolution. Journal of Homosexuality. 2017 Jan;64(1):61-74.
84. Boya H, Yang VF, Goldsen Karen F. LGBT Older Adults at a Crossroads in Mainland China: The Intersections of Stigma, Cultural Values, and Structural Changes Within a Shifting Context. The International Journal of Aging & Human Development. 2019 Jun;88(4):440-456.
85. Fung CKM. TBG and Po: Discourses on authentic desire in 2010s lesbian subcultures in Hong Kong, China, and Taiwan. Journal of Lesbian Studies. 2019 Nov 29:1-18.
86. Jhang HC. Examining Cultural Discourses in Taiwanese Gender and Sexual Minority/Tongzhi Family-of-Origin Relationships. Cross-Currents-East Asian History and Culture Review. 2019 Dec(33):115-142.
87. Worth H, Jing J, McMillan K, et al. ‘There was no mercy at all’: Hooliganism, homosexuality and the opening-up of China. International Sociology. 2019 Jan;34(1):38-57.
88. Liu TT. The empowerment of rural migrant lalas: Contending queerness and heteronormativity in China. China Information. 2019 Jul;33(2):165-184.
89. Guo H, Wei JF, Yang H, et al. Rapidly increasing prevalence of HIV and syphilis and HIV-1 subtype characterization among men who have sex with men in Jiangsu, China. Sex Transm Dis. 2009 Feb;36(2):120-5.
90. Han X, Xu J, Chu Z, et al. Screening acute HIV infections among Chinese men who have sex with men from voluntary counseling & testing centers. PLoS One. 2011;6(12):e28792.
91. Li L, Sun G, Li T, et al. Multiple introductions of HIV into men who have sex with men were found in Zhengzhou City, China. AIDS Res Hum Retroviruses. 2012 Sep;28(9):1147-51.
92. Liu Z, Li L, Chen Z, et al. Prevalence of GB virus type C viraemia in MSM with or without HIV-1 infection in Beijing, China. Epidemiology & Infection. 2012;140(12):2199-2209.
93. Chen M, Ma Y, Su Y, et al. HIV-1 genetic characteristics and transmitted drug resistance among men who have sex with men in Kunming, China. PLoS One. 2014;9(1):e87033.
94. Dai L, Li N, Wei F, et al. Transmitted antiretroviral drug resistance in the men who have sex with men HIV patient cohort, Beijing, China, 2008-2011. Viral Immunol. 2014 Oct;27(8):392-7.

95. Shen YL, Su b, Wu JJ, et al. The prevalence of transmitted HIV drug resistance among MSM in Anhui province, China. Aids Research and Therapy. 2014 Jul;11.
96. Chan DP, Lin AW, Wong KH, et al. Diverse origins of hepatitis C virus in HIV co-infected men who have sex with men in Hong Kong. Virol J. 2015 Aug 8;12:120.
97. Hou LJ, Wang HW, Duan SP, et al. The prevalence and determinants of drug-resistance-associated mutations in the HIV-1-infected MSM population of Henan Province in China. Arch Virol. 2015 Aug;160(8):2051-61.
98. Liang J, Liu L, Cheung M, et al. Community-Based HIV-1 Early Diagnosis and Risk Behavior Analysis of Men Having Sex with Men in Hong Kong. PLoS One. 2015;10(4):e0125715.
99. Yan H, Ding Y, Wong FY, et al. Epidemiological and molecular characteristics of HIV infection among money boys and general men who have sex with men in Shanghai, China. Infect Genet Evol. 2015 Apr;31:135-41.
100. Chiou CS, Izumiya H, Kawamura M, et al. The worldwide spread of ciprofloxacin-resistant Shigella sonnei among HIV-infected men who have sex with men, Taiwan. Clin Microbiol Infect. 2016 Apr;22(4):383.e11-383.e16.
101. Qin X, Zheng H, Xue Y, et al. Prevalence of Chlamydia trachomatis Genotypes in Men Who Have Sex with Men and Men Who Have Sex with Women Using Multilocus VNTR Analysis-ompA Typing in Guangzhou, China. PLoS One. 2016;11(7):e0159658.
102. Lou J, Hu P, Qian HZ, et al. Expanded antiretroviral treatment, sexual networks, and condom use: Treatment as prevention unlikely to succeed without partner reduction among men who have sex with men in China. PLoS One. 2017;12(4):e0171295.
103. Luan H, Han X, Yu X, et al. Dual Infection Contributes to Rapid Disease Progression in Men Who Have Sex With Men in China. J Acquir Immune Defic Syndr. 2017 Aug 1;75(4):480-487.
104. Zhang J, Guo Z, Pan X, et al. Highlighting the crucial role of Hangzhou in HIV-1 transmission among men who have sex with men in Zhejiang, China. Sci Rep. 2017 Oct 24;7(1):13892.
105. Lou J, Wu J, Chen L, et al. A sex-role-preference model for HIV transmission among men who have sex with men in China. BMC Public Health. 2009 Nov 18;9 Suppl 1:S10.
106. Li J, Liu H, Liu H, et al. Psychometric assessment of HIV/STI sexual risk scale among MSM: a Rasch model approach. BMC Public Health. 2011;11(1):763-763.
107. Ma N, Zheng M, Liu M, et al. Impact of condom use and standardized sexually transmitted disease treatment on HIV prevention among men who have sex with men in hunan province: using the Asian epidemic model. AIDS Res Hum Retroviruses. 2012 Oct;28(10):1273-9.
108. Liu J, Qu B, Hu B, et al. The quality of life of men who have sex with men in China: Reliability and validity testing of the SF-36 questionnaire. PLoS ONE. 2013 Dec 19;8(12):7.
109. Sun X, Xiao Y, Peng Z, et al. Modelling HIV/AIDS epidemic among men who have sex with men in China. Biomed Res Int. 2013;2013:413260.
110. Zhang J, Jin Z, Chen Y. Analysis of sexually transmitted disease spreading in heterosexual and homosexual populations. Math Biosci. 2013 Apr;242(2):143-52.

111. Chen XY, Du QQ, Jin ZD, et al. The Randomized Response Technique Application in the Survey of Homosexual Commercial Sex among Men in Beijing. Iranian Journal of Public Health. 2014 Apr;43(4):416-422.
112. Lou J, Blevins M, Ruan Y, et al. Modeling the impact on HIV incidence of combination prevention strategies among men who have sex with men in Beijing, China. PLoS One. 2014;9(3):e90985.
113. Huang CY. An agent-based epidemic simulation of social behaviors affecting HIV transmission among Taiwanese homosexuals. Comput Math Methods Med. 2015;2015:867264.
114. Luo S, Han L, Lu H, et al. Evaluating the Impact of Test-and-Treat on the HIV Epidemic among MSM in China Using a Mathematical Model. PLoS One. 2015;10(6):e0126893.
115. Zhang C, Blashill AJ, Wester SR, et al. Factor structure of the Gender Role Conflict Scale-Short Form in Chinese heterosexual and gay samples. Psychology of Men & Masculinity. 2015 Apr;16(2):229-233.
116. Shen ZF, Li YB, Yao W. A scale-free network model for HIV transmission among men who have sex with men in China. Mathematical Methods in the Applied Sciences. 2016 Dec;39(18):5131-5139.
117. Sun X, Xiao Y, Tang S, et al. Early HAART Initiation May Not Reduce Actual Reproduction Number and Prevalence of MSM Infection: Perspectives from Coupled within- and between-Host Modelling Studies of Chinese MSM Populations. PLoS One. 2016;11(3):e0150513.
118. Zhang D, Lu H, Zhuang M, et al. Enhancing HIV Testing and Treatment among Men Who Have Sex with Men in China: A Pilot Model with Two-Rapid Tests, Single Blood Draw Session, and Intensified Case Management in Six Cities in 2013. PLoS One. 2016;11(12):e0166812.
119. Zhang L, Regan DG, Chow EPF, et al. Neisseria gonorrhoeae Transmission Among Men Who Have Sex With Men: An Anatomical Site-Specific Mathematical Model Evaluating the Potential Preventive Impact of Mouthwash. Sex Transm Dis. 2017 Oct;44(10):586-592.
120. Li J, Peng L, Gilmour S, et al. A mathematical model of biomedical interventions for HIV prevention among men who have sex with men in China. BMC Infect Dis. 2018 Nov 28;18(1):600.
121. Lou J, Cheng J, Li Y, et al. Comparison of different strategies for controlling HIV/AIDS spreading in MSM [Article]. Infectious Disease Modelling. 2018;3:293-300.
122. Ren Z, Hood RW, Jr. Internalized homophobia scale for gay Chinese men: Conceptualization, factor structure, reliability, and associations with hypothesized correlates. American Journal of Men's Health. 2018 Sep;12(5):1297-1306.
123. Sun X, Xiao Y, Peng Z, et al. Frequent implementation of interventions may increase HIV infections among MSM in China. Sci Rep. 2018 Jan 11;8(1):451.
124. Wong NS, Kwan TH, Tsang OTY, et al. Pre-exposure prophylaxis (PrEP) for MSM in low HIV incidence places: should high risk individuals be targeted? Sci Rep. 2018 Aug 3;8(1):11641.
125. Wu X, Lau JTF, Mak WWS, et al. How newly diagnosed HIV-positive men who have sex with men look at HIV/AIDS - validation of the Chinese version of the revised illness perception questionnaire. BMC Infect Dis. 2018 Jan 2;18(1):2.
126. Xun Z, Peng P, Huamin S, et al. Scaling Up Human Immunodeficiency Virus Screening and Antiretroviral Therapy Among Men Who Have Sex With Men to Achieve the 90-90-90 Targets in China. Sexually Transmitted Diseases. 2018 May;45(5):343-349.

127. Cao W, Mo PK, Lau JT. Validation of the Outcome Expectancy Scale for HIV Serostatus Disclosure to Female Sex Partners Among Men Who Have Sex with Men and Women Living with HIV in China. J Sex Marital Ther. 2019;45(7):604-617.
128. Kumi SM, Stein G, Cheng W, et al. Identifying high risk subgroups of MSM: A latent class analysis using two samples [Article]. BMC Infectious Diseases. 2019 Mar 5;19(1):213.
129. Zhang C, Webb GF, Lou J, et al. Predicting the long-term impact of voluntary medical male circumcision on HIV incidence among men who have sex with men in Beijing, China. AIDS Care. 2020 Mar;32(3):343-353.
130. Zhang C, Penson DF, Qian HZ, et al. Modeling economic and epidemiological impact of voluntary medical male circumcision among men who have sex with men in Beijing, China. Int J STD AIDS. 2019 Jun;30(7):630-638.
131. Yun K, Xu J, Leuba S, et al. Development and Validation of a Personalized Social Media Platform-Based HIV Incidence Risk Assessment Tool for Men Who Have Sex With Men in China. Journal of Medical Internet Research. 2019 Jun 18;21(6):N.PAG-N.PAG.
132. Poon CM. A two-mode network approach in assessing and modelling HIV transmission patterns among men who have sex with men in Hong Kong [Ph.D.]: The Chinese University of Hong Kong (Hong Kong); 2017.
133. Wang FT, Bih HD, Brennan DJ. Have they really come out: gay men and their parents in Taiwan. Cult Health Sex. 2009 Apr;11(3):285-96.
134. Li H, Holroyd E, Lau JTF. Negotiating homosexual identities: The experiences of men who have sex with men in Guangzhou. Culture, Health & Sexuality. 2010 May;12(4):401-414.
135. Wong D, Leung PK. Modernization of power in legal and medical discourses: The birth of the (male) homosexual in Hong Kong and its aftermath. Journal of Homosexuality. 2012 Nov;59(10):1403-1423.
136. Vermund SH. Safety and Tolerability of Tenofovir for Preexposure Prophylaxis Among Men Who Have Sex With Men. Jaids-Journal of Acquired Immune Deficiency Syndromes. 2013 Sep;64(1):3-6.
137. Jing LW, Qu CY, Yu HM, et al. Estimating the Sizes of Populations at High Risk for HIV: A Comparison Study. Plos One. 2014 Apr;9(4).
138. Shaochun Y, Ying FU, Yunhua Z, et al. The Cardinal Number Estimation of Men Who Have Sex with Men, in China. Iranian Journal of Public Health. 2015;44(3):420-421.
139. Wang J, Yang Y, Zhao W, et al. Application of Network Scale Up Method in the Estimation of Population Size for Men Who Have Sex with Men in Shanghai, China. PLoS One. 2015;10(11):e0143118.
140. Suen YT. Methodological reflections on researching lesbian, gay, bisexual and transgender university students in Hong Kong: To what extent are they vulnerable interview subjects? Higher Education Research & Development. 2015 Jul;34(4):722-734.
141. Tang W, Huan X, Zhang Y, et al. Factors associated with loss-to-follow-up during behavioral interventions and HIV testing cohort among men who have sex with men in Nanjing, China. PLoS One. 2015;10(1):e115691.
142. Zheng L, Zheng Y. Young gay men’s sexism predict their male facial masculinity preference in China. Personality and Individual Differences. 2015 Apr;76:183-186.

143. Zheng L, Zheng Y. Preferences for Masculinity Across Faces, Bodies, and Personality Traits in Homosexual and Bisexual Chinese Men: Relationship to Sexual Self-Labels and Attitudes Toward Masculinity. Archives of Sexual Behavior. 2016 Apr;45(3):725-733.
144. Zhang J, Zheng LJ, Zheng Y. Consistency in preferences for masculinity in faces, bodies, voices, and personality characteristics among homosexual men in China. Personality and Individual Differences. 2018 Nov;134:137-142.
145. Zheng L, Wen G, Zheng Y. Butch–femme identity and visuospatial performance among lesbian and bisexual women in China. Archives of Sexual Behavior. 2018 May;47(4):1015-1024.
146. Zheng LJ. Preference for Male Facial Masculinity as a Function of Mental Rotation Ability in Gay and Bisexual Men, but Not in Heterosexual Men and Women in China. Frontiers in Psychology. 2019 Oct;10:2419.
147. Zheng LJ. The Dyadic Effects of Top/Bottom Sexual Self-Labels and Partner Sexual Role Requirements on Facial Masculinity Preferences Among Gay and Bisexual Men in China. Journal of Sex Research. 2019 Oct 30:1-7.
148. Zheng L. Facial masculinity preferences according to relationship status and sociosexual orientation in gay and bisexual men in China. Personality and Individual Differences. 2019 Feb 01;138:243-246.
149. Zheng L, Hart TA, Zheng Y. Attraction to male facial masculinity in gay men in china: relationship to intercourse preference positions and sociosexual behavior. Archives of Sexual Behavior. 2013 Oct;42(7):1223-1232.
150. Leung KK, Wong HT, Naftalin CM, et al. A new perspective on sexual mixing among men who have sex with men by body image. PLoS One. 2014;9(11):e113791.
151. Ho PSY, Hu YQ. Pray the gay away: identity conflict between Christianity and sexuality in Hong Kong sexual minorities. Gender Place and Culture. 2016 Dec;23(12):1725-1737.
152. Zheng L, Zheng Y. Gender Nonconformity and Butch-Femme Identity Among Lesbians in China. J Sex Res. 2016;53(2):186-93.
153. Li C, Jia M, Ma Y, et al. The relationship between digit ratio and sexual orientation in a Chinese Yunnan Han population. Personality and Individual Differences. 2016 Oct;101:26-29.
154. Qin Q, Guo W, Tang W, et al. Spatial Analysis of the Human Immunodeficiency Virus Epidemic among Men Who Have Sex with Men in China, 2006-2015. Clin Infect Dis. 2017 Apr 1;64(7):956-963.
155. Fu R, Zhao J, Wu D, et al. A spatiotemporal meta-analysis of HIV/syphilis epidemic among men who have sex with men living in mainland China. BMC Infectious Diseases. 2018;18(1):N.PAG-N.PAG.
156. Huang DC, Wang JF, Yang TF. Mapping the Spatial-Temporal Distribution and Migration Patterns of Men Who Have Sex with Men in Mainland China: A Web-Based Study. International Journal of Environmental Research and Public Health. 2020 Mar;17(5).
157. Hu MG, Xu CD, Wang JF. Spatiotemporal Analysis of Men Who Have Sex With Men in Mainland China: Social App Capture-Recapture Method. Jmir Mhealth and Uhealth. 2020 Jan;8(1):e14800.
158. Miège P. May I have the next dance? Chinese gay men exploring selves and practices through the tradition of dance in public spaces. Culture, Health & Sexuality. 2018 Aug;20(8):902-914.

159. Chen L, Guo Y, Shi JY. Social Support Seeking on Social Media Among Chinese Gay Men Living with HIV/AIDS: The Role of Perceived Threat. Telemedicine and E-Health. 2019 Jul;25(7):655-659.
160. Huang G, Cai M, Lu X. Inferring Opinions and Behavioral Characteristics of Gay Men with Large Scale Multilingual Text from Blued. Int J Environ Res Public Health. 2019 Sep 26;16(19).
161. Choi K, Diehl E, Yaqi G, et al. High HIV risk but inadequate prevention services for men in China who have sex with men: an ethnographic study. AIDS & Behavior. 2002;6(3):255-266.
162. Wei C, Guadamuz TE, Stall R, et al. STD prevalence, risky sexual behaviors, and sex with women in a national sample of Chinese men who have sex with men. American Journal of Public Health. 2009;99(11):1978-1981.
163. Lau JT, Siah PC, Tsui HY. A study of the STD/AIDS related attitudes and behaviors of men who have sex with men in Hong Kong. Arch Sex Behav. 2002 Aug;31(4):367-73.
164. de Lind van Wijngaarden JW, Brown T, Girault P, et al. The epidemiology of human immunodeficiency virus infection, sexually transmitted infections, and associated risk behaviors among men who have sex with men in the Mekong Subregion and China: implications for policy and programming. Sex Transm Dis. 2009 May;36(5):319-24.
165. Wei C, Herrick A, Raymond HF, et al. Social marketing interventions to increase HIV/STI testing uptake among men who have sex with men and male-to-female transgender women. Cochrane Database Syst Rev. 2011 Sep 7(9):Cd009337.
166. Heywood W, Smith AM. Anal sex practices in heterosexual and male homosexual populations: a review of population-based data [Article]. Sex Health. 2012 Dec;9(6):517-26.
167. Wirtz AL, Kirey A, Peryskina A, et al. Uncovering the epidemic of HIV among men who have sex with men in Central Asia. Drug Alcohol Depend. 2013 Nov;132 Suppl 1:S17-24.
168. Grosso A, Ryan O, Tram KH, et al. Financing the response to HIV among gay men and other men who have sex with men: Case studies from eight diverse countries. Global Public Health: An International Journal for Research, Policy and Practice. 2015 Nov;10(10):1172-1184.
169. Meng X, Zou H, Fan S, et al. Relative Risk for HIV Infection Among Men Who Have Sex with Men Engaging in Different Roles in Anal Sex: A Systematic Review and Meta-analysis on Global Data. AIDS and Behavior. 2015 May;19(5):882-889.
170. Xu Y, Zheng Y. Prevalence of Childhood Sexual Abuse among Lesbian, Gay, and Bisexual People: A Meta-Analysis. J Child Sex Abus. 2015;24(3):315-31.
171. Beyrer C, Baral SD, Collins C, et al. The global response to HIV in men who have sex with men. The Lancet. 2016 Jul 09;388(10040):198-206.
172. Chan LS. How Sociocultural context matters in self-presentation: A comparison of U.S. and Chinese Profiles on Jack'd, a mobile dating app for men who have sex with men [Article]. International Journal of Communication. 2016;10:6040-6059.
173. Lin C. The Dominant Value System of Chinese Gay Males in Family, Couple, and Community Relationships: A Qualitative Study. Journal of Family Psychotherapy. 2016 Oct/Dec;27(4):288-301.

174. Cheng FK. Resilience of Buddhist sexual minorities related to sexual orientation and gender identity. International Journal of Happiness and Development. 2017;3(4):368-383.
175. Tang Denise T-S, Khor D, Yi-Chien C. Legal recognition of same-sex partnerships: A comparative study of Hong Kong, Taiwan and Japan. The Sociological Review. 2020 Jan;68(1):192-208.
176. Lin C, Moore DD, Nylund D, et al. Clinical Issues among Chinese Gay Men in Counseling. Journal of LGBT Issues in Counseling. 2020;14(1):18-37.
177. Ye S, Yin L, Amico R, et al. Efficacy of peer-led interventions to reduce unprotected anal intercourse among men who have sex with men: a meta-analysis. PLoS One. 2014;9(3):e90788.
178. Yang Z, Zhang S, Dong Z, et al. Prevalence of unprotected anal intercourse in men who have sex with men recruited online versus offline: a meta-analysis. BMC Public Health. 2014 May 26;14:508.
179. Zou HC, Fairley CK, Guy R, et al. Automated, Computer Generated Reminders and Increased Detection of Gonorrhoea, Chlamydia and Syphilis in Men Who Have Sex with Men. Plos One. 2013 Apr;8(4).
180. Lin CC. Reinforcing Behaviors of Chinese Gay Male Users on Facebook. Psychology of Popular Media Culture. 2018 Jul;7(3):289-296.
181. Kam LYL. Coming out and going abroad: The chuguo mobility of queer women in china. Journal of Lesbian Studies. 2019 Jun 15;24(2):126-139.
182. Ren ZJ, Hood R, Yang CS, et al. THE EFFECTS OF SELF-CONSTRUAL AND THE LIVING ENVIRONMENT ON INTERNALIZED HOMOPHOBIAAMONG CHINESE GAY MEN. Journal of Mens Health. 2019;15(3):E25-E34.
183. Chen Y, Yang H, Zhang T, et al. Limited introductions and genetic variation of HIV-1 in a MSM cohort in China. Aids Research and Human Retroviruses; Oct2010. p. A144-A144.
184. Chen Z, Chu X, Yu F, et al. Using a social networking "app" to establish an open cohort for assessing HIV incidence among men who have sex with men in Beijing, China. Journal of the International Aids Society; Jul2018. p. 166-167.
185. Cheng RX, Mak WWS. Through a cultural lens: Filial piety and overall well-being of Chinese HIV-positive gay men. International Journal of Psychology; Jul2016. p. 674-674.
186. Fu R, Wu YM, Xie L, et al. Understanding Chinese Gay Men's Sexual Health Needs to Prepare for Implementation of PrEP in China. Aids Research and Human Retroviruses; Oct2018. p. 146-146.
187. Ho SY, Lin PH, Su LH, et al. HCV incidence among HIV-positive MSM in Taiwan during an outbreak of acute HCV. International Journal of Antimicrobial Agents; Nov2017. p. S277-S277.
188. Hou JH, Wu YM, Xie L, et al. Post-exposure Prophylaxis: An Underutilized Biomedical HIV Prevention Method Among MSM in China. Aids Research and Human Retroviruses; Oct2018. p. 148-148.
189. Hsu CH, Huang JH. COMPARING HIV ORAL RAPID SELF-TESTING INTENTION IN TAIWAN YMSM USING THEORY OF PLANNED BEHAVIOR, BY PERCEIVED LGBT FRIENDLINESS. Annals of Behavioral Medicine; Mar2019. p. S318-S318.

190. Lau JTF, Wang Z, Li JH. BEHAVIORAL MEDICINE AND THE HIV EPIDEMIC AMONG MEN WHO HAVE SEX WITH MEN IN CHINA: ROLES, CHALLENGES AND OPPORTUNITIES. International Journal of Behavioral Medicine; Nov2016. p. S210-S211.
191. Li JH, Wang KR, Peng LP, et al. ELIMINATION OF HIV INFECTION AMONG MEN WHO HAVE SEX WITH MEN IN GUANGDONG, CHINA USING COMBINED HIV BIOMEDICAL INTERVENTIONS: A MATHEMATICAL MODELLING STUDY. International Journal of Behavioral Medicine; Oct2018. p. S173-S173.
192. Lin KY, Wu PY, Liu WC, et al. Effectiveness of hepatitis A vaccination in HIV-positive men who have sex with men during an ongoing hepatitis A outbreak in Taiwan. Journal of the International Aids Society; Oct2016.
193. Mendelsohn J, Calzavara L, Cheng H, et al. DEVELOPMENT AND DESIGN OF AN OBSERVATIONAL IMPACT EVALUATION OF IMMEDIATE ANTIRETROVIRAL THERAPY ON HIV INCIDENCE AMONG HIV-POSITIVE MEN WHO HAVE SEX WITH MEN IN SHANGHAI, CHINA. American Journal of Tropical Medicine and Hygiene; Oct2015. p. 531-531.
194. Peng T, Yi L, Lan Y, et al. FACTORS AFFECTING SEXUAL SAFETY AMONG HIV-POSITIVE MEN WHO HAVE SEX WITH MEN IN HARBIN, CHINA: IMPLICATIONS FROM A QUALITATIVE RESEARCH. Journal of Sexual Medicine; Aug2015. p. 358-358.
195. Qian HZ, Lou J, Ruan Y, et al. Modeling of HIV epidemic among men who have sex with men in Beijing, China. Journal of the International Aids Society; Oct2012. p. 79-80.
196. Qin Y, Liu F, Tang W, et al. Benefits and adverse outcomes of HIV self-testing among high-risk MSM in China: an implementation perspective. Journal of the International Aids Society; Jul2016.
197. Tseng PY. Gendered HIV Prevention Policies and Self-Diagnosis of Men Who Have Sex with Men (MSM) in Taiwan. International Journal of Psychology; Jul2016. p. 1000-1000.
198. Wang XF, Wu ZY, Tang ZZ, et al. Promoting HIV testing with home self-test kit among men who have sex with men in China: a feasibility study. Lancet; Oct2015. p. 68-68.
199. Wei CZ, Liu WL. Coming out, school climate and mental health of sexual minority students in Mainland China. International Journal of Psychology; Jul2016. p. 1005-1006.
200. Wu D, Smith MK, Ong JJ, et al. Social-media based secondary distribution of HIV self-testing among Chinese men who have sex with men: A pilot implementation programme assessment. Journal of the International Aids Society; Jul2019. p. 56-56.
201. Yao Y, Li B, Gilbert S, et al. A Survey of the Prevalence of HIV and Syphilis Infections and Sexual Behaviors Among MSM in Jilin Province, China. Jaids-Journal of Acquired Immune Deficiency Syndromes; Apr2012. p. 72-72.
202. Zheng Y, Lu H, Jiang X, et al. Epidemiology of HIV infection among men having sex with men in Shanghai, China. Southeast Asian J Trop Med Public Health; Sep2012. p. 1304-6.
203. Jia ZW, Huang XJ, Wu H, et al. HIV burden in men who have sex with men: a prospective cohort study during 2007-12 in Beijing, China. Lancet; Nov2013. p. 19-19.
204. Mendelsohn JB, Cheng H, Calzavara L, et al. Key gaps in the HIV cascade of care among men who have sex with men and migrants diagnosed with HIV: findings from a surveillance study in Shanghai. The Lancet. 2018 2018/10/01/;392:S85.

205. Tang W, Mao J, Liu C, et al. Crowdsourcing health communication about condom use in men who have sex with men in China: a randomised controlled trial [Journal: Conference Abstract]. Lancet. 2016;388(SPEC.ISS 1):73-.

206. Zhang T, Tang W, Sesh T, et al. Crowdsourcing to promote hiv testing among msm in china: a pragmatic stepped wedge randomized controlled trial of a crowdsourced intervention [Journal: Conference Abstract]. Annals of global health. 2017;83(1):6-.

207. Cheng W, Friedman MR, Egan JE, et al. HIV-testing patterns and psycho-social factors associated with frequency of testing by men who have sex with men: implication for scaling up HIV testing in China. The Lancet; Dec2017. p. S45-S45.

208. Fu R, Zhang X, Tang W, et al. Mapping the HIV and syphilis epidemic and sexual behaviours in men who have sex with men in China: a meta-analysis. The Lancet; 2017/12/01/2017. p. S19.

209. Kwan TH, Poon CM, Wong KH, et al. Effects of HIV diagnosis and enrolment in care on transmission risk of HIV-infected men who have sex with men in Hong Kong: a cross-sectional study. The Lancet; 2016/10/01/2016. p. S48.

210. Liu C, Zhang Y, Pan SW, et al. Anticipated HIV stigma among men who have sex with men and transgender individuals in China: a cross-sectional study. The Lancet; 2017/12/01/2017. p. S70.

211. Mao J, Tang W, Liu C, et al. Sex tourism among Chinese men who have sex with men: a cross-sectional observational study. The Lancet; 2016/10/01/2016. p. S75.

212. Ong JJ, Li H, Dan W, et al. Coercion and HIV self-testing in Chinese men who have sex with men: implementation data from a cross-sectional survey. The Lancet; 2017/12/01/2017. p. S57.

213. Qin Y, Liu F, Tang W, et al. HIV self-testing among high-risk men who have sex with men in China: a cross-sectional study. The Lancet; 2016/10/01/2016. p. S76.

214. Tang S, Tang W, Meyers K, et al. HIV and syphilis among men who have sex with men and transgender individuals in China: a scoping review. The Lancet; 2016/10/01/2016. p. S74.

215. Tang W, Cao B, Liu C, et al. HIV serostatus disclosure from partners before sex: results from an online survey of Chinese men who have sex with men. The Lancet; 2017/12/01/2017. p. S76.

216. Tang W, Mao J, Tang S, et al. Disclosure of sexual orientation in men who have sex with men in China: a cross-sectional survey. The Lancet; 2016/10/01/2016. p. S68.

217. Zhao P, Tang S, Wang C, et al. Recreational drug use among Chinese MSM: results from a national-wide cross-sectional study. The Lancet; 2016/10/01/2016. p. S69.

218. Zhao P, Zhang Y, Cao B, et al. The interaction between social norm and self-efficacy on HIV testing among Chinese men who have sex with men: results from an online cross-sectional study. The Lancet; 2017/12/01/2017. p. S79.

219. Xing JM, Zhang KL. Men who have sex with men(MSM) and HIV epidemic in China: a web-based study on MSM. Retrovirology; May2010. p. 47-48.

220. Chen X, Jiang N, Wang B. HIV/STI PREVALENCE AMONG MEN WHO HAVE SEX WITH MEN IN 4 CITIES, CHINA AND ASSOCIATED RISK FACTORS FOR HIV INFECTION. Sexually Transmitted Infections; Jul2011. p. A50-A50.

221. Jun-Jie X, Ke Y, Reilly KH, et al. A META-ANALYSIS OF THE PREVALENCE OF HIV/SYPHILIS AMONG CHINESE BISEXUAL MEN WHO HAVE SEX WITH MEN (MSM). Journal of Epidemiology and Community Health; Aug2011. p. A117-A117.
222. Zheng YF, Lu HZ, Pan CG, et al. Epidemiology of HIV infection among men who have sex with men in Shanghai, China. International Journal of Infectious Diseases; Jul2011. p. S38-S38.
223. Qian HZ, Ruan YH, Liu Y, et al. Does Circumcision Offer Some Protection from HIV for Men who Have Sex with Men? A Cross-sectional Study in China. Aids Research and Human Retroviruses; Oct2014. p. A197-A198.
224. Huang J, Kuo P. Does Men's Body Satisfaction with Specific Parts Differentially Affect Body-image-related Mental Distress by Sexual Orientation? a Comparison of Gay, Bisexual, and Heterosexual Men in Taiwan. European Psychiatry; 2015/03/28/2015. p. 683.
225. Qian HZ, Ruan Y, Tao J, et al. Impact of text messaging and peer counseling on initiation and adherence of HIV antiretroviral therapy among newlydiagnosed MSM. Journal: Conference Abstract presented at: AIDS research and human retroviruses; Oct2016. p. 367-.
226. Yang LG, Zhang XH, Chen ZY, et al. PREVALENCE OF NEISSERIA GONORRHOEAE AND CHLAMYDIA TRACHOMATIS INFECTIONS IN DIFFERENT ANATOMIC SITES AMONG MEN WHO HAVE SEX WITH MEN: RESULTS OF 380 MSM WHO ATTENDED A STD CLINIC IN GUANGZHOU, CHINA. Sexually Transmitted Infections; Jul2017. p. A38-A39.
227. Jiang H, Li J, Chen X, et al. Syndemic factors associated with sexual HIV risk behaviors among men who have sex with men in Guangzhou, China. International Journal of Infectious Diseases; Aug2018. p. 246-247.
228. Cheng W, Friedman M, Egan J, et al. Impact of HIV self-testing on the promotion of HIV testing among men who have sex with men in China: a randomized controlled trial. Journal: Conference Abstract presented at: Sexually transmitted infections2019. p. A64-.
229. Huang W, Wang Y, Lu H, et al. High HIV incidence among men who have sex with men in eight chinese cities: results from a cohort study. Journal: Conference Abstract presented at: Sexually transmitted infections2019. p. A243-.
230. Zhang T, Yang F, Tang W, et al. Pay-it-forward gonorrhea and chlamydia testing among Chinese men who have sex with men: a cluster randomized controlled trial. Journal: Conference Abstract presented at: Sexually transmitted infections2019. p. A89-.
231. Hsieh YH, Ruan Y, Chen CW, et al. HIV prevalence and underreporting of men who have sex with men in Beijing. Int J STD AIDS. 2012 Aug;23(8):606-7.
232. Peng J, Li K, Zhang C, et al. Human papillomavirus and polyomavirus coinfections among Chinese men who have sex with men. J Infect. 2016 Jan;72(1):118-20.
233. Chow EP, Wilson DP, Zhang L. The next era of HIV in China: rapidly spreading epidemics among men who have sex with men. J Acquir Immune Defic Syndr. 2010 Dec;55(4):e32-3; author reply e33-4.
234. Zhao J, Cai W, Zheng C, et al. Origin and outbreak of HIV-1 CRF55-01B among MSM in Shenzhen, China [Letter]. Journal of Acquired Immune Deficiency Syndromes. 2014 Jul 1;66(3):e65-e67.
235. Jia MH, Sullivan SG, Lu L, et al. Curbing the Rapid Spread of HIV Among MSM in China. Jaids-Journal of Acquired Immune Deficiency Syndromes. 2010 Dec;55(4):E33-E34.

236. Chen X, Yang HT, Norris J, et al. Retention challenge in an MSM cohort in Suzhou, China. Sex Transm Infect. 2012 Oct;88(6):455.
237. Chan PC. Reemerging threat of hepatitis A among men who have sex with men in Taiwan. J Formos Med Assoc. 2014 Dec;113(12):985-6.
238. Han M, Pan PL, Wu GH, et al. Estimating HIV Incidence Rates among MSM in an Urban Area of Chongqing Using Three Approaches. Biomed Environ Sci. 2017 Dec;30(12):913-916.
239. Liao YS, Liu YY, Lo YC, et al. Azithromycin-Nonsusceptible Shigella flexneri 3a in Men Who Have Sex with Men, Taiwan, 2015-2016. Emerging Infectious Diseases. 2017 Feb;23(2):345-346.
240. Ren Z, Yuan C. Mental health professionals' ethical dilemma when working with gay men who are in heterosexual marriages in China. Journal of Gay & Lesbian Mental Health. 2018 Jul;22(3):302-307.
241. ChiCtr. Effect of comprehensive intervention based on HIV risk prediction on HIV-related high-risk behavior among MSM in China. http://wwwwhoint/trialsearch/Trial2aspx?TrialID=ChiCTR1800017268. 2018.
242. Han L, Tang W, Best J, et al. Crowdsourcing to spur first-time HIV testing among men who have sex with men and transgender individuals in China: a non-inferiority pragmatic randomized controlled trial [Journal: Conference Abstract]. Journal of the international AIDS society. 2015;18:60-61.
243. Tao J, Wang L, Kipp A, et al. Stigma and depression among newly diagnosed HIV infected MSM in china [Journal: Conference Abstract]. Journal of acquired immune deficiency syndromes. 2016;71:49-.
244. Tucker J. A Non-inferiority Randomized Controlled Trial to Evaluate Promoting Condom Use Among MSM and Transgender Individuals in China. https://clinicaltrialsgov/show/NCT02516930. 2015.
245. Wu D, Tang W, Lu H, et al. Leading by Example: web-Based Sexual Health Influencers Among Men Who Have Sex With Men Have Higher HIV and Syphilis Testing Rates in China [Journal Article; Randomized Controlled Trial; Research Support, N.I.H., Extramural; Research Support, Non-U.S. Gov't]. Journal of medical Internet research. 2019 Jan 21;21(1):e10171.
246. Zou H, Tucker JD, Fan S, et al. Learning about HIV the hard way: HIV among Chinese MSM attending university. Lancet Infect Dis. 2018 Jan;18(1):16-18.
247. Muessig KE, Cohen MS. China, HIV, and Syphilis Among Men Who Have Sex With Men: An Urgent Call to Action. Clinical Infectious Diseases. 2013 Jul;57(2):310-313.
248. Shen YZ, Yu VL, Lu HZ. Control of HIV for Men Who Have Sex With Men in China: A Role for the Internet? Clinical Infectious Diseases. 2016 Jun;62(11):1448-1449.
249. Wang YY, Hu ZS, Peng K, et al. Discrimination against LGBT populations in China. Lancet Public Health. 2019 Sep;4(9):E440-E441.
250. Wiysonge CS, Kongnyuy EJ, Shey M, et al. Male circumcision for prevention of homosexual acquisition of HIV in men. Cochrane Database of Systematic Reviews. 2011 Jun 15(6):CD007496.
251. McLaughlin K. HIV infections are spiking among young gay Chinese: Officials strive to understand infection routes and promote prevention and testing. Science. 2017 Mar 31:1359.

252. Lee SS, Tam DK, Mak DW, et al. Use of the Internet for sex partnership in men who have sex with men before HIV infection. Public Health. 2011 Jul;125(7):433-5.
253. Chen XS, Yin YP, Jiang N, et al. Setting typologies and HIV prevalence among men who have sex with men in China: implication for surveillance and intervention. Sex Transm Dis. 2012 Mar;39(3):226-8.
254. Huang YF, Chen CH, Chang FY. The emerging HIV epidemic among men who have sex with men in Taiwan. J Formos Med Assoc. 2013 Jul;112(7):369-71.
255. Huang SZ, Brouwer DC. Negotiating Performances of "Real" Marriage in Chinese Queer Xinghun. Womens Studies in Communication. 2018;41(2):140-158.
256. Kong TSK. Gay and grey: Participatory action research in Hong Kong. Qualitative Research. 2018 Jun;18(3):257-272.
257. Wei CY, Raymond HF. Pre-exposure prophylaxis for men who have sex with men in China: challenges for routine implementation. Journal of the International Aids Society. 2018 Jul;21(7).
258. Zhu J. ‘Unqueer’ kinship? Critical reflections on ‘marriage fraud’ in mainland China. Sexualities. 2018 Oct;21(7):1075-1091.
259. Hildebrandt T. The one-child policy, elder care, and LGB Chinese: A social policy explanation for family pressure. Journal of Homosexuality. 2019;66(5):590-608.
260. Chiou P-Y, Lin L-C, Chen Y-M, et al. The Effects of Early Multiple-Time PN Counseling on Newly HIV-Diagnosed Men Who Have Sex with Men in Taiwan. AIDS & Behavior. 2015 Oct;19(10):1773-1781.
261. Huang Y. Media Representation of Tongxinglian in China: A Case Study of the People's Daily. J Homosex. 2018;65(3):338-360.
262. Xing JM, Zhang KL, Chen X, et al. A cross-sectional study among men who have sex with men: a comparison of online and offline samples in Hunan Province, China. Chin Med J (Engl). 2008 Nov 20;121(22):2342-5.
263. Li J, Song J, Tan G, et al. High Seroprevalence of Human Herpesvirus 8 Infection in HIV-positive Homosexual Men in Jiangsu Province, China. Curr HIV Res. 2017;15(4):273-278.
264. Wei T. Measuring Minority Stigma Among Chinese Women with Same-Sex Attraction [Ph.D.]. Ann Arbor: The University of Oklahoma Health Sciences Center; 2017.
265. Wong IML. Psychological wellbeing of chinese sexual minority Christians in Hong Kong: Influence of individual and community resilience factors [Psy.D.]: Alliant International University; 2018.
266. Luo S. Barriers to Expanding HIV Testing among Male Students Who Have Sex with Men in Chengdu, China [Ph.D.]. Ann Arbor: University of California, Los Angeles; 2019.
267. Steele SJ. Chronicling the relationships, attitudes, and sexual encounters of men who have sex with men in Shanghai, China: A web-administered sexual events diary [Ph.D.]: University of Toronto (Canada); 2017.
268. Chan AKN. The Role of Interpersonal Competence in Relation to Psychological Symptoms Among Lesbian and Gay Adults in Hong Kong [Psy.D.]. Ann Arbor: Alliant International University; 2017.

269. Davis A. Differences in risk behaviors, intimate partner violence, and HIV & STI prevalence among men who have sex with men and men who have sex with men and women in China.: Indiana University; 2015.
270. Feng Y. Prevalence of HIV/STDs among MSM in Chengdu, China and associated risk factors [Ph.D.]. Los Angeles: University of California, Los Angeles; 2008.
271. Li Y. Understanding Chinese lesbians' subjective well-being during sexual identity development: University of Northern Colorado; 2009.
272. Wang L, Wang L, Norris JL, et al. HIV prevalence and influencing factors analysis of sentinel surveillance among men who have sex with men in China, 2003 - 2011. Chin Med J (Engl). 2012 Jun;125(11):1857-61.

Studies ineligible following full text screening-Traditional Chinese

Record	Type	Excluded studies	Reasons for exclusion	
1	Thesis	劉安真 (Liu Anchen) (2001)	Non-Health	Study did not investigate any health-related variables.
			Non-Context	Study conducted from 1988 to 1989.
2	Journal article	畢恆達 (Herng-Dar Bih) (2003)	Non-Health	Study only discussed the stage of coming out, no other health-related issues.
3	Thesis	范庭豪 (Fan Ting-Hao) (2010)	No available full text	This electronic full text is limited to the campus system to which the graduate student belongs (本篇電子全文限研究生所屬學校校內系統及 IP 範圍內開放).
4	Journal article	陳立言 (Li-Yen Chen) (2011)	Non-Health	Study did not investigate any health-related variables, while only some explanation and discussion of some phenomena and assumptions.
5	Book section	現代婦女教育基金會 (2012)	Non-academic	This was just a book section.
			Non-Health	This did not conduct any investigations, just self-help manual.
6	Thesis	鍾仁惟 (Chung Jen-Wei) (2013)	Non-Population	Study only included 4 participants, while 2 were under 18 years old.
7	Journal article	陳伯杰 et al. (2013)	Non-Population	Study samples were visitors of gay saunas, but no description of sexual orientation.
8	Journal article	黃志中 and 莊瑞君 (2013)	Non-Population	It's not a scientific study with no description of population.
9	Thesis	張嘉珍 (Chang Chia-Chen) (2015)	No available full text	The paper version of this thesis is delayed until August 20, 2020, and the reason for the delay is it was planning to be published in an international journal. (紙本延遲至民國 109 年(公元 2020 年)8 月 20 日後公開上架, 延遲原因: 發表國際期刊)
10	Thesis	洛彤 (Luo Tong) (2015)	No available full text	The paper version of this thesis was delayed until July 1, 2017 for the reason that it was planning to be published. But currently, there was still no available full text. (紙本延遲至民國 106 年 7 月 1 日後公開上架, 延遲原因: 發表期刊)

11	Thesis	吳慧娟 (Wu Huei-Jiuan) (2016)	No available full text	The paper version of this thesis is delayed until August 19, 2021, and the reason for the delay is that it was planning to be submitted and published. (紙本延遲至 2021 年 08 月 19 日後公開上架, 延遲原因 :投稿)
12	Journal article	王甯祺 and 林德宇 (2017)	Non-Population	Only mentioned the MSM group, no specific data.
13	Journal article	李大鵬 and 鍾道詮 (2018)	Non-Population	Publication only summarized the experience and the management of a gay community-based organization.
14	Journal article	莊苹 (2018)	Non-Population	This article was the observations of a nurse who had been working with people living with HIV/AIDS, but not from the perspective of MSM themselves.
15	Thesis	黃義筌 (Huang I. Chuan) (2018)	Non-Population	Study did not mention sexual orientation, but only included people living with HIV.
16	Journal article	黃翠紋 (2018)	Non-Health	This article was a literature review, while only focus on the 'Domestic Violence Prevention Act'.
17	Thesis	王建權 (Wang Chien-Chuan) (2019)	No available full text	Its electronic full text is nowhere to be found, and the paper version of the full text can only be borrowed in their university library.
18	Journal article	Freire, Grossman, and Frank (2010)	Non-Population	The populations were US sexual minorities.
19	Journal article	許維邦, 張瑞月, 蕭培靜, and 陳民虹 (2016)	Non-Population	This review described no population in Taiwan or other Chinese sample.
20-21	Journal article	徐志雲 and 顏正芳 (2017); 黃馨瑩 (2016)	Non-Health	Both articles seemed to be reviews while only described some social opinions and laws-related issues.

Excluded references:

Freire, A., Grossman, A. H., & Frank, J. A. (2010). Sexual Non-minority and Sexual Minority Youth: Identity Development, Mental Health, and Risky Recreational Sex Behaviors 青少年的性少數與性多數族群之研究：認同發展、心理健康與風險性的遊憩性行為. *休閒與遊憩研究, 4*(1), 頁 1-37.

劉安真 (Liu Anchen). (2001). *「女同志」性認同形成歷程與污名處理之分析研究*. (博士 Doctor of Philosophy). 國立彰化師範大學, Retrieved from https://hdl.handle.net/11296/gu92tg

吳慧娟 (Wu Huei-Jiuan). (2016). *HIV 暴露前預防投藥策略對台灣男男間性行為傳染愛滋疫情的防治效果 : 數理模式研究 (HIV pre-exposure prophylaxis for men who have sex with men in Taiwan : a mathematical modeling study).* (碩士 Master of Philosophy). 國立臺灣大學, Retrieved from https://hdl.handle.net/11296/z8cj6p

張嘉珍 (Chang Chia-Chen). (2015). *擴大 HIV"篩檢與治療" 策略對臺灣男男同性間性行為傳染愛滋疫情的防治效果 : 數理模式研究 (Impact of expanding hiv "test-and-treat" strategy on the hiv epidemic among men who have sex with men (msm) in Taiwan : a modeling study).* (碩士 Master of Philosophy). 國立臺灣大學, Retrieved from http://www.airitilibrary.com/searchdetail.aspx?DocIDs=U0001-1708201517423300

徐志雲, & 顏正芳. (2017). 同性婚姻之健康促進. [Same-Sex Marriage for Health Promotion]. *臺灣醫學, 21 卷*(2 期), 頁 194-202.

李大鵬, & 鍾道詮. (2018). 同志健康社區服務中心經營反思：以大臺北同學會為例. *東吳社會工作學報*(34 期), 頁 45-65.

洛彤 (Luo Tong). (2015). *早期 HIV 診斷對感染 HIV 男男間性行為者生活品質調整後平均餘命的影響 = Impact of early hiv diagnosis on quality-adjusted life expectancy in hiv-infected men who having sex with men (msm).* 民 104[2015], Retrieved from http://www.airitilibrary.com/searchdetail.aspx?DocIDs=U0001-1708201500585900

王建權 (Wang Chien-Chuan). (2019). *恐同對待對同性戀和雙性戀男性之精神健康影響 (Effects of Homophobic Maltreatment on Mental Health in Gay and Bisexual Men).* (博士 Doctor of Philosophy). 高雄醫學大學, 高雄市. Retrieved from https://hdl.handle.net/11296/77f2cn

王甯祺, & 林德宇. (2017). 急性 A 型肝炎自 2015 年開始於男男性行為族群的群聚感染. *感染控制雜誌, 27 卷*(2 期), 頁 53-58.

現代婦女教育基金會. (2012). *衣櫃中的傷痕 : 同志伴侶親密暴力自助手冊* (初版 ed.): 臺北市 : 現代婦女教育基金會, 臺灣同志諮詢熱線協會, 2012.09.

畢恆達 (Herng-Dar Bih). (2003). 男同性戀與父母：現身的考量、策略、時機與後果 (Gay men and their parents: Decisions, Strategies, and Consequences of Coming Out). *Journal of Women's and Gender Studies*(15), 37-78. doi:10.6255/JWGS.2003.15.37

范庭豪 (Fan Ting-Hao). (2010). *男同性戀者社會支持與旅遊參與動機之關係 (The relation of social support and travel participation motivation of gay men).* (碩士 Master of Philosophy). 中國文化大學, Retrieved from https://hdl.handle.net/11296/j87tae Available from Ma001469294;bg004929677;bi001492264;bj000449216

莊苹. (2018). 男同志的成長與藥／愛的糾結. *東吳社會工作學報*(34 期), 頁 69-85.

許維邦, 張瑞月, 蕭培靜, & 陳民虹. (2016). 男同性戀患者之基層照護. *家庭醫學與基層醫療, 31 卷*(6 期), 頁 187-191.

鍾仁惟 (Chung Jen-Wei). (2013). *青少年男同志向同儕出櫃對其性認同形成及面對歧視之敘事研究 (The Narrative Research of Sexual Identity Development and Discrimination of Adolescent Gays When Coming out to Their Peers).* (碩士 Master of Philosophy). 大葉大學, Retrieved from https://hdl.handle.net/11296/37gbxe Available from Ma016753561;bj000543365

陳伯杰, 鍾安峻, 吳孟姿, 陳俊霖, 張逸玫, 楊玉玟, . . . 柯乃熒. (2013). 同志三溫暖推動友善、健康及安全商店標章計畫之監測與成效分析. [The Analysis of Surveillance and Effectiveness of the “Friendly, Healthy and Safe Business Certification Program” for Gay Saunas]. *疫情報導, 29 卷*(23 期), 頁 361-368.

陳立言 (Li-Yen Chen). (2011). 在愛中沒有恐懼--同性戀行為之性愛倫理分析. [There Is No Fear in Love--A Study on the Morality of Homosexual Behavior]. *生命教育研究 (Journal of Life Education), 3 卷*(1 期), 頁 1-39.

黃志中, & 莊瑞君. (2013). 性少數的健康照顧：權利與困境. *婦研縱横*(99 期), 頁 12-16.

黃義筌 (Huang I. Chuan). (2018). *男男（MSM）愛滋感染者的性實踐經驗與主體建構 (Subject Construction in Sexual Practices of Men having Sex with Men (MSM) and Living with HIV)*. (碩士 Master of Philosophy). 國立高雄師範大學, Retrieved from https://hdl.handle.net/11296/s62p4f Available from Ma019786901;bj000651998

黃翠紋. (2018). 同性間親密關係暴力防治--家庭暴力防治法實施廿周年的展望. *中央警察大學學報*(55 期), 頁 1-28.

黃馨瑩. (2016). 「她與她」的壓力源及社會支持網絡. *諮商與輔導*(361 期), 頁 25-28.

Studies ineligible following full text screening-Simplified Chinese

Record of Excluded studies	Reference type	Reasons for exclusion	Summary
[1, 2]	Journal article	Study populations were visitors to STD clinic.	**Non-Population**<br><br>**N=150**<br><br>Did not meet the inclusion criteria in terms of population
[3-46] [47-67]	Journal article	MSM or homosexual population was one of some risk population groups in these studies, while there was no specific analysis of the target group.	
[68, 69]	Thesis		
[70-74]	Journal article	The target populations were healthcare providers, organization managers, social workers, or volunteers, but not MSM themselves.	
[75-77]	Thesis		
[78]	Journal article	Study populations were visitors of a gay website.	
[79]	Journal article	This review just mentioned gay population in one paragraph and did not review any specific health-related issues.	
[80-94]	Journal article	Studies investigated people contacting with HIV only/mostly.	
[95]	Journal article	Study populations were female college students.	
[96]	Journal article	Study populations were patients with ED.	
[97]	Journal article	Study focused on the MSM website and interviewed those managers.	
[98]	Journal article	Study populations were researchers of MSM projects.	
[99-130]	Journal article	Studies explored feasibility or effectiveness of HIV prevention programs from the CDC/clinic/NGOs/Bar owners' perspective but not MSM's.	
[131, 132]	Journal article	Only stated populations were the MSM, but no specific description.	
[133, 134]	Journal article	Study recruited only homosexual adolescents aged 11-19 or <19 years old.	
[135]	Journal article	Study population were people who visited the voluntary counseling and testing (VCT).	
[136-139]	Journal article	Study populations were college students or male university students.	
[140]	Thesis		
[141]	Thesis	This study investigated public's attitude towards gay people.	

[142]	Thesis	The study subjects were blood samples, not human being.	
[143]	Thesis	Study only mentioned sexual minorities while no specific data of homosexual or bisexual population.	
[144, 145]	Thesis	Population inclusion criteria did not mention either sexual orientation or behavior orientation.	
[146-148]	Journal article	Study samples were blood samples or HIV antibody urine samples from people of both genders, but not human beings.	
[149]	Journal article	Study populations were carers of HIV-infected MSM.	
[150]	Journal article	Study populations were male clients in anorectal departments.	
[151, 152]	Journal article	Both articles were literature reviews, while mainly focused on the reasons of homosexual and potential intervention plan.	**Non-Concept (health)**<br><br>**N=154**<br><br>Did not meet the inclusion criteria in terms of concept, e.g.: holistic health
[153]	Journal article	This was a case report talking about an attempted murder.	
[154-176]	Journal article	Studies investigated mainly the epidemiological characteristics or immunity status or molecular biological characteristics of HIV subtypes.	
[177-183]	Thesis		
[184, 185]	Journal article	Both reviews did not mention health-related issues.	
[186-191]	Journal article	These studies did not investigate any people-perceived health or other health-related variables.	
[192-204]	Journal article	Study targeted mainly on the comparison of sampling methods or factors of cohort retention, but not the health-related issues.	
[205-208]	Journal article	These articles reviewed no information of health issues.	
[209, 210]	Journal article	Studies investigated non-health variables, only aversive emotion regulation or the quality of semen.	
[211-232]	Journal article	Studies described only sociodemographic data but no investigation of specific health-related variables.	
[233-244]	Journal article	These studies discussed the development of sexual identity or focused on minorities' ideology but no other investigation of their perceived health.	
[245-257]	Thesis		

[258-275]	Journal article	Studies aimed to estimate the size of MSM population or evaluate some mathematical/prediction/risk models.	
[276-279]	Thesis		
[280, 281]	Thesis	Both studies just summarized some stories/interviews while not specifically focus on the health-related issues.	
[282]	Journal article	Study reported specific treatment method while not MSM perceived health.	
[283]	Thesis	It's a case report by a social worker while no clear description or measurements of anything relating health.	
[284]	Thesis	Study only evaluated cost-effectiveness of the homosexual clinic.	
[285-287]	Journal article	This article investigated just disease-related symptoms but not health.	
[288-298]	Journal article	These studies evaluated some testing methods or treatments while not target MSM's health.	
[299]	Journal article	It's a case report of a male student whose experience of coming out.	
[300-304]	Journal article	Studies only analyzed temporal and spatial distribution characteristics or spatial clustering characteristics of MSM.	
[305]	Journal article	Study conducted from 2000.8 to 2000.10.	**Non-Context**<br><br>**N=28**<br><br>Did not meet the inclusion criteria in terms of concept (Chinese, in or after 2001).
[306]	Journal article	Study conducted from 1994 to 1997.	
[307]	Journal article	Study conducted in 1999.	
[308]	Journal article	Study conducted from 1998 to 2000.	
[309, 310]	Journal article	Study conducted from 1997 to 1998.	
[311-314]	Journal article	Study conducted before 2001.	
[315]	Journal article	This article only reviewed syphilis overseas but not in China.	
[316]	Thesis	Study about mental health was conducted from 1998 to 2000.	
[317-328]	Journal article	No information about study setting or sources of population.	
[329-332]	Journal article	These reviews only described situations in the US or overseas.	
[333]	Conference paper	This publication was meeting abstract published in the journal.	

[334-344]	Journal article	The descriptions of the text in these articles were inconsistent.	**Others**<br><br>**N=102**<br><br>Did not meet the inclusion criteria in terms of study types, or non-academic
[345-352]	Short reports	These publications were short reports published in specific journals, without enough PCC information or relevant descriptions.	
[353]	Journal article	Non-academic, this article did not mention any details about study population and measurements and had no reference as well.	
[354-358]	Journal article	These articles were summaries of the work of some NGOs or CBOs.	
[359-382]	Journal article	These publications were only personal opinions sharing.	
[383-400]	Journal article	These studies were supposed to be intervention studies, but no information about either intervention or measurements was mentioned.	
[401-419]	Journal article	These articles had no clear description of study population.	
[420-428]	Journal article	No clear description of either study methods, measurements, or outcomes.	
[429, 430]	Journal article	No available full texts.	
[431]	Journal article	No sample size.	
[432]	Journal article	The content of this article was same with another article[433], so keep only one with more details.	
[434]	Journal article	The content of this article was same with another one[435], so keep only one with more details.	
[436]	Journal article	The content of this article was same with another one[437], so keep only one published in the journal with higher impact factor.	
**All excluded references (434) = Number of references (437)- 3**			

Excluded references:

[1] 刘意，黄晓婕，魏虹绢，等. 性病门诊就诊者中男男同性恋者HIV检测情况分析[J]. 中国艾滋病性病， 2007， 13(04): 353-354.
[2] 常晓，连石，张海萍. 男性性病门诊就诊者HIV感染状况的调查及分析[J]. 中国艾滋病性病， 2012， 18(03): 154-155.
[3] 郭蕾，韩孟杰，余冬保. 高危行为人群艾滋病自愿咨询检测需求分析[J]. 疾病监测， 2006， 21(07): 355-357.
[4] 李东民，孙新华，曾刚，等. 2005年中国高危人群艾滋病哨点监测报告[J]. 中国艾滋病性病， 2007(01): 1-3.
[5] 李宁，王哲，孙国清，等. 河南省2006年高危险人群艾滋病哨点监测分析[J]. 中国艾滋病性病， 2007(05): 427-429.

[6] 张冬梅，汤增辉. 我国艾滋病高危人群安全套使用影响因素分析[J]. 现代预防医学， 2008， 35(20): 3970-3971.
[7] 柯贤洲，肖少卿，邓健，等. 黄石市各类高危人群艾滋病感染情况调查分析[J]. 公共卫生与预防医学， 2008， 19(04): 59.
[8] 裘丹红，沈伟伟，徐佳. 2005~2007年台州市艾滋病高危人群HIV感染情况监测分析[J]. 中国卫生检验杂志， 2008， 18(07): 1405-1406.
[9] 姜珍霞，修翠珍，张西江，等. 青岛市高危行为人群艾滋病性病干预效果分析[J]. 中国麻风皮肤病杂志， 2009， 25(08): 630-631.
[10] 张万宏，聂绍发，石卫东，等. 艾滋病高危人群利用自愿咨询检测服务影响因素研究[J]. 现代预防医学， 2010， 37(08): 1405-1407.
[11] 向德平，吴丹. 艾滋病患者社会支持与生存质量的比较研究——基于对有偿供血人群、MSM人群及女性性工作者的调查[J]. 社会科学战线， 2010(04): 194-200.
[12] 李保军，梁良，赵宏儒，等. 河北省艾滋病哨点监测现状及分析[J]. 中国热带医学， 2010， 10(05): 554-615.
[13] 李雷，还锡萍，徐金水，等. 江苏省2006年～2008年艾滋病高危人群哨点监测报告[J]. 江苏预防医学， 2010， 21(02): 1-3.
[14] 曹会珍，魏学前，张庆娟，等. 唐山市2006-2009年不同人群HIV抗体检测结果分析[J]. 中国艾滋病性病， 2010， 16(05): 479-481.
[15] 席胜军，何玉芳，周晓红. 下城区CSW、MSM和IDU人群艾滋病干预检测结果分析[J]. 浙江预防医学， 2010， 22(09): 29-30.
[16] 张红，孔苏卫，王川勇，等. 宜宾市4类人群HCV检测结果分析[J]. 寄生虫病与感染性疾病， 2011， 9(04): 233-234.
[17] 王凯，赵丽，王赫秋，等. 鞍山市HIV自愿咨询检测利用现状及影响因素[J]. 中国公共卫生， 2011， 27(10): 1297-1299.
[18] 任旭琦，杨立刚，王晓华，等. 广东省性病高危人群中梅毒流行情况调查[J]. 中国艾滋病性病， 2011， 17(04): 436-438.
[19] 刘聪，许骏，周旺，等. 湖北武汉中盖艾滋病项目三类高危人群基线调查分析[J]. 公共卫生与预防医学， 2011， 22(02): 15-18.
[20] 聂志强，林鹏，李艳，等. 广东省2009年高危人群HIV哨点综合监测结果分析[J]. 热带医学杂志， 2011， 11(01): 29-31.
[21] 苏秋菊，位晓东. 鞍山市2010年七类人群HIV检测及调查结果[J]. 中国艾滋病性病， 2011， 17(06): 699.
[22] 张志芳，许宁，赵长城，等. 西宁市不同人群HIV、HCV、梅毒感染状况调查分析[J]. 医学动物防制， 2011， 27(11): 1017-1018.
[23] 张宏，陈传刚，林风华，等. 福州市2010年5类艾滋病高危人群综合监测结果分析[J]. 职业与健康， 2011， 27(21): 2406-2409.
[24] 谢爱齐，崔德勇，邱兴庆，等. 襄阳市2010年艾滋病哨点监测结果分析[J]. 公共卫生与预防医学， 2011， 22(05): 95-97.
[25] 符振旺，孙莲英，曾雪霞. 海南省特殊人群乙型病毒性肝炎血清学调查分析[J]. 现代预防医学， 2012， 39(23): 6285-6287.
[26] 高菲菲，钟晓妮，彭斌，等. HIV感染高危人群暴露前预防用药临床试验依从性分析[J]. 第二军医大学学报， 2012， 33(11): 1186-1193.
[27] 周丹，周金玲，姜凤霞，等. 2011年辽宁省艾滋病哨点高危人群监测结果分析[J]. 中国艾滋病性病， 2012， 18(10): 648-650.
[28] 陈炳莲，朱平，刘利，等. 大安区特殊人群艾滋病、梅毒和丙肝监测结果及对策[J]. 中国卫生事业管理， 2012， 29(10): 798-799.
[29] 汤杰，张振开，周芸，等. 2011年桂林市艾滋病高危人群哨点监测分析[J]. 中国艾滋病性病， 2012， 18(08): 533-536.
[30] 成浩，顾静，缪小兰，等. 2010-2012年无锡市艾滋病高危人群综合监测结果分析[J]. 中华疾病控制杂志， 2013， 17(12): 1086-1088.
[31] 张曼，芮宝玲，王云霞，等. 乌鲁木齐市高危人群HIV感染情况和相关行为学调查[J]. 职业与健康， 2013， 29(04): 477-478.
[32] 秦其荣，马颖，高媛，等. 马鞍山市三类高危人群艾滋病歧视态度调查分析[J]. 中国农村卫生事业管理， 2014， 34(06): 684-686.
[33] 罗雅凌，廖清华，龚正华，等. 江西四类高危人群接受艾滋病预防与检测服务情况分析[J]. 基层医学论坛， 2014， 18(28): 3863-3865.
[34] 张静瑜，栾荣生，罗映娟，等. 2012年四川省艾滋病高危人群哨点监测结果分析[J]. 中国艾滋病性病， 2014， 20(02): 91-94.
[35] 董薇，周楚，葛琳，等. 2008-2014年中国预防艾滋病经性传播干预措施落实情况分析[J]. 中华流行病学杂志， 2015， 36(12): 1337-1340.
[36] 苏欣，高峰，宋爱军，等. 廊坊市艾滋病高危人群综合干预方法与效果评价[J]. 职业与健康， 2015， 31(14): 1949-1951.
[37] 赵文彬，苏秋菊，姜春红. 2014年鞍山市艾滋病哨点监测结果分析[J]. 中国卫生统计， 2015， 32(06): 929-931.
[38] 孙笠翔，周丹，姜凤霞，等. 2012-2014年辽宁省艾滋病高危人群哨点监测分析[J]. 中华疾病控制杂志， 2015， 19(12): 1227-1230.

[39] 黄鹏翔，廖玫珍，陶小润，等. 2013年山东省艾滋病哨点高危人群性病艾滋病感染状况及行为特点[J]. 预防医学论坛， 2015， 21(06): 415-419.
[40] 马迪辉，张彦琦，周亮，等. 重庆市2006-2012年艾滋病高危人群HIV/AIDS流行情况分析[J]. 第三军医大学学报， 2015， 37(07): 689-692.
[41] 梁容娇，叶兴东，汤少开，等. 2015年广州市STD高危人群性行为特征及艾滋病梅毒感染率的调查结果分析[J]. 皮肤性病诊疗学杂志， 2016， 23(05): 341-345.
[42] 汤雪琴，韩春. 2012年南昌市高危人群艾滋病哨点监测结果分析[J]. 首都公共卫生， 2016， 10(03): 133-136.
[43] 何婉苹，叶兴东，汤少开，等. 2014年广州市三类STD高危人群梅毒、HIV检测调查结果分析[J]. 中国皮肤性病学杂志， 2016， 30(03): 300-303.
[44] 杨洪，苏玲，叶黎，等. 四川省2011-2015年4类高危人群HIV-1新发感染率及流行趋势分析[J]. 中华流行病学杂志， 2017， 38(12): 1649-1654.
[45] 胡婷，董丽芳，贾华，等. 2015年陕西省艾滋病高危人群哨点监测结果分析[J]. 中国艾滋病性病， 2017， 23(10): 905-908.
[46] 李翠媚. 肇庆市2014～2016年HIV高危行为人群干预情况分析[J]. 安徽预防医学杂志， 2019， 25(04): 283-285.
[47] 李芬，马树波，刘红新. 2011年北京市昌平区4类人群艾滋病监测结果分析[J]. 中国健康教育， 2012， 28(06): 469-471.
[48] 唐梦瑾，钟静，钟福华. 艾滋病在不同类型人群性途径传播关系的研究[J]. 实用预防医学， 2012， 19(06): 956-958.
[49] 郭燕，董笑月，柏建芸，等. 天津市2005-2009年部分人群HIV监测分析[J]. 中国预防医学杂志， 2012， 13(02): 133-135.
[50] 姜杰，郭琦，韩焱，等. 大连市2010-2012年不同人群HIV、梅毒、丙型肝炎感染状况分析[J]. 疾病监测与控制， 2013， 7(12): 718-720.
[51] 严钏元，田辉，王红林，等. 2012年宝鸡市6类人群艾滋病哨点监测结果分析[J]. 中国健康教育， 2013， 29(07): 623-626.
[52] 景雪源，蒋洪林，杨芳，等. 湖北省不同人群梅毒预防知识知晓情况分析[J]. 中国艾滋病性病， 2013， 19(03): 201-203.
[53] 蒋均，张文君，郭志宏，等. 中国部分HIV高危人群HPV感染现状及影响因素的Meta分析[J]. 中国艾滋病性病， 2014， 20(11): 818-822.
[54] 王卓. 2013年达州市6类人群艾滋病哨点监测结果分析[J]. 中国预防医学杂志， 2015， 16(04): 300-303.
[55] 吴忠兰，赵伟明，王雪敏，等. 2009-2013年宁夏九类人群艾滋病、梅毒和丙肝哨点监测结果分析[J]. 宁夏医学杂志， 2015， 37(08): 708-710.
[56] 杨莉，杨朝军，蒋岩，等. 云南省吸毒人群和男男性行为人群HIV-1新发感染调查[J]. 中国皮肤性病学杂志， 2015， 29(08): 817-819.
[57] 伍昆山，吴燕珍，刘小凤，等. 珠海市高危男性人群HIV、TP与HCV感染情况分析[J]. 检验医学与临床， 2015， 12(15): 2172-2174.
[58] 赵荣江，牛雅娟，杨少杰，等. 中国北方城乡两地居民对10种特殊人群社会距离及社交限制的调查[J]. 中国神经精神疾病杂志， 2015， 41(07): 395-400.
[59] 张科，韩磊，陶小润，等. MSM“知行分离”现象成因初探[J]. 中国艾滋病性病， 2015， 21(05): 404-408.
[60] 熊素平，陈凯，刘娜，等. 天津某区特殊人群HIV感染者合并HBV、HCV、HGV和TB感染的调查分析[J]. 中华实验和临床病毒学杂志， 2015， 29(01): 47-49.
[61] 赵培祯，黄澍杰，刘凤英，等. 广东省梅毒综合防治示范区梅毒防治工作现况专项调查[J]. 中国艾滋病性病， 2016， 22(06): 451-454.
[62] 彭庭海，彭国平，阳凯，等. 2010—2015年湖北省男男性行为者艾滋病新发感染流行病学特征分析[J]. 公共卫生与预防医学， 2017， 28(05): 49-52.
[63] 多吉旺姆，卢姗，雅西，等. 西藏自治区2015年暗娼等7类艾滋病重点人群HIV、梅毒和HCV横断面调查[J]. 中华流行病学杂志， 2017， 38(07): 921-925.
[64] 熊素平，陈凯，刘娜. 天津某区特殊人群HIV感染情况分析[J]. 长治医学院学报， 2017， 31(01): 29-32.
[65] 陈伟思，黎华波，张择榕，等. 三亚市不同人群梅毒感染现状分析[J]. 中国性科学， 2018， 27(07): 142-144.
[66] 曹琳，罗莉，胡荣，等. 2013—2016年武汉市HIV/AIDS阳性检测分析[J]. 公共卫生与预防医学， 2018， 29(01): 109-111.
[67] 张禧彦. 2016年驻马店市艾滋病哨点人群HIV、梅毒及HCV监测结果分析[J]. 河南预防医学杂志， 2018， 29(06): 460-462.
[68] 张英霞. 2007-2008年柳州市高危人群HIV/梅毒感染的流行病学研究[D]. 中南大学，2010.
[69] 宋文丰. 基于WebGIS西部地区HIV高危人群暴露前用药的信息管理系统[D]. 重庆医科大学，2011.
[70] 汪雁鹤，汪宁，张北川，等. 5市男男性接触者状况及影响其高危行为的因素[J]. 医学与社会， 2005(09): 1-2.
[71] 许娟，张洪波，郑迎军，等. 男男性接触者活动场所及人群特征[J]. 中国公共卫生管理， 2007(04): 389-391.

[72] 张洪波，吴尊友，徐杰，等. 中国既往男男性行为人群艾滋病干预模式[J]. 中国艾滋病性病， 2009， 15(01): 29-32.
[73] 李江涛. 白银市男男性接触者艾滋病健康教育和干预效果评价[J]. 卫生职业教育， 2009， 27(03): 120-121.
[74] 郝树伟，葛凤琴，蒋宁，等. 中国城市男男性行为者娱乐场所性病艾滋病干预现状调查[J]. 中国性科学， 2012， 21(07): 81-84.
[75] 王梅. 湖北省男男性行为人群艾滋病防治效果评估体系的建立[D]. 武汉科技大学，2009.
[76] 薛珲. 重庆市主城区不同场所男男性接触人群艾滋病干预的工作内容及实际支出成本研究[D]. 中国疾病预防控制中心，2011.
[77] 段卫芳. 情绪管理在MSM艾滋病患者中的应用探究[D]. 兰州大学，2016.
[78] 陈世海，周吉，朱建靖. 广西男男性接触者行为学特征分析[J]. 应用预防医学， 2007(06): 341-343.
[79] 罗士德，鞠鹏. 云南同性恋艾滋病防治现状及策略[J]. 河南中医学院学报， 2006， 21(06): 3-5.
[80] 丁贤彬，卢戎戎，徐静，等. 104名感染艾滋病病毒的男男性接触者性行为特征分析[J]. 现代预防医学， 2008(20): 3996-3998.
[81] 丁贤彬，张维，冯连贵，等. 重庆市艾滋病流行形势分析与防治策略研究[J]. 热带医学杂志， 2011， 11(03): 334-336.
[82] 郑毓芳，潘承恩，张仁芳，等. 上海市男男同性性传播AIDS病人的临床流行病学研究[J]. 中国艾滋病性病， 2011， 17(05): 523-525.
[83] 黑发欣，王璐，秦倩倩，等. 中国2006—2010年男男性行为者艾滋病疫情分析[J]. 中华流行病学杂志， 2012， 33(01): 67-70.
[84] 郭莹，夏炜，王潇，等. 男同性恋HIV感染者牙周情况分析[J]. 北京医学， 2012， 34(12): 1063-1064.
[85] 黄惋莉，赖文红，王秋实，等. 2014-2018年四川省预防艾滋病经性传播干预实施情况分析[J]. 预防医学情报杂志， 2020， 36(04): 457-461.
[86] 李力，罗巍，徐杰，等. 2014-2018年全国艾滋病高危人群干预数据质量评估分析[J]. 中华流行病学杂志， 2020(01): 85-89.
[87] 赵政，高永军，程绍辉，等. 同伴咨询对HIV初筛阳性者确证检测的促进[J]. 中国艾滋病性病， 2013， 19(03): 223.
[88] 周斌. HIV快速检测在MSM人群现场监测中的运用[J]. 中国艾滋病性病， 2013， 19(03): 216.
[89] 白广义，王伟，赵宏儒，等. 河北省2004-2013年性传播艾滋病男性患者流行特征分析[J]. 中华疾病控制杂志， 2014， 18(12): 1170-1172.
[90] 龚苍涛，王增强，贺兴增. 泉州市男男性行为人群艾滋病流行状态分析[J]. 海峡预防医学杂志， 2014， 20(1): 33-34.
[91] 王宵雪，段灵芝，裴京明，等. 北京市西城区2011-2015年诊断的HIV感染人群的特征和流行特点[J]. 中国艾滋病性病， 2016， 22(08): 654-655.
[92] 杨兴光，张娜，郝连正，等. 2007-2016年山东经不同性途径男性HIV感染者和艾滋病患者流行病学特征分析[J]. 中华预防医学杂志， 2018， 52(03): 292-295.
[93] 陈志胜，杨晶，玛吾兰·阿不都哈帕尔，等. 伊犁州直男男性行为人群中HIV感染者的特征及影响因素分析[J]. 疾病预防控制通报， 2018， 33(02): 45-47.
[94] 罗亚莉，曾甜甜. 2018年兰州市城关区艾滋病哨点监测数据分析[J]. 疾病预防控制通报， 2019， 34(04): 47-49.
[95] 燕虹，左丹，李十月，等. 女大学生同性恋行为及影响因素分析[J]. 中国公共卫生， 2008， 24(11): 1283-1284.
[96] 李凯英，杨桃，莫玉芬，等. 男同性恋者婚内ED的调查[J]. 中国性科学， 2008， 17(03): 21-22.
[97] 林可，高燕宁，陈远方. 中国同性恋网站服务功能与男男性接触者健康教育研究[J]. 中国健康教育， 2008， 24(03): 179-181.
[98] 朱军礼，张洪波，吴尊友，等. 多部门参与的MSM人群艾滋病预防控制模式的研究[J]. 中国艾滋病性病， 2008， 14(06): 558-561.
[99] 王毅，张洪波，徐杰，等. CDC主导与志愿者实施的MSM人群艾滋病防治模式探索[J]. 职业与健康， 2009， 25(22): 2446-2447.
[100] 蔡于茂，洪福昌，潘鹏，等. 深圳市男男性行为者艾滋病性病关怀和支持服务模式[J]. 中国热带医学， 2012， 12(08): 937-939.
[101] 许骏，刘聪，杨连第，等. 社会组织参与艾滋病病例发现与管理的实践及探讨[J]. 公共卫生与预防医学， 2012， 23(04): 49-52.
[102] 伊赫亚，辛美哲. 中盖艾滋病项目对广州市男男性接触人群社区组织干预检测能力的影响[J]. 中国预防医学杂志， 2012， 13(05): 344-346.
[103] 程绍辉，高永军，朱效科，等. 中盖项目实施对天津地区艾滋病发现与管理的效果评估[J]. 中国艾滋病性病， 2012， 18(03): 193-194.
[104] 杨家芳，胡安艳，赵彩佐，等. 男男性行为人群艾滋病综合干预模式探索[J]. 皮肤病与性病， 2013， 35(06): 360-361.
[105] 曹宁校，葛凤琴，蒋宁，等. 我国MSM人群聚集的酒吧和浴池的状况调查[J]. 中国艾滋病性病， 2013， 19(01): 64-67.

[106] 曹宁校，卜鹏滨，梁慧婷，等. 男男性接触人群性病就诊服务促进研究[J]. 中国预防医学杂志， 2013， 14(08): 636-638.
[107] 曹宁校，李士樑，葛凤琴，等. 男性性病诊疗服务体系的完善及规范化服务[J]. 中国艾滋病性病， 2013， 19(07): 526-529.
[108] 徐祖辉，李峰，王妮，等. 中盖项目对7城市NGO参与MSM HIV检测的影响[J]. 中国艾滋病性病， 2013， 19(06): 406-408.
[109] 张大鹏，程峰，明中强，等. 以结果为导向的绩效管理机制及其在与社区组织合作过程中的应用[J]. 中国艾滋病性病， 2013， 19(09): 689-693.
[110] 李希光，顾小琛，景军，等. 中国MSM人群艾滋病传播的网络干预[J]. 中国艾滋病性病， 2013， 19(08): 599-601.
[111] 梁国民，罗辉琴，陈定仪. 长沙县民间组织在艾滋病病例发现与管理中的实践及探讨[J]. 实用预防医学， 2013， 20(08): 1018-1019.
[112] 张辉，嘎嘎，高永军，等. 天津深蓝工作组参与MSM扩大HIV检测和治疗一体化服务的效果分析[J]. 中国艾滋病性病， 2013， 19(08): 580-582.
[113] 郑迎军，殷文渊，石凯，等. 全球基金艾滋病项目地区MSM的综合干预模式调查[J]. 中国艾滋病性病， 2014， 20(04): 272-274.
[114] 唐慧玲，胡跃强，张涛. 全球基金艾滋病项目对金华市NGO参与MSM的HIV检测工作的影响[J]. 中国艾滋病性病， 2014， 20(09): 687-689.
[115] 付金翠，陈良，马懿，等. 玉溪市城乡男男性接触人群防艾社群组织的建构、发展与挑战[J]. 皮肤病与性病， 2014， 36(04): 225-227.
[116] 杨彦玲，罗红兵，霍俊丽，等. 云南省全球基金艾滋病整合项目2010-2012年实施效果分析[J]. 中华疾病控制杂志， 2014， 18(12): 1129-1131.
[117] 马海玲，黄竹林，赵俊仕，等. 志愿者小组与医疗机构合作提高MSM中性病患者接受规范医疗服务模式的研究[J]. 实用预防医学， 2014， 21(05): 517-519.
[118] 杨杰，赵政，徐杰，等. 中国男同社区小组开展HIV快速检测服务的效果[J]. 中国艾滋病性病， 2014， 20(01): 37-40.
[119] 席胜军，周晓红，吕鑫，等. 社区HIV快速检测在扩大MSM检测中的效果评价[J]. 中国卫生检验杂志， 2015， 25(01): 39-41.
[120] 郑迎军，殷文渊，石凯，等. 中国全球基金艾滋病项目对MSM社区组织发展的影响[J]. 中华疾病控制杂志， 2015， 19(12): 1265-1268.
[121] 刘静，蔡纪明，王芃，等. 社区组织参与的男男性行为人群干预动员检测咨询及治疗关怀的一体化服务模式实施效果分析[J]. 中国预防医学杂志， 2015， 16(6): 471-473.
[122] 付晓静，单多，齐金蕾，等. 中国三个城市男男性行为人群艾滋病防治社区组织生存发展状况分析[J]. 中华预防医学杂志， 2015， 49(06): 518-523.
[123] 赵月娟，王娟，卢红艳，等. 北京市疾病预防控制中心VCT门诊点MSM的HIV检测和导医情况[J]. 中国艾滋病性病， 2015， 21(04): 325-328.
[124] 张国磊，梁欣，张夏燕，等. 依托社区小组在男男性行为者中开展HIV抗体快速检测的效果分析[J]. 中国艾滋病性病， 2016， 22(08): 645-647.
[125] 吴琼苗，程伟彬，钟斐，等. 男男性行为者艾滋病自检模式——岭南准初探[J]. 中国艾滋病性病， 2016， 22(05): 391-393.
[126] 王云霞，孙磊，高翔. 乌鲁木齐市在男男性行为者中开展尿液传递检测工作初探[J]. 中国艾滋病性病， 2016， 22(12): 1011-1013.
[127] 李雪静，路玉平，宋媛媛. 2016年长春市男男性行为人群检测促进项目效果分析[J]. 首都公共卫生， 2017， 11(02): 58-60.
[128] 王月华，程焕义，马仲慧，等. 社区组织参与男男性行为者的HIV快速检测质量评估[J]. 中国艾滋病性病， 2018， 24(11): 1147-1150.
[129] 杜玉华，李雪静，路玉平，等. 长春市某社区组织检测MSM人群HIV感染情况及行为分析[J]. 海峡预防医学杂志， 2018， 24(04): 40-42.
[130] 张广，王芃，李锦峰，等. 社会组织参与男男性行为者艾滋病防治工作评价[J]. 中国艾滋病性病， 2019， 25(01): 67-69.
[131] 李翔，邢爱华，常文辉，等. 2003-2008年西安男男同性性行为者艾滋病监测分析[J]. 中华疾病控制杂志， 2009， 13(04): 442-444.
[132] 程绍辉，郑辉，聂晓璞，等. 在社区开展自愿咨询检测提高HIV检测和随访的可及性分析[J]. 中国预防医学杂志， 2010， 11(12): 1207-1209.
[133] 黄凤荣，黄红，庄鸣华，等. 上海市52名青少年男同性恋者社会心理和性行为状况调查[J]. 上海交通大学学报(医学版)， 2010， 30(05): 581-584.
[134] 黄凤荣，庄鸣华，方蕙，等. 上海市青少年男男性接触者性行为安全认知和社会状况调查[J]. 环境与职业医学， 2010， 27(05): 284-287.
[135] 夏宏丽，朱平，袁建明. 南通市2010年艾滋病自愿咨询检测相关情况分析[J]. 医学动物防制， 2011， 27(03): 278-280.
[136] 李晋，刘哲. 大学生同性性倾向者的现状调查与分析[J]. 中国性科学， 2014， 23(12): 89-93.
[137] 王雷，齐亚莉，赵大力，等. 吉林省在校大学生男男同性性行为人群性行为影响因素及应对策略[J]. 中国预防医学杂志， 2015， 16(06): 499-500.
[138] 滕姗，赵久波，张小远，等. 大学生性取向变化及其与自杀风险关系的2年随访研究[J]. 中国心理卫生杂志， 2018， 32(07): 589-593.

[139] 张宇迪，付芳. 基于全国样本的性少数大学生心理健康状况的调查[J]. 中国临床心理学杂志， 2019， 27(05): 997-1002.
[140] Chevry 克洛伊 Chloee Bazin. 南京男性大学生HIV感染者性行为特征研究[D]. 东南大学，2019.
[141] 易柳. 同性恋者身份认同、对社会态度的感知与心理健康的关系研究[D]. 广州大学，2011.
[142] 戴欣. 重庆地区MSM人群EB病毒感染血清流行病学调查研究[D]. 重庆医科大学，2015.
[143] 胡馨予. 性少数群体社会支持网络构建研究[D]. 西北大学，2018.
[144] 方坤. 南京高校学生男男性行为人群性行为特征研究[D]. 东南大学，2018.
[145] 卞策. 男性同性恋焦虑、抑郁倾向与社会支持及其关系研究[D]. 首都经济贸易大学，2018.
[146] 张国磊，张夏燕，梁欣. 在MSM中开展基于药店的HIV抗体尿液匿名传递检测结果分析[J]. 中国艾滋病性病， 2016， 22(10): 810-812.
[147] 李建伟，孟毓，王静，等. 新乡地区无偿献血人群中MSM人群感染HIV特征分析[J]. 河南医学研究， 2016， 25(09): 1580-1582.
[148] 冯娟. 安阳地区无偿献血者及其中男男性行为人群HIV感染情况调查分析[J]. 安徽医学， 2018， 39(02): 170-173.
[149] 周亚静，王爱红. 男男同性恋艾滋病患者主要照顾者负担现状及影响因素调查[J]. 齐鲁护理杂志， 2016， 22(05): 56-58.
[150] 梁军，张婉筠，廖斌，等. 医院肛肠专科针对男男性行为者提供艾滋病及性病检测和转介服务的探索[J]. 中国艾滋病性病， 2019， 25(02): 172-174.
[151] 邓艳红，王明，李红卫，等. 艾滋病与男同性恋[J]. 中国健康教育， 2005， 21(02): 19-22.
[152] 何群，王晔. 男性同性恋、男性同性性行为与艾滋病[J]. 华南预防医学， 2005， 31(05): 20-22.
[153] 张连舫，贺连生. 女性同性恋导致抑郁状态杀人未遂1例报告[J]. 辽宁医学杂志， 2005， 19(04): 198.
[154] 张晓燕，王晨，李新旭，等. 北京市MSM人群HIV感染者合并HCV与梅毒感染的状况及相应免疫指标与病毒载量的变化[J]. 中国艾滋病性病， 2006(04): 294-296.
[155] 木合塔，张峰，李凡. 乌鲁木齐市男男同性恋中首例HIV感染者报告[J]. 中国艾滋病性病， 2006， 12(04): 362.
[156] 唐力，王夏，刘满清，等. 武汉市145例男男性接触者的相关血清学监测及分析[J]. 疾病监测， 2006， 21(12): 625-627.
[157] 蔡新. 2006年聊城市部分男男性接触者高危行为及血清学调查[J]. 预防医学论坛， 2007， 13(10): 888-890.
[158] 郭晓黎，乔晓春，原琛利，等. 山西省首例经MSM感染HIV的报告[J]. 中国艾滋病性病， 2007， 13(06): 528.
[159] 孟爱群，宁秀君，牛惠敏，等. MSM人群HIV及性传播感染和HIV亚型分布研究[J]. 医学动物防制， 2007， 23(07): 522-524.
[160] 丁训奇，陆洪光. 男男性接触HIV感染者并发梅毒和尖锐湿疣1例[J]. 贵州医药， 2008， 32(11): 977.
[161] 黄淑平，王召乾，冯福. 海南省首例男男性行为HIV感染者个案分析[J]. 中国热带医学， 2008(11): 2019.
[162] 崔明宇，孙怡，赵克宁，等. 男男性接触人群梅毒患者54例临床分析[J]. 中国皮肤性病学杂志， 2008(06): 363-364.
[163] 王去非，朱英华. 男男性接触者早期梅毒22例临床分析[J]. 中国皮肤性病学杂志， 2008(05): 294-295.
[164] 马伟元，彭韬，赵克宁. 男男性接触人群口腔淋病32例临床分析[J]. 中国麻风皮肤病杂志， 2010， 26(05): 374-375.
[165] 于茂河，郑敏娜，左桐，等. 天津市男男性行为人群中HIV-1阳性者的行为特征与基因亚型分析[J]. 中国艾滋病性病， 2011， 17(01): 32-34.
[166] 刘海燕，岳冀，李曦，等. 51例感染HIV的男性同性恋者传染病情况分析[J]. 中国医药指南， 2012， 10(35): 54-56.
[167] 徐萌，绳波，寇卜心，等. 北京市男男性行为人群庚型肝炎病毒感染率和流行亚型分析[J]. 中华流行病学杂志， 2013， 34(07): 757-758.
[168] 魏宏屹，徐俊杰，胡清海，等. HIV阳性MSMHSV-2感染及危险因素调查[J]. 中国公共卫生， 2014， 30(3): 357-358.
[169] 颜苹苹，张春阳，梁菲菲，等. 福建省3市男男性行为者HIV-1感染率与新发感染率初探[J]. 海峡预防医学杂志， 2014， 20(04): 1-2.
[170] 刘莹，刘小敏，舒彬，等. 深圳市男男同性恋人群艾滋病疾病进程及影响因素[J]. 公共卫生与预防医学， 2015， 26(02): 66-69.
[171] 曹栋卿，何婷婷，赵丹燕，等. 浙江省绍兴市46例男男性行为者HIV感染基因特征分析及流行病相关因素分析[J]. 疾病监测， 2015， 30(10): 841-845.

[172] 周宁，柏建芸，郭燕，等. 天津市115名有男男性行为的HIV阳性无偿献血者情况分析[J]. 中国艾滋病性病， 2016， 22(10): 828-830.
[173] 于茂河，徐鹏，赵璇，等. 天津市2013－2015年MSM HIV感染者病毒载量抽样调查[J]. 中华医院感染学杂志， 2016， 26(18): 4116-4118.
[174] 斗智，江震，潘品良，等. 参比校正对中国15个大城市MSM人群HIV感染者病毒载量抽样调查结果的影响[J]. 中华流行病学杂志， 2017， 38(12): 1683-1687.
[175] 鲍燕，宁镇，顾凯侃，等. 静安区男男性行为者中新发现的HIV感染者首次CD4~+T淋巴细胞检测情况[J]. 中国艾滋病性病， 2018， 24(10): 1016-1019.
[176] 高鸣，李永红，李凌华，等. HIV阳性的男男性行为者肛门人乳头瘤病毒感染现状分析[J]. 热带医学杂志， 2019， 19(06): 785-788.
[177] 宋丹. 郑州地区男男同性恋人群HIV-1感染者基因亚型及耐药分析[D]. 郑州大学，2012.
[178] 赵彬. 辽宁省MSM人群HIV感染者基因型耐药以及与流行毒株相关的社会网络特征研究[D]. 中国医科大学，2012.
[179] 杨蒙蒙. 江苏省男男性行为人群HIV获得性和传播性耐药流行情况研究[D]. 南京医科大学，2016.
[180] 陈力. 男男性行为者巨细胞病毒感染的血清流行病学调查[D]. 重庆医科大学，2016.
[181] 林倚伊. MSM人群无保护性行为轨迹特征及PrEP性行为去抑制化研究[D]. 重庆医科大学，2016.
[182] 田雅茹. 男男性接触HIV感染人群疾病进展过程中HBV合并感染的情况研究[D]. 首都医科大学，2016.
[183] 杨荷丹. 中国3城市男男性行为者不同解剖部位人乳头瘤病毒感染状况及分子流行病学研究[D]. 北京协和医学院，2019.
[184] 刘明华，张北川，李秀芳. 对男男性接触人群社会学因素及相关因素研究[J]. 中国艾滋病性病， 2006， 12(03): 285-287.
[185] 郭剑，马骏. 网络调查在同性恋人群研究中的应用[J]. 预防医学情报杂志， 2006(04): 498-499.
[186] 宫德萍，姬照伟，王桂玲. 莱阳市男性同性恋人群行为调查[J]. 职业与健康， 2006， 22(17): 1363-1364.
[187] 栗晓红. 女同性恋性别认同和身份认同研究[J]. 中国性科学， 2007， 16(05): 6-9.
[188] 李慧，郑迎军，吴迪，等. 高危人群安全套需求状况调查[J]. 中国艾滋病性病， 2009， 15(03): 254-256.
[189] 郑丽军，郑涌. 中国同性恋性别角色异性化倾向调查[J]. 中国公共卫生， 2009， 25(04): 499-500.
[190] 蔡玲. 女农民工中的拉拉:游离于城市边缘的特殊群体[J]. 中国性科学， 2009(9): 9-11.
[191] 闫红梅，赵东辉，罗佳伦，等. 男男性行为人群安全套使用促进策略研究[J]. 中国当代医药， 2009， 16(13): 51-52.
[192] 李新旭，李东亮，阮玉华，等. 不同方法招募的男男性接触者高危性行为及HIV和梅毒感染的比较[J]. 现代预防医学， 2007， 34(07): 1224-1227.
[193] 柳昭羽，吴国辉，徐杰，等. 男男性行为人群队列保持的影响因素研究[J]. 中国艾滋病性病， 2011， 17(06): 646-650.
[194] 张曼，王云霞，任晖琴，等. 乌鲁木齐市男男性行为人群随访队列保持的调查[J]. 职业与健康， 2013， 29(18): 2368-2369.
[195] 王毅，张光贵，李六林，等. 绵阳市男男性行为人群随访队列保持及影响因素研究[J]. 预防医学情报杂志， 2013， 29(01): 5-11.
[196] 张燕，钟晓妮，彭斌，等. 男男性行为人群艾滋病暴露前预防用药临床试验队列保持力及预测因子分析[J]. 第三军医大学学报， 2013， 35(22): 2469-2472.
[197] 周楚，徐杰，斗智，等. 男男性行为人群队列失访情况及影响因素分析[J]. 中华流行病学杂志， 2013， 34(08): 788-791.
[198] 周建波，甄森，王金塔，等. 男男性行为人群队列维持影响因素调查[J]. 中国公共卫生， 2013， 29(08): 1162-1165.
[199] 付春静，钟晓妮，彭斌，等. 男男性行为人群艾滋病暴露前预防用药临床试验队列保持力分析[J]. 重庆医科大学学报， 2014， 39(11): 1573-1577.
[200] 金玫华，杨中荣，董正全，等. 通过互联网招募MSM的HIV新发感染及队列保持影响因素分析[J]. 疾病监测， 2016， 31(04): 288-292.
[201] 陈楚莹，李雷，王曼，等. 中山市男男性行为者前瞻性队列随访影响因素研究[J]. 中国艾滋病性病， 2016， 22(02): 102-105.
[202] 牟雨婵，徐嘉悦，马原林，等. MSM艾滋病暴露前预防用药临床试验队列保持影响因素分析[J]. 现代预防医学， 2017， 44(16): 2886-2889.
[203] 杨银梅，李十月，燕虹. 男男性行为者队列保持率及影响因素的Meta分析[J]. 中国艾滋病性病， 2017， 23(05): 429-432.
[204] 郭燕，周宁，杨杰，等. 天津市2012—2015年浴池男男性行为人群队列失访影响因素分析[J]. 中华流行病学杂志， 2017， 38(02): 185-188.
[205] 李辉，张北川，李秀芳. 社会学因素对同性爱者影响的研究进展[J]. 中国性科学， 2007， 16(04): 39-42.

[206] 刘华清，陈彦芳. 同性恋生物社会心理因素的研究[J]. 中国性科学， 2008， 17(08): 38-40.
[207] 李智. 中国同性恋研究综述[J]. 黑河学院学报， 2012， 3(05): 119-121.
[208] 张椰，李现红，鄢芳. 基于共词分析的男同性恋人群艾滋病防治领域研究热点[J]. 中国预防医学杂志， 2018， 19(11): 801-807.
[209] 胡少华，许毅，李鹏，等. 男性同性恋与男性异性恋对厌恶情绪调节模式的差异[J]. 浙江大学学报(医学版)， 2008， 37(05): 487-493.
[210] 王典，谢庆东，于晓军，等. 男同性恋人类免疫缺陷病毒感染者精液质量分析[J]. 汕头大学医学院学报， 2011， 24(01): 1-2.
[211] 李钊，汪永忠，王仲宁，等. 昆明市男男性行为者HIV感染490例分析[J]. 昆明医学院学报， 2010， 31(10): 127-129.
[212] 王亮，刘中夫，曹卫华. 某市以检测为主要干预手段的男男性接触人群艾滋病综合干预效果评价[J]. 中国初级卫生保健， 2011， 25(08): 94-95.
[213] 韩雪，张晓萍，李晶，等. 银川市2010年男男性行为者高危行为及性传播疾病调查分析[J]. 宁夏医学杂志， 2012， 34(01): 69-70.
[214] 房明，赵文彬，尹晔. 2010—2011年鞍山市男男性行为人群安全套使用率及影响因素[J]. 中国冶金工业医学杂志， 2012， 29(04): 434-435.
[215] 成振晓，刘媛媛，王永香，等. 2010年山东省男男性行为者艾滋病知识知晓情况及其影响因素分析[J]. 预防医学论坛， 2012， 18(05): 329-331.
[216] 陈军仙，阮建军，朱碧香，等. 男男性行为人群88例HIV阳性病例分析[J]. 浙江预防医学， 2012， 24(10): 18-19.
[217] 马海燕，于海荣，苏大禹，等. 男男性行为人群获得性免疫缺陷综合征知识知晓情况调查[J]. 社区医学杂志， 2012， 10(17): 47-48.
[218] 陈树昶，罗艳，丁建明. 2004-2010年浙江省杭州市男男性接触者艾滋病病毒/艾滋病感染病例流行病学特征分析[J]. 疾病监测， 2012， 27(08): 637-639.
[219] 陈美芬，赵建佳，裘立晓. 男男同性恋艾滋病综合调查分析[J]. 中国卫生检验杂志， 2013， 23(01): 239-240.
[220] 许圆圆，丁盈盈，吴琼海，等. 台州地区男男性行为人群艾滋病疫情调查[J]. 上海预防医学， 2014， 26(04): 184-186.
[221] 江光煛. 襄阳市男男同性恋艾滋病阳性病例研究[J]. 热带医学杂志， 2014， 14(03): 389-391.
[222] 杨兴光，张娜，郝连正，等. 2003—2013年山东省男性同性传播艾滋病流行特征分析[J]. 中华预防医学杂志， 2015， 49(02): 181-183.
[223] 朱晨，华燕，陈鑫，等. 253名男男性行为人群HIV/梅毒监测资料分析[J]. 航空航天医学杂志， 2015， 26(07): 882-883.
[224] 范吉祥，姜雪敏. 吉林省MSM行为特征及HIV感染影响因素分析[J]. 中国卫生工程学， 2015， 14(06): 580-581.
[225] 张海东，祁玲，邢永杰. 青海省格尔木市MSM中HIV感染者特征分析[J]. 青海医药杂志， 2015， 45(01): 21-22.
[226] 王東秀，景正朝. 云南蒙自41例HIV/AIDS男男性行为人群分析[J]. 皮肤病与性病， 2015， 37(02): 88-89.
[227] 李一苇，霍炜，侯凤英，等. 菏泽市46例HIV阳性的男男性行为者分析[J]. 中国艾滋病性病， 2016， 22(01): 13-14.
[228] 李倩，贾丽君，于立芬，等. 西安市2010－2014年HIV阳性MSM的流行病学特征[J]. 中国艾滋病性病， 2016(3): 173-175, 193.
[229] 石蕾，卢园，张春阳，等. 福州市2013-2014年HIV-1新发感染情况及同性传播人群流行病学特征分析[J]. 实用预防医学， 2016， 23(07): 849-851.
[230] 岑平，农全兴，徐永芳，等. 南宁市男男同性性行为艾滋病的流行现状与干预对策研究[J]. 现代预防医学， 2016， 43(11): 1928-1931.
[231] 邱理杨，雷永良，李彦林，等. 2011年-2015年丽水市男男性行为人群艾滋病传播风险分析[J]. 中国卫生检验杂志， 2016， 26(09): 1303-1304.
[232] 马龙，胡改霞，蔺淞锋，等. 铜川市91例男男性行为者HIV/AIDS病例的流行病学分析[J]. 中国热带医学， 2017， 17(11): 1160-1162.
[233] 杨玲，谢远俊. 男同性恋者自我认同发展的质性研究[J]. 中国性科学， 2011， 20(05): 42-45.
[234] 王中杰，冯成亮，耿耀国. 男同性恋大学生性身份认同的定性研究[J]. 中国心理卫生杂志， 2012， 26(08): 620-625.
[235] 朱云武. 大学生同性恋心理状况的质化研究[J]. 校园心理， 2013， 11(05): 333-335.
[236] 李慧，杨裕华，屠爱霞. 同性恋者及其性取向的生物学研究进展[J]. 中国性科学， 2013， 22(11): 97-100.
[237] 王晴锋. 男同性恋者的性角色与性别角色[J]. 中国性科学， 2013， 22(10): 102-107.
[238] 于方舟，刘盈. 同性恋[J]. 中国实用乡村医生杂志， 2013(3): 30-32.
[239] 史俊鹏，叶蕤. 告别“默认的异性恋者”——男女同志身份认同研究[J]. 中国性科学， 2015， 24(07): 123-128.

[240] 张静，郑丽军，郑涌. 性少数人群的心理健康:理论模型与研究取向[J]. 心理科学进展， 2015， 23(06): 1021-1030.
[241] 冯一冰，步凯，张夏燕，等. 男男性行为发生的影响因素研究进展[J]. 中华流行病学杂志， 2015， 36(05): 535-538.
[242] 王丽圆. 社会工作视角下男同性恋大学生自我认同研究[J]. 中国性科学， 2016， 25(09): 139-143.
[243] 李放，王一博，邢锦涛，等. 同性恋者的内化同性恋嫌恶:形成及影响机制[J]. 心理科学， 2018， 41(06): 1470-1476.
[244] 张杰. 双性恋的历史、现实与将来[J]. 中国性科学， 2018， 27(02): 156-160.
[245] 刘颋颋. 同性恋者性身份认同的影响因素研究[D]. 华东师范大学，2008.
[246] 王誉儒. 青年男同性恋者性观念个案调查研究[D]. 四川师范大学，2010.
[247] 孙建. 大学生同性恋者性爱观念研究[D]. 四川师范大学，2010.
[248] 崔玉. 同性恋大学生的现状及思想教育方法研究[D]. 西南交通大学，2011.
[249] 胡群琼. 认同的挣扎[D]. 陕西师范大学，2011.
[250] 李博. 以社会认同视角分析中国同性恋亚性别群体生活处境现状[D]. 东北师范大学，2013.
[251] 王冬. 双性恋者的身份认同：一项深度访谈研究[D]. 南京师范大学，2013.
[252] 张培斐. 同性恋身份认同研究[D]. 西南大学，2014.
[253] 吴维. 隐匿与显现：J市男同性恋者社会互动研究[D]. 浙江师范大学，2015.
[254] 袁兆宇. 艾影如虹：未染HIV青年男同社区中的艾滋病意象[D]. 南开大学，2015.
[255] 宋思. 社会化媒体下的男同性恋身份认同研究[D]. 暨南大学，2016.
[256] 陈琪. 女同性恋家庭接纳的个案介入[D]. 中南民族大学，2016.
[257] 宋文洁. 男同性恋的身份认同对其婚育状况的影响分析[D]. 山东大学，2017.
[258] 陈磊，刘婷，徐海港，等. 针对男性性接触(MSM)人群艾滋病相关行为干预的IEC信息研究[J]. 现代预防医学， 2011， 38(02): 298-300.
[259] 韩梅，冯连贵，赵金扣，等. 重庆市男男性行为人群2009年HIV-1 BED发病率调查[J]. 中国艾滋病性病， 2011， 17(06): 654-656.
[260] 许一玲，池建民，刘忠诚. 淄博市MSM人群规模及HIV感染调查研究[J]. 中国热带医学， 2010， 10(11): 1322-1324.
[261] 胡培，钟斐，程伟彬，等. 广州市男男性行为者艾滋病感染风险模型的建立[J]. 中华流行病学杂志， 2012， 33(07): 667-671.
[262] 梁莉，周硕，范双凤，等. 2012年四川省试点城市艾滋病相关高危人群规模估计结果[J]. 预防医学情报杂志， 2015， 31(03): 197-200.
[263] 魏艳，杨佳奇，刘玲玲，等. 遵义市汇川区2015年艾滋病高危人群规模及疫情估计[J]. 实用预防医学， 2016， 23(06): 708-768.
[264] 于波，高歌，贺志龙，等. 北京市MSM人群RRT模型的两阶段抽样调查[J]. 中国卫生统计， 2012， 29(03): 351-353.
[265] 薛晓红，张晓萍，韩雪. 银川市男男性行为者规模估计及高危行为特征调查[J]. 中国皮肤性病学杂志， 2013， 27(09): 916-917.
[266] 杨雪燕，伊莎贝拉·阿塔尼，李树茁. 性别失衡与人口流动视角下的男男性行为:来自中国城市地区的证据[J]. 人口与发展， 2013， 19(01): 90-97.
[267] 马桂林，郑惠能，林碧云，等. 利用横断面调查资料估计厦门市男男性接触人群的新发感染率[J]. 海峡预防医学杂志， 2013， 19(05): 25-27.
[268] 何佳桐，钟晓妮，逯嘉，等. 男男性行为人群暴露前预防用药宣传途径的多重对应分析[J]. 第二军医大学学报， 2014， 35(02): 122-128.
[269] 李玲玲，江震，宋炜路，等. 应用德尔菲法构建男男性行为者个体HIV感染风险评估工具[J]. 中华流行病学杂志， 2017， 38(10): 1426-1430.
[270] 林倚伊，钟晓妮，彭斌，等. 基于轨迹分析模型的男男性行为人群无保护性行为特征分析[J]. 重庆医科大学学报， 2017， 42(02): 209-213.
[271] 李世福，董文斌，蔡英，等. 男男性传播与异性性传播HIV新近感染者艾滋病疾病进展比较分析[J]. 现代预防医学， 2018， 45(02): 340-343.
[272] 梁小娇，阮玉华，娄洁. 男男性行为人群中HIV/AIDS行为干预的动力学模型[J]. 上海大学学报(自然科学版)， 2019， 25(06): 898-907.
[273] 陈鹏，李丰，叶清红，等. 捕获-标记-再捕获法估计株洲市男男人群规模及相关行为调查[J]. 预防医学论坛， 2019， 25(05): 334-337.

[274] 黄掣驰，呼志丽，胡清海，等. 大学生MSM人群HIV感染风险预测模型建立及效果验证研究[J]. 实用预防医学， 2020， 27(02): 156-160.
[275] 林丹丹，曾婷，张曼，等. 新疆乌鲁木齐市男男性行为人群HIV传播动力学的预测和分析[J]. 中国感染控制杂志， 2019， 18(05): 388-395.
[276] 陈浩. 应用LMS法估计长沙市城区男男性行为人群规模的研究[D]. 中南大学，2012.
[277] 彭鹏. "暴露前预防" 对男男性行为人群性行为模式和HIV/STIs传播的影响[D]. 南通大学，2017.
[278] 黄掣驰. 大学生MSM人群的HIV自我感染风险诊断模型建立及应用效果初步评估[D]. 中国医科大学，2018.
[279] 肖贵元. MSM人群PrEP服药依从性的预测模型研究[D]. 重庆医科大学，2019.
[280] 陆静萍. 同性恋大学生心理特点及其辅导方法的个案研究[D]. 南京师范大学，2007.
[281] 卡马力提·库尔曼汗. 新疆乌鲁木齐少数民族同性恋族群认同分析[D]. 新疆师范大学，2008.
[282] 刘安，程鸽，宋映雪，等. MSM人群尖锐湿疣合并HIV感染治疗的临床分析[J]. 中国艾滋病性病， 2011， 17(03): 339-340.
[283] 吴晨阳. 男同艾滋病感染者的抗逆力提升研究[D]. 广西师范大学，2017.
[284] 邱英鹏. 深圳市同性恋门诊梅毒干预项目的卫生经济学评价[D]. 中南大学，2013.
[285] 雷章权，罗小莉，陈洪瑶，等. 四川省达州市114例男男性接触者HIV/AIDS病例分析[J]. 中华疾病控制杂志， 2014， 18(12): 1221-1222.
[286] 陈正民，雷永良. 男男性接触人群艾滋病抗体检测报告[J]. 中国卫生检验杂志， 2014， 24(12): 1778-1779.
[287] 卿勇，喻专容，陈竹，等. 64例合并HIV感染男男性行为者肛门直肠尖锐湿疣临床特征及心理评估[J]. 中华实验和临床感染病杂志(电子版)， 2019， 13(02): 117-121.
[288] 于茂河，郭燕，周宁，等. HIV唾液快速检测在MSM浴池中的应用[J]. 中华疾病控制杂志， 2014， 18(12): 1229-1231.
[289] 吕鑫，周晓红，席胜军，等. HIV快速检测在自愿咨询检测门诊男男性接触者中的运用[J]. 浙江预防医学， 2014， 26(09): 932-933.
[290] 徐勤，孙丽，王志军，等. 2014年扬州市MSM中HIV及梅毒检测结果分析[J]. 中国艾滋病性病， 2015， 21(11): 990.
[291] 傅卓华，赵艳秋，赵秀萍，等. 苏州市男男性行为人群艾滋病抗病毒治疗疗效分析[J]. 上海预防医学， 2015， 27(12): 741-744.
[292] 周超，陈宗良，周全华，等. 重庆市男男性行为人群艾滋病患者抗病毒治疗效果分析[J]. 重庆医学， 2015， 44(16): 2205-2207.
[293] 刘燕芬，黄绍标. 广西桂南地区男男性行为人群HIV/AIDS病人抗病毒治疗效果分析[J]. 内科， 2016， 11(03): 389-391.
[294] 郭映武白劲松刘俊王维波何蓉. HAART对男男性行为HIV/AIDS患者疗效观察[J]. 大理大学学报， 2016， 1(4): 80-82.
[295] 董芬，蒋长征，孙灵英，等. 男性行为人群HIV/AIDS 64例抗病毒治疗效果分析[J]. 浙江预防医学， 2016， 28(03): 274-275.
[296] 陈洋，申莉梅，曹文杰，等. 贵州省男男性行为者中的HIV/AIDS病人接受抗病毒治疗后的生存分析[J]. 中国艾滋病性病， 2017， 23(04): 307-309.
[297] 陆珍珍，苏锦明，蓝光华，等. HIV-1阳性的广西男男性行为者启动抗病毒治疗的相关影响因素分析[J]. 中国皮肤性病学杂志， 2017， 31(08): 873-876.
[298] 张静，王丽君，周新，等. 男男性行为者AIDS病人口腔黏膜受损的临床无创干预研究[J]. 中国艾滋病性病， 2018， 24(04): 416-417.
[299] 梁洁，刘文沃，温云油. 男大学生同性恋者"出柜"的辅导报告1例[J]. 中国校医， 2015， 29(09): 649-650.
[300] 郝尹虓，秦倩倩，吴国辉，等. 2004—2015年重庆市经男男性行为感染HIV者空间聚集性及行为分析[J]. 中华预防医学杂志， 2017， 51(05): 432-437.
[301] 张鹤美，王大勇，赵丽娜，等. 温州市2006-2015年男男性行为HIV/AIDS病例时空分布特征[J]. 中国卫生统计， 2018， 35(05): 710-712.
[302] 罗晓媛，李凯，陈成敏，等. 广州市花都区121名男男同性恋艾滋病人群的分布特征[J]. 公共卫生与预防医学， 2018， 29(01): 130-131.
[303] 杨作凯，方益荣，卢巧玲，等. 绍兴市男男性行为人群艾滋病疫情分析[J]. 预防医学， 2018， 30(03): 281-283.
[304] 孙晔. 男男性行为人群HIV检测时间距离分布情况及其影响因素分析[J]. 世界复合医学， 2019， 5(03): 37-39.
[305] 刘惠，刘英，肖亚. 对北京部分男男性接触者HIV/AIDS的KABP调查[J]. 中国艾滋病性病， 2001， 7(05): 289-291.
[306] 张北川，刘殿昌，李秀芳，等. 中国大陆男同性性接触者的艾滋病高危行为及影响因素研究[J]. 中国艾滋病性病， 2001， 7(01): 7-10.

[307] 张北川，李秀芳，史同新，等. 中国大陆男男性接触者艾滋病性病高危险行为情况调查[J]. 中华流行病学杂志， 2001， 22(5).
[308] 张北川，李秀芳，史同新，等. 对男性同性爱人群性病艾滋病高危行为的调查及其干预模式探索[J]. 中国麻风皮肤病杂志， 2002， 18(01): 22-24.
[309] 张北川，胡铁中，李秀芳，等. 中国男同性爱者儿童期状况对自身性别、性取向的态度及认识,少见经历和心理与艾滋病高危行为的关系(一)[J]. 中国性病艾滋病防治， 2002， 8(2): 84-87.
[310] 张北川，胡铁中，李秀芳，等. 中国男同性爱者儿童期状况对自身性别、性取向的态度及认识,少见经历和心理与艾滋病高危行为的关系(二)[J]. 中国性病艾滋病防治， 2002， 8(3): 148-150.
[311] 李秀芳，张北川，史同新，等. 11例感染艾滋病病毒的男男性接触者相关行为调查[J]. 中华流行病学杂志， 2003， 24(09): 101.
[312] 史同新，张北川，李秀芳，等. 对男男性接触者AIDS高危性行为健康干预研究[J]. 中国艾滋病性病， 2003(06): 365-367.
[313] 史同新，张北川，李秀芳，等. 男男性接触途径与艾滋病高危行为关系研究[J]. 中国麻风皮肤病杂志， 2003(03): 230-231.
[314] 史同新，张北川，李秀芳，等. 男男性接触者中STI情况及相关高危性行为调查[J]. 中国麻风皮肤病杂志， 2003(02): 135-136.
[315] 梁平，周华，吴志华. 男男性接触者(MSM)梅毒[J]. 岭南皮肤性病科杂志， 2005(03): 270-272.
[316] 许毅. 男性同性恋的相关问题研究[D]. 浙江大学，2008.
[317] 王琪，侯元，连英姿. 男男同性恋人群艾滋病、性病感染状况研究[J]. 海峡预防医学杂志， 2009， 15(03): 11-12.
[318] 翁毓秋，白玉. 239例男同性恋者性病艾滋病高危行为监测与调查[J]. 应用预防医学， 2009， 15(03): 152-153.
[319] 赵立华，张蕴慧，鱼小红，等. 西部某城市MSM预防艾滋病不同干预来源危险行为的分析[J]. 中国医药指南， 2013， 11(29): 344-345.
[320] 刘广顺，张桐亮，孙淑艳. QQ网络MSM人群艾滋病防治知识、性态度、性行为情况调查[J]. 预防医学论坛， 2014， 20(03): 214-215.
[321] 张根福，吴维. 交往方式与择偶标准——一项基于J市男同性恋者的实证研究[J]. 浙江师范大学学报(社会科学版)， 2014， 39(06): 67-74.
[322] 李林涛，旷翠萍，齐杰，等. 某移民城市男男性行为人群艾滋病相关高危行为特征分析[J]. 中国初级卫生保健， 2014， 28(04): 68-70.
[323] 焦亚芹，回言良. 男男性行为者艾滋病高危行为现状调查[J]. 医学动物防制， 2014， 30(11): 1208-1211.
[324] 张万宏，陈雷，王玫玲，等. 四类场所男性双性性行为者艾滋病、梅毒检测及性行为特征分析[J]. 中国社会医学杂志， 2014， 31(02): 112-114.
[325] 范颂，且亚玲，王海容. 西部地方高校男男性行为人群艾滋病相关行为现况调查[J]. 转化医学电子杂志， 2015， 2(07): 156-158.
[326] 朱勇，贺学俊. 性病门诊就诊者中男男同性恋者HIV检测情况研究[J]. 中国卫生产业， 2017， 14(19): 62-63.
[327] 冯俊林，冯耀辉，杨兰，等. MSM人群HIV感染现状及高危影响因素分析[J]. 中国卫生产业， 2018， 15(13): 132-134.
[328] 王芳，郭玉芳，徐月清，等. HIV阳性男男性行为者的心理弹性状况及相关因素分析[J]. 医学研究与教育， 2019， 36(02): 48-53.
[329] 李清春，陈树昶. MSM人群HIV/STD新发感染及其影响因素研究进展[J]. 中国艾滋病性病， 2010， 16(04): 433-435.
[330] 王硕，吴尊友. MSM中HIV阳性者性行为特征及其影响因素的研究进展[J]. 中国艾滋病性病， 2010， 16(02): 204-206.
[331] 米国栋，吴尊友. 男男性行为人群HIV感染者感染状态配对研究[J]. 中国艾滋病性病， 2010， 16(02): 201-203.
[332] 李建红，陈祥生. 男男性行为人群性病性淋巴肉芽肿的流行状况及其研究进展[J]. 中国艾滋病性病， 2010， 16(01): 89-92.
[333] 史同新，张北川，李秀芳，等. 2000年男男性接触者性传播感染发病状况与高危性行为监测[Z]. 2001201.
[334] 王翠华，杨艳华，卢国际，等. 男男性接触者艾滋病高危行为干预及效果评价[J]. 中国卫生检验杂志， 2007(12): 2291-2292.
[335] 刘荣凯，王文权. 中国男男性接触者现状[J]. 医学动物防制， 2007(10): 797-799.
[336] 蓝光华，刘伟，朱秋映，等. 广西外来男男性接触人群艾滋病监测结果分析[J]. 应用预防医学， 2009， 15(03): 168-170.
[337] 朱晨，陆虹. 259名男男性行为者HIV/梅毒感染监测分析[J]. 江苏预防医学， 2011， 22(06): 34.
[338] 汪伊娜，刘菁萍，徐群英，等. 非政府组织对男男同性恋人群健康教育效果评估[J]. 现代预防医学， 2011， 38(13): 2523-2526.

[339] 孙洁，袁巧菊，杨跃进，等. 男男接触者艾滋病相关知识及高风险性行为方式认知情况调查[J]. 医药论坛杂志， 2011， 32(06): 74-75.
[340] 高媛，文育锋，秦其荣，等. 马鞍山市女性性服务工作者和男男同性恋人群VCT需求调查[J]. 中国农村卫生事业管理， 2011， 31(04): 393-396.
[341] 杜桂英，姜宝法. 聊城市男男性行为大中学生艾滋病知识态度行为及HIV感染状况[J]. 中国学校卫生， 2011， 32(11): 1312-1313.
[342] 周兰，何浩岚. 男-男性接触者梅毒和HIV感染及性行为调查研究[J]. 中国中医药现代远程教育， 2012， 10(07): 133-134.
[343] 黄永清. 南通市通州区男男性行为人员艾滋病流行病学调查分析[J]. 河南预防医学杂志， 2016， 27(11): 812-814.
[344] 农秋苗，李晓霞，张爱平，等. “互联网+”中活跃的MSM人群的艾滋病相关高危行为特征及影响因素研究[J]. 中国初级卫生保健， 2019， 33(08): 86-87.
[345] 杨凭，葛凤琴，韦晓宇，等. 男同性恋及双性恋人群STD/AIDS咨询电话分析[J]. 中国艾滋病性病， 2004， 10(05): 381-382.
[346] 谢延军，皇彦杰. 男同性恋性病患者23例临床分析[J]. 吉林医学， 2005， 26(10): 1037.
[347] 姜少敏，李继钊，李瑞英，等. 威海市男男性接触者不安全性行为及艾滋病相关知识调查[J]. 预防医学论坛， 2006(06): 763-764.
[348] 张敬东，孙烨，王尚博，等. 哈尔滨市经常活动于某浴池的121名MSM艾滋病和梅毒感染情况[J]. 中国艾滋病性病， 2008， 14(01): 69.
[349] 王健，罗超，温迎春. 哈尔滨市部分MSM人群HIV感染及相关危险行为调查[J]. 中国艾滋病性病， 2008， 14(01): 75.
[350] 马骥雄，刘世文，刘志琦. 男男性接触者HIV感染危险因素调查分析[J]. 中国麻风皮肤病杂志， 2008(06): 419.
[351] 杨蓉蓉，桂希恩，熊勇，等. 100例感染HIV的MSM固定性伴间HIV传播特点分析[J]. 中国艾滋病性病， 2011， 17(05): 581.
[352] 张惠荣. 西宁地区男男性行为人群HIV感染状况分析[J]. 现代预防医学， 2012， 39(15): 3907.
[353] 杨春梅，王晓冬，杜树平，等. 成都男男性行为者人群性病艾滋病知识调查[J]. 四川医学， 2005， 26(01): 108-110.
[354] 梁德红，李坚. 民间非营利组织在同性恋群体中健康干预初探——对沈阳市方舟健康干预志愿小组成长的思考[J]. 中国艾滋病性病， 2006， 12(03): 271-272.
[355] 程绍辉，王栩冬，郑媛媛，等. 天津市男男同志社区开展VCT的具体措施和经验[J]. 中国艾滋病性病， 2011， 17(05): 591-592.
[356] 陆群，钱耀忠，孙红专，等. 上海市奉贤区社区小组参与MSM动员HIV检测工作的效果[J]. 职业与健康， 2015， 31(10): 1340-1343.
[357] 梁军，李怡，廖斌，等. 昆明市针对男男性行为者购买社区小组的艾滋病防治服务模式[J]. 中国艾滋病性病， 2018， 24(07): 742-744.
[358] 徐慧芳，王鸣. 充分发挥互联网优势 控制艾滋病在MSM中的蔓延[J]. 中华流行病学杂志， 2019(10): 1197-1202.
[359] 曾春娥. 我国女同性恋者的健康问题及其对策[J]. 中国性科学， 2004， 13(03): 39-41.
[360] 刘援朝，汪洁. 从心理咨询角度思考同性恋[J]. 中国性科学， 2005， 14(02): 35-37.
[361] 王明，邓艳红，董海英，等. 男男性行为者安全套使用影响因素分析[J]. 中国健康教育， 2005， 21(07): 527-528.
[362] 曹宁校. 性取向的心理健康与性传播疾病[J]. 中国性科学， 2005(01): 31-33.
[363] 丁贤彬，冯连贵，卢戎戎. 男男性接触人群艾滋病防治的思考[J]. 热带医学杂志， 2007， 7(01): 91-93.
[364] 张利宏，郑建中，张雪芬. 健康教育理论在改变同性恋人群生存现状中的应用[J]. 山西医科大学学报， 2007， 38(01): 89-93.
[365] 曹宁校，邢建民. 中国MSM艾滋病性病干预中的现象与思考[J]. 中国艾滋病性病， 2009， 15(01): 79-81.
[366] 徐文贤，朱武通，陈中文. 嘉兴市MSM人群艾滋病高危行为干预探讨[J]. 浙江预防医学， 2009， 21(04): 23-25.
[367] 高永军. 男男性接触者人群预防艾滋病的健康教育和行为干预[J]. 职业与健康， 2009， 25(03): 314-315.
[368] 蒋明华. 男男性接触者性病后的医学人文关怀[J]. 中国性科学， 2009， 17(01): 38-39.
[369] 江华，Lu Charles Damien，魏伟，等. 中国男男性行为群体HIV干预面临的挑战:基于社区和已发表证据的循证评价[J]. 预防医学情报杂志， 2009， 25(07): 499-503.
[370] 胡青娟，薛国平. 农村男男性接触者高危人群干预研究[J]. 实用医技杂志， 2011， 18(03): 261.
[371] 邵林宁，来亦超，陈海燕. 南京市秦淮区场所型男男性行为人群干预模式探讨[J]. 中国现代医生， 2012， 50(23): 104-106.

[372] 国青，张健，吴天亮，等. 从医学伦理学角度审视同性恋面临的有关问题[J]. 中国性科学， 2013， 22(12): 91-93.
[373] 周方，宋均，陈再芳，等. MSM人群艾滋病自愿咨询检测NGO服务模式探讨[J]. 公共卫生与预防医学， 2013， 24(04): 97-98.
[374] 张丝艳，徐震雷，李红，等. 对同性恋大学生进行非问题导向性个性化辅导的方法探究[J]. 中国性科学， 2014， 23(02): 94-97.
[375] 王园园，李十月. 男男性行为人群危险性行为影响因素对干预的启示[J]. 公共卫生与预防医学， 2015， 26(05): 1-5.
[376] 王一博. 同性恋大学生的心理健康与高校心理工作研究[J]. 林区教学， 2015(04): 114-115.
[377] 李真晖，陆林. 浴池型男男性行为人群HIV感染现状及预防[J]. 皮肤病与性病， 2016， 38(04): 275-278.
[378] 何纳. 男男同性性行为人群艾滋病持续蔓延需要精准防控[J]. 上海预防医学， 2016， 28(12): 837-838.
[379] 来帅. 社会排斥图景下的同性恋群体研究[J]. 中国性科学， 2018， 27(11): 153-157.
[380] 薛清彩，钟占琼，张昌惠. 四川省男-男性行为人群HIV感染相关文献综述[J]. 四川生理科学杂志， 2018， 40(03): 211-213.
[381] 陈晓东，黄镜颖. 社会工作介入男男性行为人群艾滋病预防的伦理困境[J]. 中国社会医学杂志， 2019， 36(05): 484-486.
[382] 尹承东，周晓燕. 高校大学生男男性行为人群中HIV传播相关因素分析[J]. 中国校医， 2020， 34(01): 69-71.
[383] 高云，王曙光，张胜康. 参与式交流策略与男同性恋艾滋病易感行为改变的实践[J]. 现代预防医学， 2005， 32(11): 94-97.
[384] 王曙光，高云，张胜康. 男男性关系人群行为改变多样化策略的成功实践[J]. 中国艾滋病性病， 2005， 11(02): 109-112.
[385] 许晶. 云南 四川省男同性恋人群项目干预效果评估[J]. 中国艾滋病性病， 2006， 12(03): 231-232.
[386] 王国芳，陆朝国，袁飞，等. 在MSM人群中开展预防艾滋病性病健康教育探索[J]. 中国艾滋病性病， 2007， 13(03): 264-265.
[387] 农全兴，徐永芳，林新勤，等. 男男性接触者艾滋病干预效果分析[J]. 预防医学情报杂志， 2010， 26(03): 200-203.
[388] 马虹，李志和. 长春市MSM人群艾滋病知识干预效果分析[J]. 吉林医学， 2010， 31(13): 1841-1843.
[389] 李芳，耿文奎，刘金纪，等. “家庭式同伴教育”干预模式对男男性接触者行为干预效果研究[J]. 内科， 2012， 7(05): 518-520.
[390] 李秀菊，崔留欣，施建春，等. 2011年南阳市对男男同性恋人群预防艾滋病干预结果分析[J]. 河南预防医学杂志， 2013， 24(02): 119-120.
[391] 郭凤莲. MSM人群干预前后AIDS知识态度行为变化[J]. 中国卫生产业， 2014， 11(22): 3-5.
[392] 张桐亮，刘忠，孙淑艳. 男性同性恋人群艾滋病防治工作网络干预效果评价[J]. 预防医学论坛， 2014， 20(08): 609-610.
[393] 季洪兵，李伟，王毅，等. 绵阳市安县地区男男性接触人群干预前后艾滋病知识、行为改变情况[J]. 社区医学杂志， 2014， 12(21): 57-61.
[394] 吕金伟，杨康生，成国明，等. 芜湖市男男性行为者焦虑状况及干预效果调查[J]. 职业与健康， 2014， 30(13): 1836-1838.
[395] 王莉，赵继民，陆子春. 2007～2013年佳木斯地区男男同性恋人群干预调查[J]. 黑龙江医药科学， 2015， 38(03): 144-146.
[396] 高良敏，付金翠，陈昭，等. 玉溪市MSM人群同伴教育骨干感染或传播HIV的风险研究[J]. 皮肤病与性病， 2015， 37(06): 332-334.
[397] 李秀菊. 对男男性行为者患艾滋病高危行为及干预措施分析[J]. 河南预防医学杂志， 2016， 27(06): 439-443.
[398] 王波，杨春霞，梁俊波，等. 2011-2014年西充县男男性接触人群艾滋病防治同伴教育效果评价[J]. 预防医学情报杂志， 2016， 32(05): 490-493.
[399] 丁琳，魏小健，赵涛，等. 2014-2018年南阳市MSM人群预防艾滋病干预效果评价及影响因素分析[J]. 河南预防医学杂志， 2019， 30(09): 681-684.
[400] 樊荣丽，严关明，李慎坚，等. 同伴推动干预在男男同性恋人群艾滋病防治中应用分析[J]. 中国初级卫生保健， 2019， 33(04): 69-70.
[401] 孙钦敏，吴淑颜，刘爱敏. 烟台市男男性接触人群行为调查[J]. 职业与健康， 2007， 23(20): 1841-1842.
[402] 许航，胡伟，赵宏宇. 鞍山地区男男同性恋人群艾滋病感染情况调查[J]. 中国卫生检验杂志， 2009， 19(03): 658-713.
[403] 李兰笛，赵文彬，姜延峰. 鞍山市MSM人群HIV/AIDS感染相关因素的现况调查[J]. 中国冶金工业医学杂志， 2010， 27(06): 684-685.
[404] 罗向红，李莹，陈继军，等. 男男性行为人群现状及流行病学调查[J]. 卫生职业教育， 2010， 28(09): 121-122.
[405] 张威，吴雅冬，姚敏，等. 贵阳市云岩区男男同性恋艾滋病阳性病例流行病学特征分析[J]. 贵州医药， 2016， 40(09): 991.

[406] 季顺锋. 苏州市2013—2014年度男性同性恋性传播性疾病检测结果分析[J]. 中国卫生产业， 2016， 13(20): 120-122.
[407] 张博. 丰县2011～2015年MSM人群艾滋病感染情况调查分析[J]. 中国农村卫生， 2016(24): 35-36.
[408] 周靖雯，程丽娟，许宏霞. 佳木斯地区15～24岁青年/学生中男男性行为人群艾滋病感染状况分析[J]. 黑龙江医药科学， 2017， 40(02): 122-124.
[409] 周翔，刘旺明，陈辉，等. 38例大学生男男性接触者心理咨询个案分析[J]. 皮肤病与性病， 2017， 39(01): 35-37.
[410] 毛敏，袁孟军，董洪. 2011-2017年德阳市男男同性性行为人群哨点监测结果分析[J]. 职业卫生与病伤， 2018， 33(04): 226-229.
[411] 王存江. 男男性行为HIV感染者艾滋病相关羞辱和歧视的调查分析[J]. 当代医学， 2018， 24(35): 43-45.
[412] 李林涛. 罗湖地区男男同性性行为者艾滋病监测结果分析[J]. 河南预防医学杂志， 2018， 29(01): 17-19.
[413] 于磊，郑雅静，李凤娟，等. 探讨艾滋病检测对MSM人群感染HIV风险的影响[J]. 中国城乡企业卫生， 2018， 33(03): 81-82.
[414] 郭凌风，刘文利. 性少数群体校园暴力与欺凌的危险和保护因子[J]. 中国学校卫生， 2019， 40(01): 156-160.
[415] 宝春辉，吴迪，尹晔. 2014年～2019年某市男男性行为人群艾滋病哨点监测结果的分析[J]. 中国医药指南， 2020， 18(07): 175-176.
[416] 梁超斌，汤有贤，吴继文，等. 2016—2018年禅城区男男性行为人群艾滋病哨点监测结果分析[J]. 中国初级卫生保健， 2019， 33(09): 77-79.
[417] 卢春如. 深圳市龙岗区MSM人群HIV、TP、HSV-2病毒感染状况及影响因素分析[J]. 中国医药科学， 2019， 9(10): 172-175.
[418] 卢春如. 深圳市龙岗区MSM人群HIV感染者流行病学特征[J]. 中国城乡企业卫生， 2019， 34(01): 9-12.
[419] 钟祥海，韦东旭，管海荣. 男男性行为者艾滋病综合干预效果评价与高危性影响因素分析[J]. 中国卫生产业， 2019， 16(12): 165-167.
[420] 陈繁. 大学生同性恋群体调查报告[J]. 当代青年研究， 2007(09): 40-46.
[421] 高良敏，陈良，李连学，等. 互联网对小城镇/农村地区男男性接触者HIV/STD的风险分析[J]. 中国健康教育， 2011， 27(10): 758-761.
[422] 苏秋菊，赵文彬，郑冰，等. 鞍山市2008-2012年MSM哨点监测结果分析[J]. 中国艾滋病性病， 2014， 20(06): 459-460.
[423] 戴继坤，李云，徐洪吕. 曲靖市男男性行为人群艾滋病干预效果分析[J]. 中国卫生产业， 2015， 12(25): 154-157.
[424] 王春学，张学金，宫晓丽，等. 临朐县男男性行为人群高危行为特征及心理因素调查[J]. 预防医学论坛， 2015， 21(08): 632-633.
[425] 李程，王洋，朱琳，等. 大连市男男性行为HIV阳性者性行为调查[J]. 微量元素与健康研究， 2016， 33(06): 57-58.
[426] 叶明骞. 男男性行为人群(MSM)早期经历对成年后性行为及艾滋病预防控制的影响[J]. 口岸卫生控制， 2016， 21(02): 50-52.
[427] 张明明，梁桂平. 两中心城市男男性接触者HIV/HCV和梅毒感染状况分析[J]. 中国继续医学教育， 2017， 9(01): 46-47.
[428] 余蕾. 男男同性恋人群行为因素与HIV感染率调查分析[J]. 医学理论与实践， 2019， 32(24): 4080-4082.
[429] 石安霞，张志华，赵越，等. 男男性行为人群HIV暴露前预防需求与使用障碍研究[J]. 中华流行病学杂志， 2020(03): 343-348.
[430] 陆春燕，蒙柳仁，莫园园，等. 艾滋病高发区男男性行为人群HIV感染者的高危行为特征分析[J]. 应用预防医学， 2019， 25(01): 1-4.
[431] 曲琳，高永明，李霞，等. 内蒙古自治区2008-2012年男男性行为者艾滋病疫情分析[J]. 慢性病学杂志， 2013， 14(04): 300-305.
[432] 于宗富，张朝. 98名男同性恋者心理健康状况调查[J]. 中国神经精神疾病杂志， 2007， 33(4): 252-253.
[433] 于宗富，张朝. 男同性恋者心理健康状况调查[J]. 中国现代医学杂志， 2007， 17(18): 2291-2293.
[434] 张朝，于宗富. 男同性恋者社会生活状况98名调查[J]. 中国组织工程研究与临床康复， 2007， 11(52): 10565-10568.
[435] 于宗富，张朝. 山东男同性恋者社会生活状况自我感觉调查[J]. 现代预防医学， 2007， 34(20): 3932-3934.
[436] 董娟，孙春. 荆州市2010—2014年男男性行为人群艾滋病哨点监测结果分析[J]. 中国初级卫生保健， 2015， 29(09): 90-92.
[437] 李明炎. 荆州市2010-2014年男男同性性行为人群艾滋病监测[J]. 公共卫生与预防医学， 2015， 26(04): 95-97.
