# Supplementary material for: The Holistic Health Status of Chinese Homosexual and Bisexual Adults: A Scoping Review
Source: Front Public Health. 2021 Aug 24;9:710575. doi: 10.3389/fpubh.2021.710575 (PMC8421524; doi:10.3389/fpubh.2021.710575)
Supplement: Supplementary file 5 [file Data_Sheet_5.pdf]

**All included references**

## English references

### *Articles*


1. Lau JT, Wong WS. HIV antibody testing among male commercial sex networkers, men who have sex with men and the lower-risk male general population in Hong Kong. AIDS care. 2002 Feb;14(1):55-61.
2. Choi K-H, Liu H, Guo Y, et al. Emerging HIV-1 epidemic in China in men who have sex with men. The Lancet. 2003;361(9375):2125-2126.
3. Lau JT, Kim JH, Lau M, et al. Prevalence and risk behaviors of Chinese men who seek same-sex partners via the internet in Hong Kong. Aids Educ Prev. 2003 Dec;15(6):516-28.
4. Wong C-Y, Tang CS-K. Personality, psychosocial variables, and life satisfaction of Chinese gay men in Hong Kong. Journal of Happiness Studies. 2003;4(3):285-293.
5. Choi K, Gibson DR, Han L, et al. High levels of unprotected sex with men and women among men who have sex with men: a potential bridge of HIV transmission in Beijing, China. AIDS Education & Prevention. 2004;16(1):19-30.
6. Kuang M-F, Mathy RM, Carol HM, et al. The Effects of Sexual Orientation, Gender Identity, and Gender Role on the Mental Health of Women in Taiwan'sT-PoLesbian Community. Journal of Psychology & Human Sexuality. 2004;15(4):163-184.
7. Lau JT, Kim JH, Lau M, et al. HIV related behaviours and attitudes among Chinese men who have sex with men in Hong Kong: a population based study. Sex Transm Infect. 2004 Dec;80(6):459-65.
8. Lau JT, Kim JH, Lau M, et al. Prevalence and risk behaviors of Hong Kong males who seek cross-border same-sex partners in mainland China. Sex Transm Dis. 2004 Sep;31(9):568-74.
9. Wong C-y, Tang CS-k. Coming Out Experiences and Psychological Distress of Chinese Homosexual Men in Hong Kong. Archives of Sexual Behavior. 2004 Apr;33(2):149-157.
10. Wong CY, Tang CS. Sexual practices and psychosocial correlates of current condom use among Chinese gay men in Hong Kong. Arch Sex Behav. 2004 Apr;33(2):159-67.
11. Zhang BC, Chu QS. MSM and HIV/AIDS in China. Cell Res. 2005 Nov-Dec;15(11-12):858-64.
12. Chen Y, Chen Y. Lesbians in China's mainland: A brief introduction. Journal of Lesbian Studies. 2006;10(3-4):113-125.
13. Choi KH, Lui H, Guo Y, et al. Lack of HIV testing and awareness of HIV infection among men who have sex with men, Beijing, China. Aids Educ Prev. 2006 Feb;18(1):33-43.
14. He Q, Wang Y, Lin P, et al. Potential bridges for HIV infection to men who have sex with men in Guangzhou, China. AIDS & Behavior. 2006;10:S17-23.
15. Jiang J, Cao N, Zhang J, et al. High prevalence of sexually transmitted diseases among men who have sex with men in Jiangsu Province, China. Sexually Transmitted Diseases. 2006;33(2):118-123.
16. Lau JT, Kim JH, Tsui HY. Prevalence and factors of sexual problems in Chinese males and females having sex with the same-sex partner in Hong Kong: a population-based study. Int J Impot Res. 2006 Mar-Apr;18(2):130-40.
17. Liu H, Yang H, Li X, et al. Men who have sex with men and human immunodeficiency

virus/sexually transmitted disease control in China. Sex Transm Dis. 2006 Feb;33(2):68-76.
18. Liu JX, Choi K. Experiences of social discrimination among men who have sex with men in Shanghai, China. AIDS & Behavior. 2006;10:S25-33.
19. Sun ZX, Farrer J, Choi KH. Sexual Identity Among Men Who Have Sex with Men in Shanghai. China Perspectives. 2006 Mar-Apr;64.
20. Wong WC, Zhang J, Wu SC, et al. The HIV related risks among men having sex with men in rural Yunnan, China: a qualitative study. Sex Transm Infect. 2006 Apr;82(2):127-30.
21. Zhou YQR. Homosexuality, seropositivity, and family obligations: Perspectives of HIV-infected men who have sex with men in China. Culture Health & Sexuality. 2006 Nov-Dec;8(6):487-500.
22. Choi K-H, Ning Z, Gregorich SE, et al. The influence of social and sexual networks in the spread of HIV and syphilis among men who have sex with men in Shanghai, China. JAIDS Journal of Acquired Immune Deficiency Syndromes. 2007 May;45(1):77-84.
23. Gao MY, Wang S. Participatory communication and HIV/AIDS prevention in a Chinese marginalized (MSM) population. AIDS care. 2007 Jul;19(6):799-810.
24. Ma X, Zhang Q, He X, et al. Trends in prevalence of HIV, Syphilis, Hepatitis C, Hepatitis B, and sexual risk behavior among men who have sex with men: Results of 3 consecutive respondent-driven sampling surveys in Beijing, 2004 through 2006. JAIDS Journal of Acquired Immune Deficiency Syndromes. 2007 Aug;45(5):581-587.
25. Ruan Y, Li D, Li X, et al. Relationship between syphilis and HIV infections among men who have sex with men in Beijing, China. Sexually Transmitted Diseases. 2007;34(8):592-597.
26. Wong WC, Kong TS. To determine factors in an initiation of a same-sex relationship in rural China: using ethnographic decision model. AIDS care. 2007 Aug;19(7):850-7.
27. Zhang D, Bi P, Lv F, et al. Internet use and risk behaviours: an online survey of visitors to three gay websites in China. Sex Transm Infect. 2007 Dec;83(7):571-6.
28. Zhang D, Bi P, Lv F, et al. Changes in HIV prevalence and sexual behavior among men who have sex with men in a northern Chinese city: 2002-2006. J Infect. 2007 Nov;55(5):456-63.
29. Zhang X, Wang C, Hengwei W, et al. Risk factors of HIV infection and prevalence of co-infections among men who have sex with men in Beijing, China. AIDS. 2007 Dec;21(Suppl8):S53-S57.
30. Choi K, Hudes ES, Steward WT. Social discrimination, concurrent sexual partnerships, and HIV risk among men who have sex with men in Shanghai, China. AIDS & Behavior. 2008;12:S71-7.
31. Cong L, Ono-Kihara M, Xu G, et al. The characterisation of sexual behaviour in Chinese male university students who have sex with other men: a cross-sectional study. BMC public health. 2008 Jul 22;8:250.
32. Feng T, Liu X, Cai Y, et al. Prevalence of syphilis and human immunodeficiency virus infections among men who have sex with men in Shenzhen, China: 2005 to 2007. Sexually Transmitted Diseases. 2008;35(12):1022-1024.
33. Hung CC, Ko NY, Ko WC, et al. Amoebiasis among patrons visiting gay saunas in Taiwan. HIV Medicine. 2008 Oct;9(9):787-9.
34. Lau JT, Kim JH, Tsui HY. Prevalence and sociocultural predictors of sexual dysfunction among Chinese men who have sex with men in Hong Kong. Journal of Sexual Medicine. 2008 Dec;5(12):2766-79.

35. Lau JT, Lau M, Cheung A, et al. A randomized controlled study to evaluate the efficacy of an Internet-based intervention in reducing HIV risk behaviors among men who have sex with men in Hong Kong [Journal Article; Randomized Controlled Trial; Research Support, Non-U.S. Gov't]. AIDS care. 2008 Aug;20(7):820-828.
36. Lau JT, Wang M, Wong HN, et al. Prevalence of bisexual behaviors among men who have sex with men (MSM) in China and associations between condom use in MSM and heterosexual behaviors. Sex Transm Dis. 2008 Apr;35(4):406-13.
37. Li SW, Zhang XY, Li XX, et al. Detection of recent HIV-1 infections among men who have sex with men in Beijing during 2005 - 2006. Chin Med J (Engl). 2008 Jun 20;121(12):1105-8.
38. Li X, Shi W, Li D, et al. Predictors of unprotected sex among men who have sex with men in Beijing, China. Southeast Asian J Trop Med Public Health. 2008 Jan;39(1):99-108.
39. Neilands TB, Steward WT, Choi K-H. Assessment of stigma towards homosexuality in China: A study of men who have sex with men. Archives of Sexual Behavior. 2008 Oct;37(5):838-844.
40. Ruan S, Yang H, Zhu Y, et al. HIV prevalence and correlates of unprotected anal intercourse among men who have sex with men, Jinan, China. AIDS & Behavior. 2008;12(3):469-475.
41. Tao X, Gai R, Zhang X, et al. Prevalence of HIV infection and HIV-related sex risk behaviors in men who have sex with men in Shandong Province, China. Biosci Trends. 2008 Jun;2(3):97-100.
42. Zhang D, Bi P, Lv F, et al. Differences between Internet and community samples of MSM: implications for behavioral surveillance among MSM in China. AIDS care. 2008;20(9):1128-1137.
43. Chapman J, Cai Y, Hillier S, et al. Sex and sexuality in the Shenzhen tongzhi circle: HIV risk context and migrant men who have sex with men in China. Culture, Health & Sexuality. 2009 October;11(7):689-702.
44. Feng L, Ding X, Lu R, et al. High HIV prevalence detected in 2006 and 2007 among men who have sex with men in China's largest municipality: An alarming epidemic in Chongqing, China. JAIDS Journal of Acquired Immune Deficiency Syndromes. 2009 Sep;52(1):79-85.
45. Gao L, Zhang L, Jin Q. Meta-analysis: prevalence of HIV infection and syphilis among MSM in China. Sex Transm Infect. 2009 Sep;85(5):354-8.
46. He Q, Wang Y, Lin P, et al. High prevalence of risk behaviour concurrent with links to other high-risk populations: a potentially explosive HIV epidemic among men who have sex with men in Guangzhou, China. Sex Transm Infect. 2009 Sep;85(5):383-90.
47. Hong FC, Zhou H, Cai YM, et al. Prevalence of syphilis and HIV infections among men who have sex with men from different settings in Shenzhen, China: implications for HIV/STD surveillance. Sex Transm Infect. 2009 Feb;85(1):42-4.
48. Lau JT, Wang M, Tse YK, et al. HIV-related behaviors among men who have sex with men in China: 2005-2006. Aids Educ Prev. 2009 Aug;21(4):325-39.
49. Lee SS, Tam DK, Ho RL, et al. Social network methodology for studying HIV epidemiology in men having sex with men. J Infect Public Health. 2009;2(4):177-83.
50. Lee SS, Tam DKP, Tan Y, et al. An exploratory study on the social and genotypic clustering of HIV infection in men having sex with men. AIDS. 2009 Aug 24;23(13):1755-1764.
51. Liu H, Feng T, Feng H, et al. Egocentric networks of Chinese men who have sex with men: network components, condom use norms, and safer sex. AIDS Patient Care & STDs. 2009;23(10):885-893.

52. Ruan S, Yang H, Zhu Y, et al. Rising HIV prevalence among married and unmarried among men who have sex with men: Jinan, China. AIDS & Behavior. 2009 Aug;13(4):671-676.
53. Ruan Y, Jia Y, Zhang X, et al. Incidence of HIV-1, syphilis, hepatitis B, and hepatitis C virus infections and predictors associated with retention in a 12-month follow-up study among men who have sex with men in Beijing, China. JAIDS Journal of Acquired Immune Deficiency Syndromes. 2009 Dec 15;52(5):604-610.
54. Ruan Y, Luo F, Jia Y, et al. Risk factors for syphilis and the prevalence of HIV, hepatitis B and C among men who have sex with men in Beijing, China: Implications for HIV prevention. AIDS and Behavior. 2009 Aug;13(4):663-670.
55. Ruan Y, Qian H, Li D, et al. Willingness to be circumcised for preventing HIV among Chinese men who have sex with men. AIDS Patient Care & STDs. 2009;23(5):315-321.
56. Wong FY, Huang ZJ, Wang W, et al. STIS AND HIV AMONG MEN HAVING SEX WITH MEN IN CHINA: A TICKING TIME BOMB? Aids Educ Prev. 2009 Oct;21(5):430-46.
57. Xiao Y, Ding X, Li C, et al. Prevalence and correlates of HIV and syphilis infections among men who have sex with men in Chongqing Municipality, China. Sexually Transmitted Diseases. 2009 Oct;36(10):647-656.
58. Chow PK, Cheng ST. Shame, internalized heterosexism, lesbian identity, and coming out to others: A comparative study of lesbians in mainland China and Hong Kong. J Couns Psychol. 2010 Jan;57(1):92-104.
59. Dongliang L, Yujiang J, Yuhua R, et al. Correlates of Incident Infections for HIV, Syphilis, and Hepatitis B Virus in A Cohort of Men Who Have Sex with Men in Beijing. AIDS Patient Care & STDs. 2010;24(9):595-602.
60. Feng Y, Wu Z, Detels R. Evolution of men who have sex with men community and experienced stigma among men who have sex with men in Chengdu, China. JAIDS Journal of Acquired Immune Deficiency Syndromes. 2010 Feb 01;53(Suppl 1):S98-S103.
61. Feng Y, Wu Z, Detels R, et al. HIV/STD prevalence among men who have sex with men in Chengdu, China and associated risk factors for HIV infection. JAIDS Journal of Acquired Immune Deficiency Syndromes. 2010 Feb 01;53(Suppl 1):S74-S80.
62. Gao L, Zhou F, Li X, et al. Anal HPV infection in HIV-positive men who have sex with men from China. PLoS One. 2010 Dec 6;5(12):e15256.
63. Ha TH, Liu H, Cai Y, et al. Concurrent sexual partnerships among men who have sex with men in shenzhen, china. Sexually Transmitted Diseases. 2010 Aug;37(8):506-511.
64. Li H, Lau JF, Holroyd E, et al. Sociocultural facilitators and barriers to condom use during anal sex among men who have sex with men in Guangzhou, China: an ethnographic study. AIDS care. 2010 Dec;22(12):1481-1486.
65. Li Q, Luo F, Zhou Z, et al. Willingness to participate in HIV vaccine clinical trials among Chinese men who have sex with men. Vaccine. 2010;28(29):4638-4643.
66. Liu S, Wang K, Yao S, et al. Knowledge and risk behaviors related to HIV/AIDS, and their association with information resource among men who have sex with men in Heilongjiang province, China. BMC public health. 2010 May 14;10:250.
67. Mak WW, Chong ES, Kwong MM. Prevalence of same-sex intimate partner violence in Hong Kong. Public Health. 2010 Mar;124(3):149-52.
68. Mak WWS, Ng AC, Mo PKH, et al. Coming out among lesbians, gays, and bisexual individuals in Hong Kong: Application of the theory of planned behavior and the moderating role of

attitudinal ambivalence. Sex Roles: A Journal of Research. 2010 Aug;63(3-4):189-200.
69. Muessig KE, Tucker JD, Wang BX, et al. HIV and syphilis among men who have sex with men in China: the time to act is now. Sex Transm Dis. 2010 Apr;37(4):214-6.
70. Shieh W-Y. Gay and lesbian couple relationship commitment in Taiwan: A preliminary study. Journal of Homosexuality. 2010 Nov;57(10):1334-1354.
71. Tang HL, Zhang WD, Lv F. Behavioral Features of Men Who Have Sex with Men (MSM) in Harbin, China. World Medical & Health Policy. 2010 Apr;2(1):317-330.
72. Tsui HY, Lau JT. Comparison of risk behaviors and socio-cultural profile of men who have sex with men survey respondents recruited via venues and the internet. BMC public health. 2010 May 6;10:232.
73. Xiao Y, Sun J, Li C, et al. Prevalence and correlates of HIV and syphilis infections among men who have sex with men in seven provinces in China with historically low HIV prevalence. JAIDS Journal of Acquired Immune Deficiency Syndromes. 2010 Feb 01;53(Suppl 1):S66-S73.
74. Xu JJ, Zhang M, Brown K, et al. Syphilis and HIV seroconversion among a 12-month prospective cohort of men who have sex with men in Shenyang, China. Sex Transm Dis. 2010 Jul;37(7):432-9.
75. Yang H, Hao C, Huan X, et al. HIV incidence and associated factors in a cohort of men who have sex with men in Nanjing, China. Sex Transm Dis. 2010 Apr;37(4):208-13.
76. Zhang H, Wu Z, Zheng Y, et al. A pilot intervention to increase condom use and HIV testing and counseling among men who have sex with men in Anhui, China. JAIDS Journal of Acquired Immune Deficiency Syndromes. 2010 Feb 01;53(Suppl 1):S88-S92.
77. Zou H, Wu Z, Yu J, et al. Sexual risk behaviors and HIV infection among men who have sex with men who use the Internet in Beijing and Urumqi, China. JAIDS Journal of Acquired Immune Deficiency Syndromes. 2010 Feb 01;53(Suppl 1):S81-S87.
78. Bai HL, Huan XP, Tang WM, et al. A survey of HIV infection and related high-risk factors among men who have sex with men in Suzhou, Jiangsu, China. Journal of Biomedical Research. 2011 Jan;25(1):17-24.
79. Berg CJ, Nehl EJ, Wong FY, et al. Prevalence and correlates of tobacco use among a sample of MSM in Shanghai, China. Nicotine Tob Res. 2011 Jan;13(1):22-8.
80. Chen B, Zhang M, Yang D, et al. Acceptability of female condom use in money boys compared with other men who have sex with men in Chengdu, China: a comparative study. Sex Health. 2011 Jun;8(2):262-3.
81. Chen Y-J, Lin Y-T, Chen M, et al. Risk factors for HIV-1 seroconversion among Taiwanese men visiting gay saunas who have sex with men. BMC Infectious Diseases. 2011;11(1):334-334.
82. Chow EP, Wilson DP, Zhang J, et al. Human immunodeficiency virus prevalence is increasing among men who have sex with men in China: findings from a review and meta-analysis. Sexually Transmitted Diseases. 2011 Sep;38(9):845-857.
83. Chow EP, Wilson DP, Zhang L. HIV and syphilis co-infection increasing among men who have sex with men in China: a systematic review and meta-analysis. PLoS One. 2011;6(8):e22768.
84. Chow EP, Wilson DP, Zhang L. What is the potential for bisexual men in China to act as a bridge of HIV transmission to the female population? Behavioural evidence from a systematic review and meta-analysis. BMC Infect Dis. 2011 Sep 15;11(1):242.
85. Fei Z, Peng L, Huifang X, et al. Possible Increase in HIV and Syphilis Prevalence Among Men Who Have Sex with Men in Guangzhou, China: Results from a Respondent-Driven Sampling

Survey. AIDS & Behavior. 2011 Jul;15(5):1058-1066.
86. Gu J, Lau JT, Tsui H. Psychological factors in association with uptake of voluntary counselling and testing for HIV among men who have sex with men in Hong Kong. Public Health. 2011 May;125(5):275-82.
87. Guo Y, Li X, Fang X, et al. A comparison of four sampling methods among men having sex with men in China: implications for HIV/STD surveillance and prevention. AIDS care. 2011;23(11):1400-1409.
88. Guo Y, Li X, Stanton B. HIV-Related Behavioral Studies of Men Who Have Sex with Men in China: A Systematic Review and Recommendations for Future Research. AIDS & Behavior. 2011 Apr;15(3):521-534.
89. Hao C, Yan H, Yang H, et al. The incidence of syphilis, HIV and HCV and associated factors in a cohort of men who have sex with men in Nanjing, China. Sex Transm Infect. 2011 Apr;87(3):199-201.
90. He Q, Xia Y, Raymond HF, et al. HIV trends and related risk factors among men having sex with men in mainland China: findings from a systematic literature review. Southeast Asian J Trop Med Public Health. 2011 May;42(3):616-33.
91. Lau JT, Lin C, Hao C, et al. Public health challenges of the emerging HIV epidemic among men who have sex with men in China. Public Health. 2011 May;125(5):260-5.
92. Lau JT, Zhang J, Yan H, et al. Acceptability of circumcision as a means of HIV prevention among men who have sex with men in China. AIDS care. 2011 Nov;23(11):1472-82.
93. Li HM, Peng RR, Li J, et al. HIV incidence among men who have sex with men in China: a meta-analysis of published studies. PLoS One. 2011;6(8):e23431.
94. Liao M, Kang D, Jiang B, et al. Bisexual Behavior and Infection with HIV and Syphilis Among Men Who Have Sex with Men Along the East Coast of China. AIDS Patient Care & STDs. 2011;25(11):683-691.
95. Liu J, Qu B, Guo H-q, et al. Factors That Influence Risky Sexual Behaviors Among Men Who Have Sex with Men in Liaoning Province, China: A Structural Equation Model. AIDS Patient Care & STDs. 2011;25(7):423-429.
96. Ruan Y, Pan SW, Chamot E, et al. Sexual mixing patterns among social networks of HIV-positive and HIV-negative Beijing men who have sex with men: A multilevel comparison using roundtable network mapping. AIDS care. 2011 Aug;23(8):1014-1025.
97. Song Y, Li X, Zhang L, et al. HIV-testing behavior among young migrant men who have sex with men (MSM) in Beijing, China. AIDS care. 2011;23(2):179-186.
98. Teng Y, Mak WW. The role of planning and self-efficacy in condom use among men who have sex with men: an application of the Health Action Process Approach model. Health Psychol. 2011 Jan;30(1):119-28.
99. Wei C, Ruan S, Zhao J, et al. Which Chinese men who have sex with men miss out on HIV testing? Sex Transm Infect. 2011 Apr;87(3):225-8.
100. Xu JJ, Reilly KH, Lu CM, et al. A cross-sectional study of HIV and syphilis infections among male students who have sex with men (MSM) in northeast China: implications for implementing HIV screening and intervention programs. BMC public health. 2011 May 10;11:287.
101. Yun K, Xu JJ, Reilly KH, et al. Prevalence of bisexual behaviour among bridge population of men who have sex with men in China: a meta-analysis of observational studies. Sex Transm

Infect. 2011 Dec;87(7):563-70.
102. Zhang L, Chow EPF, Wilson DP. Men who have sex with men in China have relatively low numbers of sexual partners [Article]. Infectious Disease Reports. 2011;3(1):46-51.
103. Zhang M, Chu Z, Wang H, et al. A rapidly increasing incidence of HIV and syphilis among men who have sex with men in a major city of China. AIDS Res Hum Retroviruses. 2011 Nov;27(11):1139-40.
104. Zheng L, Lippa RA, Zheng Y. Sex and sexual orientation differences in personality in China. Archives of Sexual Behavior. 2011 Jun;40(3):533-541.
105. Zheng Y, Zheng L. Sexual self-labels and personality differences among Chinese lesbians. Social Behavior and Personality: An International Journal. 2011;39(7):955-962.
106. Chen G, Li Y, Zhang B, et al. Psychological characteristics in high-risk MSM in China. BMC public health. 2012 Jan 20;12(1):58-58.
107. Cheng SH, Chu FY, Lin YS, et al. Influence of age and CD4+ T cell counts on the prevalence of genital human papillomavirus infection among HIV-seropositive men who have sex with men in Taiwan. J Med Virol. 2012 Dec;84(12):1876-83.
108. Chow E, Wilson D, Zhang L. Patterns of Condom Use Among Men Who Have Sex with Men in China: A Systematic Review and Meta-Analysis. AIDS & Behavior. 2012 Apr;16(3):653-663.
109. Chow EP, Wilson DP, Zhang L. The rate of HIV testing is increasing among men who have sex with men in China. HIV Med. 2012 May;13(5):255-63.
110. Chow EP, Wilson DP, Zhang L. Estimating HIV incidence among female partners of bisexual men in China. Int J Infect Dis. 2012 May;16(5):e312-20.
111. Fan S, Lu H, Ma X, et al. Behavioral and Serologic Survey of Men Who Have Sex with Men in Beijing, China: Implication for HIV Intervention. AIDS Patient Care & STDs. 2012;26(3):148-155.
112. Guo Y, Li X, Song Y, et al. Bisexual behavior among Chinese young migrant men who have sex with men: implications for HIV prevention and intervention. AIDS care. 2012;24(4):451-458.
113. Hao C, Huan X, Yan H, et al. A randomized controlled trial to evaluate the relative efficacy of enhanced versus standard voluntary counseling and testing on promoting condom use among men who have sex with men in China [Comparative Study; Journal Article; Randomized Controlled Trial; Research Support, Non-U.S. Gov't]. AIDS and behavior. 2012 Jul;16(5):1138-1147.
114. He Q, Peng WJ, Zhang JQ, et al. Prevalence of unprotected anal intercourse and unprotected vaginal intercourse among HIV-positive men who have sex with men in China: a meta-analysis. Sexually Transmitted Infections. 2012;88(3):229-233.
115. Huang ZJ, He N, Nehl EJ, et al. Social network and other correlates of HIV testing: Findings from male sex workers and other MSM in Shanghai, China. AIDS and Behavior. 2012 May;16(4):858-871.
116. Jackson T, Huang A, Chen H, et al. Cognitive, psychosocial, and sociodemographic predictors of willingness to use HIV pre-exposure prophylaxis among Chinese men who have sex with men. AIDS Behav. 2012 Oct;16(7):1853-61.
117. Jie W, Ciyong L, Xueqing D, et al. A syndemic of psychosocial problems places the MSM (men who have sex with men) population at greater risk of HIV infection. PLoS One. 2012;7(3):e32312.

118. Ko N-Y, Koe S, Lee H-C, et al. Online sex-seeking, substance use, and risky behaviors in Taiwan: Results from the 2010 Asia Internet MSM Sex Survey. Archives of Sexual Behavior. 2012 Oct;41(5):1273-1282.
119. Kong TK, Laidler K, Pang H. Relationship type, condom use and HIV/AIDS risks among men who have sex with men in six Chinese cities. AIDS care. 2012;24(4):517-528.
120. Lau JT, Yan H, Lin C, et al. How willing are men who have sex with men in China to be circumcised for the sake of protecting his female sex partner? J Sex Med. 2012 Jul;9(7):1904-12.
121. Lee SS, Lam AN, Lee CK, et al. Virtual versus physical channel for sex networking in men having sex with men of sauna customers in the City of Hong Kong. PLoS One. 2012;7(2):e31072.
122. Li DL, Li SM, Liu YJ, et al. HIV incidence among men who have sex with men in Beijing: a prospective cohort study. Bmj Open. 2012;2(6).
123. Li Q, Liu Y, Zhou Z, et al. Online sex-seeking behaviors among men who have sex with men: implications for investigation and intervention. AIDS Behav. 2012 Aug;16(6):1690-8.
124. Li X, Lu H, Ma X, et al. HIV/AIDS-Related Stigmatizing and Discriminatory Attitudes and Recent HIV Testing Among Men Who Have Sex With Men in Beijing. AIDS & Behavior. 2012;16(3):499-507.
125. Li X, Lu H, Raymond HF, et al. Untested and undiagnosed: barriers to HIV testing among men who have sex with men, Beijing, China. Sexually Transmitted Infections. 2012;88(3):187-193.
126. Liu J, Qu B, Ezeakile MC, et al. Factors associated with unprotected anal intercourse among men who have sex with men in Liaoning Province, China. PLoS One. 2012;7(11):e50493.
127. Liu Y, Li X, Zhang L, et al. Correlates of consistent condom use among young migrant men who have sex with men (MSM) in Beijing, China. European Journal of Contraception & Reproductive Health Care. 2012;17(3):219-228.
128. Ma W, Raymond F, Wilson EC, et al. Participation of HIV prevention programs among men who have sex with men in two cities of China-a mixed method study. BMC public health. 2012 Oct;12.
129. Nehl E, Wong F, He N, et al. Prevalence and correlates of alcohol use among a sample of general MSM and money boys in Shanghai, China. AIDS care. 2012;24(3):324-330.
130. Nehl EJ, Nakayama KK, He N, et al. Substance Use and Sexual Risks Among General MSM and Money Boys in Shanghai, China. Journal of Drug Issues. 2012 July;42(3):263-278.
131. Peng Z, Yang H, Norris J, et al. HIV incidence and predictors associated with retention in a cohort of men who have sex with men in Yangzhou, Jiangsu Province, China. PLoS One. 2012;7(12):e52731.
132. Song Y, Li XM, Zhang LY, et al. Demographic and behavioral determinants of self-reported history of sexually-transmitted diseases (STDs) among young migrant men who have sex with men (MSM) in Beijing, China. Health Education Journal. 2012 Nov;71(6):699-708.
133. Tseng YT, Sun HY, Chang SY, et al. Seroprevalence of hepatitis virus infection in men who have sex with men aged 18-40 years in Taiwan. J Formos Med Assoc. 2012 Aug;111(8):431-8.
134. Wang C, Wang Y, Huang X, et al. Prevalence and factors associated with hepatitis B immunization and infection among men who have sex with men in Beijing, China. PLoS One. 2012;7(10):e48219.
135. Wang K, Yan H, Liu Y, et al. Increasing prevalence of HIV and syphilis but decreasing rate of

self-reported unprotected anal intercourse among men who had sex with men in Harbin, China: results of five consecutive surveys from 2006 to 2010. International Journal of Epidemiology. 2012 Apr;41(2):423-432.
136. Wang X, Norris JL, Liu Y, et al. Health-related attitudes and risk factors for sexually transmitted infections of Chinese women who have sex with women [Article]. Chinese Medical Journal. 2012;125(16):2819-2825.
137. Wang X, Norris JL, Liu Y, et al. Risk behaviors for reproductive tract infection in women who have sex with women in Beijing, China. PLoS ONE. 2012 Jul 02;7(7):6.
138. Yang Y, Li X, Zhang Z, et al. Association of human papillomavirus infection and abnormal anal cytology among HIV-infected MSM in Beijing, China. PLoS One. 2012;7(4):e35983.
139. Ye S, Xiao Y, Jin C, et al. Effectiveness of integrated HIV prevention interventions among Chinese men who have sex with men: evaluation of a 16-city public health program. PLoS One. 2012;7(12):e50873.
140. Yin YP, Chen SC, Wang HC, et al. Prevalence and Risk Factors of HSV-2 Infection and HSV-2/HIV Coinfection in Men Who Have Sex With Men in China: A Multisite Cross-Sectional Study. Sexually Transmitted Diseases. 2012;39(5):354-358.
141. Zhang L, Chow EP, Wilson DP. Distributions and trends in sexual behaviors and HIV incidence among men who have sex with men in China. BMC public health. 2012 Jul 24;12:546.
142. Zhang L, Ding X, Lu R, et al. Predictors of HIV and syphilis among men who have sex with men in a Chinese metropolitan city: comparison of risks among students and non-students. PLoS One. 2012;7(5):e37211.
143. Zhang Y, Chen P, Lu R, et al. Prevalence of HIV among men who have sex with men in Chongqing, China, 2006-2009: cross-sectional biological and behavioural surveys. Sexually Transmitted Infections. 2012 Oct;88(6):444-450.
144. Zhao J, Cai WD, Gan YX, et al. A comparison of HIV infection and related risk factors between money boys and noncommercial men who have sex with men in Shenzhen, China. Sexually Transmitted Diseases. 2012;39(12):942-948.
145. Zheng J, Wu Z, Poundstone KE, et al. HIV, Syphilis Infection, and Risky Sexual Behaviors among Male University Students Who Have Sex with Men in Beijing, China: A Cross-Sectional Study. AIDS Education & Prevention. 2012 Feb;24(1):78-88.
146. Zheng L, Hart TA, Zheng Y. The relationship between intercourse preference positions and personality traits among gay men in china. Archives of Sexual Behavior. 2012;41(3):683-689.
147. Zheng L, Zheng Y. Efficacy of human immunodeficiency virus prevention interventions among men who have sex with men in China: a meta-analysis. Sexually Transmitted Diseases. 2012 Nov;39(11):886-893.
148. Zhou F, Gao L, Li S, et al. Willingness to accept HIV pre-exposure prophylaxis among Chinese men who have sex with men. PLoS One. 2012;7(3):e32329.
149. Zou H, Hu N, Xin Q, et al. HIV Testing Among Men Who Have Sex with Men in China: A Systematic Review and Meta-Analysis. AIDS & Behavior. 2012 Oct;16(7):1717-1728.
150. Chong ES, Mak WW, Kwong MM. Risk and protective factors of same-sex intimate partner violence in Hong Kong. J Interpers Violence. 2013 May;28(7):1476-97.
151. Chow EP, Chen L, Jing J, et al. HIV Disease Burden and Related Risk Behaviours Among Men Who Have Sex with Men in Yuxi Prefecture, Yunnan Province, China: 2010-2011. AIDS and Behavior. 2013 Sep;17(7):2387-94.

152. Chow EP, Gao L, Koo FK, et al. Qualitative exploration of HIV-related sexual behaviours and multiple partnerships among Chinese men who have sex with men living in a rural area of Yunnan Province, China. Sex Health. 2013 Dec;10(6):533-40.
153. Chow EP, Jing J, Feng Y, et al. Pattern of HIV testing and multiple sexual partnerships among men who have sex with men in China. BMC Infect Dis. 2013 Nov 16;13:549.
154. Chu Z, Xu J, Reilly KH, et al. HIV Related High Risk Behaviors and Willingness to Participate in HIV Vaccine Trials among China MSM by Computer Assisted Self-Interviewing Survey. BioMed Research International. 2013;2013:493128-493128.
155. Duan Y, Zhang H, Wang J, et al. Community-Based Peer Intervention to Reduce HIV Risk Among Men Who Have Sex With Men in Sichuan Province, China. AIDS Education & Prevention. 2013 Feb;25(1):38-48.
156. Dunkle KL, Wong FY, Nehl EJ, et al. Male-on-Male Intimate Partner Violence and Sexual Risk Behaviors Among Money Boys and Other Men Who Have Sex With Men in Shanghai, China. Sexually Transmitted Diseases. 2013 May;40(5):362-365.
157. Guo W, Wu ZY, Song AJ, et al. Impact of HIV/sexually transmitted infection testing on risky sexual behaviors among men who have sex with men in Langfang, China. Chin Med J (Engl). 2013 Apr;126(7):1257-63.
158. Hou C-N, Lu H-Y. Online Networks as a Venue for Social Support: A Qualitative Study of Married Bisexual Men in Taiwan. Journal of Homosexuality. 2013 September;60(9):1280-1296.
159. Hu Q, Xu J, Chu Z, et al. Barriers to Acceptance of Provider-Initiated Testing and Counseling among Men Who Have Sex with Men in Shenyang, China: A Cross-Sectional Study. BioMed Research International. 2013;2013:280969-280969.
160. Hu X, Wang Y. LGB identity among young Chinese: The influence of traditional culture. Journal of Homosexuality. 2013 May;60(5):667-684.
161. Hu X, Wang Y, Wu C-h. Acceptance concern and life satisfaction for Chinese LGBs: The mediating role of self-concealment. Social Indicators Research. 2013 Nov;114(2):687-701.
162. Hu Y, Qian H-Z, Sun J, et al. Anal human papillomavirus infection among HIV-infected and uninfected men who have sex with men in Beijing, China. JAIDS Journal of Acquired Immune Deficiency Syndromes. 2013 Sep 01;64(1):103-114.
163. Huan X, Tang W, Babu GR, et al. HIV risk-reduction counseling and testing on behavior change of MSM. PLoS One. 2013;8(7):e69740.
164. Huang Y-F, Nelson K, Lin Y-T, et al. Syphilis Among Men Who Have Sex With Men (MSM) in Taiwan: Its Association With HIV Prevalence, Awareness of HIV Status, and Use of Antiretroviral Therapy. AIDS & Behavior. 2013 May;17(4):1406-1414.
165. Huang Z, Wang M, Fu L, et al. Intervention to increase condom use and HIV testing among men who have sex with men in China: a meta-analysis. AIDS Res Hum Retroviruses. 2013 Mar;29(3):441-8.
166. Jin M, Yang Z, Dong Z, et al. Correlates of consistent condom use among men who have sex with men recruited through the Internet in Huzhou city: a cross-sectional survey. BMC public health. 2013 Dec 1;13(1):1101-1101.
167. Ko N-Y, Hsieh C-H, Wang M-C, et al. Effects of Internet Popular Opinion Leaders (iPOL) Among Internet-Using Men Who Have Sex With Men. Journal of Medical Internet Research. 2013 Feb 25;15(2):e40-e40.
168. Lau J, Cai W, Tsui H, et al. Prevalence and Correlates of Unprotected Anal Intercourse Among

Hong Kong Men Who Have Sex with Men Traveling to Shenzhen, China. AIDS & Behavior. 2013 May;17(4):1395-1405.
169. Lau JT, Gu J, Tsui HY, et al. Prevalence and associated factors of intention to participate in HIV voluntary counseling and testing for the first time among men who have sex with men in Hong Kong, China. Prev Med. 2013 Dec;57(6):813-8.
170. Lau JT, Tsui HY, Lau MM. A pilot clustered randomized control trial evaluating the efficacy of a network-based HIV peer-education intervention targeting men who have sex with men in Hong Kong, China [Journal Article; Randomized Controlled Trial; Research Support, Non-U.S. Gov't]. AIDS care. 2013;25(7):812-819.
171. Lau JT, Wang Z, Kim JH, et al. Acceptability of HPV vaccines and associations with perceptions related to HPV and HPV vaccines among men who have sex with men in Hong Kong. PLoS One. 2013;8(2):e57204.
172. Li G, Lu H, Li X, et al. Mutual HIV disclosure among HIV-negative men who have sex with men in Beijing, China, 2010. Archives of Sexual Behavior. 2013 Oct;42(7):1267-1273.
173. Li Y, Johnson BD, Jenkins-Guarnieri MA. Sexual identity development and subjective well-being among Chinese lesbians. International Perspectives in Psychology: Research, Practice, Consultation. 2013 Oct;2(4):242-254.
174. Li Y, Xu J, Reilly KH, et al. Prevalence of HIV and syphilis infection among high school and college student MSM in China: a systematic review and meta-analysis. PLoS One. 2013;8(7):e69137.
175. Lin H, He N, Zhou S, et al. Behavioral and Molecular Tracing of Risky Sexual Contacts in a Sample of Chinese HIV-infected Men Who Have Sex With Men. American Journal of Epidemiology. 2013;177(4):343-350.
176. Liu J, Qu B, Ezeakile MC, et al. Factors associated with HIV infection among men who have sex with men in Henan Province, China: a cross-sectional study. BMC public health. 2013 Apr 17;13:356.
177. Lu H, Han Y, He X, et al. Alcohol use and HIV risk taking among Chinese MSM in Beijing. Drug & Alcohol Dependence. 2013 Dec 1;133(2):317-323.
178. Lu H, Liu Y, Dahiya K, et al. Effectiveness of HIV risk reduction interventions among men who have sex with men in China: a systematic review and meta-analysis. PLoS One. 2013;8(8):e72747.
179. Ma W, Ding X, Lu H, et al. HIV risk perception among men who have sex with men in two municipalities of China-implications for education and intervention. AIDS care. 2013;25(3):385-389.
180. Meng X, Zou H, Beck J, et al. Trends in HIV prevalence among men who have sex with men in China 2003-09: a systematic review and meta-analysis. Sexual Health. 2013 Jul;10(3):211-219.
181. Nehl EJ, He N, Wang X, et al. Feasibility and willingness of using e-technologies for HIV prevention and research targeting Chinese MSM. AIDS care. 2013;25(7):926-930.
182. Poon CM, Lee SS. Sex networking of young men who have sex with men in densely connected saunas in Hong Kong. Sex Transm Dis. 2013 Dec;40(12):933-8.
183. She M, Zhang H, Wang J, et al. Associated factors for HIV and syphilis infection among men who have sex with men only and men who have sex with both men and women in cities of China. International Journal of STD & AIDS. 2013 Apr;24(4):293-300.

184. Song D, Zhang H, Wang J, et al. Sexual risk behaviours and their correlates among gay and non-gay identified men who have sex with men and women in Chengdu and Guangzhou, China. International Journal of STD & AIDS. 2013 Oct;24(10):780-790.
185. Song D, Zhang H, Wang J, et al. Prevalence and Correlates of HIV Infection and Unrecognized HIV Status Among Men Who Have Sex with Men and Women in Chengdu and Guangzhou, China. AIDS & Behavior. 2013 Sep;17(7):2395-2404.
186. Steward WT, Miege P, Choi KH. Charting a moral life: the influence of stigma and filial duties on marital decisions among Chinese men who have sex with men. PLoS One. 2013;8(8):e71778.
187. Tan J, Cai R, Lu Z, et al. Joint Marketing as a Framework for Targeting Men Who Have Sex With Men in China: A Pilot Intervention Study. AIDS Education & Prevention. 2013 Apr;25(2):102-111.
188. Tang W, Huan X, Mahapatra T, et al. Factors Associated with Unprotected Anal Intercourse Among Men Who Have Sex with Men: Results from a Respondent Driven Sampling Survey in Nanjing, China, 2008n. AIDS and Behavior. 2013 May;17(4):1415-22.
189. Tao J, Ruan Y, Yin L, et al. Sex with women among men who have sex with men in China: Prevalence and sexual practices. Aids Patient Care St. 2013 Sep;27(9):524-528.
190. Wang B, Li X, Stanton B, et al. Socio-demographic and behavioral correlates for HIV and syphilis infections among migrant men who have sex with men in Beijing, China. AIDS care. 2013;25(2):249-257.
191. Wang Z, Lau JT, Hao C, et al. Syphilis-related perceptions not associated with risk behaviors among men who have sex with men having regular male sex partner(s) in Nanjing, China. AIDS care. 2013 Aug;25(8):1010-7.
192. Wang Z, Mo PK, Lau JT, et al. Acceptability of HPV vaccines and perceptions related to genital warts and penile/anal cancers among men who have sex with men in Hong Kong. Vaccine. 2013 Sep 23;31(41):4675-81.
193. Wei S, Zhang H, Wang J, et al. HIV and Syphilis Prevalence and Associated Factors Among Young Men Who Have Sex with Men in 4 Cities in China. AIDS & Behavior. 2013 Mar;17(3):1151-1158.
194. Wu Z, Xu J, Liu E, et al. HIV and syphilis prevalence among men who have sex with men: a cross-sectional survey of 61 cities in China. Clin Infect Dis. 2013 Jul;57(2):298-309.
195. Xiao Z, Li X, Liu Y, et al. Sexual communication and condom use among Chinese men who have sex with men in Beijing. Psychology, health & medicine. 2013;18(1):98-106.
196. Xu HL, Jia MH, Min XD, et al. Factors influencing HIV infection in men who have sex with men in China. Asian J Androl. 2013 Jul;15(4):545-9.
197. Xu J, An M, Han X, et al. Prospective cohort study of HIV incidence and molecular characteristics of HIV among men who have sex with men(MSM) in Yunnan Province, China. BMC Infectious Diseases. 2013 Jan 4;13(1):3-3.
198. Xu J, Han X, Reilly KH, et al. New features of the HIV epidemic among men who have sex with men in China [Review]. Emerging Microbes and Infections. 2013;2.
199. Xu Y, Zhang Z, Li D, et al. Willingness to use the oral fluid HIV rapid test among men who have sex with men in Beijing, China. PLoS One. 2013;8(5):e64652.
200. Yu F, Nehl EJ, Zheng T, et al. A syndemic including cigarette smoking and sexual risk behaviors among a sample of MSM in Shanghai, China. Drug and Alcohol Dependence. 2013 Sep 01;132(1-2):265-270.

201. Yu L, Jiang C, Na J, et al. Elevated 12-month and lifetime prevalence and comorbidity rates of mood, anxiety, and alcohol use disorders in Chinese men who have sex with men. PLoS One. 2013;8(4):e50762.
202. Yu Y, Xiao S, Liu KQ. Dating Violence Among Gay Men in China. Journal of Interpersonal Violence. 2013 Aug;28(12):2491-2504.
203. Zhang L, Xiao Y, Lu R, et al. Predictors of HIV testing among men who have sex with men in a large Chinese city. Sexually Transmitted Diseases. 2013;40(3):235-240.
204. Zhang L, Zhang D, Yu B, et al. Prevalence of HIV infection and associated risk factors among men who have sex with men (MSM) in Harbin, P. R. China. PLoS One. 2013;8(3):e58440.
205. Zhang T, Lin H, Minhas V, et al. Prevalence and correlates of Kaposi's sarcoma-associated herpesvirus infection in a sample of men who have sex with men in Eastern China. Epidemiol Infect. 2013 Sep;141(9):1823-30.
206. Zhang X, Yu J, Li M, et al. Prevalence and Related Risk Behaviors of HIV, Syphilis, and Anal HPV Infection Among Men who have Sex with Men from Beijing, China. AIDS & Behavior. 2013 Mar;17(3):1129-1136.
207. Zhang Y, Peng B, She Y, et al. Attitudes Toward HIV Pre-Exposure Prophylaxis Among Men Who Have Sex with Men in Western China. AIDS Patient Care & STDs. 2013;27(3):137-141.
208. Zheng L, Zheng Y. Butch–femme identity and empathizing–systemizing cognitive traits in Chinese lesbians and bisexual women. Personality and Individual Differences. 2013 Jun;54(8):951-956.
209. Zhou C, Raymond HF, Ding X, et al. Anal sex role, circumcision status, and HIV infection among men who have sex with men in Chongqing, China. Archives of Sexual Behavior. 2013 Oct;42(7):1275-1283.
210. Zhou F, Li M, Li X, et al. Seroprevalence of Entamoeba histolytica infection among Chinese men who have sex with men. PLoS Negl Trop Dis. 2013;7(5):e2232.
211. Zhou W, Ma R, Sharma M, et al. Nutritional Attitudes of Homosexual People Living with Human Immunodeficiency Virus (HIV) in Chongqing, Southwest China: A Cross-Sectional Study. International Quarterly of Community Health Education. 2013;34(1):87-100.
212. Zou H, Wu Z, Yu J, et al. Internet-facilitated, voluntary counseling and testing (VCT) clinic-based HIV testing among men who have sex with men in China. PLoS One. 2013;8(2):e51919.
213. Bai X, Xu J, Yang J, et al. HIV prevalence and high-risk sexual behaviours among MSM repeat and first-time testers in China: implications for HIV prevention. J Int AIDS Soc. 2014;17:18848.
214. Berg CJ, Nehl EJ, Wang X, et al. Healthcare provider intervention on smoking and quit attempts among HIV-positive versus HIV-negative MSM smokers in Chengdu, China. AIDS care. 2014;26(9):1201-1207.
215. Berg CJ, Nehl EJ, Wang X, et al. Utilization of cessation resources among HIV-positive and HIV-negative men who smoke and who have sex with men in Chengdu, China. Nicotine & Tobacco Research. 2014 Oct;16(10):1283-1288.
216. Cai R, Zhao J, Cai W, et al. HIV Risk and Prevention Behaviors in Men Who Have Sex With Men and Women: A Respondent-Driven Sampling Study in Shenzhen, China. AIDS & Behavior. 2014 Aug;18(8):1560-1568.
217. Cai Y, Lau JT. Multi-dimensional factors associated with unprotected anal intercourse with regular partners among Chinese men who have sex with men in Hong Kong: a respondent-driven sampling survey. BMC Infect Dis. 2014 Apr 16;14:205.

218. Cao Z, Xu J, Zhang H, et al. Risk factors for syphilis among married men who have sex with men in china. Sexually Transmitted Diseases. 2014 Feb;41(2):98-102.
219. Chang YH, Liu WC, Chang SY, et al. Associated factors with syphilis among human immunodeficiency virus-infected men who have sex with men in Taiwan in the era of combination antiretroviral therapy. J Microbiol Immunol Infect. 2014 Dec;47(6):533-41.
220. Cheng W, Tang W, Zhong F, et al. Consistently high unprotected anal intercourse (UAI) and factors correlated with UAI among men who have sex with men: implication of a serial cross-sectional study in Guangzhou, China. BMC Infect Dis. 2014 Dec 18;14:696.
221. Chow EP, Lau JT, Zhuang X, et al. HIV prevalence trends, risky behaviours, and governmental and community responses to the epidemic among men who have sex with men in China. Biomed Res Int. 2014;2014:607261.
222. Chow EP, Tucker JD, Wong FY, et al. Disparities and risks of sexually transmissible infections among men who have sex with men in China: a meta-analysis and data synthesis. PLoS One. 2014;9(2):e89959.
223. Dong Z, Xu J, Zhang H, et al. HIV incidence and risk factors in Chinese young men who have sex with men--a prospective cohort study. PLoS One. 2014;9(5):e97527.
224. Fan W, Yin L, Qian H-Z, et al. HIV Risk Perception among HIV Negative or Status-Unknown Men Who Have Sex with Men in China. BioMed Research International. 2014;2014:232451-232451.
225. Guo Y, Li X, Liu Y, et al. Disclosure of same-sex behavior by young Chinese migrant men: Context and correlates. Psychology, health & medicine. 2014 Mar;19(2):190-200.
226. Han L, Bien CH, Wei C, et al. HIV self-testing among online MSM in China: Implications for expanding HIV testing among key populations. JAIDS Journal of Acquired Immune Deficiency Syndromes. 2014 Oct 01;67(2):216-221.
227. Hao C, Lau J, Zhao X, et al. Associations Between Perceived Characteristics of the Peer Social Network Involving Significant Others and Risk of HIV Transmission Among Men Who Have Sex with Men in China. AIDS & Behavior. 2014 Jan;18(1):99-110.
228. He H, Wang M, Zaller N, et al. Prevalence of syphilis infection and associations with sexual risk behaviours among HIV-positive men who have sex with men in Shanghai, China. International Journal of STD & AIDS. 2014 May;25(6):410-419.
229. Hong H, Xu G-Z, Zhang D-D. Prevalence of HIV and sexually transmitted diseases among female and male partners of men who have sex with men in Ningbo, China. International Journal of Gynecology & Obstetrics. 2014 Apr;125(1):83-83.
230. Hu Y, Lu H, Raymond H, et al. Measures of Condom and Safer Sex Social Norms and Stigma Towards HIV/AIDS Among Beijing MSM. AIDS & Behavior. 2014 Jun;18(6):1068-1074.
231. Huang D, Hu Y, Wu G, et al. HIV Prevention Services and Testing Utilization Behaviors among Men Who Have Sex with Men at Elevated Risk for HIV in Chongqing, China. BioMed Research International. 2014;2014:174870-174870.
232. Huang L, Nehl EJ, Lin L, et al. Sociodemographic and sexual behavior characteristics of an online MSM sample in Guangdong, China. AIDS care. 2014 May;26(5):648-652.
233. Koo FK, Chow EPF, Gao L, et al. Socio-cultural influences on the transmission of HIV among gay men in rural China. Culture, Health & Sexuality. 2014 Mar 16;16(3):302-315.
234. Lau JT, Wang Z, Lau M, et al. Perceptions of HPV, genital warts, and penile/anal cancer and high-risk sexual behaviors among men who have sex with men in Hong Kong. Arch Sex Behav.

2014 May;43(4):789-800.
235. Lee SS, Lee CK, Wong NS, et al. Low compliance of men having sex with men with self-deferral from blood donation in a Chinese population. Blood Transfus. 2014 Apr;12(2):166-71.
236. Li D, Yang X, Zhang Z, et al. Nitrite Inhalants Use and HIV Infection among Men Who Have Sex with Men in China. BioMed Research International. 2014;2014:365261-365261.
237. Li J, Lau JT, Gu J, et al. Event-specific risk factors predicting episodes of unprotected anal intercourse with male nonregular partners among men who have sex with men using case-crossover study design. Biomed Res Int. 2014;2014:475195.
238. Li X, Lu H, Cox C, et al. Changing the Landscape of the HIV Epidemic among MSM in China: Results from Three Consecutive Respondent-Driven Sampling Surveys from 2009 to 2011. BioMed Research International. 2014;2014:563517-563517.
239. Li X, Wu G, Lu R, et al. HIV-testing behavior and associated factors among MSM in Chongqing, China: results of 2 consecutive cross-sectional surveys from 2009 to 2010. Medicine. 2014;93(27):e124-e124.
240. Li Y-Z, Xu J-J, Qian H-Z, et al. High prevalence of HIV infection and unprotected anal intercourse among older men who have sex with men in China: a systematic review and meta-analysis. BMC Infectious Diseases. 2014 Oct 6;14(1):531-531.
241. Liao M, Kang D, Tao X, et al. Alcohol Use, Stigmatizing/Discriminatory Attitudes, and HIV High-Risk Sexual Behaviors among Men Who Have Sex with Men in China. BioMed Research International. 2014;2014:143738-143738.
242. Lin L, Nehl EJ, Tran A, et al. Sexually transmitted infection testing practices among 'money boys' and general men who have sex with men in Shanghai, China: objective versus self-reported status. Sexual Health. 2014;11(1):94-96.
243. Liu Y, Qian HZ, Ruan Y, et al. Alcohol use among Chinese men who have sex with men: an epidemiological survey and meta-analysis. Biomed Res Int. 2014;2014:414381.
244. Mao H, Ma W, Lu H, et al. High incidence of HIV and syphilis among migrant men who have sex with men in Beijing, China: a prospective cohort study. BMJ Open. 2014 Sep 16;4(9):e005351.
245. Pan SW, Ruan Y, Spittal PM, et al. HIV vulnerabilities and coercive sex at same-sex sexual debut among men who have sex with men in Beijing, China. AIDS care. 2014;26(6):742-749.
246. Pyun T, Santos G-M, Arreola S, et al. Internalized Homophobia and Reduced HIV Testing Among Men Who Have Sex With Men in China. Asia-Pacific Journal of Public Health. 2014 Mar;26(2):118-125.
247. Ruan Y, Wu G, Lu H, et al. Sexual Partnerships with Men and Women Among Men Who Have Sex with Men in Beijing and Chongqing, China, 2010. AIDS & Behavior. 2014 Jan;18(1):180-188.
248. Shiu C-S, Chen Y-C, Tseng P-C, et al. Curvilinear relationship between depression and unprotected sexual behaviors among men who have sex with men. Journal of Sexual Medicine. 2014 Oct;11(10):2466-2473.
249. Tao J, Li MY, Qian HZ, et al. Home-based HIV testing for men who have sex with men in China: a novel community-based partnership to complement government programs. PLoS One. 2014;9(7):e102812.
250. Tsui HY, Lau JT, Feng T, et al. Sexual dysfunction and unprotected anal intercourse among men who have sex with men in two Chinese cities. J Sex Marital Ther. 2014;40(2):139-48.

251. Wang QQ, Chen XS, Yin YP, et al. HIV prevalence, incidence and risk behaviours among men who have sex with men in Yangzhou and Guangzhou, China: a cohort study. J Int AIDS Soc. 2014;17:18849.
252. Wang X, Lan G, Shen Z, et al. HIV and syphilis prevalence trends among men who have sex with men in Guangxi, China: yearly cross-sectional surveys, 2008-2012. BMC Infectious Diseases. 2014;14(1):367-367.
253. Wei C, Muessig KE, Bien C, et al. Strategies for promoting HIV testing uptake: willingness to receive couple-based and collective HIV testing among a cross-sectional online sample of men who have sex with men in China. Sexually Transmitted Infections. 2014;90(6):469-474.
254. Wei C, Yan H, Yang C, et al. Accessing HIV testing and treatment among men who have sex with men in China: A qualitative study. AIDS care. 2014;26(3):372-378.
255. Wu J, Hu Y, Jia Y, et al. Prevalence of unprotected anal intercourse among men who have sex with men in China: an updated meta-analysis. PLoS One. 2014;9(5):e98366.
256. Wu JR, Wang B, Chen LS, et al. Alarming incidence of genital mycoplasmas among HIV-1-infected MSM in Jiangsu, China. Eur J Clin Microbiol Infect Dis. 2014 Feb;33(2):189-95.
257. Xu J-J, Qian H-Z, Chu Z-X, et al. Recreational Drug Use among Chinese Men Who Have Sex with Men: A Risky Combination with Unprotected Sex for Acquiring HIV Infection. BioMed Research International. 2014;2014:725361-725361.
258. Xu JJ, Zhang C, Hu QH, et al. Recreational drug use and risks of HIV and sexually transmitted infections among Chinese men who have sex with men: Mediation through multiple sexual partnerships. BMC Infect Dis. 2014 Dec 2;14:642.
259. Yan H, Wong FY, Zheng T, et al. Social support and depressive symptoms among 'money' boys and general men who have sex with men in Shanghai, China. Sexual Health. 2014 July;11(3):285-287.
260. Yan H, Zhang R, Wei C, et al. A peer-led, community-based rapid HIV testing intervention among untested men who have sex with men in China: an operational model for expansion of HIV testing and linkage to care. Sexually Transmitted Infections. 2014 Aug;90(5):388-393.
261. Yang HT, Tang W, Xiao ZP, et al. Worsening epidemic of HIV and syphilis among men who have sex with men in Jiangsu Province, China. Clin Infect Dis. 2014 Jun;58(12):1753-9.
262. Zeng G, Feng L, Ouyang L, et al. The Dynamic Trends of HIV Prevalence, Risks, and Prevention among Men Who Have Sex with Men in Chongqing, China. BioMed Research International. 2014;2014:602719-602719.
263. Zeng Y, Zhang L, Li T, et al. Risk Factors for HIV/Syphilis Infection and Male Circumcision Practices and Preferences among Men Who Have Sex with Men in China. BioMed Research International. 2014;2014:498987-498987.
264. Zhang DY, Yin YP, Feng TJ, et al. HPV infections among MSM in Shenzhen, China. PLoS One. 2014;9(5):e96364.
265. Zhao J, Chen L, Cai W-D, et al. HIV Infection and Sexual Behaviors Among Non-Commercial Men Who Have Sex with Men at Different Venues. Archives of Sexual Behavior. 2014 May;43(4):801-809.
266. Zheng B-J, Yin Y-P, Han Y, et al. The prevalence of urethral and rectal Mycoplasma genitalium among men who have sex with men in China, a cross-sectional study. BMC public health. 2014;14(1):195-195.
267. Zhong F, Liang B, Xu H, et al. Increasing HIV and decreasing syphilis prevalence in a context

of persistently high unprotected anal intercourse, six consecutive annual surveys among men who have sex with men in Guangzhou, China, 2008 to 2013. PLoS One. 2014;9(7):e103136.
268. Zhou W, Zhao M, Wang X, et al. Treatment adherence and health outcomes in MSM with HIV/AIDS: patients enrolled in "one-stop" and standard care clinics in Wuhan China. PLoS One. 2014;9(12):e113736.
269. Zhou Y, Li D, Lu D, et al. Prevalence of HIV and syphilis infection among men who have sex with men in China: a meta-analysis. Biomed Res Int. 2014;2014:620431.
270. Bien C, Best J, Muessig K, et al. Gay Apps for Seeking Sex Partners in China: Implications for MSM Sexual Health. AIDS & Behavior. 2015 Jun;19(6):941-946.
271. Bien CH, Muessig KE, Lee R, et al. HIV and syphilis testing preferences among men who have sex with men in South China: a qualitative analysis to inform sexual health services. PLoS One. 2015;10(4):e0124161.
272. Cai R, Cai WD, Zhao J, et al. Determinants of recent HIV testing among male sex workers and other men who have sex with men in Shenzhen, China: a cross-sectional study. Sexual Health. 2015 Nov;12(6):565-567.
273. Chen H, Li Y, Wang L, et al. Causes of suicidal behaviors in men who have sex with men in China: a national questionnaire survey. BMC public health. 2015 Feb 7;15:91.
274. Chen JP, Han MM, Liao ZJ, et al. HIV-related behaviors, social support and health-related quality of life among men who have sex with men and women (MSMW): a cross-sectional study in Chongqing, China. PLoS One. 2015;10(2):e0118651.
275. Chen X, Li X, Zheng J, et al. Club drugs and HIV/STD infection: An exploratory analysis among men who have sex with men in Changsha, China. PLoS ONE. 2015 May 07;10(5):9.
276. Chong ES, Zhang Y, Mak WW, et al. Social Media as Social Capital of LGB Individuals in Hong Kong: Its Relations with Group Membership, Stigma, and Mental Well-Being. American Journal of Community Psychology. 2015 Mar;55(1-2):228-238.
277. Chow EP, Chen X, Zhao J, et al. Factors associated with self-reported unprotected anal intercourse among men who have sex with men in Changsha city of Hunan province, China. AIDS care. 2015;27(10):1332-42.
278. Das A, Li J, Zhong F, et al. Factors associated with HIV and syphilis co-infection among men who have sex with men in seven Chinese cities. International Journal of STD & AIDS. 2015 Mar;26(3):145-155.
279. Davis A, Best J, Wei C, et al. Intimate Partner Violence and Correlates With Risk Behaviors and HIV/STI Diagnoses Among Men Who Have Sex With Men and Men Who Have Sex With Men and Women in China: A Hidden Epidemic. Sex Transm Dis. 2015 Jul;42(7):387-92.
280. Fu GF, Jiang N, Hu HY, et al. The epidemic of HIV, syphilis, chlamydia and gonorrhea and the correlates of sexual transmitted infections among men who have sex with men in Jiangsu, China, 2009 [Article]. PLoS ONE. 2015;10(3):e0118863.
281. Gao W, Li Z, Li Y, et al. Sexual Practices and the Prevalence of HIV and Syphilis among Men Who Have Sex with Men in Lanzhou, China. Jpn J Infect Dis. 2015;68(5):370-5.
282. Gu J, Lau JT, Wang Z, et al. Perceived empathy of service providers mediates the association between perceived discrimination and behavioral intention to take up HIV antibody testing again among men who have sex with men. PLoS One. 2015;10(2):e0117376.
283. Hu J, Zhang MY, Ma JJ, et al. CHARACTERISTICS AND FACTORS INFLUENCING UNPROTECTED ANAL INTERCOURSE AMONG MEN WHO HAVE SEX WITH MEN IN

FUYANG, CHINA. Southeast Asian J Trop Med Public Health. 2015 Jul;46(4):680-8.
284. Huan X, Hao C, Yan H, et al. High prevalence of HIV and syphilis among men who have sex with men recruited by respondent-driven sampling in a city in Eastern China. Asia Pac J Public Health. 2015 Mar;27(2):Np854-65.
285. Jia Z, Huang X, Wu H, et al. HIV burden in men who have sex with men: a prospective cohort study 2007-2012. Sci Rep. 2015 Jul 2;5:11205.
286. Lau J, Li D, Wang Z, et al. Repeated HIV Voluntary Counseling and Testing Increased Risk Behaviors Among Men Who Have Sex with Men in China: A Prospective Cohort Study. AIDS & Behavior. 2015;19(11):1966-1977.
287. Leung KK, Poon CM, Lee SS. A comparative analysis of behaviors and sexual affiliation networks among men who have sex with men in Hong Kong. Archives of Sexual Behavior. 2015 Oct;44(7):2067-2076.
288. Li D, Li C, Wang Z, et al. Prevalence and associated factors of unprotected anal intercourse with regular male sex partners among HIV negative men who have sex with men in China: a cross-sectional survey. PLoS One. 2015;10(3):e0119977.
289. Li H, Holroyd E, Lau J. Exploring unprotected anal intercourse among newly diagnosed HIV positive men who have sex with men in China: An ethnographic study. PLoS ONE. 2015 Oct 13;10(10):11.
290. Li H, Holroyd E, Lau J, et al. Stigma, Subsistence, Intimacy, Face, Filial Piety, and Mental Health Problems Among Newly HIV-Diagnosed Men Who Have Sex With Men in China. JANAC: Journal of the Association of Nurses in AIDS Care. 2015;26(4):454-463.
291. Li H, Holroyd E, Li X, et al. A qualitative analysis of barriers to accessing HIV/AIDS-related services among newly diagnosed HIV-positive men who have sex with men in China. International Journal of STD & AIDS. 2015 Jan;26(1):13-19.
292. Liao M, Wang M, Shen X, et al. Bisexual Behaviors, HIV Knowledge, and Stigmatizing/Discriminatory Attitudes among Men Who Have Sex with Men. PLoS One. 2015;10(6):e0130866.
293. Lin H, Ding Y, Liu X, et al. High Prevalence of HIV Infection and Bisexual Networks among a Sample of Men Who Have Sex with Men in Eastern China. PLoS One. 2015;10(6):e0129300.
294. Liu G, Lu H, Wang J, et al. Incidence of HIV and Syphilis among Men Who Have Sex with Men (MSM) in Beijing: An Open Cohort Study. PLoS One. 2015;10(10):e0138232.
295. Liu J, Qu B, Zhu Y, et al. The influence of social support on quality of life of men who have sex with men in China: a preliminary study. PLoS One. 2015;10(5):e0127644.
296. Liu Y, Ruan Y, Vermund SH, et al. Predictors of antiretroviral therapy initiation: a cross-sectional study among Chinese HIV-infected men who have sex with men. BMC Infect Dis. 2015 Dec 15;15:570.
297. Liu Y, Sun X, Qian H-Z, et al. Qualitative Assessment of Barriers and Facilitators of Access to HIV Testing Among Men Who Have Sex with Men in China. AIDS Patient Care & STDs. 2015;29(9):481-489.
298. Ma Q, Xia S, Pan X, et al. Rapid HIV antibody testing among men who have sex with men who visited a gay bathhouse in Hangzhou, China: a cross-sectional study. BMJ Open. 2015 Sep 7;5(9):e008661.
299. Ma Q, Zeng S, Xia S, et al. Risky sexual networks and concentrated HIV epidemics among men who have sex with men in Wenzhou, China: a respondent-driven sampling study. BMC

public health. 2015 Dec 16;15:1246.
300. Mi G, Wu Z, Wang X, et al. Effects of a Quasi-Randomized Web-Based Intervention on Risk Behaviors and Treatment Seeking Among HIV-Positive Men Who Have Sex With Men in Chengdu, China [Journal Article; Randomized Controlled Trial; Research Support, N.I.H., Extramural; Research Support, Non-U.S. Gov't]. Curr Hiv Res. 2015;13(6):490-496.
301. Muessig KE, Bien CH, Wei C, et al. A mixed-methods study on the acceptability of using eHealth for HIV prevention and sexual health care among men who have sex with men in China. Journal of Medical Internet Research. 2015 Apr;17(4):e100.
302. Nehl EJ, He N, Lin L, et al. Drug Use and Sexual Behaviors Among MSM in China. Substance use & misuse. 2015 Jan;50(1):123-136.
303. Pan X, Wu M, Ma Q, et al. High prevalence of HIV among men who have sex with men in Zhejiang, China: a respondent-driven sampling survey. BMJ Open. 2015 Dec 11;5(12):e008466.
304. Qi J, Zhang D, Fu X, et al. High risks of HIV transmission for men who have sex with men--a comparison of risk factors of HIV infection among MSM associated with recruitment channels in 15 cities of China. PLoS One. 2015;10(4):e0121267.
305. Shang H, Zhang L. MSM and HIV-1 infection in China. National Science Review. 2015 Dec;2(4):388-391.
306. Tang W, Mahapatra T, Liu F, et al. Burden of HIV and Syphilis: A Comparative Evaluation between Male Sex Workers and Non-Sex-Worker Men Who Have Sex with Men in Urban China. PLoS One. 2015;10(5):e0126604.
307. Tsai Y-H, Joe S-W, Liu W-T, et al. Modeling job effectiveness in the context of coming out as a sexual minority: A socio-cognitive model. Review of Managerial Science. 2015 Jan;9(1):197-218.
308. Wang H-Y, Xu J-J, Zou H-C, et al. Sexual Risk Behaviors and HIV Infection among Men Who Have Sex with Men and Women in China: Evidence from a Systematic Review and Meta-Analysis. BioMed Research International. 2015;2015:1-12.
309. Wang S, Song D, Huang W, et al. Heterosexual partnerships and the need for HIV prevention and testing for men who have sex with men and women in China: A qualitative study. Aids Educ Prev. 2015 Apr;27(2):126-138.
310. Wang Y, Huang YL, Chen HL, et al. Incidence and correlates of HIV and syphilis in a prospective cohort of men who have sex with men in Mianyang, China, over a 36-month period. Sexual Health. 2015 Nov;12(6):546-555.
311. Wang Z, Li D, Lau JT, et al. Prevalence and associated factors of inhaled nitrites use among men who have sex with men in Beijing, China. Drug Alcohol Depend. 2015 Apr 1;149:93-9.
312. Wong HT, Hoo, Tam HY, et al. Usage and Acceptability of HIV Self-testing in Men who have Sex with Men in Hong Kong. AIDS and Behavior. 2015 Mar;19(3):505-515.
313. Wu Y-L, Yang H-Y, Wang J, et al. Prevalence of suicidal ideation and associated factors among HIV-positive MSM in Anhui, China. International Journal of STD & AIDS. 2015 Jun;26(7):496-503.
314. Yan H, Yang H, Raymond H, et al. Experiences and Correlates of HIV Self-Testing Among Men Who Have Sex with Men in Jiangsu Province, China. AIDS & Behavior. 2015 Mar;19(3):485-491.
315. Yang F, Shi XY, He WH, et al. Factors of the HIV Transmission in Men Who Have Sex with Men in Suizhou City from 2009 to 2013. Sexual Medicine. 2015 Mar;3(1):24-31.

316. Yang Z, Huang Z, Dong Z, et al. Prevalence of high-risky behaviors in transmission of HIV among high school and college student MSM in China: a meta-analysis. BMC public health. 2015 Dec 21;15:1272.
317. Zhang H, Lu H, Pan SW, et al. Correlates of unprotected anal intercourse: the influence of anal sex position among men who have sex with men in beijing, china. Archives of Sexual Behavior. 2015 Feb;44(2):375-387.
318. Zhao J, Cai R, Chen L, et al. A comparison between respondent-driven sampling and time-location sampling among men who have sex with men in Shenzhen, China. Archives of Sexual Behavior. 2015 Oct;44(7):2055-2065.
319. Zhao Y, Zhang L, Zhang H, et al. HIV testing and preventive services accessibility among men who have sex with men at high risk of HIV infection in Beijing, China. Medicine. 2015;94(6):e534-e534.
320. Zheng L, Hart TA, Zheng Y. Top/bottom sexual self-labels and empathizing–systemizing cognitive styles among gay men in China. Archives of Sexual Behavior. 2015 Jul 44(5):1431-1438.
321. Zheng L, Zheng Y. Sex and sexual orientation differences in empathizing-systemizing cognitive styles in China. Personality and Individual Differences. 2015 Dec;87:267-271.
322. Zheng L, Zheng Y. Correlated preferences for male facial masculinity and partner traits in gay and bisexual men in China. Archives of Sexual Behavior. 2015 Jul 2015 2017-09-25;44(5):1423-1430.
323. Chen J, Li X, Xiong Y, et al. Reducing the risk of HIV transmission among men who have sex with men: A feasibility study of the motivational interviewing counseling method. Nursing & Health Sciences. 2016;18(3):400-407.
324. Chen Y-T, Ibragimov U, Nehl EJ, et al. Validity of the CAGE questionnaire for men who have sex with men (MSM) in China. Drug and Alcohol Dependence. 2016 Mar 01;160:151-156.
325. Choi K-H, Steward WT, Miège P, et al. Sexual stigma, coping styles, and psychological distress: A longitudinal study of men who have sex with men in Beijing, China. Archives of Sexual Behavior. 2016 Aug;45(6):1483-1491.
326. Choi SY, Luo M. Performative family: homosexuality, marriage and intergenerational dynamics in China. Br J Sociol. 2016 Jun;67(2):260-80.
327. Chuang D-M, Lacombe-Duncan A. Community engagement among men who have sex with men living with HIV/AIDS in Taiwan. AIDS care. 2016;28(4):445-449.
328. Cunge Z, Jun-jie X, Qing-hai H, et al. Commercial sex and risk of HIV, syphilis, and herpes simplex virus-2 among men who have sex with men in six Chinese cities. BMC Infectious Diseases. 2016;16:1-11.
329. Dai Z, Zhong X, Peng B, et al. Socio-demographics, sexual behaviours, and use of HIV prevention services among men who have sex with men and women in Western China. International Journal of STD & AIDS. 2016 Feb;27(2):133-140.
330. Davis A, Best J, Luo J, et al. Risk behaviours, HIV/STI testing and HIV/STI prevalence between men who have sex with men and men who have sex with both men and women in China. International Journal of STD & AIDS. 2016 Sep;27(10):840-849.
331. Ding Y, Yan H, Ning Z, et al. Low willingness and actual uptake of pre-exposure prophylaxis for HIV-1 prevention among men who have sex with men in Shanghai, China. Biosci Trends. 2016 May 23;10(2):113-9.

332. Fan W, Lu R, Wu G, et al. Alcohol drinking and HIV-related risk among men who have sex with men in Chongqing, China. Alcohol. 2016 Feb;50:1-7.
333. Fu X, Qi J, Hu Y, et al. Partner notification in cooperation with community-based organizations among HIV-positive men who have sex with men in two Chinese cities. International Journal of STD & AIDS. 2016;27(10):821-831.
334. Geng GZ, Gao G, Ruan YH, et al. Behavioral Risk Profile of Men Who Have Sex with Men in Beijing, China: Results from a Cross-sectional Survey with Randomized Response Techniques. Chin Med J (Engl). 2016 Mar 5;129(5):523-9.
335. Hidru TH, Wang F, Lolokote S, et al. Associated factors of self-reported psychopathology and health related quality of life among men who have sex with men (MSM) with HIV/AIDS in Dalian, China: a pilot study. Infect Dis Poverty. 2016 Dec 1;5(1):108.
336. Hu H, Liu X, Zhang Z, et al. Increasing HIV Incidence among Men Who Have Sex with Men in Jiangsu Province, China: Results from Five Consecutive Surveys, 2011-2015. International journal of environmental research and public health. 2016 Aug 6;13(8).
337. Hu J, Hu J, Huang G, et al. Life satisfaction, self-esteem, and loneliness among LGB adults and heterosexual adults in China. Journal of Homosexuality. 2016 Jan;63(1):72-86.
338. Huang Y, Zhang Y, Li K, et al. Changes in Prevalence of HIV or Syphilis among Male Sex Workers and Non-Commercial Men Who Have Sex with Men in Shenzhen, China: Results of a Second Survey. PLoS One. 2016;11(12):e0167619.
339. Ko N-Y, Chen B-J, Li C-W, et al. Willingness to Self-Pay for Pre-exposure Prophylaxis in Men Who Have Sex With Men: A National Online Survey in Taiwan. AIDS Education & Prevention. 2016 Apr;28(2):128-137.
340. Ko N-Y, Tseng P-C, Huang Y-C, et al. Seeking sex partners through the internet and mobile phone applications among men who have sex with men in Taiwan. AIDS care. 2016 Jul;28(7):927-931.
341. Lau J, Lee A, Tse W, et al. A Randomized Control Trial for Evaluating Efficacies of Two Online Cognitive Interventions With and Without Fear-Appeal Imagery Approaches in Preventing Unprotected Anal Sex Among Chinese Men Who Have Sex with Men. AIDS & Behavior. 2016 Sep;20(9):1851-1862.
342. Lau JT, Mo PK, Gu J, et al. Association of Situational and Environmental Factors With Last Episode of Unprotected Anal Intercourse Among MSM in Hong Kong: A Case-Crossover Analysis. Aids Educ Prev. 2016 Feb;28(1):26-42.
343. Li D, Yang X, Zhang Z, et al. Incidence of Co-Infections of HIV, Herpes Simplex Virus Type 2 and Syphilis in a Large Cohort of Men Who Have Sex with Men in Beijing, China. PLoS One. 2016;11(1):e0147422.
344. Li J, Mo PK, Kahler CW, et al. Prevalence and associated factors of depressive and anxiety symptoms among HIV-infected men who have sex with men in China. AIDS care. 2016;28(4):465-70.
345. Li R, Cai Y, Wang Y, et al. Psychological pathway to suicidal ideation among men who have sex with men in Shanghai, China: A structural equation model. J Psychiatr Res. 2016 Dec;83:203-210.
346. Li R, Cai Y, Wang Y, et al. Psychosocial syndemic associated with increased suicidal ideation among men who have sex with men in Shanghai, China. Health Psychology. 2016 Feb;35(2):148-156.

347. Li R, Pan X, Ma Q, et al. Prevalence of prior HIV testing and associated factors among MSM in Zhejiang Province, China: a cross-sectional study. BMC public health. 2016;16(1):1152-1152.
348. Li X, Lei Y, Wang H, et al. The Health Belief Model: A Qualitative Study to Understand High-risk Sexual Behavior in Chinese Men Who Have Sex With Men. JANAC: Journal of the Association of Nurses in AIDS Care. 2016 Jan-Feb;27(1):66-76.
349. Li X, Li M, Yang Y, et al. Anal HPV/HIV co-infection among Men Who Have Sex with Men: a cross-sectional survey from three cities in China. Sci Rep. 2016 Feb 19;6:21368.
350. Li Z, Hsieh E, Morano JP, et al. Exploring HIV-related stigma among HIV-infected men who have sex with men in Beijing, China: a correlation study. AIDS care. 2016;28(11):1394-1401.
351. Lin X, Chi P, Zhang L, et al. Disclosure of HIV Serostatus and Sexual Orientation Among HIV-Positive Men Who Have Sex with Men in China. Community Mental Health Journal. 2016;52(4):457-465.
352. Liu Y, Li D, Vermund SH, et al. Associations of current marital status and living arrangements with HIV and syphilis risk: findings from a community-based sample of men who have sex with men in China. AIDS care. 2016;28(11):1461-1466.
353. Liu Y, Osborn CY, Qian HZ, et al. Barriers and Facilitators of Linkage to and Engagement in HIV Care Among HIV-Positive Men Who Have Sex with Men in China: A Qualitative Study. AIDS Patient Care STDS. 2016 Feb;30(2):70-7.
354. Liu Y, Qian H-Z, Ruan Y, et al. Frequent HIV testing: Impact on HIV risk among Chinese men who have sex with men. JAIDS Journal of Acquired Immune Deficiency Syndromes. 2016 Aug 01;72(4):452-461.
355. Liu Y, Ruan Y, Strauss SM, et al. Alcohol misuse, risky sexual behaviors, and HIV or syphilis infections among Chinese men who have sex with men. Drug & Alcohol Dependence. 2016 Nov 1;168:239-246.
356. Liu Y, Wang J, Qian H-Z, et al. Seeking Male Sexual Partners via Internet and Traditional Venues among Chinese Men Who Have Sex with Men: Implications for HIV Risk Reduction Interventions. AIDS & Behavior. 2016 Oct;20(10):2222-2230.
357. Liu Y-Y, Tao H-D, Liu J, et al. Prevalence and associated factors of HIV infection among men who have sex with men in Hefei, China, 2013-2014: a cross-sectional study. International Journal of STD & AIDS. 2016 Mar;27(3):305-312.
358. Liu YY, Wang Z, Xu JT, et al. Associations between recent gay-related stressful events, emotional distress, social support and unprotected anal intercourse behavior among Chinese men who have sex with men. Australian and New Zealand Journal of Psychiatry. 2016 Jul;50(7):659-666.
359. Lo IP, Chan CH, Chan TH. Perceived importance of childbearing and attitudes toward assisted reproductive technology among Chinese lesbians in Hong Kong: implications for psychological well-being. Fertil Steril. 2016 Oct;106(5):1221-1229.
360. Ma WZ, Wu GH, Zheng H, et al. Prevalence and risk factors of HIV and syphilis, and knowledge and risk behaviors related to HIV/AIDS among men who have sex with men in Chongqing, China. Journal of Biomedical Research. 2016;30(2):101-111.
361. Mu H, Li Y, Liu L, et al. Prevalence and risk factors for lifetime suicide ideation, plan and attempt in Chinese men who have sex with men. BMC Psychiatry. 2016 Dec;16:10.
362. Pan S, Xu J-J, Han X-X, et al. Internet-Based Sex-Seeking Behavior Promotes HIV Infection Risk: A 6-Year Serial Cross-Sectional Survey to MSM in Shenyang, China. BioMed Research

International. 2016;2016:1-11.
363. Pan SW, Zhang Z, Li D, et al. Religion and HIV sexual risk among men who have sex with men in China. JAIDS Journal of Acquired Immune Deficiency Syndromes. 2016 Dec 01;73(4):463-474.
364. Qian HZ, Ruan Y, Liu Y, et al. Lower HIV Risk Among Circumcised Men Who Have Sex With Men in China: Interaction With Anal Sex Role in a Cross-Sectional Study. J Acquir Immune Defic Syndr. 2016 Apr 1;71(4):444-51.
365. Qin Q, Tang W, Ge L, et al. Changing trend of HIV, Syphilis and Hepatitis C among Men Who Have Sex with Men in China. Sci Rep. 2016 Aug 18;6:31081.
366. Qu L, Wang W, Gao Y, et al. A Cross-sectional Survey of HIV Transmission and Behavior among Men Who Have Sex with Men in Different Areas of Inner Mongolia Autonomous Region, China. BMC public health. 2016 Nov 15;16(1):1161.
367. Shen H, Tang S, Mahapatra T, et al. Condomless Vaginal Intercourse and Its Associates among Men Who Have Sex with Men in China. PLoS One. 2016;11(4):e0154132.
368. Shi L-e, Wei C, McFarland W, et al. Comparing samples of men who have sex with men recruited online and in venues, Jiangsu Province, China, 2013. LGBT Health. 2016 Jun;3(3):238-242.
369. Shieh W-Y. Why same-sex couples break up: A follow-up study in Taiwan. Journal of GLBT Family Studies. 2016 May;12(3):257-276.
370. Song B, Yan C, Lin Y, et al. Health-Related Quality of Life in HIV-Infected Men Who Have Sex with Men in China: A Cross-Sectional Study. Med Sci Monit. 2016 Aug 14;22:2859-70.
371. Tang S, Tang W, Meyers K, et al. HIV epidemiology and responses among men who have sex with men and transgender individuals in China: a scoping review. BMC Infect Dis. 2016 Oct 20;16(1):588.
372. Tang W, Best J, Zhang Y, et al. Gay mobile apps and the evolving virtual risk environment: A cross-sectional online survey among men who have sex with men in China [Article]. Sexually Transmitted Infections. 2016 Nov;92(7):508-514.
373. Tang W, Han L, Best J, et al. Crowdsourcing HIV Test Promotion Videos: a Noninferiority Randomized Controlled Trial in China [Journal Article; Randomized Controlled Trial; Research Support, N.I.H., Extramural]. Clinical infectious diseases. 2016;62(11):1436-1442.
374. Tang W, Tang S, Qin Y, et al. Will Gay Sex-Seeking Mobile Phone Applications Facilitate Group Sex? A Cross-Sectional Online Survey among Men Who Have Sex with Men in China. PLoS One. 2016;11(11):e0167238.
375. Wang AL, Peng RR, Tucker JD, et al. Optimizing Partner Notification Programs for Men Who Have Sex with Men: Factorial Survey Results from South China. PLoS One. 2016;11(7):e0157749.
376. Wang N, Wu G, Lu R, et al. Investigating HIV Infection and HIV Incidence Among Chinese Men Who Have Sex with Men with Recent Sexual Debut, Chongqing, China, 2011. AIDS & Behavior. 2016 Dec;20(12):2976-2982.
377. Wang Q, Ding H, Xu J, et al. Lipids profile among ART-naive HIV infected patients and men who have sex with men in China: a case control study. Lipids Health Dis. 2016 Sep 6;15(1):149.
378. Wei C, Cheung DH, Yan H, et al. The impact of homophobia and HIV stigma on HIV testing uptake among Chinese men who have sex with men: A mediation analysis. JAIDS Journal of Acquired Immune Deficiency Syndromes. 2016 Jan 01;71(1):87-93.

379. Wei C, Yan H, Raymond H, et al. HIV Testing and Awareness of Partner's HIV Status Among Chinese Men Who Have Sex with Men in Main Partnerships. AIDS & Behavior. 2016 Apr;20(4):833-840.
380. Wu J, Wu H, Li PS, et al. HIV/STIs risks between migrant MSM and local MSM: across-sectional comparison study in China. Peerj. 2016 Jul;4.
381. Xu JJ, Tang WM, Zou HC, et al. High HIV incidence epidemic among men who have sex with men in china: results from a multi-site cross-sectional study. Infect Dis Poverty. 2016 Sep 5;5(1):82.
382. Xu R, Dai W, Zhao G, et al. Early Sexual Debut and HIV Infection among Men Who Have Sex with Men in Shenzhen, China. BioMed Research International. 2016;2016:1-8.
383. Xu W, Zheng L, Liu Y, et al. Sexual sensation seeking, sexual compulsivity, and high-risk sexual behaviours among gay/bisexual men in Southwest China. AIDS care. 2016 Sep;28(9):1138-1144.
384. Yan H, Li J, Raymond HF, et al. Increased HIV Testing among Men Who Have Sex with Men from 2008 to 2012, Nanjing, China. PLoS One. 2016;11(4):e0154466.
385. Yang Z, Huang Z, Dong Z, et al. Risk Factors for HIV Diagnosis Among Men Who Have Sex with Men: Results of a Case-Control Study in One Sample of Eastern China. AIDS Res Hum Retroviruses. 2016 Dec;32(12):1163-1168.
386. Yaxin Z, Jie L, Bo Q, et al. Relationship between quality of life and unprotected anal intercourse among Chinese men who have sex with men: a cross-sectional study. BMC public health. 2016;16(1):1-6.
387. Yeo TE, Fung TH. Between '0' and '1': safer sex and condom use among young gay men in Hong Kong. Cult Health Sex. 2016;18(3):294-307.
388. Yeo TE, Ng YL. Sexual risk behaviors among apps-using young men who have sex with men in Hong Kong. AIDS care. 2016;28(3):314-8.
389. Zeng X, Zhong X, Peng B, et al. Prevalence and associated risk characteristics of HIV infection based on anal sexual role among men who have sex with men: a multi-city cross-sectional study in Western China. Int J Infect Dis. 2016 Aug;49:111-8.
390. Zhang C, Qian H-Z, Yin L, et al. Sexual Behaviors Linked to Drug and Alcohol Use Among Men Who Have Sex With Men in China. Substance use & misuse. 2016 Dec 5;51(14):1821-1830.
391. Zhang G, Zhang H, Yu H, et al. Hypothetical rectal microbicide acceptability and factors influencing it among men who have sex with men in Tianjin, China. PLoS ONE. 2016 May 31;11(5):16.
392. Zhang H, Teng T, Lu H, et al. Poppers use and risky sexual behaviors among men who have sex with men in Beijing, China. Drug and Alcohol Dependence. 2016 Mar 01;160:42-48.
393. Zhang S-H, Liu S-J, Hu L-L, et al. The prevalence and correlates of syphilis and HIV among homosexual and bisexual men in Shijiazhuang, China. International Journal of STD & AIDS. 2016 Feb;27(2):127-132.
394. Zhang W, Xu JJ, Zou H, et al. HIV incidence and associated risk factors in men who have sex with men in Mainland China: An updated systematic review and meta-analysis [Review]. Sexual Health. 2016 May 26;13(4):373-382.
395. Zhao J, Chen L, Chaillon A, et al. The dynamics of the HIV epidemic among men who have sex with men (MSM) from 2005 to 2012 in Shenzhen, China. Sci Rep. 2016 Jun 29;6:28703.

396. Zhao Y, Ma Y, Chen R, et al. Non-disclosure of Sexual Orientation to Parents Associated with Sexual Risk Behaviors Among Gay and Bisexual MSM in China. AIDS and Behavior. 2016 Jan;20(1):193-203.
397. Zou H, Xu J, Hu Q, et al. Decreasing age at first anal intercourse among men who have sex with men in China: a multicentre cross-sectional survey. J Int AIDS Soc. 2016;19(1):20792.
398. Bolin C, Chuncheng L, Durvasula M, et al. Social Media Engagement and HIV Testing Among Men Who Have Sex With Men in China: A Nationwide Cross-Sectional Survey. Journal of Medical Internet Research. 2017 Jul 19;19(7):1-13.
399. Cao B, Liu C, Stein G, et al. Faster and Riskier? Online Context of Sex Seeking Among Men Who Have Sex With Men in China. Sex Transm Dis. 2017 Apr;44(4):239-244.
400. Chen G, Cao Y, Yao Y, et al. Syphilis incidence among men who have sex with men in China: results from a meta-analysis. International Journal of STD & AIDS. 2017;28(2):170-178.
401. Chen Y, Tang W, Chen L, et al. Changing Epidemic of HIV and Syphilis Among Resident and Migrant Men Who Have Sex with Men in Jiangsu, China. Sci Rep. 2017 Aug 25;7(1):9478.
402. Choi K-H, Steward WT, Miège P, et al. Unpacking the influence of sexual stigma on HIV risk: Results from a prospective study of men who have sex with men in Beijing, China. JAIDS Journal of Acquired Immune Deficiency Syndromes. 2017 Feb 01;74(2):e38-e44.
403. Chong ES, Mak WW, Tam TC, et al. Impact of perceived HIV stigma within men who have sex with men community on mental health of seropositive MSM. AIDS care. 2017 Jan;29(1):118-124.
404. Dai W, Luo Z, Xu R, et al. Prevalence of HIV and syphilis co-infection and associated factors among non-commercial men who have sex with men attending a sexually transmitted disease clinic in Shenzhen, China. BMC Infect Dis. 2017 Jan 18;17(1):86.
405. Duan C, Wei L, Cai Y, et al. Recreational drug use and risk of HIV infection among men who have sex with men: A cross-sectional study in Shenzhen, China. Drug & Alcohol Dependence. 2017 Dec 1;181:30-36.
406. Gao M, Xiao C, Cao Y, et al. Associations between sexual sensation seeking and AIDS-related knowledge, attitudes and behaviors among young men who have sex with men in China. Psychology, health & medicine. 2017 Jun;22(5):596-603.
407. Han L, Wei C, Muessig KE, et al. HIV test uptake among MSM in China: Implications for enhanced HIV test promotion campaigns among key populations. Global Public Health. 2017;12(1):31-44.
408. Han-Zhu Q, Yifei H, Carlucci JG, et al. Human Immunodeficiency Virus Status Differentially Associated With Genital and Anal Human Papillomavirus Infection Among Chinese Men Who Have Sex With Men: A Cross-Sectional Survey. Sexually Transmitted Diseases. 2017 Nov;44(11):656-662.
409. Haochu L, Chongyi W, Tucker J, et al. Barriers and facilitators of linkage to HIV care among HIV-infected young Chinese men who have sex with men: a qualitative study. Bmc Health Serv Res. 2017;17:1-8.
410. Haochu L, Li X, Tucker JD, et al. Condom use peer norms and self-efficacy as mediators between community engagement and condom use among Chinese men who have sex with men. BMC public health. 2017;17:1-8.
411. He H, Lv F, Zhang NN, et al. Look into the HIV Epidemic of Gay Community with a Socio-Cultural Perspective: A Qualitative Study in China, 2015-2016. PLoS One.

2017;12(1):e0170457.
412. Hu H, Yan H, Liu X, et al. Trends in late HIV diagnosis among men who have sex with men in Jiangsu province, China: Results from four consecutive community-based surveys, 2011-2014. PLoS One. 2017;12(3):e0172664.
413. Hu J, Gu X, Tao X, et al. Prevalence and Trends of HIV, Syphilis, and HCV in Migrant and Resident Men Who Have Sex with Men in Shandong, China: Results from a Serial Cross-Sectional Study. PLoS One. 2017;12(1):e0170443.
414. Ibragimov U, Harnisch JA, Nehl EJ, et al. Estimating self-reported sex practices, drug use, depression, and intimate partner violence among MSM in China: a comparison of three recruitment methods. AIDS care. 2017 Jan;29(1):125-131.
415. Kipp A, Qian H-Z, Vermund S, et al. Relationship of Stigma and Depression Among Newly HIV-Diagnosed Chinese Men Who Have Sex with Men. AIDS & Behavior. 2017 Jan;21(1):292-299.
416. Li H, Holroyd E, Zhang J, et al. Suicidal Ideation, Resilience, and Healthcare Implications for Newly Diagnosed HIV-Positive Men Who Have Sex with Men in China: A Qualitative Study. Archives of Sexual Behavior. 2017 May;46(4):1025-1034.
417. Li H, Sankar A, Holroyd E, et al. Safer sex practices among newly diagnosed HIV-positive men who have sex with men in China: results from an ethnographic study. International Journal of Qualitative Studies on Health & Well-Being. 2017;12(1):N.PAG-N.PAG.
418. Li J, Mo PK, Wu AM, et al. Roles of Self-Stigma, Social Support, and Positive and Negative Affects as Determinants of Depressive Symptoms Among HIV Infected Men who have Sex with Men in China. AIDS and Behavior. 2017 Jan;21(1):261-273.
419. Li R, Wang H, Pan X, et al. Prevalence of condomless anal intercourse and recent HIV testing and their associated factors among men who have sex with men in Hangzhou, China: A respondent-driven sampling survey. PLoS One. 2017;12(3):e0167730.
420. Liu Y, Qian H-Z, Amico K, et al. Subsequent Sexual Risks Among Men Who Have Sex with Men May Differ by Sex of First Partner and Age at Sexual Debut: A Cross-Sectional Study in Beijing, China. AIDS & Behavior. 2017;21(10):2913-2923.
421. Ngai Sze W, Weiming T, Larry H, et al. MSM HIV testing following an online testing intervention in China. BMC Infectious Diseases. 2017;17:1-7.
422. Qin Y, Tang W, Nowacki A, et al. Benefits and Potential Harms of Human Immunodeficiency Virus Self-Testing Among Men Who Have Sex With Men in China: An Implementation Perspective. Sex Transm Dis. 2017 Apr;44(4):233-238.
423. Ren X, Ke W, Zheng H, et al. Human Papillomavirus Positivity in the Anal Canal in HIV-Infected and HIV-Uninfected Men Who Have Anal Sex with Men in Guangzhou, China: Implication for Anal Exams and Early Vaccination. Biomed Res Int. 2017;2017:2641259.
424. Ren XL, Wu ZY, Mi GD, et al. Uptake of HIV Self-testing among Men Who have Sex with Men in Beijing, China: a Cross-sectional Study. Biomed Environ Sci. 2017 Jun;30(6):407-417.
425. Ren XL, Wu ZY, Mi GD, et al. HIV care-seeking behaviour after HIV self-testing among men who have sex with men in Beijing, China: a cross-sectional study. Infect Dis Poverty. 2017 Jun 28;6(1):112.
426. Shen L, Liu X, Fu G, et al. The Epidemic of Human Immunodeficiency Virus, Hepatitis C Virus, and Syphilis Infection, and the Correlates of Sexually Transmitted Infections among Men Who Have Sex with Men in Zhenjiang, Jiangsu, China. Jpn J Infect Dis. 2017 Mar

24;70(2):171-176.
427. Tang DT-S. All I get is an emoji: dating on lesbian mobile phone app Butterfly. Media, Culture & Society. 2017 Sep;39(6):816-832.
428. Tang W, Mao J, Tang S, et al. Disclosure of sexual orientation to health professionals in China: results from an online cross-sectional study. J Int AIDS Soc. 2017 Feb 6;20(1):21416.
429. Tao J, Qian HZ, Kipp AM, et al. Effects of depression and anxiety on antiretroviral therapy adherence among newly diagnosed HIV-infected Chinese MSM [Clinical Trial, Phase I; Clinical Trial, Phase II; Journal Article; Randomized Controlled Trial; Research Support, N.I.H., Extramural]. AIDS. 2017 Jan 28;31(3):401-406.
430. Tao J, Vermund SH, Lu H, et al. Impact of Depression and Anxiety on Initiation of Antiretroviral Therapy Among Men Who Have Sex with Men with Newly Diagnosed HIV Infections in China. AIDS Patient Care & STDs. 2017;31(2):96-104.
431. Tian T, Mijiti P, Bingxue H, et al. Prevalence and risk factors of anal human papillomavirus infection among HIV-negative men who have sex with men in Urumqi city of Xinjiang Uyghur Autonomous Region, China. PLoS One. 2017;12(11):e0187928.
432. Wang X, Li Y, Wu Z, et al. Nitrite Inhalant Use and HIV Infection Among Chinese Men Who Have Sex With Men in 2 Large Cities in China. J Addict Med. 2017 Nov/Dec;11(6):468-474.
433. Wang Y, Wang Z, Jia M, et al. Association between a syndemic of psychosocial problems and unprotected anal intercourse among men who have sex with men in Shanghai, China. BMC Infect Dis. 2017 Jan 7;17(1):46.
434. Wang Y-C, Griffiths J, Grande G. Factors associated with Taiwanese lesbians' breast health-care behavior and intentions: Qualitative interview findings. Women & Health. 2017 Sep;57(8):962-975.
435. Wang Z, Wu X, Lau J, et al. Prevalence of and factors associated with unprotected anal intercourse with regular and nonregular male sexual partners among newly diagnosed HIV-positive men who have sex with men in China. HIV Med. 2017 Oct;18(9):635-646.
436. Wenjian X, Lijun Z, Yin X, et al. Internalized homophobia, mental health, sexual behaviors, and outness of gay/bisexual men from Southwest China. International Journal for Equity in Health. 2017;16:1-10.
437. Xian Y, Zhu B, Zhang X, et al. Risk factors associated with sexually transmitted infections among HIV infected men who have sex with men. PLoS One. 2017;12(2):e0170635.
438. Xiao C, Wang W, Cao Y, et al. The influence of condom use during the first-time anal intercourse on the subsequent sexual behaviors among young men who have sex with men in China [Article]. Biomedical Research (India). 2017;28(19):8537-8541.
439. Xin HN, Li XW, Zhang L, et al. Tuberculosis infection testing in HIV-positive men who have sex with men from Xi'an China. Epidemiol Infect. 2017 Feb;145(3):498-502.
440. Xu JJ, Yu YQ, Hu QH, et al. Treatment-seeking behaviour and barriers to service access for sexually transmitted diseases among men who have sex with men in China: a multicentre cross-sectional survey. Infect Dis Poverty. 2017 Jan 18;6(1):15.
441. Xu W, Zheng L, Zheng Y. Prevalence of non-contact and contact childhood sexual abuse: An Internet-based sample of men who have sex with men in China. PLoS One. 2017;12(4):e0175444.
442. Xu X, Sheng Y, Khoshnood K, et al. Factors Predicting Internalized Stigma Among Men Who Have Sex with Men Living with HIV in Beijing, China. JANAC: Journal of the Association of

Nurses in AIDS Care. 2017 Jan - Feb;28(1):142-153.
443. Xu Y, Zheng Y, Rahman Q. The relationship between self-reported sexually explicit media consumption and sexual risk behaviors among men who have sex with men in China. Journal of Sexual Medicine. 2017 Mar;14(3):357-365.
444. Yang X, Mak WW, Ho CY, et al. Self-in-love versus self-in-stigma: implications of relationship quality and love attitudes on self-stigma and mental health among HIV-positive men having sex with men. AIDS care. 2017 Jan;29(1):132-136.
445. Zhang C, Liu Y, Sun X, et al. Substance use and HIV-risk behaviors among HIV-positive men who have sex with men in China: repeated measures in a cohort study design. AIDS care. 2017;29(5):644-653.
446. Zhang J, Zheng L, Zheng Y. Moral and sexual disgust suppress sexual risk behaviors among men who have sex with men in China. Frontiers in Psychology. 2017 Jan 09;7:8.
447. Zhang TP, Liu C, Han L, et al. Community engagement in sexual health and uptake of HIV testing and syphilis testing among MSM in China: a cross-sectional online survey. J Int AIDS Soc. 2017 Apr 3;20(1):21372.
448. Zhang X, Jia M, Chen M, et al. Prevalence and the associated risk factors of HIV, STIs and HBV among men who have sex with men in Kunming, China. Int J STD AIDS. 2017 Oct;28(11):1115-1123.
449. Zhang Z, Zhang L, Zhou F, et al. Knowledge, attitude, and status of nitrite inhalant use among men who have sex with men in Tianjin, China. BMC public health. 2017 Sep 4;17(1):690.
450. Zhao P, Tang S, Wang C, et al. Recreational drug use among Chinese MSM and transgender individuals: Results from a national online cross-sectional study. PLoS ONE. 2017 Jan 20;12(1):11.
451. Zheng L, Su Y, Zheng Y. The Intersection of Gender and Sexuality: Sexism Shapes Men's Same-Sex Sexuality in Terms of Self-Label Identification and Partner Choice in China. Sex Roles. 2017 Jul;77(1-2):125-134.
452. Zheng L, Zheng Y. Sexual satisfaction in Chinese gay and bisexual men: relationship to negative sexual minority identity and sexual role preference. Sexual & Relationship Therapy. 2017;32(1):75-88.
453. Zhong F, Tang W, Cheng W, et al. Acceptability and feasibility of a social entrepreneurship testing model to promote HIV self-testing and linkage to care among men who have sex with men. HIV Medicine. 2017;18(5):376-382.
454. Cao B, Bien C, Pan S, et al. Linking young men who have sex with men (YMSM) to STI physicians: a nationwide cross-sectional survey in China. BMC Infectious Diseases. 2018;18(1):N.PAG-N.PAG.
455. Chen H, Yang Y, Huang Y, et al. Prevalence of poppers use and its sexual risks among men who have sex with men in southwestern China: a cross-sectional study. BMC public health. 2018 Sep 10;18(1):1103.
456. Chen L, Lian D, Wang B. Factors associated with disclosing men who have sex with men (MSM) sexual behaviors and HIV-positive status: A study based on a social network analysis in Nanjing, China. PLoS ONE. 2018 Apr 19;13(4):11.
457. Chen Q, Sun Y, Sun W, et al. Trends of HIV incidence and prevalence among men who have sex with men in Beijing, China: Nine consecutive cross-sectional surveys, 2008-2016. PLoS One. 2018;13(8):e0201953.

458. Cheng FK. Dilemmas of Chinese lesbian youths in contemporary mainland China. Sexuality & Culture. 2018 Mar;22(1):190-208.
459. Chia-Chun L, Ming-Chang H, Hung-Chang H, et al. Human papillomavirus prevalence and behavioral risk factors among HIV-infected and HIV-uninfected men who have sex with men in Taiwan. Medicine. 2018 Nov;97(45):1-9.
460. Chu J-H, Huang J-H. A Theory-Based Exploration of Condomless Anal Intercourse Intention Among Young Men Who Have Sex with Men of Different Sexual Roles in Taiwan. Archives of Sexual Behavior. 2018;47(7):2041-2050.
461. Chu ZX, Xu JJ, Zhang YH, et al. Poppers use and Sexual Partner Concurrency Increase the HIV Incidence of MSM: a 24-month Prospective Cohort Survey in Shenyang, China. Sci Rep. 2018 Jan 8;8(1):24.
462. Chuang D-M, Newman PA. Pre-Exposure Prophylaxis (PrEP) Awareness and Acceptability Among Men Who Have Sex With Men in Taiwan. AIDS Education & Prevention. 2018 Dec;30(6):490-501.
463. Chuang D-M, Newman PA, Li AT-W. Syndemic factors and HIV infection among men who have sex with men in Taiwan. Journal of HIV/AIDS & Social Services. 2018;17(4):337-352.
464. Ding Y, Zhou Y, Liu C, et al. Sex with older partners, condomless anal sex and unrecognized HIV infection among Chinese men who have sex with men. AIDS care. 2018 Mar;30(3):306-311.
465. Du M, Zhao J, Zhang J, et al. Depression and social support mediate the effect of HIV self-stigma on condom use intentions among Chinese HIV-infected men who have sex with men. AIDS care. 2018 Sep;30(9):1197-1206.
466. Fitzpatrick T, Pan SW, Tang W, et al. HBV and HCV test uptake and correlates among men who have sex with men in China: a nationwide cross-sectional online survey. Sex Transm Infect. 2018 Nov;94(7):502-507.
467. Group SS, Ong J, Pan SW, et al. Receiving HIV Serostatus Disclosure from Partners Before Sex: Results from an Online Survey of Chinese Men Who Have Sex with Men. AIDS & Behavior. 2018;22(12):3826-3835.
468. Guanghua L, Yi C, Shuai T, et al. HIV, syphilis and behavioral risk factors among men who have sex with men in a drug-using area of southwestern China: Results of 3 cross-sectional surveys from 2013 to 2015. Medicine (Baltimore). 2018 Apr;97(16):e0404.
469. He J, Xu H-f, Cheng W-b, et al. Intimate relationship characteristics as determinants of HIV risk among men who have sex with regular male sex partners: a cross-sectional study in Guangzhou, China. BMC Infectious Diseases. 2018;18(1):1-1.
470. He L, Pan X, Wang N, et al. New types of drug use and risks of drug use among men who have sex with men: a cross-sectional study in Hangzhou, China. BMC Infectious Diseases. 2018;18(1):N.PAG-N.PAG.
471. Hong H, Xu J, McGoogan J, et al. Relationship between the use of gay mobile phone applications and HIV infection among men who have sex with men in Ningbo, China: a cross-sectional study. International Journal of STD & AIDS. 2018 Apr;29(5):491-497.
472. Hu Y, Zhong X-n, Peng B, et al. Associations between perceived barriers and benefits of using HIV pre-exposure prophylaxis and medication adherence among men who have sex with men in Western China. BMC Infectious Diseases. 2018;18(1):1-8.
473. Jhang J. Scaffolding in Family Relationships: A Grounded Theory of Coming Out to Family.

Family Relations. 2018 Feb;67(1):161-175.
474. Jianmin S, Chang ES, Chuansheng C. The Relative Importance of Parent-Child Dynamics and Minority Stress on the Psychological Adjustment of LGBs in China. Journal of Counseling Psychology. 2018 Oct;65(5):598-604.
475. Junjie X, Huan Y, Weiming T, et al. The Effect of Using Geosocial Networking Apps on the HIV Incidence Rate Among Men Who Have Sex With Men: Eighteen-Month Prospective Cohort Study in Shenyang, China. Journal of Medical Internet Research. 2018 Dec 21;20(12):29-29.
476. Kwan N, Wong A, Fang Y, et al. 'Get an early check - Chrysanthemum tea': An outcome evaluation of a multimedia campaign promoting HIV testing among men who have sex with men in Hong Kong. HIV Med. 2018 May;19(5):347-354.
477. Kwan TH, Lee SS. Predictors of HIV testing and their influence on PrEP acceptance in men who have sex with men: A cross-sectional study. AIDS and Behavior. 2018 Apr;22(4):1150-1157.
478. Lau JTF, Wu X, Wu AMS, et al. Relationships Between Illness Perception and Post-traumatic Growth Among Newly Diagnosed HIV-Positive Men Who have Sex with Men in China. AIDS & Behavior. 2018;22(6):1885-1898.
479. Lei Y, Zhang K, Xiao X, et al. Sexual Roles, Risk Sexual Behaviours, and HIV Prevalence among Men who Have Sex with Men Seeking HIV Testing in Changsha, China. Curr Hiv Res. 2018;16(2):174-181.
480. Li M-J, Huang J-H. Healthcare Seeking Intention if Diagnosed with HIV Among Young MSM in Taiwan: A Theory-Based Comparison by Voluntary Counseling and Testing Experience. AIDS & Behavior. 2018 Aug;22(8):2480-2490.
481. Lian Y, Zhao Y, Wang J, et al. A health communication intervention to integrate partner testing with antiretroviral therapy service among men who have sex with men in China: an observational cohort study. BMC public health. 2018;18(1):N.PAG-N.PAG.
482. Lin HC, Hu HF, Chen MH, et al. Persistent and multisite homophobic harassment during childhood and adolescence and its association with school difficulties in gay and bisexual men in Taiwan. Archives of Clinical Psychiatry. 2018 Jul-Aug;45(4):94-99.
483. Liu C, Ding Y, Ning Z, et al. Factors influencing uptake of pre-exposure prophylaxis: some qualitative insights from an intervention study of men who have sex with men in China. Sexual Health. 2018 Feb;15(1):39-45.
484. Liu C, Fu R, Tang W, et al. Transplantation or rurality? Migration and HIV risk among Chinese men who have sex with men in the urban areas. J Int AIDS Soc. 2018 Jan;21(1).
485. Liu J, Yi Z, Zhao Y, et al. The psychological health and associated factors of men who have sex with men in China: A cross-sectional survey. PLoS ONE. 2018 May;13(5):14.
486. Liu X, Jiang D, Chen X, et al. Mental Health Status and Associated Contributing Factors among Gay Men in China. International journal of environmental research and public health. 2018 May 24;15(6).
487. Liu Y, Jiang C, Li S, et al. Association of recent gay-related stressful events with depressive symptoms in Chinese men who have sex with men. BMC Psychiatry. 2018;18:7.
488. Liu Y, Vermund SH, Ruan Y, et al. Peer counselling versus standard-of-care on reducing high-risk behaviours among newly diagnosed HIV-positive men who have sex with men in Beijing, China: a randomized intervention study [Comparative Study; Journal Article; Randomized

Controlled Trial; Research Support, N.I.H., Extramural]. Journal of the International AIDS Society. 2018 Feb;21(2).
489. Liu Y, Vermund SH, Ruan Y, et al. HIV testing and sexual risks among migrant men who have sex with men: findings from a large cross-sectional study in Beijing, China. AIDS care. 2018;30(1):86-94.
490. Liu Y, Zhang Y, Ning Z, et al. Intimate partner violence victimization and HIV infection among men who have sex with men in Shanghai, China. Biosci Trends. 2018;12(2):142-148.
491. Lu Y, Zhao Y, Meridith Blevins P, et al. Risk Prediction Score for HIV Infection: Development and Internal Validation with Cross-Sectional Data from Men Who Have Sex with Men in China. AIDS and Behavior. 2018 Jul;22(7):2267-2276.
492. Luo W, Hong H, Wang X, et al. Synthetic drug use and HIV infection among men who have sex with men in China: A sixteen-city, cross-sectional survey. PLoS ONE. 2018 Jul 31;13(7):13.
493. Mao J, Tang W, Liu C, et al. Sex tourism among Chinese men who have sex with men: a cross-sectional observational study. BMC public health. 2018 Mar 2;18(1):306.
494. Mao X, Wang Z, Hu Q, et al. HIV incidence is rapidly increasing with age among young men who have sex with men in China: a multicentre cross-sectional survey. HIV Medicine. 2018;19(8):513-522.
495. Meyers K, Wu Y, Qian H, et al. Interest in Long-Acting Injectable PrEP in a Cohort of Men Who have Sex with Men in China. AIDS & Behavior. 2018 Apr;22(4):1217-1227.
496. Mo PK, Lau JT, Wu X. Relationship between illness representations and mental health among HIV-positive men who have sex with men. AIDS care. 2018 Oct;30(10):1246-1251.
497. Mo PKH, Lau JTF, Lau MMC, et al. Mental health service utilization among men who have sex with men who are at risk of mental health problems in Hong Kong: Prevalence and associated factors. Psychology of Men & Masculinity. 2018 Jul;19(3):392-406.
498. Ning Z, Fu J, Zhuang M, et al. HIV and syphilis epidemic among MSM and non-MSM aged 50 and above in Shanghai, China: A yearly cross-sectional study, 2008-2014. Global Public Health. 2018 Nov;13(11):1625-1633.
499. Ong JJ, Fu H, Pan S, et al. Missed Opportunities for Human Immunodeficiency Virus and Syphilis Testing Among Men Who Have Sex With Men in China: A Cross-Sectional Study. Sex Transm Dis. 2018 Jun;45(6):382-386.
500. Ong JJ, Li H, Dan W, et al. Coercion and HIV Self-Testing in Men Who Have Sex With Men: Implementation Data From a Cross-Sectional Survey in China. J Acquir Immune Defic Syndr. 2018 Feb 1;77(2):e22-e25.
501. Ong JJ, Wu D, Huang W, et al. Pressured HIV testing "in the name of love": a mixed methods analysis of pressured HIV testing among men who have sex with men in China [Journal: Article]. J Int AIDS Soc. 2018 Mar;21(3):e25098.
502. Pan SW, Carpiano RM, Li D, et al. Ethnicity and HIV vulnerabilities among men who have sex with men in China. AIDS care. 2018 Aug;30(8):1025-1030.
503. Pan XH, Li RH, Ma QQ, et al. Sexual risk behaviour, sexual victimisation, substance use and other factors related to depression in men who have sex with men in Wenzhou, China: a cross-sectional study. Bmj Open. 2018 Apr;8(4).
504. Poon CM, Wong NS, Kwan TH, et al. Changes of sexual risk behaviors and sexual connections among HIV-positive men who have sex with men along their HIV care continuum [Article]. PLoS One. 2018;13(12):e0209008.

505. Qu D, Zhong X, Xiao G, et al. Adherence to pre-exposure prophylaxis among men who have sex with men: a prospective cohort study [Journal: Article]. Int J Infect Dis. 2018 Oct;75:52-59.
506. Shao B, Song B, Feng S, et al. The relationship of social support, mental health, and health-related quality of life in human immunodeficiency virus-positive men who have sex with men: From the analysis of canonical correlation and structural equation model: A cross-sectional study. Medicine (Baltimore). 2018 Jul;97(30):e11652.
507. Su X, Zhou AN, Li J, et al. Depression, Loneliness, and Sexual Risk-Taking Among HIV-Negative/Unknown Men Who Have Sex with Men in China. Arch Sex Behav. 2018 Oct;47(7):1959-1968.
508. Tang W, Wei C, Cao B, et al. Crowdsourcing to expand HIV testing among men who have sex with men in China: A closed cohort stepped wedge cluster randomized controlled trial [Journal Article; Randomized Controlled Trial; Research Support, N.I.H., Extramural; Research Support, Non-U.S. Gov't]. PLoS Med. 2018 Aug;15(8):e1002645.
509. Tao L, Liu M, Li S, et al. Condom use in combination with ART can reduce HIV incidence and mortality of PLWHA among MSM: a study from Beijing, China. BMC Infectious Diseases. 2018;18:1-1.
510. Wang C, Tucker JD, Liu C, et al. Condom use social norms and self-efficacy with different kinds of male partners among Chinese men who have sex with men: results from an online survey [Article]. BMC public health. 2018 Oct 16;18(1):1175.
511. Wang C-C, Lin H, Chen M-H, et al. Effects of traditional and cyber homophobic bullying in childhood on depression, anxiety, and physical pain in emerging adulthood and the moderating effects of social support among gay and bisexual men in Taiwan. Neuropsychiatric Disease and Treatment. 2018 May 22;14:9.
512. Wang HY, Wang N, Chu ZX, et al. Intimate Partner Violence Correlates With A Higher HIV Incidence Among MSM: A 12-Month Prospective Cohort Study in Shenyang, China. Sci Rep. 2018 Feb 13;8(1):2879.
513. Wang X, Bourne A, Liu P, et al. Understanding willingness to use oral pre-exposure prophylaxis for HIV prevention among men who have sex with men in China. PLoS One. 2018;13(6):e0199525.
514. Wang X, Wang Z, Jiang X, et al. A cross-sectional study of the relationship between sexual compulsivity and unprotected anal intercourse among men who have sex with men in shanghai, China. BMC Infectious Diseases. 2018;18(1):N.PAG-N.PAG.
515. Wang Y-C, Griffiths J, Grande G. The influence of gender identities on body image and breast health among sexual minority women in Taiwan: Implications for healthcare practices. Sex Roles: A Journal of Research. 2018 Feb;78(3-4):242-254.
516. Wang Z, Lau JTF, Fang Y, et al. Prevalence of actual uptake and willingness to use pre-exposure prophylaxis to prevent HIV acquisition among men who have sex with men in Hong Kong, China. PLoS One. 2018;13(2):e0191671.
517. Wang Z, Lau JTF, Ip M, et al. A Randomized Controlled Trial Evaluating Efficacy of Promoting a Home-Based HIV Self-Testing with Online Counseling on Increasing HIV Testing Among Men Who Have Sex with Men [Journal Article; Randomized Controlled Trial; Research Support, Non-U.S. Gov't]. AIDS and behavior. 2018 Jan;22(1):190-201.
518. Wang Z, Lau JTF, She R, et al. Behavioral intention to take up different types of HIV testing among men who have sex with men who were never-testers in Hong Kong [Journal: Article].

AIDS care. 2018 Jan;30(1):95-102.
519. Wang Z, Zhao X, Zhang Z, et al. Co-occurring psychosocial problems and multiple sexual partners among men who have sex with men in Shanghai, China: A syndemic approach. Journal of sex research. 2018 Sep;55(7):892-901.
520. Watson J, Tang W, Pan S, et al. Out of the Closet, Into the Clinic: Opportunities for Expanding Men Who Have Sex With Men-Competent Services in China. Sexually Transmitted Diseases. 2018;45(8):527-533.
521. Wei C, Yan L, Li J, et al. Which user errors matter during HIV self-testing? A qualitative participant observation study of men who have sex with men (MSM) in China. BMC public health. 2018 Sep 10;18(1):1108.
522. Wei C, Yan L, Lippman SA, et al. Prevalence and Correlates of Point-of-sex Human Immunodeficiency Virus Self-testing Among Human Immunodeficiency Virus-negative Men Who Have Sex With Men in China. Sexually Transmitted Diseases. 2018;45(12):818-822.
523. Wong NS, Mao J, Cheng W, et al. HIV Linkage to Care and Retention in Care Rate Among MSM in Guangzhou, China. AIDS & Behavior. 2018 Mar;22(3):701-710.
524. Wu F, Zhang A, Babbitt A, et al. Overcoming HIV stigma? A qualitative analysis of HIV cure research and stigma among men who have sex with men living with HIV. Archives of Sexual Behavior. 2018 Oct;47(7):2061-2069.
525. Wu ZL, Guan GY, Zhao JH, et al. Dynamic Characteristics and HIV Infection of Men who have Sex with Men from 2011 to 2017 in Yinchuan, Ningxia, China. Curr Hiv Res. 2018;16(5):364-373.
526. Xu W, Zheng L, Song J, et al. Relationship Between Childhood Sexual Abuse and HIV-Related Risks Among Men Who Have Sex with Men: Findings from Mainland China. Archives of Sexual Behavior. 2018 Oct;47(7):1949-1957.
527. Xu W, Zheng Y, Kaufman MR. Predictors of Recent HIV Testing Among Chinese Men Who Have Sex with Men: A Barrier Perspective. AIDS Patient Care & STDs. 2018;32(10):408-417.
528. Xu Y, Zheng Y. The Influence of Power and Intimacy Sexual Motives on Sexual Position Preference Among Men Who Have Sex with Men in China. Arch Sex Behav. 2018 Jan;47(1):245-258.
529. Yang LG, Zhang XH, Zhao PZ, et al. Gonorrhea and chlamydia prevalence in different anatomical sites among men who have sex with men: a cross-sectional study in Guangzhou, China. BMC Infect Dis. 2018 Dec 18;18(1):675.
530. Yang Z, Li J, Dong Z, et al. Correlates of recent HIV infection among men who have sex with men recruited through the internet in Huzhou City, Eastern China. J Int Med Res. 2018 Dec;46(12):5052-5061.
531. Ye ML, Giri M. Prevalence and correlates of HIV infection among men who have sex with men: a multi-provincial cross-sectional study in the southwest of China. Hiv Aids-Research and Palliative Care. 2018;10:167-175.
532. Ye Z, Chen L, Lin D. The relationship between posttraumatic stress disorder symptoms and posttraumatic growth among HIV-infected men who have sex with men in Beijing, China: The mediating roles of coping strategies. Frontiers in Psychology. 2018 Sep 27;9:9.
533. Ye Z, Yu NX, Zhu W, et al. A randomized controlled trial to enhance coping and posttraumatic growth and decrease posttraumatic stress disorder in HIV-Infected men who have sex with men in Beijing, China. AIDS care. 2018 Jun;30(6):793-801.

534. Yu L, Li Y, Liu L, et al. Association of recent gay-related stressful events and emotional distress with suicidal behaviors over 12 months in Chinese men who have sex with men. Asia Pac Psychiatry. 2018 Mar;10(1).
535. Yu YQ, Xu JJ, Hu QH, et al. High-risk behaviour and HIV infection risk among non-local men who have sex with men with less than a single year's residence in urban centres: a multicentre cross-sectional study from China. Sex Transm Infect. 2018 Feb;94(1):51-54.
536. Zhang J, Jun-jie X, Song W, et al. HIV Incidence and Care Linkage among MSM First-Time-Testers in Shenyang, China 2012–2014. AIDS and Behavior. 2018 Mar;22(3):711-721.
537. Zhang W, Wang P. Quality of life among men who have sex with men in China measured using the 36-item Short-Form Health Survey: A cross-sectional study. Medicine (Baltimore). 2018 Jul;97(27):e11310.
538. Zhao P, Liu L, Zhang Y, et al. The interaction between HIV testing social norms and self-efficacy on HIV testing among Chinese men who have sex with men: results from an online cross-sectional study. BMC Infect Dis. 2018 Oct 30;18(1):541.
539. Zhao Y, Zhu X, Pérez AE, et al. MHealth approach to promote Oral HIV self-testing among men who have sex with men in China: a qualitative description. BMC public health. 2018;18(1):N.PAG-N.PAG.
540. Zhou J, Chen J, Goldsamt L, et al. HIV Testing and Associated Factors Among Men Who Have Sex with Men in Changsha, China. JANAC: Journal of the Association of Nurses in AIDS Care. 2018;29(6):932-941.
541. Zhou N, Bauermeister J, Guo W, et al. Condomless Anal Intercourse by Partner Type Among Chinese Men Who Have Sex With Men in Tianjin. AIDS Education & Prevention. 2018 Feb;30(1):63-71.
542. Zhu Y, Liu J, Chen Y, et al. The relation between mental health, homosexual stigma, childhood abuse, community engagement, and unprotected anal intercourse among MSM in China. Sci Rep. 2018 Mar 5;8(1):3984.
543. Cao B, Fu H, Liu C, et al. MSM Behavior Disclosure Networks and HIV Testing: An Egocentric Network Analysis Among MSM in China. AIDS & Behavior. 2019 May;23(5):1368-1374.
544. Cao B, Saha PT, Leuba SI, et al. Recalling, sharing and participating in a social media intervention promoting HIV testing: A longitudinal analysis of HIV testing among MSM in China. AIDS and Behavior. 2019 May 15;23(5):1240-1249.
545. Chan RCH, Mak WWS. Cognitive, Regulatory, and Interpersonal Mechanisms of HIV Stigma on the Mental and Social Health of Men Who Have Sex With Men Living With HIV [Article]. American Journal of Men's Health. 2019 Oct;13(5):1557988319873778.
546. Chen L, Yang J, Ma Q, et al. Prevalence of Active Syphilis Infection and Risk Factors among HIV-Positive MSM in Zhejiang, China in 2015: A Cross-Sectional Study. International journal of environmental research and public health. 2019 Apr 28;16(9).
547. Chen WT, Shiu CS, Yang JP, et al. Tobacco, Alcohol, Drug Use, and Intimate Partner Violence Among MSM Living With HIV. Janac-Journal of the Association of Nurses in Aids Care. 2019 Nov-Dec;30(6):610-618.
548. Chen X, Mo PKH, Li J, et al. Factors Associated with Drug Use Among HIV-Infected Men Who Have Sex with Men in China. AIDS and Behavior. 2019 Sep;24(6):1-9.
549. Cheng W, Xu H, Tang W, et al. Online HIV prevention intervention on condomless sex among

men who have sex with men: a web-based randomized controlled trial. BMC Infect Dis. 2019 Jul 19;19(1):644.

550. Cheng WB, Jin W, Gu YZ, et al. HIV Partner Notification Across Different Sexual Partner Types Among Men Who Have Sex with Men in Guangzhou, China. Aids Patient Care St. 2019 Jul;33(7):295-298.
551. Chu J-H, Huang J-H. Psychosociobehavioral characteristics associated with high condomless anal intercourse intention: A comparison of receptive, versatile, and insertive msm in taiwan. AIDS care. 2019 Aug 20;32(6):770-778.
552. Dai Y, Musumari PM, Chen H, et al. Recreational Drug Use, Polydrug Use and Sexual Behaviors Among Men Who Have Sex With Men in Southwestern China: A Cross-Sectional Study. Behavioral Medicine. 2019 Oct-Dec;45(4):314-322.
553. Day W, Zhang W, Yee Wan K, et al. Gender Differences in Identity Concerns Among Sexual Minority Young Adults in China: Socioeconomic Status, Familial, and Cultural Factors. Sexuality & Culture. 2019 Dec;23(4):1167-1187.
554. Deng-Min C, Newman PA, Weaver J. HIV Vaccine Preparedness among Men Who Have Sex with Men in Taiwan: Sociocultural and Behavioral Factors. Journal of the International Association of Providers of AIDS Care. 2019 Jan;18.
555. Ding C, Wang T, Chen X, et al. Association of Adverse Childhood Experience and Attention Deficit Hyperactivity Disorder with depressive symptoms among men who have sex with men in China: moderated mediation effect of resilience. BMC public health. 2019 Dec 19;19(1):1706.
556. Dong MJ, Peng B, Liu ZF, et al. The prevalence of HIV among MSM in China: a large-scale systematic analysis. BMC Infect Dis. 2019 Nov 27;19(1):1000.
557. Fan S, Yang Z, Hou F, et al. HIV and syphilis and sexual risk behaviours among men who have sex with men attending university in China: a systematic review and meta-analysis [Review]. Sex Health. 2019 Nov;16(6):554-565.
558. Han J, Bouey JZ, Wang L, et al. PrEP uptake preferences among men who have sex with men in China: results from a National Internet Survey. J Int AIDS Soc. 2019 Feb;22(2):e25242.
559. Han X, Han WT, Qu JB, et al. What happens online stays online? - Social media dependency, online support behavior and offline effects for LGBT. Computers in Human Behavior. 2019 Apr;93:91-98.
560. Hu HF, Chang YP, Lin C, et al. Quality of life of gay and bisexual men during emerging adulthood in Taiwan: Roles of traditional and cyber harassment victimization. PLoS One. 2019;14(2):e0213015.
561. Hu QH, Meyers K, Xu JJ, et al. Efficacy and cost-effectiveness of early antiretroviral therapy and partners' pre-exposure prophylaxis among men who have sex with men in Shenyang, China: a prospective cohort and costing study. BMC Infect Dis. 2019 Jul 25;19(1):663.
562. Hu Y, Zhong XN, Peng B, et al. Comparison of depression and anxiety between HIV-negative men who have sex with men and women (MSMW) and men who have sex with men only (MSMO): a cross-sectional study in Western China [Journal Article; Randomized Controlled Trial; Research Support, Non-U.S. Gov't]. BMJ open. 2019 Jan 3;9(1):e023498.
563. Huang P, Wu H-J, Strong C, et al. Unspeakable PrEP: A qualitative study of sexual communication, problematic integration, and uncertainty management among men who have sex with men in Taiwan. Journal of Applied Communication Research. 2019 Dec;47(6):611-627.

564. Huang W, Wu D, Ong JJ, et al. Prepared for PrEP: preferences for HIV pre-exposure prophylaxis among Chinese men who have sex with men in an online national survey. BMC Infect Dis. 2019 Dec 16;19(1):1057.
565. Huang W, Wu D, Pan SW, et al. Driving force of condomless sex after online intervention among Chinese men who have sex with men. BMC public health. 2019 Jul 22;19(1):978.
566. Jiang D, Hou Y, Chen X, et al. Interpersonal Sensitivity and Loneliness among Chinese Gay Men: A Cross-Sectional Survey. International journal of environmental research and public health. 2019 Jun 8;16(11).
567. Jiang H, Chen X, Li J, et al. Predictors of condom use behavior among men who have sex with men in China using a modified information-motivation-behavioral skills (IMB) model. BMC public health. 2019 Mar 4;19(1):261.
568. Jiang T, Zhou X, Wang H, et al. Psychosocial Factors Associated with Quality of Life in Young Men Who Have Sex with Men Living with HIV/AIDS in Zhejiang, China. International journal of environmental research and public health. 2019 Jul 25;16(15).
569. Jiang Z, Wang Y, Hu XW, et al. Open Workplace Climate and LGB Employees' Psychological Experiences: The Roles of Self-Concealment and Self-Acceptance. Journal of Employment Counseling. 2019 Mar;56(1):2-19.
570. Jin X, Xu J, Smith MK, et al. An Internet-Based Self-Testing Model (Easy Test): Cross-Sectional Survey Targeting Men Who Have Sex With Men Who Never Tested for HIV in 14 Provinces of China. Journal of Medical Internet Research. 2019 May 15;21(5):N.PAG-N.PAG.
571. Knudson A, Shaw S, Amico KR, et al. Exploration of Implementation Patterns and Content from a Text-Based Outreach Intervention Clinical Trial for Newly Diagnosed, HIV-Positive MSM in Beijing, China. AIDS & Behavior. 2019 May;23(5):1158-1165.
572. Kong TSK. Transnational queer sociological analysis of sexual identity and civic-political activism in Hong Kong, Taiwan and mainland China. Br J Sociol. 2019 Dec;70(5):1904-1925.
573. Kong TSK. The pursuit of masculinity by young gay men in neoliberal Hong Kong and Shanghai. Journal of Youth Studies. 2019:1-18.
574. Lee JI, Yen CF, Hsiao RC, et al. Relationships of homophobic bullying during childhood and adolescence with problematic internet and smartphone use in early adulthood among sexual minority men in Taiwan. Archives of Clinical Psychiatry. 2019 Jul-Aug;46(4):97-102.
575. Lee SS, Kwan TH, Wong NS, et al. Piloting a partially self-financed mode of human immunodeficiency virus pre-exposure prophylaxis delivery for men who have sex with men in hong kong [Article]. Hong Kong Medical Journal. 2019 Oct;25(5):382-391.
576. Li DJ, Chen SL, Yen CF. Multi-Dimensional Factors Associated with Illegal Substance Use Among Gay and Bisexual Men in Taiwan. International journal of environmental research and public health. 2019 Nov 14;16(22).
577. Li KT, Tang W, Wu D, et al. Pay-it-forward strategy to enhance uptake of dual gonorrhea and chlamydia testing among men who have sex with men in China: a pragmatic, quasi-experimental study [Journal: Article]. The Lancet Infectious Diseases. 2019;19(1):76-82.
578. Li Q, Li X, Luo Y, et al. HIV incidence and cohort retention among men who have sex with men in Hangzhou, China: A prospective cohort study. Medicine (Baltimore). 2019 Oct;98(40):e17419.
579. Li XW, Cao XF, Li Z, et al. Human Papillomavirus awareness and vaccine acceptability among men who have sex with men from mainland China. Scientific Reports. 2019 Jun;9.

580. Liu FY, Qin YL, Meng SY, et al. HIV self-testing among men who have sex with men in China: a qualitative implementation research study. Journal of Virus Eradication. 2019 Oct;5(4):220-224.
581. Liu PL, Yeo TED. Breast health, risk factors, and cancer screening among lesbian, bisexual, and queer/questioning women in China [Article]. Health Care for Women International. 2019 Feb 7:1-15.
582. Lo IPY, Kim YK, Small E, et al. The gendered self of Chinese lesbians: Self-esteem as a mediator between gender roles and depression. Archives of Sexual Behavior. 2019 Jul 15;48(5):1543-1554.
583. Lu WH, Chang YP, Lin CH, et al. Negative Facebook experiences among Taiwanese gay and bisexual men in emerging adulthood: associations with traditional harassment victimization and quality of life. Neuropsychiatric Disease and Treatment. 2019;15:1163-1170.
584. Luo Q, Wu Z, Chen Z, et al. App use frequency and condomless anal intercourse among men who have sex with men in Beijing, China: a cross-sectional study. International Journal of STD & AIDS. 2019 Oct;30(11):1146-1155.
585. Ning X, Chau-Kiu C, Guo S. Using Grounded Theory to Understand a Cutting-Edge Issue: Effects of Integrative Tactics on Chinese Gay Men’s and Lesbians’ Social Well-Being. International Journal of Qualitative Methods. 2019 Jan;18.
586. Niu L, Wang Z, Fang Y, et al. Behavior intention to use routine opt-out HIV testing in primary care settings among men who have sex with men in China. AIDS care. 2019 Dec;31(12):1565-1573.
587. Ong JJ, Li CC, Fu H, et al. Risk attitudes, risky sexual behaviours and willingness to test negative for syphilis using lottery-based financial incentives among Chinese men who have sex with men [Article]. Sexually Transmitted Infections. 2019 Oct 25;96(5):355-357.
588. Ong JJ, Liao M, Lee A, et al. Bridging the HIV-syphilis testing gap: dual testing among men who have sex with men living in China. Sex Transm Infect. 2019 Jun;95(4):251-253.
589. Pan SW, Durvasula M, Ong JJ, et al. No Place Like Home? Disentangling Preferences for HIV Testing Locations and Services Among Men Who Have Sex with Men in China. AIDS & Behavior. 2019;23(4):847-859.
590. Peng L, Cao W, Gu J, et al. Willingness to Use and Adhere to HIV Pre-Exposure Prophylaxis (PrEP) among Men Who Have Sex with Men (MSM) in China. International journal of environmental research and public health. 2019 Jul 23;16(14).
591. Qu D, Zhong XN, Lai MQ, et al. Influencing Factors of Pre-Exposure Prophylaxis Self-Efficacy Among Men Who Have Sex With Men. American Journal of Mens Health. 2019 Apr;13(2).
592. Ren Z, Howe CQ, Zhang W. Maintaining "mianzi" and "lizi": Understanding the reasons for formality marriages between gay men and lesbians in China. Transcultural Psychiatry. 2019 Feb;56(1):213-232.
593. Ritchwood TD, He JY, Smith MK, et al. "Getting to Zero" Among Men Who Have Sex with Men in China: a Review of the HIV Care Continuum. Current Hiv/Aids Reports. 2019 Dec;16(6):431-438.
594. Strong C, Yu YF, Zou H, et al. Sexual network and detection of anogenital human papillomavirus in a community cohort of men who have sex with men in Taiwan. PLoS One. 2019;14(5):e0216784.

595. Tang W, Huang W, Lu H, et al. What happens after HIV self-testing? Results from a longitudinal cohort of Chinese men who have sex with men [Journal Article; Randomized Controlled Trial]. BMC infectious diseases. 2019;19(1):807.
596. Tian T, Wang D, Papamichael C, et al. HPV vaccination acceptability among men who have sex with men in Urumqi, China. Hum Vaccin Immunother. 2019;15(4):1005-1012.
597. Wang C-C, Chang Y-P, Yang Y-H, et al. Relationships between traditional and cyber harassment and self-identity confusion among Taiwanese gay and bisexual men in emerging adulthood. Comprehensive Psychiatry. 2019 Apr;90:14-20.
598. Wang CC, Hsiao RC, Yen CF. Victimization of Traditional and Cyber Bullying During Childhood and Their Correlates Among Adult Gay and Bisexual Men in Taiwan: A Retrospective Study. International journal of environmental research and public health. 2019 Nov 21;16(23).
599. Wang L, Podson D, Chen Z, et al. Using Social Media To Increase HIV Testing Among Men Who Have Sex with Men - Beijing, China, 2013-2017. MMWR Morb Mortal Wkly Rep. 2019 May 31;68(21):478-482.
600. Wang LR, Santella AJ, Wei XL, et al. Prevalence and protective factors of HIV and syphilis infection among men who have sex with men in Northwest China. Journal of Medical Virology. 2019 Nov 7;92(8):1141-1147.
601. Wang N, Wang S, Qian H-Z, et al. Negative associations between general self-efficacy and anxiety/depression among newly HIV-diagnosed men who have sex with men in Beijing, China. AIDS care. 2019 May;31(5):629-635.
602. Wang PW, Ko NY, Hsiao RC, et al. Suicidality Among Gay and Bisexual Men in Taiwan: Its Relationships with Sexuality and Gender Role Characteristics, Homophobic Bullying Victimization, and Social Support. Suicide Life Threat Behav. 2019 Apr;49(2):466-477.
603. Wang Y, Jia M, Yuan D, et al. Assessing consistent condom use among migrant men who have sex with men in Shanghai, China: validation of an information-motivation-behavioural skills model. BMC Infect Dis. 2019 May 23;19(1):462.
604. Wang Y-Y, Dong M, Zhang Q, et al. Suicidality and clinical correlates in Chinese men who have sex with men (MSM) with HIV infection. Psychology, health & medicine. 2019 Feb;24(2):137-143.
605. Wang Z, Mo PKH, Fang Y, et al. Factors predicting first-time hepatitis C virus testing uptake among men who have sex with men in China: An observational prospective cohort study [Article]. Sexually Transmitted Infections. 2019 Jun;96(4):258-264.
606. Wei C, Liu W. Coming out in Mainland China: A national survey of LGBTQ students. Journal of LGBT Youth. 2019;16(2):192-219.
607. Wei D, Hou F, Hao C, et al. Prevalence of Intimate Partner Violence and Associated Factors Among Men Who Have Sex with Men in China. J Interpers Violence. 2019 Dec 2:886260519889935.
608. Wei L, Chen L, Zhang H, et al. Use of gay app and the associated HIV/syphilis risk among non-commercial men who have sex with men in Shenzhen, China: a serial cross-sectional study. Sex Transm Infect. 2019 Nov;95(7):496-504.
609. Wei L, Chen L, Zhang HB, et al. Relationship between gay app use and HIV testing among men who have sex with men in Shenzhen, China: a serial cross-sectional study. Bmj Open. 2019 Aug;9(8):e028933.

610. Wei X, Zhang Y, Santella AJ, et al. Effect of early highly active antiretroviral therapy on viral suppression among newly diagnosed men who have sex with men living with human immunodeficiency virus in Xi'an, China. J Med Virol. 2019 Jul;91(7):1263-1271.
611. Wen GJ, Zheng LJ. The Influence of Internalized Homophobia on Health-Related Quality of Life and Life Satisfaction Among Gay and Bisexual Men in China. American Journal of Mens Health. 2019 Jul;13(4):1557988319864775.
612. Weng RX, Hong FC, Yu WY, et al. Compare HIV/syphilis infections between age groups and explore associated factors of HIV/syphilis co-infections among men who have sex with men in Shenzhen, China, from 2009 to 2017. PLoS One. 2019;14(10):e0223377.
613. Wu AMS, Lau JTF, Wang Z, et al. Prevalence and Factors of Concurrent Multiple Male Sex Partnership Among Men Who Have Sex With Men With Regular Sex Partners in Beijing, China. J Sex Marital Ther. 2019;45(3):247-258.
614. Wu D, Huang WT, Zhao PP, et al. A Crowdsourced Physician Finder Prototype Platform for Men Who Have Sex with Men in China: Qualitative Study of Acceptability and Feasibility. Jmir Public Health and Surveillance. 2019 Oct-Dec;5(4):154-164.
615. Wu D, Li KT, Tang W, et al. Low Chlamydia and Gonorrhea Testing Rates among Men Who Have Sex with Men in Guangdong and Shandong Provinces, China [Article]. Sexually Transmitted Diseases. 2019 Apr;46(4):260-265.
616. Wu D, Tao H, Dai J, et al. Study on Pre-Exposure Prophylaxis Regimens among Men Who Have Sex with Men: A Prospective Cohort Study. International journal of environmental research and public health. 2019 Dec 9;16(24).
617. Wu H, Xiu C, Fu X, et al. Syphilis associated with recreational drug use, depression and high-risk sexual behaviour in men who have sex with men: a case-control study in China. Sex Transm Infect. 2019 Jun;95(4):267-272.
618. Wu SW, Ward J. Looking for "interesting people": Chinese gay men's exploration of relationship development on dating apps. Mobile Media & Communication. 2019.
619. Wu T, Qu S, Fang Y, et al. Behavioral intention to perform risk compensation behaviors after receiving HPV vaccination among men who have sex with men in China [Journal Article; Randomized Controlled Trial; Research Support, Non-U.S. Gov't]. Human vaccines & immunotherapeutics. 2019;15(7-8):1737-1744.
620. Wu Y, Xie L, Meng S, et al. Mapping potential pre-exposure prophylaxis users onto a motivational cascade: Identifying targets to prepare for implementation in china. LGBT Health. 2019 Jun 06;6(5):250-260.
621. Xie L, Wu Y, Meng S, et al. Risk behavior not associated with self-perception of PrEP candidacy: Implications for designing PrEP services. AIDS and Behavior. 2019 Oct;23(10):2784-2794.
622. Xu W, Zheng Y, Wiginton JM, et al. Alcohol use and binge drinking among men who have sex with men in China: Prevalence and correlates. Drug & Alcohol Dependence. 2019 Sep 1;202:61-68.
623. Xuan N, Kiu CC, Guo SJ. Negotiations Between Chinese Gay Men and Lesbians and Their Parents About Marriage. International Journal of Qualitative Methods. 2019 Jul;18.
624. Yan H, Cao W, Mo P, et al. Prevalence and associated factors of HIV serostatus disclosure to regular female sex partners among HIV-positive men who have sex with both men and women in China. AIDS care. 2019 Aug;31(8):1026-1034.

625. Yan H, Li X, Li J, et al. Association between perceived HIV stigma, social support, resilience, self-esteem, and depressive symptoms among HIV-positive men who have sex with men (MSM) in Nanjing, China. AIDS care. 2019 Sep;31(9):1069-1076.
626. Yang J, Xu H, Li S, et al. The characteristics of mixing patterns of sexual dyads and factors correlated with condomless anal intercourse among men who have sex with men in Guangzhou, China. BMC public health. 2019 Jun 10;19(1):722.
627. Yu M, Xu J, Jiang G, et al. Correlates of HIV-infection among men who have sex with men: results from a community-based, cross-sectional study in Tianjin, China. AIDS care. 2019 Dec;31(12):1574-1579.
628. Zhang A, Reynolds NR, Farley JE, et al. Preferences for an HIV prevention mobile phone app: a qualitative study among men who have sex with men in China. BMC public health. 2019 Mar 12;19(1):297.
629. Zhang DC, Wu ZY, Scott SR. Factors associated with unprotected anal intercourse among male students who have sex with men in three Northern regions of China. Chin Med J (Engl). 2019 Jul 20;132(14):1639-1644.
630. Zhang P, Gao J, Wang Y, et al. Effect of chronic disease self-management program on the quality of life of HIV-infected men who have sex with men: An empirical study in Shanghai, China. Int J Health Plann Manage. 2019 Jul;34(3):1055-1064.
631. Zhang S, Wang S, Wang Z, et al. The association between involuntary subordination and common mental disorders among men who have sex with men (MSM) in Shanghai, China. BMC Psychiatry. 2019 Nov 27;19:9.
632. Zhang Y, Wu G, Lu R, et al. What has changed HIV and syphilis infection among men who have sex with men (MSM) in Southwest China: a comparison of prevalence and behavioural characteristics (2013-2017). BMC public health. 2019 Oct 21;19(1):1314.
633. Zhang YC, Worth H, Jun J, et al. 'I loved him all my life': love, duty and homosexuality in post-liberation China. Culture Health & Sexuality. 2019 May;21(5):591-604.
634. Zhao N, Li KT, Gao YY, et al. Mycoplasma Genitalium and Mycoplasma Hominis are prevalent and correlated with HIV risk in MSM: a cross-sectional study in Shenyang, China. BMC Infect Dis. 2019 Jun 4;19(1):494.
635. Zheng L. Relationships between disgust sensitivity and trait preferences in gay men in China. Personality and Individual Differences. 2019 Aug 01;146:58-61.
636. Zheng Z-W, Qiu J-L, Gu J, et al. Preexposure prophylaxis comprehension and the certainty of willingness to use preexposure prophylaxis among men who have sex with men in China. International Journal of STD & AIDS. 2019 Jan;30(1):4-11.
637. Zhu X, Zhang W, Operario D, et al. Effects of a Mobile Health Intervention to Promote HIV Self-testing with MSM in China: A Randomized Controlled Trial. AIDS and Behavior. 2019 Nov;23(11):3129-3139.
638. Zhu Z, Yan H, Wu S, et al. Trends in HIV prevalence and risk behaviours among men who have sex with men from 2013 to 2017 in Nanjing, China: a consecutive cross-sectional survey. BMJ Open. 2019 Jan 30;9(1):e021955.
639. An X, Sun QL, Fang F, et al. The Prevalence of Depression Associated with the Infection Status and Sexual Behaviors among Men Who Have Sex with Men in Shenzhen, China: A Cross-Sectional Study. International journal of environmental research and public health. 2020 Jan;17(1).

640. Cao W, Sun S, Peng L, et al. Low willingness to pay for pre-exposure prophylaxis (PrEP) among men who have sex with men (MSM) in China [Article]. BMC public health. 2020 Mar 16;20(1):337.
641. Chan RCH, Operario D, Mak WWS. Bisexual individuals are at greater risk of poor mental health than lesbians and gay men: The mediating role of sexual identity stress at multiple levels. Journal of Affective Disorders. 2020 Jan 01;260:292-301.
642. Cheng W, Egan JE, Liu Q, et al. Psychosocial Correlates of HIV Testing Frequency Among Men Who Have Sex with Men in Guangzhou, China. AIDS & Behavior. 2020 Feb;24(2):363-372.
643. Ding C, Chen X, Wang W, et al. Sexual Minority Stigma, Sexual Orientation Concealment, Social Support and Depressive Symptoms Among Men Who have Sex with Men in China: A Moderated Mediation Modeling Analysis. AIDS and Behavior. 2020 Jan;24(1):8-17.
644. Fu R, Kutner BA, Wu Y, et al. Do gay and bisexual men who conceal their same-sex behavior prefer different kinds of health services? Findings across four cities to inform client-centered HIV prevention in China. BMC public health. 2020 Jan 6;20(1):4.
645. Hou J, Wu Y, Xie L, et al. Post-exposure prophylaxis: An underutilized biomedical hiv prevention method among gay, bisexual and other men who have sex with men in china. AIDS care. 2020 Mar 20:1-8.
646. Hu J, Tan L, Huang G, et al. Disparity in depressive symptoms between heterosexual and sexual minority men in China: The role of social support. PLoS One. 2020;15(1):e0226178.
647. Hu L, Luo YT, Zhong XN, et al. Condom Use and Related Factors among Rural and Urban Men Who Have Sex With Men in Western China: Based on Information-Motivation-Behavioral Skills Model. American Journal of Mens Health. 2020 Jan;14(1):1557988319899799.
648. Huang Y-T, Chan RCH, Cui L. Filial piety, internalized homonegativity, and depressive symptoms among Taiwanese gay and bisexual men: A mediation analysis. American Journal of Orthopsychiatry. 2020;90(3):340-349.
649. Huang YT, Luo H, Ko NY, et al. Perceived Attitudes Toward Lesbian, Gay, and Bisexual (LGB) Issues and Mental Health Among Taiwanese LGB Adults: The Mediating Role of Self-Acceptance. Archives of Sexual Behavior. 2020 Jul;49(5):1671-1682.
650. Jiang H, Li J, Tan Z, et al. Syndemic Factors and HIV Risk Among Men Who Have Sex with Men in Guangzhou, China: Evidence from Synergy and Moderated Analyses [Article]. Archives of Sexual Behavior. 2020 Jan;49(1):311-320.
651. Jiang HB, Hong H, Dong HJ, et al. HIV Testing and Risks of Sexual Behavior among HIV-Negative Men Who Have Sex with Men in Ningbo, China. International journal of environmental research and public health. 2020 Feb;17(4).
652. Li DJ, Chen SL, Chang YP, et al. Factors Affecting Painkillers, Sedatives/Hypnotics, Nicotine, and Unhealthy Alcohol Use Among Gay and Bisexual Men in Taiwan. International journal of environmental research and public health. 2020 Feb;17(3).
653. Li H, Tucker JD, Ma W, et al. Growth Trajectories of Peer Norms, Self-efficacy and Condom Use Behavior Among Sexually Active Chinese Men Who Have Sex with Men: Latent Class Analysis and Growth Mixture Modeling. AIDS & Behavior. 2020 Mar;24(3):854-865.
654. Li HC, Tucker JD, Ma W, et al. Mediation Analysis of Peer Norms, Self-Efficacy, and Condom Use Among Chinese Men Who Have Sex with Men: A Parallel Process Latent Growth Curve Model. Archives of Sexual Behavior. 2020 Jan;49(1):287-297.

655. Li J, Yue XL, Wang YJ, et al. First Responses to the Symptoms of Sexually Transmitted Infections and Treatment-Seeking Behavior Among Men Who Have Sex With Men in 13 Cities in China. Sexually Transmitted Diseases. 2020 Jan;47(1):28-33.
656. Lin H, Xiaohong P, Jiezhe Y, et al. HIV risk behavior and HIV testing among rural and urban men who have sex with men in Zhejiang Province, China: A respondent-driven sampling study. PLoS ONE. 2020;15(4):e0231026.
657. Liu C. "Red is not the only color of a rainbow": The making and resistance of the "MSM" subject among gay men in China. Social Science & Medicine. 2020 May;252:N.PAG-N.PAG.
658. Liu C, Zhang Y, Pan SW, et al. Anticipated HIV stigma among HIV negative men who have sex with men in China: a cross-sectional study [Journal Article; Randomized Controlled Trial]. BMC infectious diseases. 2020 Jan 15;20(1):44.
659. Liu F, Chui H, Chung MC. The moderating effect of filial piety on the relationship between perceived public stigma and internalized homophobia: A national survey of the chinese lgb population. Sexuality Research & Social Policy: A Journal of the NSRC. 2020 Apr 03.
660. Liu JX, Zhong XN, Lu Z, et al. Anxiety and Depression Associated with Anal Sexual Practices among HIV-Negative Men Who Have Sex with Men in Western China. International journal of environmental research and public health. 2020 Jan;17(2).
661. Liu Y, Wu G, Lu R, et al. Facilitators and barriers associated with uptake of HIV self-testing among men who have sex with men in Chongqing, China: A cross-sectional survey [Article]. International journal of environmental research and public health. 2020 Mar 3;17(5).
662. Mo PKH, Chen X, Lam EHK, et al. The Moderating Role of Social Support on the Relationship Between Anxiety, Stigma, and Intention to Use Illicit Drugs Among HIV-Positive Men Who Have Sex with Men. AIDS & Behavior. 2020 Jan;24(1):55-64.
663. Pan SW, Carpiano RM, Smith MK, et al. Supernatural explanatory models of health and illness and healthcare use in China among men who have sex with men. Global Public Health. 2020 Jan;15(1):83-96.
664. Pan SW, Smith MK, Carpiano RM, et al. Supernatural Explanatory Models of Health and Illness and HIV Antiretroviral Therapy Use Among Young Men Who Have Sex with Men in China. International journal of behavioral medicine. 2020 May 15.
665. Peng L, She R, Gu J, et al. The mediating role of self-stigma and self-efficacy between intimate partner violence (IPV) victimization and depression among men who have sex with men in China. BMC public health. 2020 Jan 3;20(1):2.
666. Qiu H, Huang S. Mobile Dating, Relational Communication, and Motivations for AIDS Risk Reduction among Chinese MSM College Students. Health Communication. 2020 Mar;35(3):289-296.
667. Shen KR, Yang NS, Huang WT, et al. A crowdsourced intervention to decrease hepatitis B stigma in men who have sex with men in China: A cohort study. Journal of Viral Hepatitis. 2020 Feb;27(2):135-142.
668. Shi X, Xu W, Zheng Y. Heterosexual marital intention: Effects of internalized homophobia, homosexual identity, perceived family support, and disclosure among Chinese gay and bisexual men. Journal of Homosexuality. 2020;67(4):452-467.
669. Smith MK, Wei C, Liu C, et al. Gender Identity and Sexual Orientation in Chinese Men Who Have Sex with Men: A Latent Class Analysis [Article]. Archives of Sexual Behavior. 2020 Feb;49(2):721-731.

670. Strong C, Zou H, Ko NY, et al. Prevalence and risk factors of anogenital human papillomavirus infection in a community sample of men who have sex with men in Taiwan: baseline findings from a cohort study. Sex Transm Infect. 2020 Feb;96(1):62-66.
671. Suen Y-t, Chan RCH. A nationwide cross-sectional study of 15,611 lesbian, gay and bisexual people in China: disclosure of sexual orientation and experiences of negative treatment in health care. International Journal for Equity in Health. 2020 Apr 1;19(1):1-12.
672. Sun S, Budge S, Shen W, et al. Minority stress and health: A grounded theory exploration among men who have sex with men in China and implications for health research and interventions. Social Science & Medicine. 2020 May;252:N.PAG-N.PAG.
673. Sun S, Hoyt WT, Pachankis JE. Sexual risk behaviors in the internet age: the case of Chinese men who have sex with men. AIDS care. 2020 Mar;32(3):302-309.
674. Sun S, Pachankis JE, Li X, et al. Addressing Minority Stress and Mental Health among Men Who Have Sex with Men (MSM) in China [Review]. Current HIV/AIDS Reports. 2020 Feb;17(1):35-62.
675. Sun S, Whiteley L, Brown LK. HIV Testing Among Chinese Men Who Have Sex with Men: The Roles of HIV Knowledge, Online Social Life, and Sexual Identity Concerns. AIDS & Behavior. 2020 Feb;24(2):437-449.
676. Sun XM, Wang CY, Zhao K, et al. HIV Seroconversion and Types of Relationships Among Men Who Have Sex With Men: A Cohort Study in China. Jaids-Journal of Acquired Immune Deficiency Syndromes. 2020 Apr;83(4):365-372.
677. Wang N, Huang B, Ruan Y, et al. Association between stigma towards HIV and MSM and intimate partner violence among newly HIV-diagnosed Chinese men who have sex with men [Article]. BMC public health. 2020 Feb 10;20(1):204.
678. Wang X, Tang Z, Wu Z, et al. Promoting oral HIV self-testing via the internet among men who have sex with men in China: a feasibility assessment. HIV Medicine. 2020 May;21(5):322-333.
679. Wang YC, Chang SR, Miao NF. The Role of Butch versus Femme Identity in Body Image and Breast Health Among Lesbians in Taiwan: Results of an Online Survey. Journal of Nursing Scholarship. 2020 Mar;52(2):174-182.
680. Wang Z, Mo PKH, Ip M, et al. Uptake and willingness to use PrEP among Chinese gay, bisexual and other men who have sex with men with experience of sexualized drug use in the past year [Article]. BMC infectious diseases. 2020 Apr 22;20(1):299.
681. Wei D, Cao W, Hou F, et al. Multilevel factors associated with perpetration of five types of intimate partner violence among men who have sex with men in china: An ecological model-informed study. AIDS care. 2020 Feb 26:1-12.
682. Wen G, Zheng L. Relationship Status and Marital Intention Among Chinese Gay Men and Lesbians: The Influences of Minority Stress and Culture-Specific Stress [Article]. Archives of Sexual Behavior. 2020 Feb;49(2):681-692.
683. Wong NS, Kwan TH, Lee KCK, et al. Delineation of chemsex patterns of men who have sex with men in association with their sexual networks and linkage to HIV prevention [Article]. International Journal of Drug Policy. 2020 Jan;75:102591.
684. Wu D, Yang E, Huang W, et al. Homoprejudiced violence among Chinese men who have sex with men: A cross-sectional analysis in Guangzhou, China [Article]. BMC public health. 2020 Mar 27;20(1):400.
685. Wu W, Yan X, Zhang X, et al. Potential HIV transmission risk among spouses: Marriage

intention and expected extramarital male-to-male sex among single men who have sex with men in Hunan, China [Article]. Sexually Transmitted Infections. 2020 Mar;96(2):151-156.
686. Xiao W, Yan L, Chen L, et al. Sexual network distribution of HIV self-testing kits: Findings from the process evaluation of an intervention for men who have sex with men in China [Article]. PLoS ONE. 2020;15(4):e0232094.
687. Xu W, Tang W, Zhang J, et al. Cigarette Smoking and Its Associations with Substance Use and HIV-Related Sexual Risks among Chinese Men Who Have Sex with Men [Article]. International journal of environmental research and public health. 2020 Mar 5;17(5).
688. Yang F, Zhang TP, Tang W, et al. Pay-it-forward gonorrhoea and chlamydia testing among men who have sex with men in China: a randomised controlled trial. The Lancet Infectious Diseases. 2020 2020/04/28/.
689. Yang X, Li X, Qiao S, et al. Intersectional stigma and psychosocial well-being among MSM living with HIV in Guangxi, China. AIDS care. 2020 May;32(sup2):5-13.
690. Yang X, Wang Z, Harrison S, et al. Coverage and adherence of antiretroviral therapy among Chinese HIV-positive men who have sex with men with high CD4 counts in the era of 'Treat all' [Article]. Tropical Medicine and International Health. 2020 Mar;25(3):308-318.
691. Yang Z, Li S, Zhang R, et al. Current heterosexual marriage is associated with significantly decreased levels of anxiety symptoms among Chinese men who have sex with men. BMC Psychiatry. 2020 Apr 06;20:7.
692. Zhang J, Xu JJ, Leuba SI, et al. Increasing Condomless Anal Intercourse and HIV Prevalence Among Men Who Have Sex with Men Who Have Never Been Tested for HIV Before: A Serial Cross-Sectional Study in Shenyang, China from 2012 to 2016. Aids and Behavior. 2020 Apr 15.
693. Zheng L, Hart TA, Noor SW, et al. Stressors based on sexual orientation and mental health among lesbian, gay, and bisexual individuals in china: Minority stress and perceived pressure to get married. Archives of Sexual Behavior. 2020 Apr 13;49(5):1769-1782.
694. Zhou YG, Lin YF, Meng XJ, et al. Anal human papillomavirus among men who have sex with men in three metropolitan cities in southern China: implications for HPV vaccination. Vaccine. 2020 Mar;38(13):2849-2858.

## *Theses*

1. Wong CY. Victimization experiences of Chinese gay men and lesbians in Hong Kong: A longitudinal study and an evaluation of a psychoeducational program on sexual identity management strategies. Hong Kong: The Chinese University of Hong Kong 2006.
2. Hao C. HIV Related Sexual Risk Behaviors among Men Who Have Sex with Men in China: A Cohort and Randomized Controlled Study [Ph.D.]. Hong Kong: The Chinese University of Hong Kong (Hong Kong); 2011.
3. Li H. Secondary HIV Transmissions via Newly Diagnosed HIV Positive Men Who Have Sex with Men (MSM) in Shenzhen, China—a Qualitative Study [Ph.D.]. Ann Arbor: The Chinese University of Hong Kong (Hong Kong); 2012.
4. Zhao J. HIV transmission pattern among men who have sex with men in Shenzhen: The Chinese University of Hong Kong; 2012.
5. Zhang L. HIV Risk among Men Who Have Sex with Men in a Large Chinese City [Ph.D.]. Ann Arbor: Vanderbilt University; 2013.
6. Wu X. Mental health, risk behaviours and illness perception among newly diagnosed HIV

positive men who have sex with men in China: The Chinese University of Hong Kong; 2014.
7. Li C. A Cross-sectional Survey and a Randomized Controlled Trial to Evaluate the Efficacy of an Enhanced HIV Voluntary Counseling and Testing in Reducing HIV-related Behaviors Targeting Regular Male Sex Partners among Men Who Have Sex with Men in China [Ph.D.]. Ann Arbor: The Chinese University of Hong Kong (Hong Kong); 2015.
8. Liu Y. An Exploration of HIV Testing, Linkage-to-care and Antiretroviral Therapy Initiation among Chinese Men Who Have Sex with Men [Ph.D.]. Ann Arbor: Vanderbilt University; 2015.
9. Li J. A randomized controlled study to evaluate the efficacy of a positive psychology and social networking intervention in reducing depressive symptoms among HIV-infected men who have sex with men in China [Ph.D.]: The Chinese University of Hong Kong (Hong Kong); 2017.
10. Li M. Social Networks and Lesbians' Mental Health: A Study of a Lesbian Community in Northeast China [Ph.D.]. Ann Arbor: The Chinese University of Hong Kong (Hong Kong); 2017.
11. Soo Hoong Yean F. Well-being of Chinese gay men in Hong Kong: Study of body image, self-objectification and internalized homophobia [Psy.D.]: Alliant International University; 2017.
12. Wu J. HIV/STIs related risk among middle aged and old MSM in Shenzhen, China [Ph.D.]: University of California, Los Angeles; 2017.
13. Cao W. Factors and potential mental health impact of HIV disclosure to regular female sex partners among HIV-positive men who have sex with men and women in China [Ph.D.]: The Chinese University of Hong Kong (Hong Kong); 2018.
14. Yang X. Prevalence and Factors of Antiretroviral Therapy Usage among HIV-positive Men Who Have Sex with Men under a New Treatment Policy in China [Ph.D.]. Ann Arbor: The Chinese University of Hong Kong (Hong Kong); 2018.

## Traditional Chinese

### *Articles*

1. 陳姝蓉, 丁志音, 蔡芸芳, et al. 男同志感染者的親密關係--以情感層面為主的探討. 中華心理衛生學刊. 2004 200412;17 卷(4 期):頁 97-126.
2. 蔡春美, 徐森杰. 是「愛」還是「礙」? 初探愛滋對感染身分相異男同志伴侶互動關係之影響 [The Exploration of the Dilemma Relationship between HIV Serodiscordant Gay Couples]. 臺大社會工作學刊. 2009 200906(19 期):頁 1-40.
3. Hou) 侯 C-N. Support from Cyber Brokeback Mountain: Exploring the Interplay of the Internet and Social Support on Married Bisexual Men in Taiwan 臺灣已婚雙性戀男子之網路使用與尋求社會支持之關聯性研究. 性學研究 (Studies in Sexuality). 2011;2(1):2:1 2011.01[民 100.01],頁 1-41.
4. 梁曉藍 (Hio-Lam Leung), 黃俊豪 (Jiun-Hau Huang). 臺灣女同志之指險套使用盛行率與相關因素探討 [Prevalence and Associated Factors of Findom Use among Women Who Have Sex with Women in Taiwan]. 臺灣公共衛生雜誌. 2013 201302;32 卷(1 期):頁 31-41.
5. 李芳盈, 林垠翰, 金家玉, et al. 同志三溫暖場域推動「全程戴套」LED 宣導看板計畫之評價. 疫情報導. 2014 20141209;30 卷(23 期):頁 472-479.
6. 李芳盈, 簡子翔, 蕭惠心, et al. 資訊需求與暴露的族群間差異: 對男男間性行為者進行愛滋防治的啟示 [Group Differences in the Need for Messages and Exposure to Messages: Implications for HIV/AIDS Prevention and Control among Men Who Have Sex with Men]. 臺

灣公共衛生雜誌. 2014 201404;33 卷(2 期):頁 209-219.
7. 潘淑滿，游美貴．同志伴侶暴力及其求助之研究．臺大社會工作學刊. 2016 201612(34 期):頁 129-172.
8. 崔樂．「被出櫃」：中國同志學生遭受的身分焦慮與校園霸凌．性學研究. 2017 201707;8 卷(1 期):頁 89-118.
9. 鍾道詮，李大鵬．社會排除經驗對男同志心理健康的影響．中華心理衛生學刊. 2017 201703;30 卷(1 期):頁 37-68.
10. 侯政男．社群網絡的分手展現：男同志面對戀愛關係終止之臉書使用行為初探．性學研究. 2018 201801;8 卷(2 期):頁 35-62.
11. 李珈旻，孫可書，徐森杰．男同志藥癮減少傷害團體之實踐經驗．東吳社會工作學報. 2018 201806(34 期):頁 91-105.
12. 楊喬羽，沈瓊桃．家庭接納對成年期同性戀及雙性戀者身心健康之影響 [The Effect of Family Acceptance on the Health of Lesbian, Gay and Bisexual Adults]．臺灣公共衛生雜誌. 2018 201808;37 卷(4 期):頁 453-463.
13. 侯政男．彩虹雲端世界的使用與滿足：臺灣已婚雙性戀男性之行動社交網絡運用及尋求社會支持之關連性研究．性學研究. 2019 201907;10 卷(1 期):頁 1-27.
14. 王雅慧，李名蟬．運用 Watson 關懷理論於男同性戀者降低反覆自殺行為之護理經驗．護理雜誌. 2019 201904;66 卷(2 期):頁 107-114.

### *Theses*

1. 賴淑芬 (Lai Shu-Fen). 臺灣男同志三溫暖顧客感染 HIV 之分子流行病學研究 (Molecular epidemiology of HIV-1 infection in men who have sex with men from gay saunas in Taiwan) [Master of Philosophy]: 國立陽明大學; 2003.
2. 蘇羽湘 (Su Yu-Hsiang). 同性戀者自我認同對於休閒活動選擇之研究 ：以社會支持為調節變項 (Homosexual's self-identity and leisure choice : social support as moderator) [Master of Philosophy]: 觀光事業學系; 2013.
3. 陳偉霖 (Chen Wei-Lin). 男同志愛滋篩檢障礙之探討 (Analysis of HIV/AIDS Test Barriers within Male Homosexual Groups) [Master of Philosophy]: 醫務管理學系; 2013.
4. 魯世博 (LU Shih-Po). 男同性戀者親密關係暴力之初探 (Intimate violence experience in gay relationships) [Master of Philosophy]: 國立暨南國際大學; 2013.
5. 陳彤昀 (Chen Tung-Yun). 同性戀壓力源、壓力因應策略與心理健康狀態之調查研究－以身分認同程度高低者為例 (The Research of Lesbian and Gay Male Stressor, Stress Coping Strategy and Mental Health Status Survey－Case in the High and Low Degree of Homosexual Identity) [Master of Philosophy]: 中國文化大學; 2014.
6. 陳昶勳 (Chen Chang-Hsun). 男同志俱樂部藥物使用防治教育成效評估 [Doctor of Philosophy]. 台北市: 國立臺灣師範大學; 2016.

## Simplified Chinese

### *Articles*

[1]李秀芳，张北川，刘殿昌，等.男性同性性接触者高危性行为与性病的调查研究[J].中华皮肤科杂志，2001，34(3):189-191.
[2]张北川，李秀芳，史同新，等.2001 年 1109 例男男性接触者性病艾滋病高危行为监测与调查[J].中华皮肤科杂志，2002，35(03):25-44.

[3]曲书泉，张大鹏，吴玉华，等.东北某地男同性恋者艾滋病病毒/艾滋病的知识态度行为调查[J].中国艾滋病性病，2002，8(06):338-340.
[4]曲书泉，张大鹏，吴玉华，等.东北某地男同性恋者性行为及 HIV 感染流行病学研究[J].中国性病艾滋病防治，2002，8(3):145-147.
[5]宁镇, 徐亮, 康来仪, 等.45 例男性同性恋艾滋病感染危险因素调查[J].上海预防医学杂志，2003，15(09):454-456.
[6]郑煜煌，龚国忠.男男性接触者和艾滋病[J].中国现代医学杂志，2003(02):32-34.
[7]杨振发，房思宁，蔡文德，等.男-男性接触者梅毒和 HIV 感染及性行为调查[J].中国公共卫生，2003(11):16-17.
[8]张敬东，颜洪海，李秀芳，等.同性固定性伴侣对男男性接触者 AIDS 高危行为的影响[J].中国艾滋病性病，2003，9(02):84-86.
[9]陈培明.同性恋,性病与健康教育[J].中国皮肤性病学杂志，2003，17(01):68-69.
[10]史同新，张北川，李秀芳，等.中国不同地区男男性接触者艾滋病高危性行为对比研究[J].中国麻风皮肤病杂志，2003，19(04):335-337.
[11]张北川，李秀芳，吴绍文，等.中国大陆当今男同/双性爱者心理卫生与相关状况调查[J].中国性科学，2003(03):16-18.
[12]张北川.中国男同性爱者现状与艾滋病干预[J].预防医学情报杂志，2003，19(S1):1-2.
[13]林旭凯，刘奕广，黄广文，等.92 例男同性性接触者 STD/HIV 及相关行为调查[J].现代预防医学，2004，31(04):618-619.
[14]王继学，佟伟，李瑞，等.男男性接触者 HIV、STD 血清及行为学调查[J].中国公共卫生，2004，20(11):101-102.
[15]王全意，LGe，RossMichaelW.男男性接触者的危险性行为:一项通过互联网的调查[J].中国艾滋病性病，2004，10(5):335-337.
[16]郑迎军，王再幸，许娟，等.男男性接触者社会心理特征[J].中国行为医学科学，2004，13(06):59-61.
[17]吴绍文，张北川.男男性接触者与艾滋病[J].中华实验和临床病毒学杂志，2004，18(04):88-91.
[18]张北川，赵旭传，吴绍文，等.男男性接触者中安全套使用状况分析[J].中国麻风皮肤病杂志，2004(02):139-140.
[19]兰亚佳，顾仪，王斌，等.男-男性行为者的行为特征分析[J].四川大学学报(医学版)，2004(03):372-375.
[20]陶晓燕，蔡文德，蔡于茂，等.深圳市 114 例男性同性恋者高危行为调查[J].现代预防医学，2004，31(02):247-248.
[21]谷渊，曲鹏，宿鲁，等.沈阳市男同性恋者性病艾滋病相关 KABP 分析[J].中国公共卫生，2004，20(05):65-66.
[22]朱明泉，张北川，李秀芳，等.中国男男性接触者年龄与艾滋病高危性行为关系的研究[J].中华皮肤科杂志，2004，37(11):15-17.
[23]吴绍文，张北川，李秀芳.中国男同/双性爱者 AIDS 高危性行为监测与比较[J].中国艾滋病性病，2004(05):332-334.
[24]刘惠，刘英，肖雅，等.北京市部分男男性接触者 STD / AIDS 知识、态度、信念、行为调查[J].中国艾滋病性病，2005，11(4).
[25]杨春梅，王晓东，杜树平，等.成都地区男男性行为者的性病就医意向调查[J].寄生虫病与感染性疾病，2005，3(1):26-27.
[26]何群，王晔，林鹏，等.广东省广州市男同性性接触人群艾滋病 KAP 调查[J].疾病控制杂

志，2005，9(02):106-108.
[27]许毅，施卫星，胡少华.杭州市男性同性恋者性行为与艾滋病知识知晓率调查[J].中华预防医学杂志，2005，39(01):38-40.
[28]邵长庚，曹宁校.男男性接触和性传播疾病[J].中华皮肤科杂志，2005(05):259-261.
[29]郑迎军，许娟，张洪波.男男性接触者焦虑、抑郁与艾滋病高危性行为的关系[J].中国心理卫生杂志，2005，19(10):57-59.
[30]王明，邓艳红，李红卫，等.男男性接触者性病就医意向调查分析[J].中国艾滋病性病，2005(04):280-281.
[31]曹宁校，蒋娟，张国成，等.男男性行为者对就医环境的需求[J].中国艾滋病性病，2005，11(04):277-279.
[32]蔡文德，冯铁建，谭京广，等.男同性恋者行为特征和 STD/HIV 感染的调查[J].现代预防医学，2005，32(04):328-330.
[33]宋丹，陶晓燕，蔡文德，等.深圳市男同性恋者性行为调查及艾滋病干预[J].中国热带医学，2005，5(04):877-879.
[34]曹宁校，蒋娟，王明，等.同伴教育对男男人群性病知识的影响[J].中国性科学，2005，14(07):5-7.
[35]张北川，储全胜.同性爱与艾滋病[J].中华流行病学杂志，2005，26(05):320-322.
[36]李继钊，解乐荣，李瑞英，等.威海市男性同性恋者艾滋病相关行为调查[J].预防医学情报杂志，2005，21(06):721-722.
[37]曹宁校，蒋娟，宗文凯，等.在 MSM 人群中开展性病知识的同伴教育效果研究[J].中国艾滋病性病，2005(06):431-435.
[38]朱明泉，张北川，李秀芳，等.中国大陆样本人群男男性接触者受教育程度与艾滋病高危行为关系的研究[J].宁夏医学杂志，2005(01):27-29.
[39]田小兵，张建新，张灵麟，等.中小城市男男性接触者 HIV 感染行为调查[J].中国公共卫生，2005(11):11-12.
[40]储全胜，张北川，马铁成，等.东北某市男男性接触者安全套使用情况调查[J].中国艾滋病性病，2006，12(04):330-332.
[41]陆朝国，袁飞，石作宏，等.贵阳市男男同性恋人群 HIV 感染状况及有关艾滋病的 KABP 调查研究[J].贵州医药，2006，30(03):202-204.
[42]王亮，张婕，刘飞，等.黑龙江省某市区男男性接触者一般情况和 HIV 高危行为调查[J].中国艾滋病性病，2006，12(04):339-340.
[43]杨慧，阮师漫，王美花，等.济南市部分男性同性恋者艾滋病相关知识、危险行为与感染状况调查[J].预防医学论坛，2006，12(03):291-292.
[44]羊海涛，丁建平，陈国红，等.江苏省部分城市男男性接触者行为学特征分析[J].江苏预防医学，2006，17(01):1-4.
[45]曹宁校，张津萍，夏强，等.江苏省部分城市男男性行为者 HIV/STD 感染情况研究[J].中国艾滋病性病，2006，12(02):123-126.
[46]胡强，卢飞豹，龚俊平，等.江西省南昌市男男同性恋性病/艾滋病知信行调查[J].中国健康教育，2006，22(09):647-649.
[47]陆朝国，袁飞，石作宏，等.男男同性恋人群 HIV 感染调查[J].中国公共卫生，2006，22(11):1320-1321.
[48]齐淑贞，张国成，王千秋.男男性接触者男性性工作者与 HIV/STD 的传播[J].中国艾滋病性病，2006(01):89-91.
[49]李瑞，佟伟，张北川，等.男男性接触者 STI、AIDS 危险行为调查[J].中国公共卫生，2006，

22(05):522-523.
[50]郑迎军, 许娟, 张洪波.男男性接触者性伴网络特征与 HIV 传播[J].中国公共卫生, 2006, 22(05):531-533.
[51]李新旭，阮玉华.男男性接触者与 HIV 有关的行为学和 STD 感染状况[J].现代预防医学，2006，33(12):2320-2322.
[52]赵江宁，关瑞剑，罗湛滨，等.男男性接触者直肠肛门性传播疾病 43 例临床分析[J].岭南皮肤性病科杂志，2006(03):201-202.
[53]钱跃升，傅继华，毕振强.男男性行为与艾滋病[J].中国艾滋病性病，2006(06):583-584.
[54]曹宁校.男男性行为者的性病感染与控制[J].公共卫生与预防医学，2006(05):46-48.
[55]廖玫珍，刘学真，傅继华，等.山东省男性同性恋者综合行为监测情况分析[J].中国艾滋病性病，2006，12(06):530-532.
[56]赖永珲，蔡于茂，曾序春，等.深圳地区男男性接触者性病艾滋病知识及相关高危性行为调查[J].岭南皮肤性病科杂志，2006(02):146-149.
[57]曾惠芳，秦彦珉，叶宝英，等.深圳男同性恋性病和艾滋病感染状况调查[J].中国热带医学，2006，6(09):1686-1688.
[58]杨浩，周华，尹平，等.深圳市男男性接触者艾滋病知识态度行为调查[J].医学与社会，2006，19(07):18-19.
[59]吴炽煦，余庆，朱长才，等.生活化同伴教育对 MSM 人群艾滋病知识态度的影响[J].公共卫生与预防医学，2006(05):16-19.
[60]钟柳青, 吕繁.我国男男性接触人群的特征及艾滋病流行状况[J].中国艾滋病性病, 2006, 12(05):484-486.
[61]郑迎军，张洪波.我国男男性接触者 HIV/STIs 感染状况及高危性行为研究进展[J].中国艾滋病性病，2006(05):487-488.
[62]何建刚，豆正东，吴忠恕.芜湖市及其周边地区男男性接触者性行为学调查[J].安徽预防医学杂志，2006，12(06):353-355.
[63]曾婧，余庆，吴炽煦，等.武汉市 93 名男男性接触者艾滋病相关知识需求的调查[J].公共卫生与预防医学，2006，17(04):126-127.
[64]冯赟，张瑜，张险峰，等.武汉市 96 例男男性接触者艾滋病相关知识情况的调查[J].湘南学院学报，2006(01):65-66.
[65]吴炽煦，余庆，叶方立，等.武汉市男男性接触者艾滋病知信行现状初报[J].中国艾滋病性病，2006，12(03):233-235.
[66]李仕梁，张万宏，石卫东，等.武汉市同性恋酒吧男-男性接触者 HIV 感染高危行为现状调查[J].中国性科学，2006，15(12):11-14.
[67]冯赟，张瑜，张险峰，等.武汉市同性恋酒吧男男性接触者 HIV 高危行为的现况调查[J].武汉大学学报(医学版)，2006，27(03):315-318.
[68]李翔，王百锁，李秀芳，等.西安市部分男男性接触者 AIDS 高危行为分析[J].中国公共卫生，2006，22(05):520-521.
[69]郑迎军，许娟，张洪波，等.亚文化特征对男男性接触者高危性行为的影响[J].现代预防医学，2006，33(09):1526-1528.
[70]刘惠，汪宁，刘康迈，等.中国男男性接触者和 STD/AIDS 防治[J].中国艾滋病性病，2006(01):85-88.
[71]田小兵，吉渝南，张麟灵.中小城市男男性接触者艾滋病防治策略探索[J].中国艾滋病性病，2006，12(04):336-338.
[72]张娟娟，田小兵.中小城市男男性接触者艾滋病知识情况调查[J].川北医学院学报，

2006(06):511-513.
[73]史靖靖，易于颦，刘袁君，等.中小城市男男性接触者艾滋病知识水平与行为调查[J].川北医学院学报，2006，21(04):312-314.
[74]田小兵，吉渝南.中小城市男男性接触者安全套使用状况分析[J].中国公共卫生，2006，22(11):1316-1317.
[75]甘春霞，张娟娟，罗炎平，等.中小城市男男性接触者网络接触与高危行为的关系研究[J].川北医学院学报，2006，21(04):315-317.
[76]丁贤彬, 冯连贵, 徐静, 等.1000 名男男性接触者性行为状况调查[J].热带医学杂志, 2007，7(10):1011-1013.
[77]马爱兵，安晓静，余慧芬，等.2005 年云南省 78 例同性恋 HIV 抗体检测结果分析[J].中国艾滋病性病，2007，13(01):60-61.
[78]陈世海，周吉，朱建靖.2006 年南宁市部分男男性接触者性传播疾病感染状况调查[J].预防医学论坛，2007，13(09):772-774.
[79]陈世海，周吉，朱建靖.2006 年南宁市男男性接触者艾滋病相关知识、行为调查[J].中国健康教育，2007，23(08):576-578.
[80]钱跃升，张晓菲，陶小润，等.2006 年山东省部分男男性接触者艾滋病相关信息调查[J].预防医学论坛，2007，13(12):1074-1075.
[81]刘惠，汪宁，张琴，等.416 名男男性接触者 HIV 和梅毒感染状况及性行为特征[J].中国艾滋病性病，2007，13(03):230-232.
[82]陈波，王颖，张建新，等.MB 和一般同志人群安全套使用情况比较及影响因素的调查分析[J].现代预防医学，2007(19):3604-3605.
[83]闫红梅, 赵东辉, 金鹏.MSM 人群对人体润滑剂选用偏好的调查分析[J].中国艾滋病性病, 2007，13(05):466-467.
[84]邢建民，武锋，郑军，等.不同活动场所男男性接触人群艾滋病高危行为比较研究[J].中国艾滋病性病，2007，13(04):347-349.
[85]王岚，汤后林，张大鹏，等.不同性取向的男男性接触者行为特征分析[J].中国艾滋病性病，2007，13(02):123-126.
[86]王翠华，姜明杰，卢国际，等.承德市男男性接触者艾滋病感染情况及性行为方式的研究[J].中国卫生检验杂志，2007(05):883-884.
[87]何启卓，郑会民，钟莹，等.广州市同性恋者性病相关知、信、行调查[J].海峡预防医学杂志，2007，13(05):39-40.
[88]陆朝国，杨金芝，袁飞，等.贵州省黔东南州某地男男同性恋人群调查研究[J].贵州医药，2007，31(05):462-463.
[89]陈树昶，罗艳，程洁，等.杭州市男男性接触者艾滋病风险监测结果分析[J].疾病监测，2007，22(03):175-177.
[90]许娟，吴泽俊，张洪波，等.合肥市男男性行为者 HIV 和梅毒感染与社会及性伴特征[J].现代预防医学，2007，34(16):3009-3011.
[91]张洪波，王坚杰，郑迎军，等.合肥市男男性行为者自杀行为与社会特征关系[J].中国公共卫生，2007，23(09):1027-1029.
[92]许娟，张洪波，郑迎军，等.合肥市青年男男性行为者梅毒和 HIV 感染状况分析[J].中华行为医学与脑科学杂志，2007，16(03):205-207.
[93]邢建民，张孔来，陈曦，等.湖南省男男性接触人群艾滋病相关知识及性行为状态调查[J].中华预防医学杂志，2007，41(06):511-513.
[94]阮师漫，杨慧，朱艳文，等.济南市部分男男性接触者艾滋病相关行为研究[J].疾病监测，

2007，22(09):607-609.
[95]朱艳文，阮师漫，杨慧，等.济南市部分男男性接触者艾滋病相关知识、危险行为与感染状况调查[J].预防医学论坛，2007，13(06):490-492.
[96]王明，邓艳红，李红卫，等.昆明市男男性接触者不同场所活动人群危险性行为状况分析[J].中国健康教育，2007(07):513-515.
[97]邓艳红，王明，董海英，等.昆明市男男性行为人群艾滋病高危行为调查[J].中国公共卫生，2007，23(10):1237-1239.
[98]古丽萍，李志远，李莹，等.兰州市男男性接触者艾滋病知识知晓率及性行为危险性调查[J].中国皮肤性病学杂志，2007(11):682-683.
[99]冯连贵，丁贤彬，卢戎戎，等.重庆市男男性接触人群高危行为和性病艾滋病感染率调查[J].热带医学杂志，2007(05):483-486.
[100]孙智霞，林素芳，温明权，等.男男性接触人群性病艾滋病相关现况调查[J].现代预防医学，2007(21):4130-4132.
[101]孟爱群，宁秀君，牛惠敏，等.男男性接触人群状况及行为干预研究[J].医学动物防制，2007，23(08):640-641.
[102]林爱红，尹平，蔡文德，等.男男性接触者艾滋病感染危险因素调查[J].中国热带医学，2007，7(01):168-169.
[103]曾婧，余庆，许珊丹，等.男男性接触者艾滋病相关态度及行为调查[J].中国公共卫生，2007(12):1422-1423.
[104]朱军礼，张洪波，郑迎军，等.男男性接触者梅毒感染危险因素病例-对照研究[J].中国公共卫生，2007，23(02):131-133.
[105]钱跃升，傅继华，张晓菲，等.男男性接触者人群艾滋病防治相关信息定性研究[J].现代预防医学，2007，34(17):3281-3282.
[106]徐永芳.男男性接触者性病艾滋病现状及预防[J].现代预防医学，2007，34(11):2089-2090.
[107]宁秀君，孟爱群，芦飞，等.男男性接触者一般状况及艾滋病知信行调查[J].中国健康教育，2007，23(06):428-430.
[108]师伟，李新旭，阮玉华，等.男男性接触者异性性行为特征的调查研究[J].中国艾滋病性病，2007(01):33-36.
[109]廖留妹，张北川，李秀芳，等.男男性接触者中异性爱者与素质性同性爱者艾滋病高危性行为比较[J].中华流行病学杂志，2007，28(09):844-847.
[110]许娟，张洪波，郑迎军，等.男男性行为者 HIV 自愿咨询检测需求与利用[J].中国公共卫生，2007，23(09):1040-1042.
[111]郑迎军，张洪波，许娟，等.男男性行为者艾滋病知识及高危性行为调查[J].中国公共卫生，2007，23(09):1050-1051.
[112]于宗富，张朝.男同性恋者心理健康状况调查[J].中国现代医学杂志，2007，17(18):2291-2293.
[113]蔡军，徐群英，黄永真，等.南昌市男男同性恋者艾滋病知识知晓率及相关行为调查[J].中国医药导报，2007(17):158-168.
[114]田小兵，吉渝南，张麟灵.南充市男男性行为者安全套使用情况分析[J].中国艾滋病性病，2007，13(06):550-552.
[115]曾春娥，黄水群，田利光，等.女女性接触者/群体及艾滋病相关危险行为[J].中华流行病学杂志，2007，28(03):294-296.
[116]于宗富，张朝.山东男同性恋者社会生活状况自我感觉调查[J].现代预防医学，2007，34(20):3932-3934.

[117]钱跃升，张晓菲，陶小润，等.山东省部分男男性接触者艾滋病防治相关知识、态度和行为调查[J].中国健康教育，2007(09):665-667.
[118]陈琳，冯铁建，蔡文德，等.深圳市男男性行为者 STD/AIDS 感染情况研究[J].中国艾滋病性病，2007，13(06):547-549.
[119]马骏，郭剑.天津市男同性恋人群高危性行为与性病网络调查[J].现代预防医学，2007，34(20):3928-3929.
[120]马小燕，张启云，贺雄，等.同伴推动抽样法调查男男性接触人群艾滋病病毒/性传播疾病感染状况及相关行为[J].中华流行病学杂志，2007，28(9):851-855.
[121]杨淑娟，木合塔，李晓庆，等.乌鲁木齐市男男性接触者艾滋病知信行调查[J].现代预防医学，2007(24):4624-4625.
[122]朱军礼，张洪波，吴红花.学生中 122 名男男性行为者 HIV/STD 高危行为及其感染状况分析[J].中国艾滋病性病，2007，13(04):350-352.
[123]张建波，黄志威，KongT.Sk，等.云南省农村地区男男性接触者与 HIV 相关的风险行为[J].中国艾滋病性病，2007，13(5):441-443.
[124]傅利军，方益荣，郭天英.浙江省绍兴市男男性接触者性行为特征调查[J].疾病监测，2007(12):818-819.
[125]王全意，黎新宇，索罗丹，等.中国男男性接触者危险性行为和罹患艾滋病的风险[J].中国艾滋病性病，2007，13(02):187-188.
[126]邢建民，张孔来.中国男男性行为人群艾滋病相关知识知晓率调查回顾[J].中国艾滋病性病，2007，13(05):500-501.
[127]彭焱，郑海鸥，潘传波.重庆地区男性同性恋人群艾滋病防治知识与行为调查分析[J].现代医药卫生，2007(19):2986-2988.
[128]饶新民，熊鸿燕，丁贤彬，等.重庆市男男性行为者不安全性行为状况及其影响因素研究[J].重庆医学，2008(20):2335-2337.
[129]何勤英，吴学庆，韩德琳，等.2004-2007 年成都市男男性行为人群 HIV 感染与危险行为监测结果分析[J].职业卫生与病伤，2008(04):222-224.
[130]周宁，柳忠泉，柏建芸，等.2006 年天津市部分 40 岁以下青年男男性行为者艾滋病知晓率和行为学调查分析[J].中国艾滋病性病，2008，14(05):479-481.
[131]周生建，潘传波，孟晓容，等.重庆市男男性接触者性行为与 HIV 感染相关性研究[J].中国感染控制杂志，2008，7(06):381-384.
[132]朱秋映，刘伟，蓝光华，等.2007 年广西部分男男性行为者艾滋病知识及行为调查[J].应用预防医学，2008，14(04):206-208.
[133]卢红艳，马小燕，刘彦春，等.200 名男男性行为者 HIV/STD 现患率及影响因素调查[M].2008.467-470.
[134]郭立燕.MSM 人群艾滋病相关 KAP 调查[J].中国热带医学，2008(04):634-636.
[135]王晨，梁红元，杨烨，等.北京市男男性行为者 HIV 感染及影响因素的调查研究[J].中国艾滋病性病，2008，14(06):552-557.
[136]郑建东，吴尊友，庞琳，等.大学在校学生男男性接触者性取向自我认同与艾滋病相关危险因素定性研究[J].中国艾滋病性病，2008，14(03):258-260.
[137]秦精爽.甘肃省某市 MSM 人群艾滋病知识知晓情况及相关行为调查分析[J].卫生职业教育，2008(18):109-110.
[138]秦精爽.甘肃省某市 MSM 人群艾滋病知识知晓情况及相关行为调查分析关丌为调垒分析[J].卫生职业教育，2008，26(18):109-110.
[139]周健，祝继，黄斌，等.贵阳市两城区男男性行为人群 AIDS/STDHBVHCV 感染状况及

高危行为调查分析[J].中国艾滋病性病，2008，14(1):47-48,51.
[140]张付志.邯郸市男男同性恋艾滋病知识态度行为调查[J].实用预防医学, 2008, 15(02):424-425.
[141]郭巍，宋爱军，孟洪德，等.河北省廊坊市男男性行为人群艾滋病/性传播疾病危险行为与感染率现况调查[J].中华流行病学杂志，2008(06):545-547.
[142]郑建东，庞琳，吴尊友.互联网对男男性接触者危险性行为的影响及在艾滋病防治中的应用[J].中国健康教育，2008(04):293-295.
[143]胡锦流，吴丽萍，张兆辉，等.淮安市男男性接触者 STD/AIDS 相关知识与高危性行为及感染情况的调查分析[J].公共卫生与预防医学，2008(05):44-45.
[144]史同新，张北川，李秀芳，等.婚姻状况对男男性行为者的艾滋病高危性行为影响研究[J].中国艾滋病性病，2008，14(05):475-478.
[145]张昌庆，阮师漫，石志林，等.济南市 MSM 和暗娼人群 AIDS 相关知识和危险行为调查[J].中国艾滋病性病，2008，14(01):55-57.
[146]朱晓艳，康殿民，阮师漫，等.济南市男性同性恋人群心理健康状况研究[J].现代预防医学，2008，35(20):3987-3990.
[147]高淑艳，贾晓明.近 15 年来国内同性恋的研究概况[J].中国健康心理学杂志，2008(04):461-463.
[148]史同新，张北川，李秀芳，等.经济收入对男男性接触者艾滋病高危性行为的影响研究[J].中华流行病学杂志，2008，29(5):426-429.
[149]王毅，张洪波，张光贵，等.绵阳市 MSM 人群行为及生物学监测结果分析[J].现代预防医学，2008，35(19):3780-3783.
[150]王毅，张洪波，张光贵，等.绵阳市男男性行为人群艾滋病性病健康教育及行为干预效果研究[J].预防医学情报杂志，2008，24(12):962-967.
[151]刘励颖.牡丹江市 2006 年男男性行为人群艾滋病高危行为调查[J].医学动物防制, 2008, 24(07):534-535.
[152]王海龙，张旻，胡清海，等.男男性接触者 HIV/STD 感染及性行为调查[J].中国公共卫生，2008(08):995-997.
[153]蔡于茂，潘鹏，刘惠，等.男男性接触者艾滋病风险感知及安全套使用调查[J].中华疾病控制杂志，2008，12(06):538-540.
[154]冯素青，李生奎，刘晓松.男男性接触者艾滋病知识态度行为调查及干预模式初探[J].现代预防医学，2008(04):746-747.
[155]赖文红, 罗映娟.男男性接触者高危行为与 HIV 感染[J].预防医学情报杂志, 2008(03):186-189.
[156]朱军礼，张洪波，郑迎军，等.男男性接触者社会网络与艾滋病高危行为关系[J].中国公共卫生，2008(04):400-402.
[157]李东亮，梁红元，杨烨，等.男男性接触者首次同性性行为发生情况调查[J].预防医学情报杂志，2008(06):428-430.
[158]陶小润，张晓菲，于国防，等.男男性接触者性病艾滋病卫生服务需求调查[J].中国公共卫生管理，2008(01):94-95.
[159]储全胜，法仆，刘明华，等.男男性接触者性取向和 AIDS 相关态度、行为调查[J].职业与健康，2008，24(11):1068-1070.
[160]郭巍，宋爱军，赵学和，等.男男性行为人群 VCT 服务利用状况的定性研究[J].中国健康教育，2008(04):276-278.
[161]朱军礼，张洪波，吴尊友，等.男男性行为人群艾滋病危险行为同伴干预研究[J].中华预

防医学杂志，2008，42(12):895-900.
[162]张振开.男男性行为人群现状及艾滋病高危行为干预[J].应用预防医学，2008(S1):12-15.
[163]王毅，张洪波，张光贵，等.男男性行为者艾滋病防治需求和生物学检测影响因素调查[J].预防医学情报杂志，2008，24(03):161-165.
[164]毛茅，兰亚佳，周鼎伦，等.男-男性行为者肛交安全套使用调查[J].预防医学情报杂志，2008，24(07):493-495.
[165]李清春，杨烨，梁红元，等.男男性行为者异性性行为特征及影响因素分析[J].中国公共卫生，2008，24(12):1429-1431.
[166]郭剑，王媛，马骏.男同性恋者艾滋病检测影响因素结构方程模型分析[J].预防医学情报杂志，2008，24(10):805-807.
[167]徐永芳，周吉，陈世海，等.南宁市男男性接触者艾滋病相关知识及危险行为调查[J].预防医学情报杂志，2008(10):753-755.
[168]陈世海，周吉，朱建靖，等.南宁市男男性接触者行为学特征分析[J].现代预防医学，2008(05):902-904.
[169]吴健，陈磊，范惠俐，等.上海市男男性接触者人免疫缺陷病毒I型和梅毒感染现状以及性行为特征[J].诊断学理论与实践，2008(03):296-299.
[170]蔡于茂，刘惠，潘鹏，等.深圳市 458 例男男性接触者高危行为特征研究[J].华南预防医学，2008，24(02):13-15.
[171]蔡于茂，刘惠，潘鹏，等.深圳市桑拿、酒吧场所男性接触者 HIV 及梅素流行病学调查[J].现代预防医学，2008，35(12):2203-2205.
[172]邹华春，庞琳，吴尊友.使用互联网的男男性行为人群艾滋病/性传播疾病高危行为特征研究现状[J].中华流行病学杂志，2008(06):622-624.
[173]冯济富，林海江，裘丹红，等.台州地区男男性行为人群(MSM)HIV、梅毒、丙肝感染状况调查[J].中国卫生检验杂志，2008(09):1885-1886.
[174]冯济富，林海江，章亚夫，等.台州市男男性行为者艾滋病相关知识、行为和感染率调查[J].上海预防医学杂志，2008，20(11):531-533.
[175]陈舸，郑森兴.同性恋和男男性行为与艾滋病相关的研究进展[J].海峡预防医学杂志，2008，14(03):23-26.
[176]文小青.我国男男性接触者的行为特征与艾滋病流行状况[J].中国热带医学，2008(11):1978-1980.
[177]王志军，张军，马小剑，等.扬州市广陵区男男性接触者艾滋病性病防治知识和性传播疾病感染调查[J].江苏预防医学，2008(02):16-18.
[178]李秀芳，张北川，储全胜，等.以互联网为最主要性交往途径的男男性行为者 HIV/AIDS 相关性行为研究[J].中华流行病学杂志，2008，29(07):685-688.
[179]蔡高峰，马瞧勤，潘晓红，等.浙江省两城市男男性接触者艾滋病相关知信行及 HIV/STD 感染状况调查[J].中国预防医学杂志，2008(06):482-485.
[180]朱炜明，林海江，章亚夫，等.浙江省台州市男男性行为者艾滋病病毒/性传播感染的危险行为与性关系网络调查[J].中华流行病学杂志，2008(10):994-998.
[181]萧燕，李春梅，吕繁，等.中国 16 个城区男男性行为人群梅毒感染情况及影响因素分析[J].中华皮肤科杂志，2008(06):353-356.
[182]张北川，李秀芳，储全胜，等.中国 9 城市 2250 例男男性接触者 HIV/AIDS 相关状况调查概况[J].中国艾滋病性病，2008，14(06):541-547.
[183]郭巍，庞琳，吴尊友.中国 MSM 人群 AIDS/STD 防治工作进展及主要问题[J].中国艾滋病性病，2008(03):314-316.

[184]王万海，张凤民，张晓燕.中国 MSM 人群 HIV 感染者中流行的 HIV 生物学特点及研究进展[J].中国艾滋病性病，2008(01):93-95.
[185]高省，王丽艳，丁正伟，等.中国部分城市男男性行为人群性行为特征及 HIV 感染状况的研究[J].中国艾滋病性病，2008，14(06):548-551.
[186]萧燕，李春梅，吕繁，等.中国部分地区 MSM 人群安全套使用的变化情况及影响因素分析[J].中国艾滋病性病，2008，14(02):133-136.
[187]唐卫明，丁建平，闫红静，等.中国大陆 2001-2006 年 MSM 的性行为特征与 HIV/现症梅毒感染现状的 Meta 分析[J].中国艾滋病性病，2008，14(05):471-474.
[188]何群，王晔，林鹏，等.中国大陆男男性行为人群艾滋病防治所面临的挑战[J].中国艾滋病性病，2008，14(06):640-643.
[189]张北川，李秀芳，储全胜，等.中国九城市 2046 例男同性爱者与艾滋病相关状况调查概况[J].中国性科学，2008，17(08):6-10.
[190]陈秀元.中国同性恋研究:回顾与展望——对 1986～2006 年间 178 篇学术论文的文献综述[J].中国性科学，2008，17(11):30-35.
[191]欧阳琳，丁贤彬，周超，等.重庆市不同性取向的男男性接触者 HIV 高危性行为分析[J].华南预防医学，2008，24(02):16-19.
[192]刘芳，刘虹宏，周超，等.重庆市某区男男性接触者艾滋病综合干预效果评价[J].实用预防医学，2008(04):1055-1058.
[193]冯连贵，丁贤彬，卢戎戎，等.重庆市男男性接触者艾滋病病毒感染率及其影响因素[J].中华预防医学杂志，2008，42(12):870-874.
[194]李春梅，萧燕，刘建波，等.重庆市男男性行为者艾滋病病毒和梅毒的感染情况及其影响因素[J].中华流行病学杂志，2009，30(02):126-130.
[195]李春梅，贾玉江，丁贤彬，等.重庆市男男性行为者中异性性行为和 HIV 感染情况及其影响因素分析[J].中华流行病学杂志，2009，30(09):882-886.
[196]卢正祥，葛宁，高红茹，等.2007～2008 年盐城市男男同性恋人群艾滋病相关知识行为与感染状况调查[J].预防医学论坛，2009，15(08):702-703.
[197]杜桂英，蔡新，于海荣，等.2007 年聊城市男男性接触人群艾滋病高危险行为及 HIV 感染状况调查[J].预防医学论坛，2009，15(03):196-197.
[198]陈琳，蔡文德，谭京广，等.2007 年深圳市男男性行为者哨点监测情况分析[J].中国艾滋病性病，2009，15(04):418-419.
[199]孙茂利，李德钧，王锦，等.2008 年大连市男男性接触者有关情况及 HIV、HCV、梅毒、HBV 感染状况调查[J].预防医学论坛，2009，15(11):1074-1076.
[200]陈世海，朱建靖，李骏.2008 年广西南宁市部分男男性行为者健康体检情况分析[J].中国艾滋病性病，2009，15(03):305-307.
[201]李春霞，卢培能，唐作红，等.2008 年攀枝花市男男性接触者艾滋病行为监测[J].预防医学情报杂志，2009，25(08):649-652.
[202]马明昌，和西涛，耿玉芝.2008 年新泰市男男性接触者艾滋病相关知识、态度及行为调查[J].预防医学论坛，2009，15(12):1202-1203.
[203]郭燕，朱效科，夏建晖，等.204 例 MSM 人群 HIV/梅毒感染状况及行为学特征分析[J].中国艾滋病性病，2009，15(01):50-51.
[204]李辉，张北川，李秀芳，等.2250 例男男性接触者性伴与性传播感染率相关性研究[J].中华皮肤科杂志，2009，42(10):665-667.
[205]俸卫东，张英霞.250 名 MSM 人群艾滋病监测情况分析[J].医学动物防制，2009，25(05):356-358.

[206]王毅，张洪波，徐杰，等.MSM 艾滋病性病防治知识同伴干预效果评价[J].中国公共卫生，2009，25(05):631-633.
[207]王毅，张洪波，李志军，等.MSM 人群艾滋病性病防治行为干预效果评价[J].实用预防医学，2009，16(03):654-657.
[208]张政，李清春，李书明，等.北京市男男性行为人群队列随访中高危行为变化的调查研究[J].中国艾滋病性病，2009，15(06):586-588.
[209]汤后林，吕繁，张大鹏，等.不同招募场所的男男性行为者性网络特征研究[J].中华预防医学杂志，2009，43(11):965-969.
[210]欧阳琳，冯连贵，丁贤彬，等.采用同伴推动抽样法在重庆市男男性行为人群中开展 HIV 流行病学调查[J].中华流行病学杂志，2009，30(10):1001-1004.
[211]韩德琳，王晓东，梁娴，等.成都市不同活动场所 MSM 人群艾滋病知识态度行为比较[J].现代预防医学，2009，36(15):2910-2912.
[212]李德钧，孙茂利，赵志杰，等.大连市 401 名 MSM 人群 HIV、梅毒、丙型肝炎感染状况分析[J].中国卫生检验杂志，2009，19(03):693-694.
[213]于增照，张北川，李秀芳，等.毒品使用对男同/双性爱者艾滋病高危行为的影响[J].中国预防医学杂志，2009，10(06):433-437.
[214]陈英，王发芝，张金波.费县 182 名男男性接触者艾滋病相关知识、态度、行为状况调查[J].中华疾病控制杂志，2009，13(04):479-481.
[215]蓝光华，刘伟，朱秋映，等.广西不同年龄段男男性行为人群艾滋病监测结果分析[J].预防医学情报杂志，2009，25(08):608-611.
[216]陈世海，曹宁校.广西南宁市男男性行为者艾滋病(性病)相关知识及求医行为[J].职业与健康，2009，25(23):2586-2588.
[217]赵鸿鹏，王国明，刘海英.哈尔滨市道里区自愿咨询检测中的男男性接触者调查报告[J].中国医药指南，2009，7(10):181-183.
[218]冯福，王召乾，黄淑平，等.海口市男男性行为人群艾滋病知识、态度、行为特征及 HIV/梅毒感染状况调查分析[J].现代预防医学，2009，36(15):2902-2903.
[219]席胜军，何玉芳.杭州市不同活动场所 MSM 人群艾滋病病毒感染现况研究[J].浙江预防医学，2009，21(09):1-2.
[220]郭巍，宋爱军，王斌，等.河北省廊坊市 MSM 人群艾滋病相关危险行为研究[J].中国艾滋病性病，2009，15(05):483-485.
[221]闫红梅，王开利，赵东辉，等.黑龙江省四市 MSM 人群艾滋病相关高危行为及其影响因素分析[J].中国艾滋病性病，2009，15(03):288-291.
[222]王开利，闫红梅，赵东辉，等.黑龙江省四市男男性行为人群 HIV/梅毒/HCV 感染状况及其影响因素分析[J].中国预防医学杂志，2009，10(10):907-909.
[223]陈燕，熊馥，解瑞青，等.黄石市 MSM 人群行为特征与艾滋病相关知识调查结果分析[J].公共卫生与预防医学，2009，20(05):115-117.
[224]邱柏红，李波，张新刚，等.吉林省 MSM 人群 HIV 和梅毒感染及性行为调查[J].中国艾滋病性病，2009，15(06):604-606.
[225]杨慧，阮师漫，朱艳文，等.济南市男男性接触者梅毒和人类免疫缺陷病毒感染状况分析[J].中国预防医学杂志，2009，10(08):740-742.
[226]杨慧，阮师漫，朱艳文，等.济南市男男性行为人群的特征和高危行为分析[J].中国艾滋病性病，2009，15(04):420-421.
[227]朱武通，葛志坚，夏中华，等.嘉兴市 MSM 人群艾滋病行为干预初步研究[J].中国艾滋病性病，2009，15(04):422-427.

[228]唐卫明，闫红静，刘晓燕，等.江苏省南京、苏州、扬州市男男性行为人群 HIV 感染因素的配比病例对照研究[J].中华流行病学杂志，2009，30(05):448-451.
[229]于增照，张北川，李秀芳，等.教育背景对男男性行为者艾滋病高危行为影响的研究[J].中华流行病学杂志，2009，30(05):524-525.
[230]王明，邓艳红，董海英，等.昆明男男性行为人群 2008 年行为监测调查分析报告[J].卫生软科学，2009，23(05):593-598.
[231]谯小伟，马玺之，李莹.兰州市 2008 年男男性接触(MSM)人群 HIV-自愿咨询检测(HIV-VCT)资料流行病学分析[J].卫生职业教育，2009，27(22):104-105.
[232]陈继军，李莹，高文龙，等.兰州市男男性行为人群艾滋病流行病学调查分析[J].中国艾滋病性病，2009，15(02):188-190.
[233]姚中兆，刘聪，王红方，等.两种干预模式对 MSM 人群干预效果分析[J].公共卫生与预防医学，2009，20(06):16-19.
[234]白玉，俸卫东，韦启后.柳州市 2007～2008 年 MSM 的 HIV、HCV、TP 感染调查[J].中国热带医学，2009，9(12):2275-2276.
[235]王毅，张洪波，李志军，等.绵阳市 MSM 人群艾滋病预防服务及规范性病诊疗促进效果[J].职业与健康，2009，25(15):1586-1588.
[236]王毅，张洪波，张光贵，等.绵阳市 MSM 人群性行为现状调查结果分析[J].现代预防医学，2009，36(03):473-475.
[237]王毅，张洪波，张光贵，等.绵阳市男男性行为人群生物检测与社会特征调查[J].实用预防医学，2009，16(02):375-377.
[238]李刚，张万宏，石卫东，等.男男同性恋人群利用 VCT 服务的现状及需求分析[J].现代预防医学，2009，36(18):3555-3556.
[239]安晓静，韩瑜，杨彦玲，等.男男性接触人群寻求获得性免疫缺陷综合征自愿咨询检测服务障碍的调查[J].中华预防医学杂志，2009，43(11):1038-1039.
[240]张曼，王新迪，杨阳.男男性接触者 HIV、梅毒、丙肝感染及行为调查[J].中国公共卫生，2009，25(09):1075-1076.
[241]梁良，陈志强，苗香芬，等.男男性接触者艾滋病感染状况调查分析[J].河北医药，2009，31(15):1991-1992.
[242]梁良，陈志强，苗香芬，等.男男性接触者艾滋病相关知识及行为调查[J].河北医药，2009，31(19):2654-2655.
[243]徐俊杰，张旻，楚振兴，等.男男性接触者包皮环切术接受意愿调查[J].中国公共卫生，2009，25(12):1414-1415.
[244]王毅，张洪波，李志军，等.男男性行为人群艾滋病检测促进及血清学检测结果分析[J].中国艾滋病性病，2009，15(05):486-489.
[245]修翠珍，刘明华，李秀芳，等.男男性行为人群的艾滋病知识行为干预与感染状况研究[J].预防医学论坛，2009，15(02):97-100.
[246]张洪波，朱军礼，吴尊友，等.男男性行为人群预防控制艾滋病同伴综合干预模式研究[J].中华预防医学杂志，2009，43(11):970-976.
[247]于增照，张北川，李秀芳，等.男男性行为者儿童期性虐待经历对艾滋病高危行为及心理影响[J].中华流行病学杂志，2009，30(01):14-17.
[248]史同新，张北川，李秀芳，等.男男性行为者中流动人口艾滋病高危行为研究[J].中华流行病学杂志，2009，30(07):668-671.
[249]徐永芳，李俊，陈世海，等.南宁市男男性接触者艾滋病相关危险行为和感染状况的调查[J].现代预防医学，2009，36(15):2913-2915.

[250]陈世海，杨泞珲，朱建靖.南宁市男男性行为人群网络使用情况调查[J].中国健康教育，2009，25(11):823-825.
[251]曲琳，杨景元，张星光，等.内蒙古自治区部分城市男男性行为者 HIV、梅毒感染情况调查[J].中华流行病学杂志，2009，30(10):1089-1090.
[252]姜珍霞，张西江，何丽丽，等.青岛市男男性行为人群 AIDS/STDHCV 感染状况及高危行为调查分析[J].中国艾滋病性病，2009，15(02):174-175.
[253]蔡于茂，刘惠，潘鹏，等.深圳地区 MSM 性伴数量及影响因素分析[J].中国艾滋病性病，2009，15(03):282-284.
[254]谭京广，蔡文德，陈琳，等.深圳市 MSM 人群不使用安全套原因调查[J].内蒙古医学杂志，2009，41(06):712-714.
[255]谭京广，陈琳，蔡文德，等.深圳市男男性行为人群艾滋病性病感染及相关危险行为研究[J].实用预防医学，2009，16(05):1638-1640.
[256]蔡于茂，洪福昌，冯铁建，等.深圳市男男性行为者艾滋病知晓率及异性性行为特征调查[J].中国热带医学，2009，9(10):2026-2046.
[257]曾序春，张玲华，刘惠，等.深圳市女同性恋者性行为特征和社会心理压力研究[J].中国计划生育学杂志，2009，17(09):529-531.
[258]王疆，郑建中.太原市部分男男性接触者相关行为调查[J].山西医科大学学报，2009，40(12):1088-1090.
[259]张丽芬，梅林，乔晓春，等.太原市男男性行为人群的认知态度对安全套使用行为和意向的影响[J].中国艾滋病性病，2009，15(02):124-127.
[260]徐志宏.铁岭市男男性接触者艾滋病相关知识行为调查[J].中国公共卫生，2009，25(02):226.
[261]阮师漫，杨慧，朱艳文，等.同伴推动抽样法在济南市男男性接触人群中的应用研究[J].疾病监测，2009，24(06):416-418.
[262]鲍宇刚，张艳辉，赵金扣，等.我国 14 城市男男性行为人群艾滋病感染状况及艾滋病知识、态度、行为研究[J].中华预防医学杂志，2009，43(11):981-983.
[263]刘昆仑，吴尊友，庞琳.我国男男性行为人群中安全套使用与防治艾滋病面临的挑战[J].中国艾滋病性病，2009，15(05):546-548.
[264]石卫东，李刚，杨涛，等.武汉市 456 例男男性接触者高危性行为及 HIV、梅毒与丙肝调查[J].医学与社会，2009，22(10):42-43.
[265]许骏，周旺，周敦金，等.武汉市男男性行为人群艾滋病干预效果评价[J].中国健康教育，2009，25(10):736-738.
[266]王梅，吴炽煦.武汉市青少年 MSM 人群艾滋病认知现状及健康干预效果评价[J].公共卫生与预防医学，2009，20(02):43-46.
[267]苗志峰，李晶，雷利民，等.银川市 312 名 MSM 人群艾滋病相关知识、行为状况调查[J].宁夏医科大学学报，2009，31(06):761-762.
[268]蔡于茂，刘惠，潘鹏，等.应用同伴推动抽样法对深圳地区男男性接触者 AIDS 相关知识和危险行为调查[J].华南预防医学，2009，35(01):4-7.
[269]史同新，张北川，李秀芳，等.与女性经常性交的未婚男男性行为者艾滋病高危性行为研究[J].中华流行病学杂志，2009，30(01):10-13.
[270]夏俊瑞，郑迎军，张洪波，等.中国大陆 7 城市 MSM 人群艾滋病知识知晓率调查[J].中国艾滋病性病，2009，15(03):285-287.
[271]蓝光华.中国大陆男男性接触者艾滋病防治现状研究进展[J].内科，2009，4(03):414-415.
[272]伍传仁.中国男男同性恋的研究现状[J].实用预防医学，2009，16(03):985-987.

[273]徐静，丁贤彬，冯连贵，等.重庆市 192 名在婚 MSM 人群 HIV、梅毒感染调查[J].中国热带医学，2009，9(11):2096-2097.
[274]韩梅，冯连贵，蒋岩，等.重庆市 2006-2008 年男男性行为人群 HIV-1 感染发病率调查[J].中华流行病学杂志，2009，30(09):878-881.
[275]郭晓嘉，丁贤彬，冯连贵，等.重庆市部分青年学生中男男性接触者性行为特征及 HIV 感染状况分析[J].华南预防医学，2009，35(06):36-37.
[276]冯连贵，丁贤彬，吕繁，等.重庆市男男性行为人群艾滋病干预效果初步研究[J].中华流行病学杂志，2009，30(01):18-20.
[277]沈鹏，王振维，潘传波，等.重庆市主城某区 MSM 人群 HIV/梅毒感染情况及影响因素分析[J].重庆医学，2010，39(08):956-958.
[278]周彩霞.遵义市男男性行为人群 HIV 感染状况调查[J].安徽预防医学杂志，2010，16(03):211-212.
[279]成国明，吕金伟，方艳姣，等.129 名高校男男性行为学生艾滋病相关知识和行为调查分析[J].中华疾病控制杂志，2010，14(06):487-489.
[280]陈世海，朱建靖，杨泞珲.2006—2008 年南宁市男男性接触者艾滋病及性传播疾病感染情况调查[J].职业与健康，2010，26(01):56-58.
[281]张庆娟，吕维冬，何金奎，等.2008 年-2009 年唐山市男男同性恋 HIV 抗体检测结果分析[J].中国卫生检验杂志，2010，20(05):1184-1185.
[282]张芙蓉，石兴瑞，杜桂英.2008 年聊城市男男性行为者艾滋病高危行为调查[J].预防医学论坛，2010，16(10):865-867.
[283]王毅，张洪波，徐杰，等.2008 年绵阳市男男性行为者家庭及朋友歧视与个体行为和心理健康的关系[J].预防医学论坛，2010，16(03):209-213.
[284]李瑞，佟伟，赵志杰.2009 年大连市男男性行为者艾滋病危险行为与 HIV 感染及影响因素调查[J].预防医学论坛，2010，16(12):1127-1129.
[285]钱跃升，刘忠诚，于贤亮，等.2009 年淄博市男男性行为人群艾滋病病毒感染状况与相关行为调查[J].预防医学论坛，2010，16(05):391-392.
[286]蔡梅英，龚诚华，戴丽萍，等.435 名男男性接触者性行为特征及 HIV、梅毒感染情况分析[J].华南预防医学，2010，36(03):28-30.
[287]刘业英，田斌，宋文炜.507 例男男性接触者基本人口学特征和 HIV、HCV 及梅毒感染率分析[J].实用预防医学，2010，17(04):788-790.
[288]李秀芳，张北川，于增照，等.6 城市 1295 例男男性行为者安全套使用相关状况调查概况[J].中国艾滋病性病，2010，16(05):449-452.
[289]陈官芝，李洋，王丽欣，等.72 名 50 岁及以上男男性行为者行为特征研究[J].中华流行病学杂志，2010，31(06):718-719.
[290]李林涛，齐杰，刘文忠.MSM 人群 HIV 感染状况及行为调查[J].现代预防医学，2010，37(13):2484-2486.
[291]张丹丹，张琰，李怀亮，等.MSM 人群性行为特征及血液感染检测结果分析[J].浙江预防医学，2010，22(08):1-3.
[292]米国栋，徐杰，斗智，等.MSM 中 HIV 感染者无保护肛交行为及其影响因素分析[J].中国艾滋病性病，2010，16(03):255-258.
[293]计国平，徐杰，姚晖，等.安徽省男男性行为人群艾滋病知识水平、行为特征和感染率分析[J].安徽预防医学杂志，2010，16(05):335-338.
[294]周振海，李书明，刘英杰，等.北京市 MSMHIV 和梅毒感染与性行为影响因素调查[J].中国热带医学，2010，10(01):10-12.

[295]刘英杰，周振海，姜树林，等.北京市男男性行为人群 HIV 和梅毒新发感染率与队列保持影响因素研究[J].中华流行病学杂志，2010，31(08):948-950.
[296]周振海，李书明，刘英杰，等.北京市男男性行为者行为因素和心理状态与 HIV 感染关系的研究[J].中华流行病学杂志，2010，31(03):273-276.
[297]许骏，周敦金，周旺，等.不同场所男男性行为人群性行为影响因素分析[J].中国公共卫生，2010，26(01):44-46.
[298]于增照，史同新，李秀芳，等.不同主要性交往场所与男男性行为者艾滋病高危行为关系的研究[J].中华流行病学杂志，2010，31(06):642-646.
[299]孟霞，谢朝梅，曾希鹏.常德市 MSM 人群艾滋病、性病感染状况调查[J].热带医学杂志，2010，10(07):884-885.
[300]邓斌，江华，王文渊，等.成都市大学生男男性行为人群艾滋病性病高危行为现状调查[J].现代预防医学，2010，37(08):1543-1546.
[301]米国栋，曾亚莉，黄惋莉，等.成都市男男性行为人群 HIV 感染者卫生服务利用现况调查[J].中国预防医学杂志，2010，11(04):328-331.
[302]刘立珍，李现红，何国平，等.成都市男性行为者艾滋病相关知识和高危性行为调查分析[J].中国全科医学，2010，13(26):2970-2973.
[303]周超，丁贤彬，刘芳，等.大学生男男性接触人群艾滋病相关知识行为状况调查[J].现代预防医学，2010，37(04):763-765.
[304]赵东辉，罗佳伦，高聪，等.东北某市 MSM 人群 STD/AIDS 高危行为及健康教育需求分析[J].中外医疗，2010，29(23):102-104.
[305]史同新，于增照，李秀芳，等.对男男性行为者中“买”性者艾滋病高危行为状况调查[J].中华流行病学杂志，2010，31(11):1223-1226.
[306]潘蕴蛟，陈舸，郑健，等.福建三城市 MSM 人群艾滋病相关知行情况调查分析[J].海峡科学，2010(10):263-264.
[307]任明，曲波，孙高.抚顺市男男性接触者艾滋病高危性行为统计分析[J].中国卫生统计，2010，27(01):20.
[308]全福才，赵淑玲，刘宝录，等.甘肃省天水市男男性接触者艾滋病防治策略研究[J].卫生职业教育，2010，28(11):133-134.
[309]张振开，文小青，陈伟，等.广西桂林市男男性行为人群艾滋病高危行为调查[J].疾病监测，2010，25(03):213-215.
[310]陈世海，朱建靖，李骏.广西南宁市男男性接触者艾滋病相关知识及行为学特征调查[J].中华疾病控制杂志，2010，14(02):130-133.
[311]文芳，钟斐，程伟彬，等.广州市男男性接触者 HIV 和现症梅毒感染状况及相关因素分析[J].华南预防医学，2010，36(02):19-23.
[312]王成，陈昂，石一鸣，等.广州市男性同性恋人群艾滋病防治相关信息定性研究[J].皮肤性病诊疗学杂志，2010，17(02):97-99.
[313]文小青，蒋基权，张振开，等.桂林市 315 名 MSM 艾滋病知识、高危行为及血清学调查[J].中国热带医学，2010，10(10):1194-1195.
[314]张振开，文小青，陈伟，等.桂林市男男性行为人群艾滋病感染危险因素的调查研究[J].中华疾病控制杂志，2010，14(09):845-847.
[315]张振开，文小青，陈伟，等.桂林市男男性行为人群艾滋病高危行为干预效果评价[J].预防医学情报杂志，2010，26(10):784-787.
[316]孙定勇，马彦民，聂玉刚，等.河南省两城市男男性接触人群艾滋病病毒感染调查[J].疾病监测，2010，25(11):874-876.

[317]赵英男，闫红梅.黑龙江省男男性行为人群多性伴行为相关情况分析[J].中国艾滋病性病，2010，16(04):379-381.
[318]刘盛元，王开利，姚嵩坡，等.黑龙江省男男性行为者中艾滋病相关知识、行为及 HIV 感染检出率调查[J].中华疾病控制杂志，2010，14(05):408-411.
[319]杨慧，王美花，阮师漫，等.济南市双性恋的男男性接触者性行为及 HIV、梅毒、HCV 感染调查[J].预防医学论坛，2010，16(10):874-875.
[320]郭巍，王斌，宋爱军，等.廊坊市部分 MSM 人群 HIV 检测咨询前后高危行为变化的分析[J].中国艾滋病性病，2010，16(03):269-272.
[321]孙定勇，马彦民，聂玉刚，等.两中心城市男男性接触者 HIV/HCV 和梅毒感染状况的研究[J].中国实用医刊，2010(21):0.
[322]白玉，倖卫东.柳州市不同性取向 MSM 性行为学特征分析[J].预防医学情报杂志，2010，26(04):250-253.
[323]白玉，倖卫东，韦启后.柳州市男男性接触者性行为学特征分析[J].医学动物防制，2010，26(02):103-105.
[324]段毓雯，张洪波，王晓冬，等.绵阳市和宜宾市男男性行为人群艾滋病高危因素状况的对比分析[J].中华疾病控制杂志，2010，14(12):1189-1192.
[325]赵西和，杨宏武，何静，等.绵阳市男男性行为人群艾滋病相关知识、行为及生物学监测分析[J].预防医学情报杂志，2010，26(11):876-878.
[326]马伟元，赵宸.男男性接触人群常见性传播疾病[J].中国麻风皮肤病杂志，2010，26(11):797-799.
[327]简迅.男男性接触人群预防艾滋病健康教育现状及影响因素[J].实用预防医学，2010，17(07):1469-1471.
[328]刘盛元，王开利，姚嵩坡，等.男男性接触者艾滋病相关知识、行为及信息资源分析[J].中国公共卫生，2010，26(07):889-890.
[329]闫红静，张敏，李建军，等.男男性接触者艾滋病性病感染状况及行为特征和干预策略[J].中国预防医学杂志，2010，11(12):1249-1252.
[330]陈树昶，罗艳，李西婷，等.男男性接触者安全套使用情况调查[J].疾病监测，2010，25(01):25-27.
[331]丁贤彬，冯连贵，徐静，等.男男性接触者同性性行为特征及安全性行为影响因素研究[J].中国预防医学杂志，2010，11(12):1197-1201.
[332]韩丹，李蓬.男男性接触者行为特征与艾滋病行为干预[J].河南预防医学杂志，2010，21(4):331-333,338.
[333]李东亮，李清春，李书明，等.男男性行为人群包皮环切术预防 HIV 意愿调查[J].中国公共卫生，2010，26(06):728-729.
[334]王毅，张洪波，徐杰，等.男男性行为人群的抑郁症状及相关因素[J].中国心理卫生杂志，2010，24(05):366-367.
[335]于茂河，程绍辉，朱效科，等.男男性行为人群对艾滋病自愿咨询检测需求与利用状况分析[J].中国预防医学杂志，2010，11(10):1017-1020.
[336]马佩珠，李锐.男男性行为人群感染 AIDS 危险行为变化趋势分析[J].疾病监测与控制，2010，4(03):148-149.
[337]吴琼海，裘丹红，沈伟伟，等.男男性行为人群研究中的社会网络应用分析[J].国际流行病学传染病学杂志，2010，37(02):143-145.
[338]王毅，张洪波，徐杰，等.男男性行为人群抑郁症状危险因素 Logistic 回归分析[J].预防医学情报杂志，2010，26(05):335-339.

[339]王毅，张洪波，徐杰，等.男男性行为人群抑郁状况调查[J].预防医学情报杂志，2010，26(08):597-600.
[340]周爽，冯连贵，丁贤彬，等.男男性行为人群中 HIV 感染者艾滋病相关高危行为特征分析[J].重庆医学，2010，39(02):215-217.
[341]薛芳辉，孙智霞，林素芳，等.男男性行为者高危行为特征和性病/艾滋病病毒感染状况调查[J].疾病监测，2010，25(01):54-56.
[342]陈章颖，王鸣，陈维清，等.男男性行为者高危行为特征研究现状[J].公共卫生与预防医学，2010，21(03):51-53.
[343]许娟，徐杰，米国栋，等.男男性行为者婚姻状况及其艾滋病高危行为比较[J].中华疾病控制杂志，2010，14(05):404-407.
[344]王毅，张洪波，徐杰，等.男男性行为者歧视经历及感受现状分析[J].实用预防医学，2010，17(4):636-639.
[345]王丽欣，陈官芝，张北川，等.男男性行为者中大学生与同龄非大学生艾滋病高危行为对比研究[J].中华流行病学杂志，2010，31(01):113-114.
[346]王毅，张洪波，徐杰，等.男男性行为者自杀行为及相关影响因素分析[J].实用预防医学，2010，17(05):833-836.
[347]王毅，张洪波，徐杰，等.男男性行为者自我歧视与性行为和心理因素的关系[J].中华预防医学杂志，2010，44(07):636-644.
[348]王复选，汪玲玲.男同性恋艾滋病知识行为和 HIV 感染率调查[J].预防医学情报杂志，2010，26(06):464-465.
[349]刘晓燕，闫红静，唐卫明，等.南京市 2008 年 MSM 人群 HIV 危险因素分析[J].江苏预防医学，2010，21(02):7-11.
[350]陈世海，杨泞珲，朱建靖.南宁市男男性接触者艾滋病相关知识与行为以及性病艾滋病感染情况调查[J].现代预防医学，2010，37(20):3929-3932.
[351]施建春，卢千超，赵涛.南阳市男男性行为人群艾滋病认知及感染情况分析[J].河南预防医学杂志，2010，21(06):405-406.
[352]魏锁，张洪波，徐杰，等.年轻 MSM 性行为及 HIV、梅毒感染分析[J].中国公共卫生，2010，26(12):1479-1480.
[353]金颖，张晶，施京利.齐齐哈尔市男男性行为人群 HIV 及梅毒感染情况现状分析[J].中国预防医学杂志，2010，11(06):629.
[354]周超，丁贤彬，冯连贵，等.青年学生男男性行为者 HIV 感染状况及其影响因素研究[J].中华预防医学杂志，2010，44(05):461-462.
[355]冯连贵，丁贤彬，卢戎戎，等.青年学生中男男性行为人群 HIV、梅毒感染率及估计 HIV 发病率的趋势分析[J].第三军医大学学报，2010，32(24):2644-2646.
[356]龚苍涛，张庆虎.泉州市 252 名男男性行为者艾滋病感染分析[J].中国热带医学，2010，10(12):1496-1497.
[357]马桂林，沈理通，苏成豪，等.厦门市男男性接触人群艾滋病综合干预效果评价[J].中华疾病控制杂志，2010，14(08):726-728.
[358]侯建星，LilyWu，SioTerence，等.上海市部分男男同性恋者社会学特征及艾滋病知识调查[J].上海预防医学，2010(2):83-85.
[359]吴健，范惠俐，朱斌，等.上海市男男性接触者艾滋病梅毒流行率及服务需求研究[J].中国热带医学，2010，10(11):1306-1307.
[360]蔡于茂，洪福昌，冯铁建，等.深圳市 458 例男男性行为者艾滋病知识与性行为调查[J].中国热带医学，2010，10(07):819-820.

[361]石向东, 刘少础, 赵锦, 等.深圳市男男性接触者和男男性工作者的性行为特征及其 HIV、梅毒感染情况的比较分析[J].华南预防医学，2010，36(04):22-25.
[362]蔡文德，陈琳，赵锦，等.深圳市男男性接触者商业性性行为特征及影响因素研究[J].公共卫生与预防医学，2010，21(01):53-55.
[363]刘刚，蔡文德，陈琳，等.深圳市男男性接触者药物滥用流行特征及影响因素研究[J].中华疾病控制杂志，2010，14(11):1063-1065.
[364]刘刚，蔡文德，陈琳，等.深圳市男男性接触者异性性行为特征及影响因素研究[J].华南预防医学，2010，36(02):5-7.
[365]蔡于茂，刘惠，潘鹏，等.深圳市男男性行为者安全套使用影响因素分析[J].中华疾病控制杂志，2010，14(02):115-117.
[366]王路，宋巍，王晓丽，等.沈阳市场所型和网络型男男性行为人群艾滋病知识、行为及感染状况分析[J].中华疾病控制杂志，2010，14(12):1193-1196.
[367]王路，谷渊，王晓丽，等.沈阳市男男性行为人群 HIV 感染率及其影响因素分析[J].中国艾滋病性病，2010，16(04):382-385.
[368]王莹莹，杨海涛，刘晓松，等.石家庄市男男性接触者艾滋病知识、态度、行为及人类免疫缺陷病毒感染状况调查[J].临床荟萃，2010，25(12):1092-1094.
[369]高洁，赵秀萍，羊海涛，等.苏州市男男性行为人群 HIV/STD 感染状况[J].中国艾滋病性病，2010，16(06):601-603.
[370]赵秀萍，曹小平，张福新，等.苏州市男男性行为人群 HIV 感染的配比病例对照研究[J].中国艾滋病性病，2010，16(06):558-561.
[371]赵星，郭鹏飞，李晓松.我国 MSM 人群艾滋病健康教育干预效果的 Meta 分析[J].现代预防医学，2010，37(19):3603-3607.
[372]成国明，方艳姣，何建刚，等.芜湖市男男性行为者艾滋病知识行为状况调查[J].安徽预防医学杂志，2010，16(02):115-117.
[373]李玲.武汉市 456 例男男性行为者艾滋病知识及高危性行为调查[J].中国热带医学, 2010, 10(08):978-979.
[374]丘恣，李新跃，鲍武波，等.武汉市 MSM 人群艾滋病知识知晓率及相关行为调查[J].公共卫生与预防医学，2010，21(01):83-85.
[375]刘普林，姚中兆，石卫东，等.武汉市男男性行为人群 HIV/STD 流行病学调查分析[J].中华疾病控制杂志，2010，14(09):917-919.
[376]李玲.武汉市男男性行为人群艾滋病、梅毒及丙肝感染现状分析[J].中国社会医学杂志，2010，27(04):249-251.
[377]马玉桂，雅西.西藏主要城市男男性行为人群 HIV 感染及高危行为状况调查[J].中国社会医学杂志，2010，27(04):254-255.
[378]张建，李凡，李瑞兰，等.新疆部分城市维吾尔族男男性接触者艾滋病知信行调查[J].新疆医科大学学报，2010，33(04):434-436.
[379]简丹，谢红付，易梅，等.性病门诊男男同性恋者艾滋病知识知晓率和性行为调查[J].中南大学学报(医学版)，2010，35(07):743-748.
[380]许娟，庞琳，吴尊友.性网络与男男性行为人群的 HIV 传播[J].中国艾滋病性病，2010，16(02):198-200.
[381]白玉，蒙进怀.寻找性伴不同场所男男性接触者性行为学特征分析[J].中国公共卫生管理，2010，26(02):202-204.
[382]王志军，孙丽，马小剑.扬州市广陵区男男性行为人群艾滋病感染状况与主要危险行为研究[J].江苏预防医学，2010，21(02):4-7.

[383]张军，王志军，姜辉，等.扬州市男男性接触者艾滋病感染危险因素分析[J].现代预防医学，2010，37(01):4-6.
[384]周加华，吴丽芸，莫尚鹏，等.宜春市男男性行为人群艾滋病相关知识及行为调查[J].预防医学论坛，2010，16(12):1175-1176.
[385]王硕，福燕，马小燕，等.已知和未知自身感染状况的男男性行为人群 HIV 阳性者性行为特征分析[J].中华流行病学杂志，2010，31(06):716-717.
[386]李晶，李渊，雷利民，等.银川市男男性行为者艾滋病知识水平与高危性行为调查[J].医学动物防制，2010，26(11):1022-1023.
[387]李洋，张北川，李秀芳，等.有导致出血的性施虐-受虐行为的男男性行为者行为特征研究[J].中华流行病学杂志，2010，31(02):142-145.
[388]裘丹红，沈伟伟，蒋琼俏，等.浙江省台州地区男男性行为人群 HIV/STIs 感染状况调查[J].中国卫生检验杂志，2010，20(08):2044-2046.
[389]郑迎军，夏俊瑞，许娟，等.中国大陆 7 城市 MSM 人群的高危性行为与生活质量的关系[J].中国艾滋病性病，2010，16(06):554-557.
[390]夏俊瑞，郑迎军，张洪波，等.中国大陆 7 城市男男性行为者人群生存质量状况及其影响因素[J].现代预防医学，2010，37(20):3812-3815.
[391]佘颖，钟晓妮，张燕，等.中国西部地区男男性行为人群艾滋病相关知识及行为调查分析[J].重庆医科大学学报，2010，35(12):1902-1905.
[392]汪涛，来学惠，李雷，等.中山市男男性行为人群艾滋病/性传播疾病危险行为及感染率现况调查[J].实用预防医学，2010，17(07):1261-1263.
[393]丁贤彬，冯连贵，徐静，等.重庆市 743 名男男性行为者 HIV、梅毒、HCV、HSV-II感染率及其影响因素研究[J].中华疾病控制杂志，2010，14(03):227-231.
[394]丁贤彬，冯连贵，陈英，等.重庆市某远郊区男男性行为人群人类免疫缺陷病毒感染率及影响因素[J].中华预防医学杂志，2010，44(03):273-275.
[395]丁贤彬，冯连贵，萧燕，等.重庆市男男性行为人群干预效果评估[J].热带医学杂志，2010，10(03):323-326.
[396]周超，丁贤彬，冯连贵，等.重庆市男男性行为者艾滋病高危行为调查[J].中国公共卫生，2010，26(12):1482-1483.
[397]冯连贵，丁贤彬，徐静，等.重庆市网络型与非网络型男男性行为人群 HIV、梅毒、丙肝感染率及其相关行为特征比较研究[J].热带医学杂志，2010，10(01):78-82.
[398]曾缓，丁贤彬，徐佳薇，等.重庆市主城区男男同性恋人群艾滋病防治工作现状及需求分析[J].第二军医大学学报，2011，32(05):494-499.
[399]陈正义.遵义市 109 例男男性行为人群艾滋病监测分析[J].医学动物防制，2011，27(11):991-993.
[400]张严，蔺秀云，福燕."告知还是不告知":我国男同性恋 HIV 阳性者暴露现状和原因的质性研究[J].中国临床心理学杂志，2011，19(05):628-632.
[401]周超，丁贤彬，冯连贵，等.1166 例男男性行为者 HIV、梅毒感染状况及其影响因素研究[J].现代预防医学，2011，38(05):815-816.
[402]何华先，张大迁，周磊，等.135 例 MSM 人群行为学特征与行为干预实践[J].实用预防医学，2011，18(06):1151-1153.
[403]陈树昶，罗艳，程洁，等.2006-2008 年杭州市男男性行为人群 HIV 与性行为监测结果分析[J].中国艾滋病性病，2011，17(01):38-39.
[404]卢戎戎，易东，丁贤彬，等.2006-2009 年重庆市男男性行为人群 HIV 感染者情况分析[J].第三军医大学学报，2011，33(14):1534-1537.

[405]丁贤彬，冯连贵，卢戎戎，等.2006-2009 年重庆市男男性行为人群性行为特征及 HIV 和梅毒感染率分析[J].热带医学杂志，2011，11(06):689-692.
[406]陈强，李洋，孙燕鸣，等.2008-2009 年北京市男男性行为者 HIV-1 感染率与新发感染率调查[J].中国医药生物技术，2011，6(04):270-273.
[407]梁自勉，陈抒豪，宫艳华.2008-2010 年佛山市男男性接触人群艾滋病流行状况与防控对策[J].医学动物防制，2011，27(05):419-421.
[408]朱艳文，王美花，杨慧，等.2008 年济南市部分男男性行为人群艾滋病相关知识、性行为特征及 HIV 和梅毒感染状况调查[J].预防医学论坛，2011，17(2):142-144.
[409]王复选，黄映龙.2009～2010 年鹰潭市男男性行为人群艾滋病干预效果分析[J].预防医学论坛，2011，17(06):518-519.
[410]王复选，黄映龙.2009～2010 年鹰潭市男男性行为人群艾滋病干预效果分析[J].预防医学论坛，2011，17(06):518-519.
[411]刘英杰，姜树林，胡尧，等.2009 年北京市男男性行为者性行为特征和艾滋病等性传播疾病感染状况[J].中华预防医学杂志，2011，45(11):971-974.
[412]戴丽萍，姜宜海，刘奇，等.2009 年广州市越秀区 1596 名男男性接触者艾滋病相关危险行为及其影响因素分析[J].疾病监测，2011，26(12):972-975.
[413]王毅，徐杰，李志军，等.2009 年绵阳市男男性行为者艾滋病相关知识知晓情况及影响因素调查[J].预防医学论坛，2011，17(10):886-888.
[414]刘晓勇，许建，刘智勇，等.2010 年济宁市男男性行为人群艾滋病/梅毒/丙肝感染情况及艾滋病相关知识行为调查[J].预防医学论坛，2011，17(09):786-788.
[415]陈志娟，陈晓明，黄丽花，等.2010 年云南省大理市男男性行为人群哨点情况分析[J].卫生软科学，2011，25(10):719-720.
[416]周彩霞，潘志萍，陈正义，等.2010 年遵义市艾滋病哨点监测结果分析[J].江苏预防医学，2011，22(03):11-13.
[417]吴国富，何飞鸣，衡时雨.244 名高校男男性行为学生艾滋病知识和高危行为干预效果评价[J].安徽预防医学杂志，2011，17(02):86-88.
[418]黄文军，肖云，杨曦，等.260 名九江市男男性行为者 HIV/STD 相关知识行为及感染状况调查[J].实用预防医学，2011，18(12):2275-2277.
[419]牟李红，黄爱龙，彭鸿斌，等.309 例男同性恋者艾滋病相关知识、态度、行为的现况调查[J].中华男科学杂志，2011，17(05):401-405.
[420]曲琳，高永明，刘静，等.805 例男男性行为者 HIV 感染及其影响因素分析[J].实用预防医学，2011，18(11):2081-2083.
[421]陈辉，罗伟，李媛，等.831 名男男性行为者 HIV 和梅毒感染状况分析[J].皮肤病与性病，2011，33(04):236-237.
[422]丁贤彬，冯连贵，卢戎戎，等.HIV 检测对 MSM 人群艾滋病感染风险的影响[J].热带医学杂志，2011，11(11):1294-1297.
[423]李剑，徐丹戈，倪志敏，等.HIV 现场快速检测在浴池男男性接触人群中的应用[J].浙江预防医学，2011，23(09):6-9.
[424]魏爱旺，张俊岭，马智龙.MSM 人群艾滋病、梅毒及丙型肝炎感染检测结果分析[J].医学动物防制，2011，27(08):727-728.
[425]王文明，薛黎坚，陈彦卿.MSM 人群感染性病/艾滋病的脆弱性构成及预防控制研究[J].中国热带医学，2011，11(03):296-298.
[426]宇方，张洪波，王君，等.MSM 同伴群与 HIV 感染关系[J].现代预防医学，2011，38(05):810-814.

[427]汪方华，程晓莉，肖永康，等.安徽省 2009-2010 年男男性行为者艾滋病哨点监测分析[J].中华疾病控制杂志，2011，15(08):685-688.
[428]计国平，徐杰.安徽省男男性行为人群艾滋病防治服务利用情况分析[J].安徽预防医学杂志，2011，17(01):1-3.
[429]计国平，徐杰，李晓静，等.安徽省男男性行为人群艾滋病知识知晓率及其相关因素分析[J].中华疾病控制杂志，2011，15(07):568-570.
[430]吴焱，闫会文，屈文妍，等.北京地区 2001-2010 年 MSM 人群 HIV 感染状况及综合干预模式的探讨[J].中国艾滋病性病，2011，17(04):407-409.
[431]王万海，马燕，姜树林，等.北京男男性接触 HIV 感染者合并病毒性肝炎分析[J].中国公共卫生，2011，27(05):624-625.
[432]于明润，李书明，闫莉，等.北京市 MSM 人群 HIV 抗体检测及影响因素分析[J].中国公共卫生，2011，27(10):1234-1236.
[433]郑建东，庞琳，徐杰，等.北京市大学生男男性行为人群 HIV 与梅毒感染相关流行病学调查[J].中国艾滋病性病，2011，17(03):358-359.
[434]郑建东，庞琳，徐杰，等.北京市大学生男男性行为者 HIV 相关知信行定性调查[J].中国健康教育，2011，27(03):174-176.
[435]郑建东，庞琳，徐杰，等.北京市大学生男男性行为者同性性行为特征分析[J].疾病监测，2011，26(06):450-453.
[436]郑建东，庞琳，徐杰，等.北京市大学在校生男男性行为者 HIV 感染状况及其相关危险性行为调查[J].中华流行病学杂志，2011，32(04):337-340.
[437]陈晓霞，于建平，李民，等.北京市男男性接触者人类免疫缺陷病毒及梅毒感染调查[J].首都公共卫生，2011，5(04):153-156.
[438]李书明，周振海，姜树林，等.北京市男男性行为者 HIV 和梅毒螺旋体血清抗体阳转率及影响因素[J].中华预防医学杂志，2011，45(02):118-122.
[439]范颂，孙燕鸣，卢红艳，等.北京市男男性行为者安全套使用社会规范与无保护性肛交关系的研究[J].中华流行病学杂志，2011，32(05):473-476.
[440]林桂芹，陈英，陈晨，等.本溪市男男性行为者艾滋病知信行现状调查[J].职业与健康，2011，27(05):532-534.
[441]陶桃，曹越，殷维，等.不同活动场所 MSM 人群艾滋病知识行为比较研究[J].公共卫生与预防医学，2011，22(06):5-8.
[442]杨跃进，袁巧菊，韩丹.不同生育态度的 MSM 艾滋病患者相关知识与行为特点调查分析[J].医药论坛杂志，2011，32(02):130-131.
[443]李建石，吴志辉.郴州市 MSM 人群预防艾滋病干预模式探讨[J].实用预防医学，2011，18(09):1791-1792.
[444]朱厚宏，刘杨，张祁，等.成都市男男性行为人群艾滋病及其关联检测结果的分析[J].现代预防医学，2011，38(07):1319-1321.
[445]陈怀良，陈磊，田晋，等.成都市男男性行为人群性伴数量影响因素分析[J].预防医学情报杂志，2011，27(12):978-981.
[446]何国平，刘立珍，李现红，等.成都市男男性行为者艾滋病相关羞辱和歧视的现状研究[J].中国全科医学，2011(13):1475-1478.
[447]代泽伟，裴晓方，何杨，等.德阳市 MSM 人群 HIV、HCV 和梅毒感染现状及性行为特征研究[J].现代预防医学，2011，38(17):3544-3547.
[448]宫艳华，陈抒豪，梁自勉，等.佛山市男男性接触者 HIV 感染状况和高危行为调查[J].华南预防医学，2011，37(01):31-33.

[449]陈舸，潘蕴蛟，林丽，等.福建省某市男男性行为者 AIDS 相关危险行为及感染状况调查[J].中华预防医学杂志，2011，45(01):86-88.
[450]潘蕴蛟，陈舸，郑健，等.福建省三城市男男性行为人群艾滋病相关影响因素调查[J].中国热带医学，2011，11(06):700-701.
[451]王永香，康殿民，陶小润，等.高档酒吧男男性行为者艾滋病知识知晓及安全套使用情况调查[J].预防医学论坛，2011，17(08):683-685.
[452]王希，庾泳，肖水源，等.高校男同性恋 QQ 群用户异性婚姻意愿及相关因素[J].中国心理卫生杂志，2011，25(02):122-127.
[453]蓝光华，刘伟，黄玉满，等.广西部分男男性行为者包皮环切术接受意愿分析[J].广西医科大学学报，2011，28(04):503-505.
[454]韦所苏，邹云锋，徐永芳，等.广西地区 650 名男男性行为者对暴露前预防 HIV 感染接受意愿及其影响因素研究[J].中华流行病学杂志，2011，32(08):786-788.
[455]蓝光华，刘伟，朱秋映，等.广西男男性行为人群梅毒感染危险因素分析[J].现代预防医学，2011，38(22):4586-4588.
[456]戴丽萍，姜宜海，龚诚华，等.广州市 1315 名男男性行为人群的行为特征及艾滋病流行状况[J].中国艾滋病性病，2011，17(01):35-37.
[457]吴杰，卢次勇，郝元涛，等.广州市男男性接触人群社会心理状况调查[J].中国公共卫生，2011，27(04):476-478.
[458]郑敏，姚永明，申莉梅，等.贵阳市社区男男性行为者艾滋病感染状况调查[J].贵州医药，2011，35(09):830-831.
[459]杨宏远，胡绍源，袁飞.贵州省男男性接触人群艾滋病传播现状调查[J].中国预防医学杂志，2011，12(01):106-107.
[460]何滨，冯福，张书芳，等.海口市男男性行为人群 HIV/梅毒感染状况分析[J].中国公共卫生，2011，27(04):480-482.
[461]张睿，帅慧群，商晓春.杭州市男男同性恋人群 2006 年-2009 年艾滋病、性病感染状况研究[J].中国卫生检验杂志，2011，21(03):732-733.
[462]黄泓滟，张小鹏，李钰，等.合肥市男男性行为人群 HIV、梅毒、丙肝感染状况及性行为特征研究[J].现代预防医学，2011，38(10):1933-1935.
[463]程绍辉，朱效科，聂晓璞，等.加强与非政府组织合作促进 MSM 社区 HIV 检测与随访效果评估[J].中华疾病控制杂志，2011，15(03):207-209.
[464]还锡萍，尹跃平，傅更锋，等.江苏省男男性行为人群性传播疾病感染状况及危险因素[J].中华预防医学杂志，2011，45(11):975-978.
[465]曹宁校，张国成，闫红静，等.江苏苏南 MSM 集中活动的娱乐场所人群状况调查[J].中国性科学，2011，20(05):52-55.
[466]李建军，还锡萍，闫红静，等.结合自愿咨询检测进行男男性接触人群高危行为干预的效果评价[J].中国预防医学杂志，2011，12(08):666-669.
[467]王文明，薛黎坚，陈彦卿.昆山市男男性行为人群 HIV/梅毒感染及艾滋病知识、态度及行为调查[J].预防医学论坛，2011，17(05):396-398.
[468]陈继军，张晓宇，陈晓燕，等.兰州市男男性接触人群 HIV 流行影响因素分析[J].中国皮肤性病学杂志，2011，25(12):954-957.
[469]陈继军，刘军，李莹，等.兰州市男男性行为人群艾滋病流行趋势及影响因素分析[J].中国艾滋病性病，2011，17(03):328-331.
[470]宋爱华，宋爱军，苏欣，等.廊坊市 MSM 人群艾滋病性病感染危险因素及感染状况调查[J].医学动物防制，2011，27(10):924-926.

[471]金玫华，杨中荣，董正全.利用互联网从事男男性行为者艾滋病流行病学特征与干预对策[J].疾病监测，2011，26(07):575-578.
[472]楚振兴，马宁，徐俊杰，等.辽宁省 MSMHIV 感染现状及其影响因素分析[J].中国公共卫生，2011，27(08):967-969.
[473]槐鹏程，姜延峰，杨霞，等.辽宁省鞍山市艾滋病自愿咨询检测需求状况分析[J].疾病监测，2011，26(06):463-466.
[474]蔡高峰，马瞧勤，陈树昶，等.男男性接触人群艾滋病干预效果分析与探讨[J].中国预防医学杂志，2011，12(09):788-792.
[475]迟富利，杨景元，曲琳，等.男男性接触人群的特征及其艾滋病防治研究进展[J].内蒙古医学杂志，2011，43(06):680-684.
[476]李玲，胡春华，张春霞，等.男男性接触性病患者健康教育的实施[J].护理学杂志，2011，26(09):71-73.
[477]陈继军，王艳艳，李莹，等.男男性接触者艾滋病和梅毒感染及性行为调查[J].中国公共卫生，2011，27(12):1604.
[478]陈章颖，王鸣，徐慧芳，等.男男性接触者互联网行为特征及接受干预的意向分析[J].华南预防医学，2011，37(03):23-26.
[479]刘立珍，李现红，王红红，等.男男性行为人群艾滋病防治及对策研究现状[J].护理学杂志，2011，26(08):94-97.
[480]金慧心，王艳，王卫宪，等.男男性行为人群艾滋病及其他性传播疾病的流行概况[J].中国艾滋病性病，2011，17(04):458-459.
[481]张莹，时景璞，施建春，等.男男性行为人群艾滋病相关知识与性行为调查[J].中国医科大学学报，2011，40(12):1110-1113.
[482]刘昆仑，高燕，葛利荣.男男性行为人群安全套使用需求调查[J].中国艾滋病性病，2011，17(05):572-573.
[483]薛黎坚，王文明，徐勇，等.男男性行为人群的危险行为与社会压力及其影响因素的网络调查[J].中国艾滋病性病，2011，17(03):324-327.
[484]王婉薇，余世林，陈凯，等.男男性行为人群核心同伴教育艾滋病综合干预效果分析[J].预防医学情报杂志，2011，27(08):590-593.
[485]谭京广，陈虹，程锦泉，等.男男性行为人群危险行为干预效果分析[J].中国热带医学，2011，11(12):1469-1471.
[486]李春梅，贾玉江，刘建波，等.男男性行为人群无保护肛交影响因素分析[J].中国公共卫生，2011，27(03):351-352.
[487]赵洪鑫，赵瑞，武俊青.男男性行为人群性健康网络调查和网络干预进展[J].中国计划生育和妇产科，2011，3(05):75-78.
[488]周月芳，福燕，高美霞，等.男男性行为人群中 HIV 感染者的心理状况分析及其护理[J].北京医学，2011，33(06):514-515.
[489]李秀芳，张北川，汪宁，等.男男性行为性病患者性行为特征分析[J].中国麻风皮肤病杂志，2011，27(01):19-22.
[490]王毅，徐杰，李志军，等.男男性行为者艾滋病防治知识知晓及与人口学特征的关系[J].实用预防医学，2011，18(10):1854-1857.
[491]王毅，徐杰，李志军，等.男男性行为者艾滋病知识知晓与社会网络和预防服务的关系[J].预防医学情报杂志，2011，27(11):852-856.
[492]于海荣，王束玫，杜桂英，等.男男性行为者人口学因素与艾滋病知识、行为及认同态度关联性研究[J].医学动物防制，2011，27(10):907-908.

[493]王毅，张洪波，徐杰，等.男男性行为者社会歧视经历调查[J].中国公共卫生，2011，27(01):91-92.
[494]王毅，徐杰，李志军，等.男男性行为者无保护性行为性伴相关行为特征调查[J].实用预防医学，2011，18(11):2072-2076.
[495]王毅，张洪波，徐杰，等.男男性行为者自杀行为相关危险因素 Logistic 分析[J].现代预防医学，2011，38(12):2220-2223.
[496]王毅，张洪波，徐杰，等.男男性行为者自杀行为与社会心理因素关系[J].实用预防医学，2011，18(02):232-235.
[497]刁青华，李琦，阮世颖，等.南昌市高校学生男男同性恋性病/艾滋病知信行调查[J].南昌大学学报(医学版)，2011，51(06):78-81.
[498]路亮，徐丹，邱红恒，等.南昌市男男性接触者性病艾滋病感染状况调查[J].公共卫生与预防医学，2011，22(01):45-46.
[499]张芹，黄毅，吴涛，等.南充市男男性接触者艾滋病知识及高危性行为调查[J].现代预防医学，2011，38(01):89-91.
[500]汪娜，张敏，吴苏姝，等.南京市男男性接触人群艾滋病相关知识态度及行为调查[J].现代预防医学，2011，38(01):117-118.
[501]陈旭，傅更锋，徐晓琴，等.南京市男男性行为人群淋球菌和沙眼衣原体感染状况研究[J].安徽医科大学学报，2011，46(06):569-572.
[502]张敏，闫红静，汪娜，等.南京市男男性行为人群随访中队列保持研究[J].中华疾病控制杂志，2011，15(10):839-842.
[503]曲琳，涛波，戴纪强，等.内蒙古自治区部分城市男男性接触者艾滋病综合干预效果评价[J].中华传染病杂志，2011，29(01):30-33.
[504]王飞，刘松涛，任长庆.农村地区男男性行为人群艾滋病相关知识行为定性研究[J].重庆医学，2011，40(31):3223-3224.
[505]张臬，毛军，李朋，等.浦东新区男男性行为人群艾滋病相关的健康与求医需求分析[J].环境与职业医学，2011，28(12):781-784,788.
[506]张重辉，王素萍.山西省某市 300 例男男性接触者艾滋病感染现状及性行为特征调查[J].医学动物防制，2011，27(03):246-247.
[507]李十月，曹越.社会网络理论及其在男男性行为人群艾滋病干预中的应用[J].公共卫生与预防医学，2011，22(05):1-4.
[508]刘少础，陈琳，蔡文德，等.深圳市 MSM 艾滋病知识与预防艾滋病服务调查[J].中国艾滋病性病，2011，17(01):52-55.
[509]赵锦，蔡文德，陈珠蝶，等.深圳市男男性行为人群 HIV 梅毒及 HCV 新发感染的队列研究[J].中国艾滋病性病，2011，17(05):530-532.
[510]谢妮，谭京广，孙振球.深圳市男男性行为人群相关高危行为干预及其影响因素研究[J].中华流行病学杂志，2011，32(07):669-671.
[511]刘少础，陈琳，蔡文德，等.深圳市男男性行为者 HIV 和梅毒感染及行为调查[J].中国公共卫生，2011，27(07):908-910.
[512]王莹莹，杨海涛，李生奎，等.石家庄市 2008 年 450 例男男性接触者艾滋病和梅毒感染状况及艾滋病知识、态度、行为调查[J].现代预防医学，2011，38(19):3958-3960.
[513]周红，赵川，刘晓松，等.石家庄市 400 名 MSM 人群艾滋病监测情况分析[J].医学动物防制，2011，27(11):1007-1009.
[514]张福新，赵秀萍，曹小平，等.苏州市男男性行为人群 HIV 感染及相关因素研究[J].中华疾病控制杂志，2011，15(12):1031-1034.

[515]宁铁林，郭燕，柳忠泉，等.天津市 2008—2009 年男男性行为者 HIV 新近感染状况调查[J].中华流行病学杂志，2011，32(11):1087-1090.
[516]杨峘，张亚林.同性恋者精神健康相关问题[J].现代预防医学，2011，38(08):1482-1484.
[517]王崇金，姜宝法.我国 MSM 人群艾滋病干预效果的系统评价[J].中国卫生事业管理，2011，28(02):154-157.
[518]沈晓沛，庞琳，徐杰.我国男男性行为人群艾滋病高危行为干预方法的进展[J].中国艾滋病性病，2011，17(03):386-388.
[519]佘敏，张洪波，王君，等.我国四城市 MSM 人群 HIV 感染者性伴类型和性行为特征[J].中华疾病控制杂志，2011，15(11):952-955.
[520]王新迪，马燕，杨阳.乌鲁木齐市男男性行为人群 HIV、梅毒和 HCV 感染状况分析[J].皮肤病与性病，2011，33(02):104-105.
[521]李春芳，曹越，燕虹，等.武汉地区男男性接触者获得艾滋病知识及干预服务状况调查[J].公共卫生与预防医学，2011，22(01):67-68.
[522]赵长成，陶宜新，高晓宇，等.西宁市男男性行为人群艾滋病感染情况调查[J].中国艾滋病性病，2011，17(02):142-144.
[523]田志伟，赵淑娟，牛留业，等.许昌市男男性接触者艾滋病和梅毒感染状况调查[J].职业与健康，2011，27(23):2737-2739.
[524]何勤英，王晓冬，于飞，等.学生男男性行为人群行为特征及 HIV 感染危险因素[J].预防医学情报杂志，2011，27(11):890-893.
[525]陈宏泉，李洋，张北川，等.有自杀意念的男同性爱者/双性爱者艾滋病高危行为研究[J].中华流行病学杂志，2011，32(10):983-986.
[526]袁红梅，成磊，张敏伟.雨山区男男性行为人群艾滋病知识行为调查[J].安徽预防医学杂志，2011，17(03):239-240.
[527]李连学，马懿，高良敏，等.玉溪市男男性接触者安全套使用及影响因素分析[J].预防医学情报杂志，2011，27(10):800-804.
[528]杨桂芝，王洪，施吉昌.云南大理州男男同性恋者 HI/VTP 感染状况调查[J].中国卫生检验杂志，2011，21(04):995-996.
[529]冯英，刘春蓓，戴继坤，等.云南省曲靖市 MSM 人群艾滋病知识知晓状况调查分析[J].中国艾滋病性病，2011，17(06):667-669.
[530]冯英，戴继坤，刘春蓓，等.云南省曲靖市存在两性性伴的男男性行为者艾滋病高危行为调查分析[J].重庆医学，2011，40(30):3081-3083.
[531]冯英，刘春蓓，戴继坤，等.云南省曲靖市男男性行为者艾滋病相关同性高危行为调查分析[J].中国社会医学杂志，2011，28(06):398-400.
[532]王锦瑜，汪涛，王曼，等.中山市男男性行为人群的艾滋病感染状况及影响因素分析[J].华南预防医学，2011，37(03):27-31.
[533]李雪峰，丁贤彬，卢戎戎，等.重庆市男男性行为人群近 1 年 HIV 检测情况及影响因素[J].中国艾滋病性病，2011，17(06):651-653.
[534]冯连贵，丁贤彬，卢戎戎，等.重庆市男男性行为者 HIV 抗体阳转率及其影响因素研究[J].中华疾病控制杂志，2011，15(07):564-567.
[535]郭晓嘉，丁贤彬，冯连贵，等.重庆市学生 MSM 人群中 HIV 和梅毒的流行状况[J].中国艾滋病性病，2011，17(02):178.
[536]朱克京，杜曼，张静涛，等.珠海市男男同性性接触者 HIV 感染现状及危险行为分析[J].实用预防医学，2012，19(08):1261-1263.
[537]郭黄吉，冯丹，易兰，等.遵义市男男性行为人群 HIV、梅毒、HCV 感染状况及其行为

学特征[J].职业与健康，2012，28(04):440-441.
[538]闫玉香, 张延吉, 李淑芳.120 例男男性接触者行为学特点分析[J].首都公共卫生, 2012, 6(04):164-167.
[539]李纲, 韩威.126 例 MSMHIV/STD 感染状况及高危行为调查[J].中国艾滋病性病, 2012, 18(11):749-750.
[540]靳子义，李建军，张倩倩，等.2001-2009 年中国男男性行为人群行为学特征[J].中国艾滋病性病，2012，18(07):466-470.
[541]胡海洋，徐晓琴，傅更锋，等.2008～2009 年江苏省 3 城市男男性行为人群 HIV-1 新发感染率调查[J].南京医科大学学报(自然科学版)，2012，32(04):487-490.
[542]梅林，赵启玉，韩红，等.2008～2009 年太原市 MSM 人群艾滋病行为及血清学调查[J].预防医学论坛，2012，18(01):7-9.
[543]梅林，赵启玉，韩红，等.2008～2009 年太原市 MSM 人群艾滋病行为及血清学调查[J].预防医学论坛，2012，18(01):7-9.
[544]孙茂利，孔颖，姜杰，等.2008～2012 年大连市男男性接触者行为情况和 HIV、HCV、梅毒、HBV 感染状况调查[J].预防医学论坛，2012，18(12):907-908.
[545]周硕，赵西和，何静.2010 年绵阳市男男性行为人群艾滋病哨点监测[J].预防医学情报杂志，2012，28(03):171-174.
[546]韩红，梅林，韩婷，等.2010 年太原市男男性行为人群艾滋病相关知识、行为及 HIV 感染情况调查[J].预防医学论坛，2012，18(07):497-498.
[547]张明辉，刘晓霞，陈鑫，等.2010 年镇江市男男性行为者队列基线调查[J].职业与健康，2012，28(18):2197-2201.
[548]汤杰，张振开，蒋巍，等.2011 年桂林市男男性行为人群艾滋病相关知识、行为及 HIV/梅毒感染情况调查[J].预防医学论坛，2012，18(06):431-432.
[549]马彦民，孙定勇，王哲.2011 年河南省男男性行为人群 HIV 与梅毒感染状况[J].中华预防医学杂志，2012，46(11):1034-1035.
[550]董蕾，韩磊，张晓菲.2011 年青岛市男男性行为人群艾滋病相关知识、行为及 HIV/梅毒感染状况调查[J].预防医学论坛，2012，18(08):586-588.
[551]冯燎，杨一挥，袁丹，等.2011 年四川省 MSM 人群 HIV 感染与危险行为监测结果[J].中国艾滋病性病，2012，18(12):830-832.
[552]李林，车敏.2011 年武汉市汉阳区男男同性恋人群艾滋病相关知识、性行为特征及艾滋病病毒感染状况调查[J].预防医学论坛，2012，18(4):266-267,271.
[553]张东江，谢奎平，杨爱学.2011 年新疆奎屯市及周边地区男男性行为人群艾滋病知识知晓情况调查[J].疾病预防控制通报，2012，27(03):8-9.
[554]全信斌，林瑞玲，刘俊杰，等.2011 年玉林市 MSM 人群 AIDS 相关知识、性行为特征及 HIV/梅毒感染状况调查[J].预防医学论坛，2012，18(08):571-573.
[555]韩红，梅林，车晓文，等.2012 年山西省太原市男男性行为人群艾滋病哨点监测分析[J].疾病监测，2012，27(12):964-966.
[556]李辉，王茜茜，李秀芳，等.2250 例男男性接触者安全套使用与性传播疾病的关系[J].中华皮肤科杂志，2012，45(05):312-314.
[557]刘福宝.450 名 MSM 人群 HIV 监测情况分析[J].中国美容医学，2012，21(18):254-255.
[558]濮志清, 张敏, 陈海燕.734 例男男性行为者行为特征调查[J].中国公共卫生管理, 2012, 28(05):625-626.
[559]蔡高峰，马瞧勤，潘晓红，等.HIV 阴性 MSM 人群艾滋病相关行为特征及其干预效果[J].浙江预防医学，2012，24(12):1-3.

[560]戴丽萍，姜宜海，龚诚华，等.MSM 艾滋病相关知识行为及互联网的使用情况调查[J].中国健康教育，2012，28(12):1026-1028.
[561]黄青兰，董利民，姚菲菲，等.MSM 人群 HIV 感染状况及高危行为影响因素分析[J].中国现代医药杂志，2012，14(06):7-10.
[562]钱跃升，陶小润，郝连正，等.MSM 人群艾滋病感染状况及影响因素研究[J].中国艾滋病性病，2012，18(11):738-740.
[563]马功燕，李开春，陈海燕，等.MSM 人群艾滋病相关高危行为及影响因素分析[J].安徽预防医学杂志，2012，18(05):332-335.
[564]程伟彬，钟斐，文芳，等.艾滋病相关知识、态度对男男性行为人群高危性行为的影响[J].中华疾病控制杂志，2012，16(12):1064-1067.
[565]赵丽，赵文彬，郑冰，等.鞍山市 MSM 艾滋病相关知识行为及卫生需求调查[J].中国公共卫生，2012，28(09):1230-1231.
[566]王珏，徐杰，斗智，等.八城市男男性行为人群不同性角色高危行为及 HIV 梅毒生殖器疱疹感染状况分析[J].中国艾滋病性病，2012，18(05):306-309.
[567]高彦杰，于明润，李书明，等.北京市 MSMHIV、梅毒和 HSV-II感染及影响因素分析[J].中国公共卫生，2012，28(04):451-453.
[568]孙晓云，张向军，李敏，等.北京市男男性接触者 HIV 相关行为和 STD 感染状况[J].职业与健康，2012，28(05):574-575.
[569]李东亮，高彦杰，于明润，等.北京市男男性行为者 HIV 新发感染率及影响因素研究[J].中华流行病学杂志，2012，33(07):663-666.
[570]刘英杰，王小芳，宋亮，等.北京市女女性行为人群性行为特征与性传播疾病感染状况[J].中华预防医学杂志，2012，46(07):627-630.
[571]陈鑫，朱莹莹，还锡萍，等.不同城市男男性行为者艾滋病流行特征比较[J].中国公共卫生，2012，28(12):1549-1552.
[572]甄森，王金塔，周建波，等.不同活动场所男男性行为人群高危行为特征分析[J].中国公共卫生，2012，28(10):1307-1310.
[573]董正全，金玫华，杨中荣.不同文化程度对男男性行为人群艾滋病知识知晓率的影响[J].中国艾滋病性病，2012，18(06):405-407.
[574]周艳秋，高洁，顾凯侃，等.部分男男性行为者 HIV/梅毒感染状况及影响因素分析[J].中国初级卫生保健，2012，26(11):72-74.
[575]王金塔，郭燕丽，周建波，等.常州市男男性行为者人口流动与艾滋病高危行为关系的研究[J].卫生研究，2012，41(05):782-784.
[576]胥建中，温贤敏，陈敏，等.成都男男性行为人群中艾滋病患者合并其他传染病的调查分析[J].华西医学，2012，27(08):1140-1142.
[577]田晋，陈磊，刘婷，等.成都市男男性行为人群 HIV 检测影响因素分析[J].预防医学情报杂志，2012，28(08):605-608.
[578]唐梅.达州市男男性行为者性行为及相关疾病感染现状分析[J].职业卫生与病伤，2012，27(05):314-315.
[579]陈晓明，黄丽花，陈志娟，等.大理州男男性行为人群艾滋病病毒抗体阴性队列调查[J].中国艾滋病性病，2012，18(06):402-404.
[580]孙茂利，孔颖，姜杰.大连市男男性接触者有关情况及 HIV、HCV、梅毒、HBV 感染状况调查[J].疾病监测与控制，2012，6(12):708-709.
[581]赵志杰，李瑞，佟伟，等.大连市男男性行为人群艾滋病危险行为调查[J].中国公共卫生，2012，28(02):218-219.

[582]陈柏芬，钟洁莹，张巧利，等.东莞市男男性行为者艾滋病知识行为调查[J].热带医学杂志，2012，12(01):108-111.
[583]林勋，张明雅，陈亮，等.福建省男男性行为者艾滋病相关知识态度行为调查[J].海峡预防医学杂志，2012，18(05):24-26.
[584]杨鲁光，宋秀萍，丁心平，等.阜阳市男男性行为者艾滋病知识、行为以及 HIV 感染状况研究[J].安徽预防医学杂志，2012，18(01):22-23.
[585]刘珺，郜艳晖，梁自勉，等.广东省佛山市男男性行为者抑郁状况、性行为特征及关联研究[J].中华流行病学杂志，2012，33(05):483-487.
[586]陈伟，周芸，蒋巍，等.广西桂林市 2010 年男男性行为者艾滋病监测分析[J].中国热带医学，2012，12(01):58-60.
[587]蓝光华，刘伟，朱秋映，等.广西男男性行为人群艾滋病与性病监测结果分析[J].应用预防医学，2012，18(04):238-240.
[588]宋丹丹，张洪波，王君，等.广州和成都市男男性行为兼异性性行为者的 HIV 感染状况及性行为特征[J].中华流行病学杂志，2012，33(04):368-373.
[589]牟成惠，王强，钟逸雯，等.广州市 2010 年男男性接触者艾滋病检测相关情况分析[J].南方医科大学学报，2012，32(01):113-115.
[590]陈蔼端，钟斐，樊莉蕊，等.广州市男男性行为者无保护肛交情况及相关因素分析[J].华南预防医学，2012，38(04):1-6.
[591]吴冬华，陈佳钏.广州同性恋大学生的自我认知与公众态度调查[J].山东青年政治学院学报，2012，28(05):70-75.
[592]郑敏，刘民，姚永明，等.贵阳市男男性行为者包皮环切术接受意愿调查[J].中国公共卫生，2012，28(07):918-920.
[593]崔巍，张炎，刘洪昌，等.邯郸市男男性行为者 HIV 感染状况及影响因素研究[J].医学动物防制，2012，28(12):1352-1355.
[594]金丹丹，陈树昶，罗艳，等.杭州市男男性接触人群艾滋病病毒感染现状及感染者特征分析[J].中国预防医学杂志，2012，13(07):533-535.
[595]陈玉萍，苗香芬，张玉琪，等.河北省男男性接触人群梅毒防治知识知晓率调查[J].河北医药，2012，34(17):2676-2677.
[596]闫世春，赵亚双，李一，等.黑龙江省男男性行为者艾滋病哨点监测结果分析[J].中国公共卫生管理，2012，28(03):300-301.
[597]杨芳，王备，姚璇，等.湖北省男男性行为人群艾滋病高危行为影响因素研究[J].医学与社会，2012，25(04):1-3.
[598]王备，杨芳，姚璇，等.湖北省男男性行为人群艾滋病流行状况及影响因素调查[J].中国艾滋病性病，2012，18(04):245-247.
[599]谢爱齐，齐星，崔德勇，等.湖北襄阳市 2009 ~ 2011 年男男性接触者艾滋病哨点监测[J].中国热带医学，2012，12(04):480-481.
[600]杨中荣，董正全，金玫华，等.湖州市 MSM 人群安全套使用的影响因素研究[J].浙江预防医学，2012，24(12):4-6.
[601]李婧，陈祥生.互联网在男男性行为人群性病艾滋病相关危险行为监测和干预研究中的应用[J].中国艾滋病性病，2012，18(01):62-64.
[602]李青青，陈颖，韩德琳，等.基于扎根理论的成都市艾滋病干预效果影响因素分析[J].医学与社会，2012，25(01):21-23.
[603]朱艳文，阮师漫，胡艳霞，等.济南市不同活动场所男男性接触人群艾滋病病毒和梅毒感染状况调查[J].疾病监测，2012，27(08):631-633.

[604]黄文军，杨曦，孔飞勇，等.九江市 MSM 艾滋病相关行为学特征调查[J].医学动物防制，2012，28(07):744-745.
[605]章任重，张琬悦，李佑芳，等.昆明市男男性行为人群艾滋病新发感染队列研究[J].中华预防医学杂志，2012，46(10):955-957.
[606]王谨业.昆明市同性恋人群对 HIV 检测实名制的态度调查[J].中国性科学，2012，21(08):85-89.
[607]单飞，黄竹林，李叶兰.利用互联网对男男性行为人群进行艾滋病防治干预的可行性研究[J].中国艾滋病性病，2012，18(01):18-20.
[608]谭京广，程锦泉，卢祖询.联合干预模式对男男性行为人群的干预效果评价[J].中华预防医学杂志，2012，46(08):732-735.
[609]谭京广，卢祖询，程锦泉.联合营销干预模式推动男男性行为人群的高危行为干预效果研究[J].中国社会医学杂志，2012，29(01):52-54.
[610]周金玲，张莹，时景璞，等.辽宁省 MSM 艾滋病知识行为及感染率分析[J].中国公共卫生，2012，28(06):806-808.
[611]郑禄祥，阙金财，陈前进.龙岩市 2010-2011 年男男性行为人群 HIV 与行为学监测[J].海峡预防医学杂志，2012，18(04):34-35.
[612]孙智霞，薛芳辉，金镒华，等.鹿城区男男性行为人群 HIV 扩散流行方式分析[J].中国卫生检验杂志，2012，22(12):2983-2985.
[613]朱鑫.洛阳市男男性接触人群艾滋病病毒感染流行病学特征分析[J].医学动物防制，2012，28(07):730-732.
[614]王毅，徐杰，李志军，等.绵阳城区男男性行为者性伴特征及影响因素[J].预防医学情报杂志，2012，28(05):329-333.
[615]王毅，李六林，樊静，等.绵阳市 820 例男男性行为者艾滋病高危行为分析[J].中国热带医学，2012，12(11):1320-1322.
[616]王毅，李伟，孙玲，等.绵阳市安县男男性行为者 HIV 知识与行为及感染现状调查分析[J].中国病毒病杂志，2012，2(06):46-49.
[617]王毅，张光贵，李六林，等.绵阳市男男性行为人群 HIV/梅毒新发感染及影响因素分析[J].中国皮肤性病学杂志，2012，26(06):509-512.
[618]王毅，张光贵，李六林，等.绵阳市男男性行为人群 HIV 感染及影响因素研究[J].中国病毒病杂志，2012，2(04):271-276.
[619]王毅，徐杰，李志军，等.绵阳市男男性行为人群队列基线 HIV/梅毒感染及影响因素分析[J].中国皮肤性病学杂志，2012，26(05):410-414.
[620]王毅，张光贵，李六林，等.绵阳市男男性行为人群梅毒感染及影响因素分析[J].中国热带医学，2012，12(07):798-802.
[621]王毅，张光贵，李六林，等.绵阳市男男性行为人群随访队列艾滋病知识及相关行为变化研究[J].实用预防医学，2012，19(02):161-165.
[622]张永，石国政，殷方兰，等.某区男男性行为人群性伴网络特征与高危行为分析[J].环境与职业医学，2012，29(5):326-330.
[623]龚诚华，戴丽萍，云俊，等.男男同性性接触者异性性行为特征及影响因素研究[J].疾病监测，2012，27(10):776-779.
[624]薛芳辉，张文珠，金镒华，等.男男同性性行为人群 4 种性传播疾病感染状况及相关危险因素分析[J].疾病监测，2012，27(12):967-970.
[625]韩凌飞，文育锋，程险峰，等.男男同性性行为者 HEV 感染危险因素研究[J].中华疾病控制杂志，2012，16(07):586-589.

[626]胡晓燕，朱曦，陈磊.男男性接触梅毒感染患者 50 例的调查分析及护理对策[J].解放军护理杂志，2012，29(04):29-31.
[627]陈娆娆，李振忠，邱奕标.男男性接触人群艾滋病高危行为互联网 QQ 干预效果[J].热带医学杂志，2012，12(08):1022-1024.
[628]张琰，张丹丹，李怀亮，等.男男性接触人群艾滋病知识知晓情况及其影响因素分析[J].浙江预防医学，2012，24(03):76-78.
[629]王香梅.男男性接触人群性行为特征及艾滋病梅毒感染现状调查[J].中国药物与临床，2012，12(05):612-614.
[630]赵丽，刁文丽，赵文彬，等.男男性接触者的内在感受与高危性行为的关系分析[J].中国冶金工业医学杂志，2012，29(03):364-365.
[631]薛黎坚，王文明，徐勇，等.男男性行为人群(MSM)早期经历对成年后性行为及艾滋病预防控制的影响[J].数理医药学杂志，2012，25(03):340-343.
[632]徐洪吕，陆林，贾曼红，等.男男性行为人群 HIV 感染危险因素研究进展[J].医学研究杂志，2012，41(09):191-193.
[633]孙燕鸣，卢红艳，李洋，等.男男性行为人群 HIV 感染者/AIDS 病人耻辱感的影响因素[J].中国艾滋病性病，2012，18(08):518-520.
[634]汪敏，张洪波，王君，等.男男性行为人群 HIV 感染者 CD_4~+T 随访检测与抗病毒治疗状况及影响因素分析[J].中华疾病控制杂志，2012，16(12):1011-1014.
[635]何欢，汪敏，张洪波，等.男男性行为人群 HIV 感染者无保护性行为状况及其影响因素分析[J].中华预防医学杂志，2012，46(11):976-981.
[636]尹宁，倪莹青，朱晓华，等.男男性行为人群 HIV 相关行为学特征及其影响因素[J].环境与职业医学，2012，29(6):397-399,402.
[637]李现红，王红红，何国平，等.男男性行为人群艾滋病防治策略研究进展[J].护理研究，2012，26(24):2211-2213.
[638]刘国武，卢红艳，王娟，等.男男性行为人群艾滋病防治网络干预效果评价[J].中国艾滋病性病，2012，18(09):578-580.
[639]董蕾，韩磊，张晓菲.男男性行为人群安全套使用干预效果评价[J].中国健康教育，2012，28(10):810-812.
[640]王毅，徐杰，李志军，等.男男性行为人群不同性伴寻找场所 HIV 和梅毒感染及相关行为特征[J].中国艾滋病性病，2012，18(03):180-183.
[641]杨美霞，蔡晓峰，李申生，等.男男性行为人群健康及行为方式差异性研究[J].环境与职业医学，2012，29(3):194-195,封 4.
[642]李朋，张枭，毛军，等.男男性行为人群双性性行为的发生及影响因素[J].环境与职业医学，2012，29(6):400-402.
[643]石国政，殷方兰，张永，等.男男性行为人群同性恋身份自我认同与艾滋病危险因素的相关性[J].环境与职业医学，2012，29(5):331-333,封 4.
[644]贵蔷，刘穗军，彭继烈，等.男男性行为人群预防艾滋病/性病综合干预模式初探[J].中国艾滋病性病，2012，18(09):618-620.
[645]修翠珍，张秀云，姜珍霞.男男性行为者 294 例的艾滋病相关行为分析[J].职业与健康，2012，28(05):524-526.
[646]周建波，郝超，还锡萍，等.男男性行为者艾滋病病毒及梅毒感染状况调查[J].中国公共卫生，2012，28(08):1031-1035.
[647]王毅，徐杰，李志军，等.男男性行为者安全套使用及影响因素分析[J].中华疾病控制杂志，2012，16(02):133-136.

[648]王毅，李六林，张光贵，等.男男性行为者婚姻状况及其艾滋病相关行为特征调查[J].实用预防医学，2012，19(12):1772-1775.
[649]王毅，徐杰，李志军，等.男男性行为者女性性行为现状及影响因素分析[J].中国公共卫生，2012，28(09):1162-1165.
[650]殷方兰，石国政，任金马，等.男男性行为者社会网络及性行为特征的研究[J].环境与职业医学，2012，29(3):190-193.
[651]王毅，张光贵，李六林，等.男男性行为者生存质量及与人口学特征和社会活动网络的关系研究[J].实用预防医学，2012，19(10):1451-1455.
[652]张敏，闫红静，吴苏姝，等.男男性行为者未参加艾滋病抗体检测因素调查[J].江苏预防医学，2012，23(05):15-17.
[653]刘珺，郜艳晖，杨翌.男男性行为者抑郁症状流行病学研究现状[J].中华疾病控制杂志，2012，16(06):527-530.
[654]张万宏，石萍，石卫东，等.男性双性性行为者性取向及群体性行为特征分析[J].华中科技大学学报(医学版)，2012，41(03):375-378.
[655]曹林凤，胡有君，黄金文.男性同性恋抑郁症 26 例的心理干预[J].护理与康复，2012，11(08):767-768.
[656]刘黎，张敏.南京市男男性行为人群梅毒感染情况及影响因素调查[J].南京医科大学学报(自然科学版)，2012，32(07):1019-1023.
[657]黄秋芳.南宁市 MSM 人群特征及 4 项传染性指标的检测结果与分析[J].检验医学与临床，2012，9(03):353-354.
[658]曲琳，高永明，杨景元，等.内蒙古部分地区男男性行为人群 HIV 与梅毒螺旋体感染状况及影响因素[J].中华预防医学杂志，2012，46(11):1040-1043.
[659]施永辉，舒红萍，刘力.萍乡市 MSM 人群 HIV 感染危险性行为的调查分析[J].中国艾滋病性病，2012，18(12):882-884.
[660]郭向阳，尚小三，赵小平.潜江市男男性行为人群艾滋病知晓率及高危性行为调查分析[J].公共卫生与预防医学，2012，23(02):72-73.
[661]李晓华，徐朝阳，廖留妹，等.秦皇岛地区 803 例男男性接触者人口学及艾滋病相关高危行为状况调查[J].职业与健康，2012，28(19):2388-2389.
[662]贺生旭，张海东.青海省格尔木市 MSM 人群特征及 HIV/STD 流行病学分析[J].青海医药杂志，2012，42(04):87-88.
[663]刘亭亭.青年女同性恋者的身份认同研究[J].中国性科学，2012，21(12):80-87.
[664]黄鹏翔，王永香，廖玫珍，等.山东省男男性行为人群艾滋病梅毒感染率及其影响因素[J].中国艾滋病性病，2012，18(07):471-474.
[665]王永香，康殿民，廖玫珍，等.山东省男男性行为者安全套使用及影响因素分析[J].中国公共卫生，2012，28(11):1442-1445.
[666]石国政，康来仪，陈冬华，等.上海 MSM 人群对同志身份认同状况及其相关因素分析[J].中国艾滋病性病，2012，18(11):724-727.
[667]张永，石国政，殷方兰，等.上海 MSM 人群拥有性伴情况及其性行为特征[J].中国艾滋病性病，2012，18(11):720-723.
[668]孙乔，许磊，张枭，等.上海地区 MSM 人群艾滋病相关的需求分析[J].中国艾滋病性病，2012，18(11):732-734.
[669]贺江南，潘蓉，张晶，等.上海市 MSM 人群 HIV 和梅毒的感染率及其影响因素[J].中国艾滋病性病，2012，18(12):840-843.
[670]毛军，张枭，李朋，等.上海市浦东新区男男性行为人群艾滋病相关高危行为及社会学

特征分析[J].环境与职业医学，2012，29(1):52-55,59.
[671]李朋，张枭，毛军，等.上海市浦东新区男男性行为人群与社交对象间的性行为状况调查[J].环境与职业医学，2012，29(3):187-189.
[672]杨慧颖，查燕芬，罗珍，等.上海市松江区男男性行为人群相关高危险性行为及态度调查[J].环境与职业医学，2012，29(02):126-128.
[673]尹宁，倪莹青，石国政，等.上海市西部三区 MSM 人群的 HIV 相关行为学特征及影响因素[J].中国艾滋病性病，2012，18(11):728-731.
[674]蔡晓峰，杨美霞，宁镇.上海市徐汇区 MSM 人群艾滋病高危行为及健康需求分析[J].中国艾滋病性病，2012，18(09):581-583.
[675]宣舟斌，黄惠敏，侯云，等.上海市杨浦区 98 名男男性接触者性行为特征及 HIV 和梅毒感染状况[J].中华疾病控制杂志，2012，16(12):1028-1031.
[676]谭惠玲，龙清平，刘凤仁，等.深圳某区男男性行为(MSM)人群的行为特征与干预策略探讨[J].实用预防医学，2012，19(07):1092-1094.
[677]张倩，邓平建，耿艺介，等.深圳市 300 例 MSM 人群 HIV/梅毒感染状况及行为学特征[J].中国热带医学，2012，12(02):219-220.
[678]潘鹏，蔡于茂，洪福昌，等.深圳市 675 例男男性行为者高危行为及 HIV/梅毒感染调查[J].公共卫生与预防医学，2012，23(03):21-23.
[679]蔡于茂，宋亚娟，潘鹏，等.深圳市男男性行为人群 HIV 感染分布特征及影响因素[J].中国艾滋病性病，2012，18(10):659-661.
[680]陈琳，刘少础，赵锦，等.深圳市男男性行为人群不安全性行为状况及其影响因素[J].中国艾滋病性病，2012，18(04):248-249.
[681]蔡于茂，宋亚娟，潘鹏，等.深圳市男男性行为者梅毒感染分布特征及影响因素[J].中华疾病控制杂志，2012，16(12):1106-1108.
[682]王毅，李六林，张光贵，等.四川省绵阳市未婚男男性行为者婚姻意愿及影响因素分析[J].中华流行病学杂志，2012，33(10):1031-1035.
[683]李群，陈曦.四川省自贡市 2008～2010 年男男性行为者艾滋病哨点监测结果分析[J].现代预防医学，2012，39(12):3108-3110.
[684]佘敏，张洪波，王君，等.四座城市 MSM 人群同性恋与非同性恋者 HIV 和梅毒感染状况及相关因素[J].中华疾病控制杂志，2012，16(09):759-763.
[685]还锡萍，陈鑫，闫红静，等.苏州市 MSM 人群艾滋病高危行为特征、感染状况及影响因素分析[J].中国卫生统计，2012，29(02):202-205.
[686]徐亚斌, 张翔, 朱中奎, 等.泰州市男男性行为者中 HIV 感染情况分析[J].江苏预防医学，2012，23(05):50-51.
[687]任晖琴，亚克甫地力夏提.，艾拜米娜瓦尔.，等.乌鲁木齐市男男性行为人群 HIV/STD 新发感染率及其影响因素[J].新疆医科大学学报，2012，35(11):1495-1499.
[688]郑武，王梅，吴炽煦，等.武汉市男男性行为者自杀行为影响因素分析[J].公共卫生与预防医学，2012，23(01):51-55.
[689]杨芳，李玲，杨树星，等.武汉市在校大学生男男性行为者心理健康状况研究[J].中华流行病学杂志，2012，33(11):1139-1140.
[690]张军辉，黄建华，吾买尔江，等.新疆阿克苏地区男男性行为人群健康状况调查分析[J].疾病预防控制通报，2012，27(03):4-5.
[691]徐智贤.邢台市农村男男性接触者人群 HIV/HCV/梅毒感染状况及性行为特征分析[J].现代预防医学，2012，39(24):6484-6485.
[692]童晶，杨晋川，杨喜玲.徐州地区男男性行为者艾滋病、梅毒、丙肝合并感染状况调查

[J].中国卫生检验杂志，2012，22(10):2491-2492.
[693]段志才，刘吉姝，蔡斌郁.宣化区男男性接触者艾滋病感染及高危行为状况调查研究[J].医学动物防制，2012，28(03):322-323.
[694]陈鑫，张军，还锡萍，等.扬州市 MSM 人群艾滋病高危行为特征、感染状况及影响因素分析[J].南京医科大学学报(自然科学版)，2012，32(04):479-486.
[695]李玉凤，许建，李鹏.一般男男性行为者和男男性工作者艾滋病感染情况及相关知识行为调查[J].中华行为医学与脑科学杂志，2012，21(4):346-348.
[696]赵培来，陈韵，李燕娃，等.依托性病门诊开展男男性行为人群梅毒感染调查[J].皮肤性病诊疗学杂志，2012，19(04):244-246.
[697]周加华, 赵卫红, 莫尚鹏, 等.宜春市男男性接触者性心理及行为调查[J].现代预防医学，2012，39(07):1710-1711.
[698]阮建军，朱碧香，贾东谨.义乌市男男性行为人群三种性病感染状况及行为学特征调查[J].上海预防医学，2012，24(01):18-19.
[699]苗志峰，姜峰，韩雪，等.银川市 MSM 人群梅毒、艾滋病预防知识及感染现状调查[J].宁夏医科大学学报，2012，34(09):910-911.
[700]陈克江，赵希友，陈亮，等.永川区男男性行为人群 HIV、梅毒感染率及高危行为特征分析[J].重庆医学，2012，41(17):1730-1733.
[701]罗红兵，梅静远，宋丽军，等.云南省 2010 年 13 州(市)男男性行为者艾滋病综合监测[J].中华流行病学杂志，2012，33(07):706-709.
[702]张勇，何震，王春，等.云南省普洱市男男性行为者艾滋病病毒感染状况和危险行为调查[J].卫生软科学，2012，26(09):797-799.
[703]曹臻，张洪波，佘敏，等.在婚男男性行为人群 HIV 感染及其双性性行为状况调查[J].中华流行病学杂志，2012，33(05):488-491.
[704]郭伟，孟晓军，张云建，等.长春市男男性接触人群 HIV 感染情况及其影响因素[J].中华疾病控制杂志，2012，16(01):32-35.
[705]董正全，金玫华，邱志红，等.浙江省湖州市 410 例男男性行为者艾滋病知识和高危行为调查分析[J].疾病监测，2012，27(08):623-626.
[706]刘晓霞，张明辉，陈鑫，等.镇江市男男性行为人群前瞻性队列研究分析[J].中华疾病控制杂志，2012，16(12):1024-1027.
[707]佘敏，张洪波，王君，等.中国四座城市男男性行为者 HIV 和梅毒螺旋体感染状况及危险性行为调查[J].中华预防医学杂志，2012，46(04):324-328.
[708]丁凡，周生建，张洪波，等.重庆市城区农民工男男性行为者艾滋病性病知识及性行为的定性研究[J].中国艾滋病性病，2012，18(07):475-476.
[709]冯艳洁，曹丛烨，王志刚，等.主动求询与被动检测的男男性行为人群艾滋病性病感染情况分析[J].中国皮肤性病学杂志，2013，27(11):1134-1136.
[710]张枭，黄星，孙乔，等.综合干预对 MSM 的艾滋病相关知识行为及 HIV 感染率的影响[J].中国艾滋病性病，2013，19(10):750-752.
[711]陈洋, 申莉梅, 姚永明, 等.1045 例 MSMHIV 感染状况及影响因素[J].中国艾滋病性病，2013，19(06):402-405.
[712]罗思童，徐杰，米国栋，等.19 名新近感染 HIV 的 MSM 感染来源及相关原因的定性调查[J].中国艾滋病性病，2013，19(09):644-647.
[713]蔡于茂，洪福昌.2005-2011 年深圳市 MSM 献血者梅毒/HIV 感染调查[J].中国艾滋病性病，2013，19(04):251-253.
[714]蔡于茂，宋亚娟，潘鹏，等.2005—2011 年深圳市男男性行为者梅毒/HIV 流行趋势分析

[J].华南预防医学，2013，39(01):32-34.
[715]白玉，黎明强，谭丽娟.2007—2011 年柳州市男男性接触者艾滋病综合监测结果[J].职业与健康，2013，29(24):3334-3335.
[716]常文辉，邢爱华，王百锁，等.2007—2011 年陕西省男男性行为者哨点监测结果[J].职业与健康，2013，29(09):1058-1061.
[717]周金玲，周丹，姜凤霞，等.2008-2010 年辽宁省 MSM 人群艾滋病疫情和相关行为监测结果分析[J].中国艾滋病性病，2013，19(03):177-179.
[718]张枭，周洁，黄星，等.2008-2012 年浦东新区 MSM 人群 HIV 感染与行为现状调查[J].医学与社会，2013，26(05):51-52.
[719]刘黎，张敏，朱正平，等.2009-2011 年南京市 MSM 人群艾滋病哨点监测结果分析[J].中国艾滋病性病，2013，19(01):36-38.
[720]吴建英.2009-2011 年青海地区男男性行为者 HIV-1 新发感染率调查[J].医学动物防制，2013，29(07):792-793.
[721]杨珊，程晓松，姜聚军，等.2009-2011 年烟台市男男性行为人群艾滋病高危行为变化及影响因素研究[J].中华疾病控制杂志，2013，17(08):666-669.
[722]张琰，张丹丹，洪航.2010-2012 年宁波市男男性接触者艾滋病感染状况及高危行为动态分析[J].疾病监测，2013，28(08):639-642.
[723]杜桂英，于海荣.2010 年聊城市艾滋病自愿咨询检测门诊就诊者 MSM 人群艾滋病相关知识、行为调查[J].预防医学论坛，2013，19(04):272-274.
[724]杜桂英，于海荣.2010 年聊城市男男性行为人群艾滋病知识、态度、行为及 HIV/梅毒/丙肝感染情况调查[J].预防医学论坛，2013，19(02):93-94.
[725]陈淑霞，张梅烈，韩雪梅.2011~2012 年高密市男男同性恋人群艾滋病相关知识行为与感染状况调查[J].中国药物经济学，2013(S3):410-411.
[726]崔倩，俞文祥，祁耀，等.2011～2012 年盐城市男男性行为者艾滋病相关知识知晓情况及影响因素调查[J].预防医学论坛，2013，19(12):884-886.
[727]王海连，王伟，褚红娜，等.2011—2012 年承德市男男性行为者 HIV 和梅毒感染状况[J].职业与健康，2013，29(19):2521-2522.
[728]冯学茹，龙霏，辛丽香.2011 年保定市 MSM 的 HIV/HCV/梅毒感染情况[J].中国艾滋病性病，2013，19(07):539-541.
[729]林丽，陈舸，张宏，等.2011 年福建省部分男男性行为人群梅毒知识知晓率调查[J].预防医学论坛，2013，19(10):734-736.
[730]郑文彬，朱秋映，朱金辉，等.2011 年广西男男性接触者艾滋病监测结果分析[J].中华疾病控制杂志，2013，17(03):239-242.
[731]李文宇, 张珊珊.2012 年晋江市男男性行为人群艾滋病哨点监测信息分析[J].社区医学杂志，2013，11(06):24-26.
[732]杜桂英，蔡新，于海荣，等.2013 年聊城市部分女女性行为人群艾滋病相关知识及行为调查[J].预防医学论坛，2013，19(06):436-438.
[733]朱银霞，管文辉，魏庆，等.217 例接受 VCT 男男性行为者安全套使用情况分析[J].江苏预防医学，2013，24(03):33-34.
[734]罗晓英，石仁其.247 名男男性行为者艾滋病知信行调查[J].浙江预防医学，2013，25(05):83-84.
[735]周艳秋，朱小珍，高洁，等.294 名男男性行为者艾滋病相关知识、态度和行为调查研究[J].中国初级卫生保健，2013，27(06):52-53.
[736]董旭峰，雷静，苗志峰，等.400 名男男性行为人群艾滋病高危行为及其影响因素调查分

析[J].宁夏医科大学学报，2013，35(03):277-279.
[737]赵烨，马颖，韦晓岑，等.402 例男男性行为者社会网络与高危行为的关系研究[J].中华疾病控制杂志，2013，17(07):567-570.
[738]吕金伟，杨康生，程周祥，等.434 名男男性行为者艾滋病知识和行为调查[J].热带医学杂志，2013，13(03):345-347.
[739]蔡高峰，马瞧勤，潘晓红，等.HIV 抗体阴性男男性行为人群队列保持率及其影响因素研究[J].中国预防医学杂志，2013，14(10):724-726.
[740]张国磊，姜影，梁欣，等.HIV 唾液快速检测在公园场所 MSM 人群中的应用[J].中国艾滋病性病，2013，19(04):261-263.
[741]蓝光华，刘伟，梁富雄，等.MSMHIV、梅毒、HSV-2 新发感染及队列保持影响因素[J].中国公共卫生，2013，29(02):175-178.
[742]孙燕鸣，赵月娟，李洋，等.MSM 人群中 HIV 感染者/病人生存质量现状及影响因素初探[J].现代预防医学，2013，40(16):2964-2966.
[743]许骏，罗西，刘司航，等.MSM 中 HIV 抗体阳性率及其影响因素分析[J].中国艾滋病性病，2013，19(12):867-869.
[744]李莉，闫红静，管文辉，等.VCT 门诊男男性行为者 HIV、梅毒感染状况及高危行为影响因素分析[J].江苏预防医学，2013，24(05):8-10.
[745]李现红，刘博，赵俊仕，等.艾滋病相关知识结构对男男性行为者安全套使用率的影响[J].中国全科医学，2013，16(13):1170-1172.
[746]杜建华，杨玉荣，田俊丽，等.包头市 MSM 人群 HIV 感染状况及其影响因素分析[J].中国艾滋病性病，2013，19(04):248-250.
[747]杨家芳，胡安艳，郭建华，等.保山市男男性行为人群性行为特征及 HIV 感染状况分析[J].医学动物防制，2013，29(03):244-246.
[748]徐青，杨传坤，闫红静，等.暴露前预防在降低 MSM 人群 HIV 新发感染中的应用[J].江苏预防医学，2013，24(06):45-47.
[749]于佳，李东亮，王子昕，等.北京市 MSM 人群的心理健康影响因素[J].中国艾滋病性病，2013，19(04):244-247.
[750]徐敏，陈婧，李洋，等.北京市 MSM 中流动人口艾滋病抗病毒治疗前特征研究[J].实用预防医学，2013，20(10):1206-1207.
[751]韩扬，夏冬艳，孙燕鸣，等.北京市部分男男性行为人群 HIV 感染情况及影响因素[J].中国艾滋病性病，2013，19(06):399-401.
[752]杨烨，夏斌.北京市朝阳区男男性接触者异性性行为及 HIV 和梅毒感染调查[J].慢性病学杂志，2013，14(03):194-196.
[753]刘英杰，姜树林，吴玮，等.北京市朝阳区男男性行为者艾滋病相关知识和性行为及血清学调查[J].慢性病学杂志，2013，14(04):261-265.
[754]廖娜，于建平，陈晓霞，等.北京市男男性接触者 HIV 与肛门高危型 HPV 感染的关联性研究[J].现代预防医学，2013，40(11):2154-2158.
[755]杨雪盈，周枫，肖冬，等.北京市男男性接触者对艾滋病新型干预策略可接受性的定性研究[J].慢性病学杂志，2013，14(09):649-653.
[756]齐啸，徐杰，张政，等.北京市男男性行为人群 HIV 和梅毒本底感染及新发感染调查研究[J].中国预防医学杂志，2013，14(06):407-412.
[757]王春艳，丁海峰，杨雪盈，等.北京市男男性行为人群艾滋病病毒和人乳头瘤病毒感染状况分析[J].中国预防医学杂志，2013，14(07):542-543.
[758]罗凤基，李东亮，许瑜楠，等.北京市男男性行为者 HIV 快速检测及检测服务方式需求

的调查研究[J].中国预防医学杂志，2013，14(08):561-566.
[759]王娜，孙燕鸣，卢红艳，等.北京市年轻 MSM 人群 HIV 感染情况调查[J].中国艾滋病性病，2013，19(04):241-243.
[760]林桂芹，房本兰.本溪市 MSM 人群中 HIV 和梅毒感染状况调查[J].职业与健康，2013，29(06):636-769.
[761]杨家芳，胡安艳，郭建华，等.边境地区 MSM 人群艾滋病知识及相关行为调查[J].中国艾滋病性病，2013，19(01):76-80.
[762]曲琳，高永明，青河，等.不同活动场所男男性接触人群 HIV 感染状况及行为学研究[J].实用预防医学，2013，20(02):145-149.
[763]蒋均，徐杰，刘征，等.不同活动场所男男性行为人群 HIV 检测比例及影响因素分析[J].中华疾病控制杂志，2013，17(05):396-399.
[764]于增照，李洋，史同新，等.不同性传播感染 MSM 艾滋病高危行为对比分析[J].中国公共卫生，2013，29(08):1114-1117.
[765]周建波，王金塔，郭燕丽，等.常州市男男性行为人群多性伴影响因素及 HIV 和梅毒感染情况调查[J].中国皮肤性病学杂志，2013，27(04):371-373.
[766]姜维华，何勤英，范双凤.成都市男男性行为人群艾滋病感染状况及危险因素分析[J].预防医学情报杂志，2013，29(01):36-40.
[767]黄玉玲，陈怀良，戴映雪，等.成都市男男性行为人群安全套使用影响因素分析[J].现代预防医学，2013，40(21):3987-3990.
[768]曲波，张阳，王东博，等.大学生男男性接触人群艾滋病高危性行为特征研究[J].中国卫生统计，2013，30(02):255-256.
[769]高洁，李洲林，曹东冬，等.德宏州男男性接触人群艾滋病性病感染现况调查[J].医学动物防制，2013，29(09):945-948.
[770]陈柏芬，朱建琼，张巧利，等.东莞市 MSM 高危行为特征及 HIV 感染状况[J].中国艾滋病性病，2013，19(12):873-876.
[771]黄星，孙乔，张枭，等.独子与非独子 MSM 的 HIV 感染状况和生存心理特征分析[J].中国艾滋病性病，2013，19(09):654-656.
[772]胡春华，袁彩艳，石萍，等.对男男性接触者(MSM)肛门尖锐湿疣患者进行健康教育效果观察[J].中国医药科学，2013，3(23):64-65.
[773]周建波，王金塔，甄森，等.对男男性行为者以防治性病为主预防艾滋病综合干预效果评价[J].中国预防医学杂志，2013，14(06):417-421.
[774]管纪惠，欧光忠，陆风.福建泉州社会组织参与 MSM 人群干预活动效果分析[J].江苏预防医学，2013，24(04):3-5.
[775]颜苹苹，张春阳，梁菲菲，等.福建省 2012 年男男性行为人群 HIV 感染率与新发感染率调查[J].海峡预防医学杂志，2013，19(02):1-2.
[776]颜苹苹，林勋，陈亮，等.福建省哨点监测 MSM 人群 HIV 感染率及影响因素分析[J].海峡预防医学杂志，2013，19(03):19-21.
[777]蔡于茂，刘惠，洪福昌，等.广东省深圳地区 MSM 并行性多性伴的影响因素[J].中国艾滋病性病，2013，19(09):648-650.
[778]郑文彬，朱秋映，蓝光华，等.广西 MSM 接受艾滋病干预服务情况及其影响因素分析[J].中国热带医学，2013，13(02):175-179.
[779]戴丽萍，林玉虾，刘奇，等.广州市不同性角色 MSM 行为特征及艾滋病感染分析[J].中国公共卫生，2013，29(02):256-258.
[780]秦鸥，周健，袁飞，等.贵阳市 MSM 人群艾滋病感染危险因素分析和新发感染率调查

[J].中国皮肤性病学杂志，2013，27(12):1261-1263.
[781]李婷，闫红梅，张婕，等.哈尔滨市男男性行为者 HIV 感染相关危险因素的定性调查[J].中国公共卫生管理，2013，29(05):617-618.
[782]何滨，宣冬青，曾庆梅，等.海口市 158 例 MSM 性行为特征及其安全套使用情况[J].中国热带医学，2013，13(07):832-834.
[783]武海滨，罗艳，程洁，等.杭州市 HIV 阳性男男性行为人群梅毒感染状况及危险因素分析[J].浙江预防医学，2013，25(07):25-28.
[784]李西婷，罗艳，武海滨，等.杭州市男男性行为人群 HIV 和梅毒螺旋体血清抗体阳转情况队列研究[J].疾病监测，2013，28(04):280-283.
[785]武海滨，罗艳，丁建明，等.杭州市男男性行为人群和单阳家庭中 HIV 感染者早期抗病毒治疗可接受性调查[J].疾病监测，2013，28(10):829-832.
[786]朱义彬，张小鹏，姚晖，等.合肥市男男性行为者同伴群干预降低艾滋病高危行为队列研究[J].安徽预防医学杂志，2013，19(04):240-242.
[787]胡中旺，类延花，李钰，等.合肥市男性同性恋人群部分血源及性传播疾病感染状况调查[J].安徽预防医学杂志，2013，19(01):7-8.
[788]胡玲玲，祖淑文，魏宁.河北省省会男男同性恋人群感染艾滋病相关高危行为研究[J].河北医药，2013，35(21):3305-3308.
[789]闫世春，王开利，李一，等.黑龙江省男男性接触人群艾滋病流行趋势及影响因素[J].中国公共卫生管理，2013，29(01):64-66.
[790]刘冰，邱辉.黑龙江省男男性行为 HIV 感染者人群生存质量调查[J].哈尔滨医科大学学报，2013，47(01):71-74.
[791]端琼丽，张维义，常铃，等.红河州 MSM 艾滋病相关知识及行为的随访调查[J].中国艾滋病性病，2013，19(12):870-872.
[792]罗艳，鲍其国，张晓华.淮南市男男性行为者艾滋病相关高危行为及影响因素分析[J].安徽预防医学杂志，2013，19(02):83-86.
[793]潘晶，韩秀云，徐丽芳，等.济南市 MSM 人群特征及 HIV/梅毒感染状况调查[J].中国公共卫生管理，2013，29(06):782-783.
[794]徐金水，还锡萍，刘晓燕，等.江苏省 3348 名男男性接触人群艾滋病和梅毒感染状况分析[J].现代预防医学，2013，40(09):1678-1680.
[795]杨传坤，徐青，胡海洋，等.江苏省 HIV 阳性的 MSM 人群梅毒和 HSV-2 感染及影响因素分析[J].南京医科大学学报(自然科学版)，2013，33(05):672-675.
[796]刘排，曹宁校，施美琴，等.江苏省部分城市男男性接触者性传播疾病流行情况调查[J].中华皮肤科杂志，2013，46(09):626-629.
[797]郭燕丽，周建波，郝超，等.江苏省常州市男男性行为者婚姻状况及其对艾滋病高危行为和感染率的影响[J].中华流行病学杂志，2013，34(01):19-23.
[798]刘江，周欢，罗刚.江油市 2011 年男男性行为人群哨点监测结果分析[J].中国皮肤性病学杂志，2013，27(12):1264-1265.
[799]唐慧玲，张涛，庞志峰.金华市 MSM 人群 HIV 阳性者某些特征分析[J].中国艾滋病性病，2013，19(11):809-811.
[800]周爽，冯连贵，丁贤彬，等.进城务工人员中男男性行为人群 HIV 感染率及其影响因素分析[J].第三军医大学学报，2013，35(01):46-49.
[801]孙红敏，张茜，黄芬.抗逆转录病毒治疗用于男男性行为人群艾滋病病毒暴露前预防[J].国际流行病学传染病学杂志，2013，40(06):414-416.
[802]徐洪吕，贾曼红，闵向东，等.昆明男男性行为者安全套使用及影响因素分析[J].中国公

共卫生，2013，29(11):1659-1662.
[803]闵向东，王珏，章任重，等.昆明市男男性行为人群 HIV 感染及影响因素分析[J].中国公共卫生，2013，29(12):1729-1731.
[804]徐洪吕，贾曼红，余春洁，等.昆明市男男性行为人群艾滋病知晓率及影响因素分析[J].中华疾病控制杂志，2013，17(04):314-316.
[805]徐洪吕，陆林，闵向东，等.昆明市男男性行为人群与艾滋病相关的社会需求分析[J].中国社会医学杂志，2013，30(01):19-21.
[806]闫红梅，高聪，李一，等.利用 QQ 开展 MSM 人群艾滋病高危行为干预效果评估[J].中国艾滋病性病，2013，19(03):174-176.
[807]金玫华，杨中荣，董正全.利用互联网开展 MSM 艾滋病知识干预效果评估[J].中国艾滋病性病，2013，19(09):695.
[808]周金玲，吴少慧，周丹，等.辽宁省 MSM 人群 HIV 感染及相关知识、行为变化趋势[J].中国公共卫生，2013，29(12):1732-1734.
[809]杜桂英，于海荣.聊城市男男性行为人群艾滋病高危行为及其影响因素分析[J].中国公共卫生管理，2013，29(05):611-612.
[810]吴庆戎，李杰，陈晨，等.泸州市男男性行为人群行为干预效果分析[J].预防医学情报杂志，2013，29(08):640-643.
[811]王毅，李六林，樊静，等.绵阳市 820 例男男性行为者艾滋病知识知晓及预防服务分析[J].预防医学情报杂志，2013，29(08):654-658.
[812]王毅，李六林，樊静，等.绵阳市 820 名 MSMHIV/梅毒感染现状及影响因素分析[J].中国艾滋病性病，2013，19(04):254-258.
[813]王毅，李六林，张光贵，等.绵阳市 MSM 人群的生存质量及其影响因素研究[J].中国艾滋病性病，2013，19(03):180-184.
[814]王毅，李六林，张光贵，等.绵阳市男男性行为人群研究队列 HIV/梅毒新发感染及行为变化分析[J].实用预防医学，2013，20(12):1431-1435.
[815]王毅，徐杰，李志军，等.绵阳市男男性行为者近 6 月性行为特征及其影响因素分析[J].现代预防医学，2013，40(01):64-69.
[816]王毅，李六林，樊静，等.绵阳市男男性行为者吸毒行为与 HIV 感染及相关行为的关系研究[J].中华行为医学与脑科学杂志，2013，22(04):364-366.
[817]王毅，李六林，樊静，等.绵阳市男男性行为者性行为和 HIV 感染及影响因素分析[J].实用预防医学，2013，20(10):1166-1169.
[818]吕金伟，程周祥，杨康生，等.某市不同类型 MSM 艾滋病知识态度行为和 HIV 感染情况[J].中华疾病控制杂志，2013，17(09):774-777.
[819]吴天亮，张健，陈国永，等.男男同性恋常见精神健康问题及成因探析[J].中国性科学，2013，22(09):85-87.
[820]蒋慧惠，吕繁，何慧婧，等.男男性接触 HIV 感染者早期抗病毒治疗可接受性状况调查[J].中华预防医学杂志，2013，47(09):843-847.
[821]邱兴庆，张静，谢爱齐，等.男男性接触人群的自我效能对艾滋病知识行为影响分析[J].实用预防医学，2013，20(11):1297-1300.
[822]舒彬，司徒潮满，刘莹，等.男男性接触人群高危行为干预效果分析[J].中国热带医学，2013，13(05):592-594.
[823]王炜，邹亚洲，郝晓刚.男男性接触者艾滋病梅毒感染及危险因素调查[J].浙江预防医学，2013，25(11):43-44.
[824]林菲菲，张铁军，姜庆五，等.男男性接触者人群中卡波氏肉瘤相关疱疹病毒的流行病

学研究现状[J].中国预防医学杂志，2013，14(09):709-712.
[825]李现红，刘立珍，何国平，等.男男性行为 HIV 感染者艾滋病相关羞辱和歧视的调查分析[J].中华护理杂志，2013，48(02):142-146.
[826]肖丽，王标，王健，等.男男性行为人群 HIV、梅毒和 HCV 专题调查结果分析[J].皮肤病与性病，2013，35(02):112-113.
[827]王万海，张晓燕，明亮，等.男男性行为人群 HIV 阳性者性行为特征及相关因素分析[J].中华预防医学杂志，2013，47(01):90-92.
[828]张燕，钟晓妮，彭斌，等.男男性行为人群艾滋病暴露前预防用药相关情况分析[J].第三军医大学学报，2013，35(20):2226-2230.
[829]陈树昶，罗艳，许珂，等.男男性行为人群艾滋病流行趋势及安全套使用影响因素分析[J].浙江预防医学，2013，25(12):25-28.
[830]田志伟，赵淑娟，牛留业，等.男男性行为人群艾滋病知识、行为及 HIV 和梅毒感染调查[J].中国公共卫生，2013，29(09):1330-1333.
[831]杨介者，潘晓红，马瞧勤，等.男男性行为人群安全套使用及影响因素分析[J].浙江预防医学，2013，25(05):9-12.
[832]佘颖，钟晓妮，张燕，等.男男性行为人群安全套使用相关行为的保护动机研究[J].重庆医科大学学报，2013，38(04):400-404.
[833]陈琳，潘晓红，蔡高峰，等.男男性行为人群定期检测的影响因素研究[J].浙江预防医学，2013，25(01):1-4.
[834]马功燕，姜正好，陈海燕，等.男男性行为人群多性伴行为及影响因素分析[J].安徽预防医学杂志，2013，19(06):414-416.
[835]崔巍，袁海燕.男男性行为人群现状与艾滋病行为干预研究[J].医学动物防制，2013，29(06):630-633.
[836]张静瑜，李青青，韩德琳，等.男男性行为人群性角色状况及其与安全套使用和 HIV 检测的关系[J].中华疾病控制杂志，2013，17(05):400-403.
[837]王毅，李六林，张光贵，等.男男性行为者艾滋病知识知晓及安全套使用现状调查[J].华南预防医学，2013，39(02):65-68.
[838]王毅，李六林，张光贵，等.男男性行为者公开性取向与艾滋病相关行为的关系研究[J].中国预防医学杂志，2013，14(10):727-731.
[839]王毅，李六林，张光贵，等.男男性行为者婚姻/婚姻意愿现状及影响因素分析[J].中国公共卫生，2013，29(08):1205-1208.
[840]陈再芳，宋均，梁敏，等.男男性行为者接受艾滋病自愿咨询检测服务[J].公共卫生与预防医学，2013，24(06):60-63.
[841]蔡于茂，宋亚娟，潘鹏，等.男男性行为者梅毒合并 HIV 感染影响因素分析[J].中国公共卫生，2013，29(08):1127-1130.
[842]刘莹，刘小敏，舒彬，等.男男性行为者商业性性行为特征研究[J].中国热带医学，2013，13(03):330-332.
[843]王毅，张光贵，李六林，等.男男性行为者生存质量与性行为及艾滋病预防服务的关系研究[J].中华疾病控制杂志，2013，17(08):659-665.
[844]王毅，李六林，张光贵，等.男男性行为者网络活动及网络性伴相关社会行为特征研究[J].实用预防医学，2013，20(03):260-263.
[845]王毅，李六林，张光贵，等.男男性行为者性伴艾滋病高危行为知晓及影响因素[J].职业与健康，2013，29(15):1857-1860.
[846]胡静初，胡纪泽，萧嘉慰.男同性恋者的孤独感、自尊和依恋[J].中国心理卫生杂志，2013，

27(12):930-936.
[847]曹越，李十月，路亮，等.南昌市不同性取向男男性行为人群特征比较分析[J].公共卫生与预防医学，2013，24(04):62-66.
[848]农全兴，石健，徐永芳，等.南宁市男男性行为人群 HIV 感染率及影响因素[J].中国公共卫生，2013，29(05):682-684.
[849]王卫民，赵涛，施建春，等.南阳市 MSM 人群 HIV 新发感染情况研究[J].中国艾滋病性病，2013(1):39-42.
[850]杜桂英，于海荣，蔡新，等.女女性行为人群的性行为特征和性传播疾病感染状况[J].中国艾滋病性病，2013，19(10):747-749.
[851]付广建，田考聪，钟晓妮，等.女性性工作者和男男性行为者对经网络获得艾滋病相关知识的态度及影响因素分析[J].上海交通大学学报(医学版)，2013，33(03):345-353.
[852]冯艳洁，王志刚，王岚.秦皇岛市男男性行为者艾滋病相关行为特征及感染状况调查[J].中国皮肤性病学杂志，2013，27(03):271-273.
[853]张建明，李春霞，李永喜，等.青岛地区 MSM 中 HIV/梅毒及尖锐湿疣的感染情况[J].中国艾滋病性病，2013，19(08):583-584.
[854]孙玉兰，马永成，樊明，等.青海省男男性行为人群艾滋病知识行为及感染状况分析[J].医学动物防制，2013，29(08):844-846.
[855]龚苍涛，王增强，杨育红.泉州市 2010—2012 年男男性行为人群哨点监测结果分析[J].海峡预防医学杂志，2013，19(06):31-32.
[856]陈莲芬，陈朱，唐贤龙，等.三亚市 2012 年男男性行为人群艾滋病流行病学调查[J].中国热带医学，2013，13(07):829-831.
[857]陈莲芬，陈朱，唐贤龙，等.三亚市高校学生男男性行为人群艾滋病知识及相关疾病流行现状[J].中国学校卫生，2013，34(09):1036-1038.
[858]郑惠能，马桂林，林碧云，等.厦门市 2010—2012 年男男性行为人群艾滋病哨点监测分析[J].海峡预防医学杂志，2013，19(06):29-30.
[859]钱跃升，陶小润，杨兴光.山东省 1378 名男男性行为人群艾滋病感染状况及相关因素调查[J].中国病原生物学杂志，2013，8(10):921-923.
[860]石国政，殷方兰，张永，等.上海市嘉定区男男性接触者 HIV 抗体检测服务利用状况调查[J].健康教育与健康促进，2013，8(05):349-350.
[861]黄星，孙乔，张枭，等.上海市浦东新区 MSM 人群安全套使用及性行为特征分析[J].职业与健康，2013，29(17):2127-2129.
[862]白璐，徐震雷，汤海明.社会性别规范与男同性恋者性身份认同[J].中国性科学，2013，22(03):78-83.
[863]黄雪颜，谢霖，李美婷，等.社区卫生服务机构对男男性行为人群开展艾滋病梅毒干预检测的效果分析[J].皮肤性病诊疗学杂志，2013，20(03):213-216.
[864]舒彬，司徒潮满，刘莹，等.深圳男男性行为者艾滋病哨点监测情况分析[J].实用预防医学，2013，20(06):694-696.
[865]宋亚娟，蔡于茂，洪福昌.深圳市 2009 ~ 2011 年男男性行为人群 HIV 感染及影响因素[J].中国热带医学，2013，13(07):815-818.
[866]赖永珲，蔡于茂，宋亚娟，等.深圳市 2011 年 MSM 高危行为特征及梅毒/HIV 感染调查[J].中国热带医学，2013，13(02):226-227.
[867]蔡于茂，宋亚娟，洪福昌.深圳市 2943 例 MSM 梅毒感染影响因素分析[J].中国艾滋病性病，2013，19(10):756-759.
[868]张欣，蔡于茂，宋亚娟.深圳市 502 例男男性行为者 HIV/梅毒感染调查[J].中国热带医

学，2013，13(12):1500-1502.
[869]蔡于茂，宋亚娟，洪福昌.深圳市 MSM 利用自愿咨询检测服务的影响因素[J].中国艾滋病性病，2013，19(07):503-506.
[870]谭唯，赵锦，陈琳，等.深圳市 MSM 人群的 HIV 感染状况及相关危险因素[J].中国艾滋病性病，2013，19(11):805-808.
[871]齐杰，李林涛，张燕，等.深圳市罗湖区 MSM 的 HIV 感染情况及相关危险因素[J].中国艾滋病性病，2013，19(07):500-502.
[872]蔡于茂，洪福昌，冯铁建，等.深圳市男男性行为人群 HIV/梅毒发病密度研究[J].中国艾滋病性病，2013，19(02):125-126.
[873]蔡于茂，宋亚娟，洪福昌.深圳市男男性行为献血者梅毒分布特征及影响因素[J].中国热带医学，2013，13(03):303-306.
[874]宋亚娟，蔡于茂，洪福昌.深圳市男男性行为者双性性行为特征及影响因素[J].中国热带医学，2013，13(04):424-426.
[875]宋亚娟，蔡于茂，洪福昌.深圳市男性双性性行为者高危行为及梅毒/HIV 感染调查[J].中国热带医学，2013，13(11):1329-1331.
[876]宋亚娟，蔡于茂，张玲华，等.深圳市女同性恋人群生殖健康影响因素分析[J].中国热带医学，2013，13(02):144-146.
[877]刘淑君，李洁芳，刘丽花，等.石家庄市 450 名男男性行为者 HIV 及梅毒感染情况调查[J].河北医药，2013，35(01):129-130.
[878]邓敏莉，徐杰，斗智，等.使用互联网交友的 MSM 最近 1 年 HIV 检测情况和抗病毒治疗意愿初步调查[J].中国艾滋病性病，2013，19(02):131-133.
[879]王红梅，付中建，杨海霞，等.寿光市 MSM 艾滋病知识知晓率及高危行为调查[J].中国艾滋病性病，2013，19(05):358-359.
[880]王毅，李六林，樊静，等.四川绵阳地区 MSM 群交行为 HIV/梅毒感染及影响因素[J].中国热带医学，2013，13(10):1203-1206.
[881]赵秀萍，傅卓华，曹小平，等.苏州市 MSM 人群定期艾滋病自愿咨询检测的相关因素研究[J].中华疾病控制杂志，2013，17(12):1030-1033.
[882]何金奎，曹慧珍，张志坤，等.唐山市男男同性恋艾滋病知识与高危行为状况调查[J].河北医药，2013，35(03):442-443.
[883]郭燕，董笑月，王欣，等.天津市 2008-2011 年 MSM 人群 HIV 流行趋势分析[J].中国艾滋病性病，2013，19(01):33-35.
[884]吴丹，程绍辉，杨杰，等.同性恋浴池 MSM 人群 HIV 感染状况及影响因素调查[J].中国艾滋病性病，2013，19(02):127-130.
[885]尚红.我国男男性行为人群中人类免疫缺陷病毒感染疫情特点及防治策略[J].微生物与感染，2013，8(04):197-202.
[886]梁晓倩，寇香君，范向阳.我国女同性恋自尊感与主观幸福感的关系[J].长江大学学报(社会科学版)，2013，36(09):196-197.
[887]吴鹏.无锡市男男性行为人群 HIV、梅毒、丙肝感染状况调查[J].江苏卫生保健，2013，15(02):4-5.
[888]赵鑫，李恒新，卫晓丽，等.西安市农民工 MSM 对艾滋病的认知和 HIV 感染状况[J].中国艾滋病性病，2013，19(09):651-653.
[889]王朝才，梁军，马永成，等.西宁市 MSM 人群艾滋病感染状况及其危险因素分析[J].医学动物防制，2013，29(04):372-375.
[890]王朝才，刘燕，马永成，等.西宁市 MSM 人群艾滋病相关知识及高风险性行为方式认

知情况调查[J].医学动物防制，2013，29(03):237-240.
[891]吴建英，汪春翔，马韶辉，等.西宁特定人群 2009~2011 年 HIV 抗体检测结果分析[J].现代预防医学，2013，40(20):3842-3844.
[892]胥加耕，梅志锋，袁中行.盐都区 2012 年艾滋病哨点人群 HIV、梅毒及 HCV 监测结果[J].国际检验医学杂志，2013，34(17):2277-2278.
[893]赵敏，王夏，邱红艳，等.一站式服务模式对 MSM 中 HIV/AIDS 病人随访与治疗依从性的影响[J].中国艾滋病性病，2013，19(08):574-579.
[894]王早霞，杜立毅，陈宏平.宜昌市男男性行为人群行为特征与艾滋病/性病感染现状分析[J].公共卫生与预防医学，2013，24(03):74-76.
[895]常进锋，陆卫群.已婚男同性恋者婚姻关系的社会学分析[J].中国性科学，2013，22(07):97-100.
[896]王毅，李六林，樊静，等.有同性固定性伴侣男男性行为者非专一性行为的影响因素研究[J].中华疾病控制杂志，2013，17(12):1025-1029.
[897]张国强，赵俊仕，彭瑾瑜，等.长沙市男男性接触人群 HIV 和梅毒感染横断面调查[J].中国病毒病杂志，2013，3(02):128-130.
[898]黄竹林，单飞，晏瑞琳，等.长沙市男男性行为人群艾滋病相关高危行为及影响因素分析[J].实用预防医学，2013，20(12):1435-1438.
[899]黄竹林，单飞，李叶兰.长沙市男男性行为者人群性行为特征分析[J].医学动物防制，2013，29(02):188-189.
[900]王连华，许圆圆，翁坚，等.浙江省台州地区男男性行为人群艾滋病病毒感染及艾滋病相关危险行为横断面调查[J].疾病监测，2013，28(05):362-365.
[901]刘洁，曲波，武玉欣，等.郑州市男男性接触人群 HIV/AIDS 相关知识和行为的调查分析[J].实用预防医学，2013，20(10):1156-1158.
[902]孙晓舒，王修晓.知晓感染 HIV 前后 MSM 人群不安全性行为改变研究[J].医学与哲学(A)，2013，34(12):39-40.
[903]安明晖，韩晓旭，刘静，等.中国部分地区 HIV 阳性男男性行为者合并 HCV 感染状况及其自然清除率调查[J].中华流行病学杂志，2013，34(01):15-18.
[904]邱英鹏，刘爱忠，冯铁建.中国大陆 MSM 人群 HIV/梅毒感染状况性行为特征和艾滋病知识知晓情况的 Meta 分析[J].中国艾滋病性病，2013，19(03):169-173.
[905]陈伟.中国男男同性恋艾滋病干预与管理进展[J].中国农村卫生，2013(z1):305-306.
[906]杨中荣，金玫华，董正全，等.中国内地大、中学生中男男性行为者 HIV 感染率的 Meta 分析[J].卫生研究，2013，42(04):689-692.
[907]杨中荣，董正全，金玫华，等.中国内地学生男男性行为者梅毒患病率 Meta 分析[J].中国公共卫生，2013，29(11):1697-1699.
[908]唐日新，孙艳芳.中国心理学中同性恋研究综述[J].江西师范大学学报(哲学社会科学版)，2013，46(05):49-55.
[909]钟珣，钟晓妮，彭斌，等.重庆男男性行为者暴露前药物预防 HIV 感染接受意愿及其影响因素研究[J].重庆医科大学学报，2013，38(04):389-394.
[910]冯连贵，丁贤彬，卢戎戎，等.重庆市男男性行为人群 HIV-1 阳转率随访研究[J].热带医学杂志，2013，13(03):342-344.
[911]赵希友，陈克江，陈亮，等.重庆市永川区男男性行为人群 HIV 感染率及其影响因素分析[J].现代预防医学，2013，40(03):500-503.
[912]陈江鹏，王宏，刘靓.重庆市在校大学生男男性行为者生存质量现状及其与社会支持的关系[J].中华流行病学杂志，2013，34(09):888-892.

[913]叶浩森，郭红革，李忠明.珠海同志互联网社区 297 名男男性行为人群行为特征分析及HIV/梅毒流行状况[J].皮肤性病诊疗学杂志，2014，21(02):151-154.
[914]龙其穗，林鹏，李艳，等.珠三角地区 2009-2013 年男男性行为者 HIV 感染率及其影响因素分析[J].中华流行病学杂志，2014，35(11):1227-1230.
[915]李秀菊，施建春，崔士磊，等.1432 例 MSM 的行为特征及 HIV 感染的影响因素分析[J].中国艾滋病性病，2014，20(10):751-754.
[916]张丹丹，洪航.2008-2012 年宁波市 MSM 人群艾滋病知识知晓情况、性行为特征和 HIV/梅毒感染状况调查[J].中华疾病控制杂志，2014，18(12):1154-1156.
[917]位晓东，隋丹，尹晔.2009-2011 年鞍山市男男性行为人群 HIV 感染情况及其影响因素[J].实用预防医学，2014，21(05):565-566.
[918]赵志杰，万悦竹，周玲，等.2009-2012 年辽宁省大连市男男性行为者艾滋病哨点监测分析[J].疾病监测，2014，29(01):41-43.
[919]吕金伟，杨康生，程周祥，等.2010-2012 年芜湖市男男性行为者人群艾滋病血清学及行为学调查分析[J].中华疾病控制杂志，2014，18(12):1147-1149.
[920]文小青.2010—2013 年桂林市男男性行为者人群艾滋病健康教育及干预效果评价[J].职业与健康，2014，30(22):3241-3243.
[921]孙修福，胡锦流，张兆辉，等.2010—2013 年淮安市 MSM 人群艾滋病哨点监测结果[J].职业与健康，2014，30(15):2089-2091.
[922]吴琼海，林海江，许圆圆，等.2010-2013 年台州市男男性行为者艾滋病监测分析[J].中华疾病控制杂志，2014，18(12):1140-1142.
[923]付金翠，李世福，刘小春，等.2010-2013 年玉溪市男男性接触者 HIV 感染及相关危险行为探讨[J].现代预防医学，2014，41(23):4370-4374.
[924]陈志娟，陈晓明，黄丽花，等.2010-2013 年云南省大理市男男性行为人群艾滋病性病哨点监测结果分析[J].卫生软科学，2014，28(12):805-808.
[925]徐洪吕，保武生，何俊，等.2010—2013 年云南省曲靖市男男性行为人群艾滋病哨点监测分析[J].疾病监测，2014，29(11):897-900.
[926]欧阳琳，吴国辉，周颖，等.2010-2013 年重庆市部分男男性行为人群认知艾滋病和感染状况分析[J].疾病监测，2014，29(02):124-129.
[927]常铃，张维义，端琼丽，等.2011～2012 年红河州 911 名男男性行为者艾滋病相关知识、行为调查[J].预防医学论坛，2014，20(04):241-243.
[928]胡跃强，唐慧玲.2011-2012 年金华市男男性接触人群高危行为和艾滋病感染情况调查[J].中国农村卫生事业管理，2014，34(08):951-953.
[929]常铃，张维义，端琼丽，等.2011-2012 年云南省红河州男男性行为者艾滋病监测分析[J].疾病监测，2014，29(02):101-104.
[930]李娟，周建，袁飞，等.2011-2013 年贵阳市男男性行为哨点监测结果[J].江苏预防医学，2014，25(03):52-54.
[931]胡锦流，孙修福，张兆辉，等.2011-2013 年淮安市男男性行为人群艾滋病知识及相关行为分析[J].江苏预防医学，2014，25(06):98-99.
[932]胡跃强.2012-2013 年金华市男男性接触者艾滋病、梅毒和丙肝感染状况调查[J].中国农村卫生事业管理，2014，34(12):1493-1495.
[933]葛锐，罗建勇，尹宗婷，等.2012 年嘉兴市男男性接触者艾滋病知识及高危行为调查[J].中国农村卫生事业管理，2014，34(07):834-836.
[934]吴纪民，周金玲，周丹，等.2012 年辽宁省 MSM 的 HIV 感染现状及影响因素[J].中国艾滋病性病，2014，20(1):27-29,43.

[935]梁莉，张静瑜，刘莉，等.2012 年四川省男男性行为人群 HIV 感染水平及其影响因素[J].中华预防医学杂志，2014，48(11):980-984.
[936]田志伟，赵淑娟，牛留业，等.2012 年许昌市男男性行为人群艾滋病性病高危行为及影响因素研究[J].现代预防医学，2014，41(13):2413-2416.
[937]李宁，戴建英，高静儒.2012 中国同性恋调查报告及对同性婚姻合法化的思考[J].中国性科学，2014，23(1):92-100.
[938]丁伟，张越新.2013 年青州市男男性行为人群艾滋病知识及 HIV/梅毒感染情况调查[J].预防医学论坛，2014，20(02):112-114.
[939]欧阳雪，郑惠能，马桂林，等.2013 年厦门市男男性行为人群艾滋病哨点监测结果分析[J].疾病监测与控制，2014，8(06):347-348.
[940]梅林，车晓文，赵启玉，等.2013 年太原地区男男性行为人群行为特征和 HIV/STI 感染情况的调查[J].疾病监测，2014，29(10):776-781.
[941]倪明健，胡晓敏，李志军，等.2013 年新疆维吾尔自治区男男性行为人群个人和社会身份认同状况及其与性相关行为的关系[J].中华预防医学杂志，2014(11):974-979.
[942]梁焕益，姜宜海.852 例男同性恋者艾滋病相关知识、高危行为与感染状况调查[J].宜春学院学报，2014，36(06):70-71.
[943]李真，绳宇.HIV 阳性的 MSM 与有偿献血者感知歧视的比较分析[J].中国艾滋病性病，2014，20(12):948-949.
[944]吴小刚，李建军，管文辉，等.HIV 阳性结果告知对男男性行为者高危性行为的影响[J].江苏预防医学，2014，25(06):12-14.
[945]王毅，李六林，张光贵，等.MSM 互联网活动平台及性文化影像对艾滋病相关行为的影响[J].中国艾滋病性病，2014，20(03):196-201.
[946]刘艳，杨国莉，龚环宇，等.MSM 人群中 HIV 感染者歧视感与心理状况及性行为的相关性研究[J].中国现代医学杂志，2014，24(06):51-55.
[947]王毅，李六林，樊静，等.MSM 同性偶然性行为及影响因素调查[J].中国艾滋病性病，2014，20(01):33-36.
[948]马瞧勤，潘晓红，周欣，等.MSM 中 HIV 快速检测实际利用情况及需求调查[J].中国艾滋病性病，2014，20(05):356-359.
[949]丁红玲，陈果，周玲.安康市男男性行为人群 HIV 感染状况及高危行为调查分析[J].医学动物防制，2014，30(03):266-268.
[950]马骥雄，杨超，刘志琦.白银市男男性行为人群行为特征及艾滋病流行趋势分析[J].中华疾病控制杂志，2014，18(12):1223-1225.
[951]孙燕鸣，岳海，范思雄，等.北京地区 MSM 人群入圈地点及相关特点初探[J].现代预防医学，2014，41(05):927-929.
[952]许元龙，杨仙珊，刘凯燕，等.北京男男性接触人群性传播感染 HIV/HBV 现状及相关因素研究[J].首都医科大学学报，2014，35(01):96-100.
[953]张海艳，徐文彩，赵楠，等.北京男男性行为者 HIV 抗体唾液快检检测试剂使用情况及影响因素研究[J].国际病毒学杂志，2014，21(01):16-21.
[954]贺淑芳，孙燕鸣，李桂英，等.北京市 2009-2013 年 MSM 艾滋病哨点监测结果分析[J].中国艾滋病性病，2014，20(08):593-596.
[955]贺淑芳，孙燕鸣，李桂英，等.北京市 2009-2013 年男男性接触者人群 HIV 感染与影响因素分析[J].中国预防医学杂志，2014，15(05):161-164.
[956]陈强，李桂英，孙燕鸣，等.北京市 2012 年男男性行为者 HIV 及梅毒感染状况调查[J].中华皮肤科杂志，2014，47(05):349-350.

[957]李洋，曾吉，孙燕鸣，等.北京市 HIV 阳性男男性接触者人群梅毒感染情况及相关因素分析[J].中国预防医学杂志，2014，15(08):745-748.
[958]任胜芳，严海波，于伟，等.不同干预模式下男男性行为者艾滋病知识知晓率和安全套使用影响因素[J].国际流行病学传染病学杂志，2014，41(06):399-402.
[959]刘莹，舒彬，刘小敏，等.不同文化程度男男性行为者艾滋病高危行为影响的研究[J].预防医学情报杂志，2014，30(03):209-212.
[960]鹿茸，何勤英，范双凤，等.成都市男男性行为艾滋病哨点监测分析[J].预防医学情报杂志，2014，30(09):715-718.
[961]李迪，彭琳，何勤英，等.成都市男男性行为人群口交时安全套使用影响因素分析[J].预防医学情报杂志，2014，30(11):945-948.
[962]刘博，罗丹，黄才苡，等.大学生男男性接触者性取向及危险性性行为调查[J].中国临床心理学杂志，2014，22(04):652-657.
[963]黄才苡，罗丹，刘博，等.大学生男男性行为者自愿咨询检测服务利用情况及影响因素调查研究[J].中国全科医学，2014，17(35):4220-4224.
[964]陈柏芬，朱建琼，张巧利，等.东莞市 2010-2012 年 MSM 人群艾滋病哨点监测结果分析[J].实用预防医学，2014，21(04):426-428.
[965]李健，余爱玲，杨常敏，等.甘肃省在婚男男性行为者 HIV 感染状况及其相关行为分析[J].中华流行病学杂志，2014，35(07):809-811.
[966]付笑冰，林鹏，王晔，等.广东省 2009-2013 年男男性接触者艾滋病哨点监测艾滋病病毒/梅毒感染趋势分析[J].中国预防医学杂志，2014，15(05):169-172.
[967]蓝光华，陈怡，张鸿满，等.广西同性恋浴室 MSMHIV 感染影响因素分析[J].预防医学情报杂志，2014，30(02):87-89.
[968]钟斐，徐慧芳，秦发举，等.广州市 2000-2010 年男男性行为人群艾滋病流行状况分析[J].热带医学杂志，2014，14(03):353-357.
[969]覃春伟，李晓华，覃雄林，等.贵港市 264 名男男同性恋 HIV/梅毒感染状况及影响因素分析[J].应用预防医学，2014，20(04):206-208.
[970]常进锋，陆卫群.贵阳市大学生男男性接触人群艾滋病知信行调查[J].中国学校卫生，2014，35(04):500-502.
[971]马璐，周健，袁飞，等.贵阳市男男性行为者 HIV 感染/梅毒感染状况及梅毒感染相关危险因素分析[J].中国皮肤性病学杂志，2014，28(01):46-49.
[972]金丹丹，许珂，罗文杰，等.杭州市男男性接触人群梅毒感染现状分析[J].中国预防医学杂志，2014，15(02):121-123.
[973]陈跃，孙修福，胡锦流，等.淮安市 MSM 人群艾滋病知识、行为及感染状况[J].江苏卫生保健，2014，16(01):1-2.
[974]孙修福，胡锦流，张兆辉，等.淮安市男男性行为人群安全套使用及影响因素[J].公共卫生与预防医学，2014，25(03):88-90.
[975]金燕，李建卓，胡艳霞，等.济南市男男性行为者艾滋病感染状况及其危险因素调查[J].疾病监测，2014，29(08):638-642.
[976]石国政，殷方兰，张永，等.嘉定区男男性接触者抑郁状况及相关因素研究[J].中国初级卫生保健，2014，28(04):65-67.
[977]胡海洋，周良佳，张之，等.江苏省男男性行为者 HIV 新发感染状况及影响因素研究[J].中华疾病控制杂志，2014，18(02):93-96.
[978]付俊，龚正华，廖清华.江西省 2013 年男男性行为人群艾滋病感染率及相关高危行为现况分析[J].江西医药，2014，49(10):969-972.

[979]何林，潘晓红，杨介者，等.接受抗病毒治疗的男男性行为者高危行为研究进展[J].中华流行病学杂志，2014，35(12):1404-1406.
[980]张涛，唐慧玲，路文雯，等.金华市 2012 年男男性接触人群哨点监测结果[J].浙江预防医学，2014，26(03):286-287.
[981]唐慧玲，胡跃强，王凤英.金华市 MSM 队列 HIV 阳转及高危行为变化情况[J].中国艾滋病性病，2014，20(10):763-765.
[982]宗金莎，李雪平.近 10 年来我国女同性恋研究述评[J].中国健康心理学杂志，2014，22(03):470-473.
[983]唐义莲，何峰宁，刘军，等.九江市男男同性恋人群行为特征与 HIV 感染率调查分析[J].华南预防医学，2014，40(02):161-163.
[984]孙炀明，贾曼红，马艳玲，等.昆明男男性行为者早期性经历对 HIV 感染影响[J].中国公共卫生，2014，30(05):677-678.
[985]张婉筠，李丽，李婕，等.昆明市 2527 例 MSM 的 HIV 感染状况及影响因素分析[J].中国艾滋病性病，2014，20(10):755-758.
[986]陈继军，王宇红，牛静萍，等.兰州市 2006-2013 年男男性接触人群艾滋病流行病学调查研究[J].中国预防医学杂志，2014，15(07):649-653.
[987]陶连弟，高宜，史磊.兰州市城关区 801 名男男性接触人群艾滋病和梅毒流行病学调查分析[J].疾病预防控制通报，2014，29(03):10-12.
[988]董正全，杨中荣，金玫华，等.利用互联网对男男性接触者高危性行为干预效果评估[J].中国预防医学杂志，2014，15(05):226-229.
[989]刘连青，李平，李季.利用网络开展 MSM 人群预防艾滋病行为干预效果评价[J].中华疾病控制杂志，2014，18(12):1232-1234.
[990]周晓红，严军，费云华，等.涟水县 169 例 MSM 人群 HIV、梅毒、丙肝感染状况调查[J].江苏卫生保健，2014，16(01):22-23.
[991]秦其荣，冯松，张茜，等.马鞍山市 MSM 人群艾滋病 KABP 调查分析[J].中华疾病控制杂志，2014，18(07):621-624.
[992]王毅，李六林，樊静，等.绵阳市 2012 年 MSM 人群 HIV/梅毒感染现状及影响因素分析[J].中国热带医学，2014，14(09):1063-1066.
[993]王毅，李六林，樊静，等.绵阳市 MSM 艾滋病知识和行为及生物学检测结果分析[J].职业与健康，2014，30(23):3394-3397.
[994]王毅，李六林，徐杰，等.绵阳市 MSM 队列研究梅毒新发感染及影响因素[J].中国公共卫生，2014，30(10):1250-1255.
[995]王毅，李六林，张光贵，等.绵阳市男男性行为人群前瞻性队列保持及其影响因素[J].职业与健康，2014，30(24):3538-3542.
[996]王毅，李六林，张光贵，等.绵阳市男男性行为者买性商业性行为影响因素研究[J].预防医学情报杂志，2014，30(12):979-983.
[997]杨珺，朱晨迪，马进，等.男男性接触者心理健康及其与高危性行为关联的研究进展[J].上海交通大学学报(医学版)，2014，34(09):1406-1410.
[998]赵丽英，赵楠，张海艳.男男性行为人群艾滋病及梅毒感染状况调查[J].中国预防医学杂志，2014，15(03):253-255.
[999]陈江鹏，王宏，廖紫珺，等.男男性行为人群生存质量及影响因素分析[J].卫生研究，2014，43(01):54-57.
[1000]符学师.男男性行为人群行为特征的流行病学分析[J].广东医学，2014，35(13):2123-2124.
[1001]孙扬，周令.男男性行为人群中 HIV 流行及其危险因素与干预研究进展[J].现代预防医

学，2014，41(16):2977-2979.
[1002]白雪，罗思童，王晓东，等.男男性行为人群中新近感染 HIV 者确诊前后性行为变化分析[J].中华流行病学杂志，2014，35(05):489-493.
[1003]王毅，黄明乾，李六林，等.男男性行为者艾森克人格问卷测试结果分析[J].中国热带医学，2014，14(12):1453-1455.
[1004]王毅，李六林，张光贵，等.男男性行为者艾滋病感染危险认知及影响因素分析[J].实用预防医学，2014，21(12):1416-1419.
[1005]王毅，李六林，张光贵，等.男男性行为者艾滋病相关认知态度与行为分离现状和影响因素研究[J].中华疾病控制杂志，2014，18(10):951-955.
[1006]王毅，李六林，张光贵，等.男男性行为者艾滋病知识与行为分离的影响因素分析[J].中华行为医学与脑科学杂志，2014，23(08):732-736.
[1007]王毅，李六林，张光贵，等.男男性行为者不同性角色 HIV/梅毒感染及与相关行为特征的关系研究[J].中国病毒病杂志，2014，4(01):43-48.
[1008]姜婷婷，蔡高峰，潘晓红，等.男男性行为者高危性行为社会心理影响因素研究进展[J].中华流行病学杂志，2014，35(10):1177-1180.
[1009]王毅，李六林，樊静，等.男男性行为者家庭社会关系及相关行为特征[J].预防医学情报杂志，2014，30(08):589-593.
[1010]陈江鹏，王宏，韩明明，等.男男性行为者生存质量及其影响因素的个人深入访谈研究[J].现代预防医学，2014，41(06):1057-1059.
[1011]陈江鹏，王宏，熊伟翔.男男性行为者生存质量及与社会支持相关性[J].中国公共卫生，2014，30(04):496-499.
[1012]王毅，李六林，张光贵，等.男男性行为者同性固定性伴侣及维持时间影响因素分析[J].现代预防医学，2014，41(01):72-75.
[1013]王毅，李六林，樊静，等.男男性行为者同性偶然性伴数及影响因素[J].中国公共卫生，2014，30(03):332-336.
[1014]王毅，李六林，张光贵，等.男男性行为者性伴和性行为特征及安全意识调查[J].预防医学情报杂志，2014，30(01):17-20.
[1015]赵金星，赵哲，杜桂英，等.男男与女女两种同性性行为人群艾滋病知识知晓率比较研究[J].国际病毒学杂志，2014，21(01):5-8.
[1016]路亮，徐丹，刘明斌，等.南昌市 MSM 人群五种常见 STD 感染状况及 HIV 感染影响因素分析[J].中华疾病控制杂志，2014，18(07):617-620.
[1017]曹越，燕虹，路亮，等.南昌市不同年龄 MSM 的特征及与社区内同伴交往状况分析[J].中国艾滋病性病，2014，20(02):84-87.
[1018]程旭，袁也丰，孙伟铭，等.南昌市男男同性恋 HIV 感染者/AIDS 患者焦虑情绪状况及其影响因素分析[J].南昌大学学报(医学版)，2014，54(07):84-86.
[1019]路亮，程旭，徐丹，等.南昌市男男性接触者人群艾滋病病毒感染者/患者生活质量及相关因素分析[J].中国预防医学杂志，2014，15(05):210-213.
[1020]曹越，肖琛嫦，李晶晶，等.南昌市男男性行为人群特征与同伴交往分析[J].公共卫生与预防医学，2014，25(04):51-54.
[1021]孙伟铭，路亮，袁也丰，等.南昌市区男男同性恋艾滋病病毒感染者/患者的焦虑抑郁情绪状况及影响因素分析[J].中华疾病控制杂志，2014，18(12):1143-1146.
[1022]陈勇，史利宁，许文炯.南京地区 289 例男男性行为人群艾滋病感染血清学调查[J].中华男科学杂志，2014，20(12):1141-1143.
[1023]吴小刚，管文辉，朱银霞，等.南京地区男-男性行为人群 HIV 感染状况及影响因素分

析[J].江苏医药，2014，40(23):2833-2836.
[1024]江秀梅，游诗建.南平市 MSM 人群性行为特征及 HIV 感染状况[J].海峡预防医学杂志，2014，20(06):19-20.
[1025]孙中华，涛波，曲琳.内蒙古自治区男男性接触者艾滋病防治所面临的挑战[J].医学动物防制，2014，30(01):35-38.
[1026]董振鑫，徐杰，张洪波，等.年轻男男性行为者梅毒新发感染状况及影响因素[J].中华预防医学杂志，2014，48(03):186-191.
[1027]李放，郑雪，麦晓浩，等.年轻男同性恋者内化同性恋嫌恶与主观幸福感的关系:自尊与孤独感的中介效应[J].心理科学，2014，37(05):1204-1211.
[1028]韦丽，贾存显，傅继华.女女性行为人群健康危险因素研究进展[J].中国公共卫生，2014，30(12):1608-1611.
[1029]张枭，林涛，孙乔，等.浦东新区新发现感染 HIV 的 MSM 的双性性行为特征分析[J].中国艾滋病性病，2014，20(11):843-844.
[1030]林兆森，傅朝阳，庞耀.钦州市男男性行为者艾滋病防治知识水平及高危行为调查分析[J].广西医学，2014，36(08):1166-1168.
[1031]廖留妹，王志刚，叶茁，等.秦皇岛地区 1077 名 MSM 艾滋病相关高危行为及 HIV 等感染状况调查[J].职业与健康，2014，30(21):3088-3090.
[1032]冯艳洁，王岚，王志刚，等.秦皇岛市 MSM 的 HIV、TP、HCV 感染状况及影响因素[J].中国艾滋病性病，2014，20(10):748-750.
[1033]孙小华，李学平，杭小平，等.庆阳市男男性行为人群 HIV/梅毒感染情况及其影响因素分析[J].中国皮肤性病学杂志，2014，28(02):165-167.
[1034]吕翠霞，张晓菲，董蕾，等.山东省部分活动场所 MSM 人群艾滋病知识态度行为调查[J].中华疾病控制杂志，2014，18(12):1226-1228.
[1035]毛军，张枭，黄星，等.上海地区男男性行为人群性取向、双性性行为及社会人口学特征[J].职业与健康，2014，30(09):1233-1235.
[1036]杨美霞，李申生，黄文鸳，等.上海市城区 MSM 的 HIV 抗体检测情况及其影响因素[J].中国艾滋病性病，2014，20(11):839-842.
[1037]顾凯侃，程华，陆晔，等.上海市静安区 MSM“扩大 HIV 检测”策略实施的可行性研究[J].中国艾滋病性病，2014，20(11):835-838.
[1038]朱晓华，沈利，尹宁，等.上海市长宁区男男性行为人群梅毒感染情况及其危险因素分析[J].中华实验和临床感染病杂志(电子版)，2014，8(04):496-499.
[1039]张欣，蔡于茂，宋亚娟.深圳市 MSM 异性性行为及其对 HIV/梅毒感染的影响[J].中国艾滋病性病，2014，20(05):360-363.
[1040]钟严伟，龙清平，黄振豪，等.深圳市龙岗区 MSM 人群艾滋病和梅毒感染状况分析[J].中国热带医学，2014，14(01):54-56.
[1041]李林涛，旷翠萍，齐杰，等.深圳市罗湖区 MSM 人群 HIV 感染状况及危险因素调查[J].中国公共卫生管理，2014，30(03):384-386.
[1042]蔡于茂，宋亚娟，洪福昌，等.深圳市男男性行为人群异性性行为特征及影响因素[J].中华疾病控制杂志，2014，18(01):32-35.
[1043]蔡于茂，刘惠，宋亚娟，等.深圳市男男性行为献血者 HIV 和梅毒感染影响因素分析[J].中华预防医学杂志，2014，48(02):128-132.
[1044]王毅，李六林，徐杰，等.四川省绵阳市男男性接触者 HIV 新发感染队列研究[J].中国病毒病杂志，2014，4(03):218-225.
[1045]傅卓华，赵秀萍，曹小平，等.苏州市男男性行为人群 HIV 新发感染队列研究[J].中国

公共卫生，2014，30(06):726-728.
[1046]张鹏，袁兆康，徐群英，等.同伴教育模式对大学生男男性行为人群影响[J].中国公共卫生，2014，30(03):263-265.
[1047]任勇，陈菊娣，胡少轶，等.网络健康管理对男同性恋艾滋病感染者的影响[J].护理研究，2014，28(30):3790-3791.
[1048]叶振淼，王大勇，赵丽娜，等.温州市主动求询男男性接触者人群 HIV 感染状况和影响因素分析[J].中国预防医学杂志，2014，15(05):180-183.
[1049]郭燕丽，王东丽，周建波，等.文化程度对男男性行为者高危性行为及 HIV、梅毒螺旋体感染率的影响[J].中华预防医学杂志，2014，48(04):307-311.
[1050]倪明健，赵小龙，李志军，等.乌鲁木齐市 MSM 的 HIV 感染状况及影响因素[J].中国艾滋病性病，2014，20(01):30-32.
[1051]朱晨，华燕，陈鑫，等.无锡市 512 名男男性行为人群艾滋病高危行为分析[J].中国热带医学，2014，14(06):692-694.
[1052]王睿，吕金伟，杨康生，等.芜湖市 422 名男男性行为者艾滋病知识行为调查[J].职业与健康，2014，30(11):1532-1533.
[1053]钟坚，郑志菊，林捷，等.梧州市男男同性恋艾滋病相关知识知晓、性行为特征及感染情况[J].中国麻风皮肤病杂志，2014，30(03):164-165.
[1054]黄晓丹，李恒新，卫晓丽，等.西安市 2007-2013 年 MSM 艾滋病哨点监测结果分析[J].中国艾滋病性病，2014，20(05):353-355.
[1055]张海兰，李恒新，闫永平，等.西安市 HIV 抗体阳性 MSM 的高危行为特征及梅毒感染状况分析[J].中国艾滋病性病，2014，20(12):929-932.
[1056]黄晓丹，卫晓丽，惠慧，等.西安市男男性行为人群艾滋病感染状况及影响因素分析[J].中华疾病控制杂志，2014，18(11):1044-1046.
[1057]王朝才，梁军，刘燕，等.西宁市男男性行为者 HIV、梅毒、丙肝感染状况分析[J].现代预防医学，2014，41(20):3786-3789.
[1058]陈慧雯，杨海云，黄春妍，等.心理护理干预对男男性接触者的效果研究[J].护理实践与研究，2014，11(10):140-142.
[1059]许圆圆，林海江.行为网络理论在男男性行为者艾滋病预防控制中的应用[J].国际流行病学传染病学杂志，2014，41(05):332-335.
[1060]杨珊，程晓松，姜聚军，等.烟台市男男性行为人群艾滋病干预效果研究[J].中华疾病控制杂志，2014，18(12):1150-1153.
[1061]俞文祥，崔倩，祁耀，等.盐城市 MSM 的 HIV 感染情况及影响因素分析[J].中国艾滋病性病，2014，20(06):458-460.
[1062]曹越，李十月，路亮，等.已婚与未婚 MSM 特征及与同伴交往状况比较[J].中国公共卫生，2014，30(10):1333-1336.
[1063]赵立华，张蕴慧，王雪敏，等.银川市 2008~2012 年男男性接触者艾滋病流行趋势及性行为特征分析[J].重庆医学，2014，43(20):2629-2631.
[1064]薛黎坚，王文明，洪志强，等.有异性性经历的男男性行为人群艾滋病传播风险研究[J].数理医药学杂志，2014，27(1):51-53.
[1065]朱艳文，胡艳霞，杨慧.与女性经常性交的未婚男男性行为者艾滋病高危性行为研究[J].现代预防医学，2014，41(11):2047-2049.
[1066]刘小春，陈良，马懿，等.玉溪市 2006-2013 年男男性接触人群 HIV 感染者/AIDS 病人流行特征分析[J].卫生软科学，2014，28(08):537-539.
[1067]杨庆伟，景正朝，苏迎盈.云南省蒙自市男男性行为人群艾滋病感染率及影响因素[J].

中国健康教育，2014，30(08):716-719.
[1068]乔俊康.运城市 MSMAIDS 病人免费抗病毒治疗的疗效分析[J].中国艾滋病性病，2014，20(06):428-430.
[1069]刘琪琛，尹少禹，路新利.张家口市男男性接触人群行为特征和疾病感染状况分析[J].医学动物防制，2014，30(05):505-507.
[1070]梅淑娟.长春市 2012 年艾滋病哨点男男性行为者监测结果[J].海峡预防医学杂志，2014，20(01):25-27.
[1071]杨介者，蒋均，陈琳，等.浙江省 2010-2013 年 MSM 艾滋病哨点监测结果分析[J].中国艾滋病性病，2014，20(12):922-925.
[1072]周娓，吴峰，赵建佳.浙江省宁波市男男性行为者艾滋病病毒检测意愿及感染状况调查[J].疾病监测，2014，29(10):772-775.
[1073]张琰，张丹丹，李怀亮，等.浙江省宁波市在婚与非在婚男男性接触者性行为特征及艾滋病感染状况比较[J].疾病监测，2014，29(06):473-476.
[1074]沈俐，茅凌翔，刘晓霞，等.镇江市 2012 年男男性行为者 HIV、HBV、HCV、梅毒感染状况分析[J].中国卫生检验杂志，2014，24(01):120-121.
[1075]刘洁，曲波，武玉欣，等.郑州市男男性接触人群无保护性行为影响因素调查[J].预防医学情报杂志，2014，30(09):687-690.
[1076]李东民，葛琳，王岚，等.中国 2010-2013 年男男性行为人群艾滋病及相关行为变化趋势分析[J].中华流行病学杂志，2014，35(05):542-546.
[1077]蓝光华.中国大陆男男性行为人群艾滋病病毒新发感染研究进展[J].应用预防医学，2014，20(03):188-189.
[1078]陈芳，丁凡，林小杰，等.中国三城市 HIV 感染者中男男性行为人群男性固定性伴告知状况及其影响因素[J].中华预防医学杂志，2014，48(11):969-973.
[1079]戴浈浈，钟晓妮，彭斌，等.中国西部地区男同性恋人群与男双性恋人群相关特征比较分析[J].第三军医大学学报，2014，36(19):2025-2029.
[1080]韩明明，王宏，陈江鹏，等.重庆市男男性行为大学生社会支持及影响因素分析[J].中国学校卫生，2014，35(02):234-236.
[1081]廖紫珺，王宏，陈江鹏，等.重庆市男男性行为人群的社会支持状况及影响因素分析[J].重庆医学，2014，43(33):4503-4506.
[1082]龙翠芳，聂建平，严易平.重庆市校外男男青少年性行为与艾滋病感染影响因素分析[J].华中科技大学学报(医学版)，2015，44(05):603-607.
[1083]伍昆山，李英毅，黄弦.珠海地区 MSM 人群 HIV/梅毒感染状况分析[J].国际检验医学杂志，2015，36(16):2312-2314.
[1084]冀乃宏，陈鹏，叶清红.株洲市男男同性人群的性行为特征分析[J].实用预防医学，2015，22(12):1483-1486.
[1085]曾玲芸，曹长安，许玉梅.100 例男男性接触者社会支持与自尊水平的对照研究[J].四川精神卫生，2015，28(01):61-63.
[1086]杨莉，杨朝军，陈会超，等.2008-2011 年云南省男男性行为人群 HIV 新发感染率调查[J].现代预防医学，2015，42(01):137-139.
[1087]隋丹丹，陈抒豪，宫艳华，等.2009-2013 年佛山市男男性行为者艾滋病哨点监测结果分析[J].华南预防医学，2015，41(01):33-36.
[1088]巩翠华，杨绪红，沈春子，等.2010～2014 年淄博市男男性行为人群艾滋病哨点监测[J].预防医学论坛，2015，21(10):786-788.
[1089]古丽萍，李莹，马斌，等.2010-2013 年兰州市 MSM 艾滋病哨点监测分析[J].中国艾滋

病性病，2015，21(07):629-631.
[1090]黄勤，李巧巧，李苑，等.2010-2013 年中国男男性行为人群艾滋病/梅毒感染状况、性行为特征及艾滋病知识知晓情况的 Meta 分析[J].中华流行病学杂志，2015，36(11):1297-1304.
[1091]王卓，龙清泉，冯雷，等.2010-2014 年达州市 MSM 艾滋病哨点监测分析[J].中国艾滋病性病，2015，21(11):957-960.
[1092]丁琳，施建春，赵涛，等.2010—2014 年南阳市男男性行为人群哨点监测结果分析[J].中国健康教育，2015，31(12):1123-1125.
[1093]周碧波，朱洁群.2010-2014 年宁波市鄞州区男男性接触人群艾滋病自愿咨询检测结果分析[J].国际流行病学传染病学杂志，2015，42(04):285-286.
[1094]韩威，周长月，张利宁，等.2010-2014 年徐州市 MSM 人群性行为特征及艾滋病感染状况分析[J].江苏预防医学，2015，26(06):84-85.
[1095]刘君辉，唐梦瑾，蒋宁，等.2010—2014 年玉林市男男性行为人群艾滋病、梅毒和丙肝监测结果分析[J].应用预防医学，2015，21(05):293-295.
[1096]赵文彬，苏秋菊，姜春红.2012—2014 年鞍山市男男性行为人群的 HIV 感染情况及其影响因素[J].职业与健康，2015，31(22):3120-3122.
[1097]王忠智.2012—2014 年大连市金州新区男男性行为人群 HIV 感染现状调查[J].中国疗养医学，2015，24(11):1220-1222.
[1098]李萍.2013 年南阳市卧龙区男男性接触者行为特征及 HIV 感染的影响因素[J].职业与健康，2015，31(01):58-60.
[1099]张秀劼，霍俊丽，崔文庆，等.2013 年云南省 VCT 门诊 MSM 人群 HIV、梅毒感染状况及相关社会人口学特征分析[J].皮肤病与性病，2015，37(05):272-274.
[1100]王金亮，郭金印，乔永青.2014 年安阳市男男性行为者艾滋病哨点监测情况[J].预防医学论坛，2015，21(05):366-367.
[1101]刘云慧，闫丽，刘小翠，等.HIV/AIDS 病人中 MSM 的心理状态与需求调查[J].中国艾滋病性病，2015，21(08):702-705.
[1102]陈芳，丁凡，林小杰，等.HIV 阳性男男性行为者的抑郁焦虑状况[J].中国心理卫生杂志，2015，29(04):251-257.
[1103]彭景舆，洪福昌，蔡于茂，等.MSM 的 HIV 感染危险因素的交互作用研究[J].中国艾滋病性病，2015，21(02):132-137.
[1104]李秀芳，韩婷婷.MSM 的尖锐湿疣发病及复发研究进展[J].中国艾滋病性病，2015，21(02):164-165.
[1105]张海波，AuWilliamW.，赵锦.MSM 交友应用程序的发展及对 MSM 危险性行为的影响[J].中国艾滋病性病，2015，21(10):912-915.
[1106]胡翼飞，范颂，李慧，等.MSM 相关心理社会因素对安全性行为影响的探讨[J].中国艾滋病性病，2015，21(06):505-508.
[1107]张鹏，林涛，赵希畅，等.MSM 性行为特征及安全套使用影响因素分析[J].中国艾滋病性病，2015，21(06):534-535.
[1108]刘云慧，闫丽，刘小翠.MSM 中 HIV/AIDS 患者心理抑郁焦虑状况调查分析[J].中国预防医学杂志，2015，16(07):578-580.
[1109]杨慧云，吴贻乐，孙业桓，等.安徽省 MSM 社群内艾滋病相关羞辱和歧视的差序格局研究[J].中华疾病控制杂志，2015，19(06):586-589.
[1110]苏秋菊，金大庆，袁月，等.鞍山市 MSM 艾滋病综合干预效果评价[J].中国艾滋病性病，2015，21(10):865-867.

[1111]刘红新，王会松，郭松洋，等.北京市昌平区 2009-2013 年男男性行为者艾滋病疫情分析[J].热带医学杂志，2015，15(10):1409-1412.
[1112]王丽娟，肖冬，丁海峰，等.北京市朝阳区 MSM 的 HIV 检测意愿及影响因素分析[J].中国艾滋病性病，2015，21(11):950-953.
[1113]魏巍，李立明，曹卫华，等.北京市海淀区男男性接触者性行为特征及 HIV 感染影响因素[J].首都公共卫生，2015，9(05):198-202.
[1114]孙燕鸣，李桂英，贺淑芳，等.北京市男男性行为者入群原因及其相关特点分析[J].中国公共卫生，2015，31(12):1530-1535.
[1115]冯艳洁，王岚，董俊善，等.不同干预方式对男男性行为者的影响比较[J].中国性科学，2015，24(04):117-123.
[1116]杜梦然，梁鹏艳，戴映雪，等.不同性伴类型的 HIV 阳性男男性行为人群心理、行为特征分析[J].中国卫生事业管理，2015，32(07):542-545.
[1117]王毅，李六林，樊静，等.不同寻找性伴场所 MSM 的人口学及艾滋病相关行为特征分析[J].预防医学情报杂志，2015，31(09):673-678.
[1118]齐金蕾，张大鹏，付晓静，等.不同招募途径的男男性行为人群梅毒感染状况及影响因素分析[J].中华预防医学杂志，2015，49(05):399-404.
[1119]周建波，黎书炜，王金塔，等.常州市 MSM 的不同性取向的行为特征[J].中国艾滋病性病，2015，21(02):127-131.
[1120]潘亚峰，王东丽，王金塔.常州市不同样本来源男男性行为者哨点监测结果分析[J].江苏预防医学，2015，26(04):33-36.
[1121]徐金玲.朝阳市 2012 年男男性行为人群 HIV 感染现状分析[J].中国公共卫生，2015，31(01):118-119.
[1122]李巧巧，曾瓈，凌东，等.成都市 MSM 人群艾滋病流行影响因素的定性分析[J].现代预防医学，2015，42(07):1319-1321.
[1123]董婷，熊华英，彭琳，等.成都市 MSM 人群不同性行为角色安全套使用影响因素分析[J].现代预防医学，2015，42(18):3346-3350.
[1124]李程，熊帮洁，陈禹存，等.大连市 MSM 中 HIV 阳性者的生活质量及其影响因素[J].中国艾滋病性病，2015，21(09):782-785.
[1125]王热勤，李江红，焦永明，等.甘肃省天水市 MSM 中 HIV 感染因素的配对研究[J].中国艾滋病性病，2015，21(10):868-870.
[1126]杨燕君，张晖，秦小洁，等.广州大学城在校大学生男同性恋者生存质量及其同性恋性身份认同情况调查[J].热带医学杂志，2015，15(03):381-384.
[1127]何婉莩，叶兴东，汤少开，等.广州市男男性接触者人群 HIV 和梅毒感染及相关因素调查研究[J].中国预防医学杂志，2015，16(09):684-688.
[1128]邵冰，宋波，杜娟，等.哈尔滨市 HIV 阳性 MSM 生存质量和社会支持状况调查[J].中国艾滋病性病，2015，21(09):777-781.
[1129]惠珊，李一，闫红梅，等.哈尔滨市男男性行为人群 HIV 新发感染及失访影响因素分析[J].中华流行病学杂志，2015，36(12):1381-1383.
[1130]马彦民，李宁，孙定勇，等.河南省 2008—2013 年男男性行为人群艾滋病流行趋势分析[J].中华流行病学杂志，2015，36(02):158-161.
[1131]彭庭海，彭国平，阳凯，等.湖北省 2010—2013 年男男性行为者 HIV 新发感染分析[J].中华流行病学杂志，2015，36(02):162-166.
[1132]杨中荣，董正全，洪清瑜，等.湖州市 2012 年 MSM 艾滋病专题调查分析[J].中国艾滋病性病，2015，21(04):335-337.

[1133]吴琼苗，程伟彬，钟斐，等.互联网预约检测的男男性行为者 HIV、梅毒感染状况及相关因素分析[J].中华流行病学杂志，2015，36(05):434-439.
[1134]黄炜，蔡光辉，朱中梁.黄石地区男男同性恋人群中梅毒新发感染状况及影响因素分析[J].中国性科学，2015，24(06):63-67.
[1135]韩秀云，韩莹，杨国樑，等.济南市男男性行为人群 HIV、TP、HSV-2 多病原感染调查结果分析[J].中国卫生检验杂志，2015，25(18):3171-3173.
[1136]杨慧，胡艳霞，金燕，等.济南市男男性行为人群艾滋病病毒感染及相关因素分析[J].中华疾病控制杂志，2015，19(12):1235-1239.
[1137]阮师漫，张昌庆，胡艳霞，等.济南市男男性行为人群梅毒监测分析[J].现代预防医学，2015，42(16):3028-3030.
[1138]赵继民，闫红梅，陆子春，等.佳木斯市男男性行为人群性伴情况调查[J].齐齐哈尔医学院学报，2015，36(22):3373-3376.
[1139]陈韦冰.健康教育及行为干预对男男性行为人群性病防治的效果调查研究[J].慢性病学杂志，2015，16(05):508-510.
[1140]张之，胡海洋，刘晓燕，等.江苏省 2011-2013 年新报告 HIV 阳性 MSM 中新发感染状况分析[J].中华疾病控制杂志，2015，19(03):249-252.
[1141]刘家虹，袁兆康，廖清华，等.江西省 2013-2014 年男男性行为人群艾滋病哨点监测结果分析[J].卫生软科学，2015，29(09):576-578.
[1142]李明炎.荆州市 2010-2014 年男男同性性行为人群艾滋病监测[J].公共卫生与预防医学，2015，26(04):95-97.
[1143]张琬悦，章任重，王珏，等.昆明市男男性行为人群 HIV 感染及其影响因素分析[J].中华疾病控制杂志，2015，19(02):203-205.
[1144]李佑芳，章任重，王珏，等.昆明市男男性行为人群无保护性肛交及其影响因素分析[J].中华疾病控制杂志，2015，19(07):743-744.
[1145]李宁，穆慧娟，礼艳霞，等.辽宁省 MSM 的社会支持现状分析[J].中国艾滋病性病，2015，21(11):954-956.
[1146]刁文丽，那军，杨晓丽，等.辽宁省 MSM 自报易发生高危性行为的场景分析[J].中国艾滋病性病，2015，21(08):699-701.
[1147]刘莉，那军，礼彦侠，等.辽宁省城市 MSM 异性婚姻意愿调查[J].中国艾滋病性病，2015，21(10):858-860.
[1148]郑禄祥，阙金财，陈前进，等.龙岩市男男性行为人群 HIV 感染与行为学监测[J].中国麻风皮肤病杂志，2015，31(01):39-41.
[1149]张秀新，薛芳辉，金镒华，等.鹿城区 2011 年-2013 年男男性行为人群 HIV、梅毒调查分析[J].中国卫生检验杂志，2015，25(07):1073-1076.
[1150]刘俊华，王光阳，胡艳萍，等.漯河市男男性接触人群中艾滋病、丙型肝炎和梅毒感染状况调查分析[J].河南预防医学杂志，2015，26(04):282-283.
[1151]王毅，李六林，樊静，等.绵阳市 2013 年男男性行为者安全套使用及影响因素分析[J].中国热带医学，2015，15(04):425-429.
[1152]王毅，黄明乾，李六林，等.绵阳市 MSM 安全套使用社会规范及影响因素[J].中国艾滋病性病，2015，21(06):509-512.
[1153]王毅，李六林，樊静，等.绵阳市 MSM 的肛交频率及影响因素[J].中国艾滋病性病，2015，21(09):786-790.
[1154]贾修伟，罗琴，王飞，等.绵阳市北川羌族自治县男男性行为者 HIV 感染及知识知晓和行为现状调查[J].实用预防医学，2015，22(04):467-469.

[1155]王毅，李六林，杨琛，等.绵阳市不同地区男男性行为者 HIV/梅毒感染及影响因素分析[J].现代预防医学，2015，42(22):4168-4171.
[1156]王毅，李六林，樊静，等.绵阳市男男性行为者对艾滋病知识的知晓率及其影响因素[J].职业与健康，2015，31(17):2369-2372.
[1157]王毅，李六林，杨琛，等.绵阳市男男性行为者浴室型性伴及影响因素分析[J].现代预防医学，2015，42(05):868-871.
[1158]李仕一，王毅，李六林，等.绵阳市县两级 MSM 人口学及 AIDS 相关行为特征和疫情现状[J].中国艾滋病性病，2015，21(02):123-126.
[1159]陈珠蝶，谢巧芳，陈杏文，等.男男同性恋 HIV 知识与高危行为状况的关系研究[J].临床护理杂志，2015，14(02):7-10.
[1160]刘洁，曲波，朱亚鑫，等.男男性接触人群生命质量及影响因素调查分析[J].中国卫生统计，2015，32(06):977-978.
[1161]胡珊，钟晓妮，陈江鹏，等.男男性接触者焦虑与抑郁症状的相关性及其与艾滋病高危性行为的关系[J].第二军医大学学报，2015，36(11):1247-1253.
[1162]倪志敏，徐丹戈，蒋鸣孝，等.男男性接触者异性性行为特征及影响因素研究[J].浙江预防医学，2015，27(01):14-16.
[1163]林兆霞，李春梅.男男性行为 HIV/AIDS 患者确诊后心理体验的研究[J].中华护理杂志，2015，50(10):1167-1170.
[1164]薛珲，刘惠，蔡凌平.男男性行为人群 HIV 暴露前预防用药的使用意愿及其影响因素[J].中华预防医学杂志，2015，49(11):973-977.
[1165]陈胜林，吴文杰.男男性行为人群 HIV 感染率及影响因素分析[J].浙江预防医学，2015，27(10):992-994.
[1166]李细苟，林培森，唐军.男男性行为人群 HIV 高危行为调查及综合干预评价[J].齐齐哈尔医学院学报，2015，36(19):2823-2824.
[1167]徐里强.男男性行为人群艾滋病防控策略的研究进展[J].中国医药指南，2015，13(21):55-56.
[1168]王晶莹，廖斌，李怡，等.男男性行为人群艾滋病高危行为干预措施研究综述[J].卫生软科学，2015，29(04):214-217.
[1169]张娟，曾梓.男男性行为人群的艾滋病流行现状及预防控制[J].职业与健康，2015，31(08):1132-1134.
[1170]吴肖冰，蓝丽娜，张春来，等.男男性行为人群梅毒预防知识知晓情况及其性行为分析[J].中国健康教育，2015，31(07):634-637.
[1171]王毅，李六林，张光贵，等.男男性行为人群人格特征与艾滋病相关因素的关系[J].中华行为医学与脑科学杂志，2015，24(08):744-747.
[1172]胡铁中，张北川，刘殿昌.男男性行为者 486 例性别认同与艾滋病高危行为的相关性分析[J].中国皮肤性病学杂志，2015，29(07):710-711.
[1173]李洮庭，付春静，钟晓妮，等.男男性行为者艾滋病暴露前预防用药性行为去抑制化情况[J].重庆医科大学学报，2015，40(06):828-833.
[1174]张昭，地力夏提·亚克甫，姜袁，等.男男性行为者艾滋病预防药物可接受性及服药方式选择的影响因素研究[J].中国性科学，2015，24(05):37-41.
[1175]王毅，李六林，樊静，等.男男性行为者的人格特征、自尊及社会支持的关系[J].中国心理卫生杂志，2015，29(06):476-480.
[1176]孟晓军，贾天剑，张轩，等.男男性行为者和性病门诊男性就诊者对人乳头瘤病毒疫苗的接受意愿及影响因素分析[J].中华流行病学杂志，2015，36(10):1119-1124.

[1177]张爱迪，夏妙娟，刘艳，等.男男性行为者健康干预的手机应用软件研究进展[J].中华流行病学杂志，2015，36(11):1315-1318.
[1178]王毅，李六林，张光贵，等.男男性行为者吸烟行为及吸烟量与艾滋病相关因素的关系[J].职业与健康，2015，31(16):2226-2229.
[1179]周娓，吴锋，龚静.男男性行为者抑郁症状及相关因素研究[J].浙江预防医学，2015，27(07):657-660.
[1180]王毅，李六林，张光贵，等.男男性行为者自尊与艾滋病相关因素的关系[J].现代预防医学，2015，42(15):2760-2763.
[1181]何凯，张晖，王玲，等.男同性恋负面身体意象的特点[J].中国性科学，2015，24(09):109-112.
[1182]管文辉，朱银霞，魏庆，等.南京市 2008-2013 年男男性行为人群早期梅毒及 HIV 感染率变化趋势分析[J].中华流行病学杂志，2015，36(06):624-628.
[1183]朱正平，张敏，刘黎，等.南京市 2013 年 MSM 的 HIV 新发感染情况分析[J].中国艾滋病性病，2015，21(01):41-43.
[1184]刘黎，张敏，朱正平，等.南京市男男性行为人群艾滋病感染情况及其影响因素[J].江苏预防医学，2015，26(02):33-35.
[1185]卢耀状，农全兴，农丽萍，等.南宁市 MSM 性行为知行分离的影响因素研究[J].中华疾病控制杂志，2015，19(12):1231-1234.
[1186]岑平，徐永芳，林新勤，等.南宁市 MSM 知识知晓情况、行为特征与艾滋病感染调查[J].中国热带医学，2015，15(11):1329-1332.
[1187]高永明，曲琳，杨景元，等.内蒙古男男性行为人群 HIV 及梅毒感染影响因素的多元非条件逐步 Logistic 回归分析[J].现代预防医学，2015，42(19):3461-3464.
[1188]李放，邢锦涛，王一博，等.年轻男同性恋者性取向隐瞒对生命意义感的影响及机制[J].中国临床心理学杂志，2015，23(06):1075-1078.
[1189]孙灵英，姜桂芳，蒋长征.宁波市江东区 2008-2013 年男男性行为人群艾滋病自愿咨询检测结果分析[J].国际流行病学传染病学杂志，2015，42(03):214-215.
[1190]赵立华，蒋岸，张蕴慧，等.宁夏男男性行为者 HIV 新发感染及影响因素分析[J].中国皮肤性病学杂志，2015，29(01):50-52.
[1191]赵书森，于海荣，杜桂英.女女性行为人群的性行为卫生防护状况及其影响因素分析[J].中国艾滋病性病，2015，21(06):533-535.
[1192]李琳，施京利，王思淼.齐齐哈尔市男男性行为人群 HIV 感染影响因素分析[J].中国卫生产业，2015，12(21):180-182.
[1193]叶枝，陈丽华，郭葳，等.歧视知觉与男同性恋艾滋病感染者创伤后应激障碍的关系:应对方式的中介作用[J].中国临床心理学杂志，2015，23(01):76-79.
[1194]廖留妹，王志刚，叶茁，等.秦皇岛市 MSM 运用自愿咨询检测服务的影响因素[J].职业与健康，2015，31(18):2561-2563.
[1195]冯艳洁，董俊善，朱小凤，等.秦皇岛市男男性行为者艾滋病相关知识行为现状及血清学检测结果分析[J].中国皮肤性病学杂志，2015，29(09):930-932.
[1196]陈雪梅，燕虹，曹越，等.青少年男男性行为人群感觉寻求状况分析[J].中国公共卫生，2015，31(12):1559-1562.
[1197]徐洪吕，保武生，何俊，等.曲靖市男男性行为人群安全套使用影响因素分析[J].中国热带医学，2015，15(01):73-75.
[1198]陈杰毅，杨育红，王增强.泉州市 2010—2013 年 MSM 哨点监测结果[J].海峡预防医学杂志，2015，21(01):25-27.

[1199]陈莲芬，唐贤龙，陈朱.三亚市不同场所男男性行为人群艾滋病流行状况[J].中国热带医学，2015，15(03):322-324.
[1200]马桂林，欧阳雪，陈娟娟，等.厦门市男男性行为人群 2010-2014 年艾滋病感染状况分析[J].中国医院统计，2015，22(01):37-39.
[1201]韦丽，贾存显，张晓菲，等.山东省女女性行为人群的特征及艾滋病相关知识调查[J].中国艾滋病性病，2015，21(03):203-205.
[1202]何欢，张洪波，丁凡，等.上海和成都市 HIV 阳性 MSM 人群性传播危险行为及其影响因素[J].中华流行病学杂志，2015，36(03):254-258.
[1203]周艳秋，顾凯侃，孙丽敏，等.上海市 50 岁以上 MSM 人群 HIV 和梅毒的感染率及影响因素[J].中国健康教育，2015，31(05):473-478.
[1204]刘春馨，潘蓉，蔡晓峰，等.上海市 HIV 抗体阴性 MSM 人群 HBsAg 和 HCVAb 阳性率及其影响因素调查[J].中华疾病控制杂志，2015，19(12):1223-1226.
[1205]张晶，潘蓉，陈坤，等.上海市 MSM 艾滋病/性病综合干预措施的效果评估[J].中国艾滋病性病，2015，21(01):44-47.
[1206]王俊，林菲菲，蔡晓峰，等.上海市男男性行为者心理健康状况调查[J].中国健康教育，2015，31(07):631-633.
[1207]尹建勋，陈红，葛梅华.上海市闸北区 MSM 高危行为及影响因素调查[J].中国艾滋病性病，2015，21(07):626-628.
[1208]李婧，龚向东，蒋宁，等.社会网络理论和方法在研究 MSMHIV 感染者中的应用进展[J].中国艾滋病性病，2015，21(01):80-83.
[1209]何慧婧，吕繁.社会文化因素对男男性行为人群艾滋病传播的影响[J].中华预防医学杂志，2015，49(11):1021-1024.
[1210]方芳，钱兵，涂玉山，等.深圳市宝安区男男性接触者艾滋病卫生服务利用情况分析[J].华南预防医学，2015，41(06):507-511.
[1211]罗青山，赵锦，陈琳，等.深圳市男男性行为人群 HIV 感染现状及影响因素分析[J].中国热带医学，2015，15(07):815-818.
[1212]王路，杨楠.沈阳市不同文化程度男男性行为者艾滋病知识、行为及感染状况[J].职业与健康，2015，31(03):339-341.
[1213]党静，刘淑君，刘丽花.石家庄市男男性接触者同性固定性伴保持时间影响因素[J].中国热带医学，2015，15(12):1448-1451.
[1214]王毅，李六林，樊静，等.四川省绵阳市 MSM 安全套携带现状及影响因素[J].中国艾滋病性病，2015，21(10):861-864.
[1215]王毅，李六林，徐杰，等.四川省绵阳市男男性行为人群队列研究 HIV 新发感染及影响因素[J].中华预防医学杂志，2015，49(01):66-70.
[1216]柏建芸，于茂河，柳忠泉，等.天津市 730 名青年学生 MSM 的 HIV 感染状况及影响因素[J].中国艾滋病性病，2015，21(12):1048-1051.
[1217]张辉，杨杰，高婕，等.天津市男男性行为人群艾滋病病毒感染情况及影响因素调查[J].现代预防医学，2015，42(14):2620-2623.
[1218]郭燕，徐鹏，徐杰，等.天津市浴池男男性接触人群人类免疫缺陷病毒感染状况及其危险因素调查[J].中华传染病杂志，2015，33(07):428-431.
[1219]董晓莹，方刚.同性恋伴侣分手暴力的质性研究[J].中国性科学，2015，24(10):106-114.
[1220]李黎明，赵必华.同性恋者的公众态度感知对孤独感的影响:出柜状况的调节作用[J].中国临床心理学杂志，2015，23(05):911-914.
[1221]韩威，童晶，张培栋，等.网络招募的男男性行为者 HIV 感染状况及影响因素分析[J].

中华实验和临床病毒学杂志，2015，29(06):519-523.
[1222]赵东设，薛芳辉，陈艳艳，等.温州市 MSM 艾滋病知识知晓率调查及行为特征[J].中国艾滋病性病，2015，21(07):600-603.
[1223]李润华，韩丽媛，潘晓红，等.我国部分地区 MSM 艾滋病健康教育效果的 Meta 分析[J].中国艾滋病性病，2015，21(09):773-776.
[1224]倪明健，王森路，胡晓敏，等.乌鲁木齐市 MSM 的性伴特征与高危性行为分析[J].中国艾滋病性病，2015，21(05):409-413.
[1225]王森路，郭妮娅，胡晓敏，等.乌鲁木齐市男男性行为者 HIV 感染状况及其影响因素[J].中国全科医学，2015，18(11):1260-1264.
[1226]季亚勇，成浩，徐梦媚.无锡市 2010-2013 年男男性行为人群艾滋病哨点监测[J].江苏预防医学，2015，26(02):43-45.
[1227]杨涛，石萍，石卫东，等.武汉市男男性行为人群艾滋病相关知识及性行为调查研究[J].医学与社会，2015，28(01):57-59.
[1228]任强，庄贵华，常文辉.西安市男男性行为人群艾滋病感染危险因素调查研究[J].中国预防医学杂志，2015，16(09):733-735.
[1229]李刚，李恒新，卫晓丽，等.西安市浴池 MSM 的 HIV 感染状况及危险因素调查[J].中国艾滋病性病，2015，21(02):162-163.
[1230]秦琴，拉巴桑珠.西藏自治区拉萨市男男性行为者基本特征[J].中国公共卫生，2015，31(12):1552-1555.
[1231]赵云岩，邱兴庆，崔德勇.襄阳市男男性行为人群 HIV 感染状况及影响因素分析[J].中国热带医学，2015，15(09):1083-1085.
[1232]黄慧敏，王棠，燕虹，等.性感觉寻求与青少年男男性行为人群危险性行为的关系[J].中华流行病学杂志，2015，36(08):821-824.
[1233]史灵恩，刘晓燕，陈禹衡，等.性活动场所与男男性行为者艾滋病高危行为关系的研究[J].现代预防医学，2015，42(21):3982-3985.
[1234]魏重政，刘文利.性少数学生心理健康与遭受校园欺凌之间关系研究[J].中国临床心理学杂志，2015，23(04):701-705.
[1235]陈江鹏，文雯，彭斌，等.应用结构方程模型分析男男性行为者生存质量影响因素的研究[J].中国卫生统计，2015，32(02):248-250.
[1236]王憓，夏时畅，潘晓红，等.应用同伴推动抽样法开展杭州市 MSM 的 HIV 感染现况及影响因素调查分析[J].中国艾滋病性病，2015，21(12):1043-1047.
[1237]汤显，张晶，潘蓉，等.与社区组织合作开展 MSM 的 HIV 抗体检测和随访管理效果分析[J].中国艾滋病性病，2015，21(01):62-64.
[1238]宋丽军，梅静远，陆继云，等.云南省 2010—2013 年男男性行为人群艾滋病相关危险因素变化趋势分析[J].中华流行病学杂志，2015，36(02):153-157.
[1239]王束秀，景正朝，丁国伟.云南省蒙自市男男性行为人群艾滋病及性病流行趋势调查[J].江苏预防医学，2015，26(05):70-72.
[1240]谭文倩，赵俊仕，刘志胜，等.长沙市 2012-2014 年男男性接触人群 HIV 感染趋势分析[J].实用预防医学，2015，22(08):912-914.
[1241]徐云，潘晓红，杨介者，等.浙江省 2004-2013 年 MSM 的艾滋病疫情分析[J].中国艾滋病性病，2015，21(01):37-40.
[1242]金洁，罗艳，陈珺芳，等.浙江省杭州市男男性行为者 HIV 感染状况及影响因素分析[J].疾病监测，2015，30(08):639-643.
[1243]董正全，杨中荣，洪清瑜，等.浙江省湖州市 2014 年男男性行为人群艾滋病专题调查

报告[J].疾病监测，2015，30(10):838-840.
[1244]唐慧玲，胡跃强，张涛.浙江省金华市男男性行为者队列基线调查 HIV 感染及影响因素分析[J].中国农村卫生事业管理，2015，35(4):477-480.
[1245]叶振淼，王大勇，薛芳辉，等.浙江省温州市男男性行为人群艾滋病流行状况及影响因素分析[J].疾病监测，2015，30(03):203-208.
[1246]刘晓霞，张明辉，李张，等.镇江市男男性行为人群梅毒阳性者诊疗转介服务及相关知识知晓情况[J].江苏预防医学，2015，26(03):92-94.
[1247]农全兴，梁长威，卢耀状，等.中国男男性行为人群 HIV 感染和新发感染相关因素的 Meta 分析[J].中国艾滋病性病，2015，21(12):1038-1042.
[1248]冯一冰，步凯，李萌，等.中国男男性行为人群 HIV 新发感染率和相关危险因素的 Meta 分析[J].中华流行病学杂志，2015，36(07):752-758.
[1249]雷云霄，李现红，王红红.中国男男性行为者高危性行为结构性影响因素及干预模式的研究现状[J].中国全科医学，2015，18(05):573-576.
[1250]胡晓松，陈芳，丁凡，等.中国三城市 HIV 阳性男男性行为人群接受随访与抗病毒治疗服务状况及其影响因素分析[J].中华预防医学杂志，2015，49(11):945-949.
[1251]陈楚莹，李雷，王曼，等.中山市 2010-2014 年男男性行为人群 HIV 感染率及相关因素[J].公共卫生与预防医学，2015，26(03):12-15.
[1252]龙翠芳，严易平.重庆市男男性行为者网络交友 HIV 感染情况[J].中国感染控制杂志，2016，15(07):461-465.
[1253]巩翠华，杨绪红，沈春子，等.淄博市男男性行为者 HIV 和梅毒感染状况及高危行为特征调查[J].中国艾滋病性病，2016，22(01):60-61.
[1254]巩翠华，沈春子，杨绪红，等.淄博市中心城区男男性行为者的 HIV 和梅毒感染状况及危险因素分析[J].中国艾滋病性病，2016，22(09):743-746.
[1255]黄巍，蔡娟丽，石卫东，等.144 例 HIV 感染者及 AIDS 患者的感知歧视状态调查[J].现代预防医学，2016，43(06):1127-1129.
[1256]陈宗良，张维，吴国辉，等.2004-2015 年重庆市男男性行为人群艾滋病流行状况分析[J].现代预防医学，2016，43(18):3277-3280.
[1257]燕纪法，吕相征，梁明修，等.2008-2014 年中国男男性行为人群艾滋病防治文献计量学分析[J].预防医学情报杂志，2016，32(03):293-301.
[1258]秦倩倩，郭巍，王丽艳，等.2008—2015 年中国经男男性行为感染 HIV 者流动情况及其影响因素[J].中华预防医学杂志，2016，50(11):938-942.
[1259]侯海燕，范炜，燕清丽，等.2010-2014 年淮安市男男性行为人群艾滋病哨点监测[J].江苏预防医学，2016，27(04):430-432.
[1260]侯海燕，范炜，燕清丽.2010-2014 年淮安市男男性行为人群安全套使用及 HIV 和梅毒感染情况[J].海南医学，2016，27(09):1518-1520.
[1261]郑艳丽，杨永利，张国昌，等.2010-2014 年平顶山市男男性行为人群 HIV 感染及流行病学特征分析[J].河南预防医学杂志，2016，27(04):270-273.
[1262]黄鹏翔，訾桂玲，廖玫珍，等.2010-2014 年山东省男男性行为者哨点监测资料分析[J].中国艾滋病性病，2016，22(09):721-724.
[1263]周彩霞，王世平，马云丽，等.2010-2014 年遵义市男男性行为人群艾滋病哨点监测结果分析[J].中国皮肤性病学杂志，2016，30(01):53-55.
[1264]程伟彬，徐慧芳，钟斐，等.2010—2015 年广州市“互联网+”艾滋病预防服务在男男性行为人群中的应用[J].中华预防医学杂志，2016，50(10):853-857.
[1265]雅雪蓉，田润芳，曹小平，等.2010—2015 年苏州市艾滋病自愿检测门诊男男性行为人

群血清学检测结果[J].江苏预防医学，2016，27(04):454-455.
[1266]卢巧玲，傅利军，杨作凯，等.2011-2013 年绍兴市男男性接触者人群 HIV 和梅毒感染状况及高危行为影响因素分析[J].中国预防医学杂志，2016，17(03):232-234.
[1267]王立华，周丽丽，黎丹丹，等.2011-2015 年广东省江门市男男性行为人群 HIV 和梅毒感染状况及影响因素分析[J].皮肤性病诊疗学杂志，2016，23(04):272-276.
[1268]蔡于茂，宋亚娟，刘惠，等.2011—2015 年深圳市男男性行为者提供商业性服务状况及影响因素调查[J].中华预防医学杂志，2016，50(11):943-948.
[1269]林丽，陈亮，郑武雄，等.2012~2013 年福州市艾滋病自愿咨询检测门诊男男性行为人群 HIV 和梅毒检测[J].预防医学论坛，2016，22(04):255-256.
[1270]梁焕益，姜宜海，戴丽萍.2012-2014 年广州男男性行为接触者 HIV 感染的 Logistic 回归分析[J].河南预防医学杂志，2016，27(03):221-225.
[1271]韩晶，汤后林，许娟，等.2012 年中国新报告男男性行为 HIV 感染者随访 1 年婚姻行为情况分析[J].中华流行病学杂志，2016，37(07):981-984.
[1272]刘宇春，刘海涛，张平花.2013 ~ 2015 年响水县 MSM 人群监测分析[J].预防医学论坛，2016，22(08):556-610.
[1273]窦明阳，李雪静.2013-2015 年长春市男男同性性行为者艾滋病监测结果分析[J].中国卫生工程学，2016，15(05):484-486.
[1274]曾吉，李洋，叶景荣，等.2013 年北京市新报告 HIV 感染者及 AIDS 患者中男男性接触者晚发现病例比例及其影响因素研究[J].中国预防医学杂志，2016，17(01):11-15.
[1275]邱兴庆，孟丽丽，谢爱齐，等.2014 年襄阳市 MSM 人群艾滋病哨点监测结果分析[J].实用预防医学，2016，23(02):201-203.
[1276]王毅，李六林，樊静，等.2015 年绵阳市男男性行为人群艾滋病哨点监测结果分析[J].现代预防医学，2016，43(12):2234-2237.
[1277]邱兴庆，龚文胜，孟丽丽，等.2015 年襄阳市 MSM 人群 HIV 感染状况及影响因素分析[J].中国初级卫生保健，2016，30(11):37-40.
[1278]杨绪红，巩翠华，沈春子，等.2015 年淄博市男男性行为者艾滋病哨点监测[J].预防医学论坛，2016，22(03):218-220.
[1279]胡桂林，李向东，曾艺，等.300 例中老年 MSM 人群艾滋病抗体检测及影响因素调查[J].重庆医学，2016，45(22):3112-3114.
[1280]张月，陈芳，丁凡，等.HIV 阳性男男性行为人群多性伴高危性行为状况及其影响因素分析[J].中华流行病学杂志，2016，37(04):517-521.
[1281]刘乃鹏，陈芳，张月，等.HIV 阳性男男性行为者对男性固定性伴性传播危险分析[J].中华疾病控制杂志，2016，20(12):1240-1243.
[1282]左培颖，陈丽华，林丹华.MSMHIV 感染者的歧视知觉与心理健康问题——自尊的调节作用[J].中国临床心理学杂志，2016，24(04):627-630.
[1283]王永.MSM 人群艾滋病、梅毒感染状况调查[J].中国卫生检验杂志，2016，26(12):1792-1794.
[1284]张枭，陈盼盼，付文捷，等.MSM 人群艾滋病相关社会环境、性伴网络、健康需求的定性分析[J].实用预防医学，2016，23(05):523-525.
[1285]张鹏，朱林英，裴启星，等.MSM 人群性行为特征与社会网络分析[J].公共卫生与预防医学，2016，27(02):108-110.
[1286]肖丹朝，郑迎军，王晓春.MSM 性病感染状况及影响因素研究[J].中国艾滋病性病，2016，22(03):185-188.
[1287]林丽，陈亮，吴韶彬.VCT 门诊男男性行为人群新报告 HIV 阳性者的行为特征[J].中国

热带医学，2016，16(11):1093-1095.
[1288]任勇，陈菊娣，杭兰生.艾滋病男同性恋病人对性安全认知的质性研究[J].护理研究，2016，30(12):1531-1532.
[1289]肖永康，程晓莉，苏斌.安徽省 2014 年男男性行为者 HIV 感染情况及其相关因素分析[J].中国艾滋病性病，2016，22(09):728-730.
[1290]段生朝，杨家芳，刘丽，等.保山市 MSM 人群 AIDS 知识调查和 HIV 抗体监测结果[J].海峡预防医学杂志，2016，22(05):31-33.
[1291]孙燕鸣，孙伟东，卢红艳，等.北京市 2005-2012 年男男性行为者同伴推动抽样法 HIV 监测分析[J].中华流行病学杂志，2016，37(10):1383-1391.
[1292]张越，张金艳，罗凤基，等.北京市 2011-2013 年男男性行为者梅毒新发感染及影响因素的前瞻性随访研究[J].中国艾滋病性病，2016，22(02):115-118.
[1293]马丽萍，福燕，郭彩萍.北京市 HIV 单阳家庭男男性行为者的生存质量及影响因素分析[J].中国艾滋病性病，2016，22(06):436-440.
[1294]夏冬艳，刘国武，曾吉，等.北京市 HIV 抗体快速检测为阳性的男男性行为人群后续确认检测行为的相关因素分析[J].中华预防医学杂志，2016，50(02):153-157.
[1295]王丽娟，陆懿斐，丁海峰，等.北京市朝阳区男男性行为者 HIV 感染状况及影响因素[J].职业与健康，2016，32(20):2839-2842.
[1296]田飞，王媛媛，马韻芳，等.北京市东城区两种男男性行为人群干预模式的干预效果评价[J].首都医科大学学报，2016，37(02):233-237.
[1297]李东亮，孙喆娅，张越，等.北京市男男性行为人群 HIV 感染情况及相关因素分析[J].中国预防医学杂志，2016，17(05):321-326.
[1298]王丽娟，武士青，张舟，等.北京市男男性行为者生存质量及其影响因素分析[J].中国艾滋病性病，2016，22(02):99-101.
[1299]吕林芳，张金艳，赵丹鹤，等.北京市男男性行为者无保护性行为影响因素研究[J].中国艾滋病性病，2016，22(10):799-802.
[1300]李瑞，柳维林，廖玫珍，等.不同活动场所男男性行为者高危行为特征及 HIV 感染因素分析[J].成都医学院学报，2016，11(05):616-620.
[1301]吴学庆，都佳，何勤英，等.成都市 MSM 人群 HIV 感染者健康服务需求及利用调查[J].预防医学情报杂志，2016，32(04):352-355.
[1302]刘伦皓，范双凤，罗映娟，等.成都市艾滋病自愿咨询检测门诊男男性行为者人群特征及 HIV 感染状况[J].实用预防医学，2016，23(10):1173-1175.
[1303]韦所苏，韦挥德，黄晓红，等.大学生男男性行为者对暴露前预防 HIV 感染的接受意愿及影响因素[J].中华行为医学与脑科学杂志，2016，25(12):1128-1132.
[1304]王继宝，罗倩倩，姚仕堂，等.德宏州男男性行为 HIV 感染者抗病毒治疗现况分析[J].上海预防医学，2016，28(12):848-852.
[1305]刘渠，谢显清，龙清平，等.对 MSM 人群艾滋病知-信-行的干预现状[J].疾病监测与控制，2016，10(09):726-727.
[1306]刘洁，曲波，朱亚鑫，等.抚顺市 MSM 人群艾滋病相关知识水平、服务利用及性行为现状调查分析[J].中国卫生统计，2016，33(02):285-286.
[1307]杨冰清，郝春，田娟，等.个人社会网络分析方法在男男性行为者高危性行为研究中的应用[J].中国卫生统计，2016，33(05):758-762.
[1308]陈梦清，程伟彬，徐慧芳，等.广东省 825 名学生男男性行为人群 rushpoppers 使用情况及其影响因素[J].中华预防医学杂志，2016，50(11):949-953.
[1309]蓝光华，沈智勇，李荣健，等.广西 MSM 人群定期应用 HIV 抗体口腔黏膜渗出液诊断

试剂的意向及影响因素分析[J].应用预防医学，2016，22(06):490-492.
[1310]叶兴东，汤少开，何婉莘，等.广州地区男男性行为者梅毒感染率及危险因素分析[J].中国艾滋病性病，2016，22(11):921-925.
[1311]何婉莘，梁容娇，庄锦填，等.广州市男男性行为人群 STD/HIV 相关高危行为特征及就诊延误影响因素调查分析[J].皮肤性病诊疗学杂志，2016，23(03):198-203.
[1312]庾泳，吴婷，李亚，等.广州市区男同性恋者性别角色与精神健康状况[J].中国健康心理学杂志，2016，24(11):1618-1622.
[1313]佘敏，李劲，周健，等.贵阳市 2010～2014 年 MSM 人群 HIV 感染者性行为情况[J].中国性科学，2016，25(11):68-72.
[1314]郑敏，鲁俊端，袁智，等.贵州省 2012-2014 年男男性行为人群监测结果分析[J].慢性病学杂志，2016，17(03):289-292.
[1315]云长缨，吴季春，周承兴.海口市 321 例男男同性恋 HIV 感染状况分析[J].中国热带医学，2016，16(07):739-740.
[1316]马彦民，李宁，朱谦，等.河南省 2015 年 MSM 人群艾滋病检测咨询情况分析[J].医药论坛杂志，2016，37(06):1-3.
[1317]叶淑君，黄冬瑞，曾梓，等.贺州市 MSM 人群艾滋病流行现状分析[J].应用预防医学，2016，22(03):216-218.
[1318]邱兴庆，赵云岩，孟丽丽，等.湖北省襄阳市男男性行为人群艾滋病干预效果分析[J].中国健康教育，2016，32(12):1082-1085.
[1319]孙修福，胡锦流，张兆辉，等.淮安市 2011—2014 年男男性行为人群艾滋病哨点监测[J].江苏预防医学，2016，27(06):690-692.
[1320]吴丽萍，孙修福，胡锦流.淮安市 MSM 中 HIV 感染者/AIDS 患者生存质量的影响因素[J].职业与健康，2016，32(10):1388-1390.
[1321]柯贤洲，熊馥，解瑞青，等.黄石市 2013—2015 年 MSM 人群艾滋病哨点监测结果分析[J].中国热带医学，2016，16(09):908-910.
[1322]柯贤洲，李安，朱丹，等.黄石市 2015 年 MSM 人群 AIDS 哨点监测结果分析[J].海峡预防医学杂志，2016，22(06):21-23.
[1323]姜袁，窦亚兰，蔡爱杰，等.基于结构方程模型构建男男性行为人群预防艾滋病知识-动机-心理模型的研究[J].中华流行病学杂志，2016，37(02):281-285.
[1324]韩莹，韩秀云，李建卓，等.济南市男男性行为人群艾滋病病毒、梅毒及丙型肝炎感染状况分析[J].中国卫生检验杂志，2016，26(13):1951-1953.
[1325]王美花，景睿，曹艳民，等.济南市男男性行为者肺结核知识知晓情况及相关特征分析[J].中国艾滋病性病，2016，22(08):648-650.
[1326]史灵恩，陈禹衡，刘晓燕，等.江苏省 2010—2014 年老年男男性行为人群特征及变化趋势分析[J].东南大学学报(医学版)，2016，35(05):742-746.
[1327]杨晴，罗雅凌，付俊，等.江西省 2011-2015 年男男性行为者艾滋病监测结果分析[J].中国艾滋病性病，2016，22(09):725-727.
[1328]肖骞，程慧，石向辉，等.聚集性干预活动对 MSM 性网络及 HIV 传播影响[J].中国公共卫生，2016，32(04):541-543.
[1329]肖骞，冯铁建，舒彬，等.聚集性干预活动对男男性行为人群性行为的影响研究[J].预防医学论坛，2016，22(06):417-421.
[1330]李佑芳，章任重，王珏，等.昆明市 HIV 抗体阳性的男男性行为者动员其性伴接受 HIV 抗体检测的可行性调查[J].中国艾滋病性病，2016，22(11):883-886.
[1331]李琪，李佑芳，章任重，等.昆明市 HIV 阳性 MSM 感染者性伴 HIV 感染及影响因素

分析[J].中国公共卫生管理，2016，32(03):294-297.
[1332]李佑芳，李琪，贾曼红，等.昆明市 HIV 阳性男男性行为者动员性伴接受 HIV 检测的意愿及影响因素分析[J].中国艾滋病性病，2016，22(08):629-632.
[1333]王玉淼，章任重，李佑芳，等.昆明市 MSM 人群兼有异性性行为特征及 HIV 感染现状分析[J].中华疾病控制杂志，2016，20(06):539-542.
[1334]王玉淼，章任重，李佑芳，等.昆明市同性恋/双性恋 MSM 人群性行为特征分析[J].中国公共卫生管理，2016，32(05):573-575.
[1335]陶桃，雷永良，邱理杨，等.丽水市 2008-2015 年 MSM 艾滋病疫情分析[J].中国公共卫生管理，2016，32(06):887-889.
[1336]吕翠霞，张晓菲，董蕾，等.利用男男性行为者活动场所开展 AIDS 高危行为干预效果评价[J].中国艾滋病性病，2016，22(01):15-17.
[1337]陈振波，潘雪梅，郑文娟，等.连州市部分男男性行为人群艾滋病/性传播疾病危险行为及影响因素[J].华南预防医学，2016，42(03):208-213.
[1338]潘雪梅，陈振波，郑文娟，等.连州市男男性行为人群抑郁及生存质量现状及其影响因素[J].江苏预防医学，2016，27(01):44-47.
[1339]马宁，阎涵，周丹，等.辽宁省艾滋病病毒阳性男男性行为人群梅毒感染状况分析[J].疾病监测，2016，31(08):659-662.
[1340]叶小红，何贤松，张震，等.临海市男男性行为 HIV 感染者干预效果评估[J].上海预防医学，2016，28(12):853-855.
[1341]王毅，李六林，张光贵，等.绵阳城区男男性行为者社会支持现状研究[J].实用预防医学，2016，23(04):399-401.
[1342]王毅，李六林，樊静，等.绵阳市 2014 年 MSM 艾滋病知识和行为特征及性行为保护性影响因素[J].实用预防医学，2016，23(09):1052-1055.
[1343]王毅，李六林，樊静，等.绵阳市 2014 年男男性行为人群 HIV 感染现状及影响因素分析[J].中国病毒病杂志，2016，6(01):40-44.
[1344]王毅，李六林，樊静，等.绵阳市男男性行为人群同伴教育覆盖现状及影响因素分析[J].华南预防医学，2016，42(02):113-118.
[1345]赵西和，杨琛，王毅，等.绵阳市男男性行为者艾滋病哨点监测结果分析[J].中国艾滋病性病，2016，22(01):18-21.
[1346]王毅，李六林，樊静，等.绵阳市男男性行为者艾滋病预防服务覆盖及影响因素分析[J].预防医学情报杂志，2016，32(04):325-330.
[1347]王毅，李六林，樊静，等.绵阳市男男性行为者接受 HIV 检测现状及其影响因素[J].职业与健康，2016，32(10):1367-1370.
[1348]王毅，李六林，樊静，等.绵阳市男男性行为者饮酒行为及与艾滋病相关因素关系[J].中国公共卫生，2016，32(07):961-964.
[1349]王毅，李六林，樊静，等.绵阳市县级 MSM 艾滋病防治网络行为干预效果[J].中国艾滋病性病，2016，22(03):176-179.
[1350]王毅，李六林，樊静，等.绵阳市县级 MSM 艾滋病知识知晓及预防服务覆盖促进效果分析[J].职业与健康，2016，32(05):663-666.
[1351]徐晓华，绳宇.男男 HIV/AIDS 患者自我歧视的影响因素研究[J].护理管理杂志，2016，16(08):533-535.
[1352]徐晓华，绳宇.男男 HIV/AIDS 患者自我歧视形成的作用模型研究[J].护理学杂志，2016，31(16):80-83.
[1353]马璐，周健，袁飞，等.男男性行为 78 例梅毒临床特征及流行病学分析[J].贵州医药，

2016，40(04):416-417.
[1354]廖光荣，杨茜，黄艳芳，等.男男性行为 HIV 感染者延续护理需求的质性研究[J].四川医学，2016，37(06):591-593.
[1355]高慧，肖芙蓉，许小珍，等.男男性行为艾滋病毒感染者梅毒感染的影响因素分析[J].中国性科学，2016，25(10):91-95.
[1356]张莉，周枫，杨雪盈，等.男男性行为大学生 HIV 感染风险自我认识及影响因素[J].中国学校卫生，2016，37(08):1140-1142.
[1357]贾天剑，潘晓雯，余炳兴，等.男男性行为门诊就诊者性行为及性传播疾病分析[J].江苏预防医学，2016，27(04):456-457.
[1358]王毅，李六林，樊静，等.男男性行为人群 HIV 检测时间距离分布情况及其影响因素[J].中华疾病控制杂志，2016，20(08):785-788.
[1359]周娓，元国平，岑焕新.男男性行为人群 HIV 抗体定期检测及其影响因素分析[J].浙江预防医学，2016，28(01):28-31.
[1360]王卫军，林兆森，吴兴华，等.男男性行为人群艾滋病高危行为强化干预效果评价[J].广西医学，2016，38(12):1718-1720.
[1361]赵东设，薛芳辉，陈艳艳，等.男男性行为人群梅毒感染及影响因素分析[J].浙江预防医学，2016，28(02):152-154.
[1362]王毅，李六林，樊静，等.男男性行为人群同伴网络大小与艾滋病相关因素的关系[J].预防医学情报杂志，2016，32(11):1140-1145.
[1363]王毅，李六林，樊静，等.男男性行为人群应对方式现状及与人口学特征的关系[J].中国病毒病杂志，2016，6(05):351-355.
[1364]王毅，李六林，樊静，等.男男性行为者社会支持与艾滋病相关因素的关系研究[J].预防医学情报杂志，2016，32(03):221-228.
[1365]刘梦驰，马保力，米国栋.男男性行为者网络调查与全国哨点监测调查的比较研究[J].中国艾滋病性病，2016，22(06):441-443.
[1366]李睿，蔡泳.男男性行为者自杀行为的现状、影响因素及自杀理论模型[J].上海交通大学学报(医学版)，2016，36(11):1676-1681.
[1367]胡珊，钟晓妮，文小焱，等.男同性恋与男双性恋 HIV 阴性者的焦虑抑郁症状特点[J].中国心理卫生杂志，2016，30(03):213-219.
[1368]路亮，徐丹，涂志斌，等.南昌市 2010-2014 年 15～24 岁男男性行为者艾滋病哨点监测结果[J].中国艾滋病性病，2016，22(06):444-447.
[1369]刘云，袁也丰，徐丹，等.南昌市 281 例男男性接触人群 HIV/AIDS 的流行病学特征分析[J].实用临床医学，2016，17(07):93-96.
[1370]徐园园，朱正平，吴苏妹，等.南京市 2011-2015 年男男性行为人群 HIV 感染率变化趋势分析[J].中华流行病学杂志，2016，37(11):1503-1508.
[1371]周良佳，闫红静，徐金水，等.南京市 MSM 人群 HIV 新发感染情况及影响因素分析[J].中华疾病控制杂志，2016，20(04):333-336.
[1372]刘黎，张敏，朱正平，等.南京市在婚男男性行为人群 HIV 感染状况及其相关行为特征调查[J].江苏预防医学，2016，27(04):413-415.
[1373]夏宏丽，马平，周小毅.南通市 2010—2015 年男男性行为人群艾滋病流行病学特征及趋势预测[J].江苏预防医学，2016，27(04):458-460.
[1374]朱洁群，周碧波，毛一斌，等.宁波鄞州地区男男性接触人群艾滋病知晓率及感染状况调查分析[J].中国性科学，2016，25(04):107-110.
[1375]杨金英.女同性恋人群性行为特征及性传播疾病知识知晓情况调查[J].中国公共卫生，

2016，32(07):917-920.
[1376]张秀云，刘明华，张淑玲，等.青岛市男男性行为人群艾滋病高危性行为调查[J].中国麻风皮肤病杂志，2016，32(01):20-21.
[1377]王平，于茂河，柳忠泉，等.青年学生男男性行为者心理健康及其与童年家庭环境关系[J].中国学校卫生，2016，37(04):523-526.
[1378]贺兴增，李锋平，刘江艺，等.泉州市2010—2014年MSM人群HIV感染及危险因素研究[J].海峡预防医学杂志，2016，22(02):13-15.
[1379]原琛利，王芳，穆生财，等.山西省2010-2014年男男性行为者艾滋病哨点监测结果分析[J].中国艾滋病性病，2016，22(08):622-625.
[1380]王泽洲，王英，蔡泳.上海市264名男男性行为者孤独感与多性伴行为研究[J].上海预防医学，2016，28(12):864-867.
[1381]张鹏，裴启星，朱林英，等.上海市376例HIV抗体阳性男男性行为者的生存质量及其影响因素研究[J].中国艾滋病性病，2016，22(10):803-805.
[1382]潘蓉，廖翠勤，张晶，等.上海市虹口区2010—2015年VCT门诊男男性行为者艾滋病流行病学特征分析[J].上海预防医学，2016，28(12):860-863.
[1383]杨瑛，张星灿，赵琬，等.上海市闵行区2005—2015年新报告HIV感染男男性行为者晚发现率及其影响因素[J].上海预防医学，2016，28(12):856-859.
[1384]田飞，王媛媛，马韵芳，等.社区小组驻点式干预模式对男男性行为人群的干预效果评价[J].中国健康教育，2016，32(06):541-543.
[1385]刘少础，杨峥嵘，陈琳，等.深圳市2014年新报告MSM中HIV/AIDS晚发现情况分析[J].中国热带医学，2016，16(08):793-795.
[1386]张海波，赵锦，WauWilliam，等.深圳市不同性角色MSM性行为特征及影响因素分析[J].中国热带医学，2016，16(05):470-474.
[1387]李晓霞，蔡文德，龙清平，等.深圳市龙岗区MSM人群异性性行为及安全套使用频率影响因素[J].公共卫生与预防医学，2016，27(04):113-115.
[1388]杨永平，李晓霞，蔡文德，等.深圳市龙岗区男男性接触者艾滋病防治知识知晓率及影响因素分析[J].华南预防医学，2016，42(05):443-446.
[1389]蔡于茂，宋亚娟，洪福昌.深圳市男男性行为者肛交多性伴行为特征及影响因素[J].中国艾滋病性病，2016，22(12):978-981.
[1390]宋亚娟，蔡于茂，洪福昌.深圳市男男性行为者婚姻状况及其对梅毒和HIV感染的影响[J].中国艾滋病性病，2016，22(10):806-809.
[1391]蔡于茂，宋亚娟，刘惠，等.深圳市男男性行为者药物滥用的影响因素[J].中国艾滋病性病，2016，22(05):361-364.
[1392]党静，刘淑君，刘丽花，等.石家庄市男男性行为人群的偶然同性性行为特征及其影响因素[J].职业与健康，2016，32(05):700-702.
[1393]荆少华，钟晓妮，雷讯，等.四川、重庆地区男男性接触者婚姻状况及其对艾滋病相关行为的影响[J].第二军医大学学报，2016，37(07):910-915.
[1394]徐放，贯长辉，吴琼海，等.台州市487名男男性行为人群艾滋病知晓率及行为调查[J].预防医学，2016，28(09):910-912.
[1395]周宁，于茂河，郭燕，等.天津市男男同性性行为人群HIV发病密度快速评估[J].实用预防医学，2016，23(02):161-165.
[1396]于茂河，江国虹，斗智，等.天津市浴池男男性行为人群HIV新发感染队列研究[J].中华流行病学杂志，2016，37(03):362-366.
[1397]李黎明，赵必华.同性恋者的公众态度感知、领悟社会支持和孤独感的关系研究[J].中

国性科学，2016，25(10):141-143.
[1398]李放，邬俊芳，麦晓浩，等.同性恋者内化同性恋嫌恶与抑郁的关系:自我概念清晰度的中介及调节作用[J].中国临床心理学杂志，2016，24(03):475-479.
[1399]朱仁敏，韩从明，汪道发，等.铜陵市男男性行为人群高危行为和性病艾滋病感染现状调查[J].中华疾病控制杂志，2016，20(12):1236-1239.
[1400]赵东设，薛芳辉，陈艳艳，等.温州市男男性行为者艾滋病病毒感染及相关因素分析[J].上海预防医学，2016，28(08):575-577.
[1401]于茂河，龚卉，郭燕，等.我国 2010-2015 年浴池男男性行为人群 HIV 感染率 Meta 分析[J].中华流行病学杂志，2016，37(08):1152-1158.
[1402]蔡爱杰，田恬，张昭，等.乌鲁木齐市 2010－2014 年男男性行为兼异性性行为者 HIV 感染状况及相关因素分析[J].中华流行病学杂志，2016，37(11):1509-1513.
[1403]王云霞，米娜瓦尔，赵小龙，等.乌鲁木齐市男男性行为者艾滋病新发感染分析[J].现代预防医学，2016，43(07):1315-1320.
[1404]高梦婷，彭民金，许亚运，等.武汉市青少年男男性行为人群非保护性行为及其影响因素[J].公共卫生与预防医学，2016，27(02):56-60.
[1405]卫晓丽，李恒新，黄晓丹，等.西安市 2013—2015 年男男性行为人群 HIV 新发感染影响因素分析[J].中国公共卫生，2016，32(12):1609-1612.
[1406]黄晓丹，李恒新，卫晓丽，等.西安市 2013-2015 年男男性行为者 HIV 新发感染情况分析[J].中国艾滋病性病，2016，22(07):525-527.
[1407]邱兴庆，张静，孟丽丽，等.襄阳市 MSM 人群艾滋病新发感染影响因素探讨[J].实用预防医学，2016，23(09):1066-1069.
[1408]姜袁，张昭，窦亚兰，等.新疆地区男男性行为人群艾滋病自愿咨询检测及安全套使用情况的调查分析[J].中国性科学，2016，25(02):148-152.
[1409]马媛媛，金涛，胡晓远，等.新疆男男性行为人群艾滋病感染状况及其影响因素分析[J].重庆医学，2016，45(21):2973-2975.
[1410]倪明健，马媛媛，陈学玲，等.新疆男男性行为人群高危行为安全套使用状况及其影响因素分析[J].中国预防医学杂志，2016，17(09):663-668.
[1411]易春霞，李蕾.新疆沙依巴克区 2015 年男男性行为者 HIV 梅毒及 HCV 监测结果分析[J].中国艾滋病性病，2016，22(09):752-753.
[1412]彭碧华，罗丹，柳英，等.新近感染艾滋病病毒的男男性行为人群的压力、情绪问题及社会支持状况[J].中国现代医学杂志，2016，26(16):131-136.
[1413]陈楚莹，王曼，汪涛，等.性角色在中山市男男性行为人群艾滋病感染及相关行为中的影响分析[J].现代预防医学，2016，43(01):99-102.
[1414]田宝伟，胡心怡.压力知觉、歧视知觉及社会支持对同性恋男大学生心理健康的影响[J].中国特殊教育，2016(12):91-96.
[1415]蔡于茂，宋亚娟，刘惠，等.药物滥用对男男性行为者高危行为及梅毒/HIV 感染的影响[J].中国艾滋病性病，2016，22(11):887-890.
[1416]陈卫建，吴文君，傅涛，等.义乌市志愿者小组管理男男性行为者中 HIV 感染者 5 年效果分析[J].中国艾滋病性病，2016，22(06):448-450.
[1417]何慧婧，吕繁，栾荣生，等.影响男男性行为人群 HIV 传播的社会文化因素定性研究[J].中华预防医学杂志，2016，50(10):858-862.
[1418]厉成梅，齐金蕾，付晓静，等.与社区组织合作在男同浴池开展 MSM 的 HIV 抗体检测促进试点[J].中国艾滋病性病，2016，22(06):460-463.
[1419]张严文，叶宝娟，郑清，等.责任性对同性恋者生活满意度的影响:有调节的中介效应[J].

中国临床心理学杂志，2016，24(05):921-924.
[1420]张国强，郑军，赵俊仕，等.长沙市男男性接触人群 HIV-1、梅毒及 HSV-2 感染状况调查[J].实用预防医学，2016，23(08):947-949.
[1421]雷云霄，王红红，肖雪玲，等.长沙市男男性行为人群 rushpoppers 使用与 HIV 感染情况及其影响因素[J].中华预防医学杂志，2016，50(02):148-152.
[1422]周建波，黄竹林，陈剑.长沙市男男性行为人群艾滋病哨点五年监测结果分析[J].中国医师杂志，2016，18(07):1047-1049.
[1423]姜婷婷，潘晓红，王憓，等.浙江省男男性行为者抑郁和焦虑状况及其相关因素分析[J].中国艾滋病性病，2016，22(05):357-360.
[1424]姚媛，吴琼海，沈伟伟，等.浙江省台州市男性同性性行为人群 HIV 检测史分析[J].中国初级卫生保健，2016，30(10):51-54.
[1425]丁坚强，易文敏，杨庆伟，等.镇海区男男性行为人群艾滋病相关知识及行为调查[J].预防医学，2016，28(08):825-826.
[1426]刘晓霞，张明辉，叶鲁，等.镇江市男男性行为人群多性伴性行为影响因素分析[J].江苏预防医学，2016，27(04):411-413.
[1427]马彦民，李宁，刘征，等.郑州市 467 名男男性行为者 HIV 和性病感染状况与流行病学特征[J].中国艾滋病性病，2016，22(08):626-628.
[1428]沈鸿程，唐松源，黄澍杰，等.中国跨性别 MSM 人群高危性行为特征及 HIV/梅毒检测研究[J].现代预防医学，2016，43(11):2052-2057.
[1429]曹越，孟详喻，翁鸿，等.中国青年男男性行为人群艾滋病相关行为及感染状况 Meta 分析[J].中华流行病学杂志，2016，37(07):1021-1027.
[1430]曾馨，钟晓妮，彭斌，等.中国西部地区多城市男男性接触者近 1 年 HIV 检测行为分析[J].第二军医大学学报，2016，37(07):827-833.
[1431]陈楚莹，王曼，汪涛，等.中山市不同性角色男男性行为人群艾滋病、梅毒感染及相关行为特征分析[J].公共卫生与预防医学，2016，27(01):53-56.
[1432]张维，吴国辉，郑建琼.重庆市 MSM 的行为特征及 HIV 感染的影响因素分析[J].现代医药卫生，2016，32(12):1822-1824.
[1433]欧阳琳，吴国辉，周颖，等.重庆市男男性行为人群艾滋病传播因素定性访谈[J].实用预防医学，2016，23(11):1348-1351.
[1434]张禧彦，赵纪伟.驻马店市男男性行为者艾滋病、梅毒、丙肝感染状况调查[J].安徽预防医学杂志，2017，23(06):406-408.
[1435]杨银梅，肖琛嫦，王棠，等.自我控制与学生男男性行为人群危险行为的关系[J].中华疾病控制杂志，2017，21(12):1241-1244.
[1436]魏艳，徐享阳，李树.遵义市 2016 年男男性行为人群现状及艾滋病行为分析[J].慢性病学杂志，2017，18(07):762-763.
[1437]宣舟斌，韩雪，商颖，等.146 名男男性行为高校学生 HIV、梅毒感染现状[J].上海预防医学，2017，29(11):863-866.
[1438]李虹璇，何海，栾荣生，等.2006-2015 年成都市成华区男男性行为人群艾滋病疫情分析[J].寄生虫病与感染性疾病，2017，15(01):21-25.
[1439]陈宗良，刘朝贵，张维，等.2006-2015 年重庆市学生男男性行为人群艾滋病流行状况分析[J].现代预防医学，2017，44(02):193-195.
[1440]史宏博，洪航，张继红，等.2006—2016 年浙江省宁波市男男性行为人群艾滋病流行特征分析[J].疾病监测，2017，32(09):735-738.
[1441]单士馨，廖玫珍，訾桂玲，等.2010-2014 年山东省网络型男男性行为者 HIV 感染及其

影响因素[J].现代预防医学，2017，44(13):2457-2461.
[1442]张翔，张万里，宋本玉，等.2010-2015 年四川省西昌市男同人群 HIV 和梅毒感染及高危行为调查[J].预防医学情报杂志，2017，33(04):301-306.
[1443]石修业，周沛林，姚秋菊，等.2010-2015 年随州市男男性行为者艾滋病综合干预失败定性研究[J].实用预防医学，2017，24(06):669-671.
[1444]吴忠兰，宋玲，鱼小红，等.2011～2015 年中国银川地区男男性行为人群 HIV-1 感染率和新发感染率趋势研究[J].病毒学报，2017，33(03):367-371.
[1445]吴苏姝，徐园园，朱正平，等.2011—2015 年南京市新报告 HIV/AIDS 病例中男男性行为者的晚发现率及其影响因素[J].职业与健康，2017，33(18):2518-2521.
[1446]张梦妍，胡婷，贾华，等.2011-2015 年陕西省男男性行为者哨点 HIV 新发感染状况及其影响因素分析[J].现代预防医学，2017，44(01):160-162.
[1447]蔡于茂，宋亚娟，刘惠，等.2011-2015 年深圳市 MSM 接受同性商业性服务状况及影响因素[J].中国艾滋病性病，2017，23(05):433-436.
[1448]胡莹，刘莉，罗映娟，等.2011-2015 年四川省男男性行为者艾滋病疫情分析[J].预防医学情报杂志，2017，33(07):642-647.
[1449]李喜英，刘征，陈彦哲.2011-2015 年郑州市男男性行为人群艾滋病哨点监测结果分析[J].现代预防医学，2017，44(16):3022-3027.
[1450]蔡于茂，宋亚娟，刘惠，等.2011—2016 年深圳市男男性行为人群梅毒和 HIV 感染情况及影响因素[J].中华预防医学杂志，2017，51(11):994-1000.
[1451]杨东智，吴忠兰，曹愍，等.2011 年-2015 年宁夏男男性行为哨点人群人类免疫缺陷病毒-1 新发感染率调查研究[J].中国卫生检验杂志，2017，27(08):1164-1165.
[1452]陶连弟，程宝莲.2013—2015 年兰州市城关区男男性接触人群哨点监测分析[J].疾病预防控制通报，2017，32(01):46-48.
[1453]刘黎，张敏，郭璐.2013—2015 年南京市老年男男性行为者 HIV 感染的影响因素[J].职业与健康，2017，33(24):3357-3360.
[1454]周艳秋，郁晓磊，吴健，等.2013 年上海市男男性行为人群新发现艾滋病病毒感染者流行病学特征对比分析[J].疾病监测，2017，32(01):25-28.
[1455]张璐平，张龙，刘德育，等.2013 年至 2015 年昆明市官渡区男男性行为人群艾滋病哨点监测结果[J].昆明医科大学学报，2017，38(04):132-136.
[1456]石朝辉，张国昌，郑艳丽.2014、2016 年平顶山市男男性行为人群艾滋病知识和行为调查[J].预防医学论坛，2017，23(03):196-198.
[1457]李晓霞，龙清平，覃佩兰，等.2014-2015 年深圳龙岗区男男性接触人群艾滋病高危行为综合干预效果评价[J].实用预防医学，2017，24(06):680-683.
[1458]袁天义，薛秀娟，贾雪娇.2014-2015 年许昌市男男同性恋者 HIV 抗体及梅毒螺旋体抗体检测结果分析[J].河南预防医学杂志，2017，28(07):525-526.
[1459]林丽，陈亮，郑武雄，等.2015 年艾滋病自愿咨询检测门诊中男男性行为人群检测结果[J].海峡预防医学杂志，2017，23(1):33-35.
[1460]刘保湘，黄道平，黎雅娟，等.2015 年常德市男男性行为人群 HIV 感染情况及相关危险因素调查分析[J].实用预防医学，2017，24(03):316-318.
[1461]周玲米，汤杰，刘岳龙，等.2015 年广西桂林市男男性行为人群艾滋病哨点监测结果分析[J].应用预防医学，2017，23(04):324-325.
[1462]王毅，李六林，樊静，等.2015 年四川省绵阳市男男性行为人群不同性行为时间其社会及性行为特征分析[J].实用预防医学，2017，24(12):1417-1420.
[1463]谢棚印，王夏，刘普林，等.2015 年武汉市 301 名男男性行为人群对 HIV 暴露前预防

用药使用意愿及相关因素[J].中华预防医学杂志，2017，51(11):1001-1006.
[1464]王毅，周万明，樊静，等.2016 年绵阳市男男性行为者艾滋病哨点监测结果分析[J].预防医学情报杂志，2017，33(12):1199-1204.
[1465]单多，吴迪，刘璐，等.2016 年天津市滥用药物男男性行为人群危险性行为状况及 HIV 感染情况[J].中华预防医学杂志，2017，51(08):718-722.
[1466]黄琳，李钰，薛妙钦，等.223 例男男性行为者艾滋病高危行为与 HIV 感染影响因素分析[J].海峡预防医学杂志，2017，23(04):16-18.
[1467]张月，陈芳，丁凡，等.3 城市感染 HIVMSM 的多性伴状况及其影响因素分析[J].中国艾滋病性病，2017，23(08):734-737.
[1468]张鹏，孙晓明，娄继权，等.CD4~+T 淋巴细胞水平与 HIV 阳性男男性行为人群生存质量关联研究[J].中华疾病控制杂志，2017，21(12):1292-1294.
[1469]陆珍珍，蓝光华，朱秋映，等.HIV-1 感染男男性行为人群婚姻的相关因素及配偶 HIV-1 状况[J].中国健康教育，2017，33(08):698-701.
[1470]刘磊，郑迎军，殷文渊，等.HIV 抗体阳性 MSM 的性病感染状况及其影响因素[J].中国艾滋病性病，2017，23(07):616-619.
[1471]郑晓星，杨蕴萍.HIV 阳性成年男男性行为者依恋类型分布研究[J].中国艾滋病性病，2017，23(02):135-137.
[1472]蔡爱杰，田恬，叶·叶克吉尔格力，等.HIV 阴性 MSM 的心理及实际性行为与艾滋病风险感知的关系[J].中国艾滋病性病，2017，23(05):440-443.
[1473]邢彦，曹卫华.MSM 两种性取向者的性行为特征比较[J].海峡预防医学杂志，2017，23(05):23-25.
[1474]段青，康殿民.MSM 人群新型毒品滥用研究进展[J].中国药物滥用防治杂志，2017，23(04):244-248.
[1475]肖智毅，王路，贺江梅，等.艾滋病自愿咨询检测门诊主动求询的男男性行为者的情况调查分析[J].中国卫生检验杂志，2017，27(02):263-265.
[1476]任仙龙，米国栋，赵燕，等.北京市男男性行为人群在检测点检测 HIV 情况及影响因素分析[J].中华预防医学杂志，2017，51(04):341-346.
[1477]王丽娟，李良，丁海峰，等.北京市男男性行为者使用社交软件与 HIV 感染高危行为相关性分析[J].职业与健康，2017，33(14):1955-1958.
[1478]黄冰雪，阿比旦·艾尼瓦尔，田恬，等.不同性角色男男性行为人群的肛周人乳头瘤病毒感染及影响因素分析[J].中华疾病控制杂志，2017，21(12):1236-1240.
[1479]杨诗凡，张嘉祺，范超楠，等.成都高校男男性行为学生参加 HIV 检测影响因素分析[J].中国学校卫生，2017，38(11):1633-1636.
[1480]代珍，何勤英，范双凤，等.成都市 MSM 中 HIV 抗体快检阳性者寻求确认检测的影响因素[J].中国艾滋病性病，2017，23(04):310-313.
[1481]范双凤，施雅莹，刘芳，等.成都市感染 HIV 的男男性行为者的高危行为及其影响因素分析[J].中国艾滋病性病，2017，23(02):126-128.
[1482]徐嘉悦，牟雨婵，马原林，等.成都市男男性行为人群艾滋病暴露前预防用药服药依从性分析[J].中华流行病学杂志，2017，38(05):643-645.
[1483]段振华，范双凤，施雅莹，等.成都市男男性行为人群既往检测与 HIV 新发感染调查分析[J].现代预防医学，2017，44(05):914-916.
[1484]郑杰滔，张晓菲，傅继华.大学生 MSM 行为学特征及其 HIV 感染状况研究进展[J].中国艾滋病性病，2017，23(06):575-577.
[1485]谢亚利，陈柏芬，罗厚菊.东莞市男男性行为人群 HIV 抗体定期检测及其影响因素[J].

中国热带医学，2017，17(12):1229-1233.
[1486]谢亚利，陈柏芬，罗厚菊，等.东莞市男男性行为人群艾滋病相关知识及性行为网络调查研究[J].中国病毒病杂志，2017，7(06):440-443.
[1487]林勋，张明雅，陈亮，等.福建省 2015 年不同来源男男性行为者知信行分析[J].海峡预防医学杂志，2017，23(01):29-31.
[1488]吴韶彬，陈亮，林丽，等.福建省 3 市 MSM 人群中 HIV 抗体阳性者的高危行为特征[J].海峡预防医学杂志，2017，23(01):15-17.
[1489]郑杰滔，张晓菲，吕翠霞，等.高校学生男男性行为特征及其高危性行为与互联网交友的关联[J].山东大学学报(医学版)，2017，55(07):115-123.
[1490]鲁桂兰，范春红，孔方，等.个案管理对男男性行为 HIV/AIDS 患者行为方式的影响[J].中国护理管理，2017，17(01):133-137.
[1491]韦所苏，谢培彦，李彧，等.广西 295 名男男性行为大学生艾滋病相关知识行为调查[J].现代预防医学，2017，44(09):1708-1713.
[1492]曾梓，刘惠，徐杰，等.广西 344 名 MSM 艾滋病非职业暴露后预防服务需求及其影响因素分析[J].中国艾滋病性病，2017，23(07):620-624.
[1493]韦所苏，韦挥德，黄晓红，等.广西男男性行为大学生艾滋病自愿咨询检测服务利用及其影响因素分析[J].中国学校卫生，2017，38(03):410-412.
[1494]李英，卢斯汉，胡荣欣，等.广州地区男男性接触者肛门人类乳头瘤病毒感染现况及危险因素分析[J].广东医学，2017，38(21):3327-3332.
[1495]何虹励，朱凯星，许美振，等.广州市 520 名男男性行为者 HIV 感染情况及影响因素分析[J].皮肤性病诊疗学杂志，2017，24(03):207-211.
[1496]靳伟，程伟彬，徐慧芳，等.广州市部分在校学生 MSM 的艾滋病危险行为特征分析[J].中国艾滋病性病，2017，23(02):123-125.
[1497]宋丽萍，张振开，耿文奎，等.桂林市男男性行为者焦虑症状现状及影响因素分析[J].中国健康教育，2017，33(12):1069-1072.
[1498]宋丽萍，张振开，张运莲，等.桂林市男男性行为者同伴教育现状及影响因素分析[J].中华疾病控制杂志，2017，21(11):1132-1135.
[1499]周欣，陈琳，何林，等.杭州市和台州市 MSM 的 HIV 快速检测需求及其影响因素[J].中国艾滋病性病，2017，23(05):387-389.
[1500]周欣，潘晓红，罗明宇，等.杭州市同性恋浴池 MSM 人群既往 HIV 检测行为相关因素分析[J].中华流行病学杂志，2017，38(11):1489-1493.
[1501]白广义，王伟，马琳，等.河北省 2010-2014 年男男性行为者 HIV 及梅毒监测结果分析[J].中国艾滋病性病，2017，23(03):236-239.
[1502]白广义，王伟，张亚丽，等.河北省三城市男男性行为者安全套使用情况及影响因素分析[J].河北医药，2017，39(21):3322-3324.
[1503]孙初阳，尹松鹤，沙丽娜，等.黑龙江省牡丹江市男男同性恋人群 HIV、梅毒螺旋体和丙型肝炎病毒感染状况分析[J].医学动物防制，2017，33(02):229-230.
[1504]丁力，王维佳，姚恩龙，等.红河州 2010-2014 年 MSM 人群艾滋病相关行为变化趋势及安全套使用影响因素分析[J].实用预防医学，2017，24(04):437-441.
[1505]金霞，肖冬，修翔飞，等.互联网+自我检测 HIV 的 MSM 特征和感染因素[J].中国艾滋病性病，2017，23(11):1043-1046.
[1506]侯海燕，邢亚东，范炜，等.淮安市男男性行为人群 HIV 感染情况及影响因素分析[J].中国健康教育，2017，33(01):21-24.
[1507]高梦婷，彭民金，许亚运，等.基于聚类分析的青少年男男性行为人群艾滋病知信行关

系研究[J].重庆医学，2017，46(02):223-225.
[1508]郭伟，孟晓军，李东民，等.吉林省 2010-2015 年男男性行为人群 HIV 哨点监测分析[J].中华流行病学杂志，2017，38(12):1655-1659.
[1509]蔡勇，乔建国，孙殿伟.吉林市青年学生 MSM 人群艾滋病行为干预效果评价[J].华中科技大学学报(医学版)，2017，46(03):346-351.
[1510]蔡勇，乔建国，孙殿伟，等.吉林市青年学生 MSM 人群高危行为调查[J].中国实验诊断学，2017，21(10):1721-1726.
[1511]周建芳，王玉霞.济南地区 160 名女女性行为者人类免疫缺陷病毒感染的流行病学特征及危险因素分析[J].中国妇幼保健，2017，32(05):1038-1041.
[1512]陈莉萍，陈禹衡，还锡萍，等.江苏省男男性行为人群 HIV 和梅毒感染状况及影响因素研究[J].中华疾病控制杂志，2017，21(12):1227-1231.
[1513]徐艳声，雷黎辉，李媛，等.昆明市(2011～2016)年 MSM 人群梅毒感染情况分析[J].皮肤病与性病，2017，39(05):346-349.
[1514]马婧，李佑芳，章任重，等.昆明市 2012－2016 年 MSM 中 HIV 新发感染队列研究[J].中国艾滋病性病，2017，23(8):755-757,763.
[1515]张秀秾，崔文庆，张小斌，等.昆明市 MSM 人群性病诊疗服务需求调查[J].皮肤病与性病，2017，39(02):116-119.
[1516]高晓娟，章任重，李佑芳，等.昆明市浴池男男性行为者的 HIV 感染状况与影响因素分析[J].中国艾滋病性病，2017，23(11):1047-1049.
[1517]刘雪梅，黎明强，韦东旭，等.柳州市男男性行为队列人群 HIV 感染影响因素分析[J].中华疾病控制杂志，2017，21(12):1232-1235.
[1518]杨晓华，马彦民，朱鑫，等.洛阳市男男性行为者新型毒品使用及其影响因素分析[J].中国艾滋病性病，2017，23(09):849-851.
[1519]王毅，李六林，樊静，等.绵阳市 MSM 艾滋病知识及不同地区肛交保护性和影响因素[J].现代预防医学，2017，44(05):872-876.
[1520]王毅，李六林，樊静，等.绵阳市 MSM 首次同性性行为发生途径及关联因素分析[J].中国艾滋病性病，2017，23(07):625-629.
[1521]王毅，李六林，樊静，等.绵阳市离异/丧偶男男性行为者人口学及性行为特征分析[J].中国热带医学，2017，17(05):480-484.
[1522]王毅，李六林，樊静，等.绵阳市男男性行为人群艾滋病感染现状及影响因素[J].预防医学情报杂志，2017，33(07):623-627.
[1523]王毅，樊静，杨晓玲，等.绵阳市男男性行为人群流动性行为及影响因素[J].中国公共卫生，2017，33(03):345-349.
[1524]王毅，李六林，樊静，等.绵阳市男男性行为人群性行为特征及多性伴影响因素分析[J].华南预防医学，2017，43(01):1-6.
[1525]王毅，李六林，樊静，等.绵阳市男男性行为人群压力感受状况及影响因素分析[J].华南预防医学，2017，43(03):201-205.
[1526]王毅，李六林，樊静，等.绵阳市男男性行为者异地性行为现状及影响因素[J].中国热带医学，2017，17(10):1012-1016.
[1527]侯海燕，范炜，燕清丽，等.某市 2013～2015 年男男性行为人群人类免疫缺陷病毒新发感染研究[J].中国现代医学杂志，2017，27(25):95-98.
[1528]刘安齐，傅琳玲，方晶，等.男男性接触者中肛肠尖锐湿疣患者合并淋球菌、沙眼衣原体感染现况[J].中国感染控制杂志，2017，16(12):1137-1140.
[1529]蓝光华，梁能秀.男男性行为人群 HIV 阳性者高危行为情况及配偶检测的影响因素[J].

应用预防医学，2017，23(05):355-358.
[1530]罗淑星，周海龙，周超，等.男男性行为人群艾滋病患者心理健康状况与性行为调查分析[J].预防医学情报杂志，2017，33(05):450-453.
[1531]卢姗，李东民.男男性行为人群无保护性肛交行为及其影响因素研究进展[J].中华流行病学杂志，2017，38(11):1584-1587.
[1532]翟飞飞.男男性行为者艾滋病综合干预效果评价与高危性影响因素分析[J].蚌埠医学院学报，2017，42(04):511-514.
[1533]陈盼盼，肖绍坦，金樱枝，等.男男性行为者定期艾滋病 VCT 行为及影响因素研究[J].中国艾滋病性病，2017，23(09):840-843.
[1534]李兰英，孙越，徐向辉，等.男男性行为者肛周及肛管尖锐湿疣复发的危险因素分析[J].中国艾滋病性病，2017，23(06):542-545.
[1535]路亮，刘明斌，刘薇，等.南昌市男男性行为人群异性性行为的发生情况[J].公共卫生与预防医学，2017，28(05):132-134.
[1536]徐园园，张敏，朱正平，等.南京市 601 名 MSM 近 1 年 HIV 检测情况及影响因素分析[J].中国艾滋病性病，2017，23(04):314-317.
[1537]徐园园，朱正平，李昕，等.南京市 MSM 无保护肛交在 Rushpoppers 使用与 HIV/梅毒感染之间的中介效应[J].中国艾滋病性病，2017，23(08):726-729.
[1538]郭璐，张敏，朱正平，等.南京市不同活动场所男男性行为者行为特征及艾滋病、梅毒感染情况[J].江苏预防医学，2017，28(03):280-283.
[1539]郭璐，张敏，刘黎，等.南京市不同性角色 MSM 的行为特征及 HIV 和梅毒感染现状[J].中国艾滋病性病，2017，23(03):232-235.
[1540]赵群，孙梦琪，王军芳.南京市大学生同性恋者与双性恋者行为现状与影响因素分析[J].中国健康教育，2017，33(12):1073-1076.
[1541]刘黎，张敏，朱正平，等.南京市某浴池男男性行为者安全套使用及影响因素调查[J].中国健康教育，2017，33(03):240-243.
[1542]朱正平，张敏，徐园园，等.南京市男男性行为人群使用新型毒品亚硝酸酯类吸入剂 rushpoppers 情况调查[J].中华流行病学杂志，2017，38(2):189-193.
[1543]魏叶，马平，夏宏丽，等.南通市男男性接触人群 HIV 感染者阳性告知前后因素分析[J].中国公共卫生，2017，33(03):369-372.
[1544]顾晓敏，史宏博，姜海波，等.宁波地区 2013-2015 年男男性接触者感染艾滋病哨点监测结果分析[J].中国公共卫生管理，2017，33(05):677-680.
[1545]张晓菲，吕翠霞，郑杰滔，等.青岛市 2016 年部分高校学生男男性行为者性伴状况研究[J].中华流行病学杂志，2017，38(12):1638-1641.
[1546]许亚运，王棠，李十月，等.青少年男男性行为者感觉寻求与肛交安全套使用[J].武汉大学学报(医学版)，2017，38(01):80-84.
[1547]张梦妍，任强，邹扬帆，等.陕西省 2012 年-2015 年新报告 HIV 阳性 MSM 的新发感染状况分析[J].中国卫生检验杂志，2017，27(22):3301-3303.
[1548]张梦妍，胡婷，任强，等.陕西省不同社会人口学特征的男男性行为者 5 年 HIV 新发感染率监测分析[J].中国卫生检验杂志，2017，27(13):1925-1927.
[1549]韩琳，高宪社，许雷涛，等.陕西渭南市 2010-2015 年 HIV 阳性 MSM 的行为特征分析[J].中国艾滋病性病，2017，23(05):437-439.
[1550]成媛媛，石梅，钱伊弘，等.上海地区男男性行为者肛门部位尖锐湿疣 HPV 型别特征及口腔部位 HPV 感染情况研究[J].中国男科学杂志，2017，31(02):22-28.
[1551]裴启星，赵希畅，周晓林.上海浦东新区 HIV 抗体阳性男男性行为者的服药依从性影

响因素[J].中国热带医学，2017，17(11):1111-1114.
[1552]潘蓉，陈坤，郑煌，等.上海市 HIV 阳性男性同性性行为者的抑郁症状及其影响因素[J].复旦学报(医学版)，2017，44(04):430-434.
[1553]刘瑛，唐海丰，宁镇，等.上海市艾滋病自愿咨询检测门诊男男性行为者 HIV 与梅毒、单纯疱疹病毒 2 型共感染状况调查[J].中华流行病学杂志，2017，38(10):1363-1366.
[1554]李睿，王英，蔡泳.上海市男男性行为人群自杀意念及其相关社会心理因素研究[J].健康教育与健康促进，2017，12(06):535-540.
[1555]宋丽萍，张振开，汤杰，等.社会支持与男男性行为者抑郁症状的关系[J].中国艾滋病性病，2017，23(03):240-243.
[1556]刘少础，赵锦，陈琳，等.深圳市 2013－2015 年 MSMHIV 新发感染状况分析[J].中国热带医学，2017，17(8):774-777.
[1557]刘莹，刘小敏，舒彬，等.深圳市不同主要活动场所男男性行为者艾滋病高危行为特点[J].热带医学杂志，2017，17(10):1402-1406.
[1558]彭小雪，李林涛，齐杰，等.深圳市男男性行为者焦虑和抑郁状况及其相关因素分析[J].中国艾滋病性病，2017，23(07):630-633.
[1559]杨艳芳，田丽闪，丁一，等.深圳市南山区 MSM 个体性网络大小对感染梅毒的影响[J].中国热带医学，2017，17(09):887-890.
[1560]毛翔，于欢，胡清海，等.沈阳市 MSM 参加 HIV 暴露前预防性用药临床试验的接受意愿调查[J].中华流行病学杂志，2017，38(08):1083-1087.
[1561]王毅，李六林，樊静，等.四川省绵阳市男男性行为者首次与近 6 个月同性性行为保护性之间的关系及内在一致性影响因素[J].中国病毒病杂志，2017，7(04):287-292.
[1562]张翼.四川省某市男男性行为人群艾滋病知识行为现状及其影响因素分析[J].中国初级卫生保健，2017，31(09):54-55.
[1563]李玲玲，张玉成，吴琼海，等.台州市不同性取向男男性行为人群特征比较分析[J].中国初级卫生保健，2017，31(08):53-55.
[1564]樊爱平，成玲，杨冬芳，等.泰安市男男性行为大学生艾滋病感染及相关行为状况[J].中国学校卫生，2017，38(06):819-822.
[1565]柳忠泉，周宁.天津市 2004-2014 年男男同性性行为感染艾滋病病例生存时间及影响因素[J].中国病毒病杂志，2017，7(02):149-153.
[1566]陈官钧.同志报道对同性恋出柜的影响研究[J].中国性科学，2017，26(07):154-157.
[1567]张曼，加沙尔·哈孜泰，王凯，等.乌鲁木齐市男男同性性行为人群艾滋病病毒感染危险因素分析及流行趋势预测[J].职业与健康，2017，33(24):3395-3398.
[1568]田恬，蔡爱杰，黄冰雪，等.乌鲁木齐市男同性恋浴池与艾滋病自愿咨询检测门诊的 MSM 感染 HPV 情况比较[J].中华流行病学杂志，2017，38(1):53-57.
[1569]钟坚，李骏，谭琳琳，等.梧州市男男性行为人群艾滋病流行现状及影响因素调查[J].中国药物滥用防治杂志，2017，23(05):264-266.
[1570]毛云霞，肖琛嫦，王棠，等.武汉市年轻男男性行为者偶遇性行为及相关因素分析[J].中华流行病学杂志，2017，38(06):746-749.
[1571]燕虹，杨银梅，肖琛嫦，等.武汉市青年学生男男性行为者性感觉寻求状况[J].中国艾滋病性病，2017，23(11):1065-1067.
[1572]张雅娟，卫晓丽，刘存，等.西安市 1985 名 MSM 艾滋病感染危险因素及高危行为变化分析[J].临床医学研究与实践，2017，2(31):98-99.
[1573]贾华，张梦妍，常文辉.西安市 MSM 人群新型毒品滥用现况及其影响因素分析[J].现代预防医学，2017，44(02):332-335.

[1574]王毅，李六林，周万明，等.心灵冲击与持续强化相结合的年轻男男性行为者艾滋病行为干预模式建立[J].职业与健康，2017，33(17):2438-2441.
[1575]蒋和宏，陈于，欧阳琳.新型毒品滥用与男男性行为者艾滋病的传播[J].重庆医学，2017，46(13):1848-1850.
[1576]魏卫.信阳市 157 例男男同性恋大学生中艾滋病感染情况调查[J].河南预防医学杂志，2017，28(05):355-357.
[1577]程晓松，林荣，姜聚军，等.烟台市男男性行为人群 HIV 感染者/AIDS 病人抗病毒治疗后高危行为变化[J].中华疾病控制杂志，2017，21(12):1209-1213.
[1578]宋丽萍，汤杰，张运莲，等.移动电话短信干预对 MSM 高危性行为的影响[J].中国艾滋病性病，2017，23(10):932-934.
[1579]金莹莹，徐杰，江震，等.应用交友软件调查大学生男男性行为者 HIV 检测现状及相关因素[J].中华流行病学杂志，2017，38(05):629-633.
[1580]杨莉，陈会超，陈敏，等.云南省 2012-2014 年某男男性行为人群检测平台 HIV-1 新发感染状况分析[J].中国皮肤性病学杂志，2017，31(11):1229-1231.
[1581]罗昊，赵俊仕，谭红专，等.长沙市高校学生男男性行为人群 HIV 检测行为及影响因素分析[J].现代预防医学，2017，44(13):2435-2438.
[1582]胡小炜，刘建宁，吴虹，等.浙江省杭州市西湖区男男性行为人群 HIV 感染情况及影响因素分析[J].疾病监测，2017，32(08):651-655.
[1583]林海江，张玉成，沈伟伟，等.浙江省台州市 2013—2015 年男男性行为者人群同性群交行为调查[J].中华流行病学杂志，2017，38(05):634-637.
[1584]李健，韩晶，许娟，等.中国 2014 年新报告男男性行为者中 HIV 感染者婚姻及配偶感染现状[J].中华流行病学杂志，2017，38(06):750-753.
[1585]庾泳，肖水源.中国广州男同性恋人群健康状况和生活满意度（英文）[J].中南大学学报(医学版)，2017，42(12):1407-1416.
[1586]卢耀状.中国男男性行为者艾滋病感染情况及干预工作进展[J].应用预防医学，2017，23(04):348-351.
[1587]庾泳，肖水源.中国社会文化背景下社会态度对同性恋人群的影响[J].中国健康心理学杂志，2017，25(03):476-479.
[1588]来学惠，李雷，陈楚莹，等.中山市 2012－2014 年 MSM 艾滋病流行特点[J].中国艾滋病性病，2017，23(3):254-257.
[1589]陈楚莹，来学惠，舒波，等.中山市男男性行为者 HIV 新发感染状况研究[J].中国艾滋病性病，2017，23(06):538-541.
[1590]欧阳琳，蒋和宏，周超，等.重庆市使用新型毒品的男男性行为者感染 HIV 的影响因素[J].中国艾滋病性病，2017，23(10):928-931.
[1591]周毅，杜琳，代文灿，等.珠海市男男性行为者利用 HIV 互联网+自我检测结果分析[J].中国艾滋病性病，2017，23(12):1128-1130.
[1592]胡桂林，汪强，李向东，等.重庆某区场所型男男性接触人群艾滋病感染状况及影响因素分析[J].现代医药卫生，2018，34(21):3283-3286.
[1593]欧阳琳，吴国辉，周超，等.重庆市有婚史的男男性行为者的 HIV 感染状况及行为心理特征分析[J].中国艾滋病性病，2018，24(04):357-360.
[1594]宋潮，董舒阳，徐鑫，等.自尊在同性恋者社会支持与孤独感关系中的中介作用[J].中国临床心理学杂志，2018，26(02):401-404.
[1595]王毅，李六林，周万明，等.15～24 岁男男性行为者的焦虑和抑郁症状及相关因素[J].中国心理卫生杂志，2018，32(12):1017-1024.

[1596]徐园园，朱正平，吴苏姝，等.2004—2016 年南京市男男性行为者中首次接受抗病毒治疗的 HIV/AIDS 病人生存分析[J].中国艾滋病性病，2018，24(07):697-701.
[1597]肖明，李一苇，霍炜.2008～2017 年菏泽市男男性行为人群艾滋病疫情分析[J].预防医学论坛，2018，24(08):612-613.
[1598]安银翠，杜玉梅，魏建军，等.2009—2016 年汉中市男男性行为人群 HIV 感染状况[J].职业与健康，2018，34(23):3246-3248.
[1599]吉克春农，裴容，杨淑娟，等.2010-2015 年凉山州艾滋病哨点 MSM 人群 HIV 感染状况及影响因素分析[J].现代预防医学，2018，45(07):1301-1304.
[1600]赵淑娟，杨杰，黄丽丽，等.2010—2016 年许昌市 MSM 人群艾滋病感染及影响因素[J].职业与健康，2018，34(23):3224-3227.
[1601]王长梅，戎毅.2010—2016 年扬州市广陵区男男性行为 HIV 感染者/AIDS 病人随访分析[J].江苏预防医学，2018，29(05):520-521.
[1602]周全华，何惊春，卢戎戎，等.2010—2016 年重庆市男男性接触人群血清学检测结果分析[J].检验医学与临床，2018，15(14):2033-2035.
[1603]李建卓，韩莹，杨慧，等.2010 年-2015 年济南市男男性行为人群哨点监测资料分析[J].中国卫生检验杂志，2018，28(08):996-1000.
[1604]李雷，曹小平，陈禹衡，等.2011—2015 年苏州市男男性行为人群 HIV 新发感染率变化趋势及影响因素[J].江苏预防医学，2018，29(05):484-487.
[1605]豆正东，方艳姣，芮蓓.2011-2015 年芜湖市男男性行为人群安全套使用状况及影响因素分析[J].中华疾病控制杂志，2018，22(03):254-257.
[1606]程晓松，薛建，林荣，等.2012-2014 年烟台市男男性行为者安全性行为变化趋势及影响因素[J].热带医学杂志，2018，18(04):543-546.
[1607]陈潇潇，乔晓彤，沈伟伟，等.2013-2016 年浙江省台州市男男性行为人群艾滋病相关危险行为特征和感染状况分析[J].疾病监测，2018，33(10):854-859.
[1608]韩莹，李建卓，潘晶，等.2013 年-2016 年男男性行为人群结果分析[J].中国卫生检验杂志，2018，28(08):1007-1009.
[1609]罗亚莉，曾甜甜.2014—2016 年兰州市城关区男男性行为者哨点监测分析[J].疾病预防控制通报，2018，33(04):48-49.
[1610]蔡月华，黄金梅，吕继荣，等.2014—2016 年连州市男男性行为人群 HIV 感染状况与危险因素监测结果[J].职业与健康，2018，34(13):1817-1820.
[1611]刘少础，杨峥嵘，赵锦，等.2015—2017 年深圳市 HIV 阳性男男性行为者的婚姻状况分析[J].中国艾滋病性病，2018，24(09):905-907.
[1612]解雁茹，张飞，聂晓勇，等.2015 年山西省男男性行为者艾滋病监测结果分析[J].中国药物与临床，2018，18(04):530-531.
[1613]刘梅艳，张宏，林春仲，等.2016、2017 年福州市男男同性性行为人群艾滋病相关知识、性行为与感染状况调查[J].预防医学论坛，2018，24(05):359-361.
[1614]张宏，陈剑惠，林春仲，等.2016 年福州市依托社会组织开展 MSM 人群艾滋病监测结果分析[J].预防医学论坛，2018，24(05):336-340.
[1615]李建卓，王春梅，张昌庆.2016 年济南市 HIV 阳性男男性行为人群性病流行状况及危险因素分析[J].预防医学论坛，2018，24(12):904-907.
[1616]王毅，李六林，樊静，等.2016 年绵阳市不同地区 MSM 人群艾滋病知识、行为及检测结果[J].职业与健康，2018，34(12):1678-1681.
[1617]郭燕，周宁.2016 年天津市男男性接触人群行为特征与性病感染率调查[J].国际病毒学杂志，2018，25(01):24-29.

[1618]罗莉，曹琳，胡荣，等.2016 年武汉市新报告男男性行为 HIV 感染者 rushpoppers 使用情况及相关因素[J].中华预防医学杂志，2018，52(12):1287-1289.
[1619]罗倩倩，陈子煌，马跃，等.2017 年北京市使用某手机社交软件的男男性行为人群感染 HIV 风险及相关因素[J].中华预防医学杂志，2018，52(12):1220-1224.
[1620]陈剑，黄竹林.2017 年长沙市 401 例男男性行为者艾滋病知识行为态度调查[J].预防医学情报杂志，2018，34(09):1172-1176.
[1621]韩明明，钟晓妮，彭斌，等.288 名学生男男性行为者 HIV 检测状况及影响因素分析[J].重庆医科大学学报，2018，43(07):980-983.
[1622]周佳梁，刘宏宇，梁玉红，等.502 名男男性接触者性行为特征及艾滋病干预影响因素调查[J].医学动物防制，2018，34(02):168-170.
[1623]李桂玲，姜红，韵霞，等.50 岁及以上男男性行为人群人类免疫缺陷病毒和梅毒感染率及其影响因素[J].中国老年学杂志，2018，38(02):447-449.
[1624]严心诚，朱志先.GSNapps 对男男性行为者性行为的影响与艾滋病防控[J].中国艾滋病性病，2018，24(02):216-219.
[1625]韩舒羽，胡雁，贾守梅，等.HIV/AIDS 男男性行为者羞辱和歧视的研究进展[J].护士进修杂志，2018，33(12):1078-1082.
[1626]刘增艳，王欣心，洪佳佳，等.HIV 阳性 MSM 人群梅毒感染状况及影响因素调查[J].现代预防医学，2018，45(03):520-523.
[1627]梅竹，王泓懿，毛翔，等.HIV 阳性男男性行为者的 Rush 使用状况及其与 HIV 高危性行为的关联性[J].中国艾滋病性病，2018，24(07):687-691.
[1628]刘乃鹏，张月，王晓冬，等.HIV 阳性男男性行为者对偶然或商业性伴的传播危险分析[J].中国艾滋病性病，2018，24(02):159-163.
[1629]姜海波，洪航，顾晓敏，等.HIV 阴性男男性行为者艾滋病相关行为特征及影响因素调查[J].中国艾滋病性病，2018，24(07):702-705.
[1630]陈菊娣，任勇，曹维宁.MSM/HIV 青年患者婚姻真实感受的质性研究[J].中华现代护理杂志，2018，24(12):1422-1425.
[1631]王毅，李六林，樊静，等.MSM 艾滋病防治信息获取意愿及不同来源效力认定的人口和行为学特征[J].中国热带医学，2018，18(01):52-56.
[1632]王念，钟晓妮，黄爱龙.MSM 人群对 PrEP 干预策略的接受意愿及影响因素研究[J].重庆医学，2018，47(32):4142-4145.
[1633]赵艳秋，曹小平，傅卓华，等.VCT 门诊在婚男男性行为人群性行为及 HIV 感染状况[J].江苏预防医学，2018，29(05):526-527.
[1634]沈月兰，程晓莉，苏斌，等.安徽省 2010—2015 年男男性行为者艾滋病流行特征分析[J].中国艾滋病性病，2018，24(09):901-904.
[1635]吴弦.巴蜀文化环境下四川省“同志”群体的情绪特征研究[J].哈尔滨工业大学学报(社会科学版)，2018，20(02):52-57.
[1636]王芳，田晓红，杨学刚，等.保定市男男性行为者中 HIV/AIDS 病人创伤后成长的影响因素[J].中国艾滋病性病，2018，24(11):1128-1131.
[1637]张德川，陈子煌，马跃，等.北京市学生男男性行为人群无保护性行为相关因素的定性研究[J].中华预防医学杂志，2018，52(12):1234-1238.
[1638]孟琴，朱秋映，谢志春.不同人口学特征男男性行为人群 HIV 感染研究进展[J].应用预防医学，2018，24(01):82-84.
[1639]王毅，李六林，樊静，等.不同文化程度男男性行为者的性行为特征及 HIV 感染现状[J].预防医学情报杂志，2018，34(09):1162-1168.

[1640]殷方兰，钟培松，张永，等.不同性取向的男男性行为人群行为特征差异性分析[J].中国皮肤性病学杂志，2018，32(08):898-901.
[1641]王金塔，周建波，甄森，等.常州市自愿咨询检测门诊男男性行为学生人群高危行为及感染状况分析[J].江苏预防医学，2018，29(04):427-429.
[1642]杨诗凡，范超楠，李巨臣，等.成都高校学生男男性行为者高危性行为现状及影响因素分析[J].预防医学情报杂志，2018，34(07):866-870.
[1643]熊伟，罗青云，张睿，等.成都某高校男同性恋大学生抑郁情况及影响因素[J].中国学校卫生，2018，39(08):1239-1241.
[1644]杨诗凡，李巨臣，张嘉祺，等.成都市学生男男性行为者基于交友软件寻找性伴侣现状及影响因素[J].中国艾滋病性病，2018，24(01):66-69.
[1645]王菊，何勤英，李宓儿，等.成都市知晓自身感染艾滋病病毒的男男性行为人群无保护肛交行为研究[J].中华流行病学杂志，2018，39(07):954-958.
[1646]樊爱平，杨冬芳，成玲，等.大学生男男性行为人群首次同性性行为与相关行为调查[J].中国性科学，2018，27(05):157-160.
[1647]宣舟斌，张宏伟，商颖，等.大学生男男性行为者 HIV 传播校园环境因素的定性研究[J].中国学校卫生，2018，39(01):117-119.
[1648]陈柏芬，谢亚利，朱建琼，等.东莞市男男性行为人群 HIV 抗体检测状况及影响因素分析[J].华南预防医学，2018，44(01):34-37.
[1649]谢亚利，陈柏芬，张巧利，等.东莞市男男性行为人群艾滋病防治网络干预效果评价[J].预防医学情报杂志，2018，34(03):273-277.
[1650]刘晓霞，张明辉，叶鲁，等.对镇江市 MSM 开展以梅毒筛查和转介为主的综合干预的研究[J].中国艾滋病性病，2018，24(03):283-285.
[1651]罗晓敏，刘珺，梁自勉，等.佛山市男男性行为者抑郁症状及相关因素研究[J].广东药科大学学报，2018，34(01):115-118.
[1652]蒋平，唐德谊，聂欢，等.个案护理对提高 ART 中 MSM 人群艾滋病相关知识和行为的效果[J].现代医药卫生，2018，34(05):680-683.
[1653]孟琴，蓝光华，沈智勇，等.广西 2006-2016 年男男性行为者 HIV 感染流行病学特征及趋势分析[J].中国艾滋病性病，2018，24(02):155-158.
[1654]曾梓，黎芝，蓝光华，等.广西壮族自治区男男性行为者对艾滋病非职业暴露后预防服务需求的定性研究[J].中华疾病控制杂志，2018，22(12):1274-1277.
[1655]梁容娇，汤少开，何婉苹，等.广州地区 MSM 和 FSWs 的性病知晓情况及梅毒艾滋病感染因素分析[J].皮肤性病诊疗学杂志，2018，25(04):240-246.
[1656]庾泳，肖水源，吴婷.广州市区男同性恋人群的约会暴力病例对照研究[J].中国心理卫生杂志，2018，32(04):335-338.
[1657]张玲，王尚博，杨一偲，等.哈尔滨市 2013-2015 年男男性行为者 HIV-1 新发感染情况分析[J].中国艾滋病性病，2018，24(02):152-154.
[1658]李一，惠珊，张锦慧，等.哈尔滨市男男性行为人群队列失访组与随访组高危性行为特征比较分析[J].中国公共卫生管理，2018，34(02):246-249.
[1659]张玲，王尚博，杨一偲，等.哈尔滨市男男性行为者 HIV 和梅毒感染状况及行为特征分析[J].中国艾滋病性病，2018，24(11):1161-1162.
[1660]李西婷，罗艳，张兴亮，等.杭州市男男性行为人群 HIV 新发感染及影响因素研究[J].预防医学，2018，30(10):982-986.
[1661]杨中荣，李婧，金玫华，等.湖州市男男性行为人群艾滋病哨点监测结果分析[J].预防医学，2018，30(05):510-512.

[1662]吴丽萍，孙修福，张兆辉，等.淮安市 HIV 抗体阳性男男性行为者生存质量干预效果评估[J].江苏预防医学，2018，29(03):276-278.
[1663]罗业涛，敬丹，叶孟良.基于 IMB 模型的流动 MSM 艾滋病预防行为研究[J].中国预防医学杂志，2018，19(11):816-821.
[1664]罗业涛，敬丹，胡玲，等.基于 IMB 模型的年轻 MSM 安全套使用及其影响因素[J].中华疾病控制杂志，2018，22(12):1257-1260.
[1665]郭静，郭巍，杨洪玲，等.基于结构方程模型的男男性行为人群无保护性行为的影响因素分析[J].中华预防医学杂志，2018，52(12):1225-1228.
[1666]段青，黄鹏翔，廖玫珍，等.济南青岛两市男男性行为者毒品滥用情况及其相关因素分析[J].中国艾滋病性病，2018，24(08):809-812.
[1667]杨冬梅，张传俊，徐锴，等.库尔勒市男男性行为者的 HIV 感染状况及影响因素[J].中国艾滋病性病，2018，24(10):1024-1027.
[1668]杨爱学，李淼.奎屯市 244 例 MSM 人群艾滋病知识行为及感染状况分析[J].实用预防医学，2018，25(05):576-579.
[1669]高晓娟，章任重，李佑芳，等.昆明市男男性行为者的抑郁状况及影响因素分析[J].中国艾滋病性病，2018，24(02):170-173.
[1670]陶连弟，程宝莲.兰州市城关区男男性接触人群性病/艾滋病危险因素调查[J].疾病预防控制通报，2018，33(04):44-47.
[1671]王毅，李六林，周万明，等.绵阳市 15 ~ 24 周岁男男性行为者 HIV 感染现状及影响因素[J].中国艾滋病性病，2018，24(06):576-580.
[1672]王毅，周万明，樊静，等.绵阳市 2017 年男男性行为者艾滋病哨点监测及 HIV 感染影响因素分析[J].华南预防医学，2018，44(02):101-105.
[1673]王毅，李六林，樊静，等.绵阳市不同寻找性伴场所 MSM 的性行为特征及 HIV 感染现状[J].实用预防医学，2018，25(09):1029-1034.
[1674]王毅，周万明，樊静，等.绵阳市男男性行为人群 rushpoppers 使用及相关因素[J].中华预防医学杂志，2018，52(12):1290-1292.
[1675]王毅，李六林，樊静，等.绵阳市男男性行为人群应对方式现状及影响因素[J].预防医学情报杂志，2018，34(05):557-563.
[1676]王毅，李六林，樊静，等.绵阳市男男性行为者 HIV 意愿检测地点及影响因素[J].中国热带医学，2018，18(03):236-241.
[1677]王毅，李六林，樊静，等.绵阳市男男性行为者定期 HIV 检测现状及影响因素分析[J].中国病毒病杂志，2018，8(01):11-16.
[1678]王毅，李六林，樊静，等.绵阳市男男性行为者交友目的及关联因素分析[J].中国性科学，2018，27(09):156-160.
[1679]王毅，李六林，樊静，等.绵阳市男男性行为者需求层次及关联因素分析[J].华南预防医学，2018，44(03):210-215.
[1680]王毅，李六林，周万明，等.绵阳市年轻男男性行为者艾滋病感染风险认识与行为分离及影响因素[J].中国艾滋病性病，2018，24(11):1119-1123.
[1681]王毅，李六林，周万明，等.绵阳市年轻男男性行为者儿童期性虐待经历对个体行为及心理健康的影响[J].中国艾滋病性病，2018，24(09):912-915.
[1682]王毅，李六林，周万明，等.绵阳市年轻男男性行为者自杀行为及影响因素[J].中国病毒病杂志，2018，8(05):370-374.
[1683]马国静，田文静，王常智，等.牡丹江市男男性行为人群艾滋病感染和安全套使用及影响因素分析[J].中国公共卫生管理，2018，34(02):164-167.

[1684]马萍，王子瑜，闫忠芳.男男同性性行为人群 HIV 感染的危险因素及干预措施的研究进展[J].山东医药，2018，58(33):110-113.
[1685]鲍燕，沈冰，宁镇，等.男男性传播 HIV 感染者对早期抗病毒治疗的可接受性及影响因素研究[J].上海预防医学，2018，30(03):217-222.
[1686]鲍燕，沈冰，顾凯凯，等.男男性接触 HIV 感染者对早期抗病毒治疗认知情况及行为特征分析[J].中国初级卫生保健，2018，32(05):66-68.
[1687]肖江峰，黄倩，杨小华，等.男男性行为群体 HIV 暴露前预防用药接受的意愿及其影响因素[J].海南医学，2018，29(04):575-578.
[1688]钟晓娟.男男性行为人群 HIV 和梅毒感染现状及影响因素分析[J].中国卫生工程学，2018，17(02):226-228.
[1689]杨蓓，陈于，吴国辉.男男性行为人群 HIV 新发感染及影响因素研究进展[J].预防医学情报杂志，2018，34(08):1104-1109.
[1690]郭巍，李一，周宁，等.男男性行为人群艾滋病病毒新发感染风险的队列研究[J].中华流行病学杂志，2018，39(01):16-20.
[1691]岑平，王永.男男性行为人群艾滋病感染的影响因素研究进展[J].中国热带医学，2018，18(12):1269-1272.
[1692]史灵恩，闫红静，魏崇义，等.男男性行为人群暴露前预防意愿调查[J].江苏预防医学，2018，29(05):510-511.
[1693]李玲玲，何纳.男男性行为人群个体 HIV 感染风险评估模型研制进展及启示[J].中华预防医学杂志，2018，52(08):862-868.
[1694]杨娟，徐慧芳，程伟彬，等.男男性行为人群个体社会网络中关系强度及其相关因素分析[J].中华流行病学杂志，2018，39(04):478-482.
[1695]杨娟，徐慧芳，邢辉，等.男男性行为人群个体中心社会网络特征与 HIV 流行的关联[J].中华预防医学杂志，2018，52(12):1215-1219.
[1696]王小芳，吴尊友，唐振柱，等.男男性行为人群利用艾滋病病毒抗体唾液快速检测试剂自检的可接受性调查[J].中华流行病学杂志，2018，39(07):937-942.
[1697]范雄智，刘仲琦，程伟彬，等.男男性行为人群同伴社会网络规范对其 HIV 检测行为的影响[J].中华疾病控制杂志，2018，22(01):38-41.
[1698]李镠，刘轶，芦文丽.男男性行为人群新型毒品使用情况和 HIV 感染调查分析[J].中国慢性病预防与控制，2018，26(01):24-27.
[1699]殷方兰，张永，王嘉行，等.男男性行为人群性伴网络及高危行为特征研究[J].上海预防医学，2018，30(12):975-979.
[1700]游霞，刘激扬，王敏.男男性行为者 HIV 感染相关风险因素[J].国际流行病学传染病学杂志，2018，45(05):343-348.
[1701]王毅，李六林，樊静，等.男男性行为者 HIV 检测场所偏好及影响检测原因[J].中国艾滋病性病，2018，24(02):164-169.
[1702]张忠心.男男性行为者 HIV 自检意愿及影响因素的网络调查结果分析[J].安徽预防医学杂志，2018，24(04):262-266.
[1703]谢莉，罗淑星，周海龙，等.男男性行为者艾滋病患者相关羞辱和歧视影响因素研究[J].预防医学情报杂志，2018，34(09):1158-1161.
[1704]姜婷婷，陈琳，周欣，等.男男性行为者定期检测现状及其影响因素分析[J].中国艾滋病性病，2018，24(02):148-151.
[1705]石舒原，王泽洲，沈秋明，等.男男性行为者高危性行为影响因素及理论模型综述[J].上海交通大学学报(医学版)，2018，38(10):1242-1246.

[1706]梁能秀，蓝光华.男男性行为者及其配偶感染艾滋病风险研究进展[J].应用预防医学，2018，24(02):164-166.
[1707]王毅，李六林，樊静，等.男男性行为者流动人口现状及与艾滋病相关因素的关系[J].预防医学情报杂志，2018，34(08):1074-1079.
[1708]宋秀萍，丁心平，杨鲁光，等.男男性行为者心理弹性及其影响因素研究[J].现代预防医学，2018，45(23):4369-4371.
[1709]刘薇，路亮，刘明斌，等.南昌市男男性行为者寻找陌生同性临时性伴行为及相关因素分析[J].中国艾滋病性病，2018，24(04):365-367.
[1710]徐园园，李小杉，吴苏姝，等.南京市 2011—2016 年 15～24 岁人群艾滋病流行特征分析[J].中国学校卫生，2018，39(08):1202-1205.
[1711]郭璐，朱正平，徐园园，等.南京市男男性行为人群艾滋病感染风险评估模型的建立[J].中国健康教育，2018，34(09):788-792.
[1712]郭璐，张敏，朱正平.南京市男男性行为者中异性性行为特征及影响因素分析[J].中国健康教育，2018，34(04):325-329.
[1713]迟富利，涛波，杨景元，等.内蒙古男男性接触者 HIV 感染相关影响因素调查[J].实用预防医学，2018，25(02):219-222.
[1714]王毅，李六林，周万明，等.年轻男男性行为者艾滋病预防服务覆盖现状及信息获取渠道关联因素分析[J].华南预防医学，2018，44(06):506-512.
[1715]王毅，李六林，周万明，等.年轻男男性行为者焦虑和抑郁症状与社会行为的关系[J].华南预防医学，2018，44(05):401-405.
[1716]李放，王一博，邢锦涛，等.年轻男同性恋者自我概念清晰性与生命意义感的关系及作用机制[J].心理科学，2018，41(05):1178-1184.
[1717]陶毓敏，洪航，姜海波，等.宁波市男男性行为人群艾滋病知信行调查[J].预防医学，2018，30(03):317-319.
[1718]张丹丹，洪航，姜海波.宁波市男男性行为人群抑郁状况调查[J].预防医学，2018，30(05):442-445.
[1719]卢姗，李培龙，葛琳，等.青岛市男男性行为者中滥用新型毒品者的艾滋病相关性行为特征[J].中国艾滋病性病，2018，24(04):368-372.
[1720]朱正平，徐园园，吴苏姝，等.青年男男性行为人群艾滋病、梅毒感染的流行病学调查[J].中华疾病控制杂志，2018，22(12):1265-1269.
[1721]杨惠民，王威，陈向凡，等.青少年男男性行为人群多性伴与相关因素分析[J].现代预防医学，2018，45(13):2460-2463.
[1722]李煜，李十月，杨惠民，等.青少年男男性行为人群性感觉寻求水平与首次同性性行为研究[J].中华疾病控制杂志，2018，22(12):1270-1273.
[1723]杨特，廖玫珍，李瑞，等.山东省 2011-2015 年浴池男男性行为者 HIV 感染和行为变化及影响因素[J].中国艾滋病性病，2018，24(04):348-352.
[1724]韩莹，潘晶，韩秀云.山东省济南市男男性行为人群 HIV、梅毒、及人类单纯疱疹病毒 2 型感染状况分析[J].中华疾病控制杂志，2018，22(08):844-845.
[1725]段青，廖玫珍，黄鹏翔，等.山东省男男性行为者哨点毒品滥用者 HIV 感染状况及影响因素分析[J].中国艾滋病性病，2018，24(02):143-147.
[1726]卢姗，孙坤，李培龙，等.山东省青岛市男男性行为人群固定性伴无保护性肛交行为的通径分析[J].疾病监测，2018，33(10):844-848.
[1727]尹宁，倪莹青，王震宇，等.上海市 297 名男男性行为者对 HIV 抗体唾液快速检测的使用意愿及影响因素调查[J].中国艾滋病性病，2018，24(05):499-501.

[1728]宣舟斌，商颖，徐湘，等.上海市高校学生男男性行为者艾滋病高危性行为定向干预效果评价[J].现代预防医学，2018，45(11):2067-2072.
[1729]鲍燕，沈冰，顾凯侃，等.上海市静安区 218 例男男性行为者感染 HIV 后早期抗病毒治疗的可接受性调查[J].中国艾滋病性病，2018，24(04):353-356.
[1730]张贺礼，陈富讯，丁以标，等.上海市浦东新区男男性行为者艾滋病知识及相关危险因素调查[J].职业与健康，2018，34(21):2927-2930.
[1731]谢言，刘瑛，杨咏梅.上海市自愿咨询检测门诊男男性行为者的抑郁症状及相关因素[J].中华疾病控制杂志，2018，22(12):1248-1251.
[1732]谭唯，赵锦，刘少础，等.深圳市 2014—2016 年男男性行为者 HIV 流行特征分析[J].中国艾滋病性病，2018，24(05):502-504.
[1733]孙群露，涂玉山，钱兵，等.深圳市宝安区男男性行为者高危性行为和 HIV 感染现状[J].中国艾滋病性病，2018，24(06):573-575.
[1734]李晓霞，刘渠，谢显清，等.深圳市龙岗区男男性行为者 HIV 感染状况及其高危行为特征分析[J].中国艾滋病性病，2018，24(03):246-249.
[1735]蔡于茂，宋亚娟，刘惠，等.深圳市男男性行为者 HIV 风险感知的影响因素[J].中国艾滋病性病，2018，24(01):62-65.
[1736]黄掣驰，毛翔，张伟，等.沈阳 HIV 阳性已婚男男性行为者的配偶告知状况及阻碍因素分析[J].中国艾滋病性病，2018，24(03):250-253.
[1737]云科，张晶，楚振兴，等.沈阳市 HIV 阴性男男性行为者的心理障碍与 CD4~+T 淋巴细胞水平的关联性研究[J].中国艾滋病性病，2018，24(11):1115-1118.
[1738]张永惠，石锋，楚振兴，等.沈阳市 VCT 门诊求询男男性行为者的性社会网络特征与无保护肛交行为的关联性[J].中国艾滋病性病，2018，24(06):565-568.
[1739]苏志寿，阮菁如，王云霞，等.思明区男男性行为人群艾滋病感染状况及危险因素分析[J].海峡预防医学杂志，2018，24(01):45-47.
[1740]谭明天，钟晓妮，彭斌，等.四川和重庆地区大中学生男男性接触者 HIV 感染状况及其相关危险性行为调查[J].重庆医科大学学报，2018，43(07):975-979.
[1741]杨昊，袁琦，王宇扬，等.四川省 HIV 阳性男男性行为者社会支持及心理健康[J].四川大学学报(医学版)，2018，49(06):966-969.
[1742]王毅，李六林，周万明，等.四川省绵阳市年轻男男性行为者艾滋病病毒、梅毒感染现况及梅毒感染影响因素[J].疾病监测，2018，33(10):849-853.
[1743]陈潇潇，乔晓彤，沈伟伟，等.台州市 2010—2017 年哨点监测 50 岁以上男男性行为人群 HIV 感染状况及相关因素分析[J].上海预防医学，2018，30(12):980-985.
[1744]柏建芸，刘轶，柳忠泉，等.天津市 2012-2016 年青年学生男男性行为者的行为特点及 HIV 和梅毒感染状况[J].中国艾滋病性病，2018，24(01):58-61.
[1745]许琳.天津市 MSM 人群艾滋病知晓情况的网络调查和影响因素[J].职业与健康，2018，34(16):2275-2277.
[1746]周宁，郑敏娜，李环环，等.天津市男男性行为人群随访干预的效果评价[J].中华流行病学杂志，2018，39(09):1228-1233.
[1747]董笑月，龚卉，柏建芸.天津市男男性行为者 HIV 感染状况及影响因素分析[J].国际病毒学杂志，2018，25(05):323-327.
[1748]马龙.铜川市男男性行为人群 HIV 感染情况及相关因素分析[J].预防医学情报杂志，2018，34(04):462-466.
[1749]张倩，贺晋栋，王彤.我国男男性行为人群抑郁症状检出情况[J].职业与健康，2018，34(17):2407-2411.

[1750]田恬，桑国耀，张发东，等.乌鲁木齐市 HIV 阴性男男性行为人群自费人乳头瘤病毒疫苗接种意愿分析[J].中国公共卫生，2018，34(02):229-233.
[1751]倪永康，王云霞，刘早玲.乌鲁木齐市男男性行为者 HIV 新发感染及危险因素研究[J].实用预防医学，2018，25(06):681-684.
[1752]罗莉，曹琳，胡荣，等.武汉市 2016 年新报告 HIV 阳性男男性行为者交友模式及特征[J].中国艾滋病性病，2018，24(01):78-80.
[1753]谢年华，吴斯，许骏，等.武汉市男男性行为者扩大 HIV 检测和治疗的效果分析[J].中国艾滋病性病，2018，24(04):361-364.
[1754]陶宜新，赵长成，高晓宇，等.西宁地区男男性行为人群艾滋病高危行为综合干预效果研究[J].医学动物防制，2018，34(11):1032-1035.
[1755]薛建，程晓松，林荣，等.烟台市 MSM 人群性行为特征及安全套使用影响因素研究[J].应用预防医学，2018，24(02):98-101.
[1756]于飞，米国栋，陈子煌，等.应用 Blued 平台进行 HIV 检测影响因素的定性研究[J].中华预防医学杂志，2018，52(12):1229-1233.
[1757]宋丽军，陈会超，梅静远，等.云南省 2012—2015 年男男性行为者 HIV 感染率及新近感染率分析[J].中国艾滋病性病，2018，24(10):1020-1023.
[1758]陈琳，马瞧勤，周欣，等.浙江省 HIV 感染 MSM 人群的性伴网络特征分析[J].预防医学，2018，30(06):545-548.
[1759]赵东设，罗明宇，朱海深，等.浙江省温州市居住 5 年及以下男男性行为人群艾滋病病毒感染调查[J].中华流行病学杂志，2018，39(07):948-953.
[1760]陈彦哲，仇元营，李国伟，等.郑州市男男性接触者行为特征及 HIV、梅毒、丙肝感染情况分析[J].医学动物防制，2018，34(03):218-221.
[1761]许娟，韩晶，汤后林，等.中国 2008—2015 年新报告男男性行为 HIV/AIDS 未接受随访干预情况及相关因素分析[J].中华流行病学杂志，2018，39(04):495-499.
[1762]孟晓军，贾天剑，尹寒露，等.中国 3 个城市 MSM 人群无偿献血行为状况及影响因素分析[J].中华流行病学杂志，2018，39(11):1443-1448.
[1763]石安霞，张志华，王君，等.中国大陆学生男男性行为及 HIV 感染检出率的 Meta 分析[J].中国学校卫生，2018，39(05):702-705.
[1764]谢梅茂，熊仪，王忠军，等.中国学生男男性行为者艾滋病感染状况分析[J].中国医药导报，2018，15(02):172-175.
[1765]莫实德.中国男男性行为者艾滋病高危行为特征研究进展[J].安徽预防医学杂志，2020，26(01):42-45.
[1766]李瑶，何小庆，张露，等.重庆地区 HIV 阴性男男性行为者药物滥用情况分析[J].中国感染控制杂志，2020，19(02):126-130.
[1767]胡桂林，陈先刚，李向东，等.重庆主城区 MSM 人群 HIV 感染及影响因素分析[J].现代医药卫生，2020，36(04):498-502.
[1768]王毅，李六林，周万明，等.15 ~ 24 岁年轻男男性行为者性伴相处坚持自我及影响因素[J].实用预防医学，2019，26(04):385-388.
[1769]朱洁群.2006-2017 年宁波市鄞州区 MSM 人群 HIV/AIDS 流行特征分析[J].中国农村卫生事业管理，2019，39(09):668-670.
[1770]黄敏，王德辉，侯雪芹，等.2007-2018 年广元市男男同性传播艾滋病现况分析[J].预防医学情报杂志，2019，35(12):1431-1434.
[1771]宁铁林，郑敏娜，柏建芸，等.2010—2015 年天津市男男同性性行为者 HIV-1 新近感染状况调查[J].中国病毒病杂志，2019，9(02):91-95.

[1772]杨洪，叶黎，苏玲，等.2011—2015 年四川省男男性行为人群 HIV-1 新发感染率及流行趋势分析[J].中华预防医学杂志，2019(03):327-329.
[1773]王庆丰, 沈美枫.2013—2016 年常熟市 VCT 门诊男男性行为者检测结果[J].江苏预防医学，2019，30(02):176-177.
[1774]姜叶，林海江，许圆圆，等.2013-2016 年浙江省台州市男男性行为人群同性偶遇性行为调查[J].疾病监测，2019，34(03):260-264.
[1775]张超，任强，付钰淋，等.2013-2017 年陕西省 HIV 阳性男男性行为者行为特征分析[J].现代预防医学，2019，46(18):3419-3422.
[1776]杨蓓，卢戎戎，秦毅，等.2013—2017 年重庆市某郊区男男性行为者的 HIV 新发感染状况及影响因素[J].中国艾滋病性病，2019，25(02):153-157.
[1777]朱凯星，何虹励，许美振，等.2014-2016 年广州市海珠区男男性行为人群艾滋病知识和行为调查[J].中国健康教育，2019，35(01):40-45.
[1778]刘凤仁，龙清平，黄飞雁，等.2014-2017 年深圳市龙岗区男男同性传播 HIV 人群 HIV 检测影响因素分析[J].实用预防医学，2019，26(08):971-974.
[1779]宋秀萍，孙良，胡冰，等.2014-2018 年阜阳市男男性行为者 HIV 感染监测结果分析[J].预防医学情报杂志，2019，35(08):845-850.
[1780]王毅，李六林，周万明，等.2015-2017 年绵阳市年轻男男性行为者安全套使用及影响因素[J].实用预防医学，2019，26(09):1055-1059.
[1781]潘庆礼，周健，朱焱，等.2015—2018 年贵阳市男男性行为者 HIV 感染状况及性行为特征分析[J].中国艾滋病性病，2019，25(08):812-815.
[1782]贾中毅，徐靖，赵涛.2015-2018 年南阳市 MSM 人群 HIV 哨点监测结果及影响因素分析[J].河南预防医学杂志，2019，30(08):634-637.
[1783]李静，许圆圆，李桂霞，等.2015-2018 年台州市年轻男男性行为者异性性行为特征及其影响因素[J].中华疾病控制杂志，2019，23(12):1471-1475.
[1784]乔丹，刘征，董志伟，等.2016-2018 年郑州市男男性行为人群哨点监测结果及 HIV 感染影响因素分析[J].现代预防医学，2019，46(19):3603-3607.
[1785]王毅，周万明，樊静，等.2017 年绵阳市男男性行为者艾滋病预防服务覆盖及知识知晓影响因素[J].职业与健康，2019，35(18):2503-2507.
[1786]邱兴庆，孟丽丽，崔德勇.2017 年襄阳市 MSM 人群 HIV 感染状况及影响因素分析[J].实用预防医学，2019，26(04):393-396.
[1787]戴悦婵，齐虎成，谢燕珊.2018 年广州市天河区车陂街社区艾滋病男男性行为人群高危性行为影响因素分析[J].预防医学论坛，2019，25(05):338-340.
[1788]周宗磊，詹志强，孙群露，等.2018 年深圳市宝安区 358 名男男性行为者 HIV/TP 感染情况及影响因素分析[J].预防医学情报杂志，2019，35(12):1435-1440.
[1789]张震，李静，陈玲娟，等.2018 年浙江省台州市男男性行为人群发生异性性行为特征及其影响因素分析[J].疾病监测，2019，34(11):1017-1021.
[1790]王毅，李六林，樊静，等.2019 年绵阳市男男性行为者艾滋病哨点监测结果及其 HIV 感染影响因素分析[J].寄生虫病与感染性疾病，2019，17(04):189-194.
[1791]董文斌，赵金仙，李世福，等.400 例 MSM 行为特征及 HIV、HAV、HCV、HEV、梅毒感染状况的横断面调查[J].现代预防医学，2019，46(10):1875-1878.
[1792]高迪思，吴静，胡翼飞，等.4 城市青年学生中 HIV 感染者推介的男男性行为者多性伴侣与安全套使用的情况[J].中国艾滋病性病，2019，25(11):1120-1124.
[1793]苏叶，邢鹏博，张秀，等.HIV 感染的男男性行为者对同性固定性伴阳性告知的质性研究[J].中国护理管理，2019，19(12):1774-1778.

[1794]康婧，耿文清，姜拥军，等.HIV 感染男男性行为者抑郁状态的相关因素分析[J].中国艾滋病性病，2019，25(12):1234-1237.
[1795]梁能秀，蓝光华.HIV 阳性男男性行为人群特征及其死亡相关影响因素分析[J].中国公共卫生，2019，35(05):587-590.
[1796]刘应南，谢通，张悦怡，等.HIV 阳性男男性行为者肛门高危型 HPV 感染的危险因素[J].中国艾滋病性病，2019，25(05):543-547.
[1797]肖敏，黄金，王红红，等.HIV 阳性男男性行为者抗病毒治疗依从性现状及影响因素研究[J].中国艾滋病性病，2019，25(01):48-51.
[1798]肖敏，黄金，王红红，等.HIV 阳性男男性行为者医学应对方式和社会支持的相关性研究[J].中国艾滋病性病，2019，25(05):476-479.
[1799]陈怡.MSM 人群感染和传播 HIV 的影响因素研究进展[J].应用预防医学，2019，25(03):259-261.
[1800]罗文晓，刘志琦，刘世文，等.白银市 2009—2016 年男男性行为人群艾滋病监测结果分析[J].中国公共卫生管理，2019，35(05):710-713.
[1801]宋亮，姜树林，胡尧，等.北京市 324 名男男性行为者性病求医意向和行为分析[J].实用预防医学，2019，26(12):1470-1472.
[1802]赵丹鹤，李东亮.北京市学生男男性行为者 HIV 和梅毒感染状况[J].中国艾滋病性病，2019，25(12):1276-1279.
[1803]陆天意，毛翔，高阳阳，等.采用 HIV 自我检测策略促进男男性行为者群体 HIV 检测研究进展[J].中国艾滋病性病，2019，25(06):648-651.
[1804]余林，黄玉玲，谢天，等.成都市不同活动区域男男性行为人群高危行为特征分析[J].预防医学情报杂志，2019，35(09):1009-1013.
[1805]余雁华，黄惠燕，池招贵，等.福建省沿海城市青年 MSM 人群 HIV 检测影响因素的研究分析——以莆田市为例[J].九江学院学报(自然科学版)，2019，34(01):69-73.
[1806]张严文，叶宝娟.父母拒绝教养方式对中国同性恋者自杀的影响:歧视知觉的中介效应与出柜的调节效应[J].心理科学，2019，42(01):109-115.
[1807]张夏梦，张先庚，梁小利，等.高校男男性行为者 HIV 感染现状及“知-信-行”健康教育模式[J].中国艾滋病性病，2019，25(11):1192-1195.
[1808]曾梓，黎芝，李建标，等.广西部分地区男男性行为者安全套使用情况及影响因素分析[J].中国热带医学，2019，19(04):343-347.
[1809]刘雪梅，过恒升，范引光，等.广西柳州 MSM 艾滋病非职业性暴露后预防服务的需求和影响因素[J].中华疾病控制杂志，2019，23(12):1517-1522.
[1810]李春英，朱秋映，唐帅，等.广西年轻男男性行为人群 HIV/梅毒感染和高危性行为状况及其影响因素分析[J].应用预防医学，2019，25(02):83-87.
[1811]靳伟，程伟彬，钟斐，等.广州市 HIV 阳性男男性行为者推荐自检试剂用于性伴检测意愿及影响因素[J].中国艾滋病性病，2019，25(05):472-475.
[1812]罗业飞，古羽舟，钟斐，等.广州市 MSM"HIV 感染风险在线自评系统"使用者风险特征分析[J].中华流行病学杂志，2019(10):1217-1221.
[1813]蔡衍珊，古羽舟，钟斐，等.广州市 MSM"互联网+HIV 自检服务平台"——"岭南准"的适用性和可行性分析[J].中华流行病学杂志，2019(10):1212-1216.
[1814]古羽舟，程伟彬，王艾斯，等.广州市 MSM"互联网+艾滋病综合预防服务体系"对 HIV/AIDS 关怀效果分析[J].中华流行病学杂志，2019(10):1222-1226.
[1815]朱凯星，许美振，宋叶，等.广州市海珠区男男性行为人群安全套使用影响因素分析[J].中国初级卫生保健，2019，33(02):66-69.

[1816]程伟彬，李顺铭，古羽舟，等.广州市互联网型 MSM 使用"互联网+艾滋病综合预防服务体系"现状及特征分析[J].中华流行病学杂志，2019(10):1206-1211.
[1817]江洪波，黎静，谭芷敏，等.广州市男男性行为人群性感觉寻求与高危性行为的关联[J].中华疾病控制杂志，2019，23(07):818-821.
[1818]江洪波，谭芷敏，黎静，等.广州市男男性行为学生艾滋病检测行为及影响因素分析[J].中国学校卫生，2019，40(08):1200-1202.
[1819]梁容娇，王成，赵培祯，等.广州市男男性行为者 HIV 感染状况及影响因素[J].中华疾病控制杂志，2019，23(12):1482-1486.
[1820]谭芷敏，黎静，陈晓滨，等.广州市男男性行为者儿童期性虐待经历对成年后高危性行为的影响[J].中华疾病控制杂志，2019，23(12):1487-1491.
[1821]林铠浩，黎静，谭芷敏，等.广州市男男性行为者亲密伴侣暴力与无保护肛交的关联研究[J].现代预防医学，2019，46(16):3005-3008.
[1822]赖志胜，李艳芳，卢次勇，等.广州市男男性行为者抑郁状况及影响因素分析[J].预防医学情报杂志，2019，35(12):1354-1358.
[1823]江洪波，林铠浩，黎静，等.广州市年轻男男性行为人群艾滋病相关知识知晓情况及其影响因素分析[J].国际病毒学杂志，2019(03):160-164.
[1824]郑燕，曾文姬，宋业兵，等.贵阳市 74 名 MSM 艾滋病非职业暴露后预防调查分析[J].贵州医药，2019，43(11):1814-1816.
[1825]陈超，狄畅，闫存玲.哈尔滨市 HIV 阳性 MSM 抗病毒治疗服药依从性现状调查与分析[J].国际免疫学杂志，2019(02):142-146.
[1826]王召乾，张帆，曾小妹，等.海口市不同性角色 MSM 青年学生社会接纳和性行为特征[J].中国热带医学，2019，19(06):542-547.
[1827]笪琴，彭国平，汤恒，等.湖北省艾滋病感染男男性行为人群婚姻及配偶检测情况[J].中国热带医学，2019，19(06):538-541.
[1828]许向东，潘杰，沈金菁，等.湖州市区 94 名 50 岁以上男男性行为人群艾滋病认知现状调查分析[J].安徽预防医学杂志，2019，25(01):62-65.
[1829]许向东，吕鑫，凌建，等.湖州市区 MSM 人群艾滋病 KAP 调查及影响因素分析[J].中国农村卫生事业管理，2019，39(04):290-293.
[1830]张爱迪，黄耳，谭思敏，等.互联网对青少年同性恋身份认同发展的影响研究进展[J].中国艾滋病性病，2019，25(04):429-431.
[1831]张勇，高静，张鹏.基于 QOL 评价的自我管理模式在 HIV(+)MSM 中的实证研究[J].公共卫生与预防医学，2019，30(05):33-36.
[1832]王毅，李六林，樊静，等.基于马斯洛需求层次理论的绵阳市男男性行为者需求层次调查[J].预防医学情报杂志，2019，35(04):320-324.
[1833]秦小芬，李现红，王红红，等.基于社会生态学模型的男男性行为者高危性行为的影响因素及干预模式[J].中国艾滋病性病，2019，25(06):655-658.
[1834]孙金铭，傅泽鑫，鲍凡凡，等.基于社交软件的浙江省大学生男男性行为者艾滋病检测情况及影响因素研究[J].浙江医学教育，2019，18(06):13-15.
[1835]孙丽，周建波，王金塔.江苏省常州市自愿咨询检测门诊学生男男性行为者 HIV 感染状况及相关因素[J].中华预防医学杂志，2019(03):293-297.
[1836]吴小刚，史灵恩，陈禹衡，等.江苏省老年男男性行为人群艾滋病病毒感染状况及危险因素分析[J].东南大学学报(医学版)，2019，38(06):1055-1059.
[1837]张娜，刘丽萍，易志强，等.江西省 659 例男男性行为者尿液艾滋病匿名检测结果分析[J].中国艾滋病性病，2019，25(03):299-300.

[1838]张林，孙美艳，任勇，等.江浙沪地区艾滋病男男同性恋人群心理情绪与同妻关系相关性调查研究[J].中国预防医学杂志，2019，20(04):271-275.
[1839]王凤英，唐慧玲.金华市男男性行为艾滋病患者生存时间与影响因素分析[J].中国公共卫生，2019，35(12):1633-1636.
[1840]张林，孙美艳，周莹，等.具有同妻婚姻关系的艾滋病男男同性恋人群焦虑状况分析[J].上海预防医学，2019，31(04):294-300.
[1841]肖璨，付朝智，马婧，等.开远市 200 名男男性行为者艾滋病暴露前预防用药使用意愿[J].中国艾滋病性病，2019，25(07):741-742.
[1842]李彩霞，李琪，王玉淼，等.昆明市不同性取向男男性行为人群的性行为特征分析[J].中国皮肤性病学杂志，2019，33(11):1286-1291.
[1843]张文冉，毛翔，彭二磊，等.利用社交媒体推动男男性行为者开展 HIV 自我检测研究进展[J].中国艾滋病性病，2019，25(07):760-764.
[1844]彭二磊，毛翔，李佳明，等.磷酸可待因滥用与 MSM 人群 HIV 感染及相关高危行为的关联性[J].实用预防医学，2019，26(01):1-4.
[1845]王毅，周万明，樊静，等.绵阳市 2017 年男男性行为者 HIV、梅毒血清学检测及 HIV 感染影响因素[J].中国病毒病杂志，2019，9(04):274-280.
[1846]王毅，周万明，樊静，等.绵阳市 2017 年男男性行为者性行为特征及无保护措施肛交影响因素[J].实用预防医学，2019，26(12):1412-1416.
[1847]王毅，周万明，樊静，等.绵阳市 MSM 异性婚姻关系及婚姻时间与艾滋病高危行为的关联性[J].中国公共卫生，2019:1-3.
[1848]王毅，周万明，樊静，等.绵阳市不同寻找性伴场所男男性行为者 HIV 感染状况及社会行为特征[J].中国热带医学，2019，19(08):747-751.
[1849]王毅，樊静，赵西和，等.绵阳市男男性行为者艾滋病高危性行为并存现状及影响因素[J].中国艾滋病性病，2019，25(10):1042-1046.
[1850]王毅，李六林，樊静，等.绵阳市男男性行为者安全感与艾滋病相关社会行为的关系研究[J].预防医学情报杂志，2019，35(12):1374-1378.
[1851]王毅，周万明，樊静，等.绵阳市男男性行为者传统婚姻态度及结婚原因的关联因素[J].中国艾滋病性病，2019，25(08):820-823.
[1852]王毅，周万明，樊静，等.绵阳市男男性行为者无保护肛交和 HIV 感染的影响因素[J].国际流行病学传染病学杂志，2019(01):19-24.
[1853]王毅，周万明，樊静，等.绵阳市男男性行为者异性婚姻状况及离异或丧偶者的相关特征分析[J].华南预防医学，2019，45(02):138-141.
[1854]王毅，李六林，周万明，等.绵阳市年轻男男性行为者艾滋病感染风险认识及影响因素[J].职业与健康，2019，35(03):359-362.
[1855]王毅，徐浩天，李六林，等.绵阳市年轻男男性行为者焦虑、抑郁症状现状调查[J].中国热带医学，2019，19(04):339-342.
[1856]王毅，李六林，周万明，等.绵阳市年轻男男性行为者性行为特征及肛交多性伴影响因素[J].中国热带医学，2019，19(01):57-61.
[1857]莫园园，蒋莉，苏丽西，等.男男同性恋 HIV 感染者/艾滋病病人心理痛苦的研究[J].护理研究，2019，33(21):3754-3757.
[1858]张鹏，高静，张勇，等.男男同性恋 HIV 感染者自我管理效果评价研究[J].中华疾病控制杂志，2019，23(05):582-587.
[1859]王海东，张璐，周莹，等.男男性接触者社交软件的使用和性行为特征及性传播疾病关系的系统综述[J].中国全科医学，2019，22(32):3969-3974.

[1860]赵丽娜，俞惠婷，谢红，等.男男性行为 HIV 感染者抑郁和应对现状及其相关关系[J].护理研究，2019，33(20):3594-3596.
[1861]高迪思，吴静，张文静，等.男男性行为青年学生艾滋病知识与行为现况[J].中国学校卫生，2019，40(03):359-363.
[1862]陈婉君，阮建军，朱碧香，等.男男性行为人群 HIV 感染状态知情交友调查[J].预防医学，2019，31(01):1-4.
[1863]任仙龙，芦明月，刘国武，等.男男性行为人群 HIV 自我检测方法进展[J].首都公共卫生，2019，13(01):42-44.
[1864]胡中柱，李二辉，张萌.男男性行为人群新型毒品使用及与 HIV 梅毒感染关系[J].中国性科学，2019，28(06):157-160.
[1865]戴色莺，程晓莉，刘爱文，等.男男性行为人群与男性性伴和女性性伴的性行为特征及影响因素[J].中华疾病控制杂志，2019，23(10):1260-1264.
[1866]王毅，李六林，樊静，等.男男性行为者对 HIV 感染预期影响的认知评价及关联因素[J].实用预防医学，2019，26(02):141-145.
[1867]刘荷冰，陈彦林，杨杨，等.男男性行为者对包皮环切认知的调查[J].中国艾滋病性病，2019，25(05):514-515.
[1868]贾月如，于欢，张晶，等.男男性行为者对利用手机社交软件推送 HIV 防控信息的接受意愿调查[J].中国艾滋病性病，2019，25(01):43-47.
[1869]王毅，李六林，周万明，等.男男性行为者焦虑抑郁症状与艾滋病知识、风险认知及性行为的关系[J].中华行为医学与脑科学杂志，2019，28(01):80-84.
[1870]周月芳，田明霞，王晓兰，等.男男性行为者新发 HIV 急性感染期心理护理干预效果评价[J].北京医学，2019，41(6):523-524.
[1871]许小艺，陈潇潇，许圆圆，等.男男性行为者与年长男性性伴之间性行为及 HIV 感染的现况研究[J].中国初级卫生保健，2019，33(06):61-64.
[1872]李放，黄李佳，钟林鹏，等.男同性恋者恋爱状况与生活满意度的关系：一个链式中介模型[J].中国临床心理学杂志，2019，27(04):785-789.
[1873]闫莉，李建军，闫红静，等.南京市男男性行为人群 HIV 性伴检测影响因素的 1:2 配比分析[J].南京医科大学学报(自然科学版)，2019，39(04):550-554.
[1874]徐园园，朱正平，吴苏姝，等.南京市男男性行为者新型毒品使用情况及相关因素[J].中华疾病控制杂志，2019，23(12):1476-1481.
[1875]潘海西，黄秋芳，刘海燕，等.南宁市男男性行为人群 HIV-1 新发感染率及感染危险因素分析[J].医学动物防制，2019，35(03):226-229.
[1876]王毅，李六林，周万明，等.年轻男男性行为者艾滋病高危行为应对方法及影响因素[J].华南预防医学，2019，45(03):221-226.
[1877]王毅，李六林，周万明，等.年轻男男性行为者首次同性性行为现状及安全套使用影响因素[J].现代预防医学，2019，46(02):310-313.
[1878]王毅，李六林，周万明，等.青年男男性行为者性行为安全套准备现状及影响因素[J].预防医学情报杂志，2019，35(05):419-423.
[1879]张文静，黄晓玲，高迪思，等.青年学生男男性行为人群 HIV 相关知识认知现状及 HIV 感染影响因素分析[J].中国公共卫生，2019，35(12):1598-1602.
[1880]赵亚芳，绳宇.情绪状态对男男性行为 HIV 新诊断感染者睡眠紊乱及治疗行为影响[J].中华疾病控制杂志，2019，23(04):480-485.
[1881]黄亚兰，陈秋婷，贺兴增，等.泉州市 2018 年男男性行为人群 HIV 感染及影响因素分析[J].海峡预防医学杂志，2019，25(05):46-48.

[1882]廖玫珍，刘冬莹，朱晓艳，等.山东省男男性行为人群单性性行为者与双性性行为者特征及 HIV 感染相关因素[J].中华流行病学杂志，2019(08):941-946.
[1883]张超，付钰淋，邹扬帆，等.陕西省 2008—2017 年 HIV 阳性男男性行为者特征分析[J].中国艾滋病性病，2019，25(11):1125-1128.
[1884]陆群，钱耀忠，陶力新，等.上海市奉贤区 MSM 人群艾滋病知识知晓、性行为及感染现况调查[J].健康教育与健康促进，2019，14(01):44-47.
[1885]殷方兰，钟培松，张永，等.上海市嘉定区男男性行为者 HIV 新发感染及队列保持影响因素研究[J].中国艾滋病性病，2019，25(09):947-950.
[1886]张贺礼，陈富讯，丁以标，等.上海市浦东新区男男性行为人群社会支持量表调查与分析[J].中国初级卫生保健，2019，33(05):69-71.
[1887]谢炜，赵锦，刘少础，等.深圳 1198 例移动 APP 交友型男男性行为者 HIV 感染状况及影响因素[J].中国艾滋病性病，2019，25(03):251-255.
[1888]安霞，钱兵，饶兆伟，等.深圳市宝安区 110 例 HIV 阳性男男性行为者的性行为特征和社会支持情况[J].中国艾滋病性病，2019，25(10):1047-1051.
[1889]吴秋红，陈威英，丁一，等.深圳市男男性接触人群梅毒合并 HIV 感染状况及其危险因素分析[J].中国麻风皮肤病杂志，2019，35(07):394-397.
[1890]徐晓华，福燕，宋兵，等.书写表达改善男男性行为人类免疫缺陷病毒/艾滋病患者自我歧视的效果研究[J].解放军护理杂志，2019，36(08):1-5.
[1891]王毅，周万明，樊静，等.四川省绵阳市男男性行为人群婚后同性性行为现状及其相关因素分析[J].中华流行病学杂志，2019(04):461-465.
[1892]高洁，傅卓华，赵秀萍，等.苏州市男男性行为者梅毒感染状况及影响因素研究[J].中国艾滋病性病，2019，25(04):392-394.
[1893]柏建芸，宁铁林，周宁，等.天津市 2016－2018 年哨点监测男男性行为人群 HIV 感染状况及相关因素分析[J].中华流行病学杂志，2019(09):1106-1110.
[1894]周宁，李环环，于茂河，等.天津市反复寻求 VCT 服务的男男性行为者相关性行为研究[J].中国艾滋病性病，2019，25(09):923-927.
[1895]徐晓华，福燕，宋兵，等.同伴支持降低男男性行为 HIV 感染者自我歧视的效果研究[J].中国护理管理，2019，19(12):1765-1769.
[1896]洪航，史宏博，姜海波，等.同性交友方式对男男性行为人群高危性行为及感染 HIV 的相关影响[J].中华流行病学杂志，2019(12):1612-1617.
[1897]张宁，余林，黄玉玲，等.同性恋和双性恋者的艾滋病相关行为流行病学调查[J].中国预防医学杂志，2019，20(11):1071-1075.
[1898]查旭东，王雯雯，商学军，等.网络社交软件对男男同性性行为人群 HIV 传播的影响[J].中华男科学杂志，2019，25(04):360-364.
[1899]靳大川，左雨点，武淑芳，等.我国男男性行为人群 HPV 感染率及其与 HIV 感染的关系[J].临床医学，2019，39(06):1-6.
[1900]妥小青，桑国耀，马汉叶勒丹.，等.乌鲁木齐市男男性行为者 HPVHBV 感染状况及影响因素[J].中国艾滋病性病，2019，25(3):256-259,293.
[1901]买买提木日扎提.，乃吉木孜克娅.，魏晶晶.乌鲁木齐市男男性行为者艾滋病高危性行为及影响因素分析[J].中国艾滋病性病，2019，25(11):1117-1119,1128.
[1902]李骏，陈懿，谭琳琳，等.梧州市 2010-2017 年男男性行为人群艾滋病流行状况分析[J].中国药物依赖性杂志，2019，28(05):386-389.
[1903]赵聪，王圣然，刘光耀，等.武汉市男男性行为大学生艾滋病知信行现状[J].中国学校卫生，2019，40(09):1399-1401.

[1904]王娜, 阮玉华.新确诊 HIV 阳性 MSM 一般自我效能与性伴的关系[J].中国艾滋病性病, 2019，25(06):606-608.
[1905]王娜，仇小强，阮玉华.新确诊 HIV 阳性男男性行为者一般自我效能与 HIV 相关羞辱的关系[J].中华疾病控制杂志，2019，23(12):1531-1534.
[1906]秦园.徐州市男男同性恋艾滋病患者风险因素调查[J].中国国境卫生检疫杂志，2019，42(01):59-61.
[1907]王憓，潘晓红，马瞧勤，等.应用 Rasch 模型对浙江省男男性行为者 HIV 感染相关性行为风险评估[J].中国艾滋病性病，2019，25(06):622-626.
[1908]韩瑜，谭婷，施玉华，等.云南省昆明市 427 例 HIV 抗体阳性男男性行为者生存质量及影响因素分析[J].卫生软科学，2019，33(03):85-89.
[1909]郑婵娟，马兰心，谭业丰，等.长沙市 MSM 艾滋病知识知晓情况、性行为现状分析[J].中华疾病控制杂志，2019，23(01):106-109.
[1910]陈剑，陈洁，谢知，等.长沙市部分男男性行为者中学生艾滋病高危行为特征分析[J].中国艾滋病性病，2019，25(11):1161-1163.
[1911]陈琳，罗明宇，王憓，等.浙江省 138 名 HIV 阳性男男性行为者性伴 HIV 检测行为及感染情况[J].中国艾滋病性病，2019，25(05):464-467.
[1912]倪志侃，罗明宇，潘晓红，等.浙江省男男性行为人群 HIV 感染者成功动员性伴检测情况及相关因素分析[J].中华流行病学杂志，2019(12):1606-1611.
[1913]乔丹，刘征.郑州市 2014-2018 年男男性行为人群同性安全性行为影响因素[J].江苏预防医学，2019，30(05):510-513.
[1914]王恒杰, 唐日新.中国同性恋人群心理健康研究综述[J].校园心理, 2019, 17(03):201-204.
[1915]辛辛，张勇，周晓林，等.中青年男男性行为者高危行为特征及 HIV 检测现状[J].中国热带医学，2019，19(02):141-144.
[1916]王东方，周柏，高钰潋，等.191 例男男性行为者的 HIV 感染者儿童期创伤对其精神病性体验的影响[J].中国艾滋病性病，2020，26(03):286-289.
[1917]李玮，黄珊子，姚刚，等.2002-2018 年珠海市 MSM 人群艾滋病疫情特征分析[J].热带医学杂志，2020，20(02):253-256.
[1918]周玲米，周芸，汤杰，等.2012—2016 年桂林市男男性行为人群艾滋病哨点监测结果分析[J].应用预防医学，2020，26(01):73-76.
[1919]贾晓飞，赖文健，张宏，等.2016-2018 年广州市男性同性恋人群抑郁症状与自杀意念的关联分析[J].预防医学情报杂志，2020，36(04):371-375.
[1920]张兴亮，罗艳，丁建明，等.2017 年杭州市新确诊男男性行为人群 HIV/AIDS 病例特征分析[J].预防医学，2020，32(04):373-377.
[1921]王贞玉，卢雍，孟晓军，等.HIV 感染高风险男男性行为人群合成毒品使用现况及相关因素分析[J].中华流行病学杂志，2020(02):231-235.
[1922]陈菊娣, 任勇, 阎岩, 等.MSM 家族集聚现象心理状况的质性研究[J].护理研究, 2020, 34(01):142-145.
[1923]孙燕鸣，徐敏，李桂英，等.北京市男男性行为者梅毒预防知识知晓情况及影响因素分析[J].中国艾滋病性病，2020，26(02):187-190.
[1924]黄道平，池媛媛，周婧，等.常德和张家界两市 HIV 阳性已婚男男性行为者性行为状况分析[J].中国艾滋病性病，2020，26(03):281-285.
[1925]陆春燕，蒋莉，黄金萍，等.个案管理在高水平 CD_4~+T 淋巴细胞的男男性行为 HIV 感染者中的应用[J].护理研究，2020，34(05):888-890.
[1926]刘汉涛，朱黎丹，张贺礼，等.基于 BLUED 软件的 MSM 人群线上交流与线下行为状

况调查[J].海南医学，2020，31(05):670-673.
[1927]秦小芬，李现红，王红红，等.基于健康信念模型探讨社群亚文化对男男性行为者高危性行为的影响[J].中南大学学报(医学版)，2020，45(01):55-60.
[1928]王毅，李六林，樊静，等.绵阳市男男性行为者参加艾滋病防治社区活动及关联因素分析[J].中国艾滋病性病，2020，26(03):277-280.
[1929]康媛媛，李胜，向芳，等.男男性接触人群网络交友现状及特征分析[J].中国健康教育，2020，36(03):250-254.
[1930]王晓丹，周昕雅，张晋铭，等.男男性行为人群合成毒品滥用质性访谈调查[J].中国药物滥用防治杂志，2020，26(02):118-120.
[1931]王毅，涂浩天，周万明，等.男男性行为者艾滋病风险认识与知识和高危性行为关联性[J].华南预防医学，2020，46(01):10-13.
[1932]肖绍坦，陈盼盼，辛辛.男男性行为者定期艾滋病咨询检测动机及态度的定性研究[J].中国艾滋病性病，2020，26(03):308-309.
[1933]胡健，何孟芹.青年男同性恋者/男双性恋者购买性商业同性性行为的社会文化因素[J].中华疾病控制杂志，2020，24(01):105-108.
[1934]黄玉玲，余彬，杨诗凡，等.青年学生男男性行为者非固定性伴性行为特征及与社会规范的关系[J].中国艾滋病性病，2020，26(02):175-178.
[1935]陈威英，吴秋红，丁一，等.深圳男男性接触者多性伴与社交软件使用习惯现况及相关性分析[J].中国麻风皮肤病杂志，2020，36(01):24-27.
[1936]付楠楠，于茂河，柳忠泉，等.天津市 HIV 阳性 MSM 生活状况及社会支持情况定性访谈分析[J].职业卫生与病伤，2020，35(02):88-91.
[1937]艾克丹·塔西，马媛媛，胡晓远，等.乌鲁木齐市男男性行为人群 HIV 新发感染及影响因素分析[J].预防医学，2020，32(03):278-281.

***Theses***

[1]朱明泉.中国大陆男男性接触者人口学因素与艾滋病高危行为关系的研究[D].青岛大学，2002.
[2]吴绍文.不同自我认同的男男性接触者艾滋病高危行为监测与比较[D].青岛大学，2003.
[3]曲鹏.对男性同性恋者预防艾滋病不同干预模式的效果及其比较[D].中国医科大学，2004.
[4]谷渊.沈阳市艾滋病高危人群风险评估[D].中国医科大学，2004.
[5]曾春娥.男男性接触群体的 HIV/AIDS 预防策略研究[D].华中师范大学，2005.
[6]刘明华.江苏省男男性接触人群人口学因素与艾滋病高危行为关系的研究[D].青岛大学，2005.
[7]潘传波.重庆市中心城区不同人群预防艾滋病知识与行为现况调查研究[D].第三军医大学，2005.
[8]郑迎军.年轻的 MSM 人群高危性行为及梅毒和艾滋病感染状况[D].安徽医科大学，2006.
[9]安全平.男男性接触者性别角色自我认定与艾滋病高危行为关系的研究[D].青岛大学，2006.
[10]李辉.中国六城市男男性接触者高危行为与性传播疾病的相关关系研究[D].青岛大学，2006.
[11]邢建民.男男性行为（MSM）人群对中国艾滋病流行的影响：一项以网络为平台的 MSM 人群调查研究[D].中国协和医科大学，2007.
[12]廖留妹.男性接触者中男性性工作者与非男性性工作者艾滋病相关因素对比研究[D].青岛大学，2007.

[13]汤后林.男男性接触者性网络特征及艾滋病病毒感染危险行为研究[D].中国疾病预防控制中心，2007.
[14]窦明阳.长春市男男性接触者艾滋病知识、行为调查[D].吉林大学，2007.
[15]张寒冰.太原市 MSM 人群预防艾滋病干预效果的评价及不同干预模式的比较[D].山西医科大学，2007.
[16]王王.太原市 MSM 人群高危行为的主要影响因素研究[D].山西医科大学，2007.
[17]张学化.济南市男男性接触者艾滋病知识态度行为的调查与分析研究[D].山东大学，2007.
[18]李新旭.北京市男男性接触者高危行为和 HIV/梅毒感染调查研究[D].中国疾病预防控制中心，2007.
[19]郭巍.自愿咨询检测对廊坊市男男性行为人群艾滋病相关危险行为影响的随访研究[D].中国疾病预防控制中心，2008.
[20]邹华春.使用互联网的男男性行为者性行为特征与艾滋病感染影响因素研究[D].中国疾病预防控制中心，2008.
[21]林可.中国同性恋网站性健康干预现状与对策研究[D].复旦大学，2008.
[22]周璐璐.同性恋者的心理弹性研究[D].华东师范大学，2008.
[23]郭剑.男同性恋艾滋病危险感知影响因素结构方程模型分析[D].天津医科大学，2008.
[24]朱军礼.年轻 MSM 社会网络特征及其 HIV 危险行为同伴干预研究[D].安徽医科大学，2008.
[25]郑建东.北京市大学在校生男男性接触者艾滋病相关高危行为及其影响因素研究[D].中国疾病预防控制中心，2008.
[26]孙修福.山东某地区男男性接触者的高危行为研究[D].山东大学，2008.
[27]杨旭.重庆市男男性接触人群艾滋病高危行为相关因素研究[D].第三军医大学，2008.
[28]章任重.昆明市男男性接触者 HIV/AIDS 感染现状及相关问题研究[D].昆明医学院，2008.
[29]张志强.长春市男男性接触者 HIV 和梅毒感染率及性行为的研究[D].吉林大学，2008.
[30]蔡勇.男男性行为人群艾滋病行为干预效果评价[D].吉林大学，2009.
[31]刘昆仑.男男性行为人群安全套使用与需求研究[D].中国疾病预防控制中心，2009.
[32]李东亮.男男性行为人群包皮环切术和艾滋病疫苗接种意愿调查研究[D].中国疾病预防控制中心，2009.
[33]朱炜明.浙江省台州市男男性行为人群 HIV/STI 感染、危险行为与性关系网络研究[D].复旦大学，2009.
[34]李雪华.武汉市男男性行为人群性病、艾滋病流行病学调查[D].华中科技大学，2009.
[35]刘芳.重庆市高校男男性行为者艾滋病知识行为网络调查研究[D].第三军医大学，2009.
[36]于增照.男男性行为者儿童期性虐待经历对艾滋病高危行为及心理影响[D].青岛大学，2009.
[37]刘运泳.辽宁省 MSM 人群精神疾病与高危性行为关系研究[D].大连医科大学，2009.
[38]修翠珍.男男性行为人群的艾滋病行为干预与感染状况研究[D].山东大学，2009.
[39]陈舸.男男性接触人群中与艾滋病相关的危险行为及感染状况调查研究[D].福建医科大学，2009.
[40]李清春.北京市男男性行为人群前瞻性队列研究[D].中国协和医科大学，2009.
[41]孙国清.郑州市男男性接触人群艾滋病流行趋势及影响因素分析[D].郑州大学，2009.
[42]秦韬.重庆市男同性恋者生活状况及主观幸福感调查研究[D].西南大学，2009.
[43]李启凤.对广西 L 市同性恋人群生活状况的调查与研究[D].广西师范大学，2009.
[44]李春梅.重庆市男男性行为人群艾滋病病毒和梅毒感染情况调查及影响因素分析[D].河北医科大学，2009.

[45]许娟.四城市男男性行为人群性伴特征与艾滋病病毒/梅毒感染状况[D].中国疾病预防控制中心，2010.
[46]王硕.HIV 阳性男男性行为者自身阳性状况知晓与性行为特征的关联[D].中国疾病预防控制中心，2010.
[47]夏俊瑞.七城市同性恋酒吧中 MSM 人群高危性行为及其生活质量调查[D].安徽医科大学，2010.
[48]唐卫明.用同伴推动抽样法对南京市男男性接触人群进行抽样调查[D].南京医科大学，2010.
[49]王招福.中医药防治艾滋病概况及哈尔滨市 MSM 艾滋病高危行为调查分析[D].黑龙江中医药大学，2010.
[50]米国栋.成都市男男性行为人群艾滋病病毒感染者求医行为及高危行为干预研究[D].中国疾病预防控制中心，2010.
[51]庾泳.长沙市区男同性恋人群生存状况研究[D].中南大学，2010.
[52]郭卉.北京地区在婚男男性接触者性行为特征调查[D].中国疾病预防控制中心，2010.
[53]张建.乌鲁木齐市男男性接触者艾滋病综合干预效果的评价研究[D].新疆医科大学，2010.
[54]蓝光华.南宁市男男性行为队列人群 HIV 感染相关因素的研究[D].广西医科大学，2011.
[55]苏莹珍.MSM 人群 HIV-1 感染的影响因素及云南省 HIV-1 分子流行病学研究[D].南方医科大学，2011.
[56]张燕.中国西部男男性行为人群艾滋病暴露前预防用药的知晓及使用意愿动机模型研究[D].重庆医科大学，2011.
[57]于海荣.聊城市男男性行为人群艾滋病危险行为及其影响因素研究[D].山东大学，2011.
[58]沈晓沛.中国西南地区男男性行为人群“感染状态配对”现况调查[D].中国疾病预防控制中心，2011.
[59]李建军.南京市男男性行为人群高危行为干预和队列研究[D].南京医科大学，2011.
[60]郭燕.天津市艾滋病高危人群 HIV 和梅毒感染状况及行为特征分析[D].天津医科大学，2011.
[61]佘颖.基于保护动机理论男男性行为人群安全套使用的研究[D].重庆医科大学，2011.
[62]杜桂英.聊城市男男性行为者艾滋病高危行为及其影响因素调查研究[D].山东大学，2011.
[63]魏锁.年轻男男性行为人群 HIV 和梅毒感染状况及其相关因素[D].安徽医科大学，2011.
[64]段毓雯.男男性行为人群预防控制艾滋病同伴综合干预方法研究[D].安徽医科大学，2011.
[65]宇方.MSM 人群 HIV 高危行为同伴群干预研究[D].安徽医科大学，2011.
[66]古龙梅.女同性恋社会支持研究[D].四川省社会科学院，2011.
[67]赵淑娟.许昌市男男性行为人群艾滋病高危行为及影响因素分析[D].郑州大学，2011.
[68]李思遥.辽宁省男男性接触者同性身份认同与内在感受的关系研究[D].大连医科大学，2011.
[69]李峰.社会资本与男男性行为人群艾滋病相关知识、态度和行为的关系研究[D].安徽医科大学，2011.
[70]张重辉.临汾市男男性接触人群 HIV、TP 及 HCV 感染现状及性行为特征调查[D].山西医科大学，2011.
[71]易柳.同性恋者身份认同、对社会态度的感知与心理健康的关系研究[D].广州大学，2011.
[72]刘英杰.北京市女女性行为人群感染 HIV/STI 危险因素的流行病学调查研究[D].中国疾病预防控制中心，2011.
[73]卢戎戎.重庆市男男性行为人群艾滋病流行趋势及影响因素研究[D].第三军医大学，2011.
[74]冯英.云南省曲靖市男男性行为者艾滋病高危行为调查研究[D].第三军医大学，2011.

[75]徐静.重庆市男男性接触人群艾滋病相关知识、行为学调查及干预效果研究[D].第三军医大学，2011.
[76]范颂.北京市男男性行为人群艾滋病流行病学研究[D].中国疾病预防控制中心，2011.
[77]陈韵.佛山市男男性行为人群自杀未遂研究[D].广东药学院，2011.
[78]于茂河.天津市男男性行为 HIV-1 感染者行为特征与基因亚型、耐药性分析[D].天津医科大学，2011.
[79]席胜军.杭州市男男性行为人群艾滋病防治知识、行为特征、HIV 感染及影响因素研究[D].浙江大学，2011.
[80]柳忠泉.天津市男男性行为人群 HIV 和梅毒感染风险的研究[D].天津医科大学，2011.
[81]刘立珍.成都市男男性行为者艾滋病相关羞辱和歧视的调查研究[D].中南大学，2011.
[82]袁巧菊.开封市 MSM 艾滋病相关知识、态度、行为调查分析[D].河南大学，2011.
[83]于明润.北京市男男性行为者 HIV/STDS 流行病学研究[D].苏州大学，2011.
[84]王珏.男男性行为人群中不同性角色艾滋病性病影响因素研究[D].中国疾病预防控制中心，2012.
[85]李雪峰.北京市男男性行为者 HIV 检测及结果性伴告知情况的调查研究[D].中国疾病预防控制中心，2012.
[86]王小芳.北京市女女性行为人群性传播感染及其相关因素的研究[D].中国疾病预防控制中心，2012.
[87]张宇鑫.同性恋者的压力应对方式及其与社交回避及苦恼的研究[D].西南大学，2012.
[88]薛黎坚.男男性行为人群高危行为及影响因素研究[D].苏州大学，2012.
[89]韦所苏.广西男男性行为者与女性性工作者对暴露前药物预防 HIV 感染的接受意愿及其影响因素研究[D].广西医科大学，2012.
[90]王朝才.青海省西宁市男男性行为人群 HIV 感染状况及危险因素研究[D].山西医科大学，2012.
[91]刘珺.佛山市男男性行为者抑郁、焦虑症状现况及关联因素研究[D].广东药学院，2012.
[92]王永香.山东省男男性行为者性行为、安全套使用及 HIV/syphilis 感染研究[D].济南大学，2012.
[93]陈鑫.中国部分地区男男性行为人群艾滋病流行现况及趋势研究[D].南京医科大学，2012.
[94]柏建芸.天津市艾滋病高危人群的行为特征和 HIV-1 感染状况研究[D].天津医科大学，2012.
[95]李健.甘肃省性传播疾病高危人群相关疾病感染、行为状况及影响因素回归分析[D].兰州大学，2012.
[96]董秀平.男男性行为人群梅毒感染影响因素分析及治疗措施研究[D].青岛大学，2012.
[97]任晖琴.乌鲁木齐市男男性行为人群前瞻性队列研究[D].新疆医科大学，2012.
[98]高彦杰.北京市男男性行为人群中 HIV 等性传播疾病前瞻性队列研究[D].河北医科大学，2012.
[99]周爽.重庆市男男性行为人群艾滋病性病感染率及其影响因素研究[D].第三军医大学，2013.
[100]韩扬.北京市男男性行为者艾滋病流行病学调查研究[D].中国疾病预防控制中心，2013.
[101]王娜.重庆市男男性行为者 HIV 感染调查和阳性传播预防的 Meta 分析[D].中国疾病预防控制中心，2013.
[102]高从.男男性行为人群 HIV/HHV-8 合并感染的流行病学研究[D].北京协和医学院，2013.
[103]刘雅琼.高危性行为人群性传播疾病暴露风险及危害新问题的研究[D].第三军医大学，2013.

[104]刘湘林.沙盘游戏疗法在同性恋个案临床干预中的应用研究[D].上海师范大学，2013.
[105]徐洪吕.男男性行为人群 HIV 感染病例对照研究[D].昆明医科大学，2013.
[106]刘亚涛.南昌市男男性接触人群调查及其相关问题研究[D].南昌大学，2013.
[107]汪敏.上海市男男性行为人群 HIV 感染者健康状况及卫生服务利用现况调查[D].安徽医科大学，2013.
[108]宋丹丹.男性同性兼异性性行为者 HIV 感染状况及性行为特征[D].安徽医科大学，2013.
[109]蒋均.在场所活动的男男性行为人群 HIV 检测情况及其影响因素研究[D].安徽医科大学，2013.
[110]曹臻.在婚男男性行为者 HIV 和梅毒感染状况及其相关因素[D].安徽医科大学，2013.
[111]董旭峰.银川市男男性行为人群 HIV 高危行为流行病学调查及干预效果评价[D].宁夏医科大学，2013.
[112]胡玲玲.河北省省会男男性行为人群感染艾滋病相关高危行为研究[D].河北医科大学，2013.
[113]齐啸.北京市男男性行为者 HIV 感染和固定性伴检测研究[D].河北医科大学，2013.
[114]李琳.东北某市男男性行为人群HIV感染状况及相关行为的调查研究[D].吉林大学，2014.
[115]徐里强.常熟市 MSM 人群特征、STD 及其影响因素检出情况的研究[D].苏州大学，2014.
[116]何悦齐.大学生男同性恋者压力问题研究[D].云南师范大学，2014.
[117]谢娟.女同性恋者社会支持、自我效能感与心理健康的关系研究[D].四川师范大学，2014.
[118]程旭.男男同性恋 PLWHA 心理健康状况及干预策略研究[D].南昌大学，2014.
[119]严华美.上海市男男性行为人群艾滋病感染风险及分子流行病学研究[D].复旦大学，2014.
[120]孙炀明.昆明市 MSM 人群 HIV 新发感染及无保护性肛交影响因素分析[D].昆明医科大学，2014.
[121]熊杨.动机访谈在男男性行为人群高危性行为干预中的应用研究[D].中南大学，2014.
[122]何佳桐.HIV 阴性高风险 MSM 人群的筛选及其 PrEP 用药意愿分析[D].重庆医科大学，2014.
[123]熊明洲.男同性恋者的心理社会特征研究[D].中南大学，2014.
[124]张东妍.深圳市 MSM 人群 HPV 感染及高危行为的流行病学研究[D].北京协和医学院，2014.
[125]吴庆涛.同性恋压力问卷的编制及其调查研究[D].上海师范大学，2014.
[126]李新蕊.济南市男男同性性行为者社会支持、应对方式和主观幸福感的现况调查及相关性研究[D].山东大学，2014.
[127]董振鑫.年轻 MSM 人群 HIV 和梅毒新发感染状况及其影响因素[D].安徽医科大学，2014.
[128]陈继军.兰州市男男性行为人群艾滋病流行病学研究[D].兰州大学，2014.
[129]李欣格.北京市女女性行为人群定性研究与性传播疾病的感染状况[D].河北医科大学，2014.
[130]白雪.男男性行为人群 HIV 感染者确诊前后性行为研究[D].安徽医科大学，2014.
[131]许瑜楠.北京市男男性行为者 HIV 检测情况和口腔唾液快速检测需求的调查研究[D].中国疾病预防控制中心，2014.
[132]龙其穗.湛江市活动型男男性行为人群艾滋病现况调查及规模估计[D].暨南大学，2015.
[133]韦丽.山东省女同性恋人群的心理健康状况及行为特征研究[D].山东大学，2015.
[134]李真.HIV/AIDS 患者感知歧视现状调查及影响因素研究[D].北京协和医学院，2015.
[135]李臻.西安市男男性行为人群 HIV 感染者合并感染 HPV、TB 的流行病学调查[D].北京协和医学院，2015.
[136]付春静.MSM 人群的用药依从性对 PrEP 预防 HIV 新发感染的效果影响及其保护动机

分析[D].重庆医科大学，2015.
[137]杨慧云.MSM 社群内艾滋病相关羞辱和歧视研究[D].安徽医科大学，2015.
[138]戴浈浈.男性同性兼异性性行为人群特征及其 HIV 感染的影响因素分析[D].重庆医科大学，2015.
[139]何欢.有高危性行为的 HIV 阳性 MSM 性传播行为状况及其干预对策研究[D].安徽医科大学，2015.
[140]孙扬.MSM 人群高危性行为、生存质量与社会支持状况研究[D].大连医科大学，2015.
[141]党静.石家庄市男男性行为人群艾滋病知识、态度、行为调查及感染状况分析[D].河北医科大学，2015.
[142]刘岩岩.合肥市男男性行为人群 HIV 感染状况及其影响因素研究[D].安徽医科大学，2015.
[143]张昭.影响新疆 MSM 人群预防 HIV 医疗资源利用障碍的研究[D].新疆医科大学，2015.
[144]王森路.乌鲁木齐市男男性行为人群 HIV 感染状况及影响因素研究[D].新疆医科大学，2015.
[145]周云华.北京市男男性行为人群 HIV 和梅毒感染情况及其固定性伴检测干预效果研究[D].苏州大学，2015.
[146]刘云.男男性行为者艾滋病高危行为评估及心理干预[D].南昌大学，2016.
[147]张恒.北京市 MSM 人群 HIV 高危行为情况和抗病毒治疗病毒抑制的现场试验研究[D].中国疾病预防控制中心，2016.
[148]王玉淼.高校学生男男性行为人群性行为特征及 HIV 影响因素分析[D].昆明医科大学，2016.
[149]胡珊.MSM 人群焦虑抑郁的共病性及其与 HIV 高危行为的关系[D].重庆医科大学, 2016.
[150]曾馨.我国西部地区 HIV 阴性 MSM 人群暴露前预防用药预防 HIV 新发感染的有效性研究[D].重庆医科大学，2016.
[151]荆少华.基于结构方程模型的男男性行为接触者焦虑、抑郁症状影响因素研究[D].重庆医科大学，2016.
[152]訾桂玲.山东省男男性行为者 HIV/STD 感染状况及危险因素分析[D].济南大学，2016.
[153]李真晖.昆明市同性恋浴池内 MSM 人群 HIV 感染率和影响因素研究[D].昆明医科大学，2016.
[154]李琪.HIV 阳性男男性行为者性伴动员检测可行性研究[D].昆明医科大学，2016.
[155]王平.青年学生男男性行为者心理健康状况及影响性取向成因的因素[D].天津医科大学，2016.
[156]张越.北京市 2012-2014 年男男性行为者艾滋病和梅毒新发感染状况的队列分析[D].吉林大学，2016.
[157]姜袁.基于定性访谈及结构方程模型的 MSM 艾滋病预防行为影响因素分析[D].新疆医科大学，2016.
[158]陈芳.HIV 阳性男男性行为者性伴告知现状及其影响因素分析[D].安徽医科大学，2016.
[159]何慧婧.影响男性同性性行为人群艾滋病传播的社会文化因素定性研究[D].中国疾病预防控制中心，2017.
[160]任仙龙.北京市男男性行为人群 HIV 自我检测对求询检测行为及高危性行为的影响[D].中国疾病预防控制中心，2017.
[161]刘磊.HIV 阳性男男性行为人群合并性病感染现状及其影响因素的调查研究[D].中国疾病预防控制中心，2017.
[162]曾梓.广西部分地区 MSM 人群对 HIV 非职业暴露后预防的需求及其影响因素研究[D].

中国疾病预防控制中心，2017.
[163]赵丹鹤.2011-2016年北京市年轻男男性行为者HIV和梅毒新发感染情况的队列研究[D].吉林大学，2017.
[164]靳伟.HIV 阳性男男性行为者应用自检试剂促进性伴检测意愿及影响因素研究[D].广东药科大学，2017.
[165]李云芳.同性恋者的风险认知、心理健康与风险行为及其媒介影响因素研究[D].暨南大学，2017.
[166]韩琳.渭南市男男性行为人群 HIV 感染现状及影响因素研究[D].山西医科大学，2017.
[167]王念.MSM 人群对 PrEP 干预策略的接受意愿及影响因素研究[D].重庆医科大学，2017.
[168]王莹.湖北省男男性行为人群艾滋病干预效果评估及信息-技巧-行为模式下的干预模型研究[D].武汉大学，2017.
[169]蒙柳仁.南宁市 MSM 人群拥有同性固定性伴的影响因素及心理特征分析用[D].广西医科大学，2017.
[170]蒋和宏.重庆市使用新型毒品的男男性行为者艾滋病感染状况及影响因素研究[D].重庆医科大学，2017.
[171]罗淑星.男男性行为人群艾滋病患者心理状况与性行为影响因素的研究[D].第三军医大学，2017.
[172]郑杰滔.高校在校学生男男性行为人群行为学特征及影响因素研究[D].山东大学，2017.
[173]董安琪.男男性行为人群中跨性别群体艾滋病防治需求及传播风险研究[D].山东大学，2017.
[174]李润华.浙江省男男性行为人群 HIV 感染、检测及其影响因素的研究[D].宁波大学，2017.
[175]金莹莹.基于社交软件的大学生男男性行为者 HIV 检测现状及相关因素研究[D].安徽医科大学，2017.
[176]彭锋.运用同伴推动抽样法进行男男性行为人群 HIV 流行现状及危险因素的调查研究[D].河北医科大学，2017.
[177]张月.HIV 阳性男男性行为者多性伴传播行为状况及其影响因素分析[D].安徽医科大学，2017.
[178]刘乃鹏.HIV 阳性男男性行为者性传播危险状况调查分析[D].安徽医科大学，2017.
[179]吕林芳.北京市 MSM 人群 HIV 新发感染及固定性伴唾液检测试剂自检情况调查研究[D].河北医科大学，2017.
[180]田恬.男男性行为人群人乳头瘤病毒相关认知及感染情况调查[D].新疆医科大学，2017.
[181]杨娟.广州市男男性行为人群社会网络特征及其与 HIV 传播的关系[D].中国疾病预防控制中心，2018.
[182]卢姗.男男性行为人群药物滥用与无保护性肛交行为关系研究[D].中国疾病预防控制中心，2018.
[183]王娜.新确诊 HIV 阳性 MSM 一般自我效能与抑郁、焦虑、羞辱歧视和性行为的关系[D].广西医科大学，2018.
[184]陈晓滨.基于整合 IMB 模型的男男性行为者安全性行为研究[D].广东药科大学，2018.
[185]宋媛媛.基于非政府组织的长春市 MSM 人群 HIV、梅毒新发阳性率及影响因素分析[D].吉林大学，2018.
[186]高晓娟.昆明市男男性行为者抑郁焦虑现况及影响因素调查[D].昆明医科大学，2018.
[187]韩娜.天津市男男性行为人群新型毒品滥用情况及其影响因素分析[D].天津医科大学，2018.
[188]李镠.天津市男男性行为人群新型毒品使用和 HIV 感染状况调查[D].天津医科大学，

2018.
[189]王泽洲.上海市部分男男性行为者多性伴行为及相关社会心理因素研究[D].上海交通大学，2018.
[190]唐慧玲.金华市男男性行为人群艾滋病知信行现状和定期随访干预效果评估[D].浙江大学，2018.
[191]薛莉.HIV 检测自我效能和预期污名量表信效度分析及对 MSM 人群 HIV 检测影响[D].山东大学，2018.
[192]李梦.哈尔滨市男男性行为者 HIV 感染与药物使用相关因素研究[D].烟台大学，2018.
[193]李一苇.性少数群体亲密关系影响因素与期望研究[D].南京理工大学，2018.
[194]乔丹.男性同性恋自我认同与身份压力对安全性行为的影响[D].南京师范大学，2018.
[195]陶毓敏.宁波市 HIV 阴性 MSM 人群艾滋病知识知晓、行为特征及影响因素研究[D].安徽医科大学，2018.
[196]韩晶.男男性行为青年学生 HIV 感染者性行为特征研究[D].中国疾病预防控制中心，2019.
[197]王芳.HIV 阳性的男男同性性行为者创伤后成长的影响因素分析[D].河北大学，2019.
[198]闫莉.南京市男男性行为人群互联网+HIV 自我检测干预效果队列研究[D].东南大学，2019.
[199]魏然.男男性行为人群对 HIV 暴露后预防服务的接受程度及影响因素研究[D].山东大学，2019.
[200]黎静.社会心理行为因素对男男性行为者高危性行为的共疫作用研究[D].广东药科大学，2019.
[201]黄丹.男男性行为人群艾滋病暴露前预防用药依从性影响因素及其对策研究[D].重庆医科大学，2019.
[202]屈豆.基于信息-动机-行为技巧理论男男性行为者暴露前预防用药依从性研究[D].重庆医科大学，2019.
[203]胡莹.PrEP 策略下 MSM 人群障碍与益处感知与依从性的关系研究[D].重庆医科大学，2019.
[204]李环环.天津市男男性行为人群无保护性肛交及其影响因素的研究[D].天津医科大学，2019.
[205]李佳明.医学辅助策略推动 HIV 阳性 MSM 性伴告知的随机对照实验研究[D].中国医科大学，2019.
[206]王若曦.中国西南三地男男性行为者丙型肝炎知识、行为及健康教育的现况调查[D].重庆医科大学，2019.
[207]段青.山东省男男性行为人群新型毒品滥用现状及 HIV、梅毒感染情况调查研究[D].济南大学，2019.
[208]杨蓓.重庆市某区县男男性行为者 HIV 新发感染及影响因素研究[D].重庆医科大学，2019.
[209]张德川.三城市学生男男性行为者高危性行为状况及相关因素研究[D].安徽医科大学，2019.
[210]毛翔.基于 HIV 自我检测策略对中国 MSM 人群开展 HIV 疫情监测及检测促进的干预效果研究[D].中国医科大学，2019.
[211]赵越.男男性行为人群唾液 HIV 自我检测与咨询服务模式研究[D].安徽医科大学，2019.
[212]朱晓芳.基于微信开展男男性行为人群 HIV 自我检测服务效果研究[D].安徽医科大学，2019.

[213]朱亚鑫.男男性接触人群艾滋病高危行为影响因素分析及干预效果评价研究[D].中国医科大学，2019.
[214]彭二磊.学生 MSM 人群 HIV 新发感染率及影响因素的前瞻性队列研究[D].中国医科大学，2019.
