# Supplementary material for: The Holistic Health Status of Chinese Homosexual and Bisexual Adults: A Scoping Review
Source: Front Public Health. 2021 Aug 24;9:710575. doi: 10.3389/fpubh.2021.710575 (PMC8421524; doi:10.3389/fpubh.2021.710575)
Supplement: Supplementary file 6 [file Data_Sheet_6.pdf]

Quality evaluation of journals where all included articles published

This review evaluated quality of 2645 articles (2478 research papers and 167 reviews), but not the 234 theses.

Of all articles, 694 English (EN) articles were published in 152 EN journals, 1937 simplified Chinese (SC) articles were published in 181 SC journals, and 14 traditional Chinese (TC) articles were published in 9 TC journals.

- Regarding the quality of English journals, their impact factor (IF) according to the Journal Citation Reports (JCR, https://jcr.clarivate.com) have been searched.
- For simplified Chinese journals, there are three most scientific and commonly used journal evaluation indicators in universities, namely Chinese Science Citation Database (中国科学引文数据库 CSCD, http://sciencechina.cn/cscd_source.jsp), Chinese core journal criterion of PKU (中文核心期刊, http://hxqk.lib.pku.edu.cn/), and the Key magazine of China technology (中国科技核心 CSTPCD, https://www.istic.ac.cn/isticcms/html/1/284/338/6206.html). Accordingly, those journals which are included in the above three lists would be considered as core journals; if not, then their Chinese impact factors would be searched.
- While for traditional Chinese journals, there were no available assessment methods or evaluation indicators.

According to the latest Chinese Core Journals criteria and JCR's Impact factors, articles published in core Chinese journals or English journals with IF greater than 2 are considered to be relatively high quality, accounting for 77.54% of this review (Table below).

Table Quality assessment of all journals of published articles

**Characteristics**		**No. of Journal**	**No. of Publication**	**Percentage (%)**
Simplified CN journal				
SC Core Journal [a]		106	1579	*59.70%*[b]
Non-core Journal	CN-IF: 0.03-0.50	48	141	5.33%
	CN-IF: 0.51-1.00	19	188	7.11%
	CN-IF: 1.01-2.31	8	29	1.10%
Traditional CN Journal				
	Not available	9	14	0.53%
English journal				
Impact factor (2019)	IF: 0.25-1.00	8	13	0.49%
	IF: 1.01-2.00	42	158	5.97%
	IF: 2.01-3.00	31	246	*9.30%*[b]
	IF: 3.01-4.00	29	188	*7.11%*[b]
	IF: 4.01-10.00	14	31	*1.17%*[b]
	IF: 10.01-60.39	6	7	*0.26%*[b]
	No IF	22	51	*1.93%*[b]
**In sum**		**342**	**2645**	***77.54%***[b]

[a]: References published in relatively high-quality journals.

[b]: Relatively high-quality journals and percentages.
