# Supplementary material for: The Holistic Health Status of Chinese Homosexual and Bisexual Adults: A Scoping Review
Source: Front Public Health. 2021 Aug 24;9:710575. doi: 10.3389/fpubh.2021.710575 (PMC8421524; doi:10.3389/fpubh.2021.710575)
Supplement: Supplementary file 7 [file Data_Sheet_7.pdf]

Co-word Analysis of Keywords and Detailed Concept characteristics

Frequency of keywords in the included publications

Frequency of keywords in both included English and Chinese publications

**Chinese** (N> 50)	**F**	**Both** (N≥ 50)	**F**	**English** (N≥ 15)	**F**
男男性行为者 (MSM)	1572	Men who have Sex with Men (MSM)	1908	Men who have Sex with Men (MSM)	372
艾滋病	820	HIV	723	China	226
HIV	548	AIDS	607	HIV	174
影响因素	312	Syphilis	261	Syphilis	40
梅毒	266	China	224	HIV/AIDS	32
性行为	189	Influencing Factor	219	HIV Testing	25
高危行为	174	HIV/AIDS	193	Sexual Behavior	24
同性恋	139	Sexual Behavior	164	Sexual Orientation	23
男男性行为	136	High Risk Behavior	122	Stigma	22
AIDS艾滋病	134	Risk Factor	117	Condom Use	21
危险性行为	125	Male Homosexuality	98	Gay	20
男性	109	Knowledge	66	Risk Behavior	19
感染状况	105	STI	65	Depression	19
行为	99	High Risk Sexual Behavior	65	Pre-Exposure Prophylaxis (PrEP)	19
性传播疾病	85	Behavior	63		
危险因素	83	Homosexuality	58	Internet	19
安全套	80	Prevalence	57	STI	17
知识	77	Condom	54	Homosexuality	17
哨点监测	58	Depression	51	HIV Prevention	17
HIV病毒感染	52	Infection	51	Mental Health	16
干预	51	HIV Infection	50		

Co-word Analysis of Chinese Keywords (N=10)

Among all Chinese publications with keywords, all keywords appeared 10 times or more in the co-cooccurrence matrix have been involved in the co-word analysis.

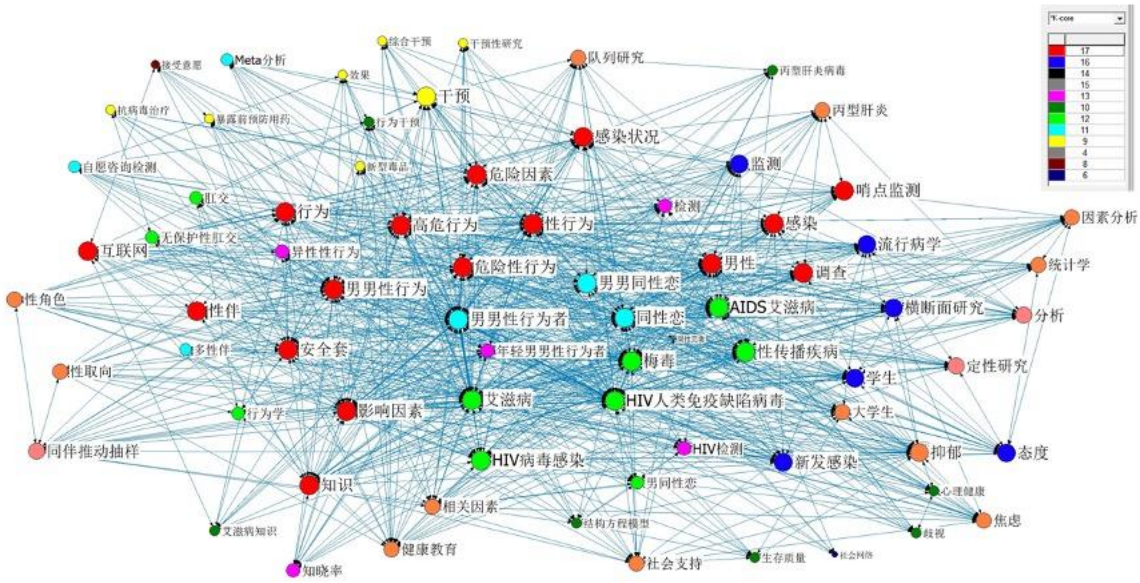

Figure 1 Co-word Analysis of Chinese Keywords (N=10)

Co-word Analysis of English Keywords (N=5)

Among all English publications with keywords, all keywords appeared 5 times or more in the co-cooccurrence matrix have been involved in the co-word analysis.

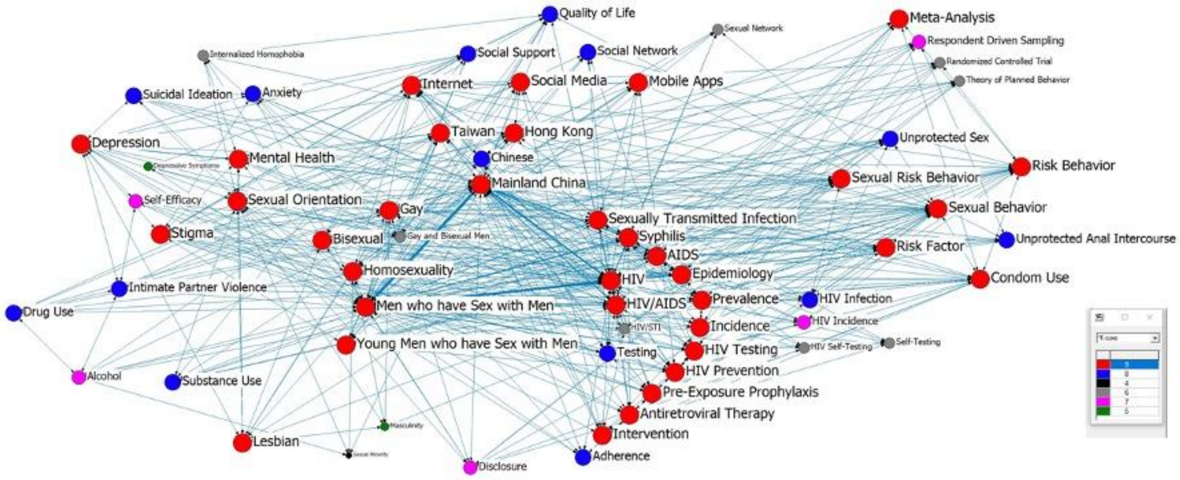

Figure 2 Co-word Analysis of English Keywords (N=5)

Comparison of study focus of different gender groups

Detailed and specific variables used in English publications

Variables in Male	Frequency	Variables in Female	Frequency
Sexual Behavior	214	Sexual Behavior	3
HIV Screening	107	G-spot Exploration	2
Condom Use	91	Sexual Self-Label	2
HIV-testing Behavior	75	Breast health	2
Syphilis Screening	69	Gender role	2
Drug Use	65	Self Esteem	2
Depression	57	Abnormalities	1
HIV Knowledge	56	Alcohol Use	1
HIV Prevalence	54	Anxiety and depression	1
Risk Behavior	43	App dating	1
Syphilis Prevalence	31	Assisted reproductive technology attitude	1
Unprotected Anal Intercourse	31	Barriers to Screening	1
Anxiety	29	Bisexual Behavior	1
Social support	29	Body image	1
Alcohol Use	26	Breast cancer knowledge	1
HIV Attitude	26	Breast Feeling	1
STI History	24	Breast Screening	1
HIV/STI Status	23	Childbearing Importance	1
HIV Stigma	20	Closeted in the Family	1
Coming Out	15	Coming Out	1
Commercial Sex	15	Condom Use	1
HIV Status	15	Depression	1
MSM Behavior	15	Depression-Happiness	1
MSW Behavior	15	Dispositional Hope	1
Service Utilization	14	Double Marginalisation	1
HPV Screening	13	Empathizing-Systemizing	1
Intimate partner violence	13	Family Support	1
HIV Incidence	12	Friends Support	1
Quality of life	12	Gender identity	1
Social Network	12	Health beliefs	1
Condomless anal intercourse	11	Information exposure	1
HIV Influencing Factor	11	Internalized Heterosexism	1
School Bullying Experience	10	Lesbian culture	1
Community engagement	9	Lesbian Identity	1
Cyberbullying Experience	9	Life Satisfaction	1
Self-esteem	9	Mental health	1
PrEP Knowledge	8	Online relationship	1
Sexual partner	8	Partner support	1
Willingness to use PrEP	8	Redefinition of Marriage	1
…		…	

The word clouds of health-related variables used have been created using Word Cloud Art Creator (https://wordart.com/).

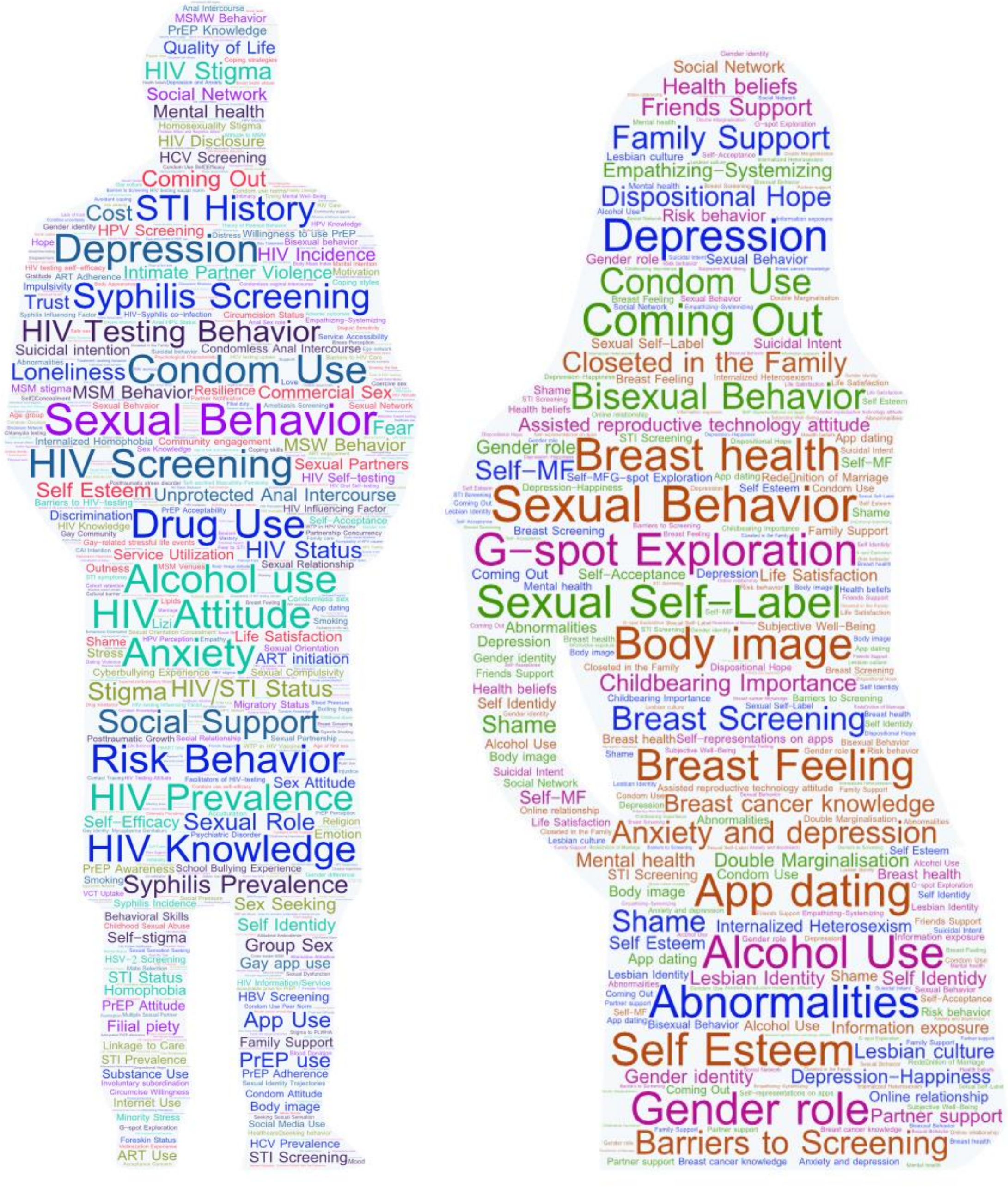

Figure 3-4 Word clouds of variables used in male and female populations
